# Direct, Asymmetric, and Stereodivergent Reactions of *N*‑Azidoacetyl Thioimides with Aromatic Acetals Catalyzed by Chiral Nickel(II) Complexes. An Approach to the Synthesis of *syn*- and *anti-*β‑Alkoxy-α-amino Acids

**DOI:** 10.1021/acs.joc.5c00390

**Published:** 2025-06-17

**Authors:** Miguel Mellado-Hidalgo, Joan Conejos-Jalencas, Saúl F. Teloxa, Andrea Suárez-Herrera, Luke McCall, Anna M. Costa, Pedro Romea, Fèlix Urpí, Gabriel Aullón, Cristina Puigjaner

**Affiliations:** a Department of Inorganic and Organic Chemistry, Section of Organic Chemistry, Institut de Biomedicina de la Universitat de Barcelona, 16724 Universitat de Barcelona, Carrer Martí i Franqués 1−11, Barcelona, Catalonia 08028, Spain; b Department of Inorganic and Organic Chemistry, Section of Inorganic Chemistry, Institut de Química Teòrica i Computacional de la Universitat de Barcelona, 16724 Universitat de Barcelona, Carrer Martí i Franqués 1−11, Barcelona, Catalonia 08028, Spain; c X-ray Diffraction Unit, CCTiUB, 16724 Universitat de Barcelona, Carrer Solé i Sabarís 1−3, Barcelona, Catalonia 08028, Spain

## Abstract

A new approach to the synthesis of all of the potential stereoisomers of β-alkoxy-α-amino acids derived from phenylalanine, tyrosine, tryptophan, and other β-aryl counterparts is reported. Such a method hinges on direct and asymmetric Lewis acid-mediated aldol-like reactions of *N*-azidoacetyl thioimides with dialkyl acetals from aromatic aldehydes catalyzed by chiral nickel­(II) complexes. This produces at will both the corresponding *syn* and *anti* adducts, which can be smoothly converted into enantiomerically pure intermediates such as dipeptide fragments. In turn, computational calculations have unveiled clues for a better understanding of such a stereocontrolled carbon–carbon bond-forming transformation.

## Graphical abstract

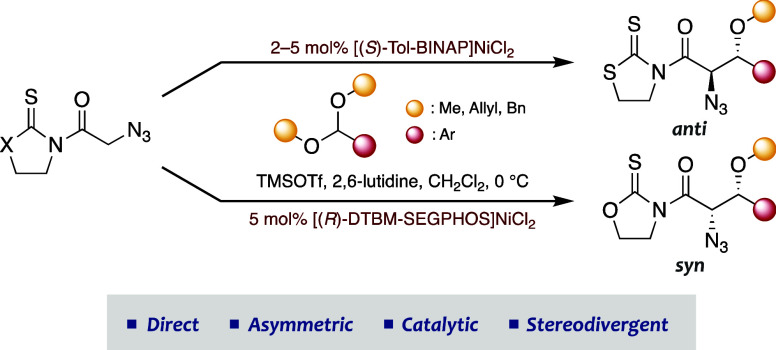

## Introduction

β-Oxygenated nonproteinogenic α-amino acids derived from phenylalanine, tyrosine, tryptophan, and other β-aryl counterparts are found in a wide array of biologically active cyclopeptides such as cyclomarins,^1^ cyclodepsipeptides such as callipeltins,^2^ papuamides,^3^ stellatolides,^4^ or swinhopeptolides,^5^ and structurally complex glycopeptide antibiotics such as vancomycin^6^ or avoparcins^7^ (Figure 1). Interestingly, the oxygenated functional group at the β-position is variable, with the alcohol (free or as part of a glycosidic bond) and the methyl ether being the most common forms. Such a structural variety has triggered the development of manifold synthetic sequences to get access to both their *syn* and *anti* stereoisomers,^8^ but there is still a lack of a general method to obtain at will any of the above-mentioned amino acids in a stereocontrolled manner.^9,10^

**Figure 1:**
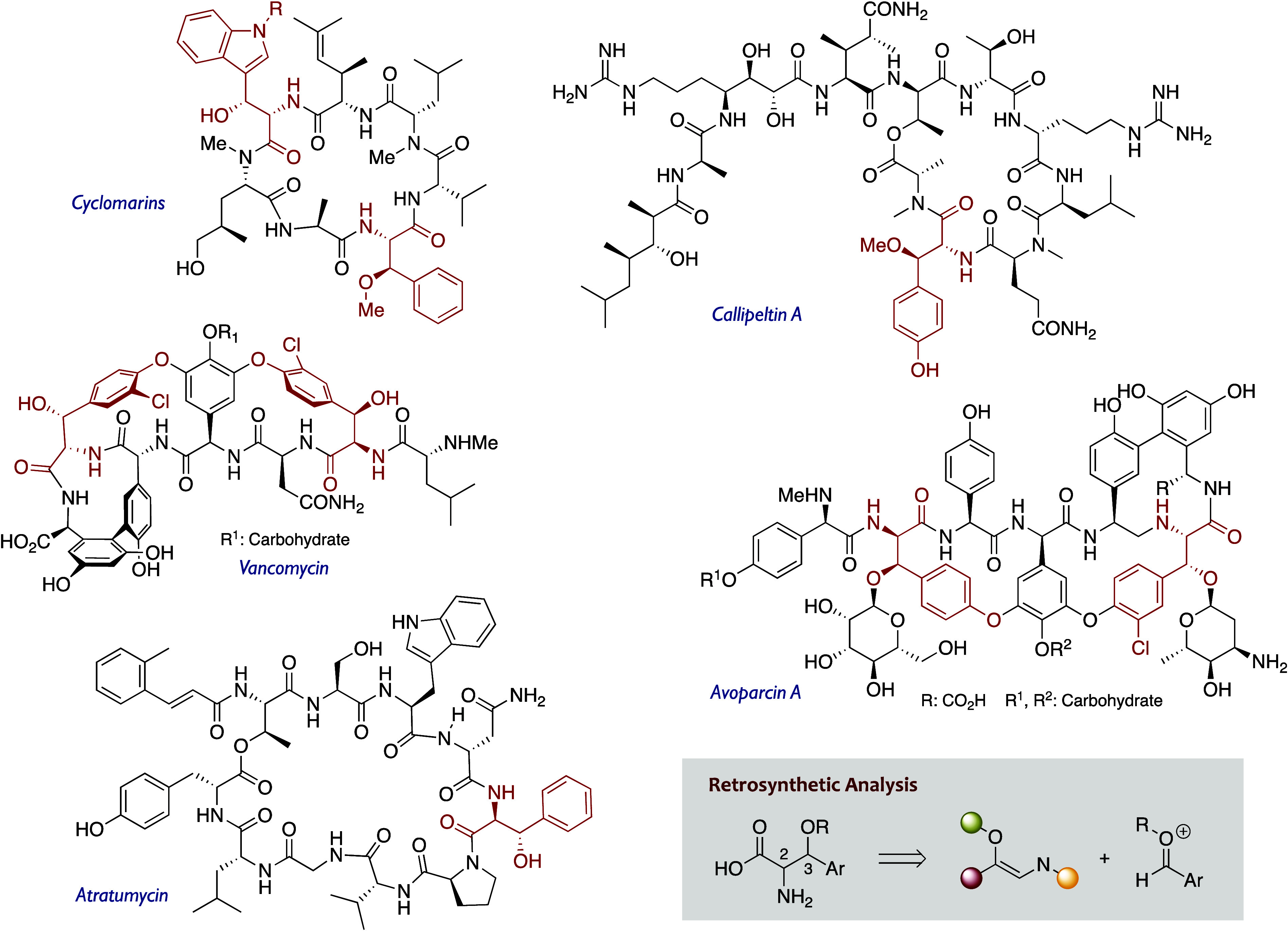
Cyclopeptides, cyclodepsipeptides, and glycopeptide antibiotics containing β-oxygenated α-amino acids.

One of the most intuitive approaches to address such a challenge takes advantage of the retrosynthetic analysis depicted in Figure 1, which involves the stereoselective construction of the C2–C3 bond through the aldol reaction of a glycine derivative.^11^ Despite the simplicity of the proposal, direct, catalytic, and asymmetric aldol reactions of glycine donors with aromatic aldehydes to provide both *syn* and *anti* stereoisomers remain elusive. Apart from the reaction conditions and the catalyst thereof, the key point to success involves the appropriate choice of the α-amino surrogate. Indeed, glycinate-derived Schiff bases proved unsuitable for aldol additions to aromatic aldehydes.^12,13^ Better results have been achieved from 5-alkoxyoxazoles as latent enolates, α-isothiocyanate esters and imides, or α-isocyanoacetate esters.^14^ Unfortunately, the resulting aldol compounds contain the α-amino and β-hydroxy functions protected with a single group, which hinders further manipulations. Aside from them, azide, a well-known masked amino group,^15^ stands as an appealing option to carry out the stereoselective construction of the carbon–carbon bond and enables selective transformations of the resultant aldol products.^16^ In this context, pioneering studies by Kumagai, Shibasaki et al. established the suitability of the direct aldol reaction of α-azido 7-azaindolinylacetamide and aromatic aldehydes catalyzed by a chiral (*R*,*R*)-Ph-BPE copper­(I) complex to obtain *syn* enantioenriched β-hydroxy-α-azido carboxylic acid derivatives (eq 1 in Scheme 1).^17^ Remarkably, parallel reactions of *ortho*-substituted aromatic aldehydes catalyzed by the same (*R*,*R*)-Ph-BPE or (*R*)-Xyl-BINAP copper­(I) complexes produced the *anti* and the complementary *syn* derivatives, respectively (eqs 2 and 3 in Scheme 1). In turn, we have recently disclosed highly stereoselective TIPSOTf–mediated aldol reactions of α-azidoacetyl-1,3-thiazolidine-2-thione with aromatic aldehydes catalyzed by a chiral nickel­(II) complex that yield enantiomerically pure *anti*-β-silyloxy-α-azido derivatives (eq 4 in Scheme 1).^18^ Lessons learned from previous examples and our own experience suggested^19–21^ that oxocarbenium intermediates bearing sterically unhindered groups might lead to both *syn* and *anti* aldol derivatives (Figure 1).^22^ Particularly, we envisaged that the Lewis acid-mediated aldol-like addition of α-azidoacetyl thioimides to acetals catalyzed by chiral nickel­(II) complexes might provide straightforward access to both *syn*- and *anti*-β-alkoxy-α-amino acids, with the allyl and the benzyl derivatives fitting precursors of the corresponding β-hydroxy-α-amino acids (Figure 1). Herein, we document a stereodivergent approach in which TMSOTf-mediated aldol-like reactions of α-azidoacetyl thioimides with a wide array of dialkyl acetals of aromatic aldehydes furnish the corresponding *anti*- or *syn*-β-alkoxy-α-azido derivatives depending on the catalyst and the thioimide employed (eq 5 and eq 6 in Scheme 1). An initial version of this work was deposited in ChemRxiv on February 11, 2025.^23^

**Scheme 1:**
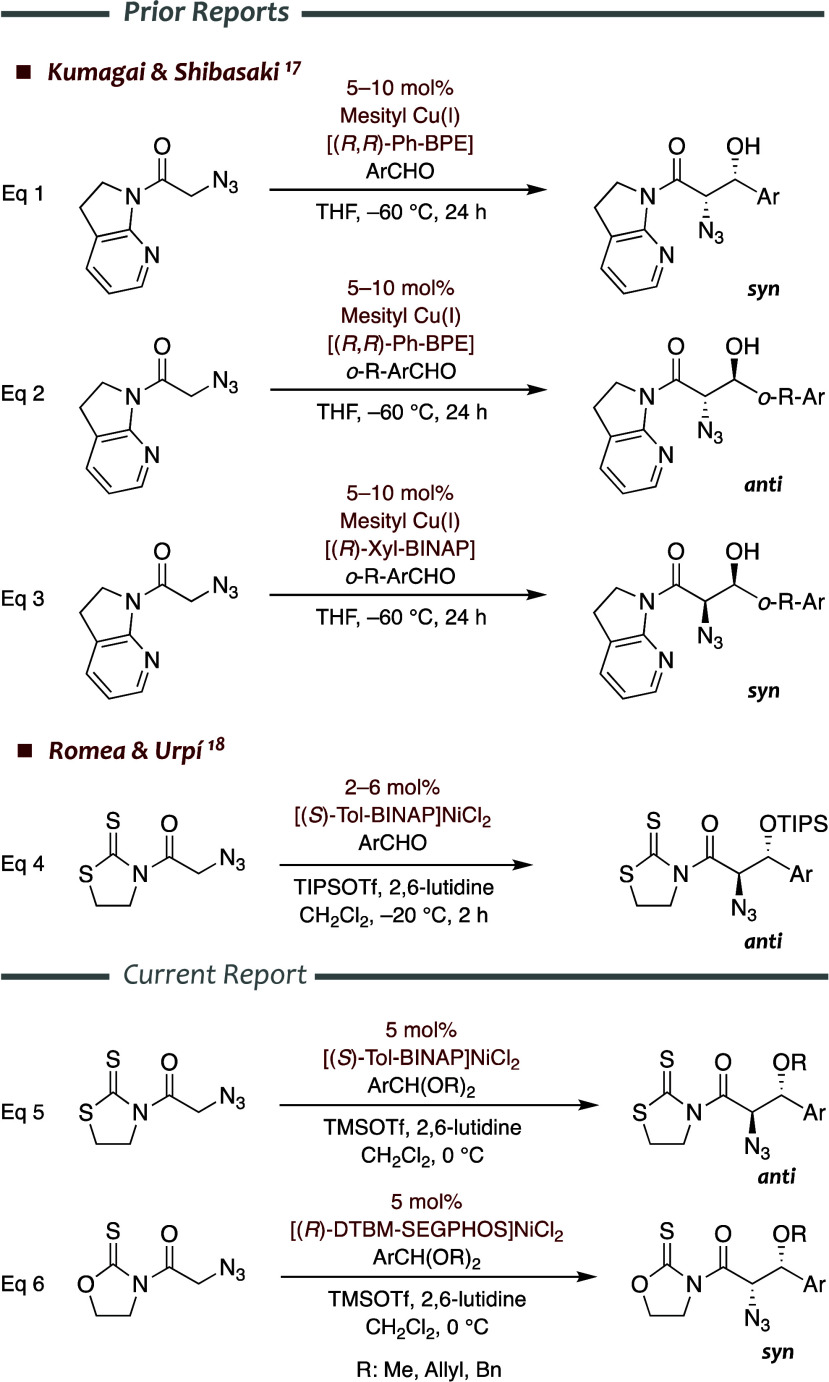
Direct and Asymmetric Aldol Reactions of α-Azido Carboxylates with Aromatic Aldehydes Catalyzed by Chiral Metal Complexes

## Results and Discussion

### Preliminary Results

Since the feasibility of the reaction was warranted by previous studies on the direct and chiral auxiliary-mediated aldol-like reaction of *N*-azidoacetyl thioimide **1** with acetals leading to *anti* adducts **2**, we initially tested the influence of achiral thioimides in parallel direct TESOTf-mediated-additions to model *p*-anisaldehyde dimethyl acetal (**a**) catalyzed by (Me_3_P)_2_NiCl_2_ (Scheme 2). To our surprise, six-membered cyclic *N*-azidoacetyl thioimides proved to be unstable, which prevented their use in further studies. Thus, we only assessed the reactivity of five-membered thioimides, namely, *N*-azidoacetyl-1,3-thiazolidine-2-thione and 1,3-oxazolidine-2-thione (**3** and **4**, respectively, in Scheme 2).^24^ The results fulfilled our expectations, and *anti* adducts **5a** and **6a** were isolated in excellent yields using 2 mol % of an achiral nickel­(II) chloride complex (Scheme 2). It is worth noting that the diastereoselectivity (dr 93:7) attained from *N*-azidoacetyl thiazolidinethione **3** was close to that imparted by chiral thioimide **1**, whereas that from the oxazolidinethione counterpart **4** was slightly lower (dr 86:14).

**Scheme 2:**
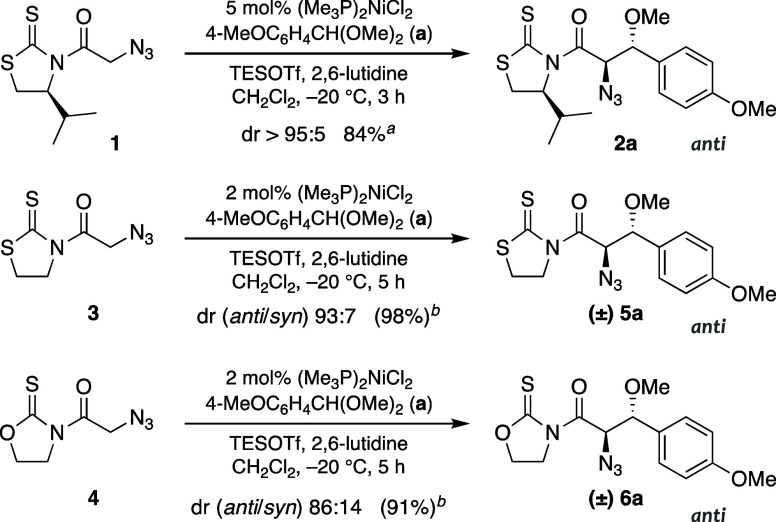
Direct TESOTf-Mediated Aldol Reactions of α-Azidoacetyl Thioimides with Anisaldehyde Dimethyl Acetal (a) Catalyzed by (Me_3_P)_2_NiCl_2_

a Isolated yield of pure *anti* diastereomer **2a**.
b Isolated overall yield of racemic *anti* and *syn* diastereomers.

Encouraged by these results, we next scrutinized the aldol-like TESOTf-mediated reaction of *N*-azidoacetyl thiazolidinethione **3** with anisaldehyde acetal **a** catalyzed by a wide array of nickel­(II) complexes from chiral diphosphine ligands (**L1**–**L13**).^25^ The results summarized in Table 1 allowed for certain conclusions to be drawn.

**Table 1:**
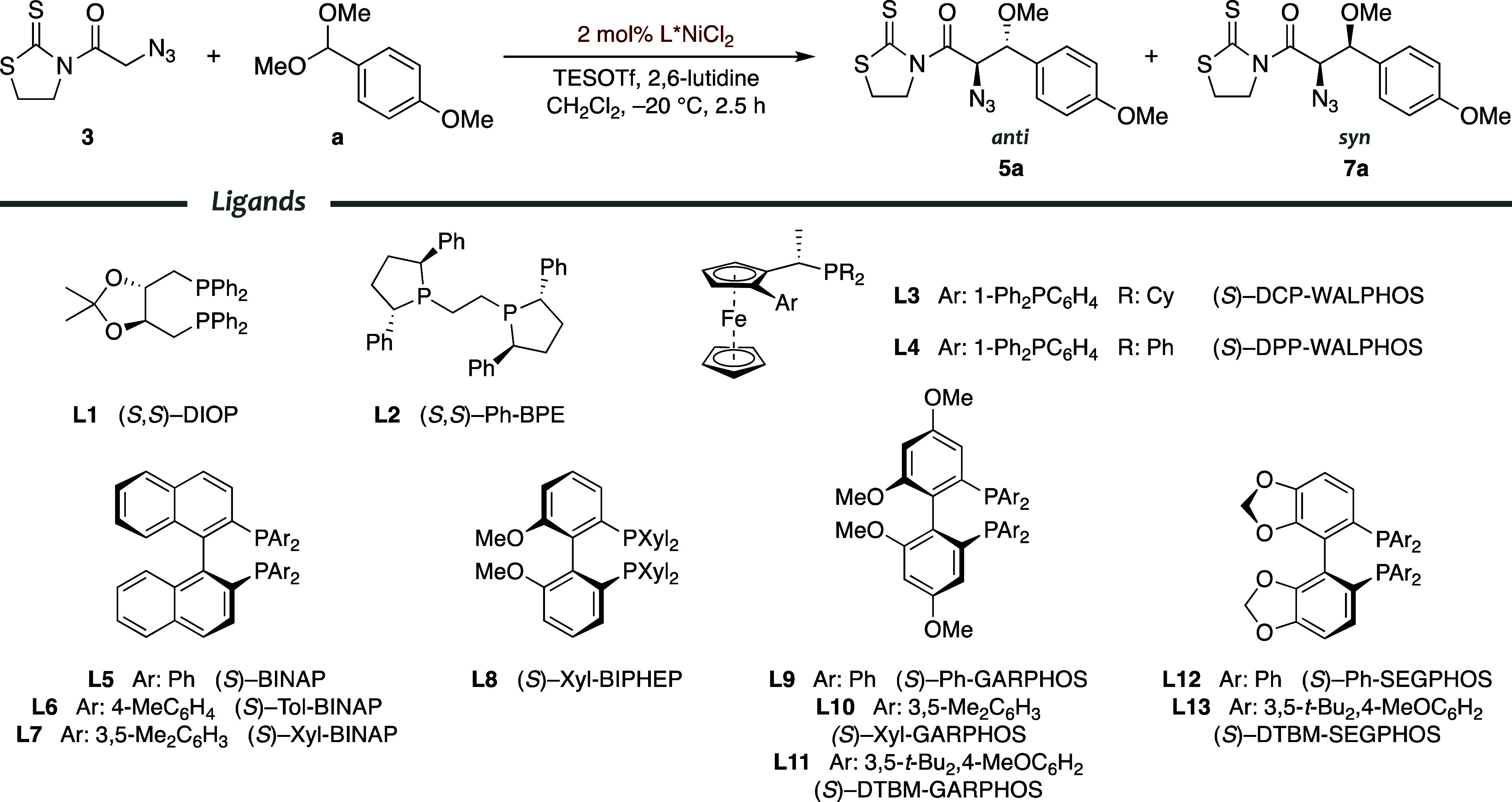
Influence of the Chiral Catalyst on the Addition of Thioimide **3** to Dimethyl Acetal **a**

entry	ligand	L*	dr **5a**:**7a**(*anti/syn*)^a^	*ee* (%)^b^	yield (%)^c^
1	**L1**	(*S*,*S*)-DIOP			
2	**L2**	(*S*,*S*)-Ph-BPE	55:45	nd	20
3	**L3**	(*S*)-DCP-WALPHOS	58:42	nd	10
4	**L4**	(*S*)-DPP-WALPHOS	59:41	nd	10
5	**L5**	(*S*)-BINAP	81:19	99	68
6	**L6**	(*S*)-Tol-BINAP	81:19	99	80
7	**L7**	(*S*)-Xyl-BINAP	82:18	96	84
8	**L8**	(*S*)-Xyl-MeOBIPHEP	80:20	98	78
9	**L9**	(*S*)-Ph-GARPHOS	82:18	98	61
10	**L10**	(*S*)-Xyl-GARPHOS	82:18	98	63
11	**L11**	(*S*)-DTBM-GARPHOS	57:43	99	77
12	**L12**	(*S*)-Ph-SEGPHOS	82:18	98	82
13	**L13**	(*S*)-DTBM-SEGPHOS	59:41	>99	82

a Determined by 400 MHz ^1^H NMR.

b Enantiomeric excess of the major stereoisomer determined by chiral HPLC analysis. nd: not determined.

c Isolated overall yield of both stereoisomers.

Indeed, some nickel­(II) complexes provided poor diastereoselectivities and low yields, which indicated that they were totally unsuitable (Table 1, entries 1–4). These include complexes from chiral diphosphines with stereocenters, such as DIOP or Ph-BPE (**L1** and **L2**, respectively), and stereogenic planes such as WALPHOSs (**L3** and **L4**). In turn, diphosphines containing a stereogenic axis such as BINAPs (**L5**–**L7**), BIPHEP (**L8**), GARPHOSs (**L9** and **L10**), and SEGPHOS (**L12**) gave *anti*-β-methoxy-α-azido derivative **5a** with high diastereomeric ratios (dr ≥ 80:20) and excellent enantioselectivities (*ee* > 96%) in good overall yields (Table 1, entries 5–10 and 12). Importantly, the substituents (Ph, Tol, or Xyl) of the phosphine group of these ligands turned out to have a minor influence on the outcome of the reaction (compare, for instance, entries 5–7 for BINAP ligands in Table 1). Eventually, similar ligands possessing two bulky *tert*-butyl groups at the phosphine group, such as DTBM-GARPHOS (**L11**) and DTBM-SEGPHOS (**L13**), produced a mixture (Table 1, entries 11 and 13) with an outstanding enantiocontrol (*ee* 99%) in good overall yield (77–82%) but low diastereoselectivity (dr < 60:40).

These results prompted us to choose the nickel­(II) complex containing Tol-BINAP (**L6** in Table 1) as the most appropriate catalyst to continue with the optimization toward the *anti* stereoisomer, being aware that other structurally related catalysts could also be used instead. Moreover, the lower diastereoselectivity attained with the nickel­(II) complexes containing the bulky DTBM-GARPHOS and -SEGPHOS diphosphines (**L11** and **L13**, respectively) spurred us on to uncover an approach to the *syn* diastereomer. Although the stereocontrol achieved with both ligands was pretty similar, DTBM-SEGPHOS was particularly promising since it was slightly more reactive (Table 1, entries 11 and 13), so we tested its impact on the reaction with oxazolidinethione **4,** which produced *anti* stereoisomers with poorer diastereoselectivities (see Scheme 2). Indeed, we hypothesized that the shorter C–O bond distance, compared to the corresponding C–S bond, upon moving from **3** to **4** may account for the differing trends observed with both thioimides. To our pleasure, such a combination leveraged both trends to get the desired switch of the relative configuration and made *syn* aldol **8a** the major diastereomer (*anti*/*syn* 23:77) with excellent enantiocontrol in good overall yield (Scheme 3).

**Scheme 3:**
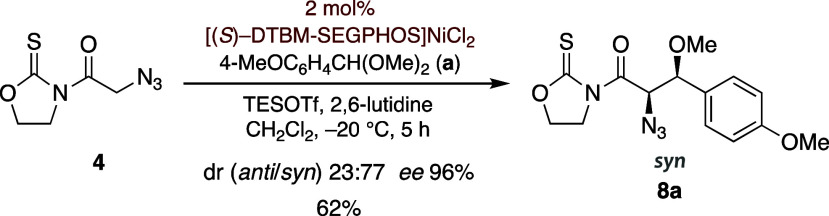
Direct TESOTf-Mediated Aldol Reactions of α-Azidoacetyl Oxazolidinethione **4** with Anisaldehyde Dimethyl Acetal (**a**) Catalyzed by [(*S*)-DTBM-SEGPHOS]­NiCl_2_

Having established the most appropriate thioimide and catalyst pairs, we examined the influence of silyl triflate. At first, the Lewis acid should not play any significant role because it only serves to create the real catalytic species, the putative nickel­(II) triflate complex, and the formation of the required oxocarbenium intermediate. However, analysis of four silyl triflates uncovered a certain impact on the conversion and, to a lesser extent, on the diastereoselectivity. Results summarized in Table 2 show that sterically hindered TIPSOTf failed to achieve full conversions of both thioimides, whereas TBSOTf led the reactions to completion but with slightly eroded diastereoselectivities (Table 2, entries 4–5 and 9–10). In turn, less bulky triflates TMSOTf and TESOTf provided identical results with regard to yield and stereocontrol (Table 2, entries 2–3 and 7–8), so the smallest TMSOTf was chosen as the Lewis acid for further studies.

**Table 2:**
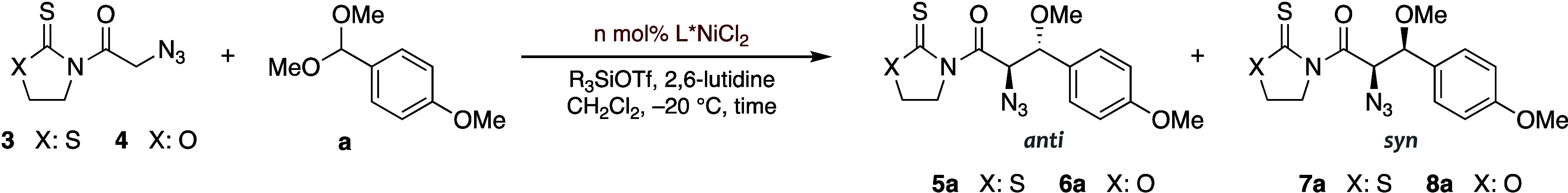
Influence of the Silyl Triflate on the Addition of Thioimides **2** and **3** to Acetal **a**

entry	thioimide	L*	*n* (mol %)	R_3_SiOTf	time (h)	conversion (%)^a^	dr (*anti*/*syn*)^a^	*ee* (%)^b^	yield (%)^c^
1	**3**	(Me_3_P)_2_	4	TESOTf	15	95	93:7		nd
2	**3**	(*S*)-Tol-BINAP	2	TESOTf	2.5	100	81:19	99	64
3	**3**	(*S*)-Tol-BINAP	2	TMSOTf	2.5	100	82:18	99	64
4	**3**	(*S*)-Tol-BINAP	2	TBSOTf	2.5	100	76:24	nd	nd
5	**3**	(*S*)-Tol-BINAP	2	TIPSOTf	2.5	75	80:20	nd	nd
6	**4**	(Me_3_P)_2_	10	TESOTf	15	83	87:13		nd
7	**4**	(*S*)-DTBM-SEGPHOS	2	TESOTf	5	100	23:77	98	67
8	**4**	(*S*)-DTBM-SEGPHOS	2	TMSOTf	5	100	24:76	97	64
9	**4**	(*S*)-DTBM-SEGPHOS	2	TBSOTf	5	100	27:73	nd	nd
10	**4**	(*S*)-DTBM-SEGPHOS	2	TIPSOTf	5	79	27:73	nd	nd

a Determined by 400 MHz ^1^H NMR.

b Enantiomeric excess of the major stereoisomer determined by chiral HPLC analysis.

c Isolated overall yield of both stereoisomers. nd: not determined.

Eventually, the influence of the temperature was also checked. Indeed, the TMSOTf-mediated additions of **3** and **4** to acetal **a** catalyzed, respectively, by chiral nickel­(II) complexes containing Tol-BINAP and DTBM-SEGPHOS diphosphines were conducted at 0 °C. The results were basically the same as those obtained at −20 °C, although slight losses of yield and stereocontrol were occasionally observed.

Altogether, the former studies indicated that the additions of thioimides **3** and **4** to acetal **a** could be carried out with 1.1 equiv of the acetal, 1.3 equiv of TMSOTf, and 1.5 equiv of 2,6-lutidine at 0 °C in CH_2_Cl_2_ (0.5 M) in the presence of [Tol-BINAP]­NiCl_2_ (for the *anti* diastereomer) or [DTBM-SEGPHOS]­NiCl_2_ (for the *syn* diastereomer).

### Scope

After optimizing the experimental conditions with acetal **a**, we focused our attention on the scope of the reaction. Initially, we evaluated the addition of thiazolidinethione **3** to dialkyl acetals from 4-oxygenated benzaldehydes, which could be viewed as precursors for tyrosine derivatives, using 5 mol % [(*S*)-Tol-BINAP]­NiCl_2_. Importantly, anisaldehyde-derived dimethyl, diallyl, and dibenzyl acetals, **a**–**c**, respectively, led to the corresponding enantiopure *anti* stereoisomers **5a**–**c** with high diastereoselectivities (dr ≥ 80:20), excellent enantioselectivities (*ee* 97–99%), and yields about 60% (Table 3). Reactions of structurally similar *O*-benzyloxy and *O*-silyloxy dimethyl acetals, **d** and **e**, respectively, gave enantiopure *anti* adducts **5d** and **5e** with 63–64% yields in lower, but still good, diastereoselectivities (Table 3). In addition, the position of the methoxy group plays a certain role. While enantiomerically pure *anti* adducts **5f** and **5g**, from the *meta*- and the *ortho*-methoxy dimethyl acetals, **f** and **g**, respectively, were isolated in a reasonably good yield of 50%, the diastereomeric ratios were lower than that for the *para* counterpart, and the *ortho*-acetal **g** necessitated a higher loading of catalyst (10 mol %) and a longer reaction time (Table 3). In turn, double oxygenated acetals **h**–**j** performed better, and methoxy and benzyloxy enantiopure (*ee* ≥ 98%) *anti* aldols **5h**–**j** were isolated with yields ranging from 60 to 70% (Table 3). As expected, the kinetics of less activated dimethyl acetals **k**–**m** were slower and required longer reaction times. Benzaldehyde dimethyl acetal (**m**) deserves a special mention, since the resulting enantiopure aldol adduct **5m**, which can be viewed as a precursor for phenylalanine β-methoxy derivatives, was isolated with a 58% yield (Table 3). In turn, dimethyl acetals from heteroaromatic aldehydes **n** and **o** also underwent highly stereocontrolled *anti* aldol reactions. Special attention should be paid to the good diastereomeric ratio (dr 74:26), excellent enantioselectivity (*ee* 98%), and good yield (66%) with which *anti* aldol **5o** was obtained, as it can be considered to be a derivative of the proteinogenic amino acid tryptophan (Table 3). Finally, acetals derived from aromatic aldehydes containing electron-withdrawing substituents, such as nitro or trifluoromethyl groups, are unsuitable and do not yield synthetically useful results. Regarding minor *syn* diastereomers **7**, it is worth mentioning that they are also obtained with an excellent enantiopurity, as proved in **7k** and **7m** (Table 3).

**Table 3:**
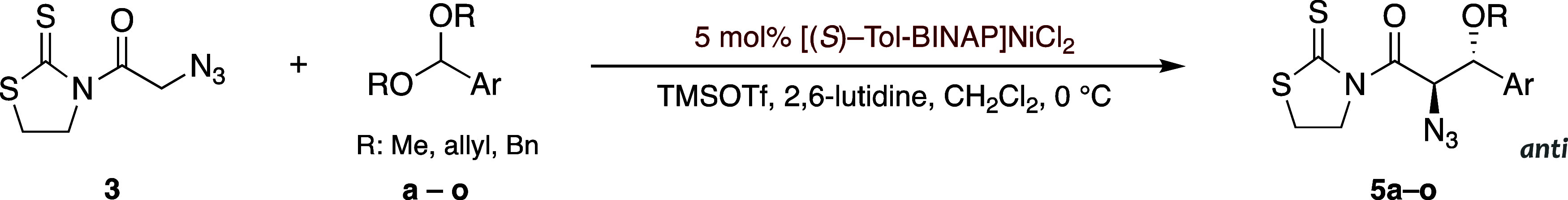

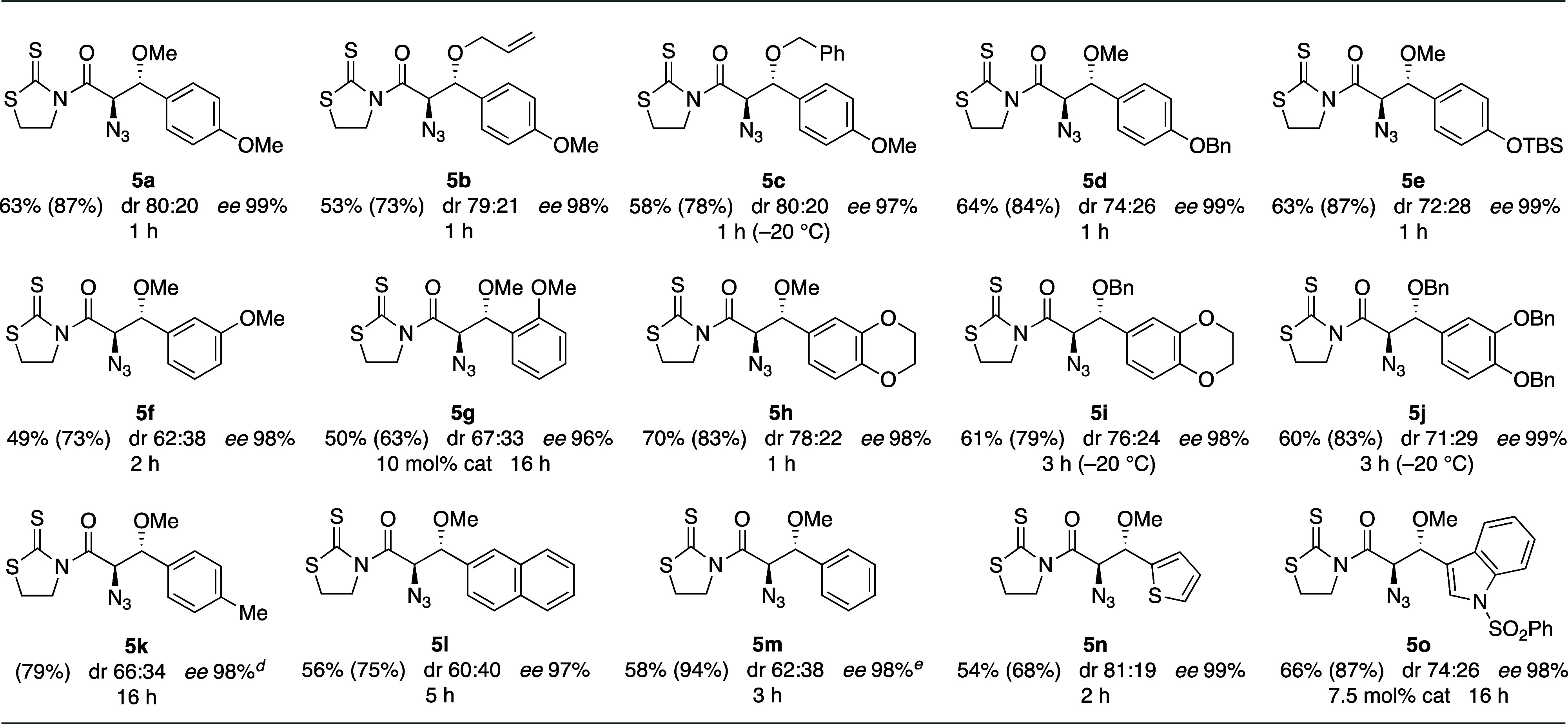
Scope of the Aldol-like inethione **3** Catalyzed by [(*S*)-Tol-BINAP]­NiCl_2_ ^a^,^b^,^c^

a Isolated yield of the *anti* diastereomer **5a**–**o** (in parentheses, overall yield).

b Diastereomeric ratio (*anti*/*syn*) determined by 400 MHz ^1^H NMR.

c Enantiomeric excess of the *anti* diastereomer **5a**–**o** determined by chiral HPLC analysis.

d The enantiomeric excess, *ee*, of minor *syn* diastereomer **7k** is 95%.

e The enantiomeric excess, *ee*, of minor *syn* diastereomer **7m** is 94%.

Having established the wide scope of the *anti* aldol-like reaction from *N*-azidoacetyl thiazolidinethione **3**, we assessed the parallel direct *syn* transformation from the related oxazolidinethione **4** catalyzed by [(*R*)-DTBM-SEGPHOS]­NiCl_2_. The results are summarized in Table 4. In this occasion, reactions of dialkyl acetals **a**–**c** from anisaldehyde proved to be puzzling. While dimethyl acetal **a** gave the *syn* aldol *ent-* **8a** with a good diastereomeric ratio (*anti/syn* 23:77), *anti* diastereomers *ent-* **6b** and *ent-* **6c** were the major components of the resultant mixtures from diallyl and dibenzyl acetals, **b** and **c**, respectively, which evidenced the subtle features that determine the stereochemical outcome of the reaction catalyzed by the DTBM-SEGPHOS nickel complex. Basically, these first results suggested that increasing the steric hindrance of the R group would favor the *anti* diastereomer (Table 4). Conversely, the bulk of the oxygenated group at the aromatic ring played an opposite role and the diastereomeric ratios from acetals **d** and **e**, containing a benzyloxy or a silyloxy group, respectively, at *para* position, were slightly better than that from **a**; a case in point is *syn* aldol *ent*-**8e** (*anti/syn* 17:83), which was isolated in an enantiomerically pure form (*ee* 99%) with a remarkable 70% yield (Table 4). As for thiazolidinethione **3**, reactions of *meta-* and *ortho-*methoxy dimethyl acetals **f** and **g** led to the corresponding *syn* aldols *ent*-**8f** and *ent*-**8g**, respectively, with 55–61% yields provided that necessary adjustments of the catalyst loading and the reaction time were carried out (Table 4). Interestingly, the influence of the steric bulk of the R and the aryl groups was also manifested in the case of double oxygenated acetals **h**–**j**. Indeed, dibenzyl acetal **i** containing small groups on the aromatic ring produced *anti* aldol *ent-* **6i** with a good diastereomeric ratio (*anti/syn* 70:30), whereas dibenzyl acetal **j** bearing benzyl protecting groups gave *syn* aldol *ent-* **8j** with a better diastereoselectivity (*anti/syn* 14:86) and a 63% yield (Table 4). Regarding the poorly activated dimethyl acetals **k**–**m**, they proved to be less reactive but enantiomerically pure *syn* aldols *ent*-**8k**–**8m** were isolated with 48–62% yields after longer reaction times. Finally, the behavior of the dimethyl acetals from heteroaromatic aldehydes matched the former trends: small thiophene-derived acetal **n** produced an almost equimolar mixture of *anti* and *syn* aldol diastereomers, where the larger indole-derived acetal **o** gave the enantiomerically pure (*ee* 99%) *syn* aldol *ent* **-8o** with an outstanding diastereomeric ratio (*anti/syn* 4:96) and a 90% yield (Table 4). Finally, as previously noted, the minor *anti* diastereomers are also obtained with excellent stereocontrol, as demonstrated by the results for *ent*-**6f**, *ent*-**6g**, and *ent*-**6m**, which achieve up to *ee* 99% (Table 4).

**Table 4:**
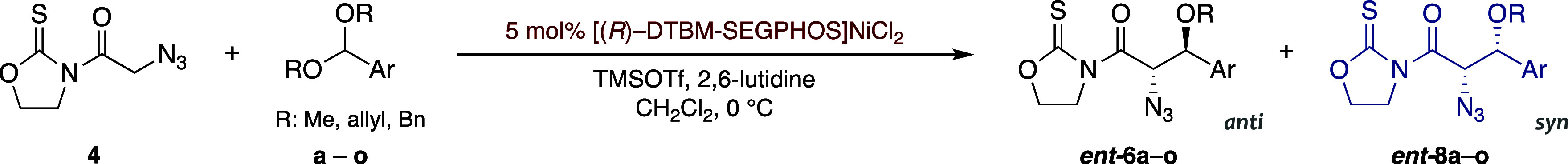

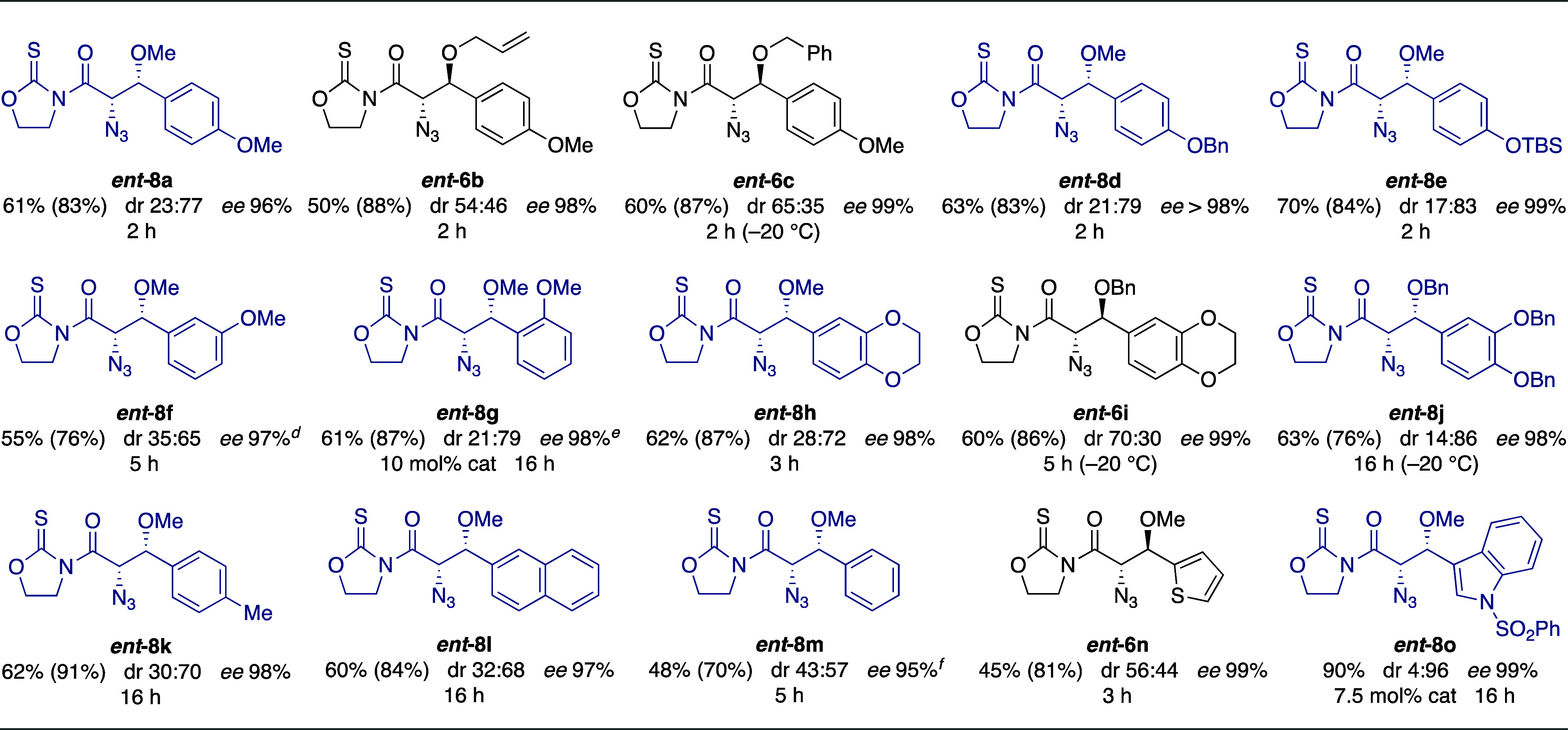
Scope of the Aldol-like Reaction from Oxazolidinethione **4** Catalyzed by [(*R*)-DTBM-SEGPHOS]­NiCl_2_ ^a^ ^‑^ ^b^ ^‑^ ^c^

a Isolated yield of the majo*r* diastereomer (in black, major *anti* stereoisomer, in blue major *syn* stereoisomer) and overall yield in parentheses.

b Diastereomeric ratio (*anti*/*syn*) determined by 400 MHz ^1^H NMR.

c Enantiomeric excess of the major diastereomer determined by chiral HPLC analysis.

d The enantiomeric excess, *ee*, of minor *anti* diastereomer *ent*-**6f** is 96%.

e The enantiomeric excess, *ee*, of minor *anti* diastereomer *ent*-**6g** is 99%.

f The enantiomeric excess, *ee*, of minor *anti* diastereomer *ent*-**6m** is 98%.

The opposite stereochemical outcome of the reaction depending on the R group of the acetal and the steric bulk of the substituents of the aromatic ring raised concerns on the structural trends that govern such transformations regarding the electrophilic partner. Indeed, the *anti* configuration of aldols **5** in Table 3 stems from the proper combination of the thioimide (thiazolidinethione) and the chiral nickel catalyst ([Tol-BINAP]­NiCl_2_), with the electrophile playing a secondary role. However, the steric bulk of the oxocarbenium intermediate was apparently crucial in parallel reactions involving oxazolidinethione **4** and [DTBM-SEGPHOS]­NiCl_2_, so we decided to closely examine model combinations of the alkyl group of the acetal.

The results summarized in Table 5 confirm such trends. Indeed, the proportion of the *syn* diastereomer from dimethyl acetals **a**, **d**, and **e** rose as the steric bulk of the protecting group (PG) increased (Table 5, entries 1–3). The same bias was observed in diallyl acetals **b**, **p**, and **q** (Table 5, entries 4–6) or dibenzyl acetals **c**, **r**, and **s** (Table 5, entries 7–9). Otherwise, *anti*-β-alkoxy-α-azido adducts become the main diastereomer in reactions catalyzed by [DTBM-SEGPHOS]­NiCl_2_ complexes provided that the electrophilic partner, the oxocarbenium intermediate, contains a bulky R alkyl group and a small phenol protecting group PG. For the sake of clarity, data of Table 5 is represented in Figure 2 using red columns to highlight the combinations in which the relative configuration of the major diastereomer is *anti* while the green ones indicate those favoring the *syn* stereoisomers. It is thus clear that the bulkier R is (R: Bn > Allyl > Me), the higher the proportion of the *anti* diastereomer, whereas sterically hindered protecting groups PG (PG: TBS > Bn > Me) show the opposite bias, favoring the *syn* diastereomers.

**Table 5:**
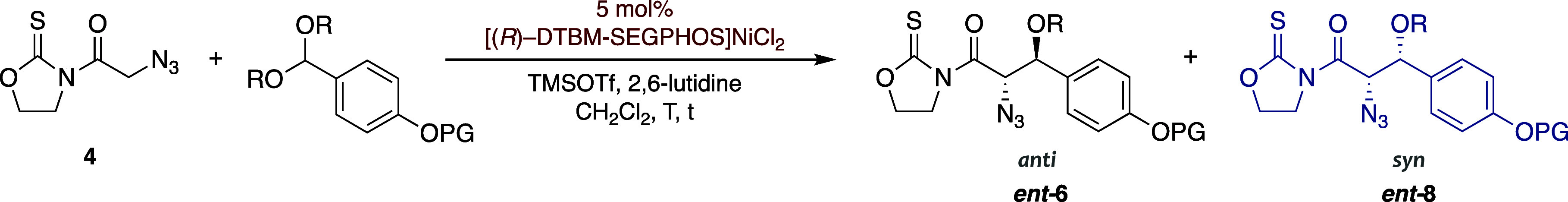

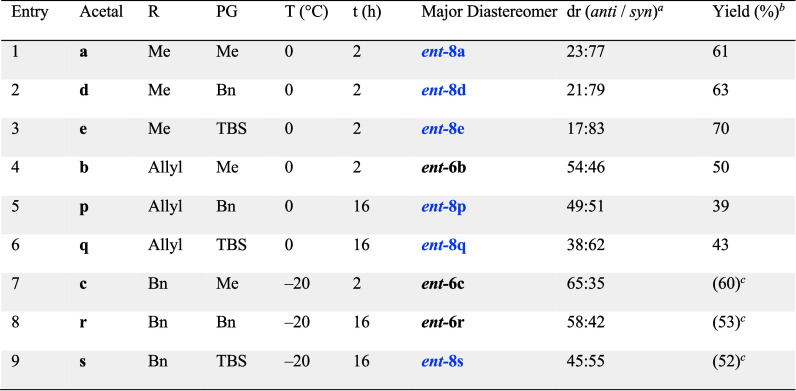
Influence of the Alkyl (R) and the Protecting Group (PG) of the *p-*Hydroxybenzaldehyde Acetal

a Determined by 400 MHz ^1^H NMR.

b Isolated yield of the major diastereomer (in black, major *anti* stereoisomer, in blue major *syn* stereoisomer).

c Isolated overall yield.

**Figure 2:**
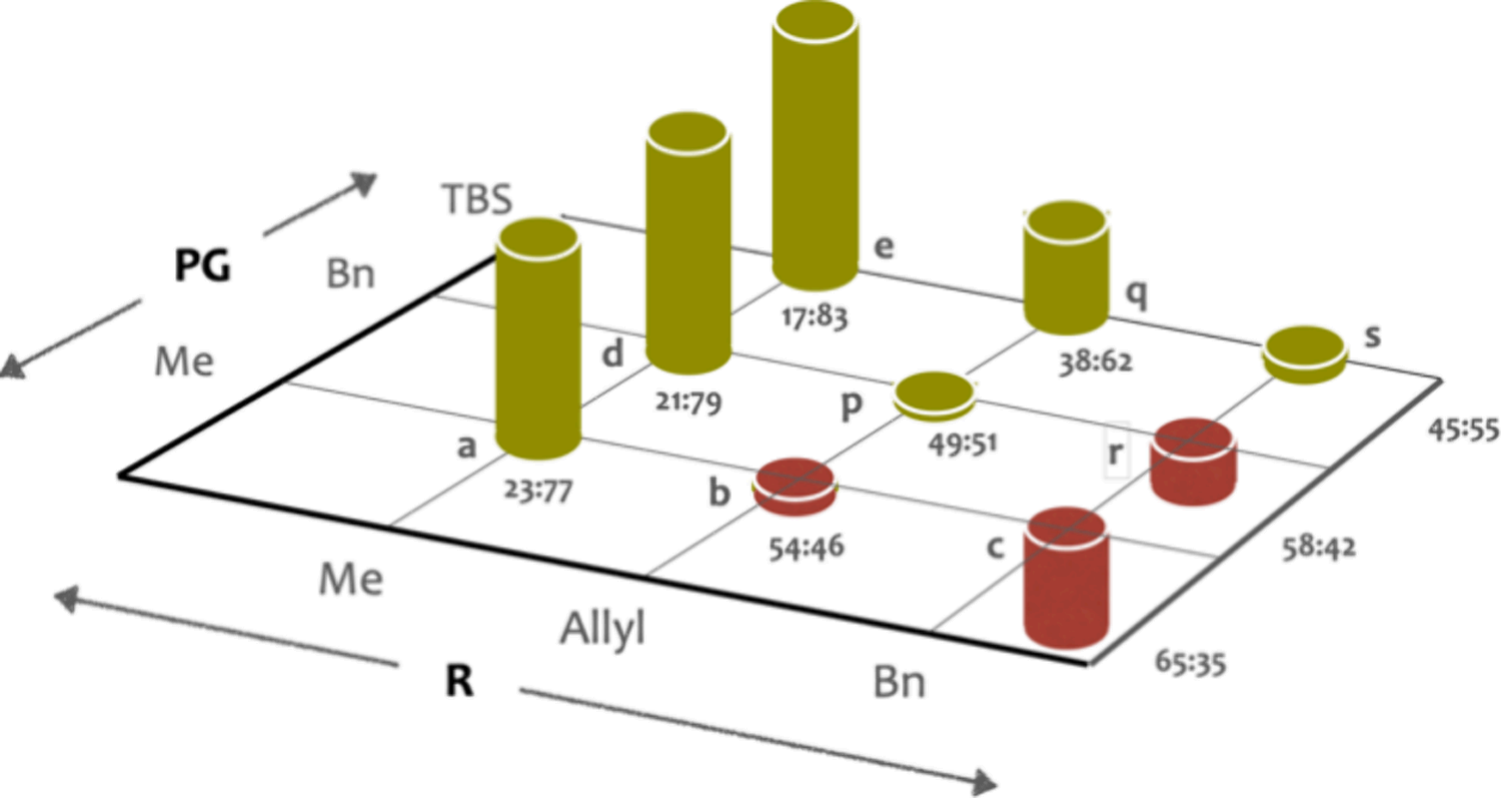
Diastereomeric ratios (*anti*/*syn*) of the reactions of **4** with dialkyl acetals from protected 4-hydroxybenzaldehyde (**a**–**e**, **p**–**s**) catalyzed by [DTBM-SEGPHOS]­NiCl_2_.

All in all, a proper understanding of the stereochemical outcome of the reactions from oxazolidinethione **4** catalyzed by [DTBM-SEGPHOS]­NiCl_2_ must take into account the steric bulk of both the alkyl group and the aryl substituents of the electrophile. Such results suggest that the active site of the nickel catalyst is extremely sensitive to the steric hindrance of the electrophile, so the stereochemical outcome of the reaction can be controlled to a certain extent by the appropriate choice of R and PG groups.

### Structural Studies

Aldol-like reactions from thioimides **3** and **4** catalyzed by achiral (Me_3_P)_2_NiCl_2_ mostly produced racemic mixtures of the *anti* diastereomer. Thus, comparison of their ^1^H NMR spectra with those from the reactions catalyzed by chiral complexes revealed the relative configuration of the major diastereomer. Furthermore, a detailed analysis of the ^1^H NMR spectra of all of the aldol products indicated that ^3^ *J*_H2–H3_ coupling constants could be used as evidence of the *anti* or *syn* relative configuration. Indeed, ^3^ *J*_H2–H3_ coupling constants are consistently larger than 8.0 Hz in *anti* aldols, while they are smaller than 7.0 Hz in the *syn* counterparts (Scheme 4). Finally, treatment of aldols **5a** and *ent*-**8a** with chiral (*S*)-1-phenylethyl amine displaced the heterocycle and produced crystalline amides **9** and **10**, whose X-ray analyses allowed us to firmly establish the absolute configuration of both series of compounds (Scheme 4).

**Scheme 4:**
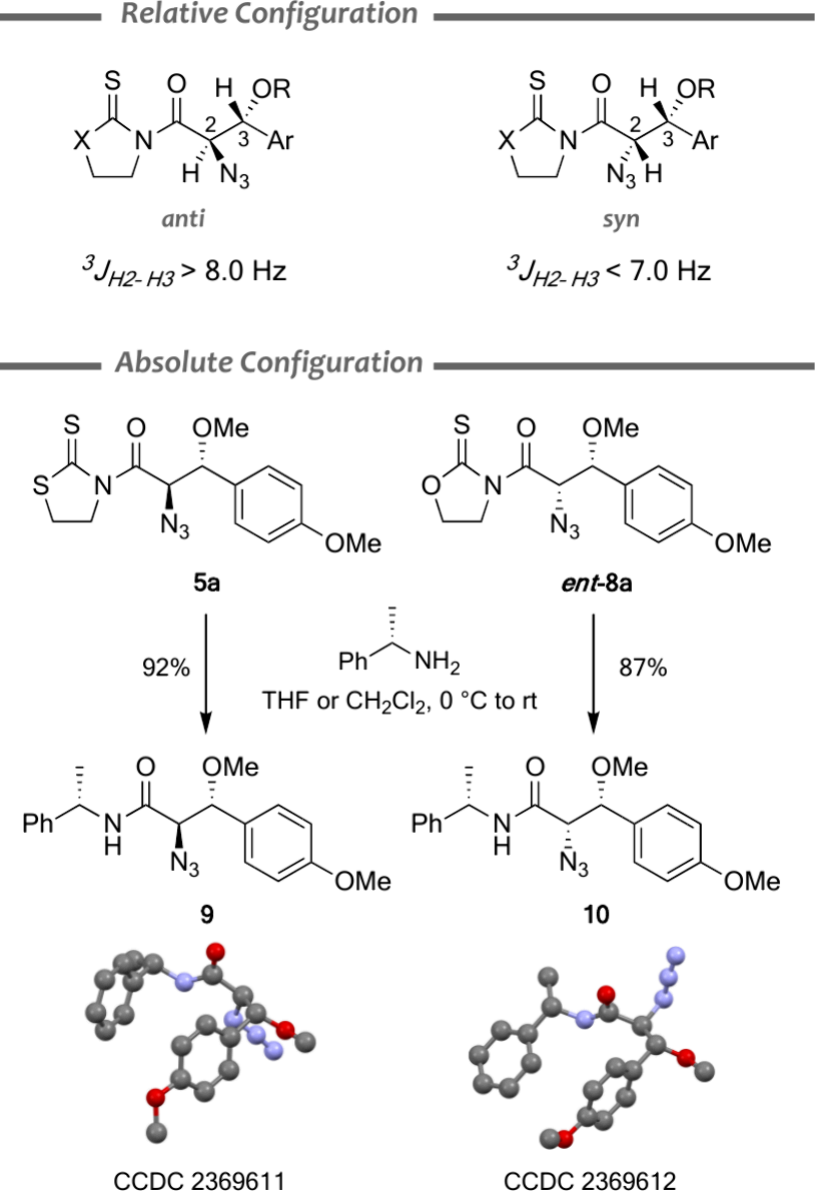
Relative and Absolute Configurations of Aldols

### Transformations

The successful displacement of the thiazolidinethione and the oxazolidinethione heterocycle to deliver a wide array of enantiomerically pure intermediates has been largely established by us and others.^19,24,26,27^ Despite such evidence and considering that the method should be able to be applied to the synthesis of peptide fragments, it was necessary to assess the use of α-amino esters as nucleophiles to remove the more reluctant oxazolidinethione heterocycle under mild experimental conditions.

Therefore, the reaction of *N*-azidoacetyl oxazolidinethione **4** with dimethyl acetal **a** catalyzed by [(*R*)-DTBM-SEGPHOS]­NiCl_2_ was scaled up to deliver enantiomerically pure (*ee* 96%) *syn* aldol *ent* **-8a** in 63% yield and the minor *anti* diastereomer *ent*-**6a** in 21% yield, which proves the robustness of the method (Scheme 5). With a reasonable amount of the *syn* adduct in hand, we evaluated the synthesis of dipeptide fragments by treatment of *ent*-**8a** with alanine and leucine esters. The results summarized in Scheme 5 proved that the oxazolidinethione scaffold can be efficiently displaced to give diastereomerically pure dipeptides **11** and **12** in 80–81% yields, which supports the use of the method for the synthesis of the peptide fragments showcased in Figure 1.

**Scheme 5:**
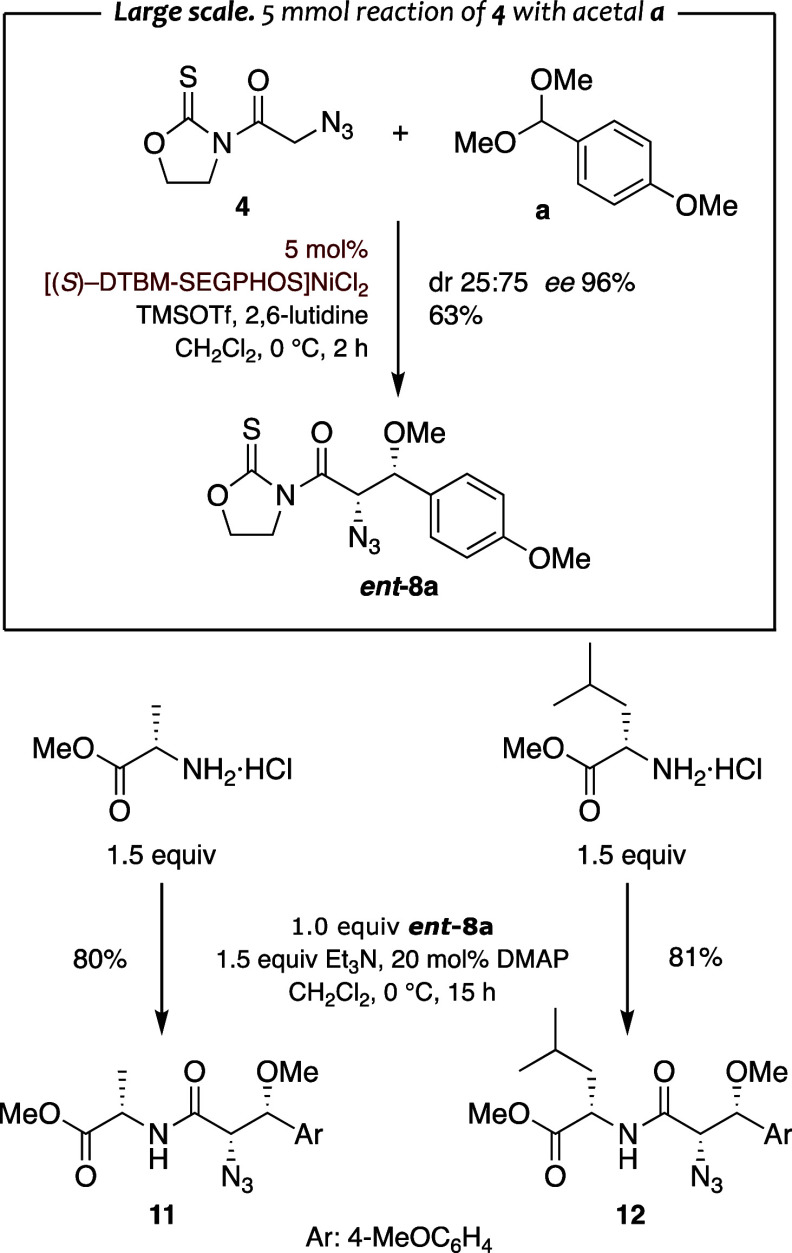
Synthesis of Dipeptide Fragments

### Computational Studies. A Mechanistic Model

Aiming to understand the intricacies of such stereodivergent reactions, we carried out a comprehensive computational study of the species and transition states involved in the overall transformation. All molecular geometries have been calculated in the singlet ground state.

Initially, we investigated the structure of the chelated nickel­(II) enolate containing the (*S*)-Tol-BINAP ligand. Our study demonstrated that **R1** and **R2** (Figure 3), two structures with identical boat conformation for the *S*,*O*-chelate and an almost planar environment for the nickel atom (*S*_SQ‑4_ ≈ 1.6, with opposite angles close to the ideal planar geometry), were the most stable. Other structures with a distorted geometry around the nickel atom (*S*_SQ‑4_ ≈ 3.5) are much less stable (see the Supporting Information). Interestingly, the envelope conformation of the ethylene fragment of the thiazolidine ring, which has less influence than the *S*,*O*-chelate conformation, determines the relative stability of **R1** and **R2**.

**Figure 3:**
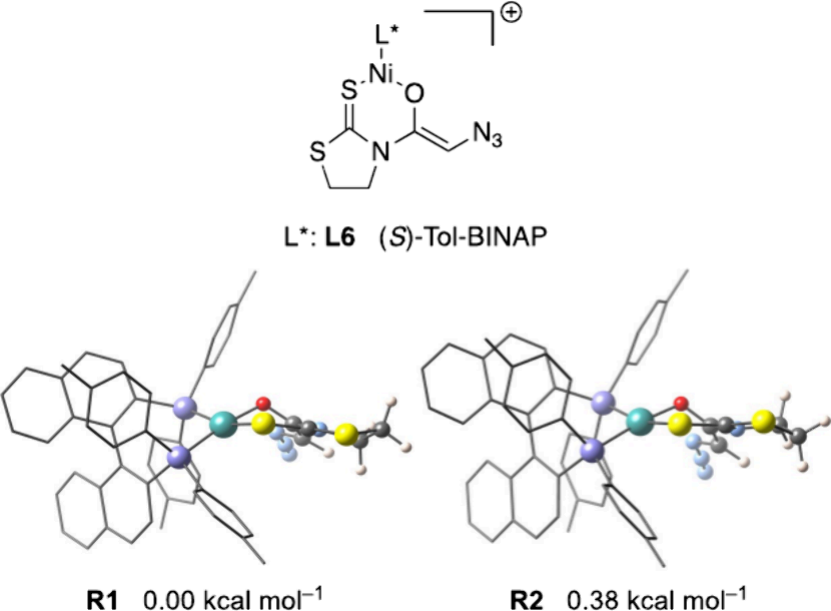
Conformations of [(*S*)-Tol-BINAP]­Ni­(II) enolate from thioimide **3**.

Then, we investigated the approach of the oxocarbenium cation from dimethyl acetal **a**, the putative electrophile, to the nucleophile, the chelated nickel­(II) enolate in both conformations, **R1** and **R2** (Figure 3). Thus, four different approaches were considered, depending on the π-face of the enolate (**A** or **B** in Figure 4) and the relative disposition of the substituents of the oxocarbenium intermediate. Calculations revealed that **B** approaches proceed through high energy transition states, as one of the tolyl groups prevents the electrophile from attacking the *Si* π-face of the enolate; therefore, their contribution to the overall reaction is negligible. Then, the most stable open transition states arise from **A** approaches to **R1** and **R2** (**TS1-A1** and **TS2-A1**, respectively) and produce the same *anti* stereoisomer **5a**, whereas the transition state to the *syn* diastereomer **7a** (**TS1-A2**, (+0.77 kcal mol^–1^) is predicted to arise from the alternative **A** approach to conformation **R1** (Figure 4). When comparing both transition states, **TS1-A1** is found to be more stable than **TS1-A2**, as the bulkier substituent, the aryl group, is positioned in an antiperiplanar arrangement relative to the carbon framework, thus minimizing steric interactions. Such results predict an 86:14 *anti*/*syn* diastereomeric ratio of a single enantiomer in excellent accordance with the experimental results (dr 80:20, *ee* 99%).

**Figure 4:**
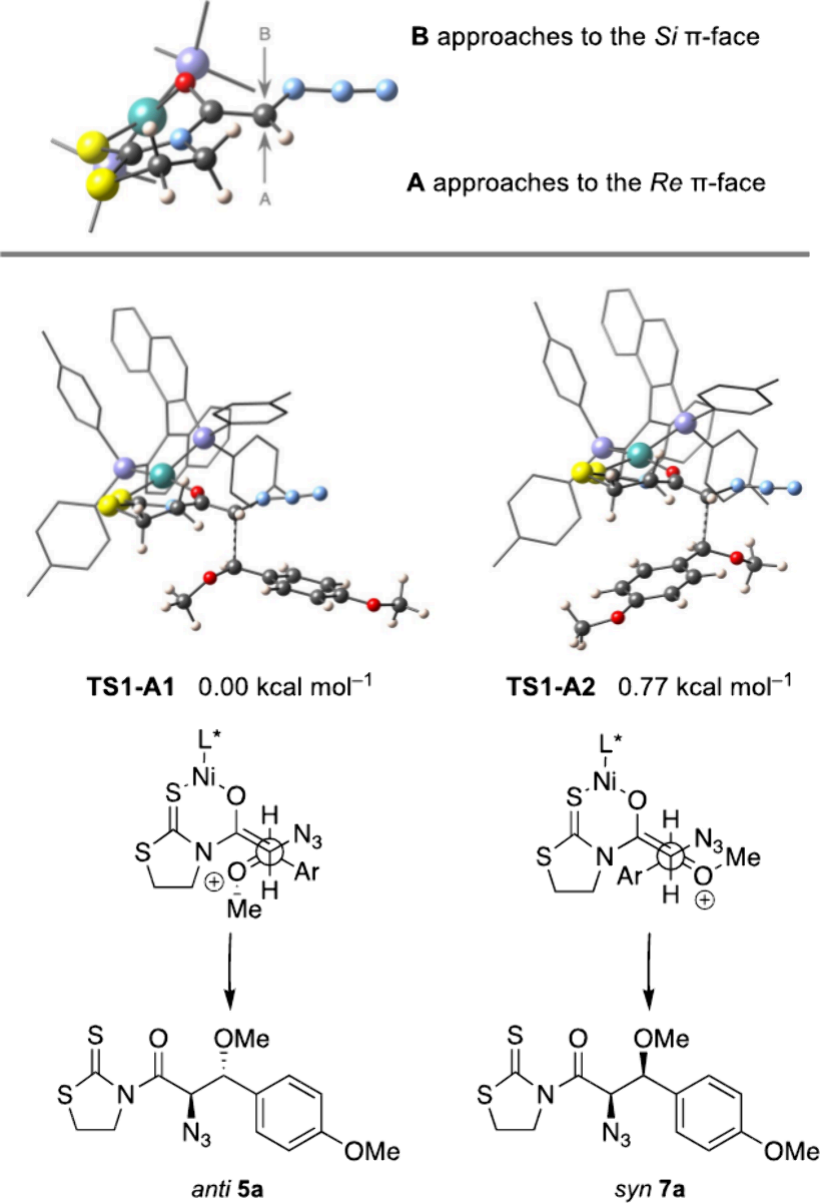
Approaches of the oxocarbenium intermediate from acetal **a** to conformation **R1** of the [(*S*)-Tol-BINAP]­Ni­(II) enolate from thioimide **3**.

We next applied a similar analysis to the reactions of thioimide **4** catalyzed by the (*R*)-DTBM-SEGPHOS nickel complex. The resultant enolate also shows a remarkable preference for structures possessing a planar geometry for the nickel atom (*S*_SQ‑4_ ≈ 2.8 and 3.0), which makes **R1** and **R2** (Figure 5) the most stable conformations. It is worth highlighting that the five-membered oxazolidinethione ring is now almost planar due to the presence of the oxygen atom instead of the sulfur in the thiazolidinethione counterpart.

**Figure 5:**
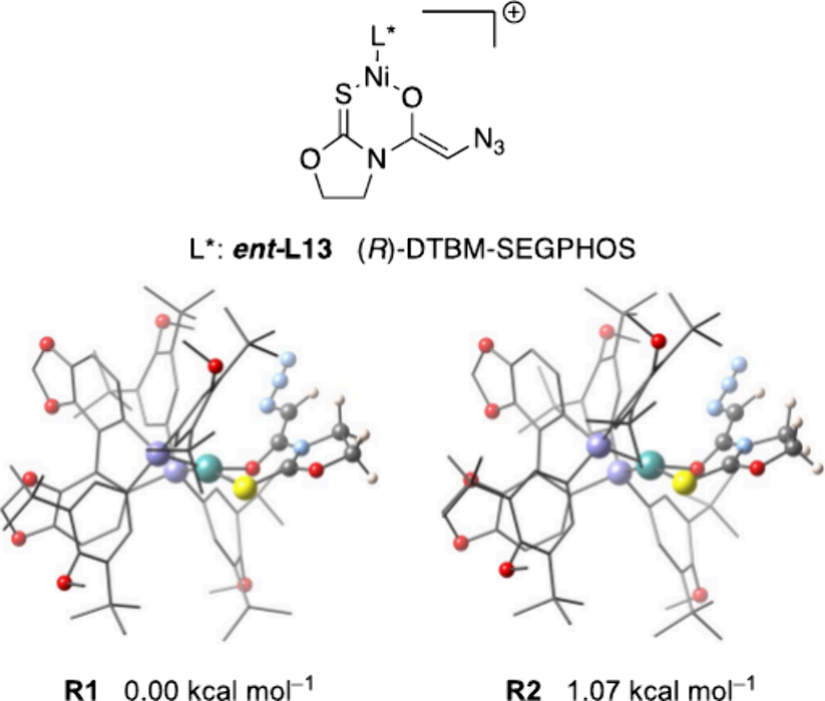
Conformations of [(*R*)-DTBM-SEGPHOS]­Ni­(II) enolate from thioimide **4**.

Having established the most stable structures of the chelated nickel enolate with the DTBM-SEGPHOS ligand, we evaluated the open transition states in which they can be involved. A thorough analysis of **R1** and **R2** showed that approaches of the oxocarbenium intermediate from dimethyl acetal **a** to the *Re* π-face of the enolate (**B** in Figure 6) were utterly impossible due to the stringent steric hindrance imparted by the phosphine substituents (see the Supporting Information). Therefore, we considered only approaches to the *Si* π-face of the enolate (**A** in Figure 6) from **R1** and **R2**, with the result of eight open transition states in a range of 6 kcal·mol^–1^. Unlike the former case, **TS2-A2** leading to the *syn ent*-**8a** stereoisomer became the most stable, while **TS1-A1** to the *anti ent*-**6a** counterpart turned out to be higher in energy by 1.1 kcal·mol^–1^ (Figure 6), which results in a dr of 10:90. Although the calculated energy is slightly overestimated in comparison to the experimental data (dr 23:77, *ee* 99%), the agreement is satisfactory, and the switch of the relative configuration by changing the phosphine substituent is totally reproduced. The interactions governing such a stereochemical change are subtle and not immediately apparent. The key lies in the *tert*-butyl groups of one of the aryl groups of the phosphine, as in **TS1-A1** (Figure 6), where one of these groups is positioned near the aryl moiety of the oxocarbenium cation. This proximity destabilizes the antiperiplanar approximation, as described in the previous case, thereby favoring a synclinal-type approach, as observed in **TS1-A2** (Figure 6).

**Figure 6:**
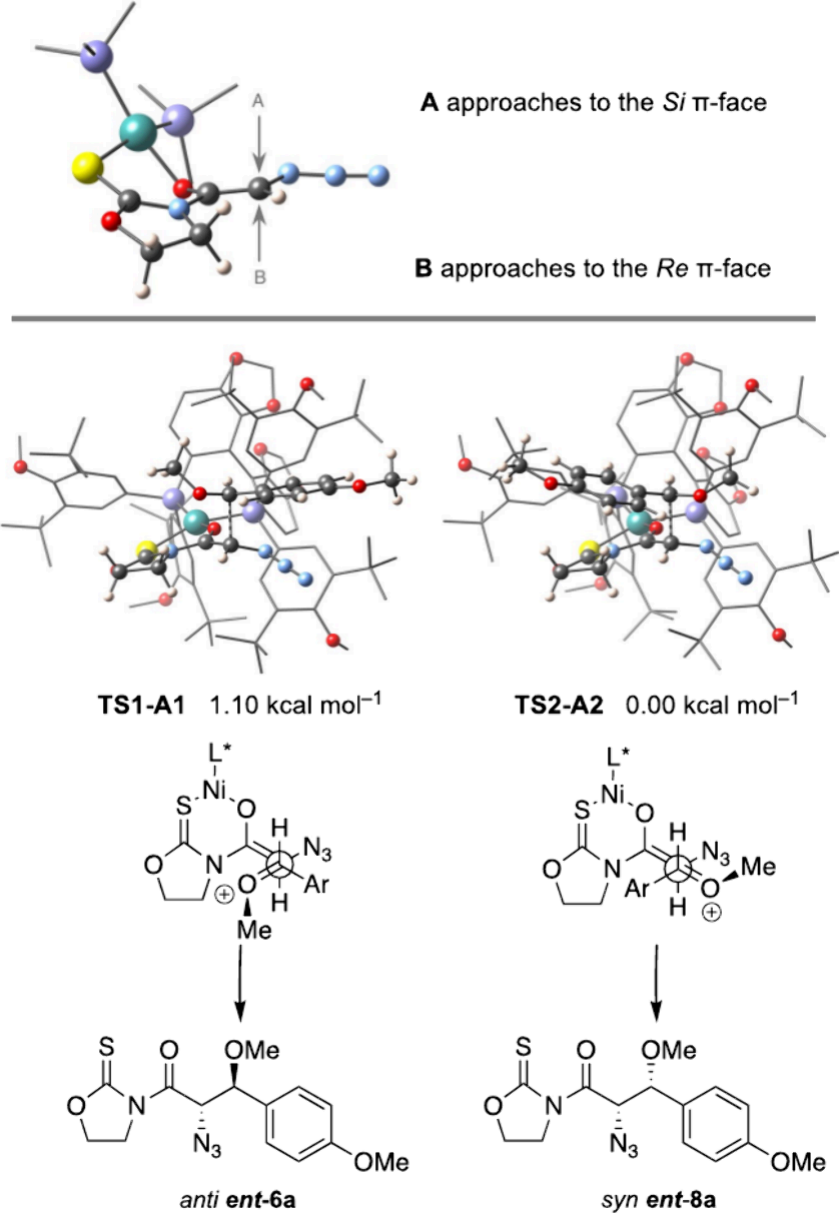
Approaches of the oxocarbenium intermediate from acetal **a** to the [(*R*)-DTBM-SEGPHOS]­Ni­(II) enolate from thioimide **4**.

Taking advantage of these results, we assessed the stereochemical outcome of the reaction of the [(*R*)-DTBM-SEGPHOS] nickel enolate with different acetals described in Table 5 and Figure 2. We found that increasing the steric bulk of the protecting group on the aromatic moiety (PG: Bn) in acetal **d** led to *syn ent*-**8d** as a single stereoisomer, with a much better diastereoselectivity than the experimental dr (*anti*/*syn)* 21:79 (entry 2 of Table 5), while the use of a bulky alkyl group (R: Bn) in acetal **c** made the transition state toward the *anti* stereoisomer the most stable and produced a dr (*anti*/*syn*) 59:41, in close agreement to the experimentally observed dr 65:35 (entry 7 in Table 5).

In summary, the computational study accounts for the experimental results. It suggests that the reaction of nickel enolates containing the Tol-BINAP ligand is mainly dominated by electronic factors, while that of enolates with the DTBM-SEGPHOS ligand entails serious steric interactions. The bulkiness of the phosphine ligands becomes essential to the stereochemical outcome of the reaction and is thus crucial for observed stereodivergence. In a similar manner, the bulk of the alkyl group of the acetal also affects the reaction with the DTBM-SEGPHOS catalyst and can even switch the relative configuration of the major diastereomer.

All of these results agree with the mechanism represented below. Indeed, the overall process begins when the silyl triflate converts nickel­(II) chloride into the triflate counterpart, **I** in Scheme 6, the true catalytic species.^28^ This interacts with the thioimide to give chelated complex **II** with enhanced acid character, which allows a weak base such as lutidine to transform it into enolate **III**, the nucleophile. In parallel, the required electrophile, oxocarbenium intermediate **IV**, stems from the reaction of the dialkyl acetal with the silyl triflate. Thus, the stage is set for the carbon–carbon bond forming step through an open transition state in which the ligand L* of the enolate directs the approach to oxocarbenium intermediate **IV** to deliver **V**. Eventually, the release of the desired β-alkoxy-α-azido adduct permits the nickel triflate to start a new catalytic cycle (Scheme 6).

**Scheme 6:**
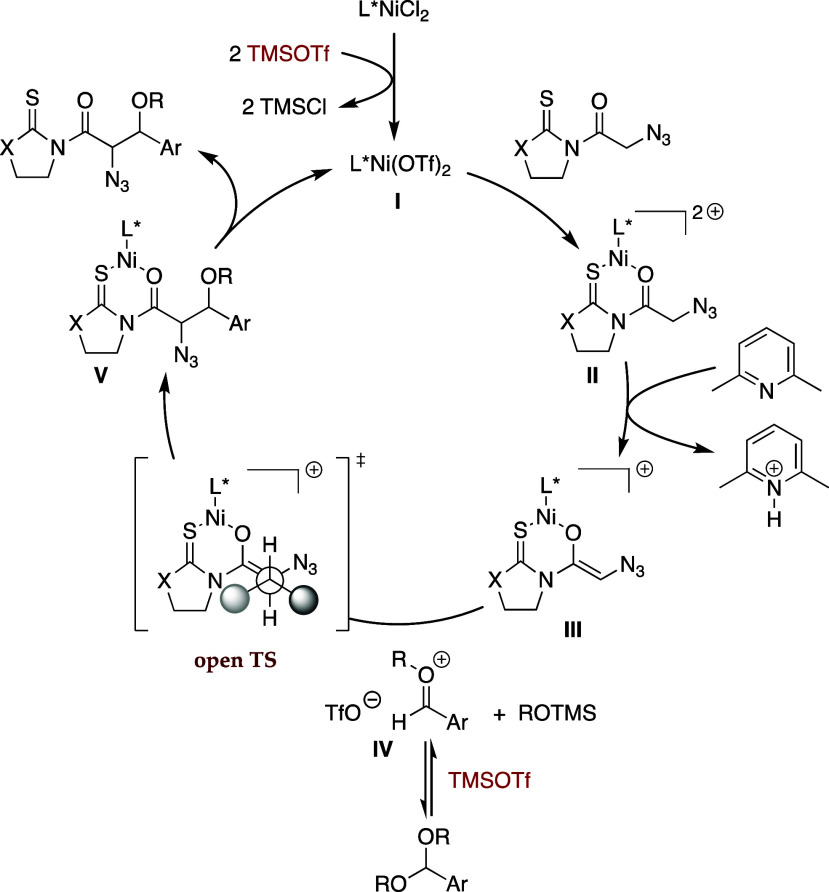
Proposed Mechanism for the Reaction of Thioimides **3** (X: S) or **4** (X: O) with Dialkyl Acetals Catalyzed by Chiral Nickel­(II) Complexes^a^

a L*: Tol-BINAP or DTBM-SEGPHOS.

## Conclusions

This study describes a direct, asymmetric, and stereodivergent method to get access at will to any of the potential stereoisomers of β-alkoxy-β-aryl-α-amino acids. This hinges on a stereocontrolled TMSOTf-mediated aldol-like addition of *N*-azidoacetyl thioimides to dialkyl acetals from aromatic aldehydes catalyzed by [Tol-BINAP]­NiCl_2_ and [DTBM-SEGPHOS]­NiCl_2_. The resultant *anti* and *syn* stereoisomers are isolated in good yields with outstanding enantioselectivity and can be easily converted into enantiomerically pure intermediates such as dipeptide fragments, which can be used for the synthesis of structurally complex cyclopeptides and cyclodepsipeptides. Furthermore, theoretical studies have unveiled some clues for a better understanding of the stereochemical outcome of such transformations and support a mechanism based on the addition of chiral nickel­(II) enolates to oxocarbenium intermediates through open-transition states.

## Supplementary Material



## Data Availability

The data underlying this study are available in the published article and its Supporting Information.

## Notes

Experimental procedures, computational calculations, characterization data of all new compounds, NMR spectra, and chromatograms (PDF)

## #

Sintenovo (Esteve Mexico), Calle 13 Este 3, Colonia Civac, Jiutepec, 62578 Morelos, México

## $

Institut de Química Avançada–CSIC, Barcelona 08028, Spain

## &

University of Cambridge, Cambridge CB2 1TN, United Kingdom
