# Supplementary material for: Direct, Asymmetric, and Stereodivergent Reactions of N‑Azidoacetyl Thioimides with Aromatic Acetals Catalyzed by Chiral Nickel(II) Complexes. An Approach to the Synthesis of syn- and anti-β‑Alkoxy-α-amino Acids
Source: J Org Chem. 2025 Jun 17;90(25):8519–30. doi: 10.1021/acs.joc.5c00390 (PMC12210260; doi:10.1021/acs.joc.5c00390)

Supporting Information

Experimental Procedures. Computational Calculations.

NMR Spectra and HPLC Chromatograms

Direct, Asymmetric, and Stereodivergent Reactions of *N*-Azidoacetyl Thioimides with Aromatic Acetals Catalyzed by Chiral Nickel(II) Complexes. An Approach to the Synthesis of *syn*- and *anti*-β-Alkoxy-α-Amino Acids

Miguel Mellado-Hidalgo,[†] Joan Conejos-Jalencas,[†] Saúl F. Teloxa,[†] Andrea Suárez-Herrera,[†] Luke McCall,[†] Anna M. Costa,[*,†] Pedro Romea,[*,†] Fèlix Urpí,[*,†] Gabriel Aullón,[◊] and Cristina Puigjaner[#]

† *Department of Inorganic and Organic Chemistry, Section of Organic Chemistry, and Institut de Biomedicina de la Universitat de Barcelona, Universitat de Barcelona, Carrer Martí i Franqués 1–11, 08028 Barcelona, Catalonia, Spain.*

◊ *Department of Inorganic and Organic Chemistry, Section of Inorganic Chemistry, and Institut de Química Teòrica i Computacional de la Universitat de Barcelona, Universitat de Barcelona, Carrer Martí i Franqués 1–11, 08028 Barcelona, Catalonia, Spain.*

X-Ray Diffraction Unity, CCTiUB, Universitat de Barcelona, Carrer Solé i Sabarís 1–3, 08028, Barcelona, Catalonia, Spain.

Contents

1. General Experimental Methods S3

2. Synthesis of Starting Materials S4

    2.1. Synthesis of 1,3-Oxazolidine-2-thione S4

    2.2. Synthesis of *N*-Azidoacetyl Thioimides S5

3. Temperature Assessment S7

4. TMSOTf–Mediated Reactions Catalyzed by [(*S*)–Tol-BINAP]$NiCl_2$ S8

    4.1. General Procedure S8

    4.2. Physical and Spectroscopic Data of the Resultant Products S9

5. TMSOTf–Mediated Reactions Catalyzed by [(*R*)–DTBM-SEGPHOS]$NiCl_2$ S26

    5.1. General Procedure S26

    5.2. Physical and Spectroscopic Data of the Resultant Products S27

6. Structural Studies S49

7. Transformations S51

8. X-Ray Analyses S53

    8.1. X-Ray Analysis of Adduct **9** S53

    8.2. X-Ray Analysis of Adduct **10** S55

9. Computational Calculations S57

    9.1. Computational Details S57

    9.2. Structural Analysis S57

    9.3. Calculations S57

    9.4. Computational Data S66

10. References S120

11. Copies of NMR Spectra and HPLC Chromatograms S121

1. General Experimental Methods

Unless otherwise noted, reactions were conducted in oven-dried glassware under inert atmosphere of N_2 with anhydrous solvents. The solvents and reagents were dried and purified when necessary according to standard procedures. Commercially available reagents were used as received.

Analytical thin-layer chromatographies (TLC) were carried out on Merck silica gel 60 F_{254} plates and analysed by UV (254 nm) and stained with *p*-anisaldehyde; column chromatographies were carried under low pressure (flash) conditions and performed on SDS silica gel 60 (35–70 μm). Eluents are indicated in brackets in each case. $\mathbf{R_f}$ values are approximate.

Chiral HPLC analyses were conducted on a Shimadzu LC-20 HPLC system, using chiral Phenomenex Lux® columns under isocratic conditions and UV-detected at 254 nm.

Melting points (**Mp**) were determined with a Stuart SMP10 apparatus and are uncorrected.

Specific rotations ($[\alpha]_D$) were determined at 20 °C on a Perkin-Elmer 241 MC polarimeter equipped with a sodium lamp (λ 589 nm, D-line).

IR spectra (Attenuated Total Reflectance, ATR) were recorded on a Nicolet 6700 FT-IR Thermo Scientific spectrometer and only the more representative frequencies (ν) are reported in cm^{-1}.

^{1}H NMR (400 MHz) and **^{13}C{^{1}H} NMR** (100.6 MHz) spectra were recorded at room temperature on a Varian Mercury 400. **^{1}H NMR** (400 MHz) spectra was recorded at room temperature on a Bruker 400. Chemical shifts (δ) are quoted in ppm and referenced to internal TMS (δ 0.00 for ^{1}H NMR) and $CDCl_3$ (δ 77.0 for ^{13}C NMR). Data are reported as follows: chemical shift (number of protons, multiplicity, coupling constants, proton); multiplicity is reported as follows: s, singlet; d, doublet; t, triplet; q, quartet; or m, multiplet (and their corresponding combinations); coupling constants (*J*) are quoted in Hz. With the exception of the starting materials, **2D COSY** and **HSQC** spectra were utilized to aid in the peak assignments of all the products.

High resolution mass spectra (**HRMS**) were obtained with an Agilent 1100 spectrometer by the Unitat d'Espectrometria de Masses, Universitat de Barcelona.

2. Synthesis of Starting Materials

2.1. Synthesis of 1,3-Oxazolidine-2-thione

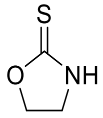

Anhydrous Et_3N (5.6 mL, 40 mmol, 1 equiv) was added dropwise to a solution of 2-aminoethanol (2.4 mL, 40 mmol, 1 equiv) in absolute methanol (40 mL) under a nitrogen atmosphere, and the mixture was then cooled to 0 °C. Carbon disulfide (3.6 mL, 60 mmol, 1.5 equiv) was added dropwise and the resultant mixture was stirred at 0 °C for 30 min, and at room temperature for another 30 min.

The resulting pale-yellow solution was then quenched with 30% (v/v) H_2O_2 (7 mL), until no more white solid is formed while its addition. The yellow suspension was stirred for 30 min, filtered, and concentrated under reduced pressure using a peroxide tramp, followed by the addition of 2 M NaOH (20 mL) and 2 M HCl (ca. 30 mL) to acidify the solution to pH 1.

The resulting bright yellow solution was extracted with CH_2Cl_2 (3 × 25 mL), and the combined organic layers were dried ($MgSO_4$), filtered, and concentrated *in vacuo*. The resulting solid was purified by recrystallization (cyclohexene/CH_2Cl_2) to give 1,3-oxazolidine-2-thione (3.08 g, 30 mmol, 75% yield) as a white crystalline powder.

White solid.
Mp 98–100 °C.
R_f 0.15 (60:40 Hexanes/EtOAc).
IR (ATR) ν 3203, 2923, 1520, 1456, 1398, 1312, 1284, 1232, 1203, 1162, 1033 cm^{-1}.
^{1}H NMR ($CDCl_3$, 400 MHz) δ 8.19 (1H, br s), 4.73 (2H, t, *J* = 8.8 Hz), 3.85 (2H, t, *J* = 8.8 Hz).
$^{13}C\{^1H\}$ NMR ($CDCl_3$, 100.6 MHz) δ 190.1, 70.4, 44.1.
HRMS (+ESI): *m/z* calcd. for $[M + H]^+$ C_3H_6NOS: 104.0165; found 104.0167.

2.2. Synthesis of *N*-Azidoacetyl Thioimides

General Procedure

A 1.3 M aqueous solution of 2-bromoacetic acid (1.0 equiv) was added dropwise to an aqueous solution (3 M) of sodium azide (2 equiv) at 0 °C. After 15 min, the ice bath was removed and the reaction mixture was stirred overnight at room temperature.

The reaction was then slowly acidified with 2 M HCl until pH 1 and the aqueous solution was extracted with Et_2O. The combined organic extracts were dried ($NaSO_4$), filtered, and the solvent was removed under reduced pressure to afford 2-azidoacetic acid (99% yield) as a colorless oil, which was used in the next step without further purification.

The former 2-azidoacetic acid (1.1 equiv) in CH_2Cl_2 (1 M) was added dropwise to a solution of the proper heterocycle (1 equiv), EDC·HCl (1.2 equiv), and DMAP (0.05 equiv) in CH_2Cl_2 (0.5 M) at 0 °C and stirred for 15 min. The solution was then allowed to reach room temperature and was stirred overnight.

The reaction mixture was washed with deionized H_2O and brine. The organic layers were combined, dried ($NaSO_4$), filtered and concentrated *in vacuo*. The resultant crude mixture was purified by flash column chromatography to afford the desired pure product.

N-(2-Azidoacetyl)-1,3-thiazolidine-2-thione (3)

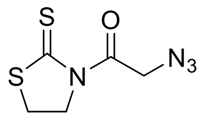

The General Procedure was followed with commercial 1,3-thiazolidine-2-thione (2.38 g, 20.0 mmol) and 2-azidoacetic acid (2.24 g, 22.2 mmol). The resulting crude mixture was purified by flash column chromatography (70:30 Hexanes/EtOAc) to afford **3** (2.95 g, 14.6 mmol, 73% yield) as a yellow solid.

Yellow solid.
Mp 57–59 °C.
R_f 0.40 (70:30 Hexanes/EtOAc).
IR (ATR) ν 2904, 2097, 1698, 1395, 1349, 1264, 1220, 1157, 1043 cm^{-1}.
^{1}H NMR ($CDCl_3$, 400 MHz) δ 4.84 (2H, s), 4.64 (2H, t, *J* = 7.6 Hz), 3.41 (2H, t, *J* = 7.6 Hz).
^{13}C{^{1}H} NMR ($CDCl_3$, 100.6 MHz) δ 201.5, 169.4, 55.6, 55.3, 29.2.
HRMS (+ESI): *m/z* calcd. for $[M + H]^+$ $C_5H_7N_4OS_2$: 203.0055; found: 203.0061.

N-(2-Azidoacetyl)-1,3-oxazolidine-2-thione (4)

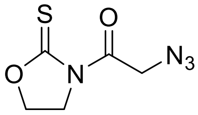

The General Procedure was followed with 1,3-oxazolidine-2-thione (3.10 g, 30.0 mmol) and 2-azidoacetic acid (3.34 g, 33.0 mmol). The resulting crude mixture was purified by flash column chromatography (85:15 Hexanes/EtOAc) to afford **4** (3.75 g, 20.1 mmol, 67% yield) as a white solid.

White solid.

Mp 32–34 °C.

R_f 0.25 (85:15 Hexanes/EtOAc).

IR (ATR) ν 2917, 2100, 1697, 1364, 1320, 1212, 1168, 1017 cm^{-1}.

^{1}H NMR ($CDCl_3$, 400 MHz) δ 4.91 (2H, s), 4.65 (2H, t, *J* = 8.5 Hz), 4.29 (2H, t, *J* = 8.5 Hz).

^{13}C{^{1}H} NMR ($CDCl_3$, 100.6 MHz) δ 184.9, 169.0, 67.5, 54.5, 46.9.

HRMS (+ESI): *m/z* calcd. for $[M + H]^+$ $C_5H_7N_4O_2S$: 187.0284; found: 187.0286. *m/z* calcd. for $[M + Na]^+$ $C_5H_6N_4NaO_2S$: 209.0104; found: 209.0105.

Both compounds (**3-4**) have also been reported elsewhere.[1,2]

3. Temperature Assessment

3.1. General Procedure

A solution of *N*-azidoacetyl thioimide (**3–4**, 0.50 mmol, 1.0 equiv), 4-methoxybenzaldehyde dimethyl acetal (**a**, 95 µL, 0.55 mmol, 1.1 equiv), a chiral catalyst (10 µmol, 2 mol%) in CH_2Cl_2 (2 mL) was cooled at 0 or –20 °C under N_2. Then, neat TMSOTf (120 µL, 0.65 mmol, 1.3 equiv) was added followed by 2,6-lutidine (90 µL, 0.75 mmol, 1.5 equiv) and the mixture was stirred at that same temperature.

The reaction mixture was quenched with sat NH_4Cl (2 mL) and partitioned in CH_2Cl_2 (15 mL) and water (15 mL). The aqueous layer was extracted with CH_2Cl_2 (2 × 15 mL), and the combined organic extracts were dried (Na_2SO_4), and concentrated. The resultant residue was analyzed by ^{1}H NMR (400 MHz). Both the diastereoselectivity (dr) and the conversion are summarized in Table SI-1.

Eventually, the crude mixture was purified by flash column chromatography on silica gel to afford the desired products and the enantioselectivity (ee) of the reaction was evaluated with chiral HPLC.

Table SI-1. Influence of the temperature on the reaction

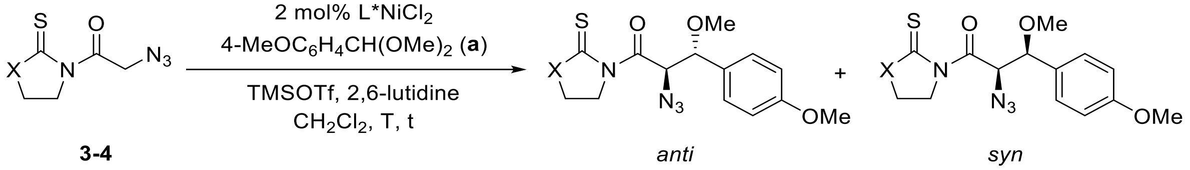

Entry	Thioimide	L*	T (°C)	t (h)	Conv. (%)	dr (*anti*/syn)	Yield (%) [a]	*ee* (%)
1	**3**	[(*S*)-Tol-BINAP]	– 20	2.5	100	82:18	64	99
2	**3**	[(*S*)-Tol-BINAP]	0	2.5	100	81:19	64	99
3	**4**	[(*S*)-DTBM-SEGPHOS]	– 20	5	100	24:76	64	97
4	**4**	[(*S*)-DTBM-SEGPHOS]	0	5	100	24:76	63	96

[a] Isolated yield of the major diastereomer

4. TMSOTf–Mediated Reactions Catalyzed by [(*S*)-Tol-BINAP]$NiCl_2$

4.1. General Procedure

A solution of *N*-(2-azidoacetyl)-1,3-thiazolidine-2-thione (3, 1.0 equiv), an aromatic dialkyl acetal (1.1 equiv) and [(*S*)-Tol-BINAP]$NiCl_2$ (2–5 mol%) in CH_2Cl_2 (0.25 M) was cooled at 0 °C under a N_2 athmosphere. Then, neat TMSOTf (1.3 equiv) was added dropwise to the stirring green-brown solution followed by the addition of 2,6-lutidine (1.5 equiv), and the resultant dark red mixture was stirred at 0 °C until completion.

For a 0.5 mmol scale reaction, the mixture was quenched with sat NH_4Cl (2 mL) and partitioned in CH_2Cl_2 (15 mL) and water (15 mL). The aqueous layer was then extracted with neat CH_2Cl_2 (2 × 15 mL), and the combined organic extracts were dried (Na_2SO_4) and concentrated *in vacuo*. Finally, the crude residue was purified by flash column chromatography to yield the named compound as a single enantiomer.

The *anti/syn* diasteromeric ratio (dr) was established by ^1H NMR analysis of the crude mixture. Similarly, the enantiomeric purity (*ee*) of the *anti* compound isolated was established by chiral HPLC analysis of the purified products of both the racemic and the enantioselective reactions.

4.2. Physical and Spectroscopic Data of the Resultant Products

_N_-[(2_R_,3_R_)-2-Azido-3-methoxy-3-(4-methoxyphenyl)propanoyl]-1,3-thiazolidine-2-thione (5a)

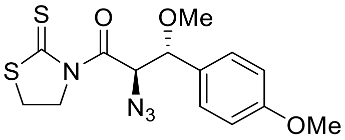

The General Procedure was followed with *N*-(2-azidoacetyl)-1,3-thiazolidine-2-thione (**3**, 101 mg, 0.50 mmol), 4-methoxybenzaldehyde dimethyl acetal (**a**, 95 µL, 0.55 mmol), [(*S*)-Tol-BINAP]$NiCl_2$ (20.2 mg, 25 µmol, 5 mol%), TMSOTf (120 µL, 0.65 mmol), and 2,6-lutidine (90 µL, 0.75 mmol) at 0 °C for 1 h.

The residue (dr 80:20) was purified by column chromatography (70:30 Hexanes/Et_2O) to afford 112 mg of adduct **5a** (0.32 mmol, 63% yield) and 18 mg (51 µmol, 24% yield) of the *syn* diastereomer.

Yellow solid.
Mp 116–118 °C.
R_f 0.45 (70:30 Hexanes/Et_2O).
Chiral HPLC (Phenomenex Lux® Cellulose-1 column, 5% *i*-PrOH in hexane, flow rate 1.0 mL·min^{-1}): Rt 32.5 min (Major 2*R*,3*R*-isomer) [Rt 30.1 min (minor 2*S*,3*S*-isomer)], 99% *ee*.
$[\alpha]_D^{20}$ –135.3 (*c* 1.00, $CHCl_3$).
IR (ATR) ν 2929, 2091, 1704, 1608, 1507, 1358, 1141, 1024 cm^{-1}.
^{1}H NMR ($CDCl_3$, 400 MHz) δ 7.34–7.30 (2H, m, Ar$\underline{H}$), 6.96–6.92 (2H, m, Ar$\underline{H}$), 6.12 (1H, d, *J* = 8.7 Hz, C$\underline{H}N_3$), 4.60 (1H, ddd, *J* = 11.9, 7.7, 3.9 Hz, NC$\underline{H}_aH_b$), 4.53 (1H, d, *J* = 8.7 Hz, C$\underline{H}OCH_3$), 4.50 (1H, ddd, *J* = 11.9, 10.5, 7.6 Hz, NC$H_a\underline{H}_b$), 3.82 (3H, s, ArOC$\underline{H}_3$), 3.43–3.36 (1H, m, SC$\underline{H}_aH_b$), 3.26 (1H, ddd, *J* = 11.2, 7.6, 3.9 Hz, SC$H_a\underline{H}_b$), 3.18 (3H, s, CHOC$\underline{H}_3$).
^{13}C{^{1}H} NMR ($CDCl_3$, 100.6 MHz) δ 202.1 (C), 171.0 (C), 160.1 (C), 128.9 (CH), 128.6 (C), 114.2 (CH), 83.7 (CH), 63.9 (CH), 56.8 (CH_3), 56.2 (CH_2), 55.3 (CH_3), 28.9 (CH_2).
HRMS (+ESI): *m/z* calcd. for $[M – OMe]^+$ $C_{13}H_{13}N_4O_2S_2$: 321.0474; found: 321.0471. *m/z* calcd. for $[M + H]^+$ $C_{14}H_{17}N_4O_3S_2$: 352.0664; found: 352.0666.

***N*-[(2*R*,3*R*)-3-Allyloxy-2-azido-3-(4-methoxyphenyl)propanoyl]-1,3-thiazolidine-2-thione (5b)**

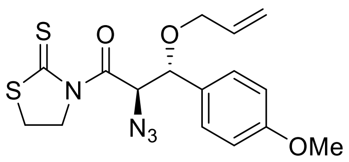

The General Procedure was followed with *N*-(2-azidoacetyl)-1,3-thiazolidine-2-thione (**3**, 101 mg, 0.50 mmol), 4-methoxybenzaldehyde diallyl acetal (**b**, 130 mg, 0.55 mmol), [(*S*)-Tol-BINAP]$NiCl_2$ (20.3 mg, 25 μmol, 5 mol%), TMSOTf (120 μL, 0.65 mmol) and 2,6-lutidine (90 μL, 0.75 mmol), and the reaction mixture was stirred at 0 °C for 1 h.

The residue (dr 79:21) was purified by column chromatography (60:40 Hexanes/Et_2O) to give 101 mg (0.27 mmol, 53% yield) of *anti* adduct **5b** and 37 mg (0.10 mmol, 20% yield) of the *syn* diastereomer.

Yellow oil.
R_f 0.35 (60:40 Hexanes/Et_2O).
Chiral HPLC (Phenomenex Lux® Cellulose-5 column, 5% *i*-PrOH in hexane, flow rate 1.0 mL·min^{-1}): Rt 18.1 min (Major 2*R*,3*R*-isomer) [Rt 20.1 min (minor 2*S*,3*S*-isomer)], 98% *ee*.
$[\alpha]_D^{20}$ –136.7 (*c* 1.00, $CHCl_3$).
IR (ATR) ν 2931, 2837, 2206, 2156, 1701, 1609, 1585, 1461, 1440, 1303, 1024 cm^{-1}.
^{1}H NMR ($CDCl_3$, 400 MHz) δ 7.36–7.30 (2H, m, Ar$\underline{H}$), 6.97–6.90 (2H, m, Ar$\underline{H}$), 6.13 (1H, d, *J* = 8.8 Hz, C$\underline{H}N_3$), 5.85–5.74 (1H, m, $OCH_2C\underline{H}=CH_2$), 5.20 (1H, dq, *J* = 17.2, 1.7 Hz, $OCH_2CH=C\underline{H}_aH_b$), 5.14 (1H, dq, *J* = 10.4, 1.4 Hz, $OCH_2CH=CH_a\underline{H}_b$), 4.66 (1H, d, *J* = 8.8 Hz, $C\underline{H}OCH_2CH=CH_2$), 4.58 (1H, ddd, *J* = 11.9, 7.7, 3.5 Hz, $NC\underline{H}_aH_b$), 4.48 (1H, ddd, *J* = 11.9, 10.9, 7.5 Hz, $NCH_a\underline{H}_b$), 3.91 (1H, ddt, *J* = 12.8, 4.9, 1.6 Hz, $OC\underline{H}_aH_bCH=CH_2$), 3.83 (3H, s, $ArOC\underline{H}_3$), 3.73 (1H, ddt, *J* = 12.8, 6.0, 1.4 Hz, $OCH_a\underline{H}_bCH=CH_2$), 3.39 (1H, td, *J* = 10.9, 7.7 Hz, $SC\underline{H}_aH_b$), 3.25 (1H, ddd, *J* = 10.9, 7.5, 3.5 Hz, $SCH_a\underline{H}_b$).
^{13}C{^{1}H} NMR ($CDCl_3$, 100.6 MHz) δ 202.1 (C), 171.5 (C), 160.2 (C), 134.1 (CH), 129.0 (CH), 128.9 (C), 116.9 (CH_2), 114.2 (CH), 81.7 (CH), 69.6 (CH_2), 64.2 (CH), 56.3 (CH_2), 55.3 (CH_3), 29.1 (CH_2).
HRMS (+ESI): *m/z* calcd. for $[M - OCH_2CH=CH_2]^+$ $C_{13}H_{13}N_4O_2S_2$: 321.0474; found: 321.0472. *m/z* calcd. for $[M + Na]^+$ $C_{16}H_{18}N_4NaO_3S_2$: 401.0713; found: 401.0713.

***N*-[(2*R*,3*R*)-2-Azido-3-benzyloxy-3-(4-methoxyphenyl)propanoyl]-1,3-thiazolidine-2-thione (5c)**

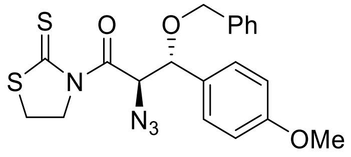

The General Procedure was followed with *N*-(2-azidoacetyl)-1,3-thiazolidine-2-thione (**3**, 101 mg, 0.50 mmol), 4-methoxybenzaldehyde dibenzyl acetal (**c**, 183 mg, 0.55 mmol), [(*S*)-Tol-BINAP]NiCl$_2$ (20.3 mg, 25 μmol, 5 mol%), TMSOTf (120 μL, 0.65 mmol) and 2,6-lutidine (90 μL, 0.75 mmol), and the reaction mixture was stirred at –20 °C for 1 h.

The residue (dr 80:20) was purified by column chromatography (80:20 Hexanes/EtOAc) to give 124 mg (0.29 mmol, 58% yield) of *anti* adduct **5c** and 37 mg (0.10 mmol, 20% yield) of the *syn* diastereomer.

Yellow oil.
R$_f$ 0.30 (80:20 Hexanes/EtOAc).
Chiral HPLC (Phenomenex Lux® Cellulose-1 column, 20% *i*-PrOH in hexane, flow rate 1.0 mL·min^{-1}): Rt 12.5 min (Major 2*R*,3*R*-isomer) [Rt 22.3 min (minor 2*S*,3*S*-isomer)], 97% *ee*.
$[\alpha]_D^{20}$ –101.8 (*c* 1.00, CHCl$_3$).
IR (ATR) ν 2925, 2105, 1698, 1609, 1585, 1511, 1454, 1303, 1141, 1022 cm^{-1}.
^{1}H NMR (CDCl$_3$, 400 MHz) δ 7.40–7.36 (2H, m, ArH), 7.34–7.30 (2H, m, ArH), 7.30–7.26 (1H, m, ArH), 7.25–7.20 (2H, m, ArH), 6.99–6.93 (2H, m, ArH), 6.17 (1H, d, *J* = 9.0 Hz, CHN$_3$), 4.70 (1H, d, *J* = 9.0 Hz, CHOBn), 4.46–4.35 (2H, m, NCH$_2$), 4.42 (1H, d, *J* = 11.3 Hz, OCH$_a$H$_b$Ar), 4.19 (1H, d, *J* = 11.3 Hz, OCH$_a$H$_b$Ar), 3.84 (3H, s, ArOCH$_3$), 3.12–3.01 (2H, m, SCH$_2$).
^{13}C{^{1}H} NMR (CDCl$_3$, 100.6 MHz) δ 202.2 (C), 171.8 (C), 160.3 (C), 137.6 (C), 129.1 (CH), 128.8 (C), 128.3 (CH), 127.8 (CH), 127.7 (CH), 114.3 (CH), 82.5 (CH), 70.9 (CH$_2$), 64.3 (CH), 56.3 (CH$_2$), 55.3 (CH$_3$), 29.0 (CH$_2$).
HRMS (+ESI): *m/z* calcd. for [M – OBn]$^+$ $C_{13}H_{13}N_4O_2S_2$: 321.0474; found: 321.0469. *m/z* calcd. for [M + Na]$^+$ $C_{20}H_{20}N_4NaO_3S_2$: 451.0869; found: 451.0873.

***N*-[(2*R*,3*R*)-2-Azido-3-(4-benzyloxyphenyl)-3-methoxypropanoyl]-1,3-thiazolidine-2-thione (5d)**

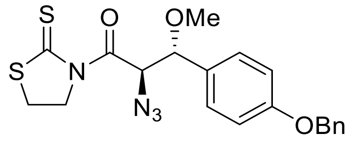

The General Procedure was followed with *N*-(2-azidoacetyl)-1,3-thiazolidine-2-thione (**3**, 51 mg, 0.25 mmol), 4-benzylozybenzaldehyde dimethyl acetal (**d**, 71 mg, 0.275 mmol), [(*S*)-Tol-BINAP]$NiCl_2$ (10.1 mg, 12.5 μmol, 5 mol%), TMSOTf (60 μL, 0.325 mmol) and 2,6-lutidine (45 μL, 0.375 mmol), and the reaction mixture was stirred at 0 °C for 1 h.

The residue (dr 74:26) was purified by column chromatography (70:30 Hexanes/EtOAc) to give 69 mg (0.16 mmol, 64% yield) of *anti* adduct **5d** and 21 mg (50 μmol, 20% yield) of the *syn* diastereomer.

Thick yellow oil.
R_f 0.30 (70:30 Hexanes/EtOAc).
Chiral HPLC (Phenomenex Lux® Cellulose-1 column, 20% *i*-PrOH in hexane, flow rate 1.0 mL·min^{-1}): Rt 23.6 min (Major 2*R*,3*R*-isomer) [Rt 21.7 min (minor 2*S*,3*S*-isomer)], 99% *ee*.
$[\alpha]_D^{20}$ –95.0 (*c* 1.00, $CHCl_3$).
IR (ATR) ν 2924, 2108, 1702, 1608, 1508, 1359, 1157, 1055 cm^{-1}.
^{1}H NMR ($CDCl_3$, 400 MHz) δ 7.45–7.30 (10H, m, Ar$\underline{H}$), 7.03–7.00 (2H, m, Ar$\underline{H}$), 6.12 (1H, d, *J* = 8.6 Hz, C$\underline{H}N_3$), 5.08 (2H, s, OC$\underline{H}_2$Ph), 4.59 (1H, ddd, *J* = 11.9, 7.7, 3.9 Hz, NC$\underline{H}_aH_b$), 4.53 (1H, d, *J* = 8.7 Hz, C$\underline{H}OCH_3$), 4.48 (1H, ddd, *J* = 11.9, 10.5, 7.6 Hz, NC$H_a\underline{H}_b$), 3.39–3.32 (1H, m, SC$\underline{H}_aH_b$), 3.23 (1H, ddd, *J* = 11.6, 7.6, 4.1 Hz, SC$H_a\underline{H}_b$), 3.19 (3H, s, CHOC$\underline{H}_3$).
^{13}C{^{1}H} NMR ($CDCl_3$, 100.6 MHz) δ 202.1 (C), 171.0 (C), 159.4 (C), 136.7 (C), 128.9 (CH), 128.6 (CH), 128.0 (CH), 127.5 (CH), 115.1 (CH), 83.7 (CH), 70.0 (CH_2), 63.9 (CH), 56.9 (CH_3), 56.2 (CH_2), 28.9 (CH_2).
HRMS (+ESI): *m/z* calcd. for [M – OMe]$^+$ $C_{19}H_{17}N_4O_2S_2$: 397.0787; found: 397.0782. *m/z* calcd. for [M + Na]$^+$ $C_{20}H_{20}N_4NaO_3S_2$: 451.0869; found: 451.0859.

***N*-[(2*R*,3*R*)-2-Azido-3-(4-(*tert*-butyldimethylsilyl)phenyl)-3-methoxypropanoyl]-1,3-thiazolidine-2-thione (5e)**

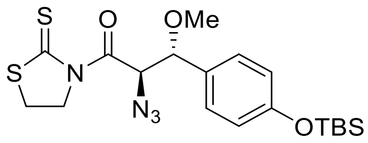

The General Procedure was followed with *N*-(2-azidoacetyl)-1,3-thiazolidine-2-thione (**3**, 51 mg, 0.25 mmol), 4-(*tert*-butyldimethylsilyloxy)benzaldehyde dimethyl acetal (**e**, 78 mg, 0.275 mmol), [(*S*)-Tol-BINAP]$NiCl_2$ (10.1 mg, 12.5 μmol, 5 mol%), TMSOTf (60 μL, 0.325 mmol) and 2,6-lutidine (45 μL, 0.375 mmol), and the reaction mixture was stirred at 0 °C for 1 h.

The residue (dr 72:28) was purified by column chromatography (80:20 Hexanes/EtOAc) to give 71 mg (0.16 mmol, 63% yield) of *anti* adduct **5e** and 27 mg (60 μmol, 24% yield) of the *syn* diastereomer.

Thick yellow oil.
R_f 0.25 (80:20 Hexanes/EtOAc).
Chiral HPLC (Phenomenex Lux® Cellulose-1 column, 10% *i*-PrOH in hexane, flow rate 1.0 mL·min^{-1}): Rt 9.5 min (Major 2*R*,3*R*-isomer) [Rt 8.8 min (minor 2*S*,3*S*-isomer)], 99% *ee*.
$[\alpha]_D^{20}$ –107.5 (*c* 1.00, $CHCl_3$).
IR (ATR) ν 2928, 2106, 1705, 1605, 1506, 1362, 1163, 1049 cm^{-1}.
^{1}H NMR ($CDCl_3$, 400 MHz) δ 7.27–7.25 (2H, m, Ar<u>H</u>), 6.89–6.85 (2H, m, Ar<u>H</u>), 6.09 (1H, d, *J* = 8.7 Hz, C<u>H</u> N_3), 4.61 (1H, ddd, *J* = 11.8, 7.8, 3.9 Hz, NC<u>H</u>$_a$H$_b$), 4.53–4.46 (1H, m, NCH$_a$<u>H</u>$_b$), 4.52 (1H, d, *J* = 8.7 Hz, C<u>H</u> OCH_3), 3.40 (1H, ddd, *J* = 11.1, 10.5, 7.8 Hz, SC<u>H</u>$_a$H$_b$), 3.26 (1H, ddd, *J* = 11.1, 7.6, 3.9 Hz, SCH$_a$<u>H</u>$_b$), 3.19 (3H, s, CHOC<u>H</u>$_3$), 0.98 (9H, s, SiC(C<u>H</u>$_3$)$_3$), 0.20 (6H, s, Si(C<u>H</u>$_3$)$_2$).
^{13}C{^{1}H} NMR ($CDCl_3$, 100.6 MHz) δ 202.0 (C), 171.0 (C), 156.4 (C), 129.3 (C), 128.9 (CH), 120.4 (CH), 83.8 (CH), 64.0 (CH), 56.8 (CH_3), 56.2 (CH_2), 28.9 (CH_2), 25.6 (CH_3), 18.2 (C), –4.4 (CH_3).
HRMS (+ESI): *m/z* calcd. for [M – OMe]$^+$ $C_{18}H_{25}N_4O_2S_2Si$: 421.1183; found: 421.1178. *m/z* calcd. for [M + Na]$^+$ $C_{19}H_{28}N_4NaO_3S_2Si$: 475.1264; found: 475.1260.

N-[(2R,3R)-2-Azido-3-methoxy-3-(3-methoxyphenyl)propanoyl]-1,3-thiazolidine-2-thione (5f)

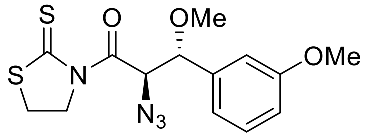

The General Procedure was followed with *N*-(2-azidoacetyl)-1,3-thiazolidine-2-thione (**3**, 101 mg, 0.50 mmol), 3-methoxybenzaldehyde dimethyl acetal (**f**, 100 mg, 0.55 mmol), [(*S*)-Tol-BINAP]$NiCl_2$ (20.4 mg, 25 μmol, 5 mol%), TMSOTf (120 μL, 0.65 mmol) and 2,6-lutidine (90 μL, 0.75 mmol), and the reaction mixture was stirred at 0 °C for 2 h.

The residue (dr 62:38) was purified by column chromatography (90:10 Hexanes/THF) to afford 86 mg of adduct **5f** (0.24 mmol, 49% yield) and 59 mg (0.17 mmol, 34% yield) of the *syn* diastereomer.

Yellow oil.

R_f 0.10 (90:10 Hexanes/THF).

Chiral HPLC (Phenomenex Lux® Cellulose-5 column, 3% *i*-PrOH in hexane, flow rate 1.0 mL·min^{-1}): Rt 28.2 min (Major 2*R*,3*R*-isomer) [Rt 32.2 min (minor 2*S*,3*S*-isomer)], 98% *ee*.

$[\alpha]_D^{20}$ –57.9 (*c* 1.00, $CHCl_3$).

IR (ATR) ν 2935, 2831, 2106, 1701, 1598, 1358, 1101 cm^{-1}.

^{1}H NMR ($CDCl_3$, 400 MHz) δ 7.33 (1H, t, *J* = 7.9 Hz, Ar<u>H</u>), 6.98 (1H, dd, *J* = 7.5, 1.3 Hz, Ar<u>H</u>), 6.95 (1H, t, *J* = 2.1 Hz, Ar<u>H</u>), 6.94–6.89 (1H, m, Ar<u>H</u>), 6.15 (1H, d, *J* = 8.5 Hz, C<u>H</u>N_3), 4.56 (1H, d, *J* = 8.5 Hz, C<u>H</u>OCH_3), 4.62–4.58 (1H, m, NC$\underline{H_a}H_b$), 4.52–4.45 (1H, m, NC$H_a\underline{H_b}$), 3.83 (3H, s, ArOC$\underline{H_3}$), 3.41–3.35 (1H, m, SC$\underline{H_a}H_b$), 3.27–3.23 (1H, m, SC$H_a\underline{H_b}$), 3.23 (3H, s, CHOC$\underline{H_3}$).

^{13}C{^{1}H} NMR ($CDCl_3$, 100.6 MHz) δ 202.1 (C), 170.8 (C), 159.9 (C), 138.4 (C), 129.8 (CH), 120.0 (CH), 114.6 (CH), 112.8 (CH), 84.1 (CH), 63.7 (CH), 57.2 (CH_3), 56.2 (CH_2), 55.3 (CH_3), 28.9 (CH_2).

HRMS (+ESI): *m/z* calcd. for $[M - N_2 + H]^+$ $C_{14}H_{17}N_2O_3S_2$: 325.0675; found: 325.0675. *m/z* calcd. for $[M + Na]^+$ $C_{14}H_{16}N_4NaO_3S_2$: 375.0556; found: 375.0560.

***N*-[(2*R*,3*R*)-2-Azido-3-methoxy-3-(2-methoxyphenyl)propanoyl]-1,3-thiazolidine-2-thione (5g)**

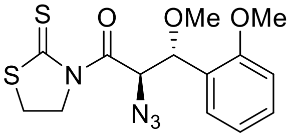

The General Procedure was followed with *N*-(2-azidoacetyl)-1,3-thiazolidine-2-thione (**3**, 101 mg, 0.50 mmol), 2-methoxybenzaldehyde dimethyl acetal (**g**, 100 mg, 0.55 mmol), [(*S*)-Tol-BINAP]$NiCl_2$ (40.6 mg, 50 µmol, 10 mol%), TMSOTf (120 µL, 0.65 mmol) and 2,6-lutidine (90 µL, 0.75 mmol), and the reaction mixture was stirred at 0 °C for 16 h.

The residue (dr 67:33) was purified by column chromatography (90:10 Hexanes/EtOAc) to afford 91 mg of adduct **5g** (0.25 mmol, 50% yield) and 22 mg (62 µmol, 13% yield) of the *syn* diastereomer.

Yellow oil.
R_f 0.30 (90:10 Hexanes/EtOAc).
Chiral HPLC (Phenomenex Lux® Cellulose-1 column, 5% *i*-PrOH in hexane, flow rate 1.0 mL·min^{-1}): Rt 49.5 min (Major 2*R*,3*R*-isomer) [Rt 29.8 min (minor 2*S*,3*S*-isomer)], 96% *ee*.
$[\alpha]_D^{20}$ –106.4 (*c* 1.00, $CHCl_3$).
IR (ATR) ν 2927, 2853, 2104, 1702, 1600, 1507, 1360, 1095 cm^{-1}.
^{1}H NMR ($CDCl_3$, 400 MHz) δ 7.41–7.30 (2H, m, Ar$\underline{H}$), 7.04–6.97 (1H, m, Ar$\underline{H}$), 6.95–6.88 (1H, m, Ar$\underline{H}$), 6.28 (1H, d, *J* = 6.3 Hz, C$\underline{H}N_3$), 5.11 (1H, d, *J* = 6.3 Hz, C$\underline{H}OCH_3$), 4.43 (1H, ddd, *J* = 11.8, 7.2, 3.6 Hz, NC$\underline{H_a}H_b$), 4.35 (1H, ddd, *J* = 11.8, 10.8, 7.8 Hz, NC$H_a\underline{H_b}$), 3.84 (3H, s, ArOC$\underline{H_3}$), 3.33 (3H, s, CHOC$\underline{H_3}$), 3.17–3.04 (2H, m, SC$\underline{H_2}$).
^{13}C{^{1}H} NMR ($CDCl_3$, 100.6 MHz) δ 202.7 (C), 171.1 (C), 157.7 (C), 129.8 (CH), 127.4 (CH), 124.5 (C), 120.6 (CH), 110.6 (CH), 79.2 (CH), 62.0 (CH), 57.6 (CH_3), 56.2 (CH_2), 55.4 (CH_3), 28.7 (CH_2).
HRMS (+ESI): *m/z* calcd. for [M – OMe – N_2]$^+$ $C_{13}H_{13}N_2O_2S_2$: 293.0413; found: 293.0417. *m/z* calcd. for [M – OMe]$^+$ $C_{13}H_{13}N_4O_2S_2$: 321.0474; found: 321.0474.

***N*-[(2*R*,3*R*)-2-Azido-3-(2,3-dihydrobenzo[*b*][1,4]dioxin-6-yl)-3-methoxypropanoyl]-1,3-thiazolidine-2-thione (5h)**

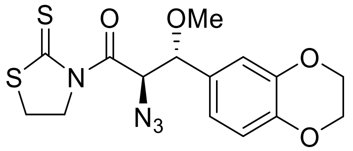

The General Procedure was followed with *N*-(2-azidoacetyl)-1,3-thiazolidine-2-thione (**3**, 101 mg, 0.50 mmol), 6-(dimethoxymethyl)-2,3-dihydrobenzo[*b*][1,4]dioxine (**h**, 115 mg, 0.55 mmol), [(*S*)-Tol-BINAP]$NiCl_2$ (20.2 mg, 25 μmol, 5 mol%), TMSOTf (120 μL, 0.65 mmol) and 2,6-lutidine (90 μL, 0.75 mmol, 1.5 equiv), and the reaction mixture was stirred at 0 °C for 1 h.

The residue (dr 78:22) was purified by column chromatography (80:20 Hexanes/EtOAc) to afford 133 mg (0.35 mmol, 70% yield) of *anti* adduct **5h** and 37 mg (26 μmol, 13% yield) of the *syn* diastereomer.

Yellow solid.
Mp 121–125 °C.
R_f 0.25 (80:20 Hexanes/EtOAc).
Chiral HPLC (Phenomenex Lux® Cellulose-1 column, 25% *i*-PrOH in hexane, flow rate 1.0 mL·min^{-1}): Rt 35.4 min (Major 2*R*,3*R*-isomer) [Rt 24.1 min (minor 2*S*,3*S*-isomer)], 98% *ee*.
$[\alpha]_D^{20}$ –93.4 (*c* 1.00, $CHCl_3$).
IR (ATR) ν 3008, 2923, 2853, 2092, 1694, 1504, 1349, 1310, 1066 cm^{-1}.
^{1}H NMR ($CDCl_3$, 400 MHz) δ 6.93–6.83 (3H, m, Ar<u>H</u>), 6.09 (1H, d, *J* = 8.6 Hz, C<u>H</u>N_3), 4.61 (1H, ddd, *J* = 11.8, 7.7, 3.8 Hz, NC<u>H</u>$_a$H$_b$), 4.52–4.45 (1H, m, NCH$_a$<u>H</u>$_b$), 4.47 (1H, d, *J* = 8.6 Hz, C<u>H</u>OCH_3), 4.27 (4H, s, OC<u>H</u>$_2$C<u>H</u>$_2$O), 3.44–3.38 (1H, m, SC<u>H</u>$_a$H$_b$), 3.26 (1H, ddd, *J* = 11.2, 7.5, 3.8 Hz, SCH$_a$<u>H</u>$_b$), 3.19 (3H, s, CHOC<u>H</u>$_3$).
^{13}C{^{1}H} NMR ($CDCl_3$, 100.6 MHz) δ 201.9 (C), 170.9 (C), 144.1 (C), 143.7 (C), 129.9 (C), 120.8 (CH), 117.6 (CH), 116.4 (CH), 83.7 (CH), 64.3 (CH_2), 64.3 (CH_2), 63.8 (CH), 56.9 (CH_3), 56.2 (CH_2), 28.9 (CH_2).
HRMS (+ESI): *m/z* calcd. for [M – OMe]$^+$ $C_{14}H_{13}N_4O_3S_2$: 349.0424; found: 349.0428. *m/z* calcd. for [M + Na]$^+$ $C_{15}H_{16}N_4NaO_4S_2$: 403.05050; found: 403.0500.

N-[(2R,3R)-2-Azido-3-benzyloxy-3-(2,3-dihydrobenzo[b][1,4]dioxin-6-yl)propanoyl]-1,3-thiazolidine-2-thione (5i)

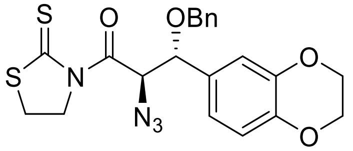

The General Procedure followed with *N*-(2-azidoacetyl)-1,3-thiazolidine-2-thione (**3**, 102 mg, 0.50 mmol), 6-(bis(benzyloxy)methyl)-2,3-dihydrobenzo[*b*][1,4]dioxine (**k**, 200 mg, 0.55 mmol), [(*S*)-Tol-BINAP]$NiCl_2$ (20.4 mg, 25 µmol, 5 mol%), TMSOTf (120 µL, 0.65 mmol) and 2,6-lutidine (90 µL, 0.75 mmol), and the reaction mixture was stirred at –20 °C for 3 h.

The residue (dr 76:24) was purified by column chromatography (80:20 Hexanes/EtOAc) to afford 139 mg (0.30 mmol, 61% yield) of *anti* adduct **5i** and 42 mg (0.09 mmol, 18% yield) of the *syn* diastereomer.

The reaction was also carried out at 3.15 mmol scale (dr 76:24) to afford 875 mg (61% yield) of the proper *anti* adduct.

Yellow oil.

R_f 0.30 (80:20 Hexanes/EtOAc).

Chiral HPLC (Phenomenex Lux® Cellulose-1 column, 40% *i*-PrOH in hexane, flow rate 1.0 mL·min^{-1}): Rt 12.6 min (Major 2*R*,3*R*-isomer) [Rt 21.8 min (minor 2*S*,3*S*-isomer)], 98% *ee*.

$[\alpha]_D^{20}$ –80.5 (*c* 1.00, $CHCl_3$).

IR (ATR) ν 2924, 2872, 2108, 17 02, 1506, 1359, 1284, 1169, 1066, 1003, 887 cm^{-1}.

^{1}H NMR ($CDCl_3$, 400 MHz) δ 7.35–7.23 (5H, m, ArH), 6.98 (1H, d, *J* = 1.7 Hz, ArH), 6.94–6.90 (2H, m, ArH), 6.14 (1H, d, *J* = 8.8 Hz, CHN$_3$), 4.64 (1H, d, *J* = 8.8 Hz, CHOCH$_2$Ph), 4.45 (1H, d, *J* = 11.4 Hz, OCH$_a$H$_b$Ph), 4.46–4.34 (2H, m, NCH$_2$), 4.29 (4H, s, OCH$_2$CH$_2$O), 4.20 (1H, d, *J* = 11.4 Hz, OCH$_a$H$_b$Ph), 3.12–3.00 (2H, m, SCH$_2$).

^{13}C{^{1}H} NMR ($CDCl_3$, 100.6 MHz) δ 202.1 (C), 171.7 (C), 144.3 (C), 143.8 (C), 137.6 (C), 130.0 (C), 128.3 (CH), 127.8 (CH), 127.7 (CH), 120.9 (CH), 117.7 (CH), 116.5 (CH), 82.4 (CH), 70.9 (CH$_2$), 64.3 (CH$_2$), 64.3 (CH$_2$), 64.2 (CH), 56.3 (CH$_2$), 28.9 (CH$_2$).

HRMS (+ESI): *m/z* calcd. for [M – OBn – N$_2$]$^+$ $C_{14}H_{13}N_2O_3S_2$: 321.0362; found: 321.0368. *m/z* calcd. for [M + Na]$^+$ $C_{21}H_{20}N_4NaO_4S_2$: 479.0818; found: 479.0826.

***N*-[(2*R*,3*R*)-2-Azido-3-benzyloxy-3-(3,4-dibenzyloxyphenyl)propanoyl]-1,3-thiazolidine-2-thione (5j)**

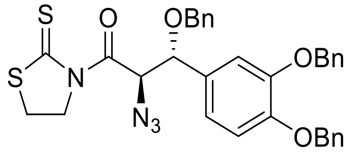

The General Procedure was followed with *N*-(2-azidoacetyl)-1,3-thiazolidine-2-thione (**3**, 101 mg, 0.50 mmol), 3,4-dibenzyloxybenzaldehyde dibenzyl acetal (**j**, 285 mg, 0.55 mmol), [(*S*)-Tol-BINAP]$NiCl_2$ (20.8 mg, 25 μmol, 5 mol%), TMSOTf (225 μL, 1.25 mmol, 2.5 equiv) and 2,6-lutidine (90 μL, 0.75 mmol), and the reaction mixture was stirred at –20 °C for 3 h.

The residue (dr 71:29) was purified by column chromatography (from 90:10 to 70:30 Hexanes/EtOAc) to afford 182 mg (0.30 mmol, 60% yield) of *anti* adduct **5j** and 72 mg (0.12 mmol, 23% yield) of the *syn* diastereomer.

Yellow oil.
$\mathbf{R_f}$ 0.10 (80:20 Hexanes/Et_2O).
Chiral HPLC (Phenomenex Lux® Cellulose-1 column, 40% *i*-PrOH in hexane, flow rate 1.0 mL·min^{-1}): Rt 24.2 min (Major 2*R*,3*R*-isomer) [Rt 43.8 min (minor 2*S*,3*S*-isomer)], 99% *ee*.
$[\alpha]_D^{20}$ –55.2 (*c* 1.0, $CHCl_3$).
IR (ATR) ν 2924, 2853, 2106, 1700, 1508, 1359, 1262, 1165, 1135, 1055, 1001, 734, 695 cm^{-1}.
^{1}H NMR ($CDCl_3$, 400 MHz) δ 7.48–7.44 (4H, m, Ar<u>H</u>), 7.40–7.27 (9H, m, Ar<u>H</u>), 7.18–7.15 (2H, m, Ar<u>H</u>), 7.03 (1H, d, *J* = 1.5 Hz, Ar<u>H</u>), 6.98–6.94 (2H, m, Ar<u>H</u>), 6.12 (1H, d, *J* = 8.8 Hz, C<u>H</u>N_3), 5.20 (2H, s, OC<u>H</u>$_2$Ph), 5.19 (2H, s, OC<u>H</u>$_2$Ph), 4.62 (1H, d, *J* = 8.8 Hz, C<u>H</u>OBn), 4.39–4.31 (2H, m, NC<u>H</u>$_2$), 4.34 (1H, d, *J* = 11.3 Hz, OC<u>H</u>$_a$H$_b$Ph), 4.14 (1H, d, *J* = 11.3 Hz, OCH$_a$<u>H</u>$_b$Ph), 3.06 (1H, ddd, *J* = 10.8, 6.9, 3.8 Hz, SC<u>H</u>$_a$H$_b$), 2.98 (1H, td, *J* = 10.8, 8.1 Hz, SCH$_a$<u>H</u>$_b$).
^{13}C{^{1}H} NMR ($CDCl_3$, 100.6 MHz) δ 202.2 (C), 171.6 (C), 149.7 (C), 149.0 (C), 137.5 (C), 137.0 (C), 136.9 (C), 129.8 (C), 128.5 (CH), 128.5 (CH), 128.5 (CH), 128.3 (CH), 127.8 (CH), 127.8 (CH), 127.7 (CH), 127.4 (CH), 127.3 (CH), 121.2 (CH), 114.6 (CH), 113.9 (CH), 82.6 (CH), 71.2 (CH_2), 71.2 (CH_2), 70.9 (CH_2), 64.0 (CH), 56.3 (CH_2), 28.9 (CH_2).
HRMS (+ESI): *m/z* calcd. for [M – OBn – N_2]$^+$ $C_{26}H_{23}N_2O_3S_2$: 475.1145; found: 475.1147. *m/z* calcd. for [M + Na]$^+$ $C_{33}H_{30}N_4NaO_4S_2$: 633.1601; found: 633.1607.

***N*-[(2*R*,3*R*)-2-Azido-3-methoxy-3-(4-methylphenyl)propanoyl]-1,3-thiazolidine-2-thione (5k)**

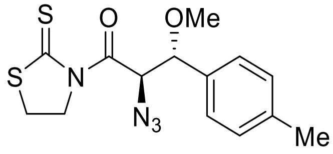

The General Procedure was followed with *N*-(2-azidoacetyl)-1,3-thiazolidine-2-thione (**3**, 51 mg, 0.25 mmol), 4-methylbenzaldehyde dimethyl acetal (**k**, 46 mg, 0.275 mmol), [(*S*)-Tol-BINAP]$NiCl_2$ (10.1 mg, 12.5 μmol, 5 mol%), TMSOTf (60120 μL, 0.325 mmol), and 2,6-lutidine (45 μL, 0.375 mmol) at 0 °C for 16 h.

The residue (dr 66:34) was purified by column chromatography (60:40 Hexanes/CH_2Cl_2) to afford 67 mg (0.20 mmol, 79% yield) of a diastereomeric mixture of adduct **5k** and the *syn* counterpart **7k**.

Yellowish oil.
R_f 0.30 (60:40 Hexanes/CH_2Cl_2).
Chiral HPLC (Phenomenex Lux® Cellulose-1 column, 10% *i*-PrOH in hexane, flow rate 1.0 mL·min^{-1}): Rt 13.5 min (Major 2*R*,3*R*-isomer) [Rt 12.7 min (minor 2*S*,3*S*-isomer)], 98% *ee*.
IR (ATR) ν 2926, 2106, 1702, 1511, 1357, 1154 cm^{-1}.
^{1}H NMR ($CDCl_3$, 400 MHz) δ 7.30–7.26 (2H, m, Ar<u>H</u>), 7.23–7.18 (2H, m, Ar<u>H</u>), 6.12 (1H, d, *J* = 8.7 Hz, C<u>H</u>N_3), 4.60 (1H, ddd, *J* = 11.8, 7.8, 3.9 Hz, NC<u>H_a</u>H_b), 4.55 (1H, d, *J* = 8.7 Hz, C<u>H</u>OCH_3), 4.49 (1H, ddd, *J* = 11.8, 10.5, 7.5 Hz, NCH_a<u>H_b</u>), 3.38 (1H, ddd, *J* = 11.1, 10.5, 7.8 Hz, SC<u>H_a</u>H_b), 3.27–3.23 (1H, m, SCH_a<u>H_b</u>), 3.20 (3H, s, CHOC<u>H</u>$_3$), 2.37 (3H, s, ArC<u>H</u>$_3$).
^{13}C{^{1}H} NMR ($CDCl_3$, 100.6 MHz) δ 202.1 (C), 171.0 (C), 138.9 (C), 133.7 (C), 129.5 (CH), 127.6 (CH), 84.0 (CH), 63.8 (CH), 56.9 (CH_3), 56.2 (CH_2), 28.9 (CH_2), 21.3 (CH_3).
HRMS (+ESI): *m/z* calcd. for [M – OMe – N_2]$^+$ $C_{13}H_{13}N_2OS_2$: 277.0464; found: 277.0468. *m/z* calcd. for [M + Na]$^+$ $C_{14}H_{16}N_4NaO_2S_2$: 359.0602; found: 359.0601.

_N_-[(2_R_,3_S_)-2-Azido-3-methoxy-3-(4-methylphenyl)propanoyl]-1,3-thiazolidine-2-thione (7k)

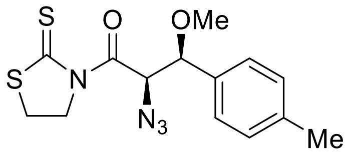

Yellowish oil.

R_f 0.30 (60:40 Hexanes/CH_2Cl_2).

Chiral HPLC (Phenomenex Lux® Cellulose-1 column, 10% *i*-PrOH in hexane, flow rate 1.0 mL·min^{-1}): Rt 21.5 min (Major 2*R*,3*S*-isomer) [Rt 14.5 min (minor 2*S*,3*R*-isomer)], 95% *ee*.

^{1}H NMR ($CDCl_3$, 400 MHz) δ 7.30–7.26 (2H, m, Ar<u>H</u>), 7.23–7.18 (2H, m, Ar<u>H</u>), 6.17 (1H, d, *J* = 6.8 Hz, C<u>H</u>N_3), 4.66 (1H, d, *J* = 6.8 Hz, C<u>H</u>OCH_3), 4.25 (1H, td, *J* = 11.8, 7.3 Hz, NC$\underline{H_a}H_b$), 4.16 (1H, ddd, *J* = 11.8, 7.6, 2.8 Hz, NC$H_a\underline{H_b}$), 3.28 (3H, s, CHOC$\underline{H}_3$), 3.05 (1H, ddd, *J* = 10.9, 7.3, 2.8 Hz, SC$\underline{H_a}H_b$), 2.84 (1H, ddd, *J* = 11.6, 10.9, 7.6 Hz, SC$H_a\underline{H_b}$), 2.36 (3H, s, ArC$\underline{H}_3$).

^{13}C{^{1}H} NMR ($CDCl_3$, 100.6 MHz) δ 201.7 (C), 170.2 (C), 138.7 (C), 133.4 (C), 129.3 (CH), 127.3 (CH), 84.4 (CH), 66.2 (CH), 57.1 (CH_3), 56.2 (CH_2), 28.8 (CH_2), 21.2 (CH_3).

***N*-[(2*R*,3*R*)-2-Azido-3-methoxy-3-(naphthalen-2-yl)propanoyl]-1,3-thiazolidine-2-thione (5l)**

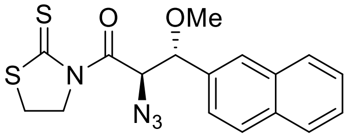

The General Procedure was followed with *N*-(2-azidoacetyl)-1,3-thiazolidine-2-thione (**3**, 101 mg, 0.50 mmol), 2-naphthaldehyde dimethyl acetal (**l**, 111 mg, 0.55 mmol), [(*S*)-Tol-BINAP]$NiCl_2$ (20.3 mg, 25 μmol, 5 mol%), TMSOTf (120 μL, 0.65 mmol) and 2,6-lutidine (90 μL, 0.75 mmol), and the reaction mixture was stirred at 0 °C for 5 h.

The residue (dr 60:40) was purified by column chromatography (80:20 Hexanes/EtOAc) to give 105 mg (0.28 mmol, 56% yield) of *anti* adduct **5l** and 35 mg (90 μmol, 19% yield) of the *syn* diastereomer.

Yellow solid.
Mp 116–118 °C.
R_f 0.20 (80:20 Hexanes/EtOAc).
Chiral HPLC (Phenomenex Lux® Amylose-3 column, 5% *i*-PrOH in hexane, flow rate 1.0 mL·min^{-1}): Rt 24.8 min (Major 2*R*,3*R*-isomer) [Rt 27.2 min (minor 2*S*,3*S*-isomer)], 97% *ee*.
$[\alpha]_D^{20}$ –120.8 (*c* 1.00, $CHCl_3$).
IR (ATR) ν 3052, 2925, 2170, 2105, 1965, 1698, 1600, 1508, 1463, 1192 cm^{-1}.
^{1}H NMR ($CDCl_3$, 400 MHz) δ 7.93–7.83 (4H, m, Ar$\underline{H}$), 7.57–7.47 (3H, m, Ar$\underline{H}$), 6.28 (1H, d, *J* = 8.5 Hz, C$\underline{H}N_3$), 4.76 (1H, d, *J* = 8.5 Hz, C$\underline{H}OCH_3$), 4.58 (1H, ddd, *J* = 11.8, 7.5, 4.1 Hz, NC$\underline{H_a}H_b$), 4.48 (1H, ddd, *J* = 11.8, 10.3, 7.8 Hz, NC$H_a\underline{H_b}$), 3.28–3.17 (2H, m, SC$\underline{H_2}$), 3.26 (3H, s, CHOC$\underline{H_3}$).
^{13}C{^{1}H} NMR ($CDCl_3$, 100.6 MHz) δ 202.1 (C), 171.0 (C), 134.2 (C), 133.7 (C), 133.1 (C), 128.9 (CH), 128.0 (CH), 127.8 (CH), 127.5 (CH), 126.5 (CH), 126.5 (CH), 124.4 (CH), 84.3 (CH), 63.5 (CH), 57.2 (CH_3), 56.2 (CH_2), 28.8 (CH_2).
HRMS (+ESI): *m/z* calcd. for [M – OMe]$^+$ $C_{16}H_{13}N_4OS_2$: 341.0525; found: 341.0528. *m/z* calcd. for [M + Na]$^+$ $C_{17}H_{16}N_4NaO_2S_2$: 395.0607; found: 395.0603.

N-[(2R,3R)-2-Azido-3-methoxy-3-phenylpropanoyl]-1,3-thiazolidine-2-thione (5m)

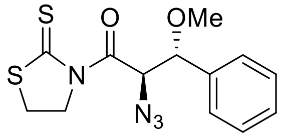

The General Procedure was followed with *N*-(2-azidoacetyl)-1,3-thiazolidine-2-thione (**3**, 101 mg, 0.50 mmol), benzaldehyde dimethyl acetal (**m**, 84 μL, 0.55 mmol), [(*S*)-Tol-BINAP]$NiCl_2$ (20.3 mg, 25 μmol, 5 mol%), TMSOTf (120 μL, 0.65 mmol) and 2,6-lutidine (90 μL, 0.75 mmol), and the reaction mixture was stirred at 0 °C for 3 h.

The residue (dr 62:38) was purified by column chromatography (80:20 Hexanes/EtOAc) to give 94 mg (0.29 mmol, 58% yield) of *anti* adduct **5m** and 58 mg (0.18 mmol, 36% yield) of the *syn* diastereomer **7m**.

Yellow oil.

R_f 0.20 (80:20 Hexanes/EtOAc).

Chiral HPLC (Phenomenex Lux® Cellulose-1 column, 5% *i*-PrOH in hexane, flow rate 1.0 mL·min^{-1}): Rt 23.5 min (Major 2*R*,3*R*-isomer) [Rt 19.5 min (minor 2*S*,3*S*-isomer)], 98% *ee*.

$[\alpha]_D^{20}$ –70.2 (*c* 1.00, $CHCl_3$).

IR (ATR) ν 2952, 2928, 2108, 1708, 1608, 1360, 1101 cm^{-1}.

^{1}H NMR ($CDCl_3$, 400 MHz) δ 7.44–7.36 (5H, m, Ar$\underline{H}$), 6.15 (1H, d, *J* = 8.6 Hz, C$\underline{H}N_3$), 4.62–4.58 (1H, m, NC$\underline{H_a}H_b$), 4.59 (1H, d, *J* = 8.6 Hz, C$\underline{H}OCH_3$), 4.49 (1H, ddd, *J* = 11.8, 10.5, 7.6 Hz, NC$H_a\underline{H_b}$), 3.40–3.34 (1H, m, SC$\underline{H_a}H_b$), 3.25 (1H, ddd, *J* = 11.3, 7.6, 3.9 Hz, SC$H_a\underline{H_b}$), 3.22 (3H, s, CHOC$\underline{H_3}$).

$^{13}C\{^1H\}$ NMR ($CDCl_3$, 100.6 MHz) δ 202.1 (C), 170.8 (C), 136.8 (C), 129.0 (CH), 128.8 (CH), 127.6 (CH), 84.1 (CH), 63.8 (CH), 57.1 (CH_3), 56.2 (CH_2), 28.9 (CH_2).

HRMS (+ESI): *m/z* calcd. for $[M - N_2 + H]^+$ $C_{13}H_{15}N_2O_2S_2$: 295.0569; found: 295.0560. *m/z* calcd. for $[M + Na]^+$ $C_{13}H_{14}N_4NaO_2S_2$: 345.0450; found: 345.0440.

***N*-[(2*R*,3*S*)-2-Azido-3-methoxy-3-phenylpropanoyl]-1,3-thiazolidine-2-thione (7m)**

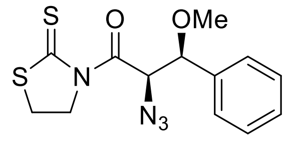

Yellow oil.

R_f 0.15 (80:20 Hexanes/EtOAc).

Chiral HPLC (Phenomenex Lux® Cellulose-1 column, 5% *i*-PrOH in hexane, flow rate 1.0 mL·min^{-1}): Rt 37.5 min (Major 2*R*,3*S*-isomer) [Rt 22.0 min (minor 2*S*,3*R*-isomer)], 94% *ee*.

^{1}H NMR ($CDCl_3$, 400 MHz) δ 7.42–7.36 (5H, m, Ar<u>H</u>), 6.20 (1H, d, *J* = 6.8 Hz, C<u>H</u>N_3), 4.69 (1H, d, *J* = 6.8 Hz, C<u>H</u>OCH_3), 4.24 (1H, td, *J* = 11.9, 7.3 Hz, NC$\underline{H_a}H_b$), 4.14 (1H, ddd, *J* = 11.9, 7.6, 2.8 Hz, NC$H_a\underline{H_b}$), 3.30 (3H, s, CHOC$\underline{H_3}$), 3.03 (1H, ddd, *J* = 11.0, 7.3, 2.8 Hz, SC$\underline{H_a}H_b$), 2.84 (1H, ddd, *J* = 11.7, 11.0, 7.6 Hz, SC$H_a\underline{H_b}$).

^{13}C{^{1}H} NMR ($CDCl_3$, 100.6 MHz) δ 201.8 (C), 170.1 (C), 136.5 (C), 128.9 (CH), 128.6 (CH), 127.4 (CH), 84.6 (CH), 66.2 (CH), 57.2 (CH_3), 56.2 (CH_2), 28.9 (CH_2).

***N*-[(2*R*,3*S*)-2-Azido-3-methoxy-3-(2-thiophenyl)propanoyl]-1,3-thiazolidine-2-thione (5n)**

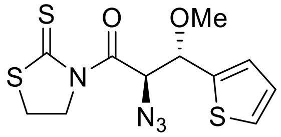

The General Procedure was followed with *N*-(2-azidoacetyl)-1,3-thiazolidine-2-thione (**3**, 101 mg, 0.50 mmol), 2-thiophenecarbaldehyde dimethyl acetal (**n**, 87 mg, 0.55 mmol), [(*S*)-Tol-BINAP]$NiCl_2$ (20.3 mg, 25 μmol, 5 mol%), TMSOTf (120 μL, 0.65 mmol) and 2,6-lutidine (90 μL, 0.75 mmol), and the reaction mixture was stirred at 0 °C for 2 h.

The residue (dr 81:19) was purified by column chromatography (60:40 Hexanes/Et_2O) to afford 88 mg (0.27 mmol, 54% yield) of *anti* adduct **5n** and 24 mg (7 μmol, 14% yield) of the *syn* diastereomer.

Yellow oil.

R_f 0.30 (60:40 Hexanes/Et_2O).

Chiral HPLC (Phenomenex Lux® Cellulose-1 column, 10% *i*-PrOH in hexane, flow rate 1.0 mL·min^{-1}): Rt 18.6 min (Major 2*R*,3*S*-isomer) [Rt 17.1 min (minor 2*S*,3*R*-isomer)], 99% *ee*.

$[\alpha]_D^{20}$ –58.2 (*c* 1.00, $CHCl_3$).

IR (ATR) ν 3095, 2925, 2825, 2107, 1709, 1608, 1458, 1435, 1356, 1304, 1097 cm^{-1}.

^{1}H NMR ($CDCl_3$, 400 MHz) δ 7.40 (1H, ddd, *J* = 5.0, 1.2, 0.6 Hz, Ar$\underline{H}$), 7.16 (1H, ddd, *J* = 3.5, 1.2, 0.6 Hz, Ar$\underline{H}$), 7.05 (1H, dd, *J* = 5.0, 3.5 Hz, Ar$\underline{H}$), 6.20 (1H, d, *J* = 8.4 Hz, C$\underline{H}N_3$), 4.89 (1H, d, *J* = 8.4 Hz, C$\underline{H}OCH_3$), 4.60 (1H, ddd, *J* = 11.9, 7.7, 4.0 Hz, NC$\underline{H_a}H_b$), 4.49 (1H, ddd, *J* = 11.9, 10.4, 7.5 Hz, NC$H_a\underline{H_b}$), 3.42 (1H, ddd, *J* = 11.1, 10.4, 7.7 Hz, SC$\underline{H_a}H_b$), 3.30–3.24 (1H, m, SC$H_a\underline{H_b}$), 3.29 (3H, s, CHOC$\underline{H_3}$).

^{13}C{^{1}H} NMR ($CDCl_3$, 100.6 MHz) δ 202.0 (C), 170.2 (C), 140.2 (C), 127.8 (CH), 127.1 (CH), 127.0 (CH), 79.8 (CH), 64.5 (CH), 57.2 (CH_3), 56.2 (CH_2), 28.9 (CH_2).

HRMS (+ESI): *m/z* calcd. for [M – OMe]$^+$ $C_{10}H_9N_4OS_3$: 296.9933; found: 296.9940. *m/z* calcd. for [M + Na]$^+$ $C_{11}H_{12}N_4NaO_2S_3$: 351.0015; found: 351.0022.

_N_-[(2_R_,3_R_)-2-Azido-3-methoxy-3-(1-(phenylsulfonyl)-1_H_-indol-3-yl)propanoyl]-1,3-thiazolidine-2-thione (5o)

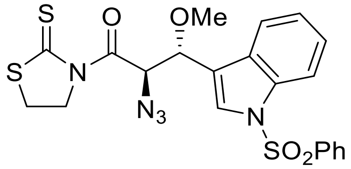

The General Procedure was followed with *N*-(2-azidoacetyl)-1,3-thiazolidine-2-thione (**3**, 51 mg, 0.25 mmol), 1-(phenylsulfonyl)-1*H*-indole-3-carbaldehyde dimethyl acetal (**o**, 91 mg, 0.275 mmol), [(*S*)-Tol-BINAP]$NiCl_2$ (15.2 mg, 19 µmol, 7.5 mol%), TMSOTf (68 µL, 0.375 mmol, 1.5 equiv) and 2,6-lutidine (45 µL, 0.375 mmol), and the reaction mixture was stirred at 0 °C for 16 h.

The residue (dr 74:26) was purified by column chromatography (70:30 Hexanes/EtOAc) to afford 83 mg (0.17 mmol, 66% yield) of *anti* adduct **5o** and 26 mg (52 µmol, 21% yield) of the *syn* diastereomer.

Thick yellow oil.

R_f 0.10 (70:30 Hexanes/EtOAc).

Chiral HPLC (Phenomenex Lux® Cellulose-5 column, 10% *i*-PrOH in hexane, flow rate 1.0 mL·min^{-1}): Rt 40.5 min (Major 2*R*,3*R*-isomer) [Rt 47.1 min (minor 2*S*,3*S*-isomer)], 98% *ee*.

$[\alpha]_D^{20}$ –32.5 (*c* 1.0, $CHCl_3$).

IR (ATR) ν 2924, 2108, 1702, 1446, 1362, 1172, 1055 cm^{-1}.

^{1}H NMR ($CDCl_3$, 400 MHz) δ 8.01 (1H, d, *J* = 8.3 Hz, ArH), 7.89 (2H, dt, *J* = 7.4, 1.3 Hz, ArH), 7.72–7.69 (2H, m, ArH), 7.56–7.51 (1H, m, ArH), 7.44 (2H, dd, *J* = 8.5, 7.1 Hz, ArH), 7.36 (1H, ddd, *J* = 8.5, 7.1, 1.3 Hz, ArH), 7.29–7.25 (1H, m, ArH), 6.44 (1H, d, *J* = 8.6 Hz, CHN_3), 4.87 (1H, d, *J* = 8.6 Hz, $CHOCH_3$), 4.56 (1H, ddd, *J* = 11.9, 7.6, 4.1 Hz, NCH_aH_b), 4.46 (1H, ddd, *J* = 11.9, 10.3, 7.7 Hz, NCH_aH_b), 3.31–3.18 (2H, m, SCH_2), 3.22 (3H, s, $CHOCH_3$).

^{13}C{^{1}H} NMR ($CDCl_3$, 100.6 MHz) δ 202.2 (C), 170.4 (C), 137.8 (C), 135.5 (C), 134.0 (CH), 129.3 (CH), 128.5 (C), 126.7 (CH), 126.4 (CH), 125.4 (CH), 123.8 (CH), 120.6 (CH), 118.2 (C), 113.8 (CH), 77.9 (CH), 61.9 (CH), 57.1 (CH_3), 56.2 (CH_2), 28.7 (CH_2).

HRMS (+ESI): *m/z* calcd. for $[M + Na]^+$ $C_{21}H_{19}N_5NaO_4S_3$: 524.0491; found: 524.0492.

5. TMSOTf–Mediated Reactions Catalyzed by [(*R*)-DTBM-SEGPHOS]$NiCl_2$

5.1. General Procedure

A solution of *N*-(2-azidoacetyl)-1,3-oxazolidine-2-thione (2, 1.0 equiv), an aromatic dialkyl acetal (1.1 equiv) and [(*R*)-DTBM-SEGPHOS]$NiCl_2$ (2–5 mol%) in CH_2Cl_2 (0.25 M) was cooled at 0 °C under a N_2 athmosphere. Then, neat TMSOTf (1.3 equiv) was added dropwise to the stirring green-brown solution followed by the addition of 2,6-lutidine (1.5 equiv), and the resultant dark green mixture was stirred at 0 °C until completion.

For a 0.5 mmol scale reaction, the mixture was quenched with sat NH_4Cl (2 mL) and partitioned in CH_2Cl_2 (15 mL) and water (15 mL). The aqueous layer was then extracted with neat CH_2Cl_2 (2 × 15 mL), and the combined organic extracts were dried (Na_2SO_4) and concentrated *in vacuo*. Finally, the crude residue was purified by flash column chromatography to yield the named compound as a single enantiomer.

The *anti/syn* diasteromeric ratio (dr) was established by ^{1}H NMR analysis of the crude mixture. Similarly, the enantiomeric purity (*ee*) of the *syn* compound isolated was established by chiral HPLC analysis of the purified products of both the racemic and the enantioselective reactions.

5.2. Physical and Spectroscopic Data of the Resultant Products

***N*-[(2*S*,3*R*)-2-Azido-3-methoxy-3-(4-methoxyphenyl)propanoyl]-1,3-oxazolidine-2-thione (*ent*-8a)**

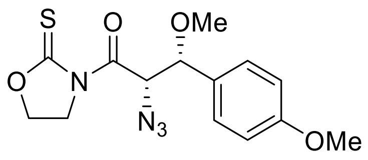

The General Procedure was followed with *N*-(2-azidoacetyl)-1,3-oxazolidine-2-thione (**4**, 93 mg, 0.50 mmol), 4-methoxybenzaldehyde dimethyl acetal (**a**, 95 µL, 0.55 mmol), [(*R*)-DTBM-SEGPHOS]$NiCl_2$ (33.3 mg, 25 µmol, 5 mol%), TMSOTf (120 µL, 0.65 mmol), and 2,6-lutidine (90 µL, 0.75 mmol) at 0 °C for 2 h.

The residue (dr 23:77) was purified by column chromatography (80:20 Hexanes/EtOAc) to afford 103 mg (0.30 mmol, 61% yield) of adduct ***ent*-8a** and 37 mg (11 mmol, 22% yield) of the *anti* diastereomer.

The reaction was also carried out at 5.0 mmol scale (dr 24:76) to afford 1064 mg (63% yield) of the *syn* adduct ***ent*-8a**.

White solid.
Mp 110–120 °C.
R_f 0.15 (80:20 Hexanes/EtOAc).
Chiral HPLC (Phenomenex Lux® Cellulose-1 column, 15% *i*-PrOH in hexane, flow rate 1.0 mL·min^{-1}): Rt 30.4 min (Major 2*S*,3*R*-isomer) [Rt 50.7 min (minor 2*R*,3*S*-isomer)], 96% *ee*.
$[\alpha]_D^{20}$ –40.4 (*c* 0.50, $CHCl_3$).
IR (ATR) ν 2955, 2922, 2852, 2111, 1704, 1610, 1367, 1084 cm^{-1}.
^{1}H NMR ($CDCl_3$, 400 MHz) δ 7.37–7.34 (2H, m, Ar<u>H</u>), 6.91–6.89 (2H, m, Ar<u>H</u>), 6.35 (1H, d, *J* = 6.3 Hz, C<u>H</u>N_3), 4.78 (1H, d, *J* = 6.3 Hz, C<u>H</u>OCH_3), 4.52 (1H, td, *J* = 9.1, 5.4 Hz, OC$\underline{H_a}H_b$), 4.26 (1H, q, *J* = 9.0 Hz, OC$H_a\underline{H_b}$), 4.20–4.14 (1H, m, NC$\underline{H_a}H_b$), 3.95–3.91 (1H, m, NC$H_a\underline{H_b}$), 3.81 (3H, s, ArOC$\underline{H}_3$), 3.28 (3H, s, CHOC$\underline{H}_3$).
^{13}C{^{1}H} NMR ($CDCl_3$, 100.6 MHz) δ 184.9 (C), 169.5 (C), 160.0 (C), 128.8 (CH), 128.3 (C), 114.0 (CH), 83.6 (CH), 66.7 (CH_2), 64.4 (CH), 57.1 (CH_3), 55.3 (CH_3), 47.3 (CH_2).
HRMS (+ESI): *m/z* calcd. for [M – OMe]$^+$ $C_{13}H_{13}N_4O_3S$: 305.0703; found: 305.0704. *m/z* calcd. for [M + Na]$^+$ $C_{14}H_{16}N_4NaO_4S$: 359.0784; found: 359.0783.

***N*-[(2*S*,3*S*)-3-Allyloxy-2-azido-3-(4-methoxyphenyl)propanoyl]-1,3-oxazolidine-2-thione (*ent*-6b)**

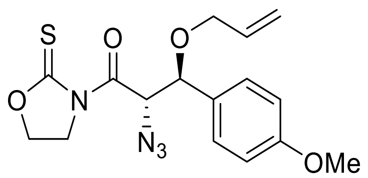

The General Procedure was followed with *N*-(2-azidoacetyl)-1,3-oxazolidine-2-thione (**4**, 47 mg, 0.25 mmol), 4-methoxybenzaldehyde diallyl acetal (**b**, 65 mg, 0.275 mmol), [(*R*)-DTBM-SEGPHOS]$NiCl_2$ (16.4 mg, 12.5 µmol, 5 mol%), TMSOTf (60 µL, 0.325 mmol) and 2,6-lutidine (45 µL, 0.375 mmol), and the reaction mixture was stirred at 0 °C for 2 h.

The residue (dr 54:46) was purified by column chromatography (80:20 Hexanes/EtOAc) to give 45 mg (0.12 mmol, 50% yield) of *anti* adduct ***ent*-6b** and 35 mg (0.10 mmol, 38% yield) of the *syn* diastereomer.

Pale white oil.

R_f 0.20 (80:20 Hexanes/EtOAc).

Chiral HPLC (Phenomenex Lux® Cellulose-1 column, 15% *i*-PrOH in hexane, flow rate 1.0 mL·min^{-1}): Rt 17.5 min (Major 2*S*,3*S*-isomer) [Rt 18.7 min (minor 2*R*,3*R*-isomer)], 98% *ee*.

$[\alpha]_D^{20}$ +52.1 (*c* 1.00, $CHCl_3$).

IR (ATR) ν 2924, 2110, 1702, 1610, 1511, 1368, 1249, 1172, 1023, 835 cm^{-1}.

^{1}H NMR ($CDCl_3$, 400 MHz) δ 7.39–7.35 (2H, m, Ar<u>H</u>), 6.96–6.93 (2H, m, Ar<u>H</u>), 6.52 (1H, d, *J* = 8.9 Hz, C<u>H</u>N_3), 5.77 (1H, dddd, *J* = 17.4, 10.4, 6.1, 4.8 Hz, $OCH_2C\underline{H}=CH_2$), 5.17 (1H, dq, *J* = 17.4, 1.7 Hz, $OCH_2CH=C\underline{H}_aH_b$), 5.12 (1H, dq, *J* = 10.4, 1.7 Hz, $OCH_2CH=CH_a\underline{H}_b$), 4.70 (1H, d, *J* = 8.9 Hz, $C\underline{H}OCH_2CH=CH_2$), 4.61 (1H, td, *J* = 9.0, 6.3 Hz, $OC\underline{H}_aH_b$), 4.52 (1H, q, *J* = 9.0 Hz, $OCH_a\underline{H}_b$), 4.33–4.19 (2H, m, $NC\underline{H}_2$), 3.90 (1H, ddt, *J* = 12.9, 4.8, 1.6 Hz, $OC\underline{H}_aH_bCH=CH_2$), 3.83 (3H, s, $ArOC\underline{H}_3$), 3.73 (1H, ddt, *J* = 12.9, 6.1, 1.6 Hz, $OCH_a\underline{H}_bCH=CH_2$).

^{13}C{^{1}H} NMR ($CDCl_3$, 100.6 MHz) δ 185.3 (C), 170.5 (C), 160.2 (C), 134.1 (CH), 129.1 (CH), 128.9 (C), 116.9 (CH_2), 114.2 (CH), 81.0 (CH), 69.4 (CH_2), 66.7 (CH_2), 62.3 (CH), 55.3 (CH_3), 47.2 (CH_2).

HRMS (+ESI): *m/z* calcd. for $[M - N_2 + H]^+$ $C_{16}H_{19}N_2O_4S$: 335.1060; found: 335.1061. *m/z* calcd. for $[M + Na]^+$ $C_{16}H_{18}N_4NaO_4S$: 385.0941; found: 385.0945.

***N*-[(2*S*,3*S*)-2-Azido-3-benzyloxy-3-(4-methoxyphenyl)propanoyl]-1,3-oxazolidine-2-thione (*ent*-6c)**

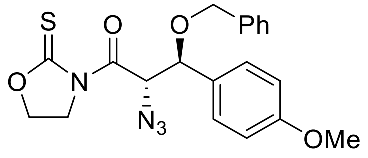

The General Procedure was followed with *N*-(2-azidoacetyl)-1,3-oxazolidine-2-thione (**4**, 93 mg, 0.50 mmol), 4-methoxybenzaldehyde dibenzyl acetal (**c**, 184 mg, 0.55 mmol), [(*R*)-DTBM-SEGPHOS]$NiCl_2$ (32.8 mg, 25 μmol, 5 mol%), TMSOTf (120 μL, 0.65 mmol) and 2,6-lutidine (90 μL, 0.75 mmol), and the reaction mixture was stirred at –20 °C for 2 h.

The residue (dr 65:35) was purified by column chromatography (80:20 Hexanes/EtOAc) to give 123 mg (0.30 mmol, 60% yield) of *anti* adduct ***ent*-6c** and 55 mg (0.13 mmol, 27% yield) of the *syn* diastereomer.

Yellowish oil.

R_f 0.25 (80:20 Hexanes/EtOAc).

Chiral HPLC (Phenomenex Lux® Cellulose-1 column, 20% *i*-PrOH in hexane, flow rate 1.0 mL·min^{-1}): Rt 26.5 min (Major 2*S*,3*S*-isomer) [Rt 17.6 min (minor 2*R*,3*R*-isomer)], 99% *ee*.

$[\alpha]_D^{20}$ +47.2 (*c* 1.00, $CHCl_3$).

IR (ATR) ν 2922, 2853, 2106, 1702, 1610, 1511, 1366, 1247, 1170, 1023, 835 cm^{-1}.

^{1}H NMR ($CDCl_3$, 400 MHz) δ 7.44–7.41 (2H, m, Ar<u>H</u>), 7.33–7.27 (3H, m, Ar<u>H</u>), 7.22–7.19 (2H, m, Ar<u>H</u>), 6.99–6.96 (2H, m, Ar<u>H</u>), 6.58 (1H, d. *J* = 9.0 Hz, C<u>H</u>N_3), 4.73 (1H, d, *J* = 9.0 Hz, C<u>H</u>OBn), 4.56–4.50 (1H, m, OC<u>H</u>$_a$H$_b$), 4.43 (1H, d, *J* = 11.4 Hz, OC<u>H</u>$_a$H$_b$Ph), 4.32–4.18 (2H, m, OCH$_a$<u>H</u>$_b$ & NC<u>H</u>$_a$H$_b$), 4.21 (1H, d, *J* = 11.4 Hz, OCH$_a$<u>H</u>$_b$Ph), 4.09 (1H, ddd, *J* = 11.0, 8.6, 5.7 Hz, NCH$_a$<u>H</u>$_b$), 3.84 (3H, s, ArOC<u>H$_3$</u>).

^{13}C{^{1}H} NMR ($CDCl_3$, 100.6 MHz) δ 185.4 (C), 170.7 (C), 160.3 (C), 137.6 (C), 129.2 (CH), 128.8 (C), 128.3 (CH), 127.7 (CH), 127.6 (CH), 114.3 (CH), 81.8 (CH), 70.7 (CH_2), 66.6 (CH_2), 62.4 (CH), 55.3 (CH_3), 47.2 (CH_2).

HRMS (+ESI): *m/z* calcd. for [M – OBn – N_2]$^+$ $C_{13}H_{13}N_2O_3S$: 277.0641; found: 277.0643. *m/z* calcd. for [M – OBn]$^+$ $C_{13}H_{13}N_4O_3S$: 305.0703; found: 305.0705. *m/z* calcd. for [M + Na]$^+$ $C_{20}H_{20}N_4NaO_4S$: 435.1097; found: 435.1108.

***N*-[(2*S*,3*R*)-2-Azido-3-(4-benzyloxyphenyl)-3-methoxypropanoyl]-1,3-oxazolidine-2-thione (*ent*-8d)**

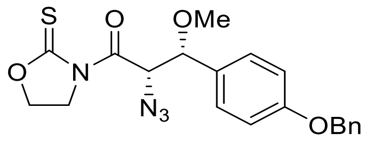

The General Procedure was followed with *N*-(2-azidoacetyl)-1,3-oxazolidine-2-thione (**4**, 47 mg, 0.25 mmol), 4-benzyloxybenzaldehyde dimethyl acetal (**d**, 71 mg, 0.275 mmol), [(*R*)-DTBM-SEGPHOS]$NiCl_2$ (16.4 mg, 12.5 μmol, 5 mol%), TMSOTf (60 μL, 0.325 mmol) and 2,6-lutidine (60 μL, 0.375 mmol), and the reaction mixture was stirred at 0 °C for 2 h.

The residue (dr 21:79) was purified by column chromatography (from 80:20 to 70:30 Hexanes/EtOAc) to give 65 mg (0.16 mmol, 63% yield) of *syn* adduct ***ent*-8d** and 21 mg (51 μmol, 20% yield) of the *anti* diastereomer.

Salmon oil.
R_f 0.30 (70:30 Hexanes/EtOAc).
Chiral HPLC (Phenomenex Lux® Cellulose-5 column 10% *i*-PrOH in hexane, flow rate 1.0 mL·min^{-1}): Rt 30.0 min (Major 2*S*,3*R*-isomer) [Rt 35.8 min (minor 2*R*,3*R*-isomer)], >98% *ee*.
$[\alpha]_D^{20}$ –90.7 (*c* 1.00, $CHCl_3$).
IR (ATR) ν 2920, 2087, 1698, 1608, 1511, 1355, 1170, 1023 cm^{-1}.
^{1}H NMR ($CDCl_3$, 400 MHz) δ 7.44–7.32 (7H, m, Ar<u>H</u>), 6.99–6.96 (2H, m, Ar<u>H</u>), 6.35 (1H, d, *J* = 6.3 Hz, C<u>H</u>N_3), 5.07 (2H, s, OC<u>H</u>$_2$Ph), 4.78 (1H, d, *J* = 6.3 Hz, C<u>H</u>OCH_3), 4.50–4.46 (1H, m, OC<u>H</u>$_a$H$_b$), 4.21–4.11 (2H, m, OCH$_a$<u>H</u>$_b$ & NC<u>H</u>$_a$H$_b$), 3.91–3.81 (1H, m, NCH$_a$<u>H</u>$_b$), 3.28 (3H, s, CHOC<u>H</u>$_3$).
^{13}C{^{1}H} NMR ($CDCl_3$, 100.6 MHz) δ 184.8 (C), 169.4 (C), 159.1 (C), 136.7 (C), 128.8 (CH), 128.6 (CH), 128.5 (C), 128.1 (CH), 127.5 (CH), 114.8 (CH), 83.6 (CH), 70.0 (CH_2), 66.6 (CH_2), 64.4 (CH), 57.1 (CH_3), 47.2 (CH_2).
HRMS (+ESI): *m/z* calcd. for [M – OMe]$^+$ $C_{19}H_{17}N_4O_3S$: 381.1016; found: 381.1009. *m/z* calcd. for [M + Na]$^+$ $C_{20}H_{20}N_4NaO_4S$: 435.1097; found: 435.1091.

***N*-[(2*S*,3*R*)-2-Azido-3-(4-(*tert*-butyldimethylsilyloxy)phenyl)-3-methoxypropanoyl]-1,3-oxazolidine-2-thione (*ent*-8e)**

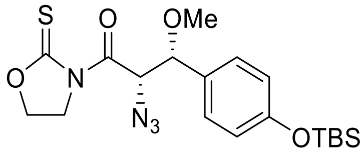

The General Procedure was followed with *N*-(2-azidoacetyl)-1,3-oxazolidine-2-thione (**4**, 47 mg, 0.25 mmol), 4-(*tert*-butyldimethylsilyloxy)benzaldehyde dimethyl acetal (**e**, 78 mg, 0.275 mmol), [(*R*)-DTBM-SEGPHOS]$NiCl_2$ (16.4 mg, 12.5 µmol, 5 mol%), TMSOTf (60 µL, 0.325 mmol) and 2,6-lutidine (45 µL, 0.375 mmol), and the reaction mixture was stirred at 0 °C for 2 h.

The residue (dr 17:83) was purified by column chromatography (from 90:10 to 70:30 Hexanes/EtOAc) to give 76 mg (0.17 mmol, 70% yield) of *syn* adduct ***ent*-8e** and 15 mg (34 µmol, 14% yield) of the *anti* diastereomer.

Thick salmon oil.
R_f 0.35 (70:30 Hexanes/EtOAc).
Chiral HPLC (Phenomenex Lux® Cellulose-1 column, 20% *i*-PrOH in hexane, flow rate 1.0 mL·min^{-1}): Rt 8.8 min (Major 2*S*,3*R*-isomer) [Rt 22.4 min (minor 2*R*,3*S*-isomer)], 99% *ee*.
$[\alpha]_D^{20}$ –85.9 (*c* 1.00, $CHCl_3$).
IR (ATR) ν 2928, 2108, 1702, 1605, 1508, 1362, 1165, 1096 cm^{-1}.
^{1}H NMR ($CDCl_3$, 400 MHz) δ 7.29–7.27 (2H, m, Ar<u>H</u>), 6.85–6.81 (2H, m, Ar<u>H</u>), 6.36 (1H, d, *J* = 6.4 Hz, C<u>H</u>N_3), 4.76 (1H, d, *J* = 6.4 Hz, C<u>H</u>OCH_3), 4.54–4.49 (1H, m, OC$\underline{H}_a H_b$), 4.25 (1H, dt, *J* = 9.4, 8.8 Hz, OC$H_a\underline{H}_b$), 4.16 (1H, dt, *J* = 11.1, 9.4 Hz, NC$\underline{H}_a H_b$), 3.89 (1H, ddd, *J* = 11.1, 8.8, 5.4 Hz, NC$H_a\underline{H}_b$), 3.27 (3H, s, ArOC$\underline{H}_3$), 0.98 (9H, s, SiC(C$\underline{H}_3$)$_3$), 0.20 (6H, s, Si(C$\underline{H}_3$)$_2$).
^{13}C{^{1}H} NMR ($CDCl_3$, 100.6 MHz) δ 184.8 (C), 169.5 (C), 156.2 (C), 128.8 (C), 128.8 (CH), 120.0 (CH), 83.6 (CH), 66.6 (CH_2), 64.4 (CH), 57.1 (CH_3), 47.2 (CH_2), 25.6 (CH_3), 18.2 (C), -4.4 (CH_3).
HRMS (+ESI): *m/z* calcd. for [M – OMe]$^+$ $C_{18}H_{25}N_4O_3SSi$: 405.1411; found: 405.1405. *m/z* calcd. for [M + Na]$^+$ $C_{19}H_{28}N_4NaO_4SSi$: 459.1493; found: 459.1487.

***N*-[(2*S*,3*R*)-2-Azido-3-methoxy-3-(3-methoxyphenyl)propanoyl]-1,3-oxazolidine-2-thione (*ent*-8f)**

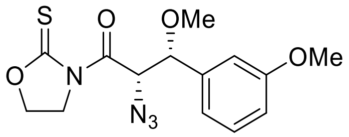

The General Procedure was followed with *N*-(2-azidoacetyl)-1,3-oxazolidine-2-thione (**4**, 93 mg, 0.50 mmol), 3-methoxybenzaldehyde dimethyl acetal (**f**, 100 mg, 0.55 mmol), [(*R*)-DTBM-SEGPHOS]$NiCl_2$ (32.7 mg, 25 µmol, 5 mol%), TMSOTf (120 µL, 0.65 mmol) and 2,6-lutidine (90 µL, 0.75 mmol), and the reaction mixture was stirred at 0 °C for 5 h.

The residue (dr 35:65) was purified by column chromatography (80:20 Hexanes/EtOAc) to afford 93 mg (0.27 mmol, 55% yield) of the *syn* adduct ***ent*-8f** and 36 mg (0.11 mmol, 21% yield) of the *anti* diastereomer ***ent*-6f**.

Light brown oil.

R_f 0.15 (80:20 Hexanes/EtOAc).

Chiral HPLC (Phenomenex Lux® Cellulose-5 column, 10% *i*-PrOH in hexane, flow rate 1.0 mL·min^{-1}): Rt 21.7 min (Major 2*S*,3*R*-isomer) [Rt 25.8 min (minor 2*R*,3*S*-isomer)], 97% *ee*.

$[\alpha]_D^{20}$ –102.6 (*c* 1.00, $CHCl_3$).

IR (ATR) ν 2923, 2107, 1703, 1601, 1372, 1257, 1097 cm^{-1}.

^{1}H NMR ($CDCl_3$, 400 MHz) δ 7.28 (1H, t, *J* = 7.6 Hz, Ar<u>H</u>), 7.02 (1H, dd, *J* = 2.6, 1.6 Hz, Ar<u>H</u>), 6.99 (1H, dd, *J* = 7.6, 1.1, Ar<u>H</u>), 6.90–6.87 (1H, m, Ar<u>H</u>), 6.39 (1H, d, *J* = 6.2 Hz, C<u>H</u>N_3), 4.81 (1H, d, *J* = 6.2 Hz, C<u>H</u>OCH_3), 4.55–4.49 (1H, m, OC$\underline{H_a}H_b$), 4.28–4.21 (1H, m, OC$H_a\underline{H_b}$), 4.20–4.13 (1H, m, NC$\underline{H_a}H_b$), 3.91 (1H, ddd, *J* = 11.0, 8.5, 5.2 Hz, NC$H_a\underline{H_b}$), 3.83 (3H, s, ArOC$\underline{H_3}$), 3.32 (3H, s, CHOC$\underline{H_3}$).

$^{13}C\{^1H\}$ NMR ($CDCl_3$, 100.6 MHz) δ 184.9 (C), 169.4 (C), 159.8 (C), 138.0 (C), 129.6 (CH), 119.8 (CH), 114.9 (CH), 112.1 (CH), 83.9 (CH), 66.7 (CH_2), 64.4 (CH), 57.4 (CH_3), 55.3 (CH_3), 47.3 (CH_2).

HRMS (+ESI): *m/z* calcd. for [M – OMe]$^+$ $C_{13}H_{13}N_4O_3S$: 305.0703; found: 305.0705. *m/z* calcd. for [M – N_2 + H]$^+$ $C_{14}H_{17}N_2O_4S$: 309.0904; found: 309.0903.

_N_-[(2_S_,3_S_)-2-Azido-3-methoxy-3-(3-methoxyphenyl)propanoyl]-1,3-oxazolidine-2-thione (_ent_-6f)

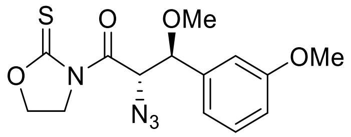

White solid.

Mp 113–116 °C.

R_f 0.20 (80:20 Hexanes/EtOAc).

Chiral HPLC (Phenomenex Lux® Cellulose-5 column, 10% *i*-PrOH in hexane, flow rate 1.0 mL·min^{-1}): Rt 19.0 min (Major 2*S*,3*S*-isomer) [Rt 17.1 min (minor 2*R*,3*R*-isomer)], 96% *ee*.

^{1}H NMR ($CDCl_3$, 400 MHz) δ 7.34 (1H, ddd, *J* = 8.2, 7.5, 0.4 Hz, Ar<u>H</u>), 7.02 (1H, dddd, *J* = 7.5, 1.5, 1.0, 0.4 Hz, Ar<u>H</u>), 7.00 (1H, dd, *J* = 2.6, 1.5, Ar<u>H</u>), 6.93 (1H, ddd, *J* = 8.2, 2.6, 1.0 Hz, Ar<u>H</u>), 6.49 (1H, d, *J* = 8.9 Hz, C<u>H</u>N_3), 4.64–4.50 (3H, m, C<u>H</u>OCH_3 & OC<u>H</u>$_2$), 4.31–4.23 (2H, m, NC<u>H</u>$_2$), 3.84 (3H, s, ArOC<u>H</u>$_3$), 3.21 (3H, s, CHOC<u>H</u>$_3$).

^{13}C{^{1}H} NMR ($CDCl_3$, 100.6 MHz) δ 185.2 (C), 170.1 (C), 160.0 (C), 138.5 (C), 129.8 (CH), 120.2 (CH), 114.7 (CH), 113.0 (CH), 83.5 (CH), 66.6 (CH_2), 61.9 (CH), 57.0 (CH_3), 55.3 (CH_3), 47.2 (CH_2).

***N*-[(2*S*,3*R*)-2-Azido-3-methoxy-3-(2-methoxyphenyl)propanoyl]-1,3-oxazolidine-2-thione (*ent*-8g)**

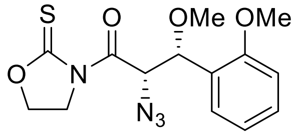

The General Procedure was followed with *N*-(2-azidoacetyl)-1,3-oxazolidine-2-thione (**4**, 93 mg, 0.50 mmol), 2-methoxybenzaldehyde dimethyl acetal (**g**, 100 mg, 0.55 mmol), [(*R*)-DTBM-SEGPHOS]$NiCl_2$ (65.5 mg, 50 μmol, 10 mol%), TMSOTf (120 μL, 0.65 mmol) and 2,6-lutidine (90 μL, 0.75 mmol), and the reaction mixture was stirred at 0 °C for 16 h.

The residue (dr 21:79) was purified by column chromatography (85:15 Hexanes/EtOAc) to afford 103 mg (0.31 mmol, 61% yield) of the *syn* adduct ***ent*-8g** and 43 mg (0.13 mmol, 26% yield) of the *anti* diastereomer ***ent*-6g**.

Pale brown solid.

Mp 90–100 °C.

R_f 0.10 (85:15 Hexanes/EtOAc).

Chiral HPLC (Phenomenex Lux® Cellulose-1 column, 7% *i*-PrOH in hexane, flow rate 1.0 mL·min^{-1}): Rt 52.1 min (Major 2*S*,3*R*-isomer) [Rt 60.3 min (minor 2*R*,3*S*-isomer)], 98% *ee*.

$[\alpha]_D^{20}$ –71.7 (*c* 1.00, $CHCl_3$).

IR (ATR) ν 2955, 2920, 2853, 2104, 1720, 1600, 1356, 1089 cm^{-1}.

^{1}H NMR ($CDCl_3$, 400 MHz) δ 7.47 (1H, dd, *J* = 7.4, 1.8 Hz, ArH), 7.33 (1H, ddd, *J* = 8.2, 7.4, 1.8 Hz, ArH), 7.01 (1H, td, *J* = 7.4, 1.1 Hz, ArH), 6.89 (1H, dd, *J* = 8.3, 1.1 Hz, ArH), 6.51 (1H, d, *J* = 6.2 Hz, C$\underline{H}N_3$), 5.20 (1H, d, *J* = 6.2 Hz, C$\underline{H}OCH_3$), 4.53 (1H, td, *J* = 8.9, 5.0 Hz, OC$\underline{H_a}H_b$), 4.27 (1H, dt, *J* = 9.8, 8.9 Hz, OC$H_a\underline{H_b}$), 4.16 (1H, ddd, , *J* = 11.0, 9.8, 9.2 Hz, NC$\underline{H}_aH_b$), 3.91–3.86 (1H, m, NC$H_a\underline{H}_b$), 3.84 (3H, s, ArOC$\underline{H}_3$), 3.31 (3H, s, CHOC$\underline{H}_3$).

^{13}C{^{1}H} NMR ($CDCl_3$, 100.6 MHz) δ 184.8 (C), 169.6 (C), 157.3 (C), 129.8 (CH), 128.5 (CH), 124.3 (C), 120.7 (CH), 110.4 (CH), 78.8 (CH), 66.7 (CH_2), 63.7 (CH), 57.5 (CH_3), 55.3 (CH_3), 47.5 (CH_2).

HRMS (+ESI): *m/z* calcd. for $[M - OMe]^+$ $C_{13}H_{13}N_4O_3S$: 305.0703; found: 305.0702. *m/z* calcd. for $[M + Na]^+$ $C_{14}H_{16}N_4NaO_4S$: 359.0784; found: 359.0782.

***N*-[(2*S*,3*S*)-2-Azido-3-methoxy-3-(2-methoxyphenyl)propanoyl]-1,3-oxazolidine-2-thione (*ent*-6g)**

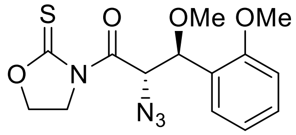

Pale oil.

R_f 0.15 (85:15 Hexanes/EtOAc).

Chiral HPLC (Phenomenex Lux® Cellulose-1 column, 7% *i*-PrOH in hexane, flow rate 1.0 mL·min^{-1}): Rt 42.7 min (Major 2*S*,3*S*-isomer) [Rt 37.4 min (minor 2*R*,3*R*-isomer)], 99% *ee*.

^{1}H NMR ($CDCl_3$, 400 MHz) δ 7.41 (1H, dd, *J* = 7.5, 1.8 Hz, ArH), 7.36–7.31 (1H, m, ArH), 7.01 (1H, td, *J* = 7.5, 1.0 Hz, ArH), 6.91 (1H, dd, *J* = 8.3, 1.0 Hz, ArH), 6.52 (1H, d, *J* = 7.0 Hz, C<u>H</u>N_3), 5.18 (1H, d, *J* = 7.0 Hz, C<u>H</u>OCH_3), 4.53 (1H, td, *J* = 9.0, 5.6 Hz, $OC\underline{H_a}H_b$), 4.35 (1H, q, *J* = 9.0 Hz, $OCH_a\underline{H_b}$), 4.26–4.10 (2H, m, $NC\underline{H_2}$), 3.86 (3H, s, $ArOC\underline{H_3}$), 3.28 (3H, s, $CHOC\underline{H_3}$).

^{13}C{^{1}H} NMR ($CDCl_3$, 100.6 MHz) δ 185.0 (C), 170.2 (C), 157.9 (C), 129.9 (CH), 128.1 (CH), 124.4 (C), 120.7 (CH), 110.7 (CH), 78.1 (CH), 66.5 (CH_2), 60.9 (CH), 57.3 (CH_3), 55.4 (CH_3), 47.2 (CH_2).

***N*-[(2*S*,3*R*)-2-Azido-3-(2,3-dihydrobenzo[*b*][1,4]dioxin-6-yl)-3-methoxypropanoyl]-1,3-oxazolidine-2-thione (*ent*-8h)**

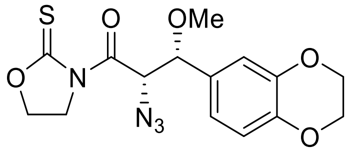

The General Procedure was followed with *N*-(2-azidoacetyl)-1,3-oxazolidine-2-thione (**4**, 93 mg, 0.50 mmol), 6-(dimethoxymethyl)-2,3-dihydrobenzo[b][1,4]dioxine (**h**, 115 mg, 0.55 mmol), [(*R*)-DTBM-SEGPHOS]$NiCl_2$ (33.1 mg, 25 µmol, 5 mol%), TMSOTf (120 µL, 0.65 mmol) and 2,6-lutidine (90 µL, 0.75 mmol, 1.5 equiv), and the reaction mixture was stirred at 0 °C for 3 h.

The residue (dr 28:72) was purified by column chromatography (80:20 Hexanes/EtOAc) to afford 113 mg (0.31 mmol, 62% yield) of *syn* adduct ***ent*-8h** and 49 mg (0.13 mmol, 25% yield) of the *anti* diastereomer.

Pale white solid.
Mp 129–132 °C.
R_f 0.15 (80:20 Hexanes/EtOAc).
Chiral HPLC (Phenomenex Lux® Cellulose-1 column, 30% *i*-PrOH in hexane, flow rate 1.0 mL·min^{-1}): Rt 47.3 min (Major 2*S*,3*R*-isomer) [Rt 36.6 min (minor 2*R*,3*S*-isomer)], 98% *ee*.
$[\alpha]_D^{20}$ –117.6 (*c* 1.00, $CHCl_3$).
IR (ATR) ν 2924, 2853, 2110, 1702, 1508, 1306, 1284, 1204, 1098, 1066, 889 cm^{-1}.
^{1}H NMR ($CDCl_3$, 400 MHz) δ 6.97 (1H, d, *J* = 2.0 Hz, ArH), 6.90 (1H, dd, *J* = 8.3, 2.0 Hz, ArH), 6.85 (1H, d, *J* = 8.3 Hz, ArH), 6.25 (1H, d, *J* = 5.9 Hz, C$\underline{H}N_3$), 4.77 (1H, d, *J* = 5.9 Hz, C$\underline{H}OCH_3$), 4.56 (1H, ddd, *J* = 9.4, 8.9, 5.5 Hz, OC$\underline{H_a}H_b$), 4.35 (1H, q, *J* = 9.1 Hz, OC$H_a\underline{H_b}$), 4.26 (4H, s, OC$\underline{H_2}$C$\underline{H_2}$O), 4.21 (1H, dt, *J* = 11.3, 9.4 Hz, NC$\underline{H_a}H_b$,), 4.01 (1H, ddd, *J* = 11.3, 9.1, 5.5 Hz, NC$H_a\underline{H_b}$), 3.28 (3H, s, CHOC$\underline{H_3}$),
^{13}C{^{1}H} NMR ($CDCl_3$, 100.6 MHz) δ 184.8 (C), 169.4 (C), 143.9 (C), 143.5 (C), 129.5 (C), 120.4 (CH), 117.3 (CH), 116.3 (CH), 83.2 (CH), 66.7 (CH_2), 64.5 (CH), 64.3 (CH_2), 64.3 (CH_2), 57.2 (CH_3), 47.3 (CH_2).
HRMS (+ESI): *m/z* calcd. for $[M + Na]^+$ $C_{15}H_{16}N_4NaO_5S$: 387.0734; found: 387.0735.

***N*-[(2*S*,3*S*)-2-Azido-3-benzyloxy-3-(2,3-dihydrobenzo[*b*][1,4]dioxin-6-yl)propanoyl]-1,3-oxazolidine-2-thione (*ent*-6i)**

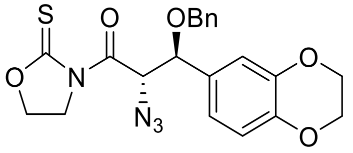

The General Procedure was followed with *N*-(2-azidoacetyl)-1,3-oxazolidine-2-thione (**4**, 93 mg, 0.50 mmol), 6-(bis(benzyloxy)methyl)-2,3-dihydrobenzo[*b*][1,4]dioxine (**i**, 200 mg, 0.55 mmol), [(*R*)-DTBM-SEGPHOS]$NiCl_2$ (32.6 mg, 25 µmol, 5 mol%), TMSOTf (120 µL, 0.65 mmol) and 2,6-lutidine (90 µL, 0.75 mmol), and the reaction mixture was stirred at –20 °C for 5 h.

The residue (dr 70:30) was purified by column chromatography (80:20 Hexanes/EtOAc) to afford 132 mg (0.30 mmol, 60% yield) of *anti* adduct ***ent*-6i** and 58 mg (0.13 mmol, 26% yield) of the *syn* diastereomer.

Yellowish oil.

R_f 0.20 (80:20 Hexanes/EtOAc).

Chiral HPLC (Phenomenex Lux® Cellulose-1 column, 25% *i*-PrOH in hexane, flow rate 1.0 mL·min^{-1}): Rt 36.7 min (Major 2*S*,3*S*-isomer) [Rt 24.9 min (minor 2*R*,3*R*-isomer)], 99% *ee*.

$[\alpha]_D^{20}$ +41.4 (*c* 1.00, $CHCl_3$).

IR (ATR) ν 2926, 2108, 1702, 1506, 1368, 1288, 1206, 1066, 889 cm^{-1}.

^{1}H NMR ($CDCl_3$, 400 MHz) δ 7.33–7.25 (3H, m, ArH), 7.22–7.20 (2H, m, ArH), 7.02 (1H, d, *J* = 2.0 Hz, ArH), 6.97 (1H, dd, *J* = 8.2, 2.0 Hz, ArH), 6.92 (1H, d, *J* = 8.2 Hz, ArH), 6.54 (1H, d, *J* = 8.9 Hz, C<u>H</u>N_3), 4.67 (1H, d, *J* = 8.9 Hz, C<u>H</u>OCH_3), 4.52 (1H, td, *J* = 8.9, 5.7 Hz, $OC\underline{H_a}H_b$), 4.46 (1H, d, *J* = 11.4 Hz, $OC\underline{H_a}H_bPh$), 4.31–4.26 (1H, m, $OCH_a\underline{H_b}$), 4.29 (4H, s, $OC\underline{H_2}C\underline{H_2}O$), 4.24–4.18 (2H, m, $OCH_a\underline{H_b}Ph$ & $NC\underline{H_a}H_b$), 4.08 (1H, ddd, *J* = 11.0, 9.0, 5.7 Hz, $NCH_a\underline{H_b}$).

$^{13}C\{^1H\}$ NMR ($CDCl_3$, 100.6 MHz) δ 185.3 (C), 170.6 (C), 144.3 (C), 143.8 (C), 137.6 (C), 130.0 (C), 128.3 (CH), 127.7 (CH), 127.6 (CH), 121.0 (CH), 117.7 (CH), 116.7 (CH), 81.7 (CH), 70.8 (CH_2), 66.6 (CH_2), 64.3 (CH_2), 64.3 (CH_2), 62.3 (CH), 47.2 (CH_2).

HRMS (+ESI): *m/z* calcd. for $[M - OBn]^+$ $C_{14}H_{13}N_4O_4S$: 333.0652; found: 333.0657. *m/z* calcd. for $[M + Na]^+$ $C_{21}H_{20}N_4NaO_5S$: 463.1047; found: 463.1059.

***N*-[(2*S*,3*R*)-2-Azido-3-benzyloxy-3-(3,4-bis(benzyloxy)phenyl)propanoyl]-1,3-oxazolidine-2-thione (*ent*-8j)**

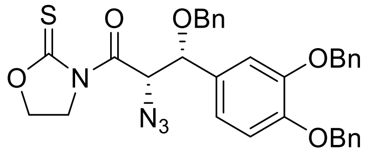

The General Procedure was followed with *N*-(2-azidoacetyl)-1,3-oxazolidine-2-thione (**4**, 47 mg, 0.25 mmol), 3,4-dibenzyloxybenzaldehyde dibenzyl acetal (**j**, 142 mg, 0.28 mmol), [(*R*)-DTBM-SEGPHOS]$NiCl_2$ (16.4 mg, 12.5 µmol, 5 mol%), TMSOTf (115 µL, 0.63 mmol, 2.5 equiv) and 2,6-lutidine (45 µL, 0.38 mmol), and the reaction mixture was stirred at –20 °C for 16 h.

The residue (dr 14:86) was purified by column chromatography (80:20 Hexanes/EtOAc) to afford 94 mg (0.16 mmol, 63% yield) of *syn* adduct ***ent*-8j** and 20 mg (33 µmol, 13% yield) of the *anti* diastereomer.

Pale orange oil.

R_f 0.25 (80:20 Hexanes/EtOAc).

Chiral HPLC (Phenomenex Lux® Cellulose-1 column, 40% *i*-PrOH in hexane, flow rate 1.0 mL·min^{-1}): Rt 25.4 min (Major 2*S*,3*R*-isomer) [Rt 43.5 min (minor 2*R*,3*S*-isomer)], 98% *ee*.

$[\alpha]_D^{20}$ –132.6 (*c* 1.0, $CHCl_3$).

IR (ATR) ν 2922, 2109, 1702, 1508, 1375, 1327, 1210, 1187, 1133, 1016, 736, 695 cm^{-1}.

^{1}H NMR ($CDCl_3$, 400 MHz) δ 7.49–7.45 (4H, m, ArH), 7.41–7.27 (9H, m, ArH), 7.25–7.21 (2H, m, ArH), 7.11 (1H, d, *J* = 1.9 Hz, ArH), 6.99 (1H, dd, *J* = 8.3, 1.9 Hz, ArH), 6.95 (1H, d, *J* = 8.3 Hz, ArH), 6.20 (1H, d, *J* = 5.1 Hz, CHN_3), 5.23–5.17 (4H, m, OCH_2Ph), 4.98 (1H, d, *J* = 5.1 Hz, CHOBn), 4.51 (1H, d, *J* = 12.2 Hz, OCH_aH_bPh), 4.37 (1H, ddd, *J* = 9.3, 8.2, 4.9 Hz, OCH_aH_b), 4.12 (1H, d, *J* = 12.2 Hz, OCH_aH_bPh), 3.97 (2H, m, OCH_aH_b & NCH_aH_b), 3.60 (1H, ddd, *J* = 10.2, 8.4, 4.9 Hz, NCH_aH_b).

^{13}C{^{1}H} NMR ($CDCl_3$, 100.6 MHz) δ 184.7 (C), 169.2 (C), 149.2 (C), 148.7 (C), 137.3 (C), 137.1 (C), 137.0 (C), 129.4 (C), 128.5 (CH), 128.5 (CH), 128.4 (CH), 128.3 (CH), 127.9 (CH), 127.9 (CH), 127.8 (CH), 127.3 (CH), 127.3 (CH), 120.5 (CH), 114.4 (CH), 113.7 (CH), 79.7 (CH), 71.1 (CH_2), 71.0 (CH_2), 70.4 (CH_2), 66.6 (CH_2), 65.1 (CH), 47.2 (CH_2).

HRMS (+ESI): *m/z* calcd. for $[M - OBn]^+$ $C_{26}H_{23}N_4O_4S$: 487.1435; found: 487.1443. *m/z* calcd. for $[M + Na]^+$ $C_{33}H_{30}N_4NaO_5S$: 617.1829; found: 617.1845.

***N*-[(2*S*,3*R*)-2-Azido-3-methoxy-3-(4-methylphenyl)propanoyl]-1,3-oxazolidine-2-thione (*ent*-8k)**

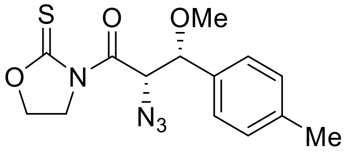

The General Procedure was followed with *N*-(2-azidoacetyl)-1,3-oxazolidine-2-thione (**4**, 47 mg, 0.25 mmol), 4-methylbenzaldehyde dimethyl acetal (**k**, 46 mg, 0.275 mmol), [(*R*)-DTBM-SEGPHOS]$NiCl_2$ (16.4 mg, 12.5 μmol, 5 mol%), TMSOTf (60 μL, 0.325 mmol), and 2,6-lutidine (45 μL, 0.375 mmol) at 0 °C for 16 h.

The residue (dr 30:70) was purified by column chromatography (from 40:60 to 0:100 Hexanes/CH_2Cl_2) to afford 50 mg (0.16 mmol, 62% yield) of adduct ***ent*-8k** and 23 mg (72 mmol, 29% yield) of the *anti* diastereomer.

White solid.

Mp 119–122 °C.

R_f 0.35 (CH_2Cl_2).

Chiral HPLC (Phenomenex Lux® Cellulose-5 column, 10% *i*-PrOH in hexane, flow rate 1.0 mL·min^{-1}): Rt 16.8 min (Major 2*S*,3*R*-isomer) [Rt 22.2 min (minor 2*R*,3*S*-isomer)], 98% *ee*.

$[\alpha]_D^{20}$ –61.1 (*c* 1.0, $CHCl_3$).

IR (ATR) ν 2920, 2093, 1705, 1511, 1359, 1169, 1018 cm^{-1}.

^{1}H NMR ($CDCl_3$, 400 MHz) δ 7.33–7.31 (2H, m, ArH), 7.19–7.18 (2H, m, ArH), 6.33 (1H, d, *J* = 6.1 Hz, CHN$_3$), 4.82 (1H, d, *J* = 6.1 Hz, CHOCH$_3$), 4.52 (1H, td, *J* = 8.3, 7.6, 5.3 Hz, OCH_aH$_b$), 4.26–4.12 (2H, m, OCH$_a$$H_b$ & NCH_aH$_b$), 3.92 (1H, ddd, *J* = 10.9, 8.1, 5.3 Hz, NCH$_a$$H_b$), 3.28 (3H, s, CHOC$H_3$), 2.35 (3H, s, ArC$H_3$).

^{13}C{^{1}H} NMR ($CDCl_3$, 100.6 MHz) δ 184.8 (C), 169.4 (C), 138.6 (C), 133.3 (C), 129.2 (CH), 127.4 (CH), 83.8 (CH), 66.7 (CH_2), 64.5 (CH), 57.2 (CH_3), 47.3 (CH_2), 21.2 (CH_3).

HRMS (+ESI): *m/z* calcd. for [M – OMe]$^+$ $C_{13}H_{13}N_4O_2S$: 289.0754; found: 289.0750. *m/z* calcd. for [M + Na]$^+$ $C_{14}H_{16}N_4NaO_3S$: 343.0835; found: 343.0832.

***N*-[(2*S*,3*R*)-2-Azido-3-methoxy-3-(naphthalen-2-yl)propanoyl]-1,3-oxazolidine-2-thione (*ent*-8l)**

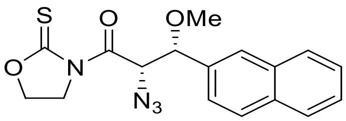

The General Procedure was followed with *N*-(2-azidoacetyl)-1,3-oxazolidine-2-thione (**4**, 93 mg, 0.50 mmol), 2-naphthaldehyde dimethyl acetal (**l**, 110 mg, 0.55 mmol), [(*R*)-DTBM-SEGPHOS]$NiCl_2$ (33.5 mg, 25 μmol, 5 mol%), TMSOTf (120 μL, 0.65 mmol) and 2,6-lutidine (90 μL, 0.75 mmol), and the reaction mixture was stirred at 0 °C for 16 h.

The residue (dr 32:68) was purified by column chromatography (80:20 Hexanes/EtOAc) to give 107 mg (0.30 mmol, 60% yield) of *syn* adduct ***ent*-8l** and 43 mg (0.12 mmol, 24% yield) of the *anti* diastereomer.

White solid.
Mp 132–135 °C.
R_f 0.15 (80:20 Hexanes/EtOAc).
Chiral HPLC (Phenomenex Lux® Cellulose-1 column, 15% *i*-PrOH in hexane, flow rate 1.0 mL·min^{-1}): Rt 28.1 min (Major 2*S*,3*R*-isomer) [Rt 41.6 min (minor 2*R*,3*S*-isomer)], 97% *ee*.
$[\alpha]_D^{20}$ –167.3 (*c* 1.00, $CHCl_3$).
IR (ATR) ν 2924, 2110, 1705, 1375, 1327, 1210, 1167, 1100, 1019, 949, 822 cm^{-1}.
^{1}H NMR ($CDCl_3$, 400 MHz) δ 7.90–7.83 (4H, m, Ar$\underline{H}$), 7.59 (1H, dd, *J* = 8.6, 1.6 Hz, Ar$\underline{H}$), 7.54–7.49 (2H, m, Ar$\underline{H}$), 6.50 (1H, d, *J* = 6.4 Hz, C$\underline{H}N_3$), 5.00 (1H, d, *J* = 6.4 Hz, C$\underline{H}OCH_3$), 4.41 (1H, td, *J* = 9.2, 5.2 Hz, OC$\underline{H}_aH_b$), 4.11 (1H, dt, *J* = 10.9, 9.4 Hz, NC$\underline{H}_aH_b$), 3.97–3.91 (1H, m, OC$H_a\underline{H}_b$), 3.80 (1H, ddd, *J* = 10.9, 8.9, 5.2 Hz, NC$H_a\underline{H}_b$), 3.35 (3H, s, CHOC$\underline{H}_3$).
$^{13}C\{^1H\}$ NMR ($CDCl_3$, 100.6 MHz) δ 184.8 (C), 169.3 (C), 133.9 (C), 133.5 (C), 132.9 (C), 128.5 (CH), 128.0 (CH), 127.7 (CH), 127.1 (CH), 126.6 (CH), 126.5 (CH), 124.6 (CH), 84.2 (CH), 66.6 (CH_2), 64.4 (CH), 57.4 (CH_3), 47.2 (CH_2).
HRMS (+ESI): *m/z* calcd. for $[M – OMe]^+$ $C_{16}H_{13}N_4O_2S$: 325.0754; found: 325.0760. *m/z* calcd. for $[M + Na]^+$ $C_{17}H_{16}N_4NaO_3S$: 379.0835; found: 379.0847.

***N*-[(2*S*,3*R*)-2-Azido-3-methoxy-3-phenylpropanoyl]-1,3-oxazolidine-2-thione (*ent*-8m)**

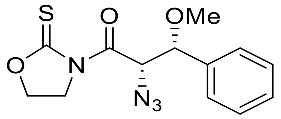

The General Procedure was followed with *N*-(2-azidoacetyl)-1,3-oxazolidine-2-thione (**4**, 93 mg, 0.50 mmol), benzaldehyde dimethyl acetal (**m**, 84 µL, 0.55 mmol), [(*R*)-DTBM-SEGPHOS]$NiCl_2$ (32.7 mg, 25 µmol, 5 mol%), TMSOTf (120 µL, 0.65 mmol) and 2,6-lutidine (90 µL, 0.75 mmol), and the reaction mixture was stirred at 0 °C for 5 h.

The residue (dr 43:57) was purified by column chromatography (98:2 toluene/THF) to give 74 mg (0.24 mmol, 48% yield) of *syn* adduct ***ent*-8m** and 34 mg (0.11 mmol, 22% yield) of the *anti* diastereomer ***ent*-6m**.

Brown oil.

R_f 0.15 (98:2 Toluene/EtOAc).

Chiral HPLC (Phenomenex Lux® Cellulose-1 column, 10% *i*-PrOH in hexane, flow rate 1.0 mL·min^{-1}): Rt 36.1 min (Major 2*S*,3*R*-isomer) [Rt 64.3 min (minor 2*R*,3*S*-isomer)], 95% *ee*.

$[\alpha]_D^{20}$ –110.0 (*c* 1.00, $CHCl_3$).

IR (ATR) ν 2930, 2878, 2105, 1701, 1471, 1367, 1254, 1100 cm^{-1}.

^{1}H NMR ($CDCl_3$, 400 MHz) δ 7.45–7.43 (2H, m, ArH), 7.40–7.35 (3H, m, ArH), 6.36 (1H, d, *J* = 6.1 Hz, C<u>H</u>N_3), 4.85 (1H, d, *J* = 6.1 Hz, C<u>H</u>OCH_3), 4.54–4.49 (1H, m, OC$\underline{H_a}H_b$), 4.23 (1H, dt, *J* = 9.5, 8.6 Hz, OC$H_a\underline{H_b}$), 4.19–4.13 (1H, m, NC$\underline{H_a}H_b$), 3.91 (1H, ddd, *J* = 11.0, 8.6, 5.3 Hz, NC$H_a\underline{H_b}$), 3.30 (3H, s, CHOC$\underline{H_3}$).

$^{13}C\{^1H\}$ NMR ($CDCl_3$, 100.6 MHz) δ 184.8 (C), 169.4 (C), 136.4 (C), 128.8 (CH), 128.6 (CH), 127.4 (CH), 84.0 (CH), 66.7 (CH_2), 64.5 (CH), 57.3 (CH_3), 47.2 (CH_2).

HRMS (+ESI): *m/z* calcd. for $[M – OMe]^+$ $C_{12}H_{11}N_4O_2S$: 275.0597; found: 275.0591. *m/z* calcd. for $[M – N_2 + H]^+$ $C_{13}H_{15}N_2O_3S$: 279.0798; found: 279.0792.

***N*-[(2*S*,3*S*)-2-Azido-3-methoxy-3-phenylpropanoyl]-1,3-oxazolidine-2-thione (*ent*-6m)**

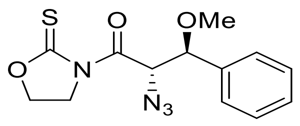

Brown oil.

R$_f$ 0.20 (98:2 Toluene/EtOAc).

Chiral HPLC (Phenomenex Lux® Cellulose-1 column, 10% *i*-PrOH in hexane, flow rate 1.0 mL·min^{-1}): Rt 24.4 min (Major 2*S*,3*S*-isomer) [Rt 34.0 min (minor 2*R*,3*R*-isomer)], 98% *ee*.

^{1}H NMR ($CDCl_3$, 400 MHz) δ 7.46–7.37 (5H, m, ArH), 6.50 (1H, d, *J* = 8.9 Hz, C<u>H</u>N_3), 4.60 (1H, d, *J* = 8.9 Hz, C<u>H</u>OCH_3), 4.63–4.57 (1H, m, OCH_aH_b), 4.53 (1H, td, *J* = 9.2, 8.5 Hz, OCH_aH_b), 4.32–4.21 (2H, m, NCH_2), 3.20 (3H, s, $CHOCH_3$).

^{13}C{^{1}H} NMR ($CDCl_3$, 100.6 MHz) δ 185.2 (C), 170.0 (C), 136.7 (C), 129.0 (CH), 128.7 (CH), 127.8 (CH), 83.5 (CH), 66.6 (CH_2), 61.9 (CH), 56.9 (CH_3), 47.1 (CH_2).

***N*-[(2*S*,3*R*)-2-Azido-3-methoxy-3-(2-thiophenyl)propanoyl]-1,3-oxazolidine-2-thione (*ent*-6n)**

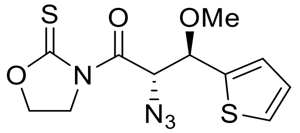

The General Procedure was followed with *N*-(2-azidoacetyl)-1,3-oxazolidine-2-thione (**4**, 47 mg, 0.25 mmol), 2-thiophenecarbaldehyde dimethyl acetal (**n**, 45 mg, 0.275 mmol), [(*R*)-DTBM-SEGPHOS]$NiCl_2$ (16.6 mg, 12.5 μmol, 5 mol%), TMSOTf (115 μL, 0.625 mmol, 2.5 equiv) and 2,6-lutidine (45 μL, 0.375 mmol), and the reaction mixture was stirred at 0 °C for 3 h.

The residue (dr 56:44) was purified by column chromatography (80:20 Hexanes/EtOAc) to afford 35 mg (0.11 mmol, 45% yield) of *anti* adduct ***ent*-6n** and 28 mg (0.09 mmol, 36% yield) of the *syn* diastereomer.

Pale orange/white oil.

R_f 0.20 (80:20 Hexanes/EtOAc).

Chiral HPLC (Phenomenex Lux® Cellulose-1 column, 15% *i*-PrOH in hexane, flow rate 1.0 mL·min^{-1}): Rt 20.9 min (Major 2*S*,3*R*-isomer) [Rt 24.9 min (minor 2*R*,3*S*-isomer)], 99% *ee*.

$[\alpha]_D^{20}$ +31.8 (*c* 1.0, $CHCl_3$).

IR (ATR) ν 2924, 2853, 2110, 1702, 1366, 1325, 1176, 1100, 1019, 949, 710 cm^{-1}.

^{1}H NMR ($CDCl_3$, 400 MHz) δ 7.41 (1H, ddd, *J* = 5.1, 1.3, 0.6 Hz, Ar<u>H</u>), 7.18 (1H, ddd, *J* = 3.5, 1.3, 0.6 Hz, Ar<u>H</u>), 7.06 (1H, dd, *J* = 5.1, 3.5 Hz, Ar<u>H</u>), 6.54 (1H, d, *J* = 8.6 Hz, C<u>H</u>N_3), 4.91 (1H, d, *J* = 8.6 Hz, C<u>H</u>OCH_3), 4.65–4.52 (2H, m, OC<u>H</u>$_2$), 4.32–4.20 (2H, m, NC<u>H</u>$_2$), 3.27 (3H, s, CHOC<u>H</u>$_3$).

^{13}C{^{1}H} NMR ($CDCl_3$, 100.6 MHz) δ 185.1 (C), 169.3 (C), 140.2 (C), 127.9 (CH), 127.1 (CH), 79.1 (CH), 66.7 (CH_2), 62.7 (CH), 57.1 (CH_3), 47.2 (CH_2).

HRMS (+ESI): *m/z* calcd. for [M – OMe – N_2]$^+$ $C_{10}H_9N_2O_2S_2$: 253.0100; found: 253.0098. *m/z* calcd. for [M + Na]$^+$ $C_{11}H_{12}N_4NaO_3S_2$: 335.0243; found: 335.0247.

***N*-[(2*S*,3*R*)-2-Azido-3-methoxy-3-(1-(phenylsulfonyl)-1*H*-indol-3-yl)propanoyl]-1,3-oxazolidine-2-thione (*ent*-8o)**

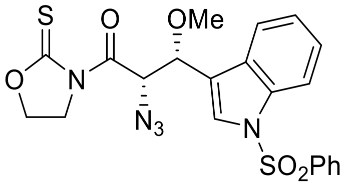

The General Procedure was followed with *N*-(2-azidoacetyl)-1,3-oxazolidine-2-thione (**4**, 47 mg, 0.25 mmol), 1-phenylsulfonyl-1*H*-indole-3-carbaldehyde dimethyl acetal (**o**, 91 mg, 0.275 mmol), [(*R*)-DTBM-SEGPHOS]$NiCl_2$ (25 mg, 19 µmol, 7.5 mol%), TMSOTf (68 µL, 0.375 mmol, 1.5 equiv) and 2,6-lutidine (45 µL, 0.375 mmol), and the reaction mixture was stirred at 0 °C for 16 h.

The residue (dr 4:96) was purified by column chromatography (80:20 Hexanes/EtOAc) to afford 109 mg (0.23 mmol, 90% yield) of *syn* adduct ***ent*-8o** as the unique diastereomer.

Thick salmon oil.

R_f 0.15 (80:20 Hexanes/EtOAc).

Chiral HPLC (Phenomenex Lux® Cellulose-5 column, 10% *i*-PrOH in hexane, flow rate 1.0 mL·min^{-1}): Rt 49.6 min (Major 2*S*,3*R*-isomer) [Rt 61.3 min (minor 2*R*,3*S*-isomer)], 99% *ee*.

$[\alpha]_D^{20}$ –39.6 (*c* 1.0, $CHCl_3$).

IR (ATR) ν 2926, 2110, 1702, 1446, 1364, 1172, 1090 cm^{-1}.

^{1}H NMR ($CDCl_3$, 400 MHz) δ 7.99 (1H, dt, *J* = 8.3, 0.9 Hz, ArH), 7.93–7.90 (2H, m, ArH), 7.74 (1H, ddd, *J* = 7.9, 1.3, 0.7 Hz, ArH), 7.67 (1H, s, ArH), 7.58–7.54 (1H, m, ArH), 7.49–7.44 (2H, m, ArH), 7.35 (1H, ddd, *J* = 8.5, 7.2, 1.3 Hz, ArH), 7.29–7.25 (1H, m, ArH), 6.52 (1H, d, *J* = 6.8 Hz, C<u>H</u>N_3), 5.09 (1H, d, *J* = 6.8 Hz, C<u>H</u>OCH_3), 4.41 (1H, td, *J* = 9.0, 8.5 Hz, OC$\underline{H}_a H_b$), 4.13–3.96 (2H, m, NC$\underline{H}_a H_b$ & OC$H_a \underline{H}_b$), 3.71–3.65 (1H, m, NC$H_a \underline{H}_b$), 3.32 (3H, s, CHOC$\underline{H}_3$).

^{13}C{^{1}H} NMR ($CDCl_3$, 100.6 MHz) δ 184.8 (C), 168.8 (C), 137.9 (C), 135.0 (C), 134.0 (CH), 129.4 (CH), 128.7 (C), 126.9 (CH), 125.8 (CH), 125.3 (CH), 123.7 (CH), 120.7 (CH), 118.1 (C), 113.6 (CH), 77.8 (CH), 66.6 (CH_2), 63.5 (CH), 57.4 (CH_3), 47.1 (CH_2).

HRMS (+ESI): *m/z* calcd. for [M + Na]$^+$ $C_{21}H_{19}N_5NaO_5S_2$: 508.0720; found: 508.0710.

***N*-[(2*S*,3*R*)-3-Allyloxy-2-azido-3-(4-benzyloxyphenyl)propanoyl]-1,3-oxazolidine-2-thione (*ent*-8p)**

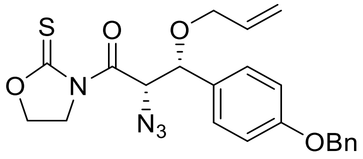

The General Procedure was followed with *N*-(2-azidoacetyl)-1,3-oxazolidine-2-thione (**4**, 47 mg, 0.25 mmol), 4-benzyloxybenzaldehyde diallyl acetal (**p**, 85 mg, 0.275 mmol), [(*R*)-DTBM-SEGPHOS]$NiCl_2$ (16.0 mg, 12.5 μmol, 5 mol%), TMSOTf (60 μL, 0.325 mmol) and 2,6-lutidine (45 μL, 0.375 mmol), and the reaction mixture was stirred at 0 °C for 16 h.

The residue (dr 49:51) was purified by column chromatography (40:60 Hexanes/Et_2O) to give 43 mg (0.10 mmol, 39% yield) of *syn* adduct ***ent*-8p** and 24 mg (55 μmol, 22% yield) of the *anti* diastereomer.

Thick salmon oil.

R_f 0.30 (40:60 Hexanes/Et_2O).

$[\alpha]_D^{20}$ –37.2 (*c* 0.5, $CHCl_3$).

IR (ATR) ν 2922, 2110, 1705, 1608, 1508, 1375, 1169, 1016 cm^{-1}.

^1H NMR ($CDCl_3$, 400 MHz) δ 7.45–7.32 (7H, m, Ar$\underline{H}$), 7.00–6.96 (2H, m, Ar$\underline{H}$), 6.33 (1H, d, *J* = 6.1 Hz, C$\underline{H}N_3$), 5.85 (1H, dddd, *J* = 17.3, 10.4, 6.2, 4.9 Hz, $OCH_2C\underline{H}=CH_2$), 5.26 (1H, dq, *J* = 17.3, 1.4 Hz, $OCH_2CH=C\underline{H}_aH_b$), 5.19 (1H, dq, *J* = 10.4, 1.4 Hz, $OCH_2CH=CH_a\underline{H}_b$), 5.07 (2H, s, $OC\underline{H}_2Ph$), 4.99 (1H, d, *J* = 6.1 Hz, $C\underline{H}OCH_2CH=CH_2$), 4.52–4.45 (1H, m, $OC\underline{H}_aH_b$), 4.23–4.11 (2H, m, $OCH_a\underline{H}_b$ & $NC\underline{H}_aH_b$), 4.03 (1H, ddt, *J* = 13.0, 4.9, 1.5 Hz, $OC\underline{H}_aH_bCH=CH_2$), 3.92–3.85 (1H, m, $NCH_a\underline{H}_b$), 3.77 (1H, ddt, *J* = 13.0, 6.2, 1.4 Hz, $OCH_a\underline{H}_bCH=CH_2$).

$^{13}C\{^1H\}$ NMR ($CDCl_3$, 100.6 MHz) δ 184.9 (C), 169.4 (C), 159.1 (C), 136.7 (C), 133.8 (CH), 128.8 (CH), 128.6 (CH), 128.1 (C), 127.5 (CH), 117.5 (CH_2), 114.8 (CH), 80.8 (CH), 70.0 (CH_2), 69.7 (CH_2), 66.7 (CH_2), 64.7 (CH), 47.3 (CH_2).

HRMS (+ESI): *m/z* calcd. for $[M + Na]^+$ $C_{22}H_{22}N_4NaO_4S$: 461.1254; found: 461.1239.

***N*-[(2*S*,3*R*)-3-Allyloxy-2-azido-3-(4-*tert*-butyldimethylsilyloxyphenyl)propanoyl]-1,3-oxazolidine-2-thione (*ent*-8q)**

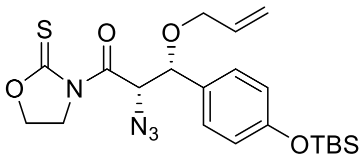

The General Procedure was followed with *N*-(2-azidoacetyl)-1,3-oxazolidine-2-thione (**4**, 47 mg, 0.25 mmol), 4-(*tert*-butyldimethylsilyloxy)benzaldehyde diallyl acetal (**q**, 92 mg, 0.275 mmol), [(*R*)-DTBM-SEGPHOS]$NiCl_2$ (16.1 mg, 12.5 μmol, 5 mol%), TMSOTf (60 μL, 0.325 mmol) and 2,6-lutidine (45 μL, 0.375 mmol), and the reaction mixture was stirred at 0 °C for 16 h.

The residue (dr 38:62) was purified by column chromatography (80:20 Hexanes/EtOAc) to give 76 mg (0.17 mmol, 66% yield) of a diastereomeric mixture of adduct ***ent*-8q** and the *anti* counterpart.

Thick salmon oil.

R_f 0.30 (80:20 Hexanes/EtOAc).

IR (ATR) ν 2928, 2108, 1702, 1606, 1508, 1364, 1252, 1165, 1075 cm^{-1}.

^{1}H NMR ($CDCl_3$, 400 MHz) δ 7.32–7.30 (2H, m, Ar$\underline{H}$), 6.84–6.80 (2H, m, Ar$\underline{H}$), 6.33 (1H, d, *J* = 6.2 Hz, C$\underline{H}N_3$), 5.85 (1H, dddd, *J* = 17.3, 10.5, 6.2, 4.9 Hz, $OCH_2C\underline{H}=CH_2$), 5.25 (1H, dq, *J* = 17.3, 1.3 Hz, $OCH_2CH=C\underline{H}_aH_b$), 5.18 (1H, dq, *J* = 10.4, 1.3 Hz, $OCH_2CH=CH_a\underline{H}_b$), 4.97 (1H, d, *J* = 6.2 Hz, $C\underline{H}OCH_2CH=CH_2$), 4.55–4.49 (1H, m, $OC\underline{H}_aH_b$), 4.32–4.13 (2H, m, $OCH_a\underline{H}_b$ & $NC\underline{H}_aH_b$), 4.03 (1H, dddd, *J* = 13.0, 4.8, 1.7, 1.3 Hz, $OC\underline{H}_aH_bCH=CH_2$), 3.93–3.87 (1H, m, $NCH_a\underline{H}_b$), 3.77 (1H, ddt, *J* = 13.1, 6.2, 1.4 Hz, $OCH_a\underline{H}_bCH=CH_2$), 0.98 (9H, s, $SiC(C\underline{H}_3)_3$), 0.20 (6H, s, $Si(C\underline{H}_3)_2$).

^{13}C{^{1}H} NMR ($CDCl_3$, 100.6 MHz) δ 184.8 (C), 169.4 (C), 156.1 (C), 133.8 (CH), 129.0 (C), 128.7 (CH), 120.0 (CH), 117.5 (CH_2), 80.9 (CH), 69.7 (CH_2), 66.6 (CH_2), 64.7 (CH), 47.3 (CH_2), 25.6 (CH_3), 18.2 (C), –4.4 (CH_3).

HRMS (+ESI): *m/z* calcd. for $[M – OCH_2CH=CH_2]^+$ $C_{18}H_{25}N_4O_3SSi$: 405.1400; found: 405.1411. *m/z* calcd. for $[M + Na]^+$ $C_{21}H_{30}N_4NaO_4SSi$: 485.1649; found: 485.1634.

***N*-[(2*S*,3*S*)-2-Azido-3-benzyloxy-3-(4-benzyloxyphenyl)propanoyl]-1,3-oxazolidine-2-thione (*ent*-6r)**

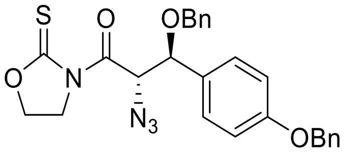

The General Procedure was followed with *N*-(2-azidoacetyl)-1,3-oxazolidine-2-thione (**4**, 47 mg, 0.25 mmol), 4-benzyloxybenzaldehyde dibenzyl acetal (**r**, 113 mg, 0.275 mmol), [(*R*)-DTBM-SEGPHOS]$NiCl_2$ (16.4 mg, 12.5 µmol, 5 mol%), TMSOTf (60 µL, 0.325 mmol) and 2,6-lutidine (45 µL, 0.3.75 mmol), and the reaction mixture was stirred at −20 °C for 16 h.

The residue (dr 58:42) was purified by column chromatography (from 80:20 to 70:30 Hexanes/EtOAc) to give 65 mg (0.13 mmol, 53% yield) of *anti* adduct ***ent*-6r** and 48 mg (98 µmol, 39% yield) of the *syn* diastereomer.

Thick white oil.

R_f 0.40 (70:30 Hexanes/EtOAc).

$[\alpha]_D^{20}$ +41.6 (*c* 1.00, $CHCl_3$).

IR (ATR) ν 2920, 2106, 1700, 1608, 1508, 1366, 1169, 1070 cm^{-1}.

^{1}H NMR ($CDCl_3$, 400 MHz) δ 7.46–7.26 (10H, m, Ar<u>H</u>), 7.22–7.20 (2H, m, Ar<u>H</u>), 7.06–7.04 (2H, m, Ar<u>H</u>), 6.58 (1H, d, *J* = 9.0 Hz, C<u>H</u>N_3), 5.10 (2H, s, OC$\underline{H}_2$Ph), 4.73 (1H, d, *J* = 9.0 Hz, C<u>H</u>OBn), 4.53 (1H, td, *J* = 8.7, 5.7 Hz, OC$\underline{H}_a H_b$), 4.44 (1H, d, *J* = 11.4 Hz, OC$\underline{H}_a H_b$Ph), 4.31–4.26 (1H, m, OC$H_a \underline{H}_b$), 4.25–4.19 (2H, m, N$\underline{H}_a H_b$ & OC$H_a \underline{H}_b$Ph), 4.09 (1H, ddd, *J* = 10.9, 8.7, 5.7 Hz, NC$H_a \underline{H}_b$).

^{13}C{^{1}H} NMR ($CDCl_3$, 100.6 MHz) δ 185.4 (C), 170.7 (C), 159.5 (C), 137.6 (C), 136.7 (C), 129.2 (CH), 129.1 (C), 128.6 (CH), 128.3 (CH), 128.0 (CH), 127.7 (CH), 127.6 (CH), 127.5 (CH), 115.2 (CH), 81.8 (CH), 70.8 (CH_2), 70.1 (CH_2), 66.6 (CH_2), 62.4 (CH), 47.2 (CH_2).

HRMS (+ESI): *m/z* calcd. for $[M - OBn]^+$ $C_{19}H_{17}N_4O_3S$: 381.1016; found: 381.1013. *m/z* calcd. for $[M + Na]^+$ $C_{26}H_{24}N_4NaO_4S$: 511.1410; found: 511.1410.

***N*-[(2*S*,3*R*)-2-Azido-3-benzyloxy-3-(4-*tert*-butyldimethylsilyloxyphenyl)propanoyl]-1,3-oxazolidine-2-thione (*ent*-8s)**

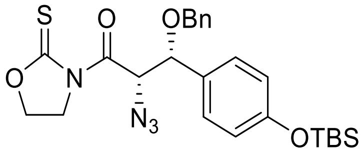

The General Procedure was followed with *N*-(2-azidoacetyl)-1,3-oxazolidine-2-thione (**4**, 47 mg, 0.25 mmol), 4-(*tert*-butyldimethylsilyloxy)benzaldehyde dibenzyl acetal (**s**, 120 mg, 0.275 mmol), [(*R*)-DTBM-SEGPHOS]$NiCl_2$ (32.2 mg, 24.6 μmol, 10 mol%), TMSOTf (77 μL, 0.425 mmol, 1.7 equiv) and 2,6-lutidine (45 μL, 0.375 mmol), and the reaction mixture was stirred at –20 °C for 16 h.

The residue (dr 45:55) was purified by column chromatography (80:20 Hexanes/EtOAc) to give 67 mg (0.13 mmol, 52% yield) of a diastereomeric mixture of adduct ***ent*-8s** and the *anti* counterpart.

Ocre oil.
$\mathbf{R_f}$ 0.30 (80:20 Hexanes/EtOAc).
IR (ATR) ν 2928, 2108, 1702, 1605, 1508, 1364, 1252, 1165, 1014 cm^{-1}.
^{1}H NMR ($CDCl_3$, 400 MHz) δ 7.40–7.27 (6H, m, Ar<u>H</u>), 7.21 (1H, d, *J* = 1.9 Hz, Ar<u>H</u>), 6.89–6.86 (2H, m, Ar<u>H</u>), 6.23 (1H, d, *J* = 5.1 Hz, C<u>H</u>N_3), 5.06 (1H, d, *J* = 5.1 Hz, C<u>H</u>OBn), 4.61 (1H, d, *J* = 12.2 Hz, OC<u>H</u>$_a$H$_b$Ph), 4.45–4.41 (1H, m, OC<u>H</u>$_a$H$_b$), 4.21 (1H, d, *J* = 12.2 Hz, OCH$_a$<u>H</u>$_b$Ph), 4.25–4.18 (1H, m, NC<u>H</u>$_a$H$_b$), 4.13–4.02 (2H, m, OCH$_a$<u>H</u>$_b$ & NCH$_a$<u>H</u>$_b$), 1.00 (9H, s, SiC(C<u>H</u>$_3$)$_3$), 0.23 (6H, s, Si(C<u>H</u>$_3$)$_2$).
^{13}C{^{1}H} NMR ($CDCl_3$, 100.6 MHz) δ 184.7 (C), 169.3 (C), 156.1 (C), 137.4 (C), 128.9 (C), 128.7 (CH), 128.3 (CH), 128.3 (CH), 127.9 (CH), 120.1 (CH), 79.8 (CH), 70.4 (CH_2), 66.6 (CH_2), 65.2 (CH), 47.2 (CH_2), 25.6 (CH_3), 18.2 (C), –4.4 (CH_3).
HRMS (+ESI): *m/z* calcd. for $[M + Na]^+$ $C_{25}H_{32}N_4NaO_4SSi$: 535.1806; found: 535.1801.

6. Structural Studies

(2*R*,3*R*)-2-Azido-3-methoxy-3-(4-methoxyphenyl)-*N*-[(*S*)-1-phenylethyl)]propanamide (9)

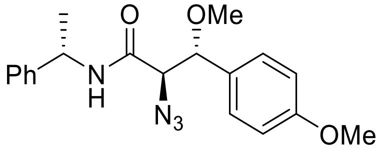

Neat (*S*)-1-phenylethylamine (240 µL, 1.86 mmol, 1.1 equiv) was added dropwise to a solution of **5a** (600 mg, 1.7 mmol, 1.0 equiv) in THF (8 mL) at 0 °C under N_2 atmosphere. After 10 min, the solution was allowed to warm to room temperature and stirred for 1 h.

The volatiles were removed *in vacuo* and the resulting crude mixture was purified by flash column chromatography (60:40 Hexanes/EtOAc) to afford amide **9** (554 mg, 1.56 mmol, 92% yield) as a white off solid and as a single enantiomer.

Amide **9** was recrystallized (Hexane/CH_2Cl_2) to produce colorless needles, which were submitted to X-ray analysis.

White off solid.
Mp 118–120 °C.
R_f 0.60 (60:40 Hexanes/EtOAc).
$[\alpha]_D^{20}$ –228.5 (*c* 1.0, $CHCl_3$).
IR (ATR) ν 3330, 2933, 2829, 2123, 1650, 1613, 1529, 1510, 1494, 1288, 1249, 1170, 1094, 1025 cm^{-1}.
^{1}H NMR (400 MHz, $CDCl_3$) δ 7.21–7.13 (5H, m, Ar<u>H</u>), 6.84–6.80 (2H, m, Ar<u>H</u>), 6.76–6.73 (2H, m, Ar<u>H</u>), 6.32 (1H, d, *J* = 8.3 Hz, N<u>H</u>), 5.00–4.93 (1H, m, $CH_3C\underline{H}NH$), 4.84 (1H, d, *J* = 3.7 Hz, $C\underline{H}OCH_3$), 4.54 (1H, d, *J* = 3.7 Hz, $C\underline{H}N_3$), 3.79 (3H, s, $ArOC\underline{H_3}$), 3.30 (3H, s, $CHOC\underline{H_3}$), 1.40 (3H, d, *J* = 7.0 Hz, $C\underline{H}_3CHNH$).
$^{13}C\{^1H\}$ NMR (100.6 MHz, $CDCl_3$,) δ 165.5 (C), 159.6 (C), 142.1 (C), 129.1 (CH), 128.3 (CH), 127.4 (C), 127.1 (CH), 125.9 (CH), 113.6 (CH), 83.7 (CH), 67.7 (CH), 56.8 (CH_3), 55.1 (CH_3), 48.1 (CH), 21.5 (CH_3).
HRMS (+ESI): *m/z* calcd. for $[M + H]^+$ $C_{19}H_{23}N_4O_3$: 355.1765; found: 355.1763.

(2*S*,3*R*)-2-Azido-3-methoxy-3-(4-methoxyphenyl)-*N*-[(*S*)-1-phenylethyl)]propanamide (10)

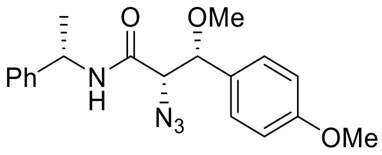

Neat (*S*)-1-phenylethylamine (195 µL, 1.5 mmol, 1.5 equiv) was added dropwise to a solution of ***ent*-8a** (85 mg, 0.25 mmol, 1.0 equiv) in CH_2Cl_2 (1.5 mL) at 0 °C under N_2 atmosphere. After 10 min, the solution was allowed to warm to room temperature and stirred for 2 h.

The volatiles were removed *in vacuo* and the resulting crude was purified by flash column chromatography (60:40 Hexanes/EtOAc) to afford amide **10** (70 mg, 0.20 mmol, 79% yield) as a white solid and 20 mg (0.19 mmol, 78%) of recovered 1,3-oxazolidine-2-thione.

Amide **10** was recrystallized (hexane/CH_2Cl_2) to produce colorless needles, which were submitted to X-ray analysis.

White solid.
Mp 93–95 °C.
R_f 0.80 (60:40 Hexanes/EtOAc).
$[\alpha]_D^{20}$ –53.6 (*c* 1.0, $CHCl_3$).
IR (ATR) ν 3286, 3083, 2930, 2106, 1649, 1511, 1249, 1101, 1034, 699 cm^{-1}.
^{1}H NMR (400 MHz, $CDCl_3$) δ 7.35–7.26 (5H, m, Ar$\underline{H}$), 7.25–7.22 (2H, m, Ar$\underline{H}$), 6.92–6.89 (2H, m, Ar$\underline{H}$), 6.51 (1H, d, *J* = 8.2 Hz, N$\underline{H}$), 5.14–5.07 (1H, m, $CH_3C\underline{H}NH$), 4.82 (1H, d, *J* = 3.8 Hz, $C\underline{H}OCH_3$), 3.94 (1H, d, *J* = 3.8 Hz, $C\underline{H}N_3$), 3.82 (3H, s, $ArOC\underline{H_3}$), 3.32 (3H, s, $CHOC\underline{H_3}$), 1.51 (3H, d, *J* = 6.9 Hz, $C\underline{H}_3CHNH$).
$^{13}C\{^1H\}$ NMR (100.6 MHz, $CDCl_3$,) δ 166.3 (C), 159.8 (C), 142.4 (C), 128.8 (C), 128.7 (CH), 128.1 (CH), 127.5 (CH), 126.2 (CH), 114.2 (CH), 82.5 (CH), 69.7 (CH), 57.6 (CH_3), 55.2 (CH_3), 49.0 (CH), 21.6 (CH_3).
HRMS (+ESI): *m/z* calcd. for $[M + Na]^+$ $C_{19}H_{22}N_4NaO_3$: 377.1584; found: 377.1574.

7. Transformations

Methyl *N*-[(2*S*,3*R*)-2-azido-3-methoxy-3-(4-methoxyphenyl)propanoyl]-*L*-alaninate (11)

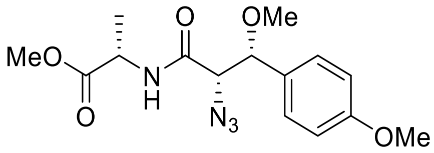

Neat Et_3N (42 µL, 0.3 mmol, 1.5 equiv) was added dropwise to a colorless solution of ***ent*-8a** (67 mg, 0.2 mmol, 1.0 equiv), methyl L-alaninate hydrochloride (42 mg, 0.30 mmol, 1.5 equiv) and DMAP (5 mg, 40 µmol, 20 mol%) in CH_2Cl_2 (5 mL) at 0 °C under N_2 atmosphere. The solution was stirred at 0 °C for 16 h.

The reaction mixture was diluted with CH_2Cl_2 (3 mL) and washed with H_2O (5 mL), dried (Na_2SO_4) and filtered. Finally, the volatiles were removed *in vacuo* and the resulting crude mixture was purified by flash column chromatography (70:30 Hexanes/EtOAc) to afford amide **11** (54 mg, 0.16 mmol, 80% yield) as a single diastereomer.

Colorless oil.
R_f 0.30 (70:30 Hexanes/EtOAc).
$[\alpha]_D^{20}$ –17.0 (*c* 1.0, $CHCl_3$).
IR (ATR) ν 3327, 2989, 2933, 2837, 2110, 1743, 1664, 1612, 1513, 1454, 1249, 1176, 1101, 1034 cm^{-1}.
^{1}H NMR (400 MHz, $CDCl_3$) δ 7.34–7.30 (2H, m, Ar<u>H</u>), 6.95–6.91 (3H, m, Ar<u>H</u> & N<u>H</u>), 4.80 (1H, d, *J* = 3.5 Hz, C<u>H</u>OCH$_3$), 4.57 (1H, q, *J* = 7.2 Hz, CH$_3$C<u>H</u>NH), 4.01 (1H, d, *J* = 3.5 Hz, C<u>H</u>N$_3$), 3.81 (3H, s, C<u>H</u>$_3$OOC), 3.75 (3H, s, ArOC<u>H$_3$</u>), 3.28 (3H, s, CHOC<u>H$_3$</u>), 1.42 (3H, d, *J* = 7.2 Hz, C<u>H</u>$_3$CHNH).
^{13}C{^{1}H} NMR (100.6 MHz, $CDCl_3$,) δ 172.8 (C), 166.8 (C), 159.8 (C), 128.7 (C), 128.1 (CH), 114.2 (CH), 82.6 (CH), 69.6 (CH), 57.6 (CH_3), 55.2 (CH_3), 52.5 (CH_3), 48.2 (CH), 18.2 (CH_3).
HRMS (+ESI): *m/z* calcd. for [M – $CH_3OOCCHCH_3$ + H]$^+$ $C_{11}H_{14}N_4O_3$: 250.1060; found: 250.1072. *m/z* calcd. for [M + Na]$^+$ $C_{15}H_{20}N_4NaO_5$: 359.1326; found: 359.1328.

Methyl *N*-[(2*S*,3*R*)-2-azido-3-methoxy-3-(4-methoxyphenyl)propanoyl]-*L*-leucinate (12)

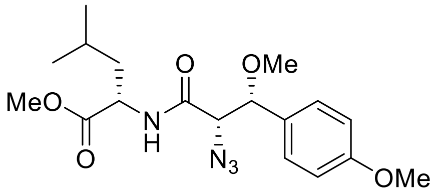

Neat Et_3N (42 µL, 0.3 mmol, 1.5 equiv) was added dropwise to a colorless solution of ***ent*-8a** (67 mg, 0.2 mmol, 1.0 equiv), methyl L-leucinate hydrochloride (55 mg, 0.30 mmol, 1.5 equiv) and DMAP (5 mg, 40 µmol, 20 mol%) in CH_2Cl_2 (5 mL) at 0 °C under N_2 atmosphere. The solution was stirred at 0 °C for 16 h.

The reaction mixture was diluted with CH_2Cl_2 (3 mL) and washed with H_2O (5 mL), dried (Na_2SO_4) and filtered. Finally, the volatiles were removed *in vacuo* and the resulting crude mixture was purified by flash column chromatography (70:30 Hexanes/EtOAc) to afford amide **12** (61 mg, 0.16 mmol, 81% yield) as a single diastereomer.

Colorless oil.
R$_f$ 0.55 (70:30 Hexanes/EtOAc).
$[\alpha]_D^{20}$ –21.7 (*c* 1.0, $CHCl_3$).
IR (ATR) ν 3308, 2956, 2930, 2872, 2110, 1743, 1661, 1612, 1513, 1441, 1251, 1176, 1103, 1034 cm^{-1}.
^{1}H NMR (400 MHz, $CDCl_3$) δ 7.35–7.31 (2H, m, Ar$\underline{H}$), 6.95–6.92 (2H, m, Ar$\underline{H}$), 6.77 (1H, d, *J* = 8.8 Hz, N$\underline{H}$), 4.81 (1H, d, *J* = 3.1 Hz, C$\underline{H}$OCH$_3$), 4.67 (1H, td, *J* = 8.8, 4.8 Hz, CH$_2$C$\underline{H}$NH), 4.02 (1H, d, *J* = 3.1 Hz, C$\underline{H}$N$_3$), 3.82 (3H, s, C$\underline{H_3}$OOC), 3.73 (3H, s, ArOC$\underline{H_3}$), 3.27 (3H, s, CHOC$\underline{H_3}$), 1.76–1.55 (3H, m, (CH$_3$)$_2$C$\underline{H}$C$\underline{H}_2$), 0.95 (3H, d, *J* = 6.2 Hz, C$\underline{H}_3$CH), 0.95 (3H, d, *J* = 6.3 Hz, C$\underline{H}_3$CH).
^{13}C{^{1}H} NMR (100.6 MHz, $CDCl_3$,) δ 172.9 (C), 167.0 (C), 159.8 (C), 128.7 (C), 128.1 (CH), 114.2 (CH), 82.5 (CH), 69.9 (CH), 57.5 (CH$_3$), 55.3 (CH$_3$), 52.3 (CH$_3$), 50.7 (CH), 41.6 (CH$_2$), 24.5 (CH), 22.9 (CH$_3$), 21.6 (CH$_3$).
HRMS (+ESI): *m/z* calcd. for [M – OMe – N$_2$]$^+$ $C_{17}H_{23}N_2O_4$: 319.1652; found: 319.1650. *m/z* calcd. for [M + Na]$^+$ $C_{18}H_{26}N_4NaO_5$: 401.1795; found: 401.1797.

8. X-Ray Analyses

8.1. X-Ray Analysis of Adduct 9

Adduct **9** was dissolved in a vial with the minimum amount of 1:1 CH_2Cl_2/hexanes. The vial was opened and crystals were allowed to grow at room temperature for several days. Biggest and clearest crystals were washed with hexanes, dried, and submitted to X-ray analysis described as follows.

A colorless needle-like specimen of $C_{19}H_{21}N_4O_3$, approximate dimensions 0.078 mm × 0.080 mm × 0.350 mm, was used for the X-ray crystallographic analysis. The X-ray intensity data were measured on a D8 Venture system equipped with a multilayer monochromator and a Mo microfocus (λ = 0.71073 Å).

The frames were integrated with the Bruker SAINT software package using a narrow-frame algorithm. The integration of the data using an orthorhombic unit cell yielded a total of 10312 reflections to a maximum θ angle of 27.47° (0.77 Å resolution), of which 4014 were independent (average redundancy 2.569, completeness = 98.9%, R_{int} = 2.74%, R_{sig} = 3.29%) and 3604 (89.79%) were greater than 2σ(F^2). The final cell constants of a = 5.40990(10) Å, b = 17.6136(4) Å, c = 18.7475(5) Å, volume = 1786.41(7) Å^3, are based upon the refinement of the XYZ-centroids of reflections above 20 σ(I). Data were corrected for absorption effects using the Multi-Scan method (SADABS). The calculated minimum and maximum transmission coefficients (based on crystal size) are 0.7027 and 0.7456.

The structure was solved and refined using the Bruker SHELXTL Software Package, using the space group P 21 21 21, with Z = 4 for the formula unit, $C_{19}H_{21}N_4O_3$. The final anisotropic full-matrix least-squares refinement on F^2 with 238 variables converged at R1 = 3.63%, for the observed data and wR2 = 9.64% for all data. The goodness-of-fit was 1.040. The largest peak in the final difference electron density synthesis was 0.475 e^-/Å^3 and the largest hole was -0.180 e^-/Å^3 with an RMS deviation of 0.043 e^-/Å^3. On the basis of the final model, the calculated density was 1.314 g/cm^3 and F(000), 748 e^-.

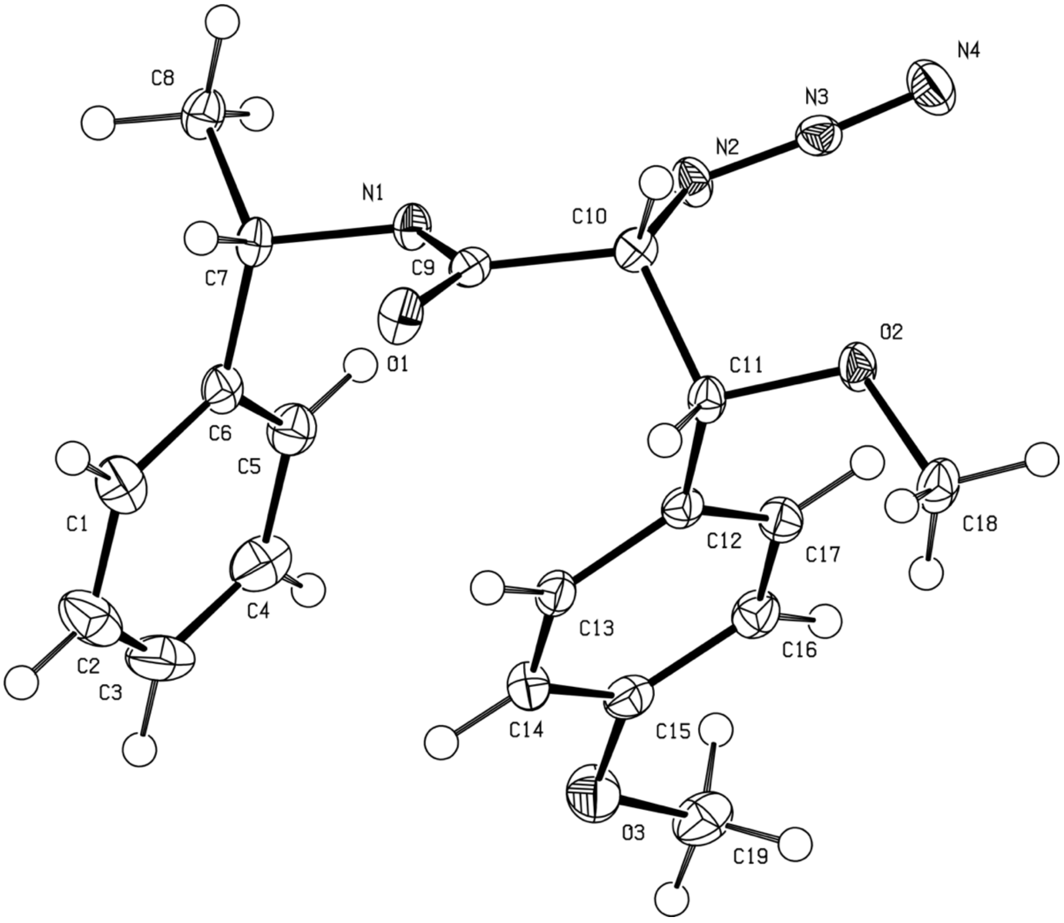

X-Ray structure of amide 9. The ellipsoid contour represents 50% probability.[3]

8.2. X-Ray Analysis of Adduct 10

Adduct **10** was dissolved in a vial with the minimum amount of 1:1 CH_2Cl_2/hexanes. The vial was opened and crystals were allowed to grow at room temperature for several days. Biggest and clearest crystals were washed with hexanes, dried, and submitted to X-ray analysis described as follows.

A colorless block-like specimen with approximate dimensions 0.496 mm x 0.326 mm x 0.280 mm, was used for the X-ray crystallographic analysis. The X-ray intensity data were measured at 304K on a D8 Venture system equipped with a multilayer monochromator and a Mo microfocus (λ = 0.71073 Å).

The frames were integrated with the Bruker SAINT software package using a narrow-frame algorithm. The integration of the data using an orthorhombic unit cell yielded a total of 160668 reflections to a maximum θ angle of 30.62° (0.70 Å resolution), of which 12112 were independent (average redundancy 13.265, completeness = 99.7%, R_{int} = 6.29%, R_{sig} = 3.03%) and 7734 (63.85%) were greater than $2\sigma(F^2)$. The final cell constants of a = 10.5586(4) Å, b = 18.2594(5) Å, c = 20.4930(7) Å, volume = 3950.9(2) $Å^3$, are based upon the refinement of the XYZ-centroids of reflections above 20 σ(I). Data were corrected for absorption effects using the multi-scan method (SADABS). The calculated minimum and maximum transmission coefficients (based on crystal size) are 0.5674 and 0.7461.

The structure was solved and refined using the Bruker SHELXTL Software Package, using the space group $P2_12_12_1$, with *Z* = 8 for the formula unit, $C_{19}H_{22}N_4O_3$. The final anisotropic full-matrix least-squares refinement on F^2 with 492 variables converged at R1 = 8.58%, for the observed data and wR2 = 26.86% for all data. The goodness-of-fit was 1.062. The largest peak in the final difference electron density synthesis was 0.271 $e^-/Å^3$ and the largest hole was -0.260 $e^-/Å^3$ with an RMS deviation of 0.042 $e^-/Å^3$. On the basis of the final model, the calculated density was 1.192 g/cm^3 and F(000), 1504 e^-.

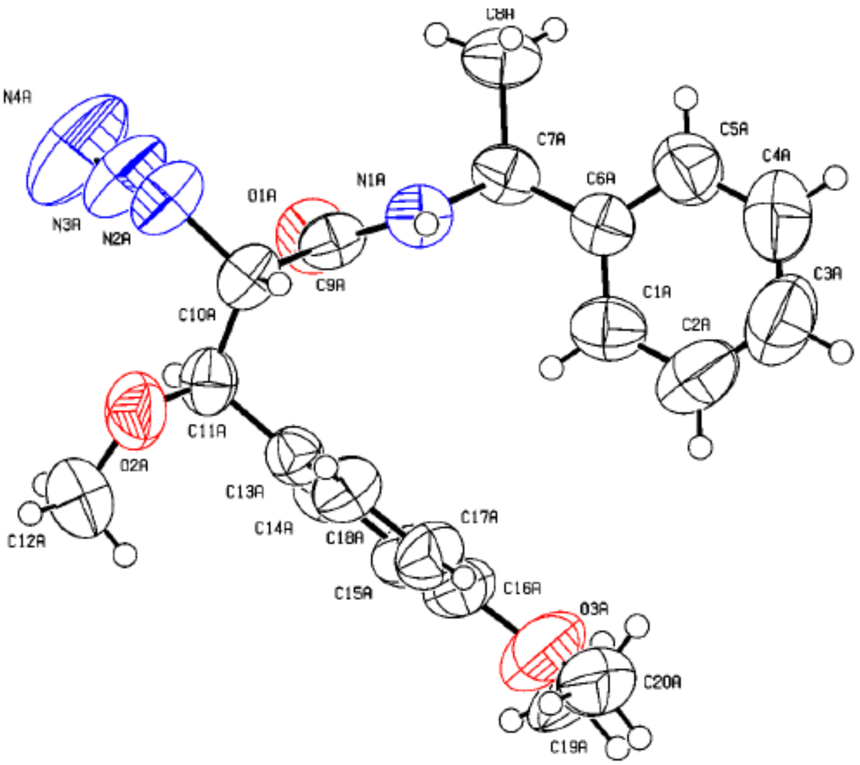

X-Ray structure of amide 10. The ellipsoid contour represents 50% probability.

9. Computational studies

9.1. Computational details

ONIOM calculations were carried out using the Gaussian09 package.[4] High quantum layer was defined by nickel, phosphorous and the *N*-azidoacetyl thioimide with the electrophile in the reaction pathway, while a low layer included the organic framework of both diphosphine ligands excluding phosphorous atoms by treatment of an universal field force.[5] The hybrid density functional known as B3LYP was applied.[6] The all-electron basis sets having triple-ξ quality with an extra polarization function were used for all elements (TZVP).[7] The geometries were fully optimized without restrictions and transition states were confirmed by vibrational analysis. Solvent effects were considered by PCM algorithm,[8] keeping the optimized geometry for the gas phase (single-point calculations).

The molecular structures were fully optimized without restrictions and well characterized by vibrational analysis, obtaining all real frequencies for the minima and the only imaginary one for the transition states. For the transition states, the obtained imaginary frequency was analyzed and confirmed with the help of molecular visualization programs (GaussView) to ensure that it corresponds to the formation of the indicated C–C bond. The values of the imaginary frequencies for all transition states are now provided in the supporting material.

Since all transition states are confirmed by vibrational analysis, no IRC calculation were carried out for the present systems. It should be noted that previous works with similar systems, the IRC calculation were performed and did not bring relevant information.

9.2. Structural analysis

Continuous shape measures were calculated with the SHAPE program,[9] that provides quantitative information of how much the environment is deviated from an ideal polyhedron.

9.3. Calculations

In order to understand the different stereochemical outcome of the reactions of [(diphosphine)Ni(α-azidoacetyl thioimides)]$^+$ with aromatic acetals, a computational study was carried out. All molecular geometries were calculated in singlet ground state.

Initially, we focused on the structure of [(Tol-BINAP}Ni(α-azidoacetyl-1,3-thiazolidine-2-thione)]$^+$, optimizing the four conformations shown in Fig. S1a. The two structures with identical boat conformation for the *S*,*O*-chelate, **R1** and **R2**, possess a more planar environment for the nickel atom ($S_{SQ\text{-}4} \approx 1.6$, with opposite angles close to the ideal planar geometry) and result the most stable with differences of only 0.4 kcal·mol^{-1} (Table S1). The other two conformations showed a distorted

geometry around the nickel atom ($S_{SQ-4} \approx 3.5$) and a higher energy by more than 3 kcal·mol^{-1}. Notice that the difference in each pair is the envelope conformation for the ethylene fragment of the thiazolidinethione ring, which has less influence than the *S*,*O*-chelate conformation in the relative stability of the reactants.

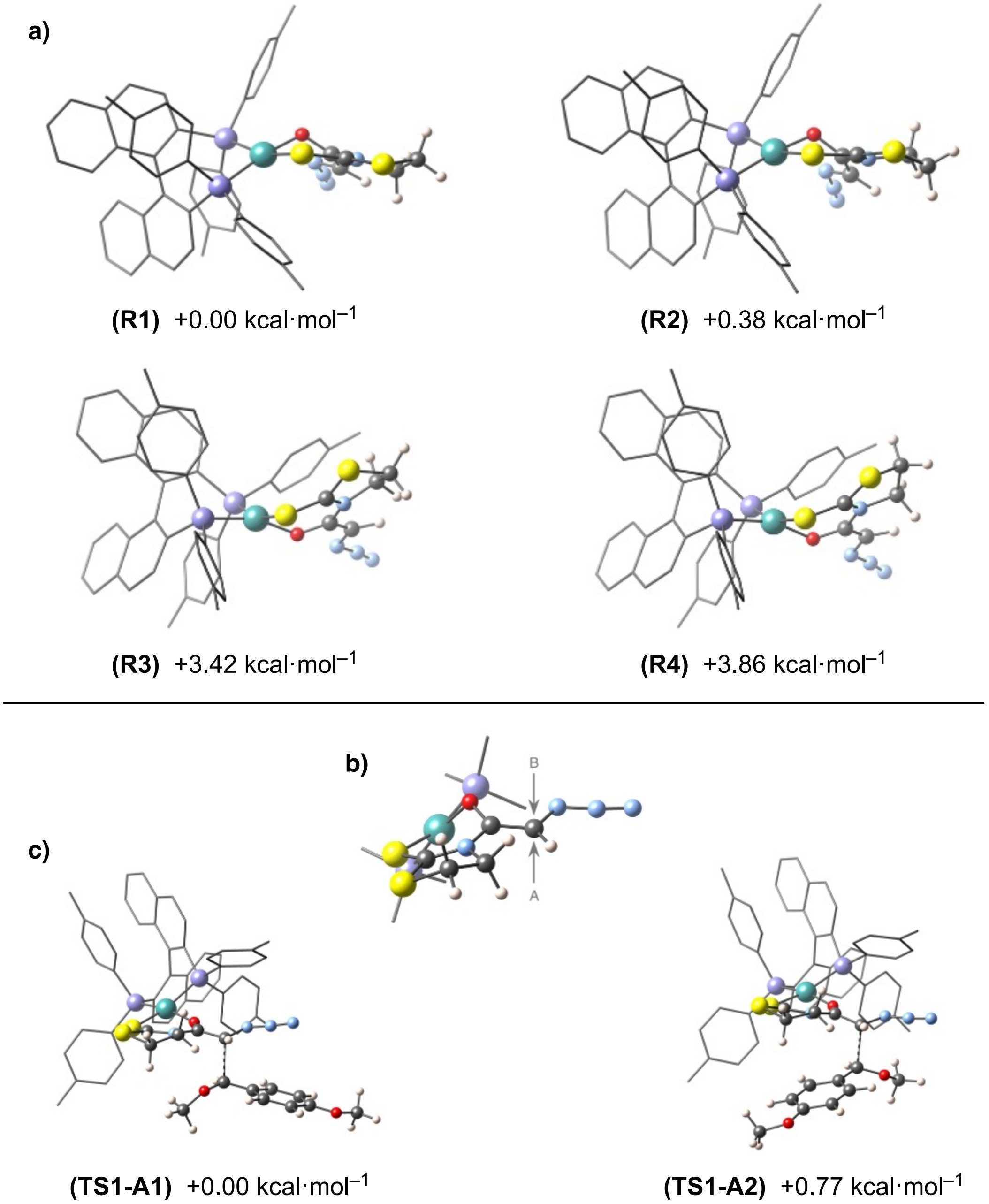

Fig. S1 (**a**) Optimized geometry of the four conformers of [(*S*)-Tol-BINAP}Ni(α-azidoacetyl-1,3-thiazolidine-2-thione)]$^{+}$ complex. (**b**) Detailed approximation of the electrophile to the enolate in **R1**. (**c**) The most favored transition states for each diastereomer.

The electrophile can approach the enolate from four different pathways depending on the reaction side (**A** or **B**, Fig. S1b) and the relative disposition of the substituents of the oxocarbenium intermediate,

which generates four related transition states (Fig. S1c). Taken the combinations of the previous four conformations, sixteen transition states were calculated, all of them with a range of 9 kcal·mol^{-1}. Since only the two former conformations for the reactant were clearly present in the solution, with a ratio of 68:32, an analysis including only the related transition states were considered. However, the small energetic difference between these two conformations could imply that the transition states generated still had similar energies. In the present case, the most stable **TS1-A1** and **TS2-A1** promoted the same diastereomer followed by **TS1-A2** (Fig. S1c), in agreement with the experimental data. The combination of these three structures results in an 86:14 (*anti*/*syn*) mixture, while other transition states contribute to very low amounts.

In a second stage, we applied the same analysis to [{(*R*)-DTBM-SEGPHOS}Ni(α-azidoacetyl-1,3-oxazolidine-2-thione)]$^{+}$ enolate (**Note:** the configuration is the opposite). The four geometries were reoptimized for the bulkiest DTBM-SEGPHOS ligand (Fig. S2a), resulting in an identical stability order. Such a preference for the conformations **R1** and **R2** were also related to a more planar environment for the nickel atom (S_{SQ-4} ≈ 2.8 and 3.0), which suggests that the relative stability is basically associated to the *S*,*O*-chelate and oxazolidine rings (Table S1). The difference of energy was only 0.4 kcal·mol^{-1}, while the other two conformers turned out to be higher in energy by more than 3 kcal·mol^{-1}. Nevertheless, the five-membered ring of the oxazolidinethione was practically planar due to the presence of the oxygen atom, in comparison to the previous thiazolidinethione. The deviation from planarity of the nickel atoms can be related to the steric hindrance of the bulky substituents of the diphosphine ligand.

However, a thorough analysis of the molecular geometry showed that the electrophile approach was only feasible on one side (**A** in Figure S2b), while the opposite should be rejected due to serious steric hindrance; therefore, the corresponding transitions states were not calculated. The other eight transition states were found in a range of 6 kcal·mol^{-1}, but with an important change respect to the previous system. To simplify the reasoning, we only considered the most favored transition state for each conformation. The most stable was now **TS2-A2**, while **TS1-A1** resulted higher in energy by 1.1 kcal·mol^{-1} (Figure S2c), resulting in a 90:10 mixture. Although the calculated energy was slightly overestimated in comparison to the experimental result, the modification of the preferred stereoisomer by changing the disphosphine is well reproduced (Table S3).

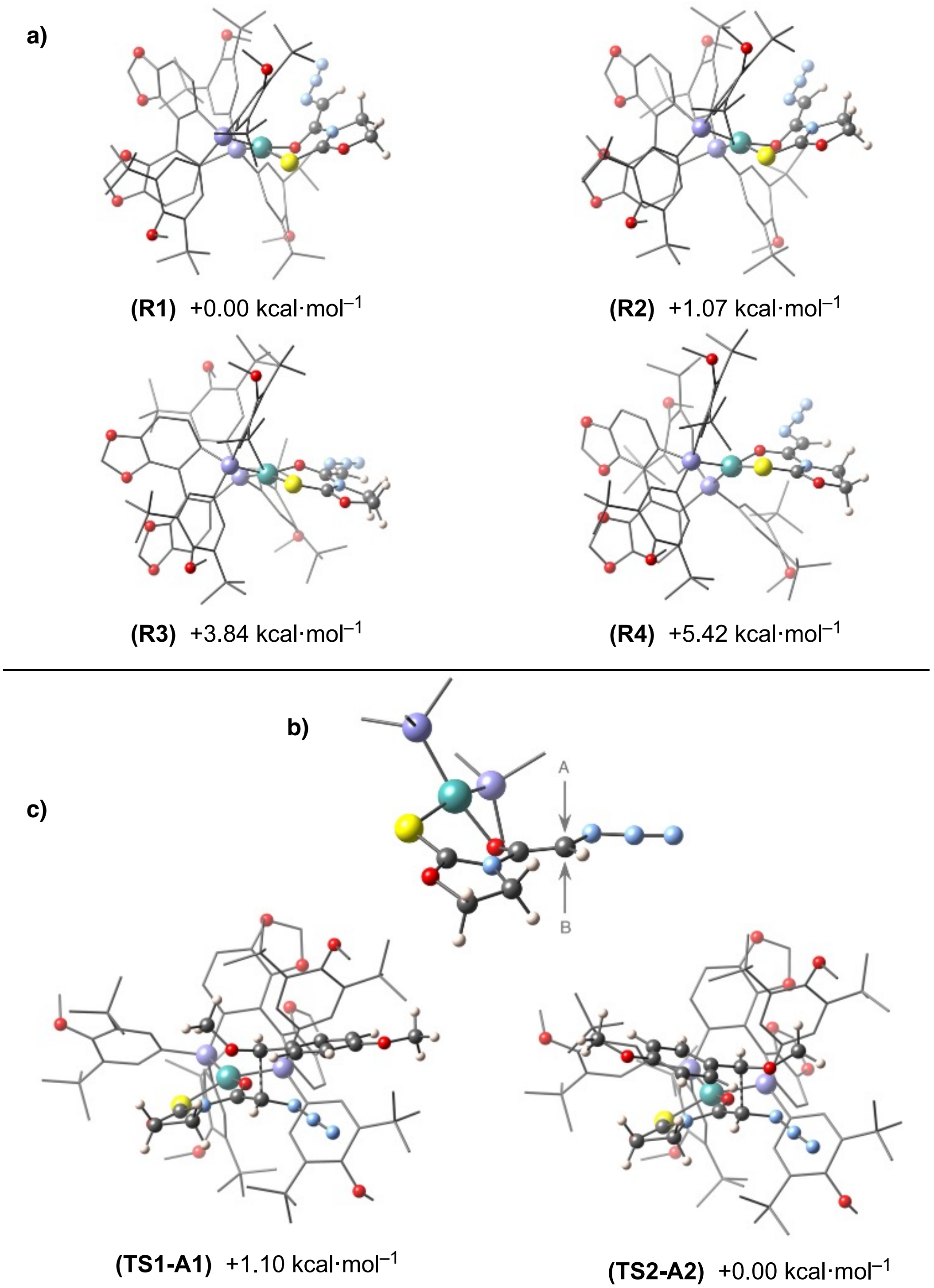

Fig. S2 (**a**) Optimized geometry of the four conformers of [{(*R*)-DTBM-SEGPHOS}Ni(α-azidoacetyl-1,3-oxazolidine-2-thione)]$^+$ complex. (**b**) Detailed approximation of the electrophile to the enolate in **R1**. (**c**) The most favored transition states for each diastereomer.

We completed the computational study of the complex [{(*R*)-DTBM-SEGPHOS}Ni(α-azidoacetyl-1,3-oxazolidine-2-thione)]$^{+}$ with the analysis of the influence of the substituents (R and PG) on the electrophile (Fig. S3 and Table S3). The substrate with PG: Bn showed the same behavior than that from PG: Me; furthermore **TS1-A2** were more stable than **TS1-A1** by 2.5 kcal·mol^{-1} to yield the *syn* diastereomer exclusively. Nevertheless, the transition state **TS2-A1** from the substrate with R: Bn became the most stable over **TS2-A2** by just 0.2 kcal·mol^{-1} to give a 59:41 *anti*/*syn* mixture. As expected, the substrate with R, PG: Bn mainly evolved through **TS1-A1** to provide a 70:30 *anti*/*syn* mixture.

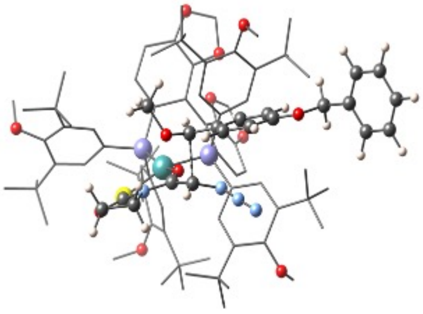

(TS1-A1) +2.51 kcal·mol^{-1}

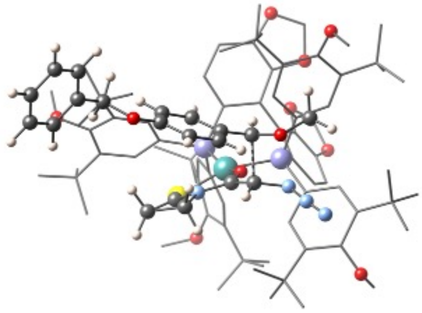

(TS1-A2) +0.00 kcal·mol^{-1}

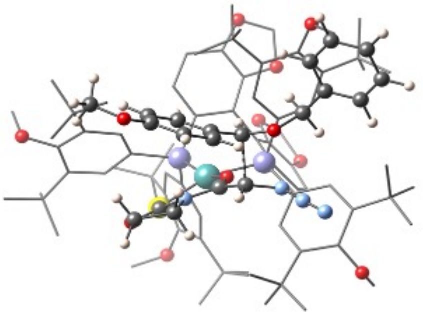

(TS2-A1) +0.00 kcal·mol^{-1}

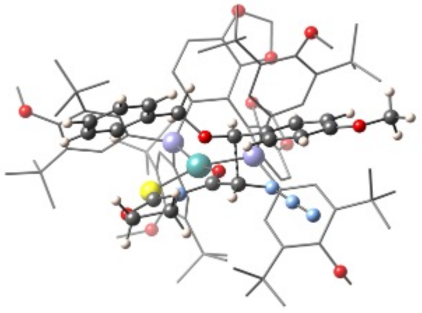

(TS2-A2) +0.19 kcal·mol^{-1}

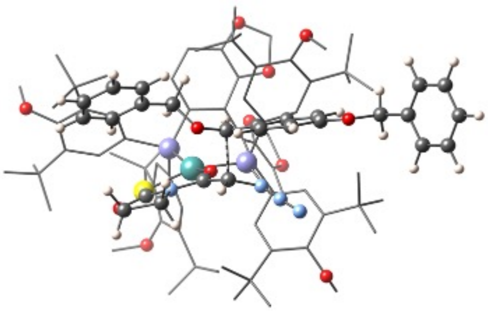

(TS1-A1) +0.00 kcal·mol^{-1}

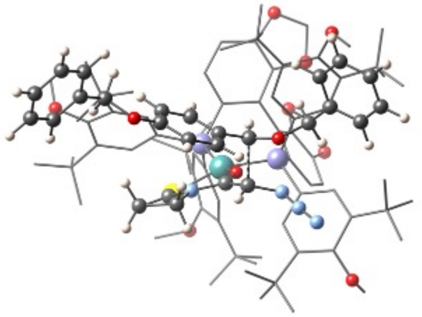

(TS1-A2) +0.44 kcal·mol^{-1}

Fig. S3 Favored transition states for diastereomer **A1** (left) and **A2** (right) using the electrophile with: (*a*) R: Bn, (*b*) PG: Bn, and (*c*) R, PG: Bn.

Table S1. Relative energies (in kcal·mol^{-1}) in CH_2Cl_2 for optimized reactants with their population distribution at -20 °C. Some parameters about the environment of nickel atom are also shown, and representation of the relative energy from square-planar continuous shape measures.

(*a*) [{(*S*)-Tol-BINAP}Ni(*N*-azidoacetyl-1,3-thiazolidine-2-thione)]$^+$

Conform.	**R1**	**R2**	**R3**	**R4**
E_{rel}	0.00	0.38	3.42	3.86
%	68.1	31.8	0.1	0.0
P-Ni-S (°)	163.6	165.1	160.1	159.6
P-Ni-O (°)	171.7	173.1	161.1	161.3
P-Ni-P (°)	96.5	96.1	97.0	97.1
S_{SQ-4}	1.84	1.61	3.44	3.50

(*b*) [{(*R*)-DTBM-SEGPHOS}Ni(*N*-azidoacetyl-1,3-oxazolidine-2-thione)]$^+$

Conform.	**R1**	**R2**	**R3**	**R4**
E_{rel}	0.00	1.07	3.84	5.42
%	89.2	10.7	0.0	0.0
P-Ni-S (°)	160.2	158.4	165.5	154.1
P-Ni-O (°)	166.9	166.3	157.0	151.1
P-Ni-P (°)	99.3	99.6	94.3	100.3
S_{SQ-4}	2.75	3.04	3.19	6.25

Table S2. Relative energies (in kcal·mol^{-1}) for the four transition states in CH_2Cl_2 derived from conformation [{(*S*)-Tol-BINAP}Ni(*N*-azidoacetyl-1,3-thiazolidine-2-thione)]$^+$ and the electrophile, and its population distribution at –20 °C. Imaginary frequencies and several geometrical parameters are also shown.

TS	**TS1-A1**	**TS2-A1**	**TS1-A2**
E_{rel}	0.00	0.53	0.77
%	63.7	22.4	13.9
Final	86.1		13.9
ν_{img}/cm^{-1}	312.3 i	330.0 i	315.2 i
C···C / Å	2.111	2.099	2.048
HC···CH (º)	169.8	179.6	164.2
P-Ni-S (º)	162.3	162.4	162.6
P-Ni-O (º)	164.5	165.1	165.1
P-Ni-P (º)	95.2	95.5	96.2
S_{SQ-4}	2.66	2.25	2.58

Table S3. Relative energies (in kcal·mol^{-1}) for the transition states of [{(*R*)-DTBM-SEGPHOS}Ni(*N*-azidoacetyl-1,3-oxazolidine-2-thione)]$^+$ and the electrophile with methyl (*a*) and their substitution by benzyls (*b*-*d*). All energies are provided in CH_2Cl_2, and their population distribution at –20 °C. Imaginary frequencies and several geometrical parameters are also shown.

(a) Electrophile with two R, PG: Me.

TS	**TS1-A1**	**TS2-A2**
E_{rel}	1.10	0.00
%	10.0	90.0
ν_{img}/cm^{-1}	345.4 *i*	340.7 *i*
C···C / Å	2.073	2.068
HC···CH (º)	159.3	163.3
P-Ni-S (º)	159.8	160.6
P-Ni-O (º)	163.9	160.3
P-Ni-P (º)	95.5	96.1
S_{SQ-4}	3.02	3.39

(b) Electrophile from R: Me and PG: Bn.

TS	**TS1-A1**	**TS1-A2**
E_{rel}	2.51	0.00
%	0.7	99.3
ν_{img}/cm^{-1}	328.6 *i*	347.7 *i*
C···C / Å	2.117	2.051
HC···CH (º)	154.9	164.4
P-Ni-S (º)	160.8	160.8
P-Ni-O (º)	164.1	160.7
P-Ni-P (º)	95.5	96.2
S_{SQ-4}	2.87	3.32

(c) Electrophile from R: Bn and PG: Me.

TS	**TS2-A1**	**TS2-A2**
E_{rel}	0.00	0.19
%	59.3	40.7
ν_{img}/cm^{-1}	333.8 *i*	357.7 *i*
C···C / Å	2.074	2.043
HC···CH (º)	163.8	160.7
P-Ni-S (º)	162.3	161.3
P-Ni-O (º)	161.9	161.2
P-Ni-P (º)	96.0	96.6
S_{SQ-4}	2.98	3.02

(d) Electrophile with R, PG: Bn.

TS	**TS1-A1**	**TS1-A2**
E_{rel}	0.00	0.44
%	70.4	29.6
ν_{img}/cm^{-1}	348.8 *i*	366.0 *i*
C···C / Å	2.049	2.028
HC···CH (º)	163.4	160.3
P-Ni-S (º)	162.4	160.7
P-Ni-O (º)	162.2	160.6
P-Ni-P (º)	96.0	96.6
S_{SQ-4}	2.93	3.35

9.4. Computational data

Atomic coordinates for reactants and transition states for BINAP system.

(a) [{(S)-Tol-BINAP}Ni(α-azidoacetyl-1,3-thiazolidine-2-thione)]$^+$ (**R1**)

Ni	-1.33551100	0.40004800	0.54533900
S	-2.71329500	2.10582700	1.03694300
C	-4.28012600	1.56976900	0.62333500
S	-5.58878200	2.74012200	0.68228000
C	-6.82940300	1.40054400	0.42094000
C	-6.05984500	0.30099400	-0.29500300
N	-4.66553000	0.36067300	0.20793100
C	-3.77622000	-0.76294500	0.12668900
O	-2.75762600	-0.76469300	0.93328800
H	-7.20223600	1.07942100	1.39178500
H	-7.64954100	1.78484400	-0.17979900
H	-6.47652800	-0.67932100	-0.07490900
H	-6.05181400	0.45945400	-1.37650900
C	-4.07251100	-1.77546200	-0.73188600
H	-4.96630600	-1.78837200	-1.33938200
N	-3.19409900	-2.85530200	-0.80992100
N	-3.48474700	-3.79150300	-1.56333600
N	-3.61607800	-4.70630900	-2.21069600
P	0.27194400	1.79239200	-0.22981600
P	-0.02644100	-1.45793600	0.67903300
C	1.39504900	0.96399800	-1.43214200
C	-0.49032500	3.22210200	-1.08313700
C	1.39529400	2.45755600	1.03733400
C	1.68931700	-1.09119900	1.23431700
C	-0.76818200	-2.63106500	1.87189400
C	0.20016900	-2.34553400	-0.89187800
C	-0.18563100	4.55083300	-0.74408000
C	2.56829900	-0.33581600	0.41819100
C	2.10551400	-1.50237800	2.51602500
C	2.28053600	-0.06354600	-1.01604800
C	3.04874100	-0.81018000	-2.05202400
C	3.34045000	-1.14666200	3.05941600
C	4.23907400	-0.36108100	2.34495800
C	3.89205400	0.06506500	0.97233100
C	2.88023600	-0.42339200	-3.46882000
C	2.03776800	0.63634600	-3.78714800
C	1.32046400	1.30818400	-2.79688600
C	3.90066800	-1.88554900	-1.75002300
C	4.56416200	-2.56916700	-2.77244300
C	4.39765500	-2.19922200	-4.11057900
C	3.56529100	-1.13356900	-4.46073300
C	5.46196000	0.03431700	2.89794000
C	6.34364400	0.82343400	2.15555700
C	6.02526800	1.22430100	0.85427600
C	4.81509800	0.85123300	0.26320900
C	-0.83997800	5.60957400	-1.38077700
C	-1.82294200	5.35772800	-2.34766200
C	-2.15125000	4.03193700	-2.66565300
C	-1.49594700	2.97441100	-2.03048700
C	1.17484600	2.13816700	2.38183900
C	2.09163100	2.53681100	3.35836600
C	3.23593100	3.26435200	2.99927900
C	3.44489700	3.60161200	1.65275500
C	2.53335000	3.19180400	0.67574300
C	-1.07854600	-3.95679400	1.52654400
C	-1.69793400	-4.80115800	2.45252700
C	-2.03282800	-4.32991700	3.72930000
C	-1.74979200	-2.99856300	4.06715400
C	-1.13213600	-2.15476100	3.14079200
C	-0.43450000	-1.86887500	-2.04452300

C	-0.19214100	-2.47212100	-3.28101500
C	0.69014400	-3.55824300	-3.37673700
C	1.31768300	-4.04211800	-2.21787500
C	1.08042700	-3.43221600	-0.98301900
H	1.46669900	-2.10971400	3.13421500
H	3.58690500	-1.48171500	4.06015100
H	1.91518000	0.94248700	-4.81947600
H	0.68588200	2.11124400	-3.12953600
H	4.59911000	1.19977100	-0.73786400
H	7.28512200	1.12905500	2.59334200
H	6.72260500	1.83881600	0.29976000
H	5.73489700	-0.26177600	3.90373800
H	3.45314900	-0.87051300	-5.50573400
H	4.04139800	-2.21769900	-0.73017900
H	5.21006200	-3.40180000	-2.52560800
H	4.91874200	-2.74701300	-4.88503000
H	-0.58823500	6.62820400	-1.11173300
H	-2.91790100	3.81435600	-3.39878500
H	-1.77382000	1.95626500	-2.27482300
H	0.53830400	4.78525900	0.01982000
H	0.31042200	1.55242300	2.66894900
H	1.91938900	2.26389000	4.39215600
H	2.72631500	3.42422900	-0.36393600
H	4.32564200	4.15601100	1.35382100
H	-0.86849900	-4.34603800	0.54308500
H	-1.92628600	-5.82177400	2.17066600
H	-2.01174200	-2.60994300	5.04339300
H	-0.93469000	-1.12483100	3.41221900
H	1.59745800	-3.79191900	-0.10242100
H	2.00904000	-4.87364400	-2.27178600
H	-1.09726100	-1.01412900	-1.98876600
H	-0.67879900	-2.08279200	-4.16665700
C	-2.69894900	-5.24777700	4.71099800
H	-2.04181300	-6.12023500	4.91063000
H	-3.66200700	-5.60727600	4.29124000
H	-2.90467800	-4.73457800	5.67447600
C	0.96835200	-4.17429600	-4.71497600
H	0.02333700	-4.55987100	-5.15196600
H	1.68943400	-5.01535600	-4.63447300
H	1.39772300	-3.40808400	-5.39451100
C	4.23073400	3.65344100	4.05161900
H	3.73764300	4.29335100	4.81321700
H	5.08480000	4.21541800	3.61728700
H	4.62647000	2.74030100	4.54439200
C	-2.51908700	6.50468800	-3.01823700
H	-1.77414800	7.13330100	-3.55002300
H	-3.03776400	7.12327500	-2.25584100
H	-3.27105200	6.15075700	-3.75528000

(*b*) [{(*S*)-Tol-BINAP}Ni(α-azidoacetyl-1,3-thiazolidine-2-thione)]$^+$ (**R2**)

Ni	-1.35592700	0.43953500	0.42271300
S	-2.72526200	2.19617200	0.74049900
C	-4.32624500	1.62372000	0.57049000
S	-5.64320000	2.72119200	0.94705500
C	-6.85276500	1.57663400	0.15341700
C	-6.19780200	0.20475600	0.23078500
N	-4.73071500	0.42362800	0.15773000
C	-3.81724500	-0.68099000	-0.00080000
O	-2.79158800	-0.70471000	0.79922400
H	-7.79214900	1.61348200	0.69889200
H	-7.01069300	1.90167300	-0.87323800
H	-6.41446700	-0.30206100	1.17420400
H	-6.52185100	-0.42949100	-0.59001400
C	-4.11041400	-1.64742600	-0.90611500
H	-5.00031600	-1.61887500	-1.51958600
N	-3.23826900	-2.72827500	-1.03375900

N	-3.52616000	-3.61939400	-1.84119700
N	-3.65525800	-4.49704400	-2.53815400
P	0.32864000	1.78426500	-0.29808100
P	-0.08268700	-1.43441800	0.67114000
C	1.50447000	0.90767100	-1.41406700
C	-0.33950100	3.21826500	-1.22258300
C	1.38886400	2.44687200	1.02389000
C	1.60911600	-1.09164900	1.30997500
C	-0.90996100	-2.57707500	1.83832700
C	0.20901900	-2.35451700	-0.87041300
C	-0.02229400	4.54475400	-0.88569500
C	2.54597700	-0.37101300	0.52782500
C	1.94929900	-1.48757900	2.61864300
C	2.34318900	-0.12741400	-0.92562100
C	3.15292400	-0.91342100	-1.89916300
C	3.16148900	-1.14729700	3.21987800
C	4.11298900	-0.39426200	2.53982500
C	3.84739400	0.01288600	1.14357300
C	3.07602700	-0.55469500	-3.33102100
C	2.27835100	0.51586800	-3.71991900
C	1.51917900	1.22385500	-2.78775700
C	3.96071700	-2.00031300	-1.52546100
C	4.66619100	-2.72105300	-2.49275600
C	4.58628200	-2.37736600	-3.84577100
C	3.80085500	-1.30154100	-4.26616600
C	5.31419300	-0.01511500	3.14875200
C	6.25094000	0.74019300	2.43939100
C	6.00972300	1.12289700	1.11624600
C	4.82366600	0.76499400	0.46952900
C	-0.59768500	5.60837600	-1.58728400
C	-1.51426600	5.36470700	-2.61921800
C	-1.85823200	4.04256000	-2.93555700
C	-1.28254700	2.98011600	-2.23472500
C	1.07871000	2.15862500	2.35765100
C	1.94425500	2.55307200	3.38140800
C	3.12650900	3.24553600	3.08050300
C	3.42512600	3.55283800	1.74372700
C	2.56464000	3.14696400	0.72000100
C	-1.24860000	-3.89375700	1.48404300
C	-1.93829500	-4.71212700	2.38321500
C	-2.31733500	-4.22283700	3.64061200
C	-2.00676200	-2.89957300	3.98587000
C	-1.31876700	-2.08164300	3.08621600
C	-0.35186300	-1.88569300	-2.06380200
C	-0.05793300	-2.51743700	-3.27467200
C	0.80201500	-3.62507900	-3.30351000
C	1.35504500	-4.10111300	-2.10417900
C	1.06659300	-3.46276300	-0.89478100
H	1.26744800	-2.07145900	3.21256800
H	3.34828200	-1.46910700	4.23770400
H	2.22392100	0.80244500	-4.76366300
H	0.92506600	2.03317600	-3.17453100
H	4.66794600	1.09871200	-0.54768200
H	7.17525200	1.03327800	2.92016300
H	6.74881200	1.71112300	0.58790200
H	5.52786100	-0.29811300	4.17248500
H	3.75567200	-1.05935900	-5.32120900
H	4.03414700	-2.31289600	-0.49245000
H	5.27687200	-3.56217100	-2.19114600
H	5.13834000	-2.95377300	-4.57678900
H	-0.33593700	6.62476900	-1.31937700
H	-2.57504200	3.83180200	-3.71939300
H	-1.57084700	1.96542200	-2.48122700
H	0.65143400	4.77451900	-0.07586600
H	0.18374300	1.59960100	2.60122800
H	1.70258100	2.30380700	4.40723200
H	2.82611000	3.35508300	-0.30981500

H	4.33609800	4.08006600	1.48966200
H	-1.00819800	-4.29482200	0.51224900
H	-2.18762400	-5.72600300	2.09476700
H	-2.30236600	-2.49668600	4.94657100
H	-1.10287400	-1.05645700	3.36137500
H	1.52631600	-3.81803700	0.01875800
H	2.02811100	-4.94927700	-2.10611000
H	-0.99653500	-1.01545000	-2.05900300
H	-0.48722600	-2.13404100	-4.19198500
C	-3.06057800	-5.11223800	4.59269900
H	-2.44392300	-6.00535400	4.82706200
H	-4.01410700	-5.44124300	4.12848400
H	-3.29469400	-4.58700400	5.54311000
C	1.13592900	-4.27304800	-4.61370400
H	0.20639900	-4.64465300	-5.09392100
H	1.83081700	-5.12915500	-4.47947900
H	1.61955000	-3.53036300	-5.28282100
C	4.06571500	3.62885300	4.18466500
H	3.54435500	4.29459800	4.90409900
H	4.95873300	4.16131800	3.79366100
H	4.40718100	2.71524500	4.71569000
C	-2.12369400	6.51683300	-3.36160400
H	-1.32264000	7.11196300	-3.84845700
H	-2.67815100	7.16646400	-2.65205500
H	-2.82951000	6.16917000	-4.14577400

(*c*) [{(*S*)-Tol-BINAP}Ni(α-azidoacetyl-1,3-thiazolidine-2-thione)]$^+$ (**R3**)

Ni	1.13861400	0.73725200	0.53502000
S	2.10875700	2.75069000	0.65465500
C	3.66599300	2.41284300	0.02088300
S	4.43559200	3.71135200	-0.87560200
C	6.04507100	2.82186400	-0.82901900
C	5.66219600	1.35769600	-0.69153900
N	4.40783800	1.30498200	0.10974600
C	3.96254800	0.06780800	0.68150400
O	2.73585900	-0.03346900	1.08389700
H	6.59611800	3.01607800	-1.74572800
H	6.61620500	3.18043200	0.02539900
H	5.46753400	0.88974600	-1.65888900
H	6.45010400	0.80968700	-0.18508100
C	4.85410700	-0.95683900	0.82049600
H	5.90904200	-0.85435000	0.62264200
N	4.38990400	-2.17108900	1.32091900
N	5.20867600	-3.08361400	1.47392700
N	5.84057100	-4.00031700	1.66017000
P	-0.92293200	1.63919200	0.59860900
P	0.52634500	-1.29048700	-0.27599700
C	-2.18164000	0.41884700	1.16535200
C	-0.96594800	3.05686400	1.75829400
C	-1.55487800	2.23355900	-0.99977900
C	-0.85417300	-1.18901000	-1.48699100
C	1.95442500	-2.03151800	-1.14275200
C	-0.04901800	-2.48766500	0.96526700
C	-1.44712500	4.32186300	1.38152800
C	-2.15053300	-0.79204200	-1.07447300
C	-0.59888400	-1.43647100	-2.85061700
C	-2.53134500	-0.69638700	0.36069300
C	-3.42795500	-1.73995400	0.93302800
C	-1.56301800	-1.25962400	-3.84345000
C	-2.84715600	-0.82775800	-3.52770100
C	-3.20097900	-0.58996000	-2.11179100
C	-3.93736000	-1.55357100	2.30815700
C	-3.59303600	-0.40269600	3.00901400
C	-2.74342700	0.55298800	2.45087700
C	-3.79067500	-2.90593500	0.23863800
C	-4.60639400	-3.86435600	0.84604200

C	-5.07928600	-3.68407500	2.14951200
C	-4.75249200	-2.53780400	2.87778400
C	-3.80735400	-0.60851600	-4.52135100
C	-5.08678600	-0.16866300	-4.17392100
C	-5.42996200	0.05409300	-2.83677800
C	-4.50207600	-0.15309100	-1.81255600
C	-1.41358700	5.38803100	2.28517100
C	-0.87999100	5.21327300	3.56928900
C	-0.36962600	3.95992000	3.93718700
C	-0.40323800	2.89484100	3.03367200
C	-0.72198600	2.18691500	-2.12340200
C	-1.22089500	2.53040900	-3.38260300
C	-2.55778400	2.92916100	-3.52889200
C	-3.38621500	2.99485300	-2.39745900
C	-2.88878800	2.64046800	-1.14033800
C	2.39975600	-3.33788600	-0.88365300
C	3.53873100	-3.83445700	-1.52453400
C	4.25822000	-3.02894500	-2.41787800
C	3.82160900	-1.72023600	-2.66941600
C	2.68072800	-1.22610000	-2.03292500
C	-0.03540100	-2.12776600	2.31743000
C	-0.59000000	-2.97894900	3.27689000
C	-1.16056900	-4.20136600	2.89257800
C	-1.15679900	-4.56907800	1.53780500
C	-0.61233700	-3.71132500	0.57808000
H	0.37279800	-1.76559700	-3.17882800
H	-1.29150700	-1.45029500	-4.87512100
H	-3.97184200	-0.24280700	4.01172800
H	-2.52215600	1.41013000	3.06282700
H	-4.80222000	0.05189300	-0.79371200
H	-5.82146800	0.00363200	-4.94976100
H	-6.42601300	0.39974700	-2.59221300
H	-3.56872000	-0.77105900	-5.56558400
H	-5.13297100	-2.42457000	3.88585600
H	-3.43014200	-3.09431700	-0.76374000
H	-4.86936800	-4.76264000	0.30261600
H	-5.70576700	-4.44289400	2.60017000
H	-1.79562600	6.35474800	1.98042400
H	0.05951500	3.80623800	4.91944500
H	0.00831200	1.93723500	3.32850500
H	-1.83507500	4.50522700	0.39244700
H	0.30438100	1.85449600	-2.02879100
H	-0.57068900	2.46949700	-4.24654300
H	-3.54786200	2.65914600	-0.28158600
H	-4.42290500	3.29331600	-2.49007200
H	1.89662800	-3.97360800	-0.17311300
H	3.87178400	-4.84252000	-1.30979100
H	4.36786400	-1.07746900	-3.34836200
H	2.36806300	-0.20655800	-2.22319400
H	-0.65332700	-3.98774100	-0.46791600
H	-1.60010300	-5.50420400	1.21936000
H	0.37451400	-1.17351500	2.62416500
H	-0.59129200	-2.67798300	4.31722300
C	5.49103600	-3.56879000	-3.07940800
H	5.23017600	-4.46659800	-3.67832400
H	6.23499900	-3.85203100	-2.30520800
H	5.95406400	-2.81751600	-3.75404500
C	-1.78615300	-5.08809600	3.92726900
H	-1.02460100	-5.37584900	4.68218500
H	-2.20149600	-6.01321900	3.47396500
H	-2.61203100	-4.54397700	4.43238000
C	-0.84944100	6.36772800	4.52651900
H	-1.88404600	6.72562900	4.71195500
H	-0.25118200	7.19635700	4.09247400
H	-0.39731100	6.07917300	5.49928100
C	-3.09062500	3.25920400	-4.89099800
H	-2.51311700	4.10309800	-5.32341500

H	-4.16232400	3.54843900	-4.85033200
H	-2.99160000	2.37326400	-5.55321500

(*d*) [{(*S*)-Tol-BINAP}Ni(α-azidoacetyl-1,3-thiazolidine-2-thione)]$^+$ (**R4**)

Ni	-1.12705100	0.73342700	-0.55211900
S	-2.08338200	2.75759700	-0.68415700
C	-3.63785800	2.43563100	-0.03091200
S	-4.43837300	3.77328600	0.77778100
C	-5.74932400	2.64766900	1.41625400
C	-5.74669400	1.46464400	0.46248100
N	-4.36447700	1.31515800	-0.06638600
C	-3.94987400	0.08392800	-0.66658100
O	-2.72866000	-0.03624200	-1.08015700
H	-5.48475700	2.36036600	2.43232800
H	-6.70555300	3.16439000	1.42064000
H	-6.03053500	0.55156100	0.98039900
H	-6.41829500	1.62800700	-0.38299000
C	-4.86770600	-0.91620500	-0.81935200
H	-5.91875400	-0.78808200	-0.61255500
N	-4.43944400	-2.12606100	-1.36041000
N	-5.28257000	-3.01168000	-1.53548700
N	-5.93960100	-3.90574100	-1.74401400
P	0.93908300	1.62055200	-0.62993400
P	-0.53452900	-1.28244200	0.29165800
C	2.19211200	0.38166500	-1.16702000
C	0.99023900	3.01140200	-1.82074600
C	1.57200900	2.24526300	0.95636700
C	0.84067700	-1.17160000	1.50777700
C	-1.97554000	-1.99769800	1.15865400
C	0.03866100	-2.50426400	-0.92623100
C	1.47657200	4.28243300	-1.47224700
C	2.14060300	-0.78643900	1.09545000
C	0.57839500	-1.40021800	2.87321000
C	2.53004300	-0.72013300	-0.33921300
C	3.42179100	-1.78078400	-0.88699700
C	1.53836900	-1.21236500	3.86802700
C	2.82514600	-0.78832800	3.55227100
C	3.18657300	-0.57249400	2.13474700
C	3.94091300	-1.62336600	-2.26215400
C	3.60823100	-0.48370400	-2.98667700
C	2.76157300	0.48778500	-2.45176400
C	3.77060600	-2.93695700	-0.16950800
C	4.58329200	-3.91206900	-0.75398500
C	5.06657900	-3.75857100	-2.05706700
C	4.75282700	-2.62346100	-2.80823400
C	3.78131100	-0.55763800	4.54722500
C	5.06388800	-0.12734600	4.19947000
C	5.41437600	0.07399100	2.86078100
C	4.49071200	-0.14530300	1.83525600
C	1.44742200	5.32816200	-2.39971700
C	0.91325900	5.12666200	-3.67975900
C	0.39805800	3.86737300	-4.01952600
C	0.42729800	2.82282900	-3.09228400
C	0.73938300	2.22272900	2.08096500
C	1.24027500	2.58746900	3.33344700
C	2.57885300	2.98345000	3.47184400
C	3.40703600	3.02475800	2.33910900
C	2.90765400	2.64905900	1.08902200
C	-2.44924000	-3.29248200	0.89166800
C	-3.60423700	-3.76456400	1.52239100
C	-4.31209900	-2.94513000	2.41243000
C	-3.84762900	-1.64738800	2.67108800
C	-2.69019700	-1.17830500	2.04562000
C	0.04221400	-2.16549900	-2.28385100
C	0.59725600	-3.03758100	-3.22408400
C	1.15065600	-4.25984400	-2.81476100

C	1.12812200	-4.60668100	-1.45467100
C	0.58295900	-3.72837700	-0.51426600
H	-0.39534100	-1.72352800	3.20090700
H	1.26158700	-1.38835100	4.90089500
H	3.99372600	-0.34539100	-3.99005500
H	2.54884400	1.33460800	-3.08107900
H	4.79644700	0.04282500	0.81480400
H	5.79543400	0.05400000	4.97621700
H	6.41284800	0.41226800	2.61588700
H	3.53713700	-0.70406900	5.59257000
H	5.14069000	-2.53132500	-3.81563300
H	3.40183700	-3.10464500	0.83355600
H	4.83595800	-4.80230900	-0.19270900
H	5.69076600	-4.52974300	-2.48956800
H	1.83316300	6.30000000	-2.11672400
H	-0.03160700	3.69330300	-4.99815500
H	0.01197900	1.86046400	-3.36556600
H	1.86502300	4.48608500	-0.48729300
H	-0.28814100	1.89207900	1.99306500
H	0.59032900	2.54491200	4.19868300
H	3.56667200	2.64842900	0.23001800
H	4.44506000	3.32033600	2.42580700
H	-1.95627500	-3.93667900	0.18131700
H	-3.95911100	-4.76385500	1.30156000
H	-4.38554100	-0.99308000	3.34566500
H	-2.35726500	-0.16580600	2.23910100
H	0.60876500	-3.98931200	0.53619100
H	1.55732500	-5.54170300	-1.11714000
H	-0.35404900	-1.21172800	-2.60946400
H	0.61268900	-2.75294400	-4.26888600
C	-5.56282100	-3.45792800	3.06190300
H	-5.32814100	-4.36330200	3.66026900
H	-6.30662200	-3.72153600	2.28063700
H	-6.01397000	-2.69802200	3.73491900
C	1.77837500	-5.16873200	-3.82857100
H	1.02226200	-5.46036000	-4.58742600
H	2.17915700	-6.09083100	-3.35638300
H	2.61537800	-4.64080700	-4.33253900
C	0.88717000	6.25931900	-4.66281500
H	1.92313200	6.60895800	-4.85622000
H	0.29201100	7.09978600	-4.24770700
H	0.43395900	5.95057100	-5.62886900
C	3.11382000	3.33636800	4.82738900
H	2.53999100	4.19040300	5.24451100
H	4.18664700	3.62053300	4.78084700
H	3.01171700	2.46317000	5.50585600

(e) Transition state **TS1-A1**, from **R1** and the electrophile in the position **A1**

Ni	-0.11861400	1.07349300	-0.05954000
S	0.25395200	3.25539900	0.09856000
C	1.68094700	3.55485200	-0.79185400
S	1.87915500	5.17526300	-1.38412600
C	3.65020100	4.86834000	-1.78093700
C	3.75874200	3.36253300	-1.96022600
N	2.71246100	2.72994100	-1.09055800
C	2.73273200	1.34823100	-0.81690800
O	1.69907400	0.75651300	-0.39138400
H	3.90852100	5.40699100	-2.68949200
H	4.25536800	5.23703700	-0.95546200
H	3.56505400	3.05573600	-2.98947000
H	4.74014900	3.02633600	-1.64643500
C	3.95450400	0.63425400	-0.93928900
H	4.75012200	1.06553000	-1.52826100
N	3.78778900	-0.76611100	-1.01099100
N	4.72124800	-1.41243700	-1.51250400
N	5.46698500	-2.12754300	-1.95892500

P	-2.15844100	1.27598700	0.90296500
P	-0.50000900	-1.01872400	-0.91180900
C	-2.73455800	-0.30536200	1.65433400
C	-2.08956700	2.53107700	2.23547400
C	-3.49234800	1.76447100	-0.23292600
C	-2.21624200	-1.24165800	-1.52603800
C	0.63124900	-1.26683200	-2.32472300
C	-0.22477100	-2.38023500	0.26055800
C	-2.98787700	3.60914200	2.29976200
C	-3.30813100	-1.28721900	-0.62485400
C	-2.44983800	-1.28751400	-2.91448400
C	-3.11421200	-1.40819500	0.84526500
C	-3.43311300	-2.70605600	1.50562800
C	-3.73215400	-1.33907800	-3.46224700
C	-4.86032300	-1.34848900	-2.64779300
C	-4.69064400	-1.33640600	-1.17858900
C	-3.36274200	-2.78147100	2.98031200
C	-3.03505300	-1.63960500	3.70311800
C	-2.73452400	-0.44014000	3.05720800
C	-3.76427700	-3.87069600	0.79284400
C	-4.01432200	-5.06666600	1.47117000
C	-3.94178300	-5.13085600	2.86602600
C	-3.62123500	-3.99910900	3.61918800
C	-6.15026100	-1.35987500	-3.18948300
C	-7.26394300	-1.35838300	-2.34632300
C	-7.11112800	-1.34946700	-0.95636100
C	-5.84127100	-1.33869100	-0.37296200
C	-2.86204800	4.57391400	3.30364200
C	-1.82625100	4.48857900	4.24400100
C	-0.91012900	3.42984000	4.16397500
C	-1.03668400	2.46576800	3.16109700
C	-3.18558000	2.05124000	-1.56781700
C	-4.20857700	2.31414300	-2.48305200
C	-5.54908500	2.29741500	-2.06985300
C	-5.85363300	2.02931600	-0.72608600
C	-4.83025100	1.75568500	0.18550600
C	1.39587800	-2.43392100	-2.48366500
C	2.28987900	-2.55080500	-3.55191900
C	2.44940900	-1.49695300	-4.46200900
C	1.69618600	-0.32541200	-4.29736100
C	0.79538800	-0.21297100	-3.23571400
C	0.26358400	-2.08990500	1.53925800
C	0.37205000	-3.10193900	2.49658800
C	-0.00461900	-4.41607300	2.18222700
C	-0.47739200	-4.70888800	0.89331800
C	-0.59498600	-3.69352800	-0.05982600
H	-1.62764500	-1.26820700	-3.61112100
H	-3.83939800	-1.35534200	-4.54051200
H	-2.99415200	-1.67429900	4.78552800
H	-2.48553000	0.39278800	3.69118100
H	-5.76796000	-1.31040800	0.70588800
H	-8.25802500	-1.36172400	-2.77433500
H	-7.98898600	-1.34282900	-0.32331800
H	-6.29869900	-1.36362500	-4.26268400
H	-3.57026400	-4.07843700	4.69850500
H	-3.80946900	-3.87098400	-0.28786500
H	-4.25938600	-5.95800800	0.90824600
H	-4.13468800	-6.06993000	3.36835400
H	-3.56733800	5.39513100	3.34239500
H	-0.09423300	3.35195900	4.87165000
H	-0.31123600	1.66337300	3.10339000
H	-3.77777700	3.72797000	1.57564200
H	-2.15698500	2.03920500	-1.90616500
H	-3.95789800	2.51333600	-3.51761800
H	-5.08080500	1.51214400	1.21028300
H	-6.88217200	2.00400400	-0.38850000
H	1.32805700	-3.25136200	-1.78467800

H	2.87351700	-3.45696700	-3.65920500
H	1.80945000	0.50406600	-4.98411900
H	0.23102300	0.70363300	-3.11456100
H	-0.99917600	-3.92481400	-1.03728100
H	-0.78127200	-5.71506900	0.63313600
H	0.53175100	-1.07447800	1.80348700
H	0.73347000	-2.85896300	3.48813700
C	3.43080000	-1.62878600	-5.58826200
H	3.15332300	-2.49484700	-6.22526700
H	4.44933000	-1.79186100	-5.17702200
H	3.44908500	-0.71676400	-6.22211100
C	0.08135500	-5.48529700	3.22984900
H	1.13143100	-5.58486100	3.57641500
H	-0.25598400	-6.46826900	2.83762600
H	-0.56254400	-5.21249900	4.09253600
C	-6.63920600	2.54363400	-3.06957000
H	-6.51351700	3.55119500	-3.51860100
H	-7.64385700	2.48943500	-2.59873300
H	-6.58690900	1.77815700	-3.87238200
C	-1.70249100	5.53380000	5.31263600
H	-2.62515100	5.54649100	5.93019800
H	-1.56531500	6.53127100	4.84449900
H	-0.83591000	5.33545300	5.97871000
C	4.85607800	1.06958400	0.91962800
H	3.98901500	0.81549700	1.52801400
C	5.95495700	0.14853600	0.95810500
C	7.24435700	0.50090800	0.49065200
C	8.27206600	-0.40355600	0.53276500
C	8.05786100	-1.70609200	1.04052200
C	6.78394600	-2.06807100	1.51749200
C	5.75490600	-1.15022200	1.46804500
H	7.42402700	1.50101200	0.11856200
H	9.26623700	-0.14713100	0.19163800
H	6.60759300	-3.04997600	1.93137100
H	4.77913600	-1.43135200	1.84588200
O	9.12095500	-2.50191300	1.03652100
C	9.03226700	-3.84788100	1.53872000
H	8.75933300	-3.84656200	2.59522500
H	10.02718700	-4.26407400	1.41720500
H	8.31517500	-4.42876600	0.95635500
O	5.12104700	2.39915600	0.81608900
C	4.71621400	3.19091100	1.95564500
H	3.64650200	3.07287900	2.15003100
H	4.93969300	4.22463200	1.70640800
H	5.28711700	2.89960600	2.83797700

(*f*) Transition state **TS2-A1**, from **R2** and the electrophile in the position **A1**

Ni	-0.12254200	1.08241300	-0.26324900
S	0.27392500	3.26015100	-0.12229000
C	1.74415300	3.56891300	-0.93460700
S	2.06520400	5.22709200	-1.34349600
C	3.52205800	4.74726700	-2.36662400
C	3.95891000	3.39617900	-1.82360900
N	2.73591300	2.71606100	-1.28669000
C	2.70381900	1.32621900	-1.09046900
O	1.61676900	0.73170800	-0.84062500
H	3.19993200	4.70287400	-3.40520100
H	4.29793500	5.50061500	-2.25622900
H	4.38420000	2.78521600	-2.61595600
H	4.67094300	3.49298600	-1.00491700
C	3.92107500	0.59395600	-1.08640400
H	4.80342200	1.04971700	-1.51202000
N	3.74935600	-0.79795700	-1.28205000
N	4.69807700	-1.41036000	-1.79556200
N	5.46064300	-2.09396800	-2.26233600
P	-2.03032000	1.27086300	0.93369800

P	-0.58685100	-0.99892500	-1.08631400
C	-2.51972400	-0.32002800	1.72328900
C	-1.78995500	2.49985400	2.26955700
C	-3.48444500	1.78201300	-0.03061200
C	-2.35751000	-1.23219500	-1.50796300
C	0.40150700	-1.23161200	-2.60584300
C	-0.17149700	-2.35627500	0.04772400
C	-2.66824600	3.57768700	2.46758100
C	-3.34240500	-1.27222300	-0.49123500
C	-2.74279400	-1.28299000	-2.86150600
C	-2.98639100	-1.41197300	0.94638700
C	-3.22518500	-2.72026400	1.61892500
C	-4.07835600	-1.32391100	-3.26371700
C	-5.10941400	-1.31982200	-2.32905200
C	-4.77767300	-1.31063400	-0.88776300
C	-2.99079200	-2.81618100	3.07539100
C	-2.58767000	-1.68318700	3.77380200
C	-2.36292700	-0.47382200	3.11552800
C	-3.62699800	-3.87664000	0.92966600
C	-3.79478000	-5.08367200	1.61361900
C	-3.56858200	-5.16711800	2.99094000
C	-3.17182100	-4.04427000	3.72083000
C	-6.45158300	-1.31775900	-2.72394400
C	-7.46452400	-1.30721100	-1.76206200
C	-7.15804800	-1.30373500	-0.39762200
C	-5.83114200	-1.30622700	0.04096600
C	-2.41144200	4.52273600	3.46547300
C	-1.26428200	4.41778500	4.26427800
C	-0.37001400	3.35930700	4.04819000
C	-0.62822300	2.41419400	3.05272300
C	-3.33364300	2.08101800	-1.38935300
C	-4.45434700	2.36085600	-2.17580000
C	-5.73727300	2.34860300	-1.60859400
C	-5.88462300	2.06731900	-0.24118900
C	-4.76385400	1.77663300	0.54151100
C	1.23391700	-2.34669600	-2.79577000
C	2.04255400	-2.43786800	-3.93226600
C	2.04506000	-1.41026000	-4.88522000
C	1.22528300	-0.28904700	-4.69047000
C	0.41507400	-0.19967600	-3.55597300
C	0.44987400	-2.06143100	1.26640100
C	0.67832800	-3.07442900	2.20114900
C	0.29005500	-4.39349800	1.92330000
C	-0.31433100	-4.69040300	0.69138300
C	-0.54926500	-3.67467000	-0.23937700
H	-2.00186600	-1.27616300	-3.64401400
H	-4.30450400	-1.34210500	-4.32337800
H	-2.42608700	-1.73302200	4.84426800
H	-2.04533600	0.35122600	3.72932000
H	-5.63743400	-1.28205800	1.10499800
H	-8.50000400	-1.30008000	-2.07686800
H	-7.95996700	-1.29077200	0.32911700
H	-6.71839600	-1.31860200	-3.77398600
H	-3.00103300	-4.13871800	4.78653000
H	-3.79143200	-3.86160600	-0.13934000
H	-4.09624700	-5.96843400	1.06798900
H	-3.70052500	-6.11442300	3.49749300
H	-3.10286700	5.34399900	3.60965100
H	0.52962900	3.26675100	4.64384600
H	0.08051100	1.61150700	2.88838400
H	-3.54356400	3.71050700	1.85220600
H	-2.35181300	2.06577700	-1.84601700
H	-4.32472800	2.57018700	-3.23043100
H	-4.89436000	1.52260100	1.58585200
H	-6.86670400	2.04513100	0.21423800
H	1.28779500	-3.13933700	-2.06677400
H	2.68105300	-3.30323100	-4.06168500

H	1.21946200	0.52108200	-5.40894700
H	-0.19531700	0.68303100	-3.40902000
H	-1.04775400	-3.90972700	-1.17127400
H	-0.62703600	-5.70092500	0.46021900
H	0.73116200	-1.04279800	1.50382800
H	1.14259800	-2.82877600	3.14824500
C	2.93260000	-1.51457300	-6.08964200
H	2.66473900	-2.42056300	-6.67294400
H	3.99201700	-1.59118700	-5.76576900
H	2.82982400	-0.62732200	-6.75006500
C	0.50642900	-5.46432600	2.95020000
H	1.58937200	-5.54741600	3.18048800
H	0.14457700	-6.45156400	2.59201700
H	-0.04455900	-5.20490100	3.87883600
C	-6.93461100	2.61395100	-2.47126900
H	-6.85344300	3.62542300	-2.92180500
H	-7.87766600	2.56217800	-1.88660400
H	-6.98313800	1.85698400	-3.28235700
C	-1.00134600	5.44164900	5.32844900
H	-1.83778500	5.44346800	6.05868700
H	-0.92098800	6.44797200	4.86612300
H	-0.05782900	5.22896900	5.87483500
C	4.46705600	0.81357700	0.92811500
H	3.55557600	0.35221300	1.30606300
C	5.66588900	0.02698900	1.02124700
C	6.95097600	0.60239900	0.87984400
C	8.07680800	-0.17152600	0.98443300
C	7.97026900	-1.55889700	1.23446600
C	6.69958800	-2.14367000	1.38586400
C	5.57171400	-1.35546100	1.27487100
H	7.04612500	1.66735200	0.71361000
H	9.06677600	0.25511500	0.89262200
H	6.59833200	-3.19686300	1.60276500
H	4.59694400	-1.80963200	1.40718600
O	9.12317000	-2.21357300	1.32231600
C	9.14465800	-3.62723100	1.59034500
H	8.68934900	-3.84173200	2.55863600
H	10.19542700	-3.89802700	1.61116600
H	8.63721000	-4.17718100	0.79602900
O	4.56246200	2.15392600	1.12347200
C	3.74977000	2.68645100	2.19104400
H	2.69772400	2.42429800	2.04771400
H	3.87065700	3.76581600	2.16083500
H	4.09838200	2.30786300	3.15250500

(g) Transition state **TS1-A2**, from **R1** and the electrophile in the position **A2**

Ni	-0.27047800	0.43408700	0.28802100
S	-1.59309200	2.16148800	0.72932500
C	-2.95907100	1.52384100	1.52777400
S	-3.83375400	2.62422800	2.54202600
C	-5.26177400	1.46705700	2.65427500
C	-4.67380400	0.09202200	2.38529000
N	-3.49843800	0.28002300	1.47011900
C	-2.91198400	-0.80700700	0.80194400
O	-1.73041300	-0.74142000	0.36645500
H	-5.69771800	1.53745600	3.64790700
H	-5.99316000	1.75820800	1.90444700
H	-4.31060000	-0.38848800	3.29542200
H	-5.41699500	-0.53558800	1.90686600
C	-3.70039500	-1.96266300	0.52242100
H	-4.60996700	-2.11019200	1.08674900
N	-2.92640800	-3.12259300	0.27191700
N	-3.50849400	-4.21124300	0.41088600
N	-3.88993000	-5.26504300	0.50776000
P	1.38904700	1.82353600	-0.38637400
P	1.04705100	-1.43169600	0.51903500

C	2.60267900	1.02284100	-1.51136500
C	0.68169600	3.25829200	-1.27733600
C	2.39878000	2.47194700	0.97914800
C	2.70512600	-1.08343500	1.21628500
C	0.20895800	-2.59584700	1.64951000
C	1.36590200	-2.30694600	-1.04115500
C	0.94554600	4.58362300	-0.89410700
C	3.69736500	-0.29672900	0.43585000
C	3.02241300	-1.48316100	2.52401900
C	3.51496800	-0.04481600	-1.01181700
C	4.35147200	-0.76725400	-2.00091300
C	4.21460100	-1.11705000	3.15231400
C	5.17172600	-0.32891000	2.51143200
C	4.95560700	0.10233500	1.11124300
C	4.27297500	-0.36038600	-3.42196000
C	3.45038300	0.70226200	-3.79580100
C	2.64810600	1.37325600	-2.87084400
C	5.19407900	-1.83453100	-1.65266200
C	5.93122200	-2.49984700	-2.63545500
C	5.84930000	-2.11682000	-3.97766900
C	5.03150300	-1.05486000	-4.37155900
C	6.33927700	0.06951600	3.17277900
C	7.28316400	0.86310100	2.51604900
C	7.08491900	1.26717500	1.19215800
C	5.93593800	0.89225400	0.49133200
C	0.33571600	5.64765000	-1.56560000
C	-0.56164100	5.40215700	-2.61577100
C	-0.84710600	4.07987100	-2.98094100
C	-0.23727400	3.01724800	-2.31008500
C	2.06616900	2.13443100	2.29587500
C	2.89518300	2.52289500	3.35157200
C	4.06336700	3.25759600	3.10001000
C	4.38510200	3.61192000	1.78027100
C	3.56082200	3.21313500	0.72439600
C	0.02789800	-3.95243300	1.33530600
C	-0.67283400	-4.79037500	2.20773000
C	-1.22010300	-4.28148600	3.39353300
C	-1.05072000	-2.92397000	3.70274100
C	-0.34222200	-2.08908000	2.83542800
C	0.75174100	-1.85336800	-2.21382700
C	1.07510900	-2.43017400	-3.44466400
C	2.01466700	-3.46947300	-3.51341700
C	2.61528900	-3.93618400	-2.33349700
C	2.29807500	-3.35156100	-1.10431500
H	2.32851000	-2.07501900	3.09944500
H	4.38037400	-1.44295100	4.17251000
H	3.41039200	1.01315800	-4.83325500
H	2.03768800	2.17381300	-3.25300500
H	5.81003200	1.24069100	-0.52526900
H	8.17943100	1.17018100	3.03932500
H	7.82857600	1.88508500	0.70580500
H	6.52088400	-0.22671400	4.19899200
H	4.98949000	-0.78119500	-5.41904500
H	5.27050400	-2.17321000	-0.62790700
H	6.56925300	-3.32782300	-2.35480300
H	6.42701900	-2.65030000	-4.72133600
H	0.55668100	6.66203100	-1.25734000
H	-1.54746100	3.87221000	-3.78062700
H	-0.48227100	2.00101800	-2.59403500
H	1.60324700	4.81008300	-0.06994900
H	1.18237500	1.54331600	2.50144200
H	2.63640300	2.23680700	4.36354200
H	3.83977000	3.46005800	-0.29217000
H	5.28602600	4.17229000	1.56416400
H	0.40267800	-4.37201300	0.41572300
H	-0.80587800	-5.83392600	1.94965600
H	-1.47193900	-2.50825700	4.60952700

H	-0.23248600	-1.03991200	3.08143500
H	2.79779400	-3.69364400	-0.20686700
H	3.34869500	-4.73210600	-2.36597700
H	0.04567500	-1.03288300	-2.17821400
H	0.60668600	-2.05574300	-4.34646700
C	-1.98996100	-5.18823000	4.30688200
H	-1.33525200	-6.01769900	4.64769000
H	-2.86037500	-5.61231300	3.76314400
H	-2.36413900	-4.64483800	5.20055200
C	2.38144400	-4.05348100	-4.84475300
H	1.47687300	-4.47924000	-5.32762400
H	3.13931700	-4.85898900	-4.74163400
H	2.80167700	-3.25809000	-5.49593400
C	4.96310400	3.63674100	4.23798700
H	4.40441700	4.26653100	4.96179400
H	5.84925600	4.20581200	3.88477600
H	5.31825400	2.71891800	4.75244900
C	-1.22679500	6.53465000	-3.34027900
H	-2.32859700	6.45189600	-3.23000100
H	-0.96579100	6.49179500	-4.41865000
H	-0.90602800	7.51973500	-2.93945000
C	-4.56291600	-1.49990300	-1.27605100
H	-3.67895500	-1.07253400	-1.74851400
C	-5.67379300	-0.58020600	-1.05104900
C	-6.96299800	-1.04247700	-0.71823800
C	-8.01156100	-0.16107500	-0.58896200
C	-7.81871000	1.21782000	-0.81046100
C	-6.53850900	1.69117800	-1.13574700
C	-5.48477000	0.79377900	-1.24578000
H	-7.14068500	-2.10378500	-0.59934700
H	-9.00988600	-0.50741600	-0.35572200
H	-6.37120600	2.73854700	-1.34215700
H	-4.51064300	1.16921200	-1.54157800
O	-8.91017700	1.98118700	-0.69416600
C	-8.85071100	3.37896600	-1.01462700
H	-9.86241300	3.74924400	-0.88131900
H	-8.53921000	3.52585800	-2.05059600
H	-8.17616500	3.90812100	-0.33737400
O	-4.88619200	-2.73233600	-1.68513300
C	-4.05958800	-3.34378200	-2.70707500
H	-4.25512200	-2.85626700	-3.66258000
H	-4.36482500	-4.38470400	-2.75609500
H	-3.00457800	-3.26946300	-2.44327200

Atomic coordinates for reactants and transition states for DTBM-SEGPHOS system.

(a) [{(R)-DTBM-SEGPHOS}Ni(α-azidoacetyl-1,3-oxazolidine-2-thione)]$^+$ (**R1**)

Ni	-0.05689800	-0.42395500	-1.33184800
S	-1.46243400	-0.81282700	-3.06212100
C	-0.76538700	-2.14258000	-3.86544700
O	-1.45497500	-2.70346400	-4.84904000
C	-0.64904900	-3.75527000	-5.46447200
C	0.51053700	-3.96484700	-4.48469000
N	0.39485800	-2.75792400	-3.64022300
C	1.37390300	-2.33029200	-2.68542200
O	1.37230200	-1.06687400	-2.37838900
H	-0.32387900	-3.38815000	-6.43636000
H	-1.28884100	-4.62419000	-5.58579000
H	1.47981600	-3.98453400	-4.97903900
H	0.39377400	-4.86430900	-3.87820400
C	2.27070100	-3.24561200	-2.23701600
H	2.28396700	-4.26301800	-2.60384500
N	3.23151000	-2.85790100	-1.30447000
N	4.13570900	-3.66292100	-1.05004500
N	5.01258800	-4.29525800	-0.72812500
P	-1.79198400	-0.14165200	0.09157600
P	1.58620200	0.51826700	-0.09617300
C	-1.32819700	-0.37523600	1.84554400
C	-3.04209700	-1.41997000	-0.30457800
C	-2.68942100	1.44025700	0.16230200
C	1.04420700	1.98776500	0.84619600
C	2.89978100	1.08591100	-1.24160700
C	2.38805900	-0.48670300	1.19081700
C	0.03691400	1.87249100	1.95052000
C	1.57217200	3.26316100	0.56102800
C	-0.39091200	0.57323400	2.53323700
C	-0.05069600	0.27119300	3.86145000
C	1.11696200	4.38799400	1.25465000
C	0.14536300	4.23604200	2.24033000
C	-0.35814300	3.06972200	2.56958000
C	-0.54990100	-0.75825100	4.50324800
C	-1.46932200	-1.63189800	3.92856700
O	-0.33589700	5.21481500	2.95818000
O	-1.22638500	3.16469300	3.54328100
O	0.76028100	0.97314900	4.60971600
O	-0.11949200	-0.84342800	5.73330700
H	2.33154000	3.40814400	-0.19041300
H	1.51694400	5.36772000	1.02714400
H	-1.87386000	-2.45556600	4.50266100
H	-2.59492000	-2.11504400	2.19167300
H	-4.58639000	-0.04549600	-0.83051100
H	-1.61838000	-2.97412300	-0.06509500
H	-1.38609000	2.35457200	-1.21804900
H	-3.99054600	0.73858000	1.69402500
H	4.47108500	-0.09070100	-0.38284500
H	1.46592900	2.07329800	-2.44531500
H	3.66071200	1.10949700	1.79214800
H	1.09452600	-2.11877700	0.84918200
H	1.78634700	-0.07842300	6.10406700
H	0.40057800	0.95142100	6.66992200
H	-0.95614600	4.73722300	4.89603600
H	-2.27895300	4.97401600	3.67364700
C	-1.86796500	-1.43448500	2.60336200
C	-2.63935300	-2.75254700	-0.34116100
C	0.76212000	0.27758800	5.86417400
C	-1.25924400	4.56795800	3.84081100
C	-3.52673200	-3.78581800	-0.70224200
C	-4.88843300	-3.43318800	-0.93422000
C	-5.25961200	-2.06900700	-1.11604700

C	-4.32050100	-1.08429600	-0.75144500
C	-2.26044100	2.51886900	-0.60747600
C	-2.94129000	3.75914400	-0.60149000
C	-4.13832200	3.84569400	0.16399100
C	-4.45865100	2.77927600	1.17557200
C	-3.74391400	1.58079500	1.06257600
C	4.22413900	0.65441600	-1.11943800
C	5.22384700	1.11058700	-1.99320400
C	4.83240500	2.19498100	-2.95113900
C	3.46246500	2.40413200	-3.24452700
C	2.52007300	1.86588500	-2.34010300
C	1.92856900	-1.77902200	1.44386900
C	2.54127200	-2.61505400	2.40635800
C	3.68941700	-2.10947900	3.07371300
C	4.00972600	-0.64330100	3.00301300
C	3.38446800	0.08243500	1.98188000
O	4.61633500	-3.00452000	3.62020900
O	-5.07208400	4.84415000	-0.12860200
O	5.78792300	3.09163400	-3.43898100
O	-5.88398300	-4.40327600	-0.78986700
C	6.19757600	4.04780200	-2.45887700
H	6.93573100	4.73748300	-2.91642200
H	6.67521400	3.55179400	-1.58975100
H	5.32983800	4.64512800	-2.10438200
C	-6.13607500	4.37943000	-0.96455700
H	-6.81852100	5.22617400	-1.18175100
H	-6.71954100	3.58102500	-0.46788100
H	-5.74515300	3.98569800	-1.92654200
C	5.76935600	-3.21787300	2.79287500
H	5.74854200	-2.63261600	1.84458600
H	5.84222600	-4.29181200	2.52918800
H	6.68616800	-2.95096600	3.35522500
C	3.01955700	3.21175400	-4.49361300
C	6.59532500	0.39617900	-2.00895400
C	6.37831800	-1.11380600	-2.26210000
H	7.34856900	-1.64750200	-2.35971700
H	5.79502900	-1.27214300	-3.19516400
H	5.83600800	-1.59027500	-1.42208100
C	7.54373700	0.90600400	-3.11974300
H	7.90701100	1.93126300	-2.90836100
H	7.04284600	0.87964000	-4.11152500
H	8.45185200	0.26808600	-3.18632500
C	7.29841600	0.57861900	-0.64715900
H	7.41997800	1.65496400	-0.40657400
H	8.30594300	0.10916500	-0.66000400
H	6.72040500	0.10322000	0.17210400
C	3.32225300	4.71351300	-4.30604200
H	2.83729000	5.31612200	-5.10458400
H	4.40634700	4.92887900	-4.36805400
H	2.93876500	5.06623200	-3.32412400
C	3.75783600	2.68450800	-5.74699200
H	4.85207300	2.84981200	-5.68858600
H	3.40362300	3.20443100	-6.66358800
H	3.57662700	1.59477500	-5.87138800
C	1.50326500	3.07479300	-4.78355000
H	1.20612100	2.00524300	-4.84718600
H	0.90486300	3.58000900	-3.99925200
H	1.23502400	3.55814000	-5.74827400
C	1.97472500	-4.02401900	2.73609200
C	4.96387900	0.08546800	3.97909500
C	0.66006300	-4.33676300	1.97882300
H	0.83426400	-4.38271600	0.88181800
H	-0.11584600	-3.57565600	2.19793000
H	0.24940600	-5.32236900	2.28877000
C	2.98027700	-5.13848800	2.36951400
H	2.48473900	-6.13340100	2.34400400
H	3.78930600	-5.23162900	3.11894700

H	3.42048600	-4.95105600	1.36968300
C	1.65972900	-4.09837400	4.24641500
H	2.57248400	-3.95598800	4.86149900
H	1.22819200	-5.08775800	4.51240500
H	0.92906800	-3.31173900	4.52886600
C	6.29283000	0.38844800	3.26168500
H	6.76840700	-0.54385600	2.89562200
H	7.00408300	0.89321600	3.95077400
H	6.12596800	1.05669600	2.38999500
C	5.24995400	-0.73737200	5.25928900
H	5.74995200	-0.11386400	6.03185000
H	5.94214100	-1.57833100	5.06896100
H	4.30421200	-1.12576400	5.69535600
C	4.34420400	1.42827800	4.44712200
H	3.34189900	1.26202900	4.88556600
H	4.25046500	2.16233900	3.62133700
H	4.98034000	1.91397500	5.21858200
C	-2.97503500	-5.23304600	-0.79929700
C	-6.62886400	-1.60153700	-1.68100200
C	-3.93378800	-6.22551500	-1.50698200
H	-4.78111800	-6.52164200	-0.85807200
H	-3.41423400	-7.17663800	-1.75438800
H	-4.31704600	-5.79339100	-2.45656600
C	-2.68440900	-5.75665900	0.61925900
H	-2.25410300	-6.78080400	0.58094700
H	-3.61776900	-5.78985700	1.22031000
H	-1.96513100	-5.09722900	1.14519600
C	-7.49637200	-2.74254700	-2.27089900
H	-6.90231000	-3.36892600	-2.97088400
H	-8.36133900	-2.33417900	-2.83745900
H	-7.94203100	-3.37540800	-1.47869000
C	-7.42947400	-0.91143800	-0.55913800
H	-8.42544000	-0.58506400	-0.92948200
H	-6.89643100	-0.01405900	-0.18148100
H	-7.58196800	-1.60890700	0.29249400
C	-6.39912300	-0.59136000	-2.83654000
H	-7.35869900	-0.31471200	-3.32485600
H	-5.73113200	-1.03321300	-3.60744700
H	-5.95148600	0.36061000	-2.48612300
C	-5.45447900	2.94129200	2.35164500
C	-2.39088200	4.98541600	-1.38377100
C	-6.63565400	1.96285900	2.17727300
H	-7.36690400	2.08049300	3.00622600
H	-6.28949000	0.90774500	2.18125100
H	-7.16890300	2.14557700	1.22346200
C	-4.74448300	2.61386300	3.68885800
H	-4.38814900	1.56433700	3.73221700
H	-5.43459100	2.75136200	4.54955700
H	-3.87639600	3.28316600	3.83814600
C	-6.01621300	4.37609600	2.48839800
H	-6.73700600	4.61562300	1.68264600
H	-5.19310000	5.12298600	2.49344900
H	-6.57957700	4.49085700	3.43997700
C	-3.34182300	5.39528800	-2.53061700
H	-2.84544300	6.11042500	-3.22228600
H	-4.24387200	5.91832000	-2.15964600
H	-3.64512400	4.50377100	-3.12042800
C	-2.21410100	6.17254800	-0.41102400
H	-3.18077800	6.49193000	0.02904600
H	-1.78241000	7.05274400	-0.93515500
H	-1.53248500	5.89255700	0.41997000
C	-1.00698900	4.71574800	-2.02601100
H	-1.08933600	3.93298100	-2.80959200
H	-0.26675700	4.40002500	-1.26319000
H	-0.60601400	5.63032000	-2.51505400
C	-1.66151200	-5.24080700	-1.62593700
H	-1.29818500	-6.27736300	-1.79614400

H	-0.83234900	-4.71036400	-1.11644500
H	-1.83213800	-4.76614300	-2.61580300
C	-6.33154100	-4.56783300	0.56233200
H	-5.80271600	-3.90662900	1.28722900
H	-6.17695600	-5.62037500	0.87467200
H	-7.41674400	-4.34824300	0.62035500

(b) [{(R)-DTBM-SEGPHOS}Ni(α-azidoacetyl-1,3-oxazolidine-2-thione)]$^+$ (**R2**)

Ni	-0.07276300	-0.38774700	-1.29089300
S	-1.53045700	-0.67043800	-2.99809600
C	-0.87289100	-1.96895700	-3.88348800
O	-1.56254100	-2.42658000	-4.91722200
C	-0.90479800	-3.61225000	-5.45985400
C	0.41763300	-3.71350300	-4.68438800
N	0.25725400	-2.64209700	-3.67882600
C	1.26714500	-2.26283500	-2.73081700
O	1.31547000	-1.00541000	-2.40277800
H	-0.77634600	-3.45438800	-6.52706100
H	-1.56945900	-4.45539700	-5.28462900
H	1.29491500	-3.50739800	-5.29740200
H	0.53429500	-4.68283800	-4.20220900
C	2.13757800	-3.21542300	-2.31045200
H	2.10567300	-4.22863800	-2.68746700
N	3.12695200	-2.87359300	-1.39001700
N	4.01470000	-3.70767800	-1.17295000
N	4.88097900	-4.36894900	-0.88275200
P	-1.77294400	-0.16753900	0.16661900
P	1.58990000	0.55166700	-0.07724100
C	-1.27696600	-0.36434700	1.91393700
C	-2.97480800	-1.49647200	-0.20687600
C	-2.71566400	1.38671100	0.21451700
C	1.04933100	2.02612900	0.85916100
C	2.87635500	1.11249700	-1.25354600
C	2.43261000	-0.42652600	1.20338800
C	0.03535200	1.91435600	1.95732300
C	1.56611500	3.30384400	0.56324000
C	-0.35764100	0.62003200	2.57262400
C	0.01101000	0.34894700	3.89955100
C	1.08148500	4.43376300	1.22883600
C	0.09311100	4.28437300	2.19847400
C	-0.39345600	3.11566400	2.54421900
C	-0.44504900	-0.68726900	4.56246000
C	-1.34248000	-1.59968100	4.01237300
O	-0.41967900	5.26836000	2.88675500
O	-1.27996900	3.21358300	3.50102000
O	0.81159000	1.08610200	4.62499500
O	0.00736200	-0.74204900	5.78639800
H	2.33721600	3.44718000	-0.17621200
H	1.47004500	5.41554600	0.99059600
H	-1.70892400	-2.42990600	4.60234300
H	-2.47233100	-2.14572800	2.29370900
H	-4.58981400	-0.19078300	-0.69153000
H	-1.47772800	-2.98412000	0.00293000
H	-1.44188300	2.30583600	-1.19188900
H	-3.98482000	0.68772400	1.77403100
H	4.43740900	-0.11975400	-0.46570600
H	1.43611900	2.20495700	-2.36571500
H	3.70134300	1.19509400	1.74202200
H	1.15908400	-2.08427300	0.91516400
H	1.89309500	0.08888000	6.11757800
H	0.48359600	1.08152100	6.69074100
H	-1.06779700	4.82108200	4.82290200
H	-2.37108000	5.00316500	3.57028800
C	-1.76689500	-1.43349500	2.69047400
C	-2.51015500	-2.80791100	-0.26274000
C	0.85445300	0.40844300	5.88855800

C	-1.34677100	4.62217900	3.76618400
C	-3.34729500	-3.87605300	-0.64152900
C	-4.72639800	-3.58663900	-0.85802000
C	-5.16659700	-2.23896100	-1.00750200
C	-4.27319100	-1.21618100	-0.63294200
C	-2.31893700	2.45823400	-0.58189700
C	-3.03520600	3.67660100	-0.60488400
C	-4.23675100	3.74345200	0.15430900
C	-4.52174500	2.69681100	1.19665000
C	-3.76825800	1.51913200	1.11760700
C	4.18463000	0.62660300	-1.19932600
C	5.15922900	1.03860700	-2.12264200
C	4.76383100	2.11802400	-3.08790200
C	3.38854300	2.42131100	-3.27446400
C	2.48055400	1.92903300	-2.31511200
C	2.00176700	-1.72272000	1.48427400
C	2.65327800	-2.53457900	2.44251700
C	3.80702500	-1.99940600	3.07651300
C	4.10161100	-0.53024500	2.97504100
C	3.44319100	0.16807600	1.95637000
O	4.76029700	-2.86942000	3.61796500
O	-5.20654200	4.69489200	-0.17523700
O	5.74550800	2.94727800	-3.63455000
O	-5.67098300	-4.60917400	-0.73504500
C	6.12671400	4.00492800	-2.75461500
H	6.90349400	4.62192400	-3.25041700
H	6.54979500	3.60134500	-1.81066500
H	5.25788000	4.65596800	-2.52199300
C	-6.24560900	4.16206600	-1.00154000
H	-6.95911700	4.97391200	-1.24975300
H	-6.80064800	3.35768600	-0.48186100
H	-5.83245400	3.75373400	-1.94824400
C	5.89906900	-3.07774600	2.76986000
H	5.84626000	-2.51289200	1.81040600
H	5.98688300	-4.15539000	2.52657900
H	6.82242100	-2.78202700	3.30650800
C	2.83145200	3.28009800	-4.44055300
C	6.52843900	0.31807300	-2.13655200
C	6.30470000	-1.20203900	-2.31531400
H	7.27190800	-1.74051300	-2.41639800
H	5.69577100	-1.40113900	-3.22381500
H	5.78775000	-1.64261400	-1.44003200
C	7.45818000	0.77428000	-3.28546100
H	7.83399100	1.80438700	-3.11993000
H	6.93720000	0.71194700	-4.26517200
H	8.36034100	0.12720500	-3.34411600
C	7.25833300	0.56455000	-0.79903000
H	7.38923100	1.65240500	-0.61943400
H	8.26293900	0.08874000	-0.80641100
H	6.69321300	0.13728900	0.05534500
C	2.27635500	4.60673000	-3.88377600
H	1.84913600	5.22819600	-4.70045700
H	3.08005400	5.18924500	-3.38705300
H	1.47623700	4.42636500	-3.13913800
C	3.87459200	3.61603400	-5.53551200
H	4.60725700	4.37236000	-5.18608200
H	3.38440700	4.06594400	-6.42619800
H	4.40471300	2.69860600	-5.87104200
C	1.68671700	2.50699000	-5.14472700
H	2.04415700	1.50882600	-5.47895800
H	0.80822100	2.36131800	-4.48345000
H	1.31798700	3.06086600	-6.03525700
C	2.12232000	-3.94911700	2.80420700
C	5.05989000	0.23131200	3.92097800
C	0.80391600	-4.30246300	2.07257500
H	0.96281200	-4.35885600	0.97381400
H	0.01332800	-3.55718600	2.29393900

H	0.42163600	-5.29297700	2.40246900
C	3.14688800	-5.04767700	2.44071700
H	2.67078900	-6.05216900	2.42311700
H	3.96036000	-5.12081600	3.18793700
H	3.58103700	-4.85765900	1.43858700
C	1.83179200	-4.00359100	4.32020300
H	2.75035100	-3.82928800	4.91829400
H	1.42705100	-4.99760100	4.60992000
H	1.08776300	-3.22851300	4.60012700
C	6.36937500	0.54707300	3.17364700
H	6.85818600	-0.38283000	2.81894600
H	7.08145200	1.08115200	3.83939600
H	6.17371100	1.19230800	2.29054600
C	5.38519200	-0.56530000	5.20832200
H	5.88975100	0.07924900	5.96035800
H	6.08764000	-1.39769400	5.01774000
H	4.45488100	-0.96200800	5.66948300
C	4.42199800	1.56909500	4.37916100
H	3.42722500	1.39051500	4.83089400
H	4.30546300	2.28993500	3.54456800
H	5.05816600	2.07658100	5.13642100
C	-2.72564000	-5.29194600	-0.77653700
C	-6.56260600	-1.82805300	-1.55088800
C	-3.63156200	-6.30544600	-1.52323400
H	-4.46747800	-6.66344900	-0.89090200
H	-3.06596500	-7.22216600	-1.79796800
H	-4.02991100	-5.86061200	-2.46067300
C	-2.42231200	-5.84333500	0.62843600
H	-1.93608200	-6.84081800	0.56414300
H	-3.35813900	-5.94613500	1.21724700
H	-1.74664300	-5.16148500	1.18247400
C	-7.37723000	-2.99721900	-2.16068800
H	-6.75893300	-3.57640500	-2.88006400
H	-8.26677700	-2.61990000	-2.71040800
H	-7.78353900	-3.66983300	-1.38017300
C	-7.38637900	-1.20555400	-0.40657200
H	-8.39972600	-0.91837400	-0.76186800
H	-6.89380300	-0.29349200	-0.00958900
H	-7.49843800	-1.93074600	0.42803000
C	-6.39479000	-0.78084700	-2.68420100
H	-7.37086900	-0.54478200	-3.16089400
H	-5.70917700	-1.16856500	-3.46866900
H	-5.99694400	0.18586100	-2.31453300
C	-5.52221300	2.86142300	2.36747300
C	-2.52074800	4.89867200	-1.41580000
C	-6.66678100	1.83525200	2.22683000
H	-7.40473000	1.95687300	3.04921300
H	-6.28302300	0.79417900	2.27073600
H	-7.20228000	1.96362100	1.26520900
C	-4.79960300	2.60567300	3.71331400
H	-4.40210000	1.57285700	3.78912200
H	-5.49463200	2.74366800	4.56993400
H	-3.95808900	3.31313900	3.83974200
C	-6.13662900	4.27821100	2.45587000
H	-6.86366500	4.46357700	1.64131300
H	-5.34165000	5.05476300	2.43666200
H	-6.70572600	4.40350300	3.40265200
C	-3.46573100	5.22867700	-2.59211400
H	-2.98860000	5.94523400	-3.29570700
H	-4.39900100	5.71768200	-2.25321900
H	-3.71585400	4.30630400	-3.15912500
C	-2.41315400	6.12316600	-0.47890900
H	-3.40086400	6.41961300	-0.07120100
H	-2.00437800	7.00270500	-1.02213500
H	-1.74019300	5.89702500	0.37583900
C	-1.11187600	4.66995200	-2.01987200
H	-1.13210000	3.87057100	-2.79225100

H	-0.38087500	4.40069100	-1.23032900
H	-0.73744300	5.59190700	-2.51606100
C	-1.40538600	-5.21485900	-1.59013000
H	-0.99724300	-6.22826300	-1.79469100
H	-0.60330700	-4.67112200	-1.05243300
H	-1.58580000	-4.71117400	-2.56343600
C	-6.09758600	-4.83629800	0.61473600
H	-5.59159100	-4.17540100	1.35599800
H	-5.89357200	-5.89039600	0.89143300
H	-7.19086000	-4.66746800	0.68874500

(*c*) [{(*R*)-DTBM-SEGPHOS}Ni(α-azidoacetyl-1,3-oxazolidine-2-thione)]$^+$ (**R3**)

Ni	-0.18915300	-0.18864300	-1.38520800
S	1.11210200	-0.61567500	-3.14861800
C	0.04584500	-0.93954800	-4.44121700
O	0.60031900	-1.46769300	-5.52425500
C	-0.39356100	-1.53482000	-6.58821400
C	-1.72137500	-1.30309300	-5.86735100
N	-1.26437300	-0.75753500	-4.56639600
C	-2.17289400	-0.29681900	-3.55222900
O	-1.68703800	0.15181900	-2.43705100
H	-0.30603400	-2.51010800	-7.05780100
H	-0.15559100	-0.75024600	-7.30479600
H	-2.28775800	-2.22053600	-5.70318700
H	-2.34478100	-0.57696000	-6.38390000
C	-3.49438000	-0.30446200	-3.86877200
H	-3.84261300	-0.58980600	-4.84946400
N	-4.44673700	0.10897400	-2.93452900
N	-5.62535900	0.06171000	-3.30129200
N	-6.73214000	0.05763300	-3.52545900
P	1.66511600	0.28913400	-0.20942800
P	-1.54719100	-0.32950100	0.48062400
C	1.20852200	1.10128700	1.39236700
C	2.68291700	1.47349600	-1.16794100
C	2.74735600	-1.10420500	0.24285800
C	-0.88044100	-1.44684200	1.77862200
C	-3.17570000	-1.03306600	0.03925600
C	-1.94964900	1.25200400	1.29835200
C	0.44875500	-1.21331400	2.45090500
C	-1.53324000	-2.67435000	2.03312600
C	1.12542700	0.12036800	2.56189100
C	1.72479100	0.50098700	3.77888300
C	-0.89666200	-3.68363700	2.75909300
C	0.39710300	-3.47467600	3.21935400
C	1.02603100	-2.33752800	3.07325200
C	2.40688100	1.60728900	3.94057200
C	2.59956400	2.54598300	2.93899300
O	1.16032800	-4.40815500	3.89118900
O	2.29489600	-2.39982600	3.61401100
O	1.64330900	-0.20578300	4.96632300
O	2.87265900	1.74454600	5.23306000
H	-2.51152000	-2.89318500	1.63668700
H	-1.38734600	-4.63461100	2.92091800
H	3.15436400	3.45261900	3.14242500
H	2.09344300	3.13843900	0.96255200
H	4.40160700	0.21627700	-1.29974200
H	1.04893000	2.81133200	-1.37746700
H	1.41865000	-2.48634600	-0.63352600
H	4.09995800	0.11057200	1.35479600
H	-4.30893400	0.33337800	1.23533000
H	-2.27103900	-2.41944900	-1.26699000
H	-1.87699000	0.46702100	3.28639200
H	-1.97558800	2.24814700	-0.55974300
H	1.73094100	0.87599500	6.75002800
H	3.24512600	-0.01666000	6.29258100
H	2.56742900	-3.66978300	5.24884200

H	3.23670100	-4.26237200	3.67139700
C	2.02444900	2.34403900	1.68997600
C	2.08486600	2.64016300	-1.63588800
C	2.38908900	0.57662100	5.90730400
C	2.39365300	-3.72454500	4.15361900
C	2.80157300	3.58708300	-2.39629100
C	4.19202100	3.35146700	-2.60046700
C	4.75149500	2.06806400	-2.33590700
C	3.98320400	1.16778600	-1.57174700
C	2.36611300	-2.39191100	-0.12683300
C	3.18636100	-3.51489300	0.11196400
C	4.47142200	-3.27304000	0.67022500
C	4.76475800	-1.94736700	1.32164300
C	3.89597900	-0.89188300	1.00470100
C	-4.35504100	-0.51963600	0.58045600
C	-5.60608500	-1.05455800	0.25074300
C	-5.60198500	-2.35098100	-0.51256200
C	-4.41897500	-2.77344500	-1.18064500
C	-3.21763400	-2.10231100	-0.85811900
C	-2.16670500	2.37113700	0.49456400
C	-2.67790800	3.58281300	1.01123300
C	-3.01871200	3.61041000	2.38929500
C	-2.53087800	2.51888900	3.29867400
C	-2.10999700	1.33050200	2.68258000
O	-3.92036800	4.57093100	2.85832800
O	5.49999500	-4.19281300	0.44323500
O	-6.70210900	-3.21204000	-0.44825700
O	5.04101000	4.43002800	-2.86324500
C	-6.79257900	-3.90743700	0.79840700
H	-7.65960000	-4.59799000	0.76235300
H	-6.94197900	-3.20642300	1.64112900
H	-5.87476700	-4.50289100	0.99034100
C	6.34769200	-3.81728900	-0.64605600
H	7.11331200	-4.60578200	-0.79412800
H	6.86754300	-2.86182000	-0.44054200
H	5.76526100	-3.70938300	-1.58592400
C	-5.24649400	4.05859600	3.01467500
H	-5.27650900	3.23450500	3.75356900
H	-5.64408400	3.68622000	2.04745000
H	-5.90647100	4.87398600	3.37429000
C	-4.43619900	-3.93552700	-2.21573700
C	-6.87407600	-0.23910900	0.60783600
C	-6.74132900	1.20106700	0.04972000
H	-7.65322100	1.79810300	0.26846300
H	-6.59792600	1.18374500	-1.04838100
H	-5.88724600	1.74746700	0.49890000
C	-8.17150000	-0.83186200	0.01008300
H	-8.47110100	-1.76687100	0.52226500
H	-8.05462400	-1.02091500	-1.07857200
H	-9.02281800	-0.12868800	0.13958300
C	-7.03177900	-0.15160600	2.14180200
H	-7.09595100	-1.16014200	2.59599700
H	-7.95677600	0.40447300	2.40820900
H	-6.17618100	0.38082000	2.60849800
C	-4.76134600	-5.28151300	-1.53160500
H	-4.54374700	-6.13519900	-2.20986700
H	-5.83428700	-5.36861900	-1.27429100
H	-4.14885900	-5.40814200	-0.61295600
C	-5.49160100	-3.64460500	-3.30689800
H	-6.51425800	-3.56461000	-2.88702000
H	-5.51081000	-4.45892100	-4.06346400
H	-5.25634700	-2.69430500	-3.82991400
C	-3.07803200	-4.11588000	-2.94451500
H	-2.76102800	-3.17148400	-3.43299600
H	-2.28659300	-4.45103600	-2.23931700
H	-3.15175900	-4.88942000	-3.73984800
C	-2.86948300	4.83010700	0.10401900

C	-2.36779700	2.66750200	4.82996100
C	-2.27849000	4.63417500	-1.31448600
H	-2.84323800	3.86252300	-1.88153600
H	-1.21361300	4.33664200	-1.24847300
H	-2.32763900	5.57523000	-1.90475400
C	-4.36647700	5.16705500	-0.07302500
H	-4.51305600	5.90151400	-0.89473700
H	-4.79819900	5.63669700	0.83145300
H	-4.94346400	4.25076500	-0.32367100
C	-2.13476200	6.03681200	0.73143700
H	-2.55807800	6.31179200	1.71863800
H	-2.21659800	6.93305700	0.07877200
H	-1.05664100	5.80265700	0.86958100
C	-3.28354600	1.66150000	5.56002300
H	-4.34698100	1.82466400	5.29292600
H	-3.18414700	1.77166100	6.66173200
H	-3.02254700	0.61351400	5.30303800
C	-2.68703800	4.08930600	5.34955500
H	-2.39831400	4.19321000	6.41812800
H	-3.77219700	4.30779100	5.30814300
H	-2.12503000	4.85626400	4.77409700
C	-0.89914100	2.37176000	5.21355700
H	-0.20769200	3.02051700	4.63322900
H	-0.63858500	1.31239400	5.02029300
H	-0.72123700	2.55047500	6.29615400
C	2.03498300	4.81755000	-2.94864600
C	6.15274200	1.61080700	-2.82463800
C	2.84878000	5.68736000	-3.94040900
H	3.62174600	6.29096200	-3.42545100
H	2.19241600	6.42520100	-4.45134800
H	3.31939300	5.05714200	-4.72569400
C	1.59741700	5.71250000	-1.77122800
H	1.01239100	6.58482900	-2.13505800
H	2.48784000	6.09045800	-1.22412800
H	0.96892900	5.15574800	-1.04733000
C	6.79515800	2.55549300	-3.87282300
H	6.07215500	2.79725400	-4.68155300
H	7.68358700	2.08317300	-4.34548700
H	7.17501500	3.48876900	-3.41297000
C	7.10018200	1.50572900	-1.61468600
H	8.11619900	1.18953300	-1.93613600
H	6.72595900	0.76151700	-0.88230700
H	7.18312900	2.48685100	-1.10003600
C	6.04282700	0.21977700	-3.50493300
H	7.01140700	-0.08360200	-3.95827300
H	5.27498600	0.24304600	-4.30839200
H	5.77997700	-0.58441200	-2.78802700
C	5.91022400	-1.69868300	2.33502400
C	2.71377300	-4.94574600	-0.27105000
C	6.90071500	-0.66230100	1.76255200
H	7.74948500	-0.50242200	2.46239900
H	6.40958300	0.32058100	1.60329700
H	7.31772400	-1.00314500	0.79409000
C	5.33321500	-1.14211800	3.65956200
H	4.74815000	-0.21315300	3.50572100
H	6.15035700	-0.89507900	4.37175000
H	4.68442800	-1.89450600	4.14952800
C	6.69687300	-2.97973700	2.69925700
H	7.33554500	-3.32292900	1.86188900
H	6.00536100	-3.79479200	3.00399400
H	7.38643100	-2.78937100	3.55047100
C	3.48071200	-5.46284300	-1.50622400
H	3.00153600	-6.37940500	-1.91417800
H	4.52184000	-5.74512000	-1.25699500
H	3.49093300	-4.69288500	-2.30763000
C	2.93859800	-5.91231900	0.91401800
H	4.00613400	-5.98404600	1.20187700

H	2.60255200	-6.93952500	0.65349600
H	2.35972100	-5.57717200	1.79815400
C	1.19905600	-5.00521700	-0.60747200
H	0.97483200	-4.46738200	-1.55403000
H	0.59303000	-4.57061100	0.21697800
H	0.86349200	-6.05545400	-0.75146600
C	0.78572900	4.33633600	-3.73158300
H	0.21916000	5.19770900	-4.14749100
H	0.07628300	3.76854100	-3.10121900
H	1.09132100	3.67983800	-4.57508700
C	5.53386900	5.07127400	-1.67907400
H	5.16483400	4.60464900	-0.73646300
H	5.22596300	6.13636700	-1.68199000
H	6.64196200	5.03356600	-1.67457700

(*d*) [{(*R*)-DTBM-SEGPHOS}Ni(α-azidoacetyl-1,3-oxazolidine-2-thione)]$^+$ (**R4**)

Ni	0.03943600	-0.05251200	-1.30738000
S	1.26127900	-0.72660000	-3.07284300
C	0.05725300	-1.10292100	-4.22808800
O	0.41300900	-1.93387000	-5.19825600
C	-0.73765000	-2.22548200	-6.04673300
C	-1.80260000	-1.22372200	-5.59291700
N	-1.20588100	-0.69121700	-4.34846700
C	-1.89857100	0.21034900	-3.48641400
O	-1.33194500	0.56418700	-2.37507100
H	-1.01677800	-3.26267900	-5.87010100
H	-0.42382600	-2.09741900	-7.07862900
H	-2.76235300	-1.69017800	-5.37944400
H	-1.94417300	-0.41125200	-6.30646700
C	-3.09279000	0.71931200	-3.89198900
H	-3.50714600	0.52514400	-4.87032200
N	-3.75761900	1.59933400	-3.03592400
N	-4.70275300	2.24780600	-3.49364500
N	-5.58529200	2.89428800	-3.77409200
P	1.85643000	0.30323200	-0.05140800
P	-1.50770200	-0.31714400	0.29340800
C	1.48618100	1.34054400	1.40877600
C	3.02647600	1.24941500	-1.09286200
C	2.80404600	-1.06477900	0.68160900
C	-0.87355400	-1.19904500	1.76076700
C	-2.83248800	-1.36491800	-0.41219800
C	-2.29731000	1.14836500	1.02061000
C	0.17959100	-0.58672000	2.63429200
C	-1.36691100	-2.47257900	2.10727300
C	0.61288400	0.83112300	2.51745100
C	0.33084600	1.72742600	3.56054900
C	-0.84766600	-3.15380300	3.21215700
C	0.15649600	-2.55879300	3.97236700
C	0.63624700	-1.36556000	3.70974300
C	0.82283400	2.94313700	3.60131300
C	1.67903700	3.44383300	2.62413700
O	0.69939100	-3.09965900	5.02959400
O	1.54933300	-1.00007800	4.57220200
O	-0.41581000	1.46240300	4.60086900
O	0.45180400	3.60258000	4.66560300
H	-2.14677600	-2.95252900	1.53697000
H	-1.22221700	-4.13532000	3.47279500
H	2.08006200	4.44561100	2.70962800
H	2.70427400	3.04499100	0.80577000
H	4.59327600	-0.19477800	-1.01807000
H	1.55723000	2.72313300	-1.50098800
H	1.44905600	-2.51392400	-0.02999900
H	4.17151300	0.26562700	1.62502500
H	-4.44830900	-0.02403800	0.01432300
H	-1.38791600	-2.69010400	-1.20834900
H	-3.44236900	-0.00958000	2.39223800

H	-1.11622700	2.45696200	-0.13688000
H	-1.40230400	3.09267200	5.47452900
H	0.04730300	2.46929100	6.37481500
H	1.38522500	-1.78643200	6.50388400
H	2.66219700	-2.54229700	5.45646100
C	2.02203100	2.63868600	1.53405500
C	2.56820500	2.40725300	-1.71526400
C	-0.37950600	2.67306900	5.36965700
C	1.63831900	-2.11357900	5.47289200
C	3.39290400	3.16797900	-2.56657800
C	4.75226200	2.75898200	-2.70358900
C	5.16627300	1.46827100	-2.26020300
C	4.29003300	0.75679800	-1.41697400
C	2.36394900	-2.37728900	0.52564700
C	3.08528400	-3.47480200	1.05166600
C	4.32888600	-3.19727800	1.68598700
C	4.67378300	-1.78461000	2.07084300
C	3.91540700	-0.77322500	1.46924600
C	-4.17952000	-0.99284100	-0.36872100
C	-5.18506200	-1.83036800	-0.87823700
C	-4.75480100	-3.19228800	-1.33485200
C	-3.38982800	-3.42981700	-1.63107100
C	-2.44856000	-2.51292600	-1.11297000
C	-1.89691600	2.41674000	0.60687900
C	-2.50098800	3.59074000	1.10992500
C	-3.58339600	3.43197600	2.01718600
C	-3.82294300	2.09798100	2.66658400
C	-3.21504000	0.99257700	2.05868800
O	-4.52939800	4.45630100	2.14431600
O	5.27693500	-4.21608700	1.81862100
O	-5.65359600	-4.26314300	-1.32091000
O	5.71559400	3.69397300	-3.09199400
C	-5.90876300	-4.75042800	-0.00148400
H	-6.60935800	-5.60779400	-0.06427400
H	-6.37113900	-3.96735200	0.63300600
H	-4.96894900	-5.09773300	0.47952200
C	6.27308200	-4.18220200	0.79227600
H	6.97060800	-5.03157700	0.94023500
H	6.85663000	-3.24247000	0.82785500
H	5.81254400	-4.27521300	-0.21400300
C	-5.74149700	4.21872200	1.41383700
H	-5.73919400	3.25533700	0.85351900
H	-5.90030600	5.03716600	0.68384800
H	-6.60372600	4.22430600	2.10992000
C	-2.94858300	-4.65922200	-2.46944000
C	-6.61944900	-1.27429600	-1.04248700
C	-6.57436800	0.00019100	-1.91561000
H	-7.59706900	0.39469700	-2.10067500
H	-6.10391600	-0.22320000	-2.89762800
H	-6.00249200	0.81377900	-1.42381700
C	-7.58873200	-2.25883200	-1.73873400
H	-7.83672100	-3.11932000	-1.08637200
H	-7.16283700	-2.62226300	-2.69879900
H	-8.55653400	-1.76244300	-1.96918600
C	-7.20388000	-0.91184700	0.33890700
H	-7.19071000	-1.79049500	1.01639600
H	-8.25487500	-0.56308100	0.24155800
H	-6.62618700	-0.09491000	0.81766000
C	-3.12265000	-5.96308800	-1.66208400
H	-2.62642200	-6.81461100	-2.17657300
H	-4.18771500	-6.24456300	-1.55291800
H	-2.66977900	-5.85691700	-0.65250100
C	-3.78819200	-4.73429100	-3.76650600
H	-4.86262400	-4.90966200	-3.55850900
H	-3.44449600	-5.57031200	-4.41364400
H	-3.69578900	-3.78777900	-4.34104200
C	-1.46444200	-4.57872000	-2.90634000

H	-1.25599700	-3.61467500	-3.41449000
H	-0.78921000	-4.68726400	-2.03445800
H	-1.20861300	-5.39737500	-3.61378400
C	-2.00717800	4.99893800	0.68239000
C	-4.67579500	1.89312800	3.94176600
C	-0.72513500	4.95033200	-0.18778100
H	-0.92563800	4.46296000	-1.16629800
H	0.08967800	4.40799900	0.33545000
H	-0.35311400	5.97544200	-0.40460500
C	-3.08733900	5.73802200	-0.13904700
H	-2.65306900	6.60379600	-0.68478800
H	-3.87956600	6.16096600	0.50770000
H	-3.54597800	5.05425200	-0.88581700
C	-1.66273500	5.82010100	1.94536400
H	-2.55091800	5.97146700	2.59263000
H	-1.27806400	6.82607000	1.67002800
H	-0.88459200	5.29959700	2.54427200
C	-6.02532800	1.25810600	3.55774200
H	-6.56993100	1.89028100	2.82788600
H	-6.66874800	1.13180200	4.45531700
H	-5.87200100	0.25667100	3.10244600
C	-4.93129600	3.21571700	4.70537200
H	-5.34934200	3.01927200	5.71644600
H	-5.67875900	3.85298100	4.19790400
H	-3.98452800	3.78379300	4.83319600
C	-3.95667600	0.94252800	4.93433800
H	-2.94430800	1.32003000	5.17026600
H	-3.86498200	-0.09038800	4.54163500
H	-4.52230400	0.85961700	5.88775000
C	2.78208400	4.41176600	-3.26606300
C	6.51971100	0.80557200	-2.63592700
C	3.65487600	4.97604100	-4.41704200
H	4.53079700	5.53864100	-4.03900700
H	3.08570500	5.70830000	-5.02986300
H	3.99166700	4.15991500	-5.09209600
C	2.56788700	5.52172900	-2.21970300
H	2.10632700	6.41889000	-2.68618800
H	3.53638800	5.82094100	-1.76598300
H	1.90202200	5.17456100	-1.40312800
C	7.30070200	1.54542700	-3.75191500
H	6.63910800	1.76574300	-4.61736300
H	8.14642200	0.92746300	-4.12441000
H	7.76640800	2.47969000	-3.38206400
C	7.41229200	0.73302700	-1.38139500
H	8.39775800	0.28018900	-1.62510900
H	6.93997800	0.11466500	-0.58955000
H	7.58495100	1.75079800	-0.97005900
C	6.26861700	-0.63199000	-3.16430300
H	7.20968800	-1.09429300	-3.53363200
H	5.53839600	-0.61252300	-4.00223100
H	5.88475800	-1.31286400	-2.37787000
C	5.73893200	-1.38666600	3.12276300
C	2.53041200	-4.92468100	0.95888600
C	6.87440200	-0.59081700	2.44417600
H	7.65537400	-0.31421100	3.18530200
H	6.49385200	0.34839000	1.99015200
H	7.35768800	-1.18677900	1.64474300
C	5.09250500	-0.48826000	4.20632000
H	4.70817300	0.46516700	3.78946700
H	5.83138500	-0.21768100	4.99165100
H	4.25278400	-1.01817800	4.69496700
C	6.35639100	-2.59750500	3.86132700
H	7.03707000	-3.17634400	3.20721400
H	5.56110100	-3.26259400	4.26201600
H	6.97746100	-2.26165000	4.72008900
C	3.42550100	-5.81469800	0.06745400
H	2.91333400	-6.76920900	-0.18316600

H	4.36464900	-6.10610100	0.57533000
H	3.66395000	-5.29442700	-0.88499800
C	2.45015500	-5.53013000	2.37786600
H	3.45114600	-5.60800600	2.84924100
H	2.01819600	-6.55399300	2.34735800
H	1.80918700	-4.90004900	3.03074400
C	1.10321300	-4.98452000	0.35895600
H	1.11466800	-4.64969700	-0.69936500
H	0.39845700	-4.35538500	0.93923000
H	0.70512600	-6.02265800	0.37176400
C	1.41425400	4.04249700	-3.90189200
H	1.01046900	4.88393500	-4.50567800
H	0.63893000	3.81983900	-3.14175800
H	1.52346400	3.15833900	-4.56680700
C	6.24082800	4.46216600	-2.00111600
H	5.78585000	4.20242400	-1.01731500
H	6.06419700	5.53988800	-2.19242100

(*e*) Transition state **TS1-A1**, from **R1** and the electrophile in the position **A1**

Ni	0.51931200	0.63922600	-0.77669400
S	1.59295200	1.81501600	-2.34159300
C	0.45685100	2.87004300	-3.03440700
O	0.91735800	3.74148700	-3.89524700
C	-0.16096800	4.63188000	-4.33694200
C	-1.42596600	3.87148000	-3.95092800
N	-0.89283000	2.93696100	-2.91303900
C	-1.68802500	2.00449600	-2.23001700
O	-1.17315700	1.23584800	-1.37148500
H	-0.03702300	4.78684300	-5.40402500
H	-0.03513900	5.57015100	-3.79924700
H	-1.83173700	3.28903800	-4.77921100
H	-2.19315000	4.51211700	-3.52653200
C	-3.09170400	2.01594600	-2.44363700
H	-3.47362200	2.63486600	-3.24500700
N	-3.69691900	0.74586300	-2.27511700
N	-4.77267700	0.58392700	-2.87253000
N	-5.73623100	0.29930600	-3.37910400
P	2.39256700	0.30660500	0.41527600
P	-0.65962200	-1.04799000	0.19498100
C	2.10003300	0.16752700	2.22092200
C	3.50754500	1.74016900	0.18888600
C	3.31327700	-1.17298400	-0.09063600
C	0.28405800	-2.43399700	0.90793500
C	-1.73077900	-1.74076800	-1.11669300
C	-1.70800700	-0.46067400	1.56555700
C	1.10264900	-2.25479100	2.14626800
C	0.17646200	-3.72774000	0.36306700
C	1.29003400	-0.94526100	2.81744600
C	0.90041000	-0.79426500	4.15887600
C	0.78162000	-4.81539200	1.00335600
C	1.47483200	-4.60802500	2.19585300
C	1.62969200	-3.41914300	2.72915000
C	1.28705900	0.21751600	4.89829800
C	2.12270500	1.22209400	4.42178400
O	2.00925400	-5.55710800	2.91818000
O	2.29132600	-3.46557500	3.85754800
O	0.15520600	-1.63573000	4.82693100
O	0.83807500	0.15039200	6.12250900
H	-0.39308200	-3.91185200	-0.53600900
H	0.68058400	-5.81279100	0.59528800
H	2.43881500	2.02609400	5.07401600
H	3.16831600	1.99629100	2.74715300
H	5.17490400	0.60990800	-0.52476300
H	1.92456300	3.07560100	0.65190400
H	2.02309700	-1.48474600	-1.74579000
H	4.62438700	-1.12993500	1.58565700

H	-3.20947900	-2.56131900	0.18645200
H	-0.45621600	-0.94176000	-2.60079300
H	-2.33232100	-2.41038400	2.14021300
H	-0.94968000	1.49522400	1.35727200
H	-0.99170900	-0.81589100	6.37758600
H	0.47563700	-1.75948200	6.89021100
H	2.04848200	-5.21200600	4.97996400
H	3.63887400	-5.04626200	4.13159100
C	2.53468100	1.19429100	3.08694800
C	2.96401600	3.01535100	0.35869400
C	0.06581700	-1.05485500	6.13558000
C	2.54790300	-4.86273300	4.05095500
C	3.71087700	4.19324500	0.16773800
C	5.09396700	4.04909900	-0.12617500
C	5.62700900	2.70686200	-0.54029100
C	4.80948700	1.59379200	-0.28914800
C	2.91899200	-1.83427000	-1.25164200
C	3.67810400	-2.87780400	-1.81717000
C	4.91895800	-3.19082100	-1.19721900
C	5.21144200	-2.67059600	0.18187400
C	4.40595200	-1.61852800	0.64610900
C	-2.90337300	-2.43916600	-0.83017700
C	-3.73241200	-2.93174000	-1.84719200
C	-3.19243300	-2.86249700	-3.24751400
C	-2.10238900	-2.00147100	-3.53812700
C	-1.36048000	-1.50769200	-2.44390600
C	-1.62329300	0.88049800	1.93311400
C	-2.35431900	1.41166800	3.01806400
C	-3.31115600	0.56138900	3.63828100
C	-3.24325300	-0.92675300	3.41043600
C	-2.43362900	-1.35776000	2.34754700
O	-4.34706300	1.12120300	4.39253800
O	5.95157000	-3.75647200	-1.94832200
O	-3.64562000	-3.75162400	-4.22678800
O	5.98585200	5.08310200	0.16602900
C	-3.11618500	-5.06978000	-4.06503500
H	-3.48284900	-5.70842600	-4.89419200
H	-3.44496500	-5.51932100	-3.10830600
H	-2.00524900	-5.05822600	-4.09281600
C	6.78265400	-2.77425800	-2.56853000
H	7.57802000	-3.28890800	-3.14498300
H	7.26546100	-2.12510000	-1.80778200
H	6.19350500	-2.14529700	-3.26880100
C	-5.55541000	1.28443000	3.64544600
H	-5.91732700	0.31718100	3.24448900
H	-5.40815400	1.98620500	2.80131200
H	-6.33674300	1.70221500	4.31222200
C	-1.73196300	-1.62003400	-4.99917200
C	-5.16020000	-3.40385100	-1.47780700
C	-5.88819500	-2.29058700	-0.68166800
H	-6.93290300	-2.58742700	-0.44452800
H	-5.92360000	-1.35248000	-1.26982000
H	-5.39263800	-2.07247000	0.28628100
C	-6.03801600	-3.71701100	-2.71193500
H	-5.71181400	-4.64445500	-3.22274100
H	-6.02707200	-2.86838500	-3.42957100
H	-7.09425100	-3.89007900	-2.41128100
C	-5.08419600	-4.66824100	-0.59561100
H	-4.56607700	-5.49292200	-1.12452300
H	-6.10418400	-5.01944600	-0.32723700
H	-4.53666100	-4.47120600	0.34929100
C	-1.13605500	-2.83071500	-5.74764700
H	-0.67924600	-2.51497200	-6.71075500
H	-1.90934900	-3.57968600	-6.00486200
H	-0.34656000	-3.31503000	-5.13451100
C	-2.99106200	-1.11844200	-5.74438400
H	-3.77135300	-1.90101900	-5.82419300

H	-2.73842700	-0.80742700	-6.78137100
H	-3.42770100	-0.24326200	-5.21798200
C	-0.69099400	-0.47194300	-5.07593200
H	-1.03919000	0.41562900	-4.50722100
H	0.29645100	-0.79677400	-4.68752200
H	-0.52884600	-0.14980700	-6.12764200
C	-2.09045000	2.85552800	3.53157900
C	-3.97474000	-1.99489600	4.26686500
C	-0.85503400	3.51537200	2.86260400
H	-1.03118900	3.69003400	1.78069000
H	0.05057100	2.88463600	2.99081900
H	-0.63629500	4.50632900	3.31691600
C	-3.30221000	3.77267300	3.26158400
H	-3.02905300	4.84245300	3.39137900
H	-4.12965300	3.58280200	3.97243000
H	-3.67005100	3.63555700	2.22282200
C	-1.80105100	2.81648800	5.04990100
H	-2.67075700	2.44889100	5.63056300
H	-1.55913300	3.83168100	5.43295100
H	-0.93828100	2.14998500	5.26085700
C	-5.04062100	-2.69710500	3.39942700
H	-5.76626400	-1.96510900	2.99020600
H	-5.60420700	-3.44580000	3.99730400
H	-4.57223900	-3.22923700	2.54508400
C	-4.66399000	-1.41781100	5.52620800
H	-5.01093500	-2.23375900	6.19701300
H	-5.57299600	-0.83951100	5.27366200
H	-3.95825400	-0.78564800	6.10714300
C	-2.97389400	-3.06575400	4.77136200
H	-2.19273300	-2.60017500	5.40034700
H	-2.48184300	-3.61840500	3.94654500
H	-3.49232700	-3.82989000	5.39056200
C	2.97209300	5.55175700	0.29726300
C	6.96186100	2.49169800	-1.29144200
C	3.84093900	6.78475900	-0.05188900
H	4.60477400	6.98005800	0.72868300
H	3.21902400	7.70500400	-0.10246300
H	4.32982700	6.65724800	-1.04182000
C	2.45509800	5.72540300	1.74156300
H	1.94449800	6.70562000	1.86092800
H	3.29780800	5.68092700	2.46351600
H	1.72605400	4.93406000	2.01263000
C	7.70328000	3.80704400	-1.62609900
H	7.03207400	4.51642400	-2.15655900
H	8.57429500	3.61097400	-2.28865300
H	8.11502800	4.28484000	-0.71377900
C	7.90188200	1.61421500	-0.43767400
H	8.88070700	1.47707100	-0.94638600
H	7.47143600	0.60616000	-0.26420200
H	8.08572100	2.08709600	0.55023600
C	6.68281300	1.77646000	-2.63640400
H	7.61761700	1.65985700	-3.22660400
H	5.95845700	2.36317100	-3.24208200
H	6.26942500	0.75706500	-2.48902400
C	6.29131200	-3.25502100	1.11881800
C	3.13793900	-3.56771800	-3.09809000
C	7.33743700	-2.17152200	1.45731700
H	8.13313300	-2.58689400	2.11307400
H	6.88052800	-1.31204500	1.99054300
H	7.81658500	-1.79078200	0.53118400
C	5.61384100	-3.73543400	2.42039800
H	5.12493700	-2.90214000	2.96679300
H	6.35440800	-4.19121400	3.11282900
H	4.84358700	-4.49838300	2.17875900
C	7.03623300	-4.47332200	0.52410500
H	7.70884500	-4.17364300	-0.30526500
H	6.31783100	-5.24380300	0.16979800

H	7.68655100	-4.94917700	1.29014600
C	3.08912400	-2.54944300	-4.25688400
H	2.69436800	-3.02478700	-5.18111600
H	4.10431900	-2.15642100	-4.47388300
H	2.43340100	-1.68866400	-4.01468800
C	3.97456200	-4.78535800	-3.56266300
H	4.94268300	-4.47213800	-4.00492400
H	3.44656700	-5.34632300	-4.36433300
H	4.15032600	-5.48882600	-2.72032300
C	1.70668000	-4.09373700	-2.82877500
H	0.99232700	-3.27176600	-2.62219300
H	1.70972600	-4.78756300	-1.96100900
H	1.30975700	-4.64391200	-3.70912200
C	1.76743200	5.56983500	-0.67832700
H	1.24233600	6.54915300	-0.64773100
H	1.01138300	4.79862800	-0.42671700
H	2.11241000	5.39202100	-1.71991100
C	6.50684900	5.00086800	1.49267800
H	5.68878300	5.07123400	2.24101700
H	7.20507300	5.84664000	1.65718800
H	7.06497900	4.05195300	1.64010200
C	-3.68780400	3.21213600	-0.85837700
H	-2.98418100	2.80624200	-0.13293900
C	-5.06317600	2.83551300	-0.68353100
C	-6.12566000	3.58626900	-1.23852300
C	-7.42442400	3.17635600	-1.08151000
C	-7.71904200	1.98846700	-0.37550800
C	-6.67300300	1.23503300	0.18790900
C	-5.37000600	1.65884600	0.02686600
H	-5.91098000	4.50324000	-1.77095900
H	-8.24938900	3.74783600	-1.48550500
H	-6.88056000	0.33882100	0.75359700
H	-4.57056000	1.07711100	0.46928200
O	-9.00795500	1.67594600	-0.28683800
C	-9.42280400	0.48675500	0.40742700
H	-9.13304900	0.53338600	1.45867700
H	-10.50493100	0.47036300	0.32600900
H	-9.00330900	-0.40199200	-0.06772000
O	-3.41327500	4.47287100	-1.28560200
C	-2.51061900	5.23734600	-0.46143300
H	-2.94151700	5.40119400	0.52659900
H	-1.54657400	4.73038100	-0.36807300
H	-2.37856700	6.19475000	-0.95816200

(*f*) Transition state **TS2-A2**, from **R2** and the electrophile in the position **A2**

Ni	0.09839200	0.50890700	-0.70481600
S	0.69553500	2.23661500	-1.97891200
C	-0.72071500	3.10341900	-2.33298100
O	-0.56594300	4.22494500	-2.98746400
C	-1.86043800	4.89650800	-3.14864100
C	-2.86731900	3.78145300	-2.89678200
N	-2.03119400	2.81041500	-2.12549700
C	-2.52125000	1.60304100	-1.60965600
O	-1.75124700	0.80640000	-1.00912100
H	-1.88773900	5.31237300	-4.15077500
H	-1.90003900	5.68712500	-2.40188000
H	-3.20488500	3.30402700	-3.81787600
H	-3.71358400	4.11545900	-2.30555300
C	-3.91844800	1.34481200	-1.70278900
H	-4.48846900	1.96299300	-2.38433500
N	-4.24148300	-0.04020000	-1.69610300
N	-5.26106300	-0.34727400	-2.33459900
N	-6.15098400	-0.75832300	-2.88714500
P	2.09756500	0.45861800	0.33811300
P	-0.54361900	-1.58198800	-0.09582400
C	1.97415500	-0.19424900	2.04707200

C	2.71200100	2.18189500	0.46918300
C	3.44071700	-0.50501200	-0.41970400
C	0.79101700	-2.79807700	0.13758100
C	-1.63324200	-2.20473600	-1.43220300
C	-1.47521300	-1.63463800	1.47012100
C	1.73292500	-2.71102700	1.30048500
C	0.89928800	-3.90290200	-0.72784300
C	1.66790300	-1.63907900	2.32877400
C	1.46231600	-1.98636400	3.67378000
C	1.84149400	-4.90535900	-0.47671400
C	2.65948100	-4.80655400	0.64691000
C	2.61707400	-3.78973700	1.47116900
C	1.56955400	-1.12614400	4.65325100
C	1.91593100	0.20538900	4.45352000
O	3.54383800	-5.77765600	1.07028800
O	3.48403300	-3.96806300	2.53394100
O	1.16660100	-3.24958900	4.14671300
O	1.35368500	-1.71190400	5.88335400
H	0.24637100	-4.01224500	-1.58168500
H	1.90814300	-5.76748400	-1.12729200
H	2.01925100	0.87525800	5.29725100
H	2.40362000	1.70333000	3.03377400
H	4.51067700	1.84539000	-0.62925200
H	0.88141100	2.81812900	1.30662200
H	2.23776500	-1.07369700	-2.05796600
H	4.75174200	-0.18634500	1.22839000
H	-2.76196900	-3.53892600	-0.20725400
H	-0.73216100	-0.89016700	-2.83898000
H	-1.50261300	-3.75738800	1.56167700
H	-1.29025900	0.45279700	1.70684700
H	0.07387700	-3.35892000	5.92933100
H	1.84326300	-3.73509000	6.06418300
H	3.77567500	-5.94740600	3.14012200
H	5.16174200	-5.19677400	2.25782200
C	2.12914700	0.66929200	3.15264500
C	1.85138400	3.15566300	0.97479600
C	1.08863200	-3.08643900	5.57167700
C	4.05689700	-5.26648100	2.30916300
C	2.22401400	4.51560100	1.03626400
C	3.55081600	4.85140300	0.63767100
C	4.33862500	3.92010000	-0.10407100
C	3.91591600	2.57740100	-0.11404500
C	3.23436800	-1.13123100	-1.64807600
C	4.28629900	-1.76757300	-2.34699300
C	5.59054200	-1.67096100	-1.79067800
C	5.76110200	-1.24560700	-0.36046400
C	4.66456300	-0.61006900	0.23727600
C	-2.62958600	-3.15083800	-1.19350900
C	-3.50375200	-3.57614500	-2.20305200
C	-3.20841600	-3.10520100	-3.59693200
C	-2.30319400	-2.02687600	-3.79818600
C	-1.49507900	-1.64480000	-2.70565000
C	-1.69546900	-0.44132300	2.15405400
C	-2.40945000	-0.39571100	3.37111900
C	-3.01857600	-1.59958500	3.81541800
C	-2.57611300	-2.91674000	3.23603700
C	-1.82994900	-2.85375300	2.04889100
O	-4.11510700	-1.53326100	4.68377000
O	6.70597400	-1.81177500	-2.62135700
O	-3.65354600	-3.86236000	-4.68253800
O	4.08926500	6.07729300	1.03944000
C	-2.77443500	-4.94193700	-4.99971000
H	-3.18807400	-5.49938100	-5.86459200
H	-2.68227700	-5.64219300	-4.14356000
H	-1.76822000	-4.56217600	-5.27667300
C	7.24624300	-0.55345600	-3.03531200
H	8.11108000	-0.73622100	-3.70499400

H	7.59606600	0.03992800	-2.16742200
H	6.48927700	0.03998900	-3.59118000
C	-5.38286400	-1.69447500	4.03269000
H	-5.29586800	-1.88532800	2.93923000
H	-5.99390500	-0.78155500	4.17550700
H	-5.93569000	-2.53504000	4.49715600
C	-2.14536200	-1.25405800	-5.13745100
C	-4.72123500	-4.44920400	-1.81147100
C	-5.52675700	-3.74952100	-0.68660500
H	-6.43065300	-4.33881800	-0.41956400
H	-5.85637100	-2.74211000	-1.01257000
H	-4.93771600	-3.63421300	0.24562900
C	-5.70451600	-4.69486200	-2.97989000
H	-5.27816600	-5.38875300	-3.73277000
H	-5.98853000	-3.73669300	-3.46581500
H	-6.63780500	-5.17727900	-2.61624500
C	-4.23097700	-5.81898000	-1.29661700
H	-3.64279800	-6.34356200	-2.07803500
H	-5.09233200	-6.46463100	-1.01918400
H	-3.58830900	-5.70998300	-0.39844800
C	-0.72312400	-1.46443400	-5.69528500
H	-0.61782500	-0.99443800	-6.69720700
H	-0.49495200	-2.54689800	-5.79093000
H	0.03542400	-1.00114900	-5.03401700
C	-3.15684900	-1.66480100	-6.23593500
H	-2.92582800	-2.66787800	-6.64931500
H	-3.10900300	-0.96628600	-7.09968000
H	-4.19734600	-1.63971100	-5.84584400
C	-2.36403000	0.25819900	-4.88599500
H	-3.37319200	0.43664900	-4.45856700
H	-1.60687000	0.67979000	-4.19484800
H	-2.28367300	0.83599800	-5.83224600
C	-2.51017600	0.91545100	4.19681100
C	-2.91098400	-4.29633900	3.86150600
C	-1.61353400	2.04900100	3.63591900
H	-1.96807800	2.37748000	2.63632300
H	-0.55541200	1.72013100	3.56198400
H	-1.63775500	2.94156600	4.29846200
C	-3.95920800	1.44761700	4.22002400
H	-3.99181200	2.50169400	4.57185500
H	-4.59613800	0.87669900	4.92144400
H	-4.40357200	1.41144500	3.20375200
C	-2.03425000	0.64454900	5.64238400
H	-2.67808900	-0.09747300	6.15711500
H	-2.05754300	1.57662500	6.24777400
H	-0.99338700	0.25515300	5.63984200
C	-4.08817100	-4.92852600	3.09486600
H	-4.99235900	-4.29207500	3.14686100
H	-4.34417900	-5.92315300	3.52005600
H	-3.82741800	-5.06651100	2.02418900
C	-3.27064800	-4.19068700	5.36457100
H	-3.27404600	-5.19070000	5.84970800
H	-4.28795500	-3.78993000	5.52523800
H	-2.53319100	-3.55464300	5.89977000
C	-1.70525500	-5.26928500	3.76607700
H	-0.79782900	-4.81048800	4.19799900
H	-1.49077900	-5.57950000	2.72323000
H	-1.90629000	-6.21137200	4.32086700
C	1.19625800	5.61088500	1.44119800
C	5.57131500	4.29521600	-0.97495200
C	1.10346500	6.66849200	0.31712000
H	2.06265700	7.20480000	0.17113100
H	0.33400000	7.43441300	0.55687300
H	0.82623200	6.18503400	-0.64518500
C	1.60043000	6.29465100	2.76550000
H	0.76350300	6.90484900	3.16959100
H	2.44536200	6.99659900	2.63048100

H	1.86971200	5.53305200	3.52864600
C	5.86341800	5.81431400	-1.05654400
H	4.95120500	6.37835400	-1.34779300
H	6.64222600	6.02663200	-1.82108200
H	6.27028800	6.21032100	-0.10618300
C	6.83080200	3.58751600	-0.43065900
H	7.72630800	3.86548100	-1.02773500
H	6.72790900	2.48367000	-0.47797200
H	7.01642100	3.87079500	0.62571900
C	5.32448800	3.82519000	-2.43289700
H	6.15378400	4.14192400	-3.10215200
H	4.37786100	4.25886200	-2.82248300
H	5.26301400	2.72081000	-2.51695000
C	7.01007300	-1.54517600	0.50111300
C	4.02997000	-2.54043100	-3.67024900
C	7.66918000	-0.22573600	0.95675500
H	8.56680100	-0.43221100	1.57911500
H	6.97336600	0.38954500	1.56467700
H	7.99010800	0.37756300	0.08376300
C	6.57860300	-2.34652500	1.74999000
H	5.89084100	-1.76895100	2.40188000
H	7.45685100	-2.62651900	2.37134300
H	6.06404900	-3.27456400	1.43230200
C	8.07541900	-2.39753200	-0.22861800
H	8.59280800	-1.82016300	-1.01977600
H	7.61952200	-3.31197700	-0.66587500
H	8.86999500	-2.72378500	0.47729700
C	4.70425900	-1.83710800	-4.86949500
H	4.33969800	-2.25723800	-5.83216500
H	5.80175600	-1.98035200	-4.87620100
H	4.47419800	-0.74996200	-4.86063500
C	4.58156000	-3.97737500	-3.53453000
H	5.68008300	-3.98353200	-3.38089300
H	4.37333200	-4.56957800	-4.45202300
H	4.10822400	-4.49242100	-2.67032400
C	2.52403500	-2.66518000	-4.00975300
H	2.09059200	-1.66623300	-4.22044500
H	1.96658300	-3.14375900	-3.17969100
H	2.36715700	-3.28857600	-4.91699500
C	-0.23494100	5.04929600	1.63896700
H	-0.95466400	5.86610900	1.86091500
H	-0.26821200	4.34927200	2.49984900
H	-0.58983500	4.53297700	0.72025500
C	4.91355600	5.95923700	2.20377300
H	4.33832100	5.54749700	3.05997600
H	5.28899400	6.96493000	2.48224300
H	5.78480300	5.30034500	2.01460000
C	-4.73639400	2.03960900	0.06437000
H	-3.95554100	1.65206700	0.71783400
C	-4.85685700	3.48960200	-0.01765500
C	-5.98867300	4.10577000	-0.58882200
C	-6.09613100	5.47702500	-0.62544500
C	-5.08606800	6.28873400	-0.07047400
C	-3.95554200	5.68617800	0.50296600
C	-3.85188100	4.30243900	0.52102900
H	-6.79306600	3.49615100	-0.97960900
H	-6.96883000	5.96007400	-1.04502400
H	-3.18859900	6.28834200	0.96671900
H	-2.99340300	3.84853700	1.00137700
O	-5.30030600	7.60613300	-0.13196700
C	-4.38052000	8.51687600	0.48874900
H	-3.39390900	8.45359900	0.02368300
H	-4.79704100	9.50553900	0.32325500
H	-4.30753600	8.32375100	1.56073300
O	-5.87674400	1.35041400	0.05585300
C	-5.97974400	0.16020600	0.87474400
H	-6.29887200	0.45559500	1.87387000

H	-6.74107700	-0.46628600	0.41900900
H	-5.02746400	-0.36759200	0.90790900

(g) Transition state **TS1-A1**, from **R1** and the electrophile having benzyloxo group in the aromatic ring in the position **A1**

Ni	1.09380500	0.58269500	-0.83910300
S	2.20669500	1.66458300	-2.44494200
C	1.09795200	2.72368000	-3.17847000
O	1.58777300	3.58586400	-4.03360900
C	0.52334500	4.47605600	-4.51011200
C	-0.75076700	3.71140900	-4.16987500
N	-0.25243800	2.79492700	-3.09967100
C	-1.06822700	1.87526000	-2.42219100
O	-0.58084000	1.14331300	-1.51400800
H	0.68411100	4.63554900	-5.57151500
H	0.62769100	5.41235700	-3.96406100
H	-1.11397500	3.11433300	-5.00776100
H	-1.54128700	4.34691800	-3.78264200
C	-2.45542900	1.86231600	-2.70165000
H	-2.81339600	2.43835900	-3.54452000
N	-3.07653700	0.61849700	-2.45654800
N	-4.14777700	0.42173200	-3.05123400
N	-5.10738600	0.10378800	-3.54551200
P	2.93903500	0.31434000	0.41662300
P	-0.13123700	-1.01482000	0.22683900
C	2.60622300	0.32331900	2.22104800
C	4.08653800	1.70501200	0.10249900
C	3.84801400	-1.21566000	0.05951800
C	0.76875400	-2.35013600	1.08027900
C	-1.17968600	-1.79864200	-1.05140300
C	-1.20007400	-0.29302700	1.51428300
C	1.56545300	-2.07882400	2.31604400
C	0.65015500	-3.68416700	0.64551300
C	1.75698100	-0.72141600	2.88185000
C	1.34059400	-0.45814000	4.19741300
C	1.22634800	-4.72212100	1.38708700
C	1.90205600	-4.42420100	2.57029400
C	2.06514700	-3.19678400	3.00434200
C	1.73564000	0.59817200	4.86648900
C	2.60824500	1.54228300	4.33548700
O	2.40971500	-5.31591100	3.37978600
O	2.70601600	-3.15652100	4.14491200
O	0.56193100	-1.22964300	4.91004700
O	1.25823100	0.63526200	6.08129000
H	0.09519200	-3.93621000	-0.24610300
H	1.11732700	-5.74903900	1.06279300
H	2.93141400	2.38549900	4.93232900
H	3.71580200	2.15647900	2.63475100
H	5.72336000	0.49218700	-0.54392800
H	2.53557300	3.10402900	0.47724800
H	2.58328900	-1.65964500	-1.58517200
H	5.13159900	-1.03708200	1.74833200
H	-2.69727100	-2.50246000	0.27533500
H	0.13743900	-1.12963400	-2.56264000
H	-1.89332800	-2.16996100	2.23078200
H	-0.38214300	1.61562200	1.16103400
H	-0.59989600	-0.26870100	6.36509300
H	0.83319300	-1.20140500	6.98369900
H	2.41817800	-4.79854300	5.40560000
H	4.02528900	-4.72589700	4.57598800
C	3.05030200	1.40122500	3.01740600
C	3.57284100	3.00094400	0.18856500
C	0.45711400	-0.54804700	6.16784800
C	2.93873500	-4.53555700	4.45991000
C	4.34780300	4.14654900	-0.07317500
C	5.72563600	3.95058700	-0.36257900

C	6.22382000	2.57272500	-0.69500000
C	5.38285300	1.49760700	-0.36839400
C	3.46643600	-1.97293000	-1.04585700
C	4.22394000	-3.06939700	-1.50361200
C	5.45056400	-3.33823100	-0.83606400
C	5.72507600	-2.70124900	0.49692400
C	4.92341500	-1.60472500	0.85187000
C	-2.36285300	-2.46793200	-0.73917500
C	-3.16330500	-3.04777700	-1.73318700
C	-2.58397600	-3.09805700	-3.11834500
C	-1.48730000	-2.26136800	-3.45104600
C	-0.77365300	-1.67760500	-2.38290200
C	-1.08400400	1.07075400	1.77189700
C	-1.81541800	1.71066100	2.79567700
C	-2.80371100	0.94087600	3.47038800
C	-2.77918200	-0.56312400	3.36146100
C	-1.96760000	-1.10200800	2.34986800
O	-3.81926700	1.58963800	4.18011100
O	6.49008000	-3.97683100	-1.51583800
O	-3.00499800	-4.07282000	-4.02816000
O	6.64405700	4.97829800	-0.13855400
C	-2.47644400	-5.36846200	-3.73499600
H	-2.81647400	-6.08002000	-4.51469000
H	-2.83212900	-5.73356000	-2.75214300
H	-1.36516800	-5.35298000	-3.73138000
C	7.34199100	-3.06023200	-2.20447200
H	8.14202300	-3.63049500	-2.71878600
H	7.81805000	-2.35090400	-1.49490600
H	6.77168400	-2.49020800	-2.96795300
C	-5.02529600	1.73472100	3.42571300
H	-5.42257600	0.75188700	3.10549900
H	-4.85979900	2.36120500	2.52753300
H	-5.78803000	2.23111800	4.05928000
C	-1.07712700	-2.00347600	-4.92795200
C	-4.60052400	-3.49146700	-1.36550600
C	-5.35363700	-2.31437200	-0.69573000
H	-6.40338800	-2.59455100	-0.46069100
H	-5.37587500	-1.43479800	-1.36905500
H	-4.88493800	-2.00579600	0.26094400
C	-5.44051700	-3.91880100	-2.59175400
H	-5.09691400	-4.88676200	-3.00708700
H	-5.41041200	-3.13804000	-3.38224500
H	-6.50487000	-4.06931000	-2.30793400
C	-4.54895100	-4.67049600	-0.37046500
H	-4.00745900	-5.53471800	-0.80451800
H	-5.57593000	-5.00505500	-0.10744900
H	-4.03726900	-4.38403500	0.57190000
C	-0.44777500	-3.26753600	-5.54999000
H	0.03076400	-3.03202500	-6.52541400
H	-1.20545700	-4.04716600	-5.75804900
H	0.33054200	-3.68471600	-4.87610800
C	-2.31800700	-1.58352200	-5.75052700
H	-3.08423000	-2.38273800	-5.79234500
H	-2.03615200	-1.35271100	-6.80085000
H	-2.78399400	-0.67657200	-5.30977500
C	-0.04541000	-0.85316400	-5.07252700
H	-0.41603700	0.07402700	-4.58601300
H	0.93538800	-1.13378000	-4.63533000
H	0.14076600	-0.61642300	-6.14278200
C	-1.51692100	3.18613700	3.18779500
C	-3.55505100	-1.53890700	4.28696000
C	-0.25854900	3.75500300	2.47830000
H	-0.41789900	3.83733700	1.38239000
H	0.62982300	3.11707600	2.67366800
H	-0.02009000	4.77665200	2.84646800
C	-2.70081000	4.10861900	2.82770700
H	-2.39867700	5.17792800	2.86216900

H	-3.53620800	4.00683400	3.54718900
H	-3.06733100	3.88885600	1.80310800
C	-1.24134300	3.26980300	4.70677200
H	-2.12525500	2.97745500	5.30808900
H	-0.97407400	4.30718000	5.00387000
H	-0.39942800	2.60104700	4.98302300
C	-4.63746400	-2.26581700	3.46115700
H	-5.33199800	-1.54146900	2.98907800
H	-5.23349300	-2.94717400	4.10623500
H	-4.18034100	-2.87653000	2.65423400
C	-4.23284400	-0.84689900	5.49370000
H	-4.61024200	-1.59819600	6.22116300
H	-5.12162400	-0.26126200	5.19235100
H	-3.51030800	-0.19583900	6.03148000
C	-2.59776300	-2.60440500	4.88075000
H	-1.80040900	-2.12091000	5.47503800
H	-2.12634500	-3.24097100	4.10600300
H	-3.14695500	-3.29653800	5.55559200
C	3.64468900	5.52879000	-0.01405800
C	7.54547700	2.27934000	-1.44290200
C	4.54007900	6.71673100	-0.44336600
H	5.31704600	6.93817600	0.31697500
H	3.94108100	7.64821500	-0.54177500
H	5.01466900	6.51884000	-1.42868100
C	3.15160400	5.79730800	1.42388200
H	2.66183700	6.79292200	1.49186500
H	4.00319200	5.77801700	2.13640400
H	2.41198000	5.03743200	1.75030500
C	8.31396600	3.55342400	-1.86464300
H	7.65413100	4.24459700	-2.43213200
H	9.17338900	3.29611000	-2.52135000
H	8.74577900	4.07662800	-0.98720900
C	8.47332500	1.43325700	-0.54519200
H	9.44370200	1.24253200	-1.05282600
H	8.02157100	0.44786700	-0.30740700
H	8.67775700	1.96047500	0.41059900
C	7.23566400	1.48973300	-2.73862300
H	8.16129600	1.31432700	-3.32873300
H	6.51915200	2.05543400	-3.37295000
H	6.79971300	0.49171000	-2.52480700
C	6.78277400	-3.21048400	1.50096200
C	3.69884000	-3.86430500	-2.72890600
C	7.83374000	-2.11042300	1.76262800
H	8.61400600	-2.47378700	2.46609500
H	7.37650800	-1.20396800	2.21099200
H	8.33244700	-1.81583600	0.81567800
C	6.07804700	-3.56944000	2.82705500
H	5.58655900	-2.68784400	3.28879600
H	6.80225000	-3.96764700	3.57046100
H	5.30580500	-4.34532400	2.63921000
C	7.52597200	-4.48234500	1.02875100
H	8.21564300	-4.26211300	0.18855900
H	6.80639800	-5.27512300	0.73048500
H	8.15839800	-4.89437600	1.84513300
C	3.67826700	-2.95070300	-3.97269300
H	3.30105600	-3.50391500	-4.86018900
H	4.69937400	-2.58028100	-4.20147400
H	3.02029600	-2.07109500	-3.82046900
C	4.53194400	-5.12419600	-3.07119200
H	5.51019000	-4.85799500	-3.52190800
H	4.01240100	-5.74877300	-3.83021400
H	4.68700400	-5.75315100	-2.16803300
C	2.25858800	-4.35374200	-2.43996200
H	1.54893000	-3.51105900	-2.31730400
H	2.24094900	-4.97028600	-1.51587200
H	1.87090400	-4.97452200	-3.27643300
C	2.42761400	5.52349000	-0.97466700

H	1.93368800	6.51901800	-0.99997500
H	1.64961300	4.79496500	-0.66684800
H	2.75390700	5.27126000	-2.00667200
C	7.17744300	4.95978500	1.18557600
H	6.36927900	5.09113000	1.93640300
H	7.89595800	5.79745100	1.29484300
H	7.71594200	4.00842600	1.38177400
C	-3.11495800	3.19574400	-1.19510000
H	-2.38673300	2.89317800	-0.44254900
C	-4.46276900	2.77418800	-0.98312100
C	-5.56254700	3.40668000	-1.61495000
C	-6.83782100	2.95010800	-1.41549200
C	-7.07774700	1.82464900	-0.58947800
C	-5.99170600	1.18332600	0.04185600
C	-4.71419400	1.65545500	-0.16088500
H	-5.39034600	4.26973900	-2.24450200
H	-7.68980900	3.43262500	-1.87566300
H	-6.15522200	0.33628100	0.69056800
H	-3.88689400	1.16310000	0.33526600
O	-8.34411000	1.46177100	-0.46449800
C	-8.71187100	0.31069100	0.37181200
H	-8.37948800	0.52006900	1.39078100
H	-8.18506000	-0.56441300	-0.01438800
O	-2.87192500	4.40582000	-1.77632300
C	-2.39982300	5.43345100	-0.87455000
H	-3.15216500	5.64714900	-0.11470800
H	-1.46433300	5.12943500	-0.39815700
H	-2.23608000	6.31939000	-1.48209100
C	-10.19336800	0.12847700	0.30379500
C	-10.75592900	-0.69113200	-0.67606700
C	-11.02698900	0.78167500	1.21303300
C	-12.13435500	-0.85061200	-0.75045100
H	-10.11499100	-1.20836900	-1.38141700
C	-12.40560800	0.62286700	1.13910500
H	-10.59858400	1.41444800	1.98227300
C	-12.95964900	-0.19270000	0.15675900
H	-12.56451800	-1.49060100	-1.51024600
H	-13.04685600	1.12965800	1.84893500
H	-14.03341600	-0.32004900	0.10202000

(h) Transition state **TS1-A2**, from **R1** and the electrophile having benzyloxo group in the aromatic ring in the position **A2**

Ni	0.19999000	0.27565800	-0.72030900
S	-0.86754000	1.74245800	-2.01464900
C	-2.37591500	1.06161900	-2.39489900
O	-3.20717700	1.82404700	-3.05613900
C	-4.48689600	1.12993700	-3.24316300
C	-4.13350900	-0.33079900	-2.99381300
N	-2.87117700	-0.18886000	-2.20404900
C	-2.15618700	-1.27336300	-1.68062700
O	-1.07730800	-1.08505600	-1.05700000
H	-4.82873400	1.34635800	-4.25026300
H	-5.17651100	1.53656100	-2.50604300
H	-3.91534800	-0.87337800	-3.91478300
H	-4.89336600	-0.84569400	-2.41534800
C	-2.72548800	-2.57504100	-1.78586900
H	-3.54436200	-2.69776100	-2.48315900
N	-1.75774000	-3.61853800	-1.77091400
N	-2.06581900	-4.63229800	-2.41771300
N	-2.21642200	-5.59708500	-2.97700000
P	1.34974900	1.90036100	0.34148100
P	1.54713600	-1.43713400	-0.07816700
C	1.77641600	1.43754900	2.06376700
C	0.27833600	3.38610600	0.43840200
C	2.92501700	2.45584000	-0.37774100
C	3.29858500	-1.02785500	0.20343700

C	1.47934300	-2.68397800	-1.42033000
C	1.02425400	-2.23730600	1.47427700
C	3.72676200	-0.19571800	1.37413900
C	4.29504800	-1.57434700	-0.62706500
C	2.77801400	0.36039500	2.37438000
C	2.90812600	-0.00574500	3.72358900
C	5.64707300	-1.36500900	-0.33735200
C	5.99633600	-0.62491700	0.79033300
C	5.11084300	-0.07700800	1.58443700
C	2.24015900	0.57774300	4.68482400
C	1.35644800	1.62724200	4.46005400
O	7.28465600	-0.44283400	1.25020500
O	5.71744700	0.54447100	2.66080900
O	3.75988000	-0.97235500	4.22038200
O	2.56294100	0.06619900	5.92456900
H	4.04035500	-2.18221600	-1.48312400
H	6.41383100	-1.80501200	-0.96144300
H	0.84668200	2.09920600	5.28989200
H	0.44909300	2.88498700	3.01375800
H	1.59156700	4.65787200	-0.66261900
H	-1.29677700	2.25285100	1.26941200
H	2.76332700	1.12574900	-2.00836300
H	3.35066000	3.73621300	1.26983300
H	1.91269300	-4.37276600	-0.18975800
H	0.93785400	-1.19345600	-2.83630800
H	2.75412300	-3.46511400	1.60437500
H	-0.59283000	-0.89830500	1.67806200
H	3.17271500	-1.92809900	5.98808800
H	4.49401800	-0.69687100	6.15821600
H	7.49618300	-0.32929100	3.32611300
H	7.68687100	1.23048900	2.43472000
C	1.13286200	2.06531000	3.15187100
C	-1.01865600	3.23847200	0.92881300
C	3.54304500	-0.94139400	5.63994400
C	7.11799700	0.28036500	2.47845700
C	-1.92498900	4.31985600	0.96772100
C	-1.44280500	5.59588600	0.55297500
C	-0.22045700	5.70282600	-0.17701900
C	0.64472600	4.59266600	-0.15792200
C	3.36273900	1.91815800	-1.58736300
C	4.50707700	2.41475100	-2.25403500
C	5.15167700	3.54772300	-1.68704700
C	4.84928500	3.94663200	-0.27090600
C	3.68338100	3.41229800	0.29318700
C	1.69331200	-4.04104500	-1.18106200
C	1.58127200	-4.99871800	-2.19824500
C	1.39465700	-4.48369200	-3.59543600
C	1.01713800	-3.12800700	-3.80088800
C	1.12827000	-2.24937700	-2.70182200
C	-0.09675900	-1.74004900	2.13530200
C	-0.56472700	-2.30124200	3.34341900
C	0.06962900	-3.48700700	3.80039300
C	1.41733900	-3.87003300	3.25047800
C	1.81366100	-3.22167200	2.07043600
O	-0.62821300	-4.35416200	4.65012200
O	5.93523900	4.37273300	-2.49904400
O	1.80001300	-5.27233900	-4.67418700
O	-2.15034500	6.74216600	0.92669600
C	3.19448000	-5.14792400	-4.95513700
H	3.44659400	-5.79933800	-5.81650500
H	3.80387200	-5.46754900	-4.08429100
H	3.45079400	-4.10059500	-5.22130700
C	5.22753800	5.52969000	-2.95433600
H	5.89644900	6.12664400	-3.60704900
H	4.90841700	6.16663800	-2.10585700
H	4.32920200	5.24064400	-3.54040800
C	-1.19523600	-5.49028500	3.98327200

H	-0.95937100	-5.52618600	2.89580200
H	-2.29710100	-5.47711000	4.09696200
H	-0.82770800	-6.42215000	4.45698400
C	0.49947700	-2.55773400	-5.15093400
C	1.61099900	-6.49808500	-1.81251700
C	0.55070700	-6.77779400	-0.71650000
H	0.52442600	-7.85796900	-0.45547500
H	-0.45927400	-6.48375800	-1.06752700
H	0.76132800	-6.23025800	0.22436800
C	1.29420200	-7.44459600	-2.99410300
H	2.12755800	-7.47730500	-3.72523600
H	0.35480700	-7.14014900	-3.50375400
H	1.16074700	-8.48951300	-2.63868700
C	3.00597000	-6.86258800	-1.26213900
H	3.79072600	-6.66448100	-2.02155400
H	3.05087200	-7.93946600	-0.98996200
H	3.25125600	-6.27472000	-0.35330300
C	1.48423800	-1.49505900	-5.67947200
H	1.18110600	-1.14246800	-6.68923800
H	2.51144800	-1.91112400	-5.74801300
H	1.50593200	-0.60839100	-5.01551100
C	0.30040500	-3.62047300	-6.25970300
H	1.27075900	-3.98997400	-6.64996900
H	-0.22886400	-3.18506500	-7.13524400
H	-0.31370400	-4.46956000	-5.88910900
C	-0.88133600	-1.89230300	-4.93065100
H	-1.60543000	-2.62997900	-4.52506500
H	-0.82324700	-1.03269800	-4.23317300
H	-1.29076700	-1.49683900	-5.88547100
C	-1.71818600	-1.63989500	4.14509400
C	2.35112600	-4.92556600	3.89866900
C	-2.11868700	-0.25073100	3.58544100
H	-2.56731400	-0.34402600	2.57466000
H	-1.24040700	0.42695600	3.53685600
H	-2.87917700	0.23661800	4.23379100
C	-2.98360700	-2.52390400	4.12690600
H	-3.87453000	-1.95185000	4.46593900
H	-2.89536000	-3.38276400	4.81832000
H	-3.18448400	-2.89561300	3.10056700
C	-1.26240800	-1.41622500	5.60537200
H	-1.03909300	-2.37263400	6.12037200
H	-2.05495100	-0.90420600	6.19318900
H	-0.34622800	-0.78772300	5.63203800
C	2.22996300	-6.25235100	3.12528700
H	1.19308900	-6.63972400	3.14898600
H	2.89522200	-7.02578200	3.56669800
H	2.51952100	-6.11251400	2.06220800
C	2.02169200	-5.16720600	5.39295700
H	2.83276400	-5.73520500	5.89788600
H	1.11323700	-5.78171400	5.52816600
H	1.89826100	-4.20110600	5.92795100
C	3.83613600	-4.47718700	3.84410500
H	3.95755000	-3.47270900	4.28784600
H	4.23980500	-4.46631100	2.81139400
H	4.48659900	-5.17877000	4.41017400
C	-3.41329300	4.10420100	1.36644900
C	0.18050200	6.91470800	-1.06547000
C	-4.32767200	4.60324900	0.22399900
H	-4.22170300	5.69364800	0.05393300
H	-5.39700100	4.41108400	0.46035700
H	-4.08057300	4.07852300	-0.72480000
C	-3.75755100	4.85398300	2.67200700
H	-4.73748900	4.51917300	3.07663600
H	-3.85703900	5.94455700	2.51208800
H	-2.98318400	4.66150400	3.44543400
C	-0.90282900	8.01619000	-1.17883000
H	-1.88204000	7.58048200	-1.47275900

H	-0.62691500	8.76366400	-1.95427300
H	-1.00787100	8.59339100	-0.24007800
C	1.47270000	7.55921900	-0.51902800
H	1.75833900	8.44376100	-1.12870200
H	2.32370300	6.84742800	-0.54521000
H	1.33416100	7.89127600	0.53032200
C	0.44254200	6.41826000	-2.51195400
H	0.66075300	7.26835400	-3.19435500
H	-0.44819100	5.87898400	-2.90150800
H	1.31685200	5.73842900	-2.57427200
C	5.77379300	4.81562300	0.61363400
C	5.04187100	1.74952600	-3.55264800
C	5.04690200	6.11427600	1.02381600
H	5.70399800	6.74507600	1.66097300
H	4.12377400	5.89991400	1.60197300
H	4.76403200	6.70792500	0.13129300
C	6.14028700	4.02346500	1.88918800
H	5.25077300	3.79802700	2.51323800
H	6.84824900	4.59493700	2.52793300
H	6.61990300	3.06666500	1.60407600
C	7.10740700	5.19636300	-0.07218900
H	6.95748300	5.93997100	-0.87928200
H	7.61486400	4.29506500	-0.47890100
H	7.80167200	5.67310900	0.65362000
C	4.89652000	2.69327600	-4.76753200
H	5.06610400	2.14419900	-5.71933100
H	5.64379700	3.50964100	-4.75583800
H	3.87453700	3.12838700	-4.80020300
C	6.52948800	1.38007400	-3.35814400
H	7.15761500	2.27857800	-3.18907600
H	6.92941300	0.86172600	-4.25659900
H	6.64825600	0.70517300	-2.48266500
C	4.29417700	0.44074300	-3.90839600
H	3.23495100	0.65547500	-4.15713000
H	4.33867000	-0.28406300	-3.07129200
H	4.74668000	-0.05169400	-4.79670400
C	-3.76509700	2.61199600	1.59548300
H	-4.84674100	2.48929200	1.81774300
H	-3.20984300	2.20508300	2.46657300
H	-3.54020600	2.00714000	0.68995600
C	-1.59622600	7.37607000	2.08443200
H	-1.59166400	6.68531400	2.95405300
H	-2.21376500	8.26101100	2.34026500
H	-0.55751200	7.71589600	1.89875300
C	-3.77525500	-2.86208800	-0.04795600
H	-3.01730400	-2.45269100	0.61940600
C	-5.03144800	-2.12875400	-0.12691800
C	-6.18971100	-2.69950200	-0.69314800
C	-7.37180600	-1.99657900	-0.72830800
C	-7.45461400	-0.69922900	-0.17786800
C	-6.30475500	-0.12249000	0.38892700
C	-5.11569300	-0.83599000	0.40549000
H	-6.15653300	-3.70956500	-1.08070000
H	-8.27094400	-2.43291900	-1.14327400
H	-6.35040600	0.85276700	0.84855800
H	-4.24799200	-0.39289700	0.87904400
O	-8.65228900	-0.11743600	-0.23549100
C	-8.86883300	1.18176600	0.40275100
H	-8.21742200	1.91392500	-0.08199300
H	-8.59088100	1.09082600	1.45536000
C	-10.30845700	1.55726600	0.25088700
C	-10.72734800	2.32542600	-0.83631300
C	-11.24756800	1.13686200	1.19431200
C	-12.06720800	2.66446100	-0.98284600
C	-12.58756000	1.47445900	1.04865500
C	-12.99792200	2.23816300	-0.04024500
H	-10.00369400	2.66396500	-1.56976300

H	-10.93071700	0.54605600	2.04637600
H	-12.38499900	3.26441200	-1.82609400
H	-13.31013400	1.14840600	1.78580700
H	-14.04108600	2.50599100	-0.15062800
O	-3.86409600	-4.19408800	-0.06813600
C	-2.96490300	-4.95936000	0.76936300
H	-3.39349900	-5.02560500	1.76909200
H	-2.90257000	-5.95186800	0.33240300
H	-1.97838800	-4.49777900	0.79676200

(*i*) Transition state **TS2-A1**, from **R2** and the electrophile having benzyl group into carbonyl in the position **A1**

Ni	0.49415000	0.44781200	-0.73700300
S	1.28857500	2.12090000	-1.97630200
C	-0.04175100	3.02739200	-2.52525500
O	0.23436700	4.06876200	-3.26577900
C	-0.99510800	4.78841900	-3.62283000
C	-2.11092600	3.81765300	-3.23883500
N	-1.37522300	2.84049500	-2.37828500
C	-1.98103800	1.71298000	-1.80665600
O	-1.29761400	0.88134700	-1.14544400
H	-0.93910200	5.00888600	-4.68435100
H	-1.00676500	5.70582800	-3.03935300
H	-2.52363000	3.29134200	-4.09968400
H	-2.90033800	4.29822000	-2.66774300
C	-3.38969000	1.57675300	-1.91351300
H	-3.90452600	2.24253600	-2.59275700
N	-3.83632700	0.23005400	-1.88270000
N	-4.88643900	0.00876700	-2.50475700
N	-5.81541000	-0.32297300	-3.04651900
P	2.45111600	0.21722100	0.37590500
P	-0.37849200	-1.55997000	-0.10246200
C	2.22246100	-0.37606500	2.09781000
C	3.27018800	1.85769300	0.47719500
C	3.68905400	-0.91423900	-0.32508200
C	0.81142200	-2.90016500	0.21905800
C	-1.49723000	-2.12259900	-1.44444300
C	-1.35242100	-1.45955000	1.43657600
C	1.71507300	-2.87247200	1.41474600
C	0.82886800	-4.04014800	-0.60577500
C	1.72705700	-1.76208400	2.40682500
C	1.43175600	-2.03822900	3.75357700
C	1.64463200	-5.12964700	-0.28650400
C	2.42197800	-5.08569100	0.86944900
C	2.46332600	-4.03830800	1.66055700
C	1.62371100	-1.16768800	4.71565800
C	2.15349700	0.09760800	4.49099600
O	3.14552100	-6.08018400	1.31034600
O	3.23167000	-4.23805300	2.70168000
O	0.97264300	-3.17556800	4.20597200
O	1.30968400	-1.63630700	5.89336600
H	0.19901100	-4.10831000	-1.48094400
H	1.64156300	-6.01392800	-0.91076000
H	2.32411200	0.77397200	5.31879300
H	2.89310000	1.46613600	3.05588900
H	4.99636100	1.29093300	-0.64120500
H	1.56067300	2.71580100	1.36507100
H	2.46222000	-1.39212000	-1.97410600
H	4.99788600	-0.68889400	1.33933700
H	-2.79268900	-3.26847400	-0.19368000
H	-0.42569200	-0.98458700	-2.88637300
H	-1.59604200	-3.56398600	1.60901200
H	-0.96545400	0.60690900	1.60065900
H	-0.19267200	-3.08268100	5.94452000
H	1.50228000	-3.69999300	6.15989500
H	3.33066000	-6.21035600	3.38766500

H	4.79732300	-5.62333200	2.50751800
C	2.46775600	0.48713500	3.18725800
C	2.55460000	2.93174100	1.00516700
C	0.86313700	-2.96859700	5.62084500
C	3.69003900	-5.58774700	2.54046200
C	3.11108600	4.22743800	1.08626400
C	4.44267400	4.40184500	0.60814400
C	5.06763500	3.38540000	-0.17361200
C	4.49829900	2.09916100	-0.13895100
C	3.43881200	-1.54681500	-1.54184800
C	4.42848900	-2.31123600	-2.20256200
C	5.72509300	-2.34125200	-1.62102000
C	5.91513100	-1.89545800	-0.19937400
C	4.88266400	-1.13045900	0.35894600
C	-2.59853700	-2.94062900	-1.19078900
C	-3.49594300	-3.31201200	-2.20085100
C	-3.12410700	-2.93955700	-3.60587100
C	-2.09924000	-1.97944300	-3.83201900
C	-1.27109700	-1.64250800	-2.73849100
C	-1.47170600	-0.22358600	2.06711200
C	-2.21676300	-0.05672800	3.25418600
C	-2.96017500	-1.17326100	3.72084600
C	-2.63525000	-2.55166500	3.20911400
C	-1.84567700	-2.61283300	2.04969100
O	-4.07373200	-0.95770800	4.54249400
O	6.83336200	-2.62756700	-2.42323000
O	-3.63459900	-3.68797200	-4.66852000
O	5.16933900	5.53052700	1.00803000
C	-2.87501500	-4.86914000	-4.92686500
H	-3.33578300	-5.41352700	-5.77610000
H	-2.87152900	-5.53908600	-4.04206400
H	-1.82967100	-4.61333500	-5.20081200
C	7.51398900	-1.44844100	-2.86291800
H	8.36595500	-1.74456200	-3.50810500
H	7.91004900	-0.86843800	-2.00599700
H	6.83614300	-0.79482800	-3.45218100
C	-5.32852700	-1.03087300	3.85234200
H	-5.22452900	-1.32158500	2.78378900
H	-5.83680400	-0.04723000	3.89342900
H	-5.98773000	-1.76540800	4.35635100
C	-1.83701000	-1.28646500	-5.19877200
C	-4.80763500	-4.03270900	-1.80079700
C	-5.55255100	-3.20832500	-0.71880800
H	-6.51531800	-3.69093200	-0.44345700
H	-5.77537300	-2.18854900	-1.09079400
H	-4.96796600	-3.11204700	0.21806300
C	-5.79305100	-4.21752600	-2.97860000
H	-5.43391300	-4.98445700	-3.69476800
H	-5.96189400	-3.25518500	-3.50776600
H	-6.77960600	-4.57932400	-2.61551200
C	-4.47601500	-5.42575200	-1.22561800
H	-3.93921300	-6.04206500	-1.97637300
H	-5.40587100	-5.96255800	-0.93758000
H	-3.83565700	-5.35109000	-0.32246100
C	-0.44065300	-1.67805000	-5.72196300
H	-0.27139100	-1.26975500	-6.74202100
H	-0.33197900	-2.78220900	-5.76443600
H	0.35500700	-1.26945600	-5.06918800
C	-2.87359300	-1.62668300	-6.29817100
H	-2.75119300	-2.66535900	-6.66766800
H	-2.73623300	-0.97412400	-7.18789600
H	-3.90981900	-1.46933000	-5.92811300
C	-1.88691000	0.24995300	-5.01358400
H	-2.87641700	0.55870800	-4.61562500
H	-1.09801400	0.61267500	-4.32454800
H	-1.72624000	0.77420500	-5.98058000
C	-2.21925800	1.29377400	4.01934300

C	-3.13163800	-3.86311800	3.87413600
C	-1.18424600	2.30318100	3.45935900
H	-1.45514000	2.62401400	2.43241900
H	-0.16416500	1.86281000	3.45050100
H	-1.14566300	3.22150000	4.08489300
C	-3.60739800	1.96709900	3.94695300
H	-3.54718800	3.03971400	4.23287700
H	-4.32407700	1.50616700	4.65286900
H	-4.01827300	1.90830400	2.91706900
C	-1.83846300	1.04391200	5.49693800
H	-2.57709600	0.39653300	6.01167900
H	-1.79218000	2.00098200	6.06059900
H	-0.84400000	0.55147600	5.56144400
C	-4.34700400	-4.40157900	3.09547000
H	-5.18658100	-3.68087400	3.10442200
H	-4.70974400	-5.35199600	3.54355800
H	-4.07509200	-4.59978400	2.03741400
C	-3.52051500	-3.65850700	5.35961700
H	-3.64157600	-4.63165700	5.88276300
H	-4.49449900	-3.14820600	5.47136800
H	-2.73708100	-3.07935500	5.89364500
C	-2.03463000	-4.96131100	3.85344600
H	-1.09599500	-4.58703800	4.29883300
H	-1.82473000	-5.33269600	2.82981000
H	-2.35194000	-5.85440300	4.43434400
C	2.27179600	5.42993400	1.60339900
C	6.30508800	3.61823600	-1.08417800
C	2.15712200	6.48069600	0.47688000
H	3.15089400	6.88489500	0.19302000
H	1.52559700	7.33782000	0.79722400
H	1.70006200	6.02721000	-0.42929300
C	2.92012300	6.07483900	2.84989900
H	2.19041700	6.70380000	3.40491400
H	3.74612000	6.75927600	2.57680200
H	3.29644200	5.29260000	3.54386800
C	6.60313200	5.11558300	-1.35775900
H	5.67538300	5.65440700	-1.64806300
H	7.33585800	5.23490600	-2.18510700
H	7.07119200	5.61727800	-0.49213100
C	7.54232000	2.96623100	-0.43852300
H	8.44087300	3.11347300	-1.07604300
H	7.38979900	1.87408400	-0.30426900
H	7.74524300	3.41023800	0.55788700
C	6.06900000	2.97080200	-2.47770600
H	6.87964200	3.23769400	-3.18994700
H	5.10306700	3.31632600	-2.90600500
H	6.06419500	1.86248400	-2.43749200
C	7.11208200	-2.29553800	0.69430700
C	4.11397800	-3.08852700	-3.51076000
C	7.89477300	-1.03662300	1.12536600
H	8.76383900	-1.31521300	1.76001000
H	7.25884500	-0.34235800	1.71351200
H	8.27742000	-0.48721400	0.24156400
C	6.57997600	-3.01103300	1.95640000
H	5.94101500	-2.34876800	2.57662000
H	7.41444800	-3.35732300	2.60409300
H	5.98177100	-3.89285000	1.65438900
C	8.09618600	-3.27396800	0.01013100
H	8.68517300	-2.77636300	-0.78499800
H	7.55581600	-4.14883400	-0.41164400
H	8.83964600	-3.66015200	0.74102300
C	4.89051300	-2.50417600	-4.71222100
H	4.49943900	-2.90730200	-5.67175900
H	5.96349000	-2.77474600	-4.68954600
H	4.78859600	-1.39793500	-4.73855000
C	4.49275500	-4.57429100	-3.32161200
H	5.57939000	-4.70094200	-3.13793200

H	4.23925500	-5.16583500	-4.22807200
H	3.94294400	-5.00565500	-2.45687500
C	2.61206500	-3.04873100	-3.88496700
H	2.30338800	-2.01155500	-4.12754800
H	1.98440300	-3.43889600	-3.05904300
H	2.40436200	-3.67240200	-4.78176800
C	0.82863700	5.03059300	2.00403400
H	0.23425300	5.92041400	2.30644600
H	0.84397300	4.33055200	2.86638000
H	0.29664900	4.55682800	1.15413100
C	6.09069500	5.26968600	2.07963300
H	6.07264100	4.21223400	2.43062900
H	5.85248300	5.92083200	2.94368600
H	7.12221300	5.51611800	1.75992800
C	-4.02381300	2.47808700	-0.15625400
H	-3.27893000	2.00835600	0.48475500
C	-5.36691800	1.97791100	-0.03169100
C	-6.48651100	2.70868400	-0.49116000
C	-7.74916300	2.18297100	-0.39213600
C	-7.94651700	0.89749700	0.15945900
C	-6.84290400	0.16232200	0.62702400
C	-5.57672900	0.70284300	0.52532400
H	-6.34490100	3.69782200	-0.90618000
H	-8.61845400	2.73346700	-0.72632900
H	-6.97916400	-0.81402000	1.06763000
H	-4.72766200	0.13560300	0.88554200
O	-3.84531200	3.80154100	-0.38044700
C	-2.93297000	4.48015100	0.55024600
H	-1.95701800	3.99252500	0.48631400
H	-3.33318900	4.34080900	1.55682200
C	-2.83344400	5.93065300	0.19727800
C	-1.65994300	6.45132200	-0.35128600
C	-3.90662500	6.79082900	0.44310800
C	-1.55218700	7.80721000	-0.64499300
C	-3.80733500	8.14202700	0.13634500
C	-2.62983900	8.65286900	-0.40495100
H	-0.80886600	5.80128000	-0.51973200
H	-4.81818100	6.40465100	0.88389700
H	-0.62666800	8.20572800	-1.04186900
H	-4.64191700	8.80217900	0.33460100
H	-2.54934300	9.70952400	-0.62557300
O	-9.20817100	0.47732300	0.20299400
C	-9.52449800	-0.81731300	0.74247400
H	-9.23308300	-0.87944800	1.79253000
H	-10.60249500	-0.90867100	0.65554200
H	-9.03986400	-1.60545900	0.16298700

(*j*) Transition state **TS2-A2**, from **R2** and the electrophile having benzyl group into carbonyl in the position **A2**

Ni	-0.54526300	0.49651700	0.79938800
S	-1.32374300	2.16396700	2.05690700
C	0.02042800	3.07913000	2.54703800
O	-0.23465300	4.19127000	3.18575500
C	1.01577000	4.90427900	3.47244700
C	2.07938100	3.82455200	3.31561000
N	1.35302200	2.83015200	2.46693700
C	1.92704600	1.64089000	1.99965000
O	1.24688700	0.83136400	1.31342100
H	0.93264200	5.31496000	4.47361500
H	1.10024200	5.70006600	2.73497100
H	2.34390800	3.35427000	4.26375600
H	2.96605200	4.18874500	2.80700500
C	3.31553200	1.42027200	2.22536200
H	3.80583500	2.06214800	2.94607600
N	3.66905300	0.04299300	2.27658300
N	4.64353900	-0.22989000	2.99455600

N	5.49827700	-0.61714500	3.61589700
P	-2.43924700	0.42741500	-0.42779400
P	0.24627500	-1.54230600	0.17537900
C	-2.14415600	-0.17791000	-2.13275300
C	-3.08844200	2.13854400	-0.56666100
C	-3.82993700	-0.59023100	0.15446800
C	-1.01293800	-2.80372200	-0.20067700
C	1.22653200	-2.16872500	1.59179800
C	1.31242700	-1.51832500	-1.30429500
C	-1.88660900	-2.70167500	-1.41446800
C	-1.13253300	-3.94720100	0.61240700
C	-1.77450700	-1.60759900	-2.41446400
C	-1.46244300	-1.92505500	-3.74562700
C	-2.03110000	-4.96320400	0.27393800
C	-2.79688800	-4.83866400	-0.88247200
C	-2.73752600	-3.79140900	-1.66647900
C	-1.52206500	-1.05080300	-4.71641800
C	-1.92116900	0.26702500	-4.52138000
O	-3.64130200	-5.80968700	-1.38000100
O	-3.55060800	-3.94712800	-2.77432500
O	-1.10301300	-3.17274700	-4.21547500
O	-1.20252300	-1.60975900	-5.93657200
H	-0.52846500	-4.07303700	1.49893900
H	-2.10904100	-5.85068500	0.88827600
H	-1.98353900	0.94849200	-5.35979300
H	-2.56278700	1.72269900	-3.11749500
H	-4.93304800	1.73288500	0.42816500
H	-1.22757200	2.84409200	-1.27256700
H	-2.77221100	-1.25043200	1.85673800
H	-4.99232000	-0.16825300	-1.57873000
H	2.56019300	-3.37800800	0.44498500
H	0.10817100	-0.96731500	2.94251600
H	1.43958600	-3.63407300	-1.42149100
H	1.06531800	0.56544600	-1.51616000
H	0.11804000	-3.22643200	-5.91405600
H	-1.62705700	-3.64075200	-6.18406100
H	-3.79054100	-5.90710500	-3.46181000
H	-5.21990500	-5.20724300	-2.60479800
C	-2.24440700	0.70086400	-3.23240600
C	-2.22604500	3.14693800	-0.99663900
C	-0.92630400	-2.98312200	-5.62795000
C	-4.11300600	-5.26047900	-2.61857200
C	-2.63579400	4.49699800	-1.05830900
C	-3.99238600	4.78717800	-0.73095000
C	-4.79192000	3.82087100	-0.05016600
C	-4.33338100	2.49023700	-0.04351100
C	-3.72577800	-1.29356600	1.35342700
C	-4.82394500	-1.99466200	1.90465600
C	-6.07283400	-1.88480100	1.23283800
C	-6.12095600	-1.35530700	-0.17277300
C	-4.98703300	-0.66398600	-0.61766500
C	2.28477300	-3.06285800	1.42847800
C	3.02581000	-3.53999100	2.51984000
C	2.54252500	-3.15308400	3.88773700
C	1.59289900	-2.10410600	4.02728800
C	0.91101100	-1.68761600	2.86515500
C	1.53539100	-0.30344100	-1.94979500
C	2.32421000	-0.20718600	-3.11833900
C	3.00578000	-1.37967900	-3.54407500
C	2.58075400	-2.72309400	-3.01240000
C	1.76038400	-2.71016800	-1.87354500
O	4.12805300	-1.26072000	-4.37376700
O	-7.25687400	-2.12244900	1.93681300
O	2.85800100	-3.96333100	4.98041800
O	-4.54392600	6.00434400	-1.14149800
C	1.96100300	-5.06475600	5.12710300
H	2.27033800	-5.66484200	6.00690400

H	1.98736100	-5.71863100	4.23034200
H	0.92270600	-4.70812300	5.29411800
C	-7.87330800	-0.91800700	2.40187900
H	-8.78899300	-1.17906900	2.97039700
H	-8.16206700	-0.26132300	1.55831400
H	-7.19041300	-0.35460200	3.07265100
C	5.37971600	-1.41152100	-3.69065700
H	5.26553800	-1.63539700	-2.60649200
H	5.97255000	-0.48255400	-3.79359900
H	5.96802800	-2.22365400	-4.16141100
C	1.25608700	-1.39932100	5.37036700
C	4.28988100	-4.39156200	2.24439500
C	5.24165200	-3.62106400	1.29629700
H	6.16911700	-4.20308600	1.10450400
H	5.53240700	-2.65258000	1.75109700
H	4.78293900	-3.41966600	0.30670600
C	5.10284300	-4.72312300	3.51786800
H	4.57812900	-5.46305300	4.15586000
H	5.31684400	-3.80176800	4.10145300
H	6.07812200	-5.18720800	3.25409600
C	3.88181600	-5.72025200	1.57336800
H	3.18313900	-6.28773000	2.22304200
H	4.77460700	-6.35537700	1.38542100
H	3.38101900	-5.54730400	0.59818500
C	-0.21789800	-1.66059500	5.74228600
H	-0.45260600	-1.24153700	6.74482900
H	-0.43140500	-2.75000300	5.76023300
H	-0.90329500	-1.17888200	5.01690800
C	2.13827200	-1.84426100	6.56279000
H	1.88267400	-2.86999700	6.89905500
H	1.97168700	-1.18834400	7.44497700
H	3.21702100	-1.78320200	6.30201600
C	1.46816300	0.12698300	5.21386000
H	2.51746600	0.34395600	4.92216500
H	0.79245200	0.56614300	4.45236700
H	1.25849300	0.65859600	6.16733600
C	2.41320500	1.12013500	-3.92187000
C	2.99229100	-4.07868500	-3.64578400
C	1.42528300	2.19902700	-3.40971200
H	1.69615900	2.53169200	-2.38573800
H	0.38345700	1.81630100	-3.40885600
H	1.44713600	3.10094300	-4.05958200
C	3.82861500	1.72968500	-3.84266400
H	3.83521900	2.76705300	-4.24239600
H	4.55649500	1.16664100	-4.45401700
H	4.17750000	1.76960800	-2.79066200
C	2.04909600	0.84946400	-5.39995400
H	2.77170400	0.16355600	-5.88629100
H	2.04906300	1.79326300	-5.98739300
H	1.03816500	0.39403600	-5.47151900
C	4.16223000	-4.67933200	-2.84302200
H	5.05137200	-4.02123300	-2.86866700
H	4.45690000	-5.66698400	-3.25933100
H	3.87386800	-4.82210600	-1.77969600
C	3.40268800	-3.93415900	-5.13276200
H	3.45892100	-4.92428800	-5.63453600
H	4.41005200	-3.49680600	-5.25069400
H	2.66414600	-3.31370700	-5.68417600
C	1.82154300	-5.09836200	-3.61263000
H	0.90921900	-4.66011000	-4.05692300
H	1.59148600	-5.44666100	-2.58502500
H	2.07217500	-6.01673900	-4.18658000
C	-1.62203900	5.62987200	-1.38786500
C	-6.07665200	4.14638500	0.76337700
C	-1.62301100	6.66921500	-0.24304800
H	-2.60466000	7.17414600	-0.14114100
H	-0.86541000	7.46182000	-0.42749700

H	-1.38396900	6.17684600	0.72497400
C	-1.97438500	6.32518600	-2.72110500
H	-1.13552400	6.96649400	-3.06912200
H	-2.84592600	6.99994600	-2.62124300
H	-2.17927600	5.56983700	-3.51003500
C	-6.41685200	5.65497100	0.85340900
H	-5.53762300	6.23978900	1.19989900
H	-7.23930500	5.83194400	1.58033400
H	-6.78622500	6.05520800	-0.11041100
C	-7.28657400	3.41237200	0.14631900
H	-8.21894100	3.65826300	0.69941900
H	-7.15615500	2.31115600	0.18641600
H	-7.42509400	3.70441900	-0.91495100
C	-5.88798100	3.65956700	2.22435900
H	-6.75896800	3.93965200	2.85594400
H	-4.97580300	4.11516000	2.66751500
H	-5.79583300	2.55635700	2.29413100
C	-7.28457700	-1.59178300	-1.16548900
C	-4.67235600	-2.85774900	3.18867200
C	-7.93492600	-0.24527500	-1.54923000
H	-8.76489500	-0.40434700	-2.27141800
H	-7.20188200	0.44019000	-2.02407100
H	-8.35267200	0.26256600	-0.65726000
C	-6.73259500	-2.25828700	-2.44719800
H	-6.01758100	-1.60353800	-2.98685500
H	-7.55130800	-2.49748800	-3.16018100
H	-6.21388900	-3.19980800	-2.18343000
C	-8.38482100	-2.52981600	-0.61487200
H	-8.98678100	-2.03964200	0.17507400
H	-7.94213900	-3.47082900	-0.22274000
H	-9.10519200	-2.80562900	-1.41553100
C	-5.48699100	-2.26872300	4.36223500
H	-5.20173100	-2.74808300	5.32400200
H	-6.57329600	-2.44856800	4.25038000
H	-5.29957300	-1.17736900	4.45493200
C	-5.15531800	-4.29524900	2.89279500
H	-6.23209800	-4.32065900	2.62753600
H	-5.01809800	-4.94967900	3.78088900
H	-4.58034100	-4.72908400	2.04596700
C	-3.20462700	-2.96427200	3.67172900
H	-2.82815500	-1.97106400	3.99122000
H	-2.55104100	-3.36911700	2.87425500
H	-3.11802500	-3.64527300	4.54639500
C	-0.16635800	5.11474500	-1.51610100
H	0.53966500	5.95589900	-1.68562200
H	-0.06664000	4.43104900	-2.38463700
H	0.15232200	4.59338600	-0.58732700
C	-5.30296800	5.88314000	-2.34903000
H	-4.67186100	5.50353100	-3.18038600
H	-5.69317200	6.88236300	-2.63048600
H	-6.16296300	5.19612000	-2.21630600
C	4.20723200	2.09620200	0.51636800
H	3.46132900	1.71198200	-0.18061600
C	4.30374400	3.55334200	0.60141400
C	5.38802300	4.19014900	1.23518700
C	5.47406100	5.56414300	1.26206400
C	4.49060300	6.35509600	0.63552500
C	3.40713100	5.73109000	0.00005900
C	3.32551200	4.34496600	-0.00918100
H	6.17491600	3.59516400	1.68080900
H	6.31221600	6.06402000	1.72963700
H	2.66175700	6.31620700	-0.51740900
H	2.50711400	3.87210900	-0.53864000
O	4.68296700	7.67803400	0.69232600
C	3.79287600	8.56288300	-0.00238000
H	2.77937500	8.49530900	0.40051900
H	3.78886700	8.34849100	-1.07302900

H	4.18473700	9.56076900	0.16823000
O	5.35411300	1.42507900	0.54840800
C	5.49316800	0.20181500	-0.27642500
H	4.74677600	0.27138300	-1.06906200
H	5.25551600	-0.65126300	0.35236900
C	6.88491000	0.12191100	-0.81061800
C	7.74831900	-0.87827200	-0.36385900
C	7.32693400	1.02784600	-1.77937300
C	9.03442200	-0.97982700	-0.88354700
H	7.41819600	-1.58578600	0.38762600
C	8.61183800	0.93042000	-2.29350000
H	6.66460800	1.80770800	-2.13786600
C	9.46638900	-0.07597700	-1.84730300
H	9.69656600	-1.76240200	-0.53620700
H	8.94746700	1.63119200	-3.04715400
H	10.46663000	-0.15497900	-2.25367300

(k) Transition state **TS1-A1**, from **R1** and the electrophile with two benzyls in the position **A1**

Ni	1.06771300	0.43871600	-0.75800900
S	2.01181700	2.02720000	-2.00409500
C	0.76487200	3.01952700	-2.59907500
O	1.13289500	4.02138700	-3.35397300
C	-0.03110800	4.82261000	-3.75413400
C	-1.22703900	3.95730300	-3.35649700
N	-0.58131800	2.93750100	-2.47416100
C	-1.28132200	1.87324100	-1.89204400
O	-0.67504700	1.00033200	-1.20945500
H	0.05651100	5.00112000	-4.82146300
H	0.02268500	5.75788100	-3.20252300
H	-1.68118200	3.45256100	-4.20920000
H	-1.97427500	4.51441800	-2.79793800
C	-2.69767700	1.84853100	-2.00543300
H	-3.15299500	2.54961400	-2.69209300
N	-3.24037800	0.53549600	-1.98870500
N	-4.30106100	0.39373200	-2.61498100
N	-5.24906700	0.13065100	-3.16161000
P	2.98291900	0.07011400	0.39042600
P	0.02713200	-1.48468500	-0.10920000
C	2.68403200	-0.48921100	2.11321000
C	3.92442300	1.64379000	0.49270600
C	4.14277100	-1.15915700	-0.27911200
C	1.10629600	-2.91034500	0.23384500
C	-1.11872700	-1.97533300	-1.45676300
C	-0.94870100	-1.29542700	1.42066200
C	1.99623100	-2.94505800	1.43954600
C	1.04488900	-4.05330900	-0.58484900
C	2.08328400	-1.83237400	2.42533600
C	1.75382400	-2.07635000	3.77040000
C	1.76930900	-5.20087000	-0.24993900
C	2.53413800	-5.21028800	0.91495500
C	2.64836600	-4.16417800	1.70061800
C	1.99939600	-1.21568100	4.72913000
C	2.62366200	0.00537900	4.50253500
O	3.17218500	-6.25554200	1.36998900
O	3.38683500	-4.41676600	2.75182800
O	1.20649900	-3.17321500	4.22463900
O	1.63874300	-1.65144000	5.90611800
H	0.42257600	-4.07792500	-1.46765400
H	1.70453500	-6.08602900	-0.86949900
H	2.83467600	0.67320000	5.32795500
H	3.47846700	1.30511300	3.06760800
H	5.62072200	0.93516400	-0.58991800
H	2.27216100	2.64083900	1.34352800
H	2.91096900	-1.56059000	-1.94465600
H	5.43680100	-1.01466400	1.40562600
H	-2.52658100	-2.98106900	-0.20612800

H	0.06505000	-0.95895600	-2.90179200
H	-1.36093100	-3.37228100	1.60315800
H	-0.40217200	0.73564300	1.57426700
H	0.03299500	-2.98239100	5.94956400
H	1.67512600	-3.72186600	6.18742400
H	3.32238600	-6.38621500	3.45000200
H	4.84074800	-5.92240800	2.58404900
C	2.98024500	0.36129200	3.20003500
C	3.28659500	2.77480400	1.00136500
C	1.09766500	-2.94891100	5.63688400
C	3.73945300	-5.79937800	2.60370900
C	3.94379900	4.02225600	1.08879400
C	5.29442700	4.08657600	0.63728200
C	5.85119400	3.01922100	-0.12790600
C	5.17927100	1.78328300	-0.10082600
C	3.86540000	-1.78464700	-1.49358000
C	4.80442400	-2.63077100	-2.12828100
C	6.08462800	-2.75410400	-1.52288200
C	6.28433700	-2.30671300	-0.10299600
C	5.30445500	-1.45814800	0.42827900
C	-2.28751100	-2.69545900	-1.20653500
C	-3.19083200	-3.02298100	-2.22686700
C	-2.76548600	-2.71186300	-3.63166300
C	-1.66657800	-1.83761000	-3.85726500
C	-0.83275900	-1.54388900	-2.75598000
C	-0.97534500	-0.04938000	2.04217300
C	-1.71453900	0.18394100	3.22192200
C	-2.54837200	-0.86673700	3.68875400
C	-2.32986200	-2.27000900	3.18815100
C	-1.53716300	-2.40156200	2.03664200
O	-3.64802300	-0.55758600	4.49943500
O	7.18084200	-3.13616900	-2.30142100
O	-3.31013000	-3.44432400	-4.68842600
O	6.10153200	5.15500300	1.04824500
C	-2.63399200	-4.68273400	-4.90774300
H	-3.11805900	-5.21096700	-5.75420100
H	-2.69479000	-5.33151100	-4.00933000
H	-1.56815900	-4.50941800	-5.16675900
C	7.95858600	-2.01936600	-2.74279300
H	8.79566300	-2.38920500	-3.36915000
H	8.38411600	-1.46067900	-1.88593300
H	7.34382300	-1.32276900	-3.35164600
C	-4.89937000	-0.53785500	3.79968100
H	-4.80959100	-0.84475700	2.73467500
H	-5.32952200	0.48272200	3.82860600
H	-5.61815700	-1.21404000	4.30387900
C	-1.33020400	-1.19361500	-5.23150000
C	-4.55862100	-3.63898400	-1.83948100
C	-5.26328600	-2.73952900	-0.79134800
H	-6.26386100	-3.14514000	-0.52662600
H	-5.40430800	-1.71560300	-1.19089400
H	-4.69218600	-2.66427900	0.15579300
C	-5.53212200	-3.77909400	-3.03331300
H	-5.21579400	-4.58574800	-3.72561900
H	-5.62104700	-2.81906500	-3.58575800
H	-6.54896400	-4.06114000	-2.68324300
C	-4.33913300	-5.03901800	-1.22869500
H	-3.83626900	-5.70947600	-1.95619600
H	-5.31027600	-5.50035200	-0.94619400
H	-3.70977700	-4.99064200	-0.31616700
C	0.04032000	-1.70026900	-5.72318600
H	0.25659800	-1.32553600	-6.74722100
H	0.06519600	-2.81005000	-5.74406100
H	0.85418300	-1.34096600	-5.06398800
C	-2.37231500	-1.47558700	-6.34204100
H	-2.32351800	-2.52759500	-6.69032300
H	-2.17182100	-0.85268300	-7.24097000

H	-3.39924700	-1.23323400	-5.99254600
C	-1.26546800	0.34550500	-5.07493300
H	-2.23445500	0.73664600	-4.69948800
H	-0.46209300	0.66014000	-4.37896700
H	-1.04996500	0.83719400	-6.04834100
C	-1.61895200	1.53678800	3.97673000
C	-2.93572100	-3.53335200	3.85570900
C	-0.49558300	2.45267200	3.42688000
H	-0.72307600	2.78759000	2.39419300
H	0.48414300	1.92904500	3.43623000
H	-0.39046900	3.36923800	4.04732800
C	-2.94600000	2.32001600	3.87392900
H	-2.80365700	3.38653000	4.15353700
H	-3.71008200	1.92455200	4.56958800
H	-3.34166200	2.28595900	2.83673300
C	-1.28405900	1.27147600	5.46294800
H	-2.08241000	0.69464800	5.97211400
H	-1.16621900	2.22718300	6.01845700
H	-0.33574300	0.69791500	5.54859400
C	-4.18496900	-3.97616700	3.07032400
H	-4.96604900	-3.19258200	3.07370000
H	-4.62342500	-4.89484400	3.51691800
H	-3.92325000	-4.19587400	2.01405000
C	-3.31747200	-3.28966600	5.33715200
H	-3.52029600	-4.24688800	5.86435400
H	-4.24787000	-2.70190900	5.43903200
H	-2.49390300	-2.77230400	5.87414000
C	-1.93115600	-4.71692400	3.84870000
H	-0.96788000	-4.41714800	4.29787200
H	-1.74605100	-5.11055800	2.82857300
H	-2.32323000	-5.57774000	4.43267100
C	3.19427000	5.29128900	1.58458500
C	7.12353800	3.14623300	-1.01094100
C	3.18437800	6.34047300	0.45090400
H	4.21246200	6.66058800	0.18227600
H	2.61940100	7.24811600	0.75593800
H	2.70728400	5.91981000	-0.46070800
C	3.86922500	5.89075300	2.83965600
H	3.18167900	6.57853800	3.37851000
H	4.75107600	6.50673000	2.57837300
H	4.17033600	5.08557000	3.54400200
C	7.54753200	4.61306500	-1.28367600
H	6.67294000	5.22332500	-1.59669100
H	8.30552400	4.66818100	-2.09486400
H	8.03557300	5.08032000	-0.40984000
C	8.28923800	2.40048400	-0.33438500
H	9.21094900	2.47101800	-0.95161200
H	8.04590700	1.32509100	-0.19931000
H	8.50480400	2.83235400	0.66467900
C	6.86759800	2.51164900	-2.40684700
H	7.71299800	2.70828500	-3.10132100
H	5.94255100	2.93117600	-2.85862800
H	6.77281700	1.40755000	-2.36227900
C	7.43215600	-2.78542900	0.81635800
C	4.45319800	-3.39730500	-3.43353800
C	8.30369600	-1.58504000	1.24375600
H	9.13643800	-1.92069200	1.89915000
H	7.71380000	-0.83347300	1.80904000
H	8.74468300	-1.08192400	0.35968100
C	6.82634000	-3.43838600	2.07922800
H	6.23343700	-2.71871400	2.68093500
H	7.62093500	-3.84078900	2.74440400
H	6.16414300	-4.27383200	1.77957900
C	8.34747500	-3.84751300	0.16214000
H	8.98636300	-3.40902000	-0.62923700
H	7.74700400	-4.68343100	-0.25733600
H	9.04661700	-4.27995900	0.91064600

C	5.29445400	-2.89084600	-4.62679600
H	4.88976300	-3.27383800	-5.58893400
H	6.34214000	-3.24480700	-4.58140700
H	5.28056100	-1.78043700	-4.66725400
C	4.71056700	-4.90568500	-3.22088800
H	5.78043800	-5.11473600	-3.01535100
H	4.42764300	-5.48679600	-4.12539000
H	4.11317100	-5.28181500	-2.36196700
C	2.96604600	-3.24439000	-3.83630900
H	2.74468000	-2.18908400	-4.09577100
H	2.29481300	-3.57403700	-3.01825400
H	2.72598300	-3.86070900	-4.73011000
C	1.71714000	5.01109500	1.96080800
H	1.19017500	5.94747500	2.24722800
H	1.66167400	4.31800600	2.82695700
H	1.16447900	4.57533600	1.10419000
C	6.97519500	4.82681300	2.14111700
H	6.86307900	3.77670800	2.49671000
H	6.77265700	5.50092900	2.99660400
H	8.03005600	4.98608600	1.84305000
C	-3.26092300	2.77388800	-0.26642000
H	-2.55518800	2.25312900	0.37997600
C	-4.63586800	2.37242800	-0.13451200
C	-5.70624900	3.17302000	-0.59549700
C	-6.99925600	2.72507100	-0.50789700
C	-7.28149900	1.44713200	0.02928700
C	-6.22493100	0.64854600	0.50768100
C	-4.92888000	1.11218500	0.41952000
H	-5.50146100	4.15371100	-1.00459300
H	-7.83062700	3.32938300	-0.84563400
H	-6.42258400	-0.31825000	0.94480000
H	-4.11947200	0.49341100	0.78692500
O	-2.98287500	4.08477400	-0.48455100
C	-2.01820400	4.67544300	0.45028200
H	-1.09017500	4.10116100	0.38733300
H	-2.43167700	4.57065000	1.45570100
C	-1.78142900	6.11256000	0.10830700
C	-0.57781300	6.51768000	-0.47180400
C	-2.74963700	7.07593300	0.40140800
C	-0.33691200	7.85960100	-0.74919100
C	-2.51790600	8.41491800	0.11236300
C	-1.31075700	8.80937800	-0.45967800
H	0.19530800	5.78528400	-0.67626800
H	-3.68249900	6.77928200	0.86633400
H	0.61264500	8.16626000	-1.17026000
H	-3.27141100	9.15511000	0.34887000
H	-1.12553500	9.85578900	-0.66554100
O	-8.56142000	1.09712500	0.04764600
C	-8.95732300	-0.22574400	0.54118500
H	-8.64863700	-0.30321700	1.58588000
H	-8.42920100	-0.97569700	-0.05247900
C	-10.43922300	-0.36320400	0.39999300
C	-10.99256200	-0.82117300	-0.79705300
C	-11.28205100	-0.02949300	1.46085400
C	-12.37043900	-0.93637600	-0.93403700
H	-10.34448200	-1.09048600	-1.62368000
C	-12.66051900	-0.14629200	1.32551100
H	-10.86051400	0.31986400	2.39675000
C	-13.20506100	-0.59859800	0.12759400
H	-12.79341900	-1.29485900	-1.86388300
H	-13.30851800	0.11021000	2.15389500
H	-14.27851600	-0.69346900	0.02311700

(*l*) Transition state **TS1-A2**, from **R1** and the electrophile with two benzyls in the position **A2**

Ni	-0.58168400	0.51075400	0.75815700
S	-0.08982300	2.31218100	1.97715800

C	1.55707700	2.19093000	2.37630700
O	2.09598100	3.23500300	2.94989400
C	3.52951500	3.00351200	3.16590600
C	3.65666600	1.48912800	3.06968000
N	2.42690900	1.15140400	2.28748600
C	2.09580500	-0.14591800	1.87701000
O	1.03373300	-0.35724800	1.23324000
H	3.78238600	3.41823700	4.13637200
H	4.05638000	3.52944500	2.37186200
H	3.60090300	1.00018200	4.04344600
H	4.54937000	1.18162300	2.53548500
C	3.02965000	-1.19787900	2.11013800
H	3.82750000	-0.99725300	2.81429300
N	2.41144000	-2.47619900	2.22036900
N	3.00154300	-3.29329000	2.94322900
N	3.42211000	-4.12378200	3.57595300
P	-2.11519000	1.63442100	-0.45181000
P	-1.28883000	-1.57479100	0.20498400
C	-2.31561800	0.94211500	-2.13670700
C	-1.51803400	3.35807700	-0.64701500
C	-3.81607600	1.75167600	0.18170400
C	-3.07234800	-1.74990200	-0.12203100
C	-0.89656600	-2.64456600	1.64039200
C	-0.48785600	-2.27359000	-1.27699300
C	-3.70874100	-1.14927600	-1.33918600
C	-3.87452100	-2.52869100	0.73399900
C	-2.94994600	-0.39982200	-2.37461900
C	-2.94110100	-0.87298900	-3.69612900
C	-5.22140200	-2.74991900	0.43156600
C	-5.75756500	-2.20351700	-0.73185600
C	-5.06303200	-1.45728800	-1.55382700
C	-2.44859900	-0.18462600	-4.69315100
C	-1.90754600	1.08707400	-4.53765600
O	-7.03619200	-2.43267100	-1.19694800
O	-5.81255700	-1.09547800	-2.65840200
O	-3.46980300	-2.07325600	-4.12801300
O	-2.58420300	-0.84649800	-5.89599500
H	-3.46860000	-2.98361500	1.62565700
H	-5.83254700	-3.36567600	1.07838600
H	-1.53535700	1.63091100	-5.39610100
H	-1.43502300	2.64514900	-3.17613400
H	-3.19468000	4.26610700	0.31219800
H	0.35751000	2.67763700	-1.33904300
H	-3.36090600	0.61979800	1.90383300
H	-4.50731700	2.75844000	-1.56254500
H	-0.69666100	-4.46049500	0.53671500
H	-0.93143900	-0.96755200	2.94659500
H	-1.74408600	-3.98414600	-1.32481500
H	0.64603500	-0.51792700	-1.55813800
H	-2.59867900	-2.93275900	-5.82547900
H	-4.21186800	-2.14380800	-6.08082900
H	-7.25219400	-2.48081000	-3.27467700
H	-7.89888900	-1.00648500	-2.45359900
C	-1.85096600	1.65568800	-3.26192600
C	-0.20912800	3.56019100	-1.08420500
C	-3.24157500	-2.07241100	-5.54582600
C	-7.07648500	-1.74931600	-2.45783000
C	0.35397000	4.85199400	-1.16754400
C	-0.49509000	5.95852500	-0.87433000
C	-1.73609200	5.75607300	-0.19893500
C	-2.24472900	4.44408100	-0.15782500
C	-4.14134100	1.17800900	1.41002300
C	-5.41460700	1.35242900	2.00102000
C	-6.33040100	2.21306200	1.33585200
C	-6.08169500	2.60683200	-0.09267700
C	-4.78343800	2.40467900	-0.57865900
C	-0.66899300	-4.01430100	1.50766400

C	-0.36797000	-4.82478500	2.61220000
C	-0.44169300	-4.17974500	3.96589800
C	-0.48954200	-2.76237200	4.06774600
C	-0.78334900	-2.03742500	2.89404300
C	0.44408300	-1.49717300	-1.96324200
C	1.08591600	-1.95509500	-3.13594400
C	0.85136500	-3.30208400	-3.52452300
C	-0.32091700	-4.05093000	-2.94733900
C	-0.91721100	-3.48993500	-1.80723900
O	1.77175200	-3.94780500	-4.35978900
O	-7.36538700	2.81021100	2.06126300
O	-0.68362800	-4.97225500	5.08989200
O	-0.12356000	7.23269300	-1.31312800
C	-2.07272200	-5.23662200	5.28912900
H	-2.19170900	-5.86998100	6.19165500
H	-2.50049300	-5.78028400	4.42081500
H	-2.63491400	-4.29211900	5.44760100
C	-7.05278800	4.14493800	2.47061700
H	-7.90188600	4.54889500	3.05856300
H	-6.88744900	4.80588600	1.59740500
H	-6.14283500	4.16285300	3.10744400
C	2.65619400	-4.84377500	-3.67333900
H	2.45140600	-4.91486000	-2.58167800
H	3.70228500	-4.50885400	-3.80979900
H	2.58065400	-5.85664300	-4.11573500
C	-0.24949900	-1.96939500	5.38231200
C	0.04969300	-6.29508200	2.36290800
C	1.24340700	-6.33708200	1.37732500
H	1.57902600	-7.38236900	1.20286600
H	2.09926400	-5.76593900	1.79031200
H	0.98780500	-5.91503800	0.38391600
C	0.50287800	-7.03567100	3.64300300
H	-0.35285600	-7.24912000	4.31534700
H	1.27613400	-6.45030500	4.18585300
H	0.94565100	-8.02400800	3.39147500
C	-1.13438600	-7.07253000	1.74987800
H	-2.01416200	-7.04133800	2.42617600
H	-0.86105400	-8.13727200	1.58438300
H	-1.43776300	-6.64763900	0.77048400
C	-1.53302900	-1.21020800	5.77613000
H	-1.41155200	-0.71408900	6.76351100
H	-2.39702100	-1.90482900	5.83795700
H	-1.76894200	-0.41786400	5.03824700
C	0.17895400	-2.84459300	6.58589100
H	-0.66502400	-3.45517400	6.96729300
H	0.50245200	-2.21136200	7.44065500
H	1.03510700	-3.49948800	6.31468900
C	0.88863100	-0.94261600	5.16018400
H	1.82281500	-1.46000700	4.85602800
H	0.63039600	-0.19492500	4.38332900
H	1.09834000	-0.37246100	6.09113000
C	1.98202200	-1.01001500	-3.98423000
C	-0.88398500	-5.37175500	-3.53590100
C	1.92921700	0.46142600	-3.49997500
H	2.37533400	0.56183900	-2.48823500
H	0.88493800	0.83709400	-3.48079400
H	2.50834400	1.12612900	-4.17750100
C	3.46135400	-1.44751400	-3.93882700
H	4.11926600	-0.66235500	-4.37084300
H	3.64289900	-2.35624400	-4.54053000
H	3.78495000	-1.62231800	-2.89262400
C	1.48732600	-1.01505400	-5.44892200
H	1.58657800	-2.01563600	-5.91622800
H	2.07654300	-0.30470200	-6.06864900
H	0.41841100	-0.71592600	-5.49667700
C	-0.35033200	-6.56088900	-2.71428600
H	0.75365800	-6.62153600	-2.76000100

H	-0.76107800	-7.51921300	-3.09966000
H	-0.64294600	-6.46125900	-1.64713200
C	-0.50798300	-5.56043800	-5.02701700
H	-1.10814500	-6.36997700	-5.49580100
H	0.54359700	-5.87129900	-5.15964100
H	-0.69143300	-4.62535000	-5.59837100
C	-2.43502000	-5.40647600	-3.46853300
H	-2.86628900	-4.49826200	-3.92778500
H	-2.81260500	-5.50017900	-2.42987600
H	-2.84105900	-6.28766800	-4.01102200
C	1.86525000	5.05030900	-1.47807000
C	-2.51563600	6.85745300	0.57440000
C	2.51967000	5.87033900	-0.34213800
H	2.09794800	6.89318800	-0.27202800
H	3.61321400	5.97869400	-0.51136900
H	2.36682300	5.36421500	0.63612100
C	2.06848700	5.77599200	-2.82597200
H	3.12887100	5.71483600	-3.15451600
H	1.83779900	6.85617600	-2.75614200
H	1.43730500	5.31283300	-3.61465300
C	-1.80105500	8.23057700	0.63444500
H	-0.75656500	8.11847200	0.99718700
H	-2.32304400	8.91754000	1.33570700
H	-1.81230900	8.74731100	-0.34456900
C	-3.90499900	7.06170200	-0.06669000
H	-4.46596300	7.86459000	0.45904400
H	-4.51613800	6.13720400	-0.01214500
H	-3.80784300	7.34776400	-1.13418300
C	-2.70533000	6.40610300	2.04672100
H	-3.19406300	7.20162600	2.65024300
H	-1.72095200	6.17419900	2.50846000
H	-3.35200100	5.50915800	2.13462000
C	-7.16697500	3.12931000	-1.06396600
C	-5.80151600	0.62974800	3.32159900
C	-6.82643200	4.56580000	-1.51512700
H	-7.59688800	4.94664300	-2.22013000
H	-5.84553200	4.60818600	-2.03342900
H	-6.78755200	5.25496600	-0.64797500
C	-7.21487900	2.21532400	-2.31013400
H	-6.26675700	2.24038400	-2.88606500
H	-8.02375800	2.52695600	-3.00613500
H	-7.40669100	1.17086700	-1.99725500
C	-8.59030400	3.12978900	-0.45744500
H	-8.71060500	3.91930100	0.31000900
H	-8.83401400	2.13771400	-0.01966900
H	-9.35142400	3.34854500	-1.23776300
C	-6.00469600	1.63776300	4.47498100
H	-6.05706500	1.11491500	5.45480700
H	-6.95679700	2.19440000	4.38213700
H	-5.15824300	2.35660100	4.51512100
C	-7.10336700	-0.17205400	3.09862400
H	-7.95664900	0.49069800	2.84749900
H	-7.38232100	-0.73677600	4.01466400
H	-6.97217800	-0.89749200	2.26641200
C	-4.72476600	-0.37923000	3.79200800
H	-3.78902400	0.15056600	4.06331400
H	-4.51100300	-1.12949400	3.00529300
H	-5.06130500	-0.93405100	4.69492300
C	2.64285700	3.71229100	-1.56111000
H	3.72808100	3.89317900	-1.71550900
H	2.29227300	3.10688400	-2.42260200
H	2.53297300	3.12865000	-0.62093000
C	-0.76332800	7.59809900	-2.54032100
H	-0.51464700	6.87893200	-3.34934700
H	-0.41155100	8.60522200	-2.84345100
H	-1.86546000	7.63236100	-2.42441500
C	4.12586200	-1.31271300	0.40786200

H	3.31218200	-1.14388700	-0.29891700
C	5.14569400	-0.26539100	0.46046500
C	6.36421100	-0.45158000	1.14179200
C	7.31826400	0.54042500	1.15451600
C	7.10569000	1.75186600	0.46277600
C	5.89719900	1.94215900	-0.22683500
C	4.93623000	0.93974500	-0.21797300
H	6.56339500	-1.39268300	1.63856700
H	8.26417200	0.40162300	1.66154300
H	5.72672700	2.84193900	-0.79808600
H	4.02507900	1.08511700	-0.78623100
O	8.10452400	2.63580900	0.51763100
C	8.03059600	3.87176400	-0.25847900
H	7.16800600	4.44719500	0.08842700
H	7.88673400	3.60369700	-1.30807400
C	9.30019300	4.63828700	-0.06120900
C	9.37854600	5.63816900	0.90874000
C	10.42122600	4.35527200	-0.84367700
C	10.56154800	6.34303000	1.09895400
C	11.60511300	5.05689300	-0.65287100
C	11.67592200	6.05140000	0.31866600
H	8.51034000	5.87174100	1.51535000
H	10.36846600	3.58560700	-1.60544200
H	10.61335200	7.12118900	1.84982500
H	12.46971900	4.83421300	-1.26505600
H	12.59667700	6.60233400	0.46282000
O	4.56599000	-2.56757200	0.47731300
C	3.87815600	-3.61540300	-0.31289000
H	3.36613600	-3.10841100	-1.13158400
H	3.13569500	-4.08064900	0.32921200
C	4.88594100	-4.60333000	-0.79942800
C	4.95373100	-5.87481000	-0.22957400
C	5.76149800	-4.26833300	-1.83630900
C	5.87946400	-6.80345500	-0.69283300
H	4.28145600	-6.14578800	0.57595200
C	6.68800100	-5.19322900	-2.29619900
H	5.71418100	-3.28508000	-2.29088900
C	6.74637300	-6.46289100	-1.72511700
H	5.92266700	-7.78997500	-0.24959200
H	7.35986500	-4.93099800	-3.10336900
H	7.46565600	-7.18600100	-2.08801300

10. References

[1] Teloxa, S. F.; Kennington, S. C. D.; Camats, M.; Romea, P.; Urpí, F.; Aullón, G.; Font-Bardia, M. Direct, enantioselective, and nickel(II) catalyzed reactions of *N*-azidoacetyl thioimides with trimethyl orthoformate: a new combined methodology for the rapid synthesis of lacosamide and derivatives. *Chem. Eur. J.* **2020**, *26*, 11540–11548.

[2] Teloxa, S. F.; Mellado-Hidalgo, M.; Kennington, S. C. D.; Romea, P.; Urpí, F.; Aullón, G.; Font-Bardia, M. Direct and asymmetric aldol reactions of *N*-azidoacetyl-1,3-thiazolidine-2-thione catalyzed by chiral nickel(II) complexes. A new approach to the synthesis of β-hydroxy-α-amino acids. *Chem. Eur. J.* **2022**, *28*, e202200671.

[3] X-Ray of amide **9** had been previously reported, see: Fernández-Valparis, J.; Romea, P.; Urpí, F.; Font-Bardia, M. Stereoselective and Catalytic Synthesis of *anti*-β-Alkoxy-α-azido Carboxylic Derivatives. *Org. Lett.* **2017**, *19*, 6400–6403.

[4] Frisch, M. J.; Trucks, G. W.; Schlegel, H. B.; Scuseria, G. E.; Robb, M. A.; Cheeseman, J. R.; Scalmani, G.; Barone, V.; Mennucci, B.; Petersson, G. A.; Nakatsuji, H.; Caricato, M.; Li, X.; Hratchian, H. P.; Izmaylov, A. F.; Bloino, J.; Zheng, G.; Sonnenberg, J. L.; Hada, M.; Ehara, M.; Toyota, K.; Fukuda, R.; Hasegawa, J.; Ishida, M.; Nakajima, T.; Honda, Y.; Kitao, O.; Nakai, H.; Vreven, T.; Montgomery, Jr., J. A.; Peralta, J. E.; Ogliaro, F.; Bearpark, M.; Heyd, J. J.; Brothers, E.; Kudin, K. N.; Staroverov, V. N.; Keith, T.; Kobayashi, R.; Normand, J.; Raghavachari, K.; Rendell, A.; Burant, J. C.; Iyengar, S. S.; Tomasi, J.; Cossi, M.; Rega, N.; Millam, J. M.; Klene, M.; Knox, J. E.; Cross, J. B.; Bakken, V.; Adamo, C.; Jaramillo, J.; Gomperts, R.; Stratmann, R. E.; Yazyev, O.; Austin, A. J.; Cammi, R.; Pomelli, C.; Ochterski, J. W.; Martin, R. L.; Morokuma, K.; Zakrzewski, V. G.; Voth, G. A.; Salvador, P.; Dannenberg, J. J.; Dapprich, S.; Daniels, A. D.; Farkas, O.; Foresman, J. B.; Ortiz, J. V.; Cioslowski, J.; Fox, D. J. *Gaussian 09 (Revission B.1)*; Gaussian Inc.: Wallingford CT 2010.

[5] (a) Maseras, F.; Morokuma, K. IMOMM: A new integrated *ab initio* + molecular mechanics geometry optimization scheme of equilibrium structures and transition states *J. Comput. Chem.* **1995**, *16*, 1170–1179. (b) Dapprich, S.; Komáromi, I.; Byun, K. S.; Morokuma, K.; Frisch, M. J A new ONIOM implementation in Gaussian98. Part I. The calculation of energies, gradients, vibrational frequencies, and electric field derivatives. *J. Mol. Struct.–Theochem.* **1999**, *462*, 1–21.

[6] (a) Becke, A. D. Density-functional thermochemistry. III. The role of exact exchange. *J. Chem. Phys.* **1993**, *98*, 5648–5652. (b) Lee, C.; Yang, W.; Parr, R. G. Development of the Colle-Salvetti correlation-energy formula into a functional of the electron density. *Phys. Rev. B*, **1988**, *37*, 785–789.

[7] Schäfer, A.; Huber, C.; Ahlrichs, R. Fully optimized contracted Gaussian basis set of triple zeta valence quality for atoms Li to Kr. *J. Chem. Phys.* **1994**, *100*, 5829–5835.

[8] Tomasi, J. ; Mennucci, B. ; Cammi, R. Quantum Mechanical Continuum Solvation Models. *Chem. Rev.* **2005**, *105*, 2999–3094.

[9] Llunell, M.; Casanova, D.; Cirera, J.; Alemany, P.; Alvarez, S. *SHAPE (version 2.0)*, Barcelona, 2010.

11. Copies of NMR Spectra and HPLC Chromatograms

^{1}H NMR (400 MHz, $CDCl_3$)

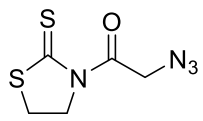

3

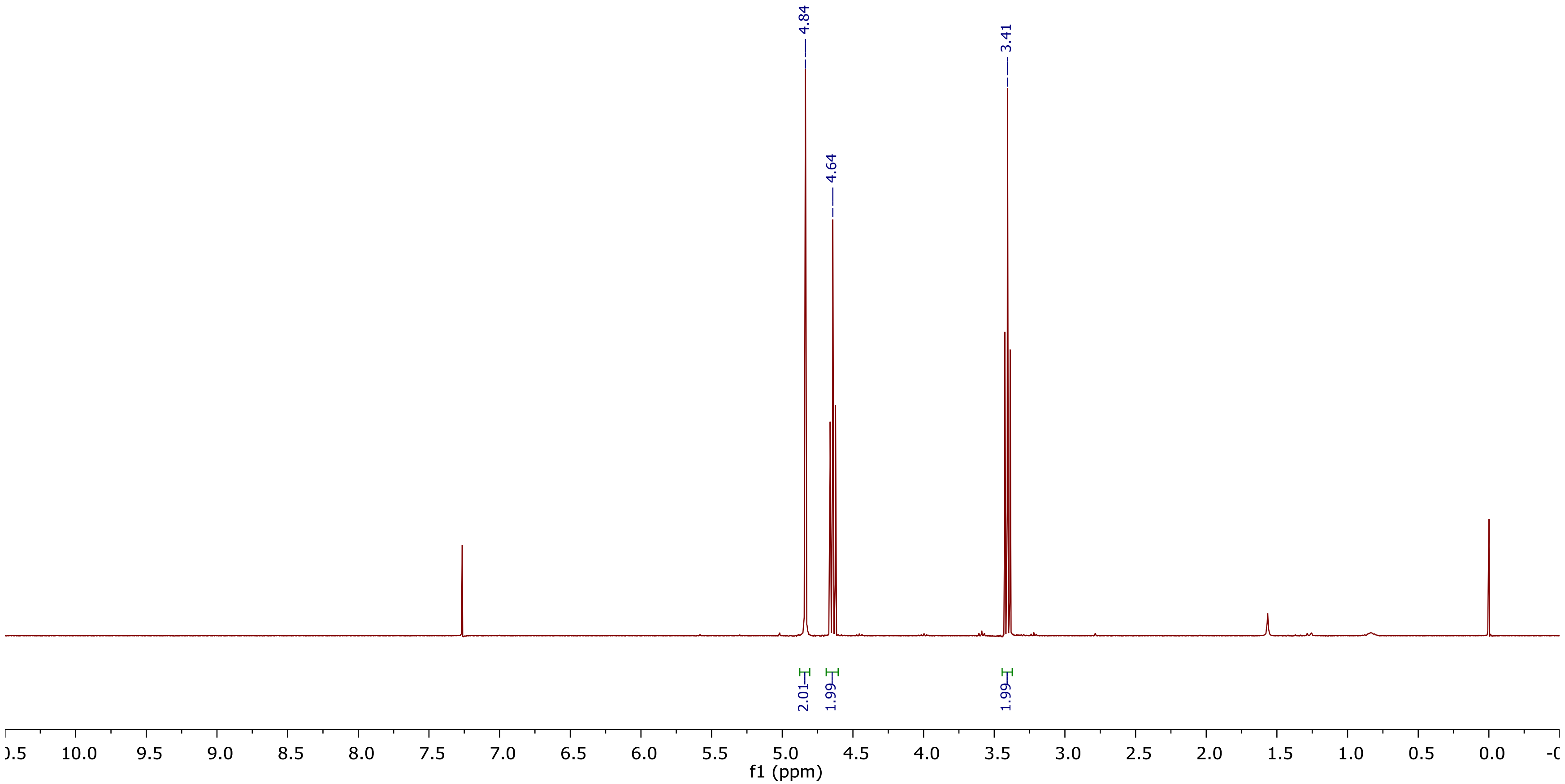

^{13}C NMR (100.6 MHz, $CDCl_3$)

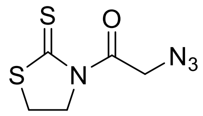

3

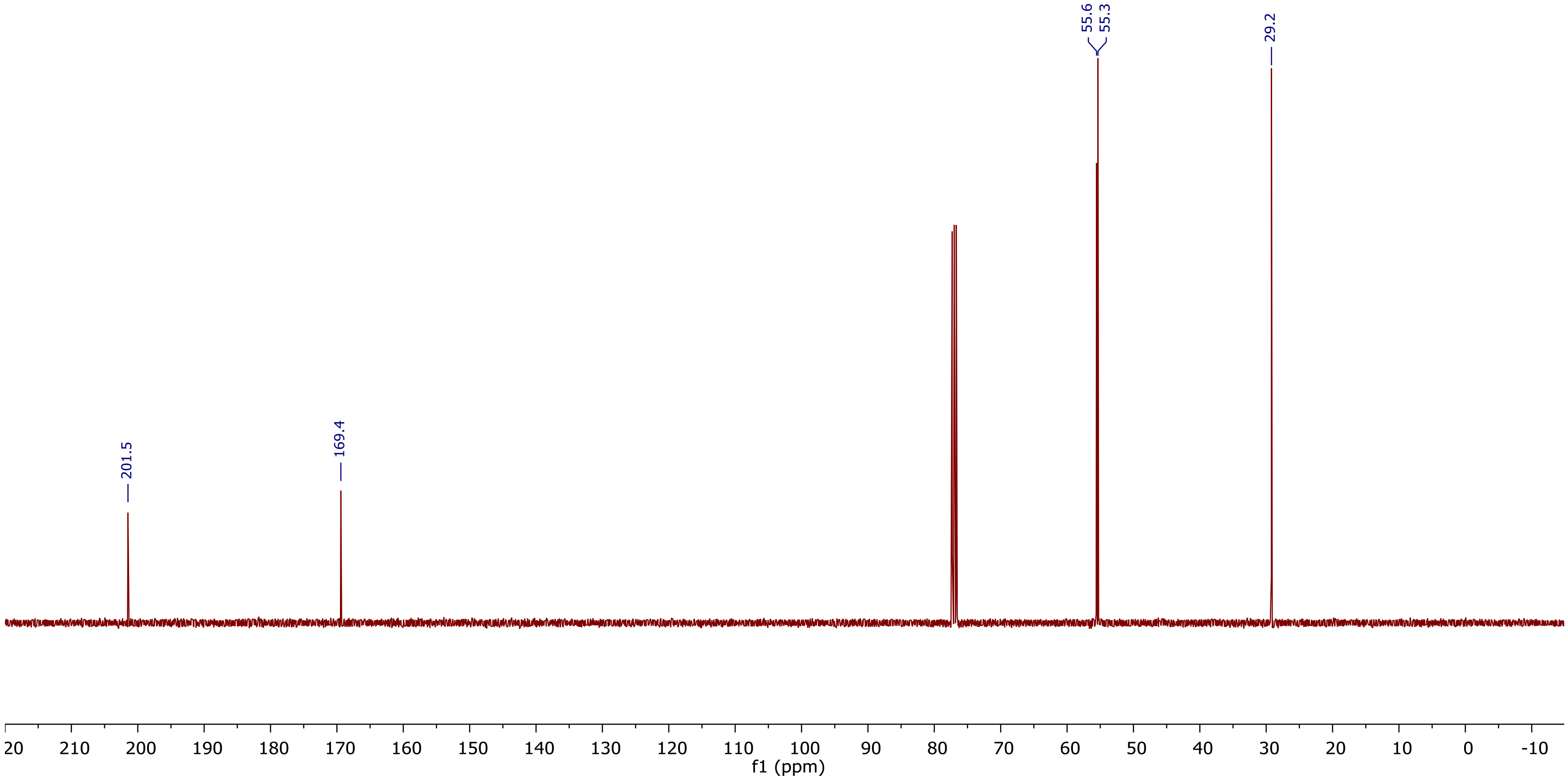

^1H NMR (400 MHz, $CDCl_3$)

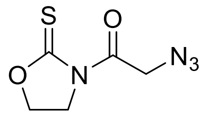

4

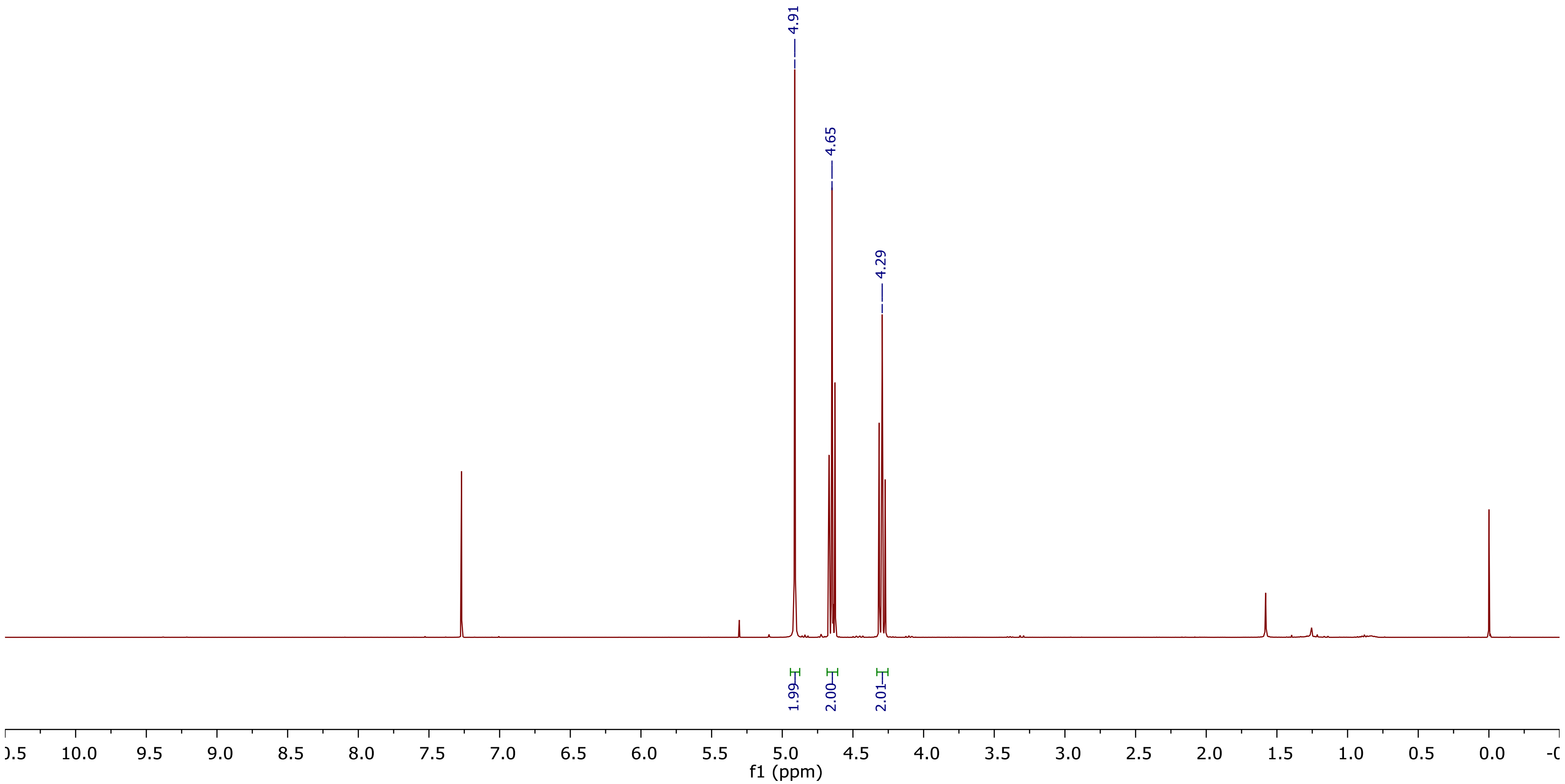

^{13}C NMR (100.6 MHz, $CDCl_3$)

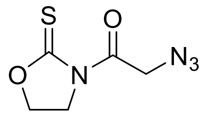

4

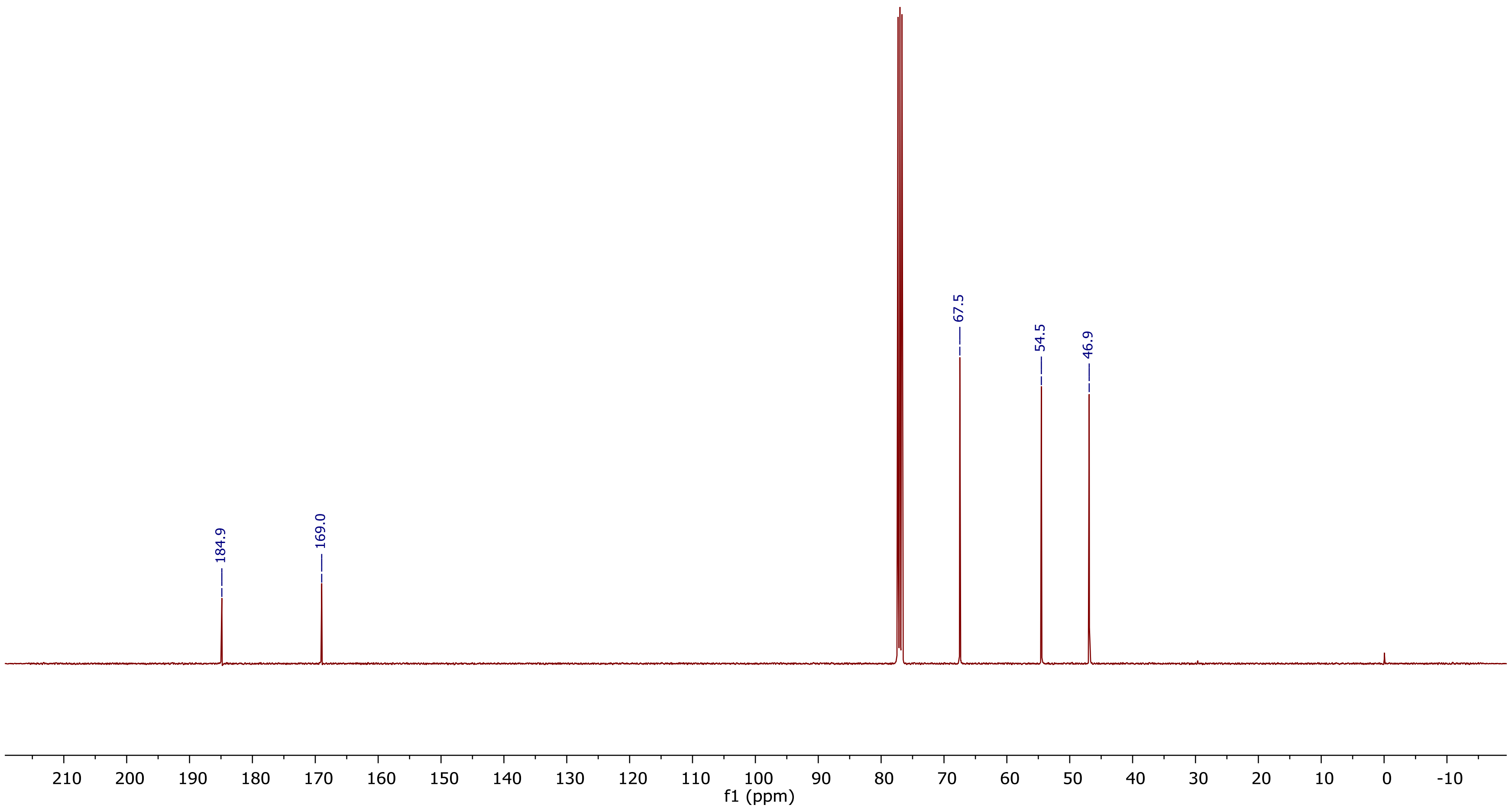

^{1}H NMR (400 MHz, $CDCl_3$)

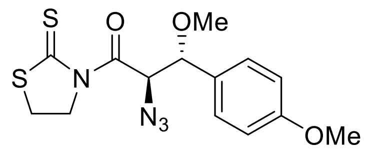

5a

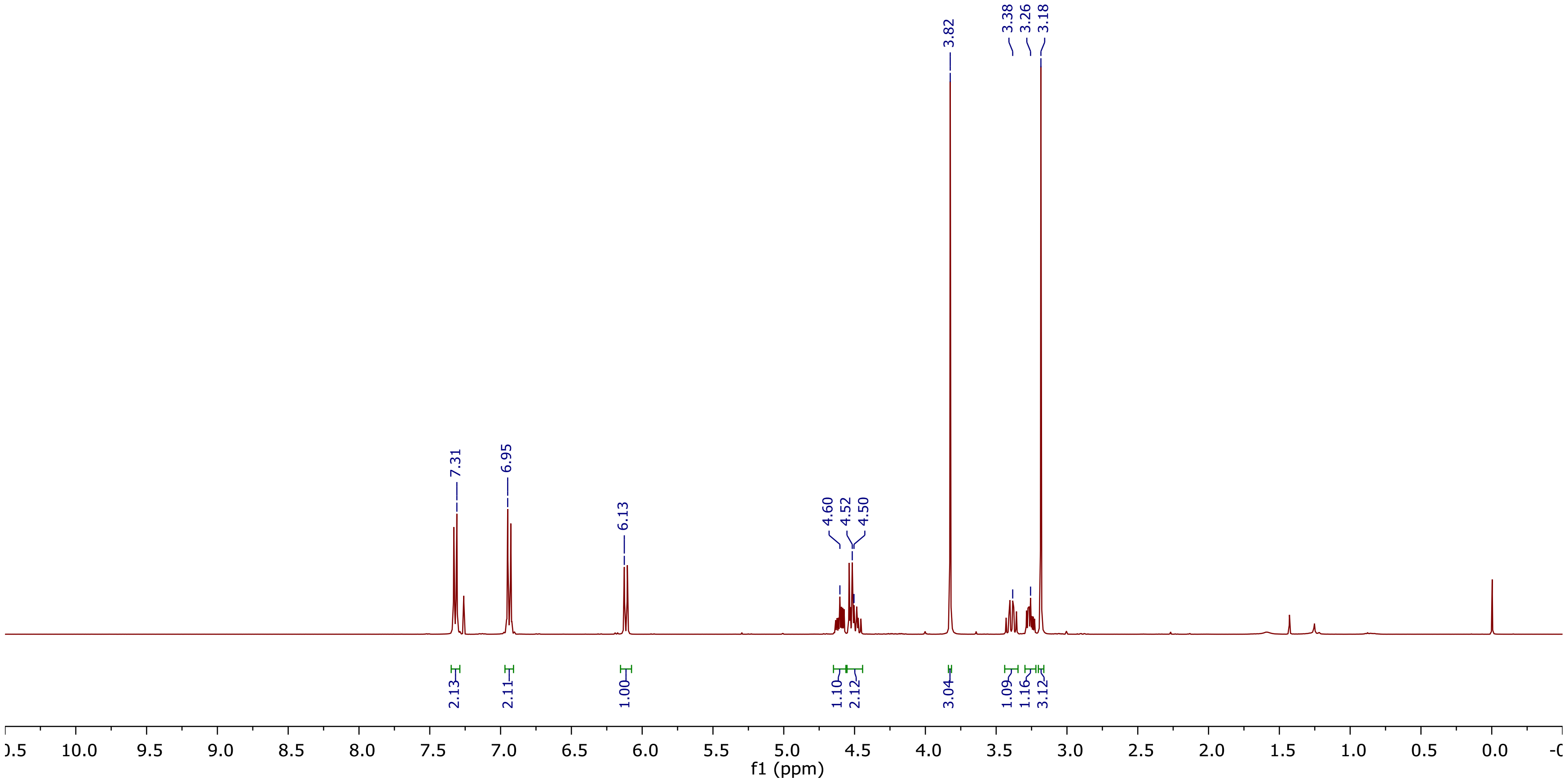

$^{13}C\{^{1}H\}$ NMR (100.6 MHz, $CDCl_3$)

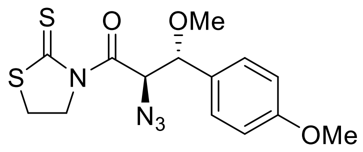

5a

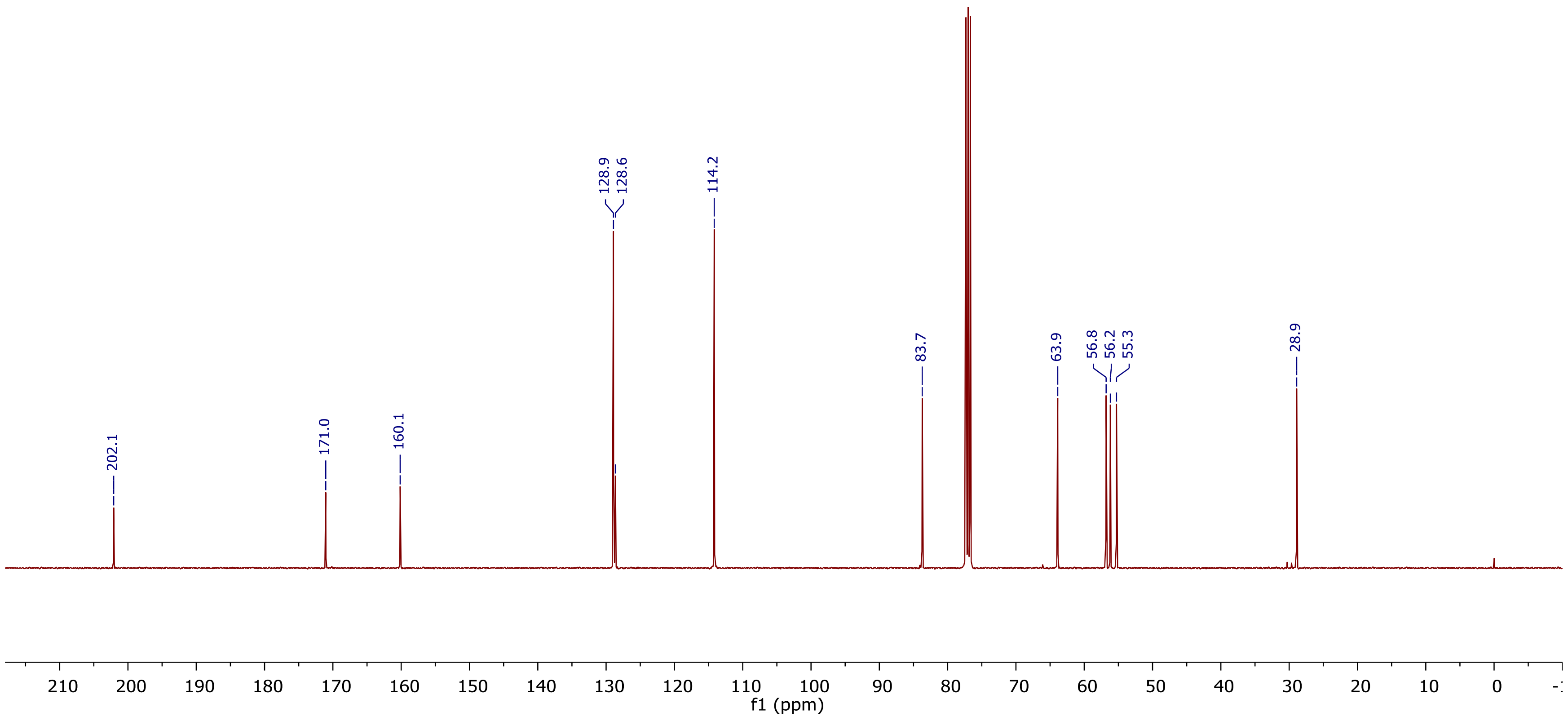

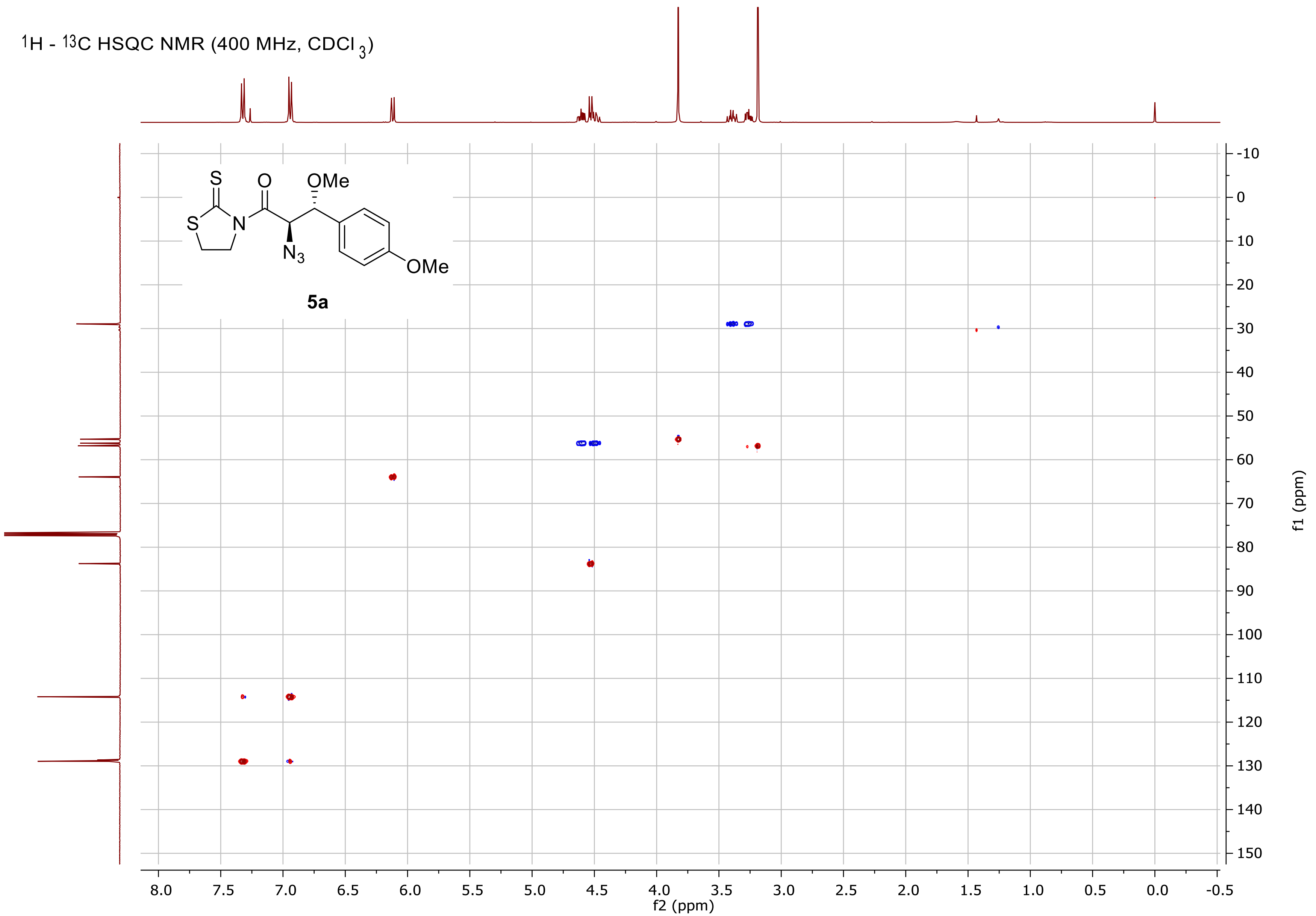

^1H - ^{13}C HSQC NMR (400 MHz, $CDCl_3$)
S
O
OMe
N
S
N_3
OMe
5a
f2 (ppm)
f1 (ppm)

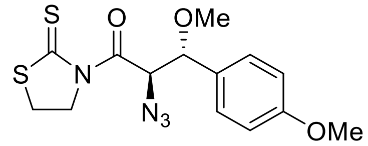

5a

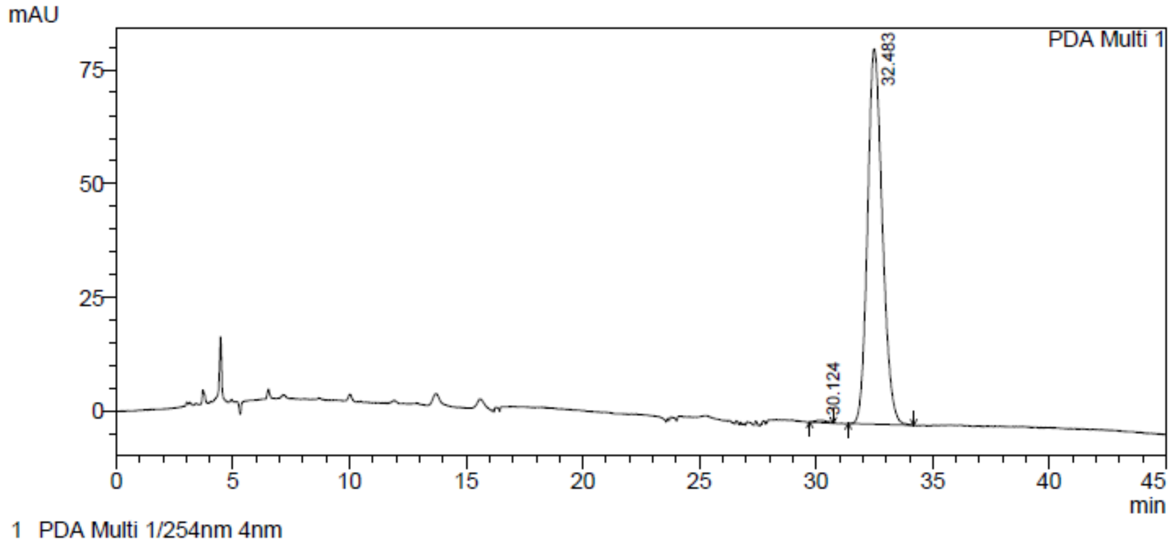

PeakTable

PDA Ch1 254nm 4nm

Peak#	Ret. Time	Area	Height	Area %	Height %
1	30.124	17085	534	0.468	0.644
2	32.483	3636746	82388	99.532	99.356
Total		3653831	82921	100.000	100.000

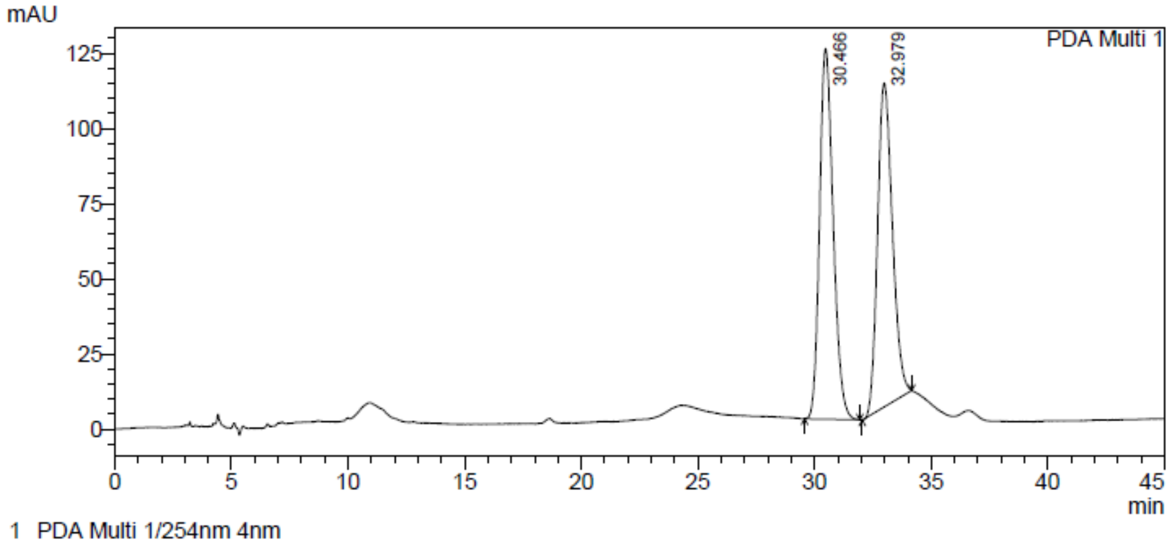

PeakTable

PDA Ch1 254nm 4nm

Peak#	Ret. Time	Area	Height	Area %	Height %
1	30.466	5011562	123330	51.274	53.350
2	32.979	4762486	107842	48.726	46.650
Total		9774048	231172	100.000	100.000

^1H NMR (400 MHz, $CDCl_3$)

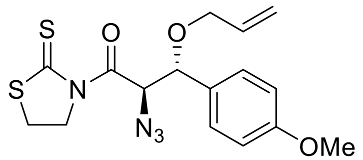

5b

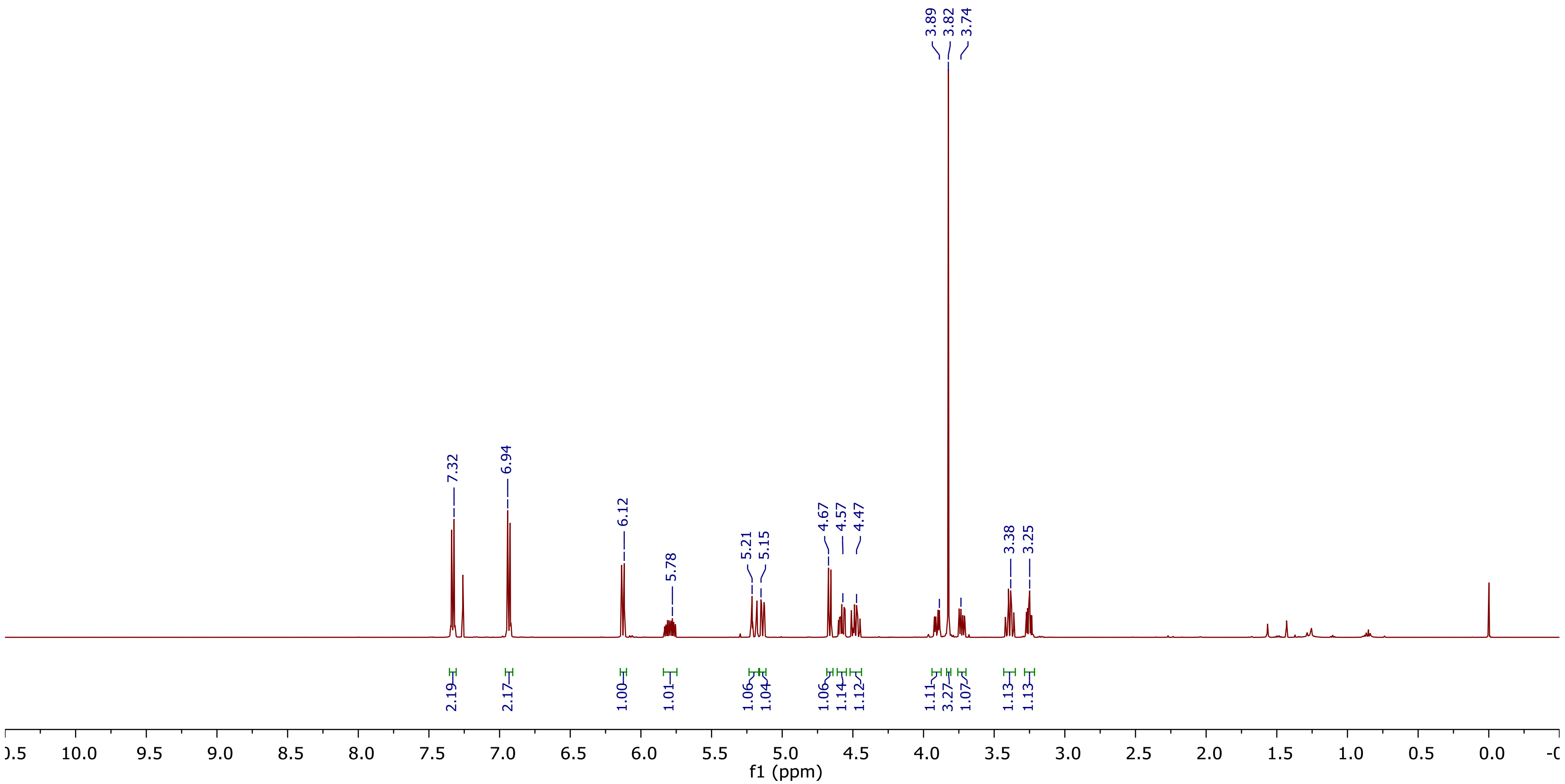

$^{13}C\{^1H\}$ NMR (100.6 MHz, $CDCl_3$)

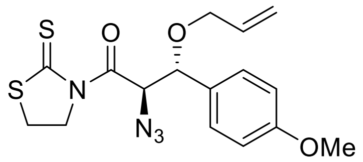

5b

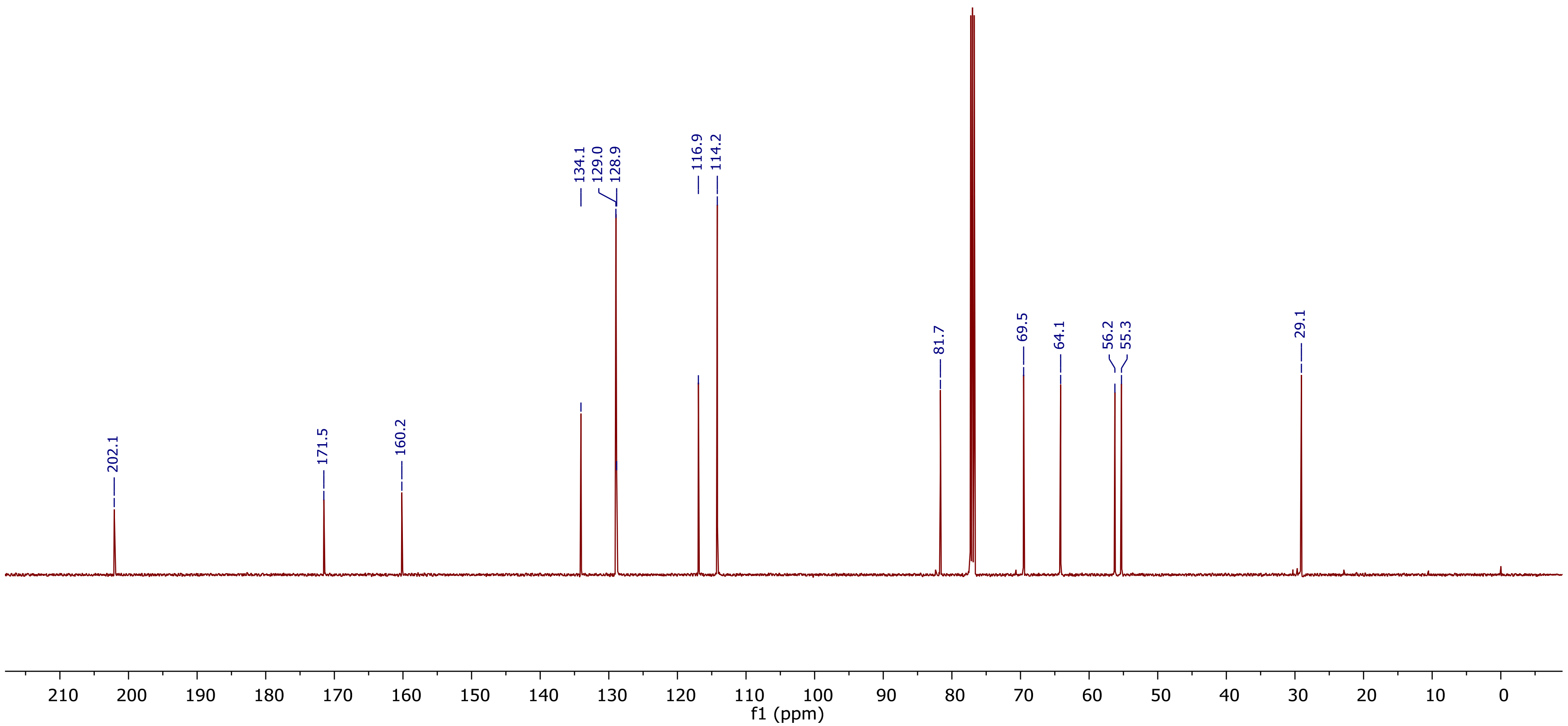

^1H - ^1H COSY NMR (400 MHz, $CDCl_3$)

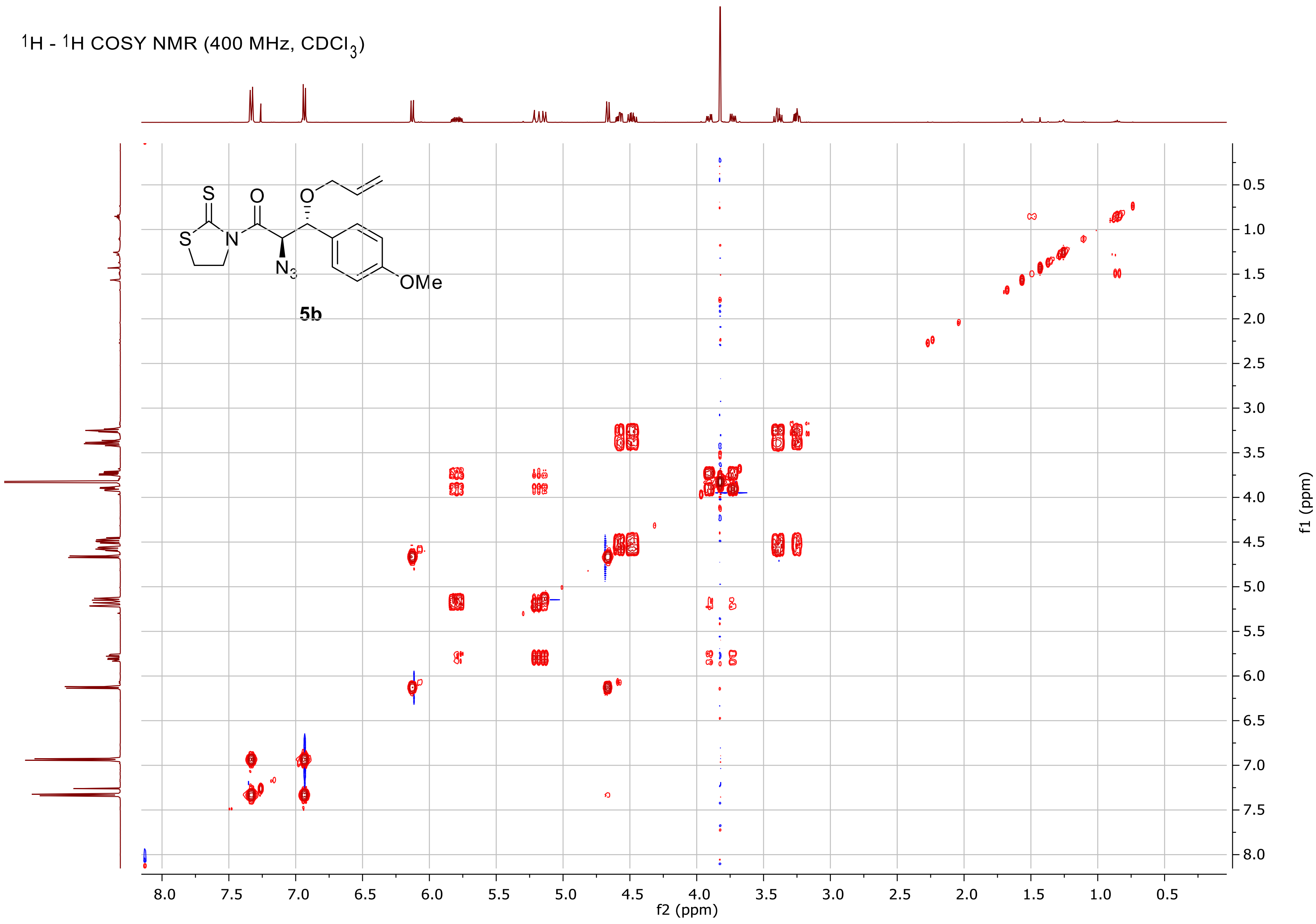

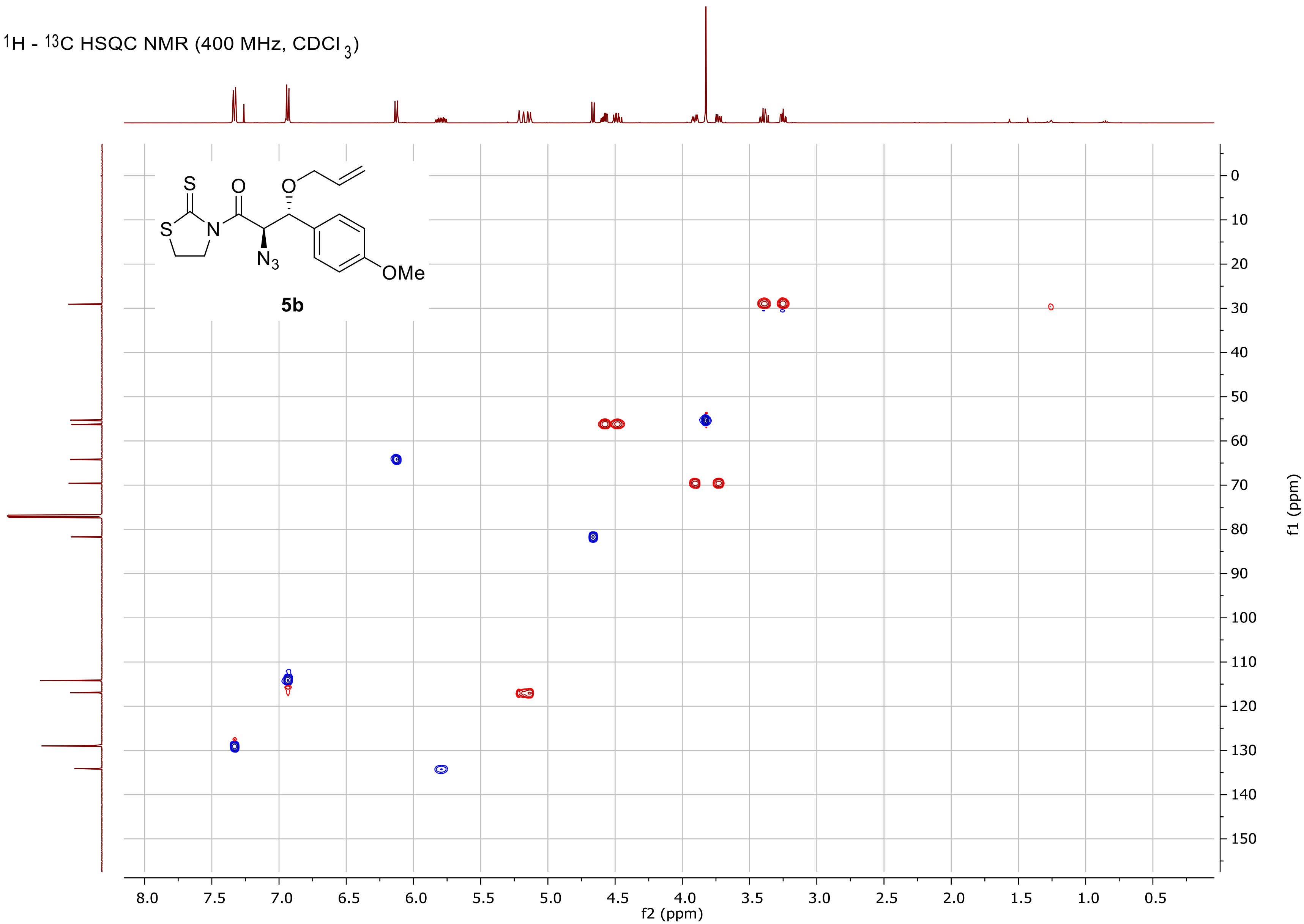

^{1}H - ^{13}C HSQC NMR (400 MHz, $CDCl_3$)
5b
f2 (ppm)
f1 (ppm)

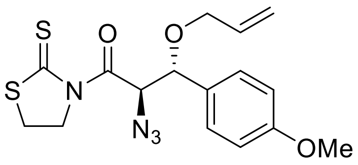

5b

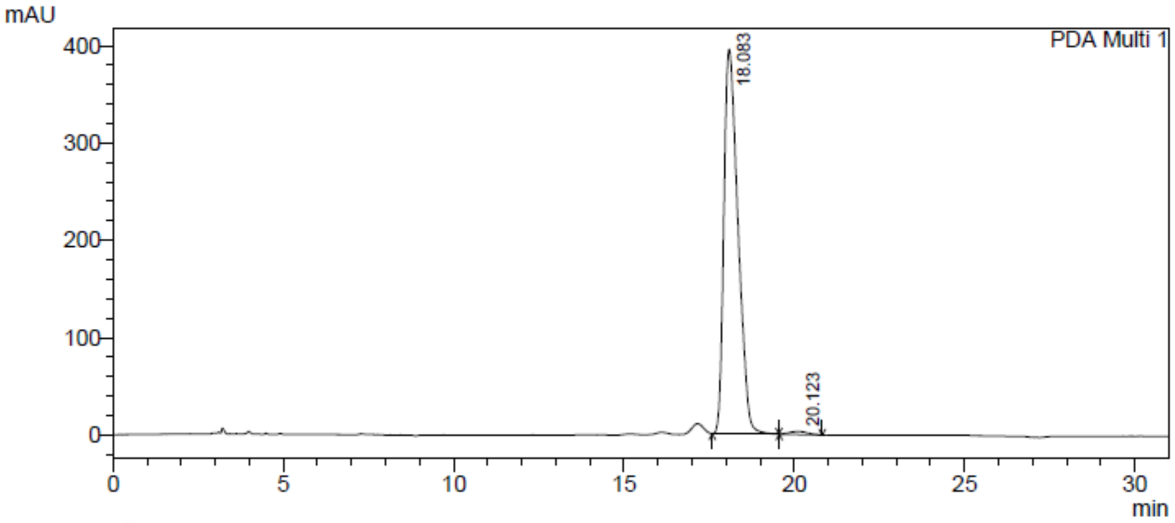

1 PDA Multi 1/254nm 4nm

PeakTable

PDA Ch1 254nm 4nm

Peak#	Ret. Time	Area	Height	Area %	Height %
1	18.083	11385138	395418	98.994	99.194
2	20.123	115648	3213	1.006	0.806
Total		11500786	398631	100.000	100.000

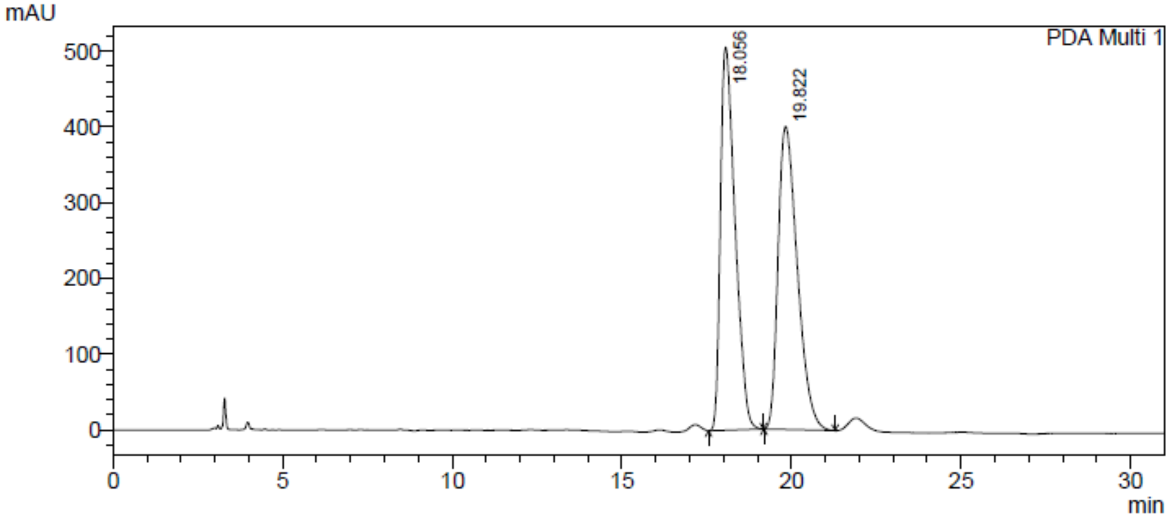

1 PDA Multi 1/254nm 4nm

PeakTable

PDA Ch1 254nm 4nm

Peak#	Ret. Time	Area	Height	Area %	Height %
1	18.056	15230309	505659	49.060	55.821
2	19.822	15813916	400199	50.940	44.179
Total		31044225	905858	100.000	100.000

^{1}H NMR (400 MHz, $CDCl_3$)

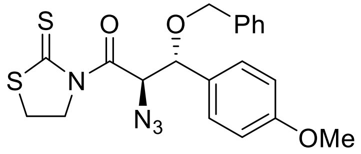

5c

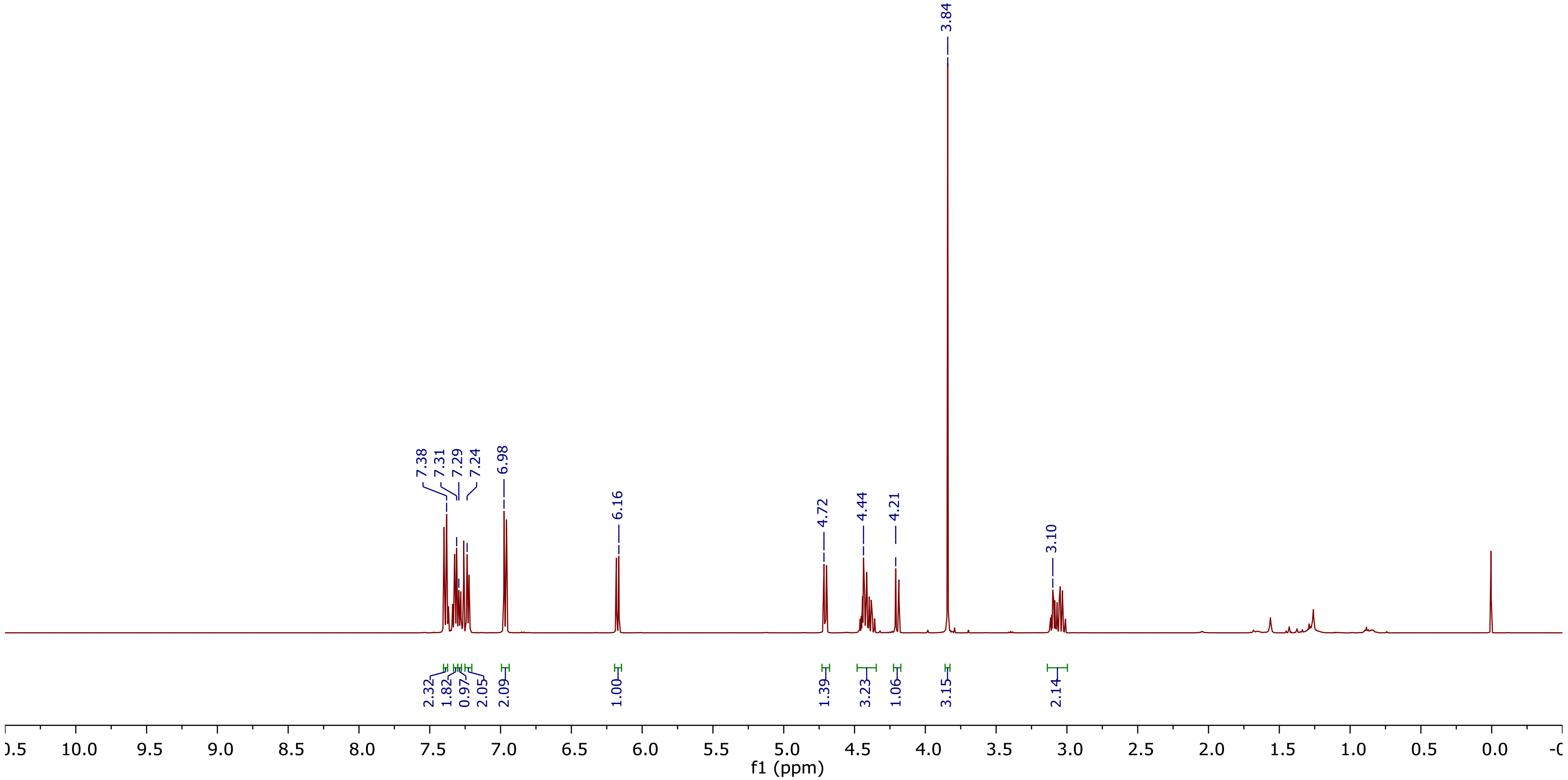

13C{1H} NMR (100.6 MHz, CDCl3)

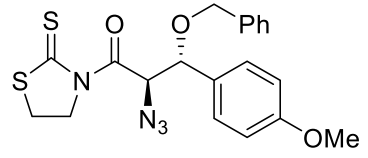

5c

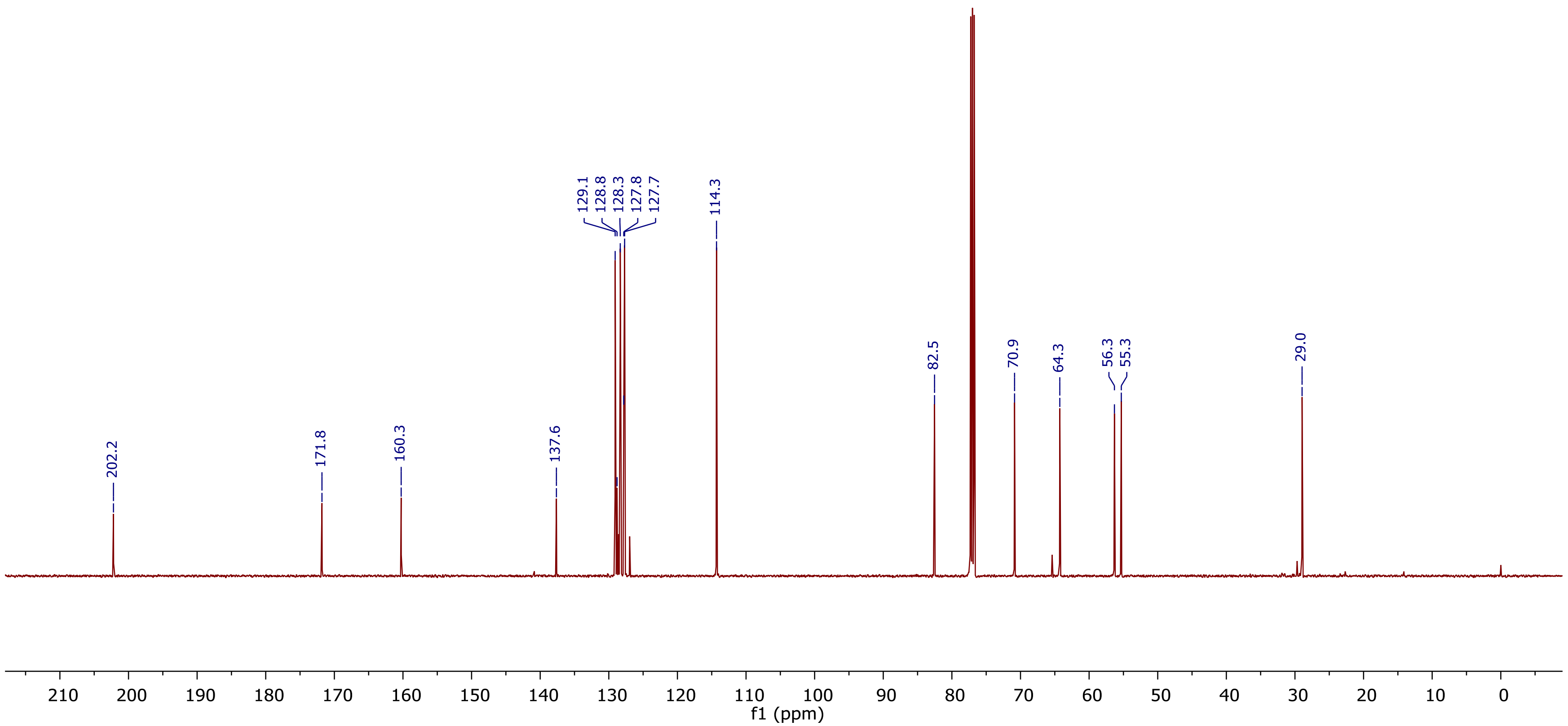

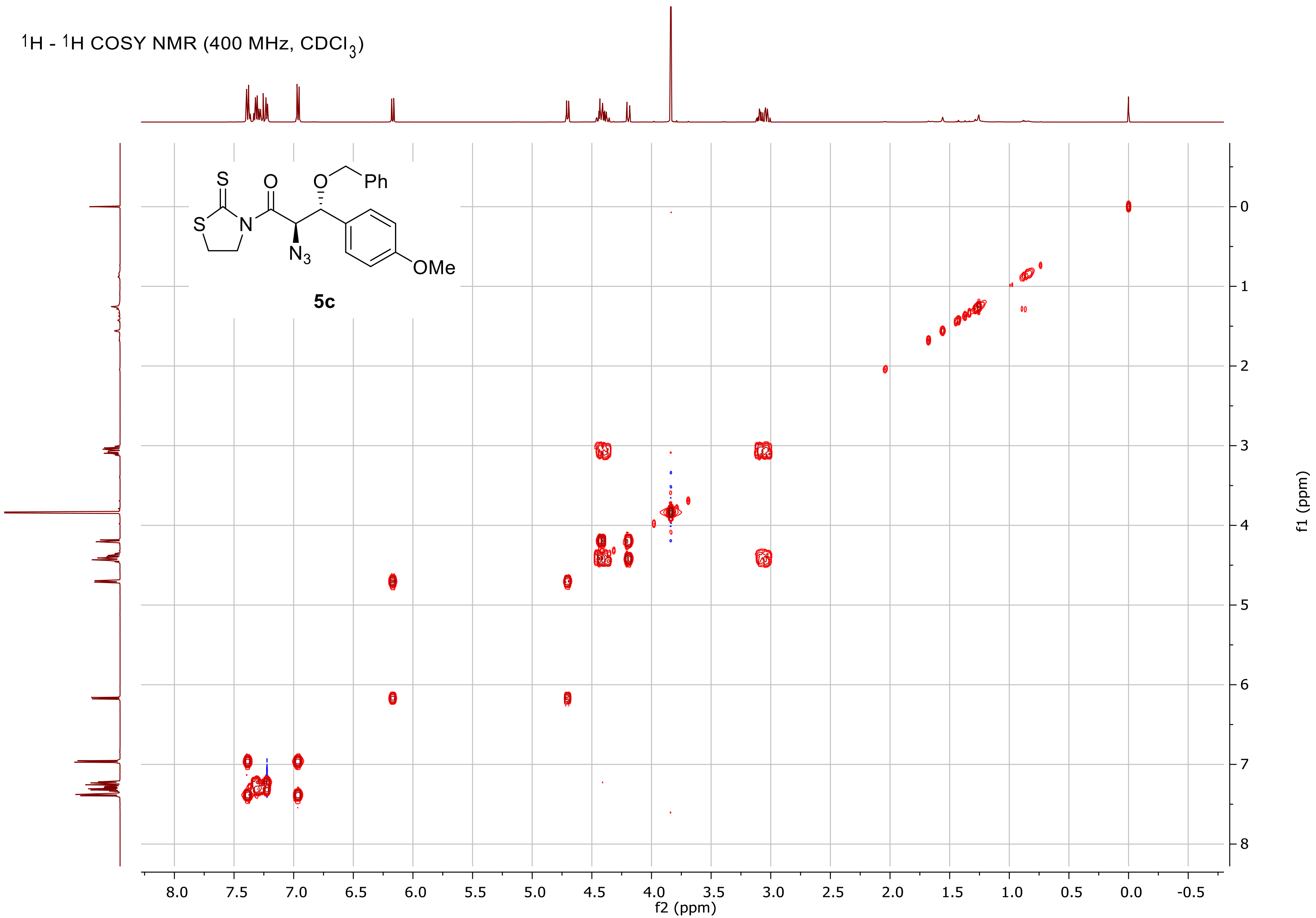
^{1}H - ^{1}H COSY NMR (400 MHz, $CDCl_3$)
S
O
O
Ph
S
N
N_3
OMe
5c
f2 (ppm)
f1 (ppm)
8.0
7.5
7.0
6.5
6.0
5.5
5.0
4.5
4.0
3.5
3.0
2.5
2.0
1.5
1.0
0.5
0.0
-0.5
0
1
2
3
4
5
6
7
8

^1H - ^{13}C HSQC NMR (400 MHz, $CDCl_3$)

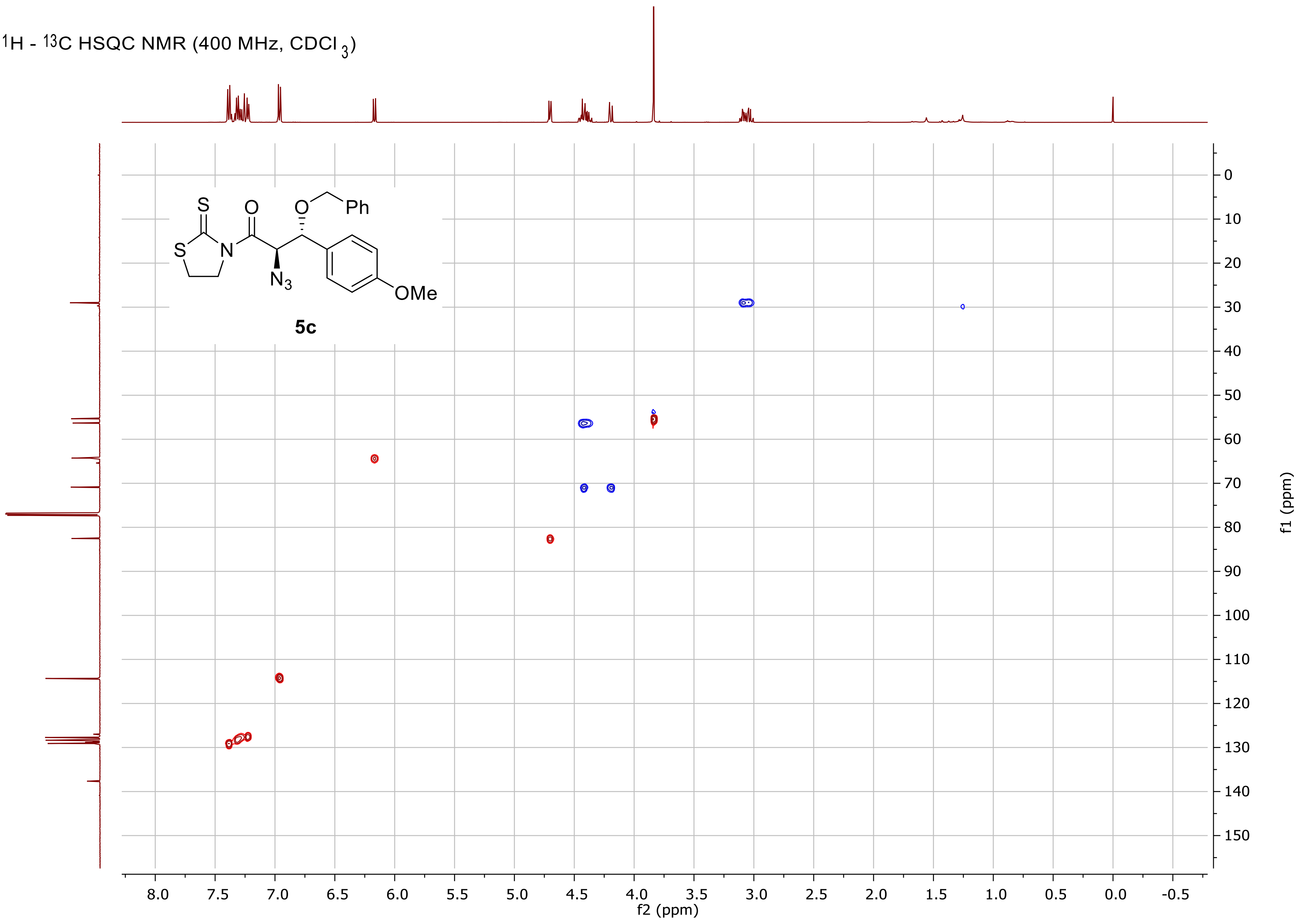

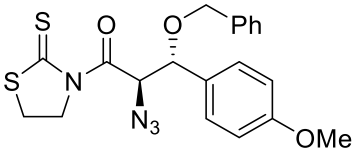

5c

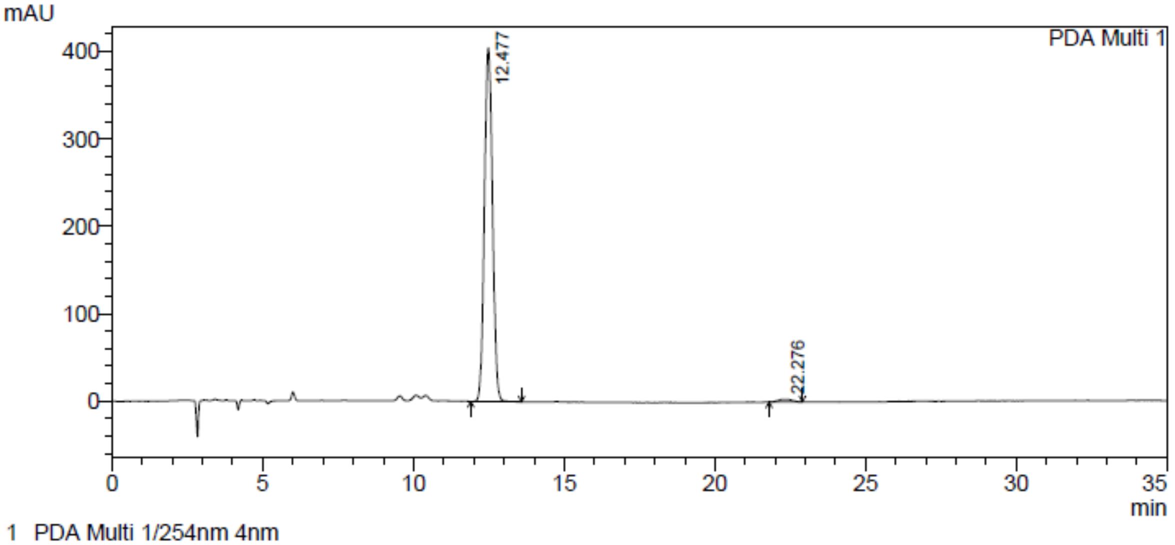

PeakTable

PDA Ch1 254nm 4nm

Peak#	Ret. Time	Area	Height	Area %	Height %
1	12.477	7540094	404554	98.724	99.226
2	22.276	97460	3157	1.276	0.774
Total		7637554	407711	100.000	100.000

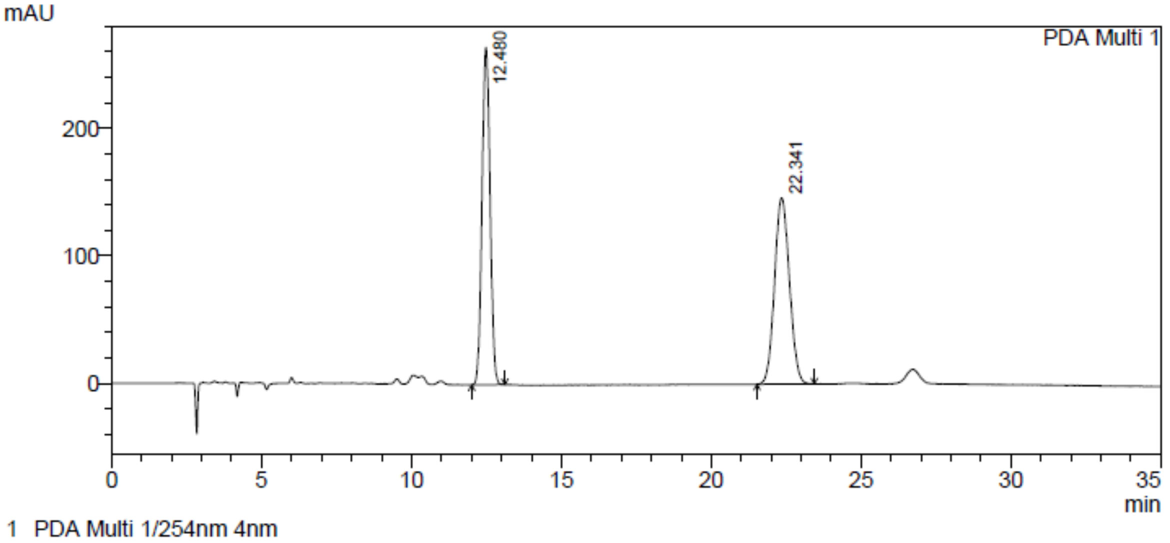

PeakTable

PDA Ch1 254nm 4nm

Peak#	Ret. Time	Area	Height	Area %	Height %
1	12.480	4941863	264677	49.409	64.428
2	22.341	5060128	146135	50.591	35.572
Total		10001990	410811	100.000	100.000

^1H NMR (400 MHz, $CDCl_3$)

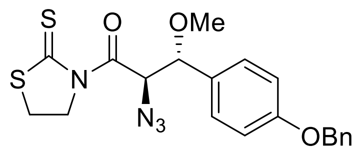

5d

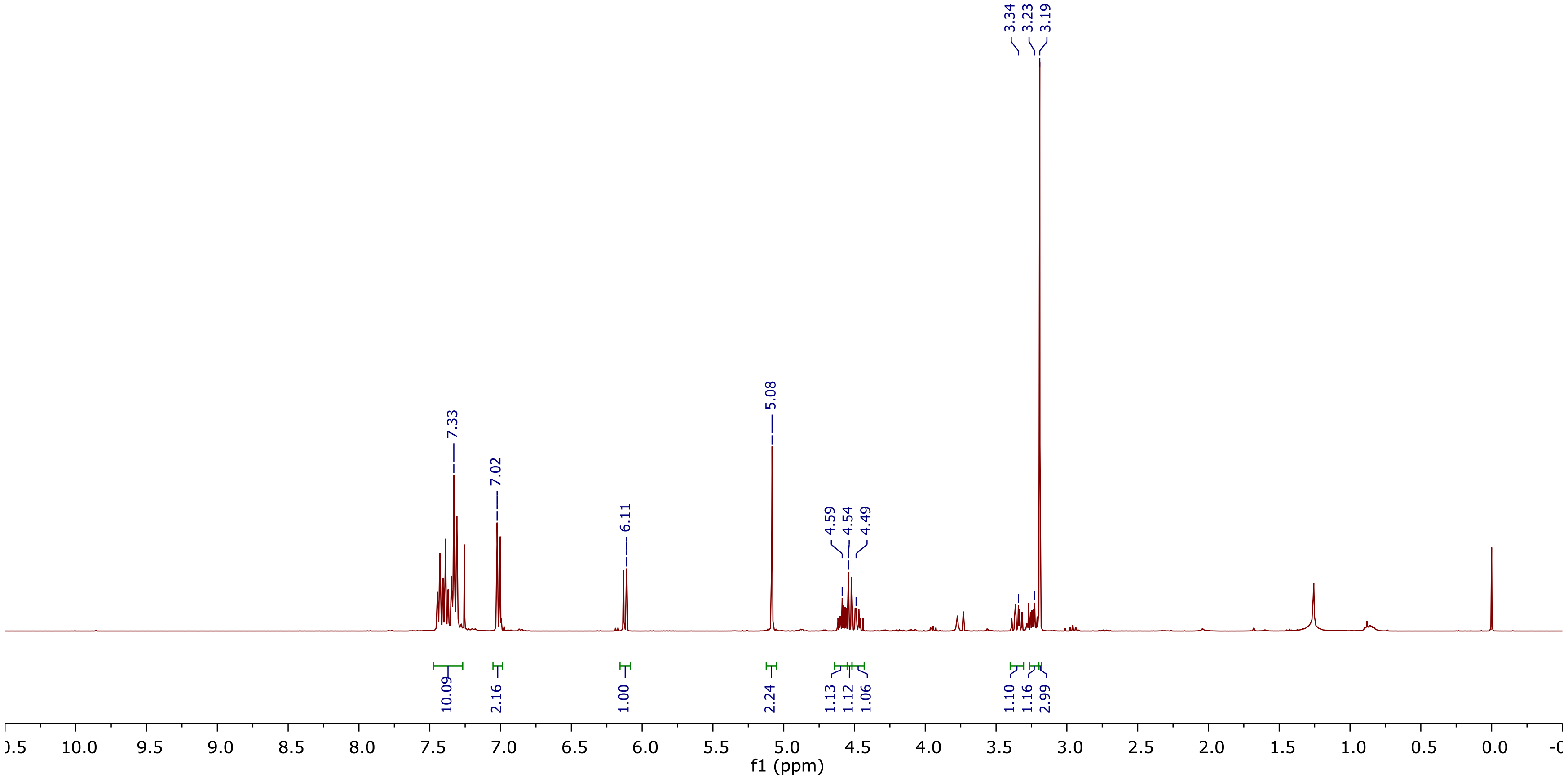

$^{13}C\{^{1}H\}$ NMR (100.6 MHz, $CDCl_3$)

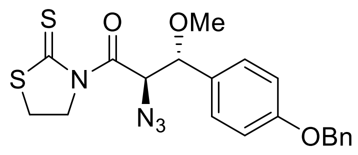

5d

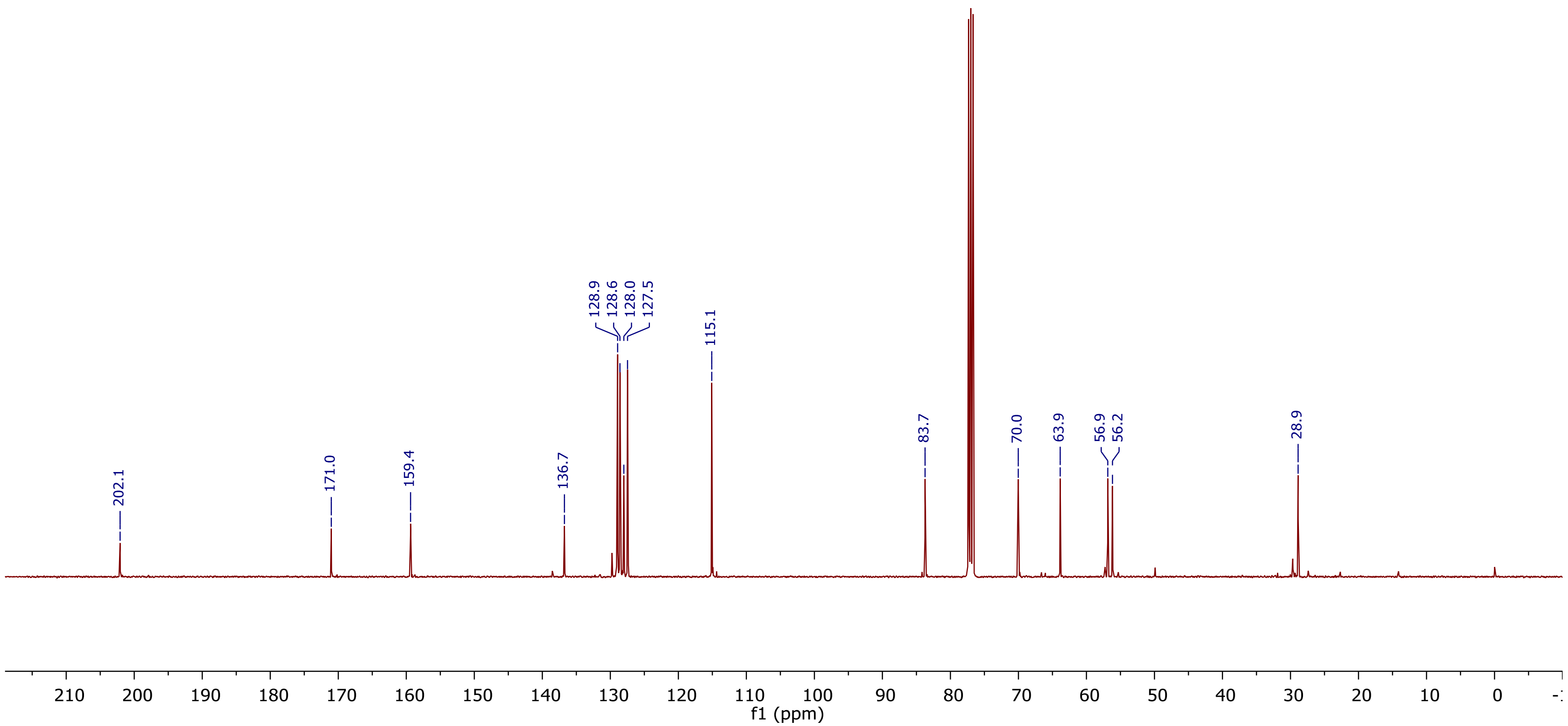

^1H - ^1H COSY NMR (400 MHz, $CDCl_3$)

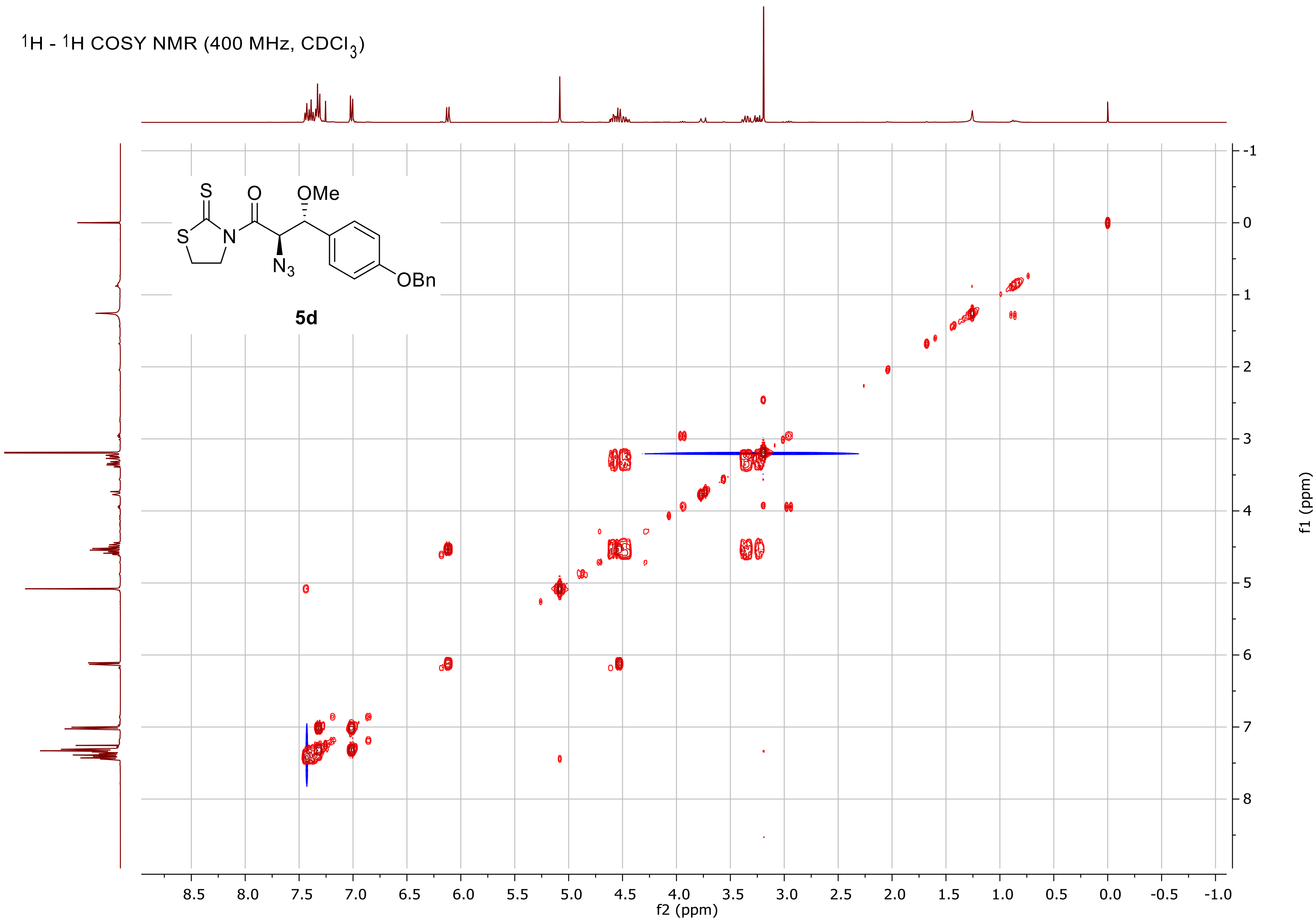

^{1}H - ^{13}C HSQC NMR (400 MHz, $CDCl_3$)

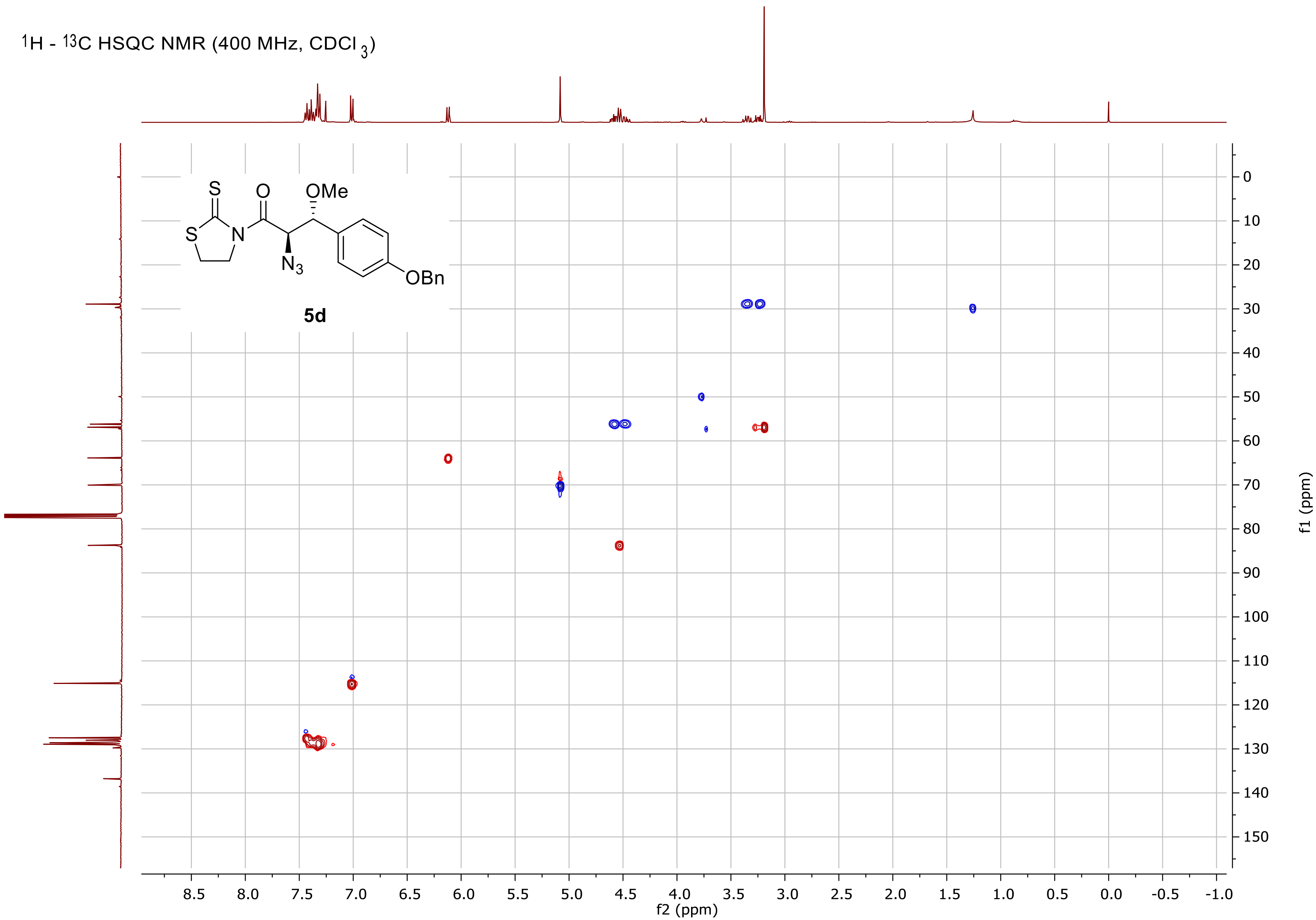

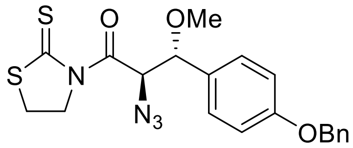

5d

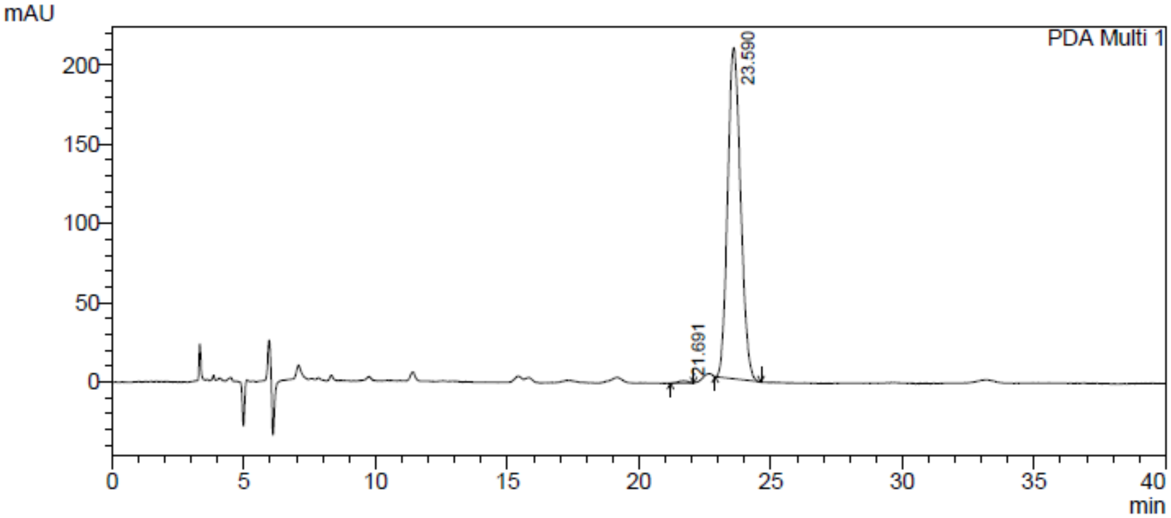

1 PDA Multi 1/254nm 4nm

PeakTable

PDA Ch1 254nm 4nm

Peak#	Ret. Time	Area	Height	Area %	Height %
1	21.691	39333	1655	0.547	0.786
2	23.590	7145486	208847	99.453	99.214
Total		7184819	210502	100.000	100.000

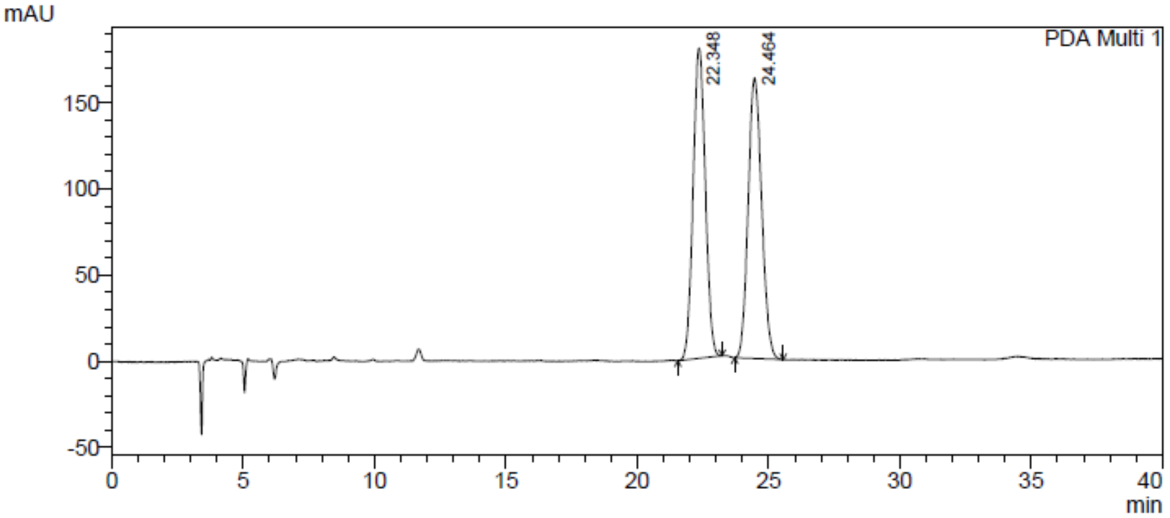

1 PDA Multi 1/254nm 4nm

PeakTable

PDA Ch1 254nm 4nm

Peak#	Ret. Time	Area	Height	Area %	Height %
1	22.348	5798398	179965	49.885	52.495
2	24.464	5825224	162862	50.115	47.505
Total		11623622	342827	100.000	100.000

^1H NMR (400 MHz, $CDCl_3$)

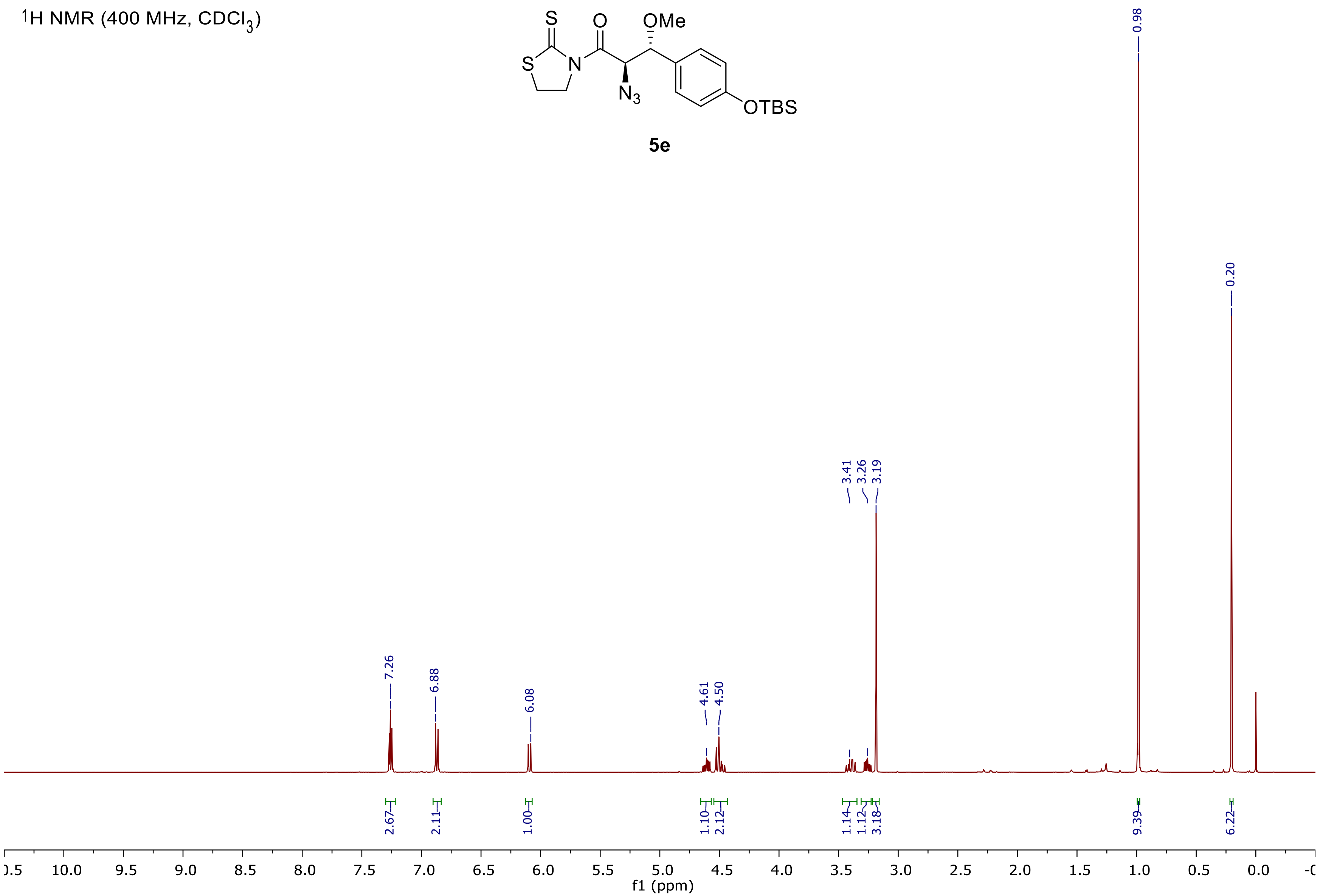

$^{13}C\{^1H\}$ NMR (100.6 MHz, $CDCl_3$)

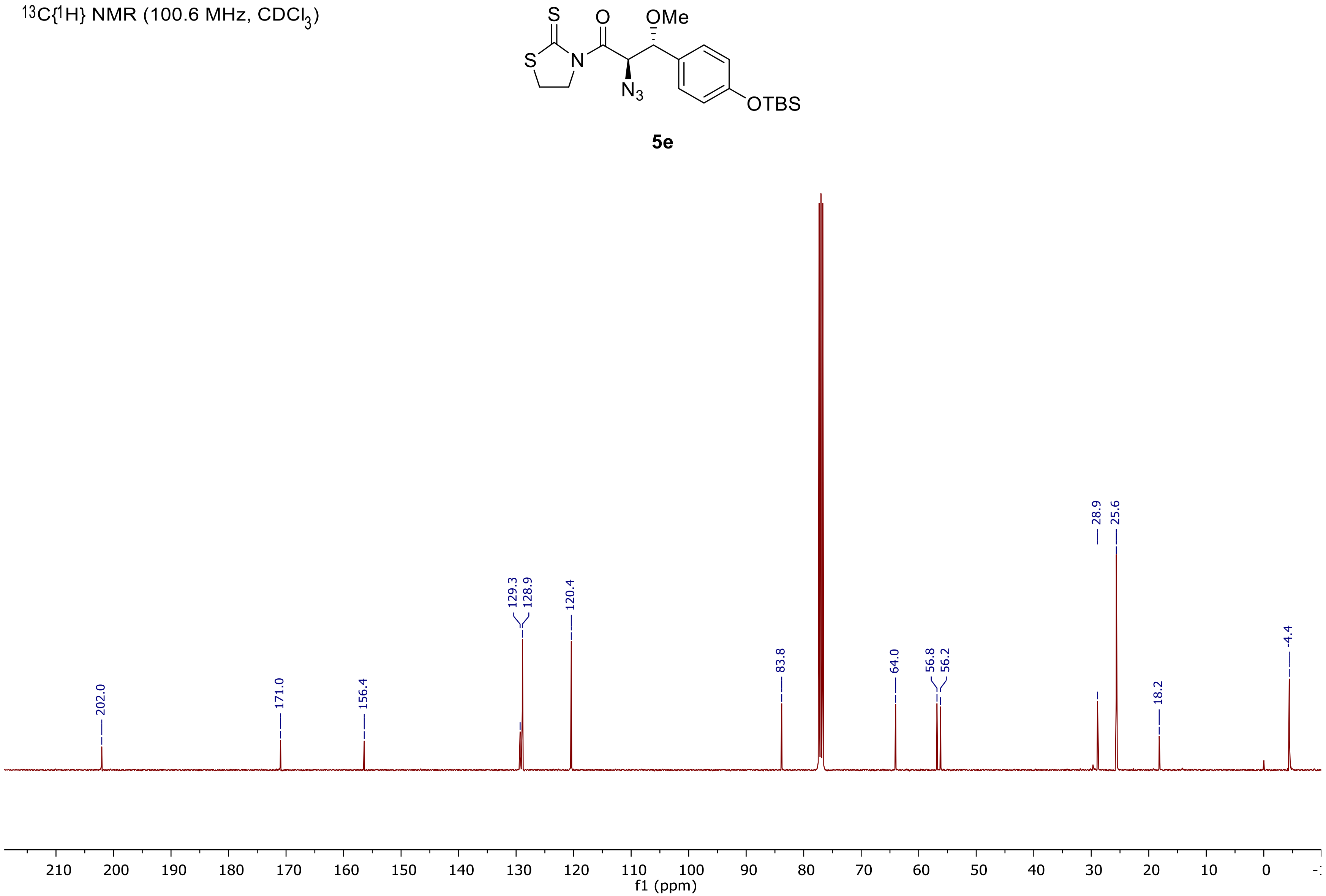

^{1}H - ^{1}H COSY NMR (400 MHz, $CDCl_3$)

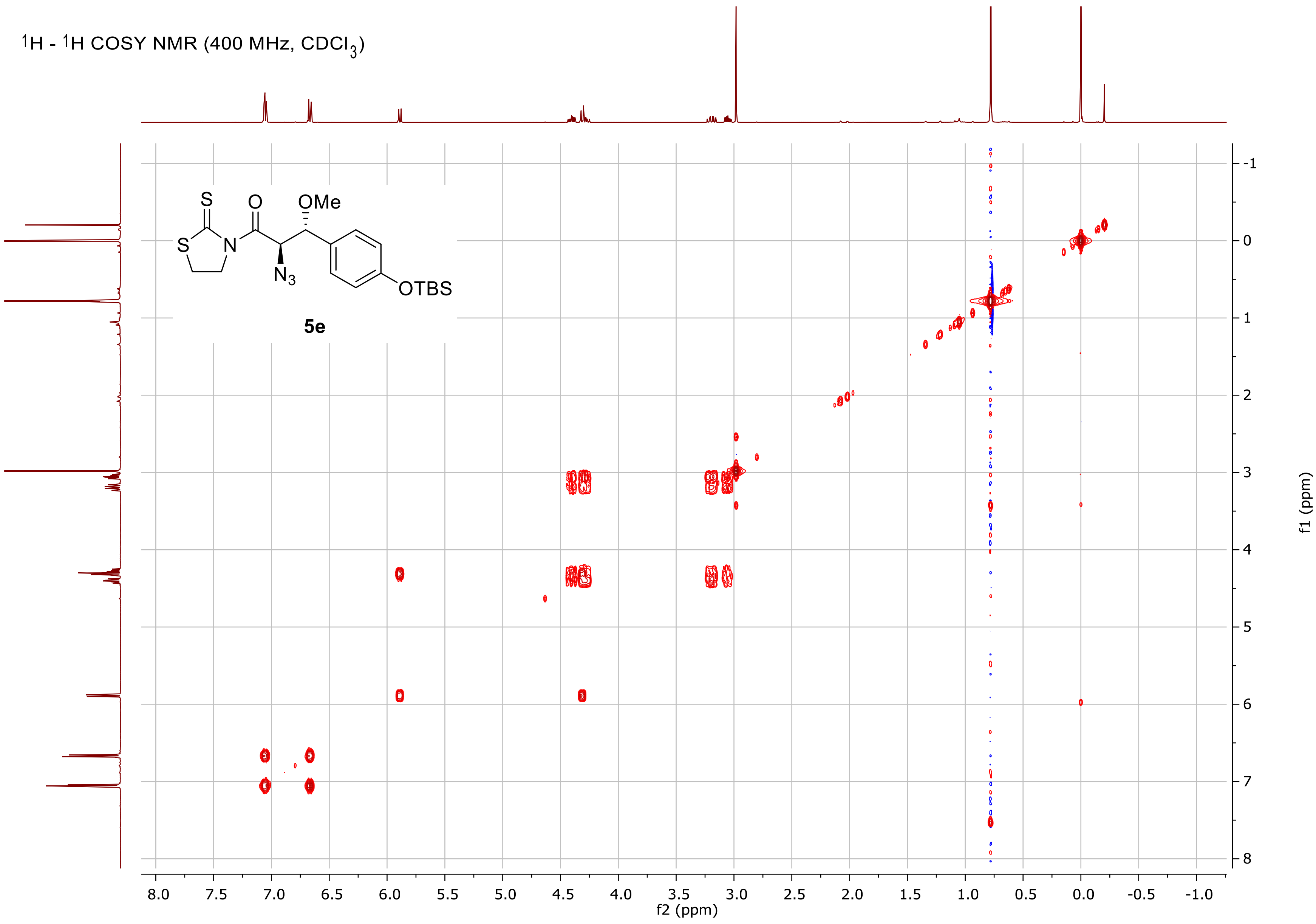

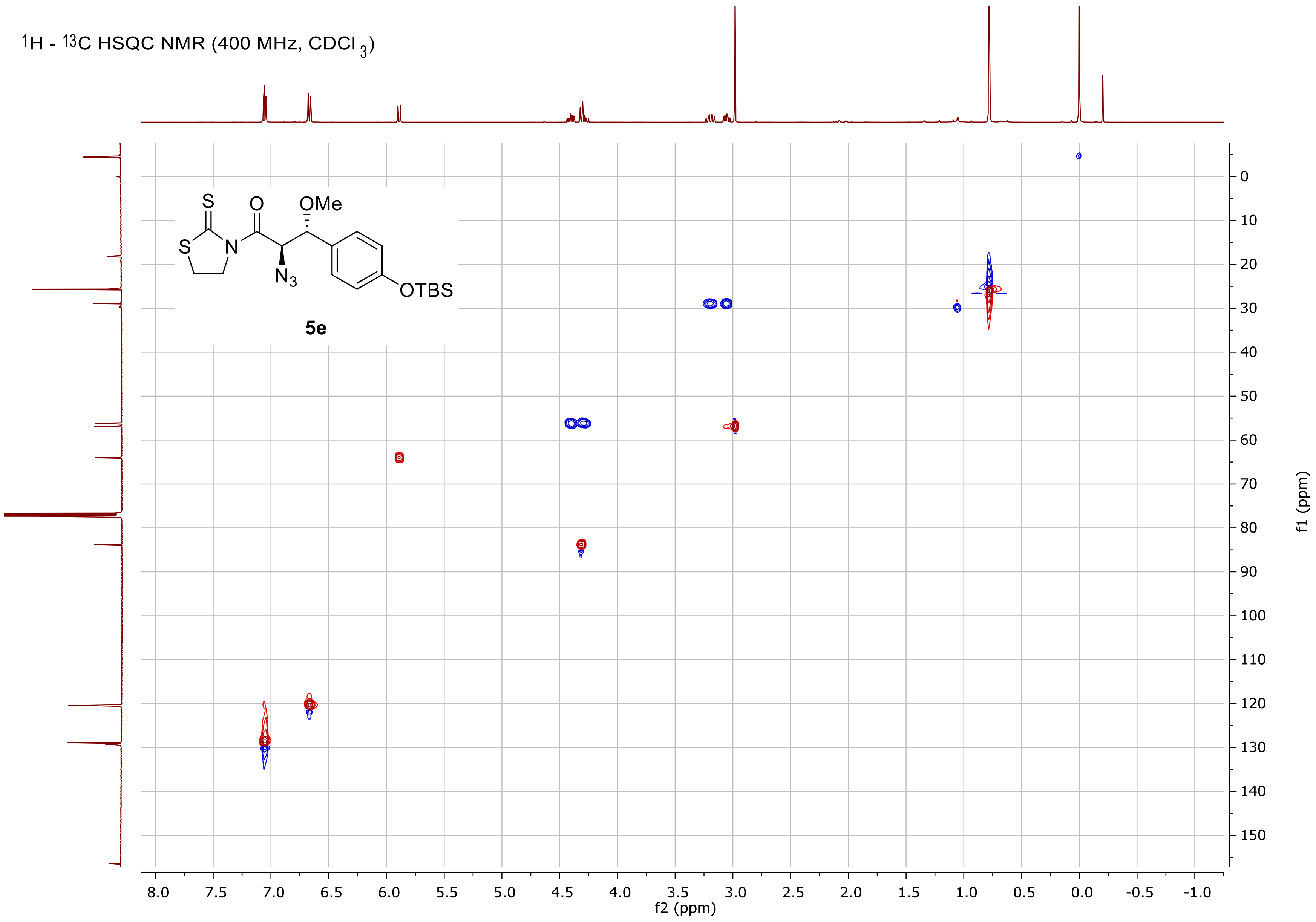

1H - 13C HSQC NMR (400 MHz, CDCl 3)
S
O
OMe
S
N
N3
OTBS
5e
f2 (ppm)
f1 (ppm)

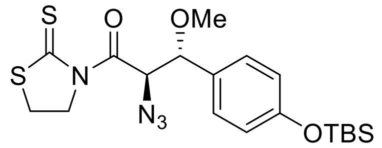

5e

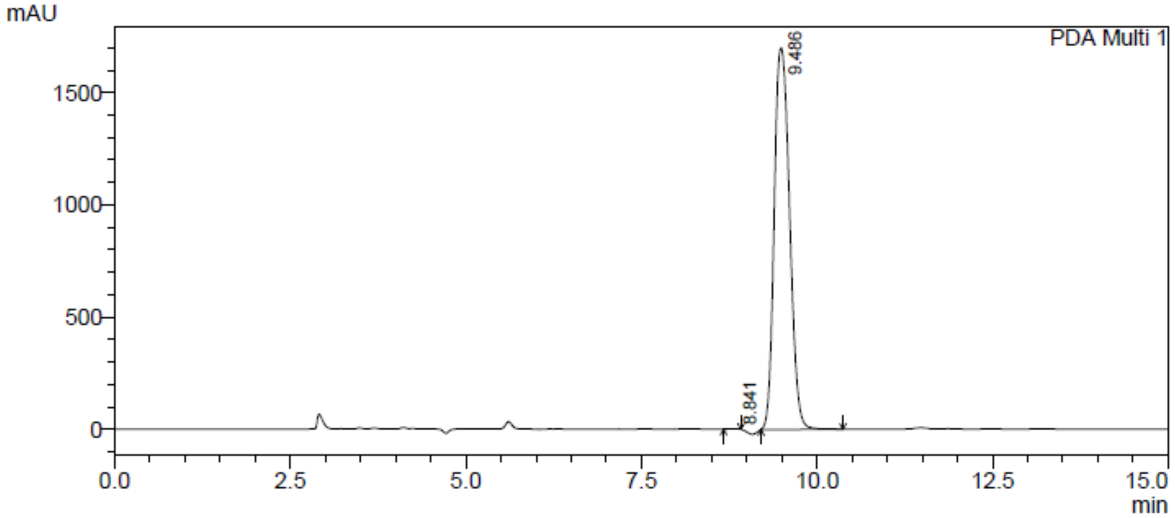

1 PDA Multi 1/254nm 4nm

PeakTable

PDA Ch1 254nm 4nm

Peak#	Ret. Time	Area	Height	Area %	Height %
1	8.841	14547	1885	0.056	0.111
2	9.486	25874375	1701544	99.944	99.889
Total		25888923	1703429	100.000	100.000

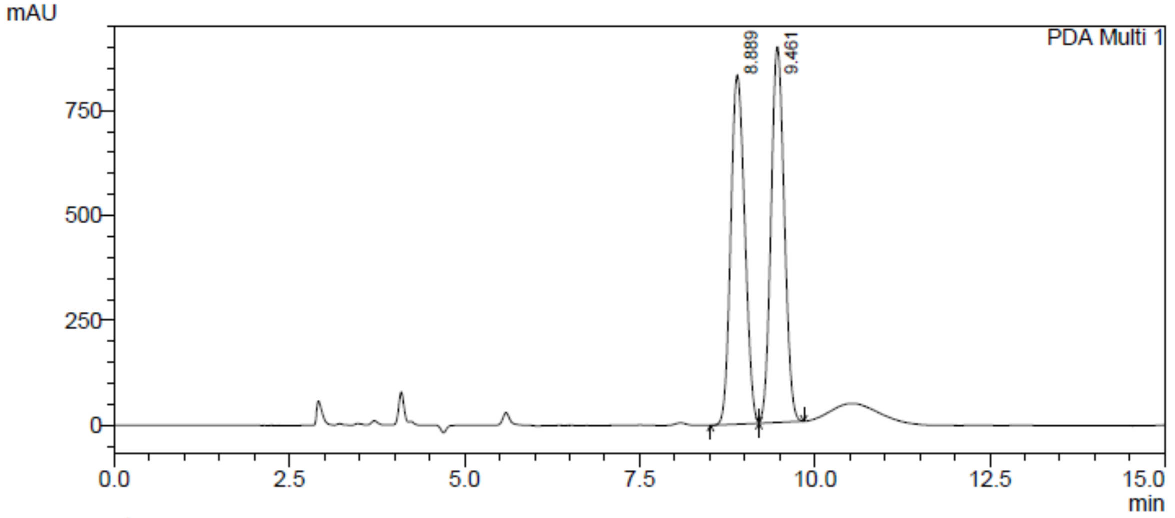

1 PDA Multi 1/254nm 4nm

PeakTable

PDA Ch1 254nm 4nm

Peak#	Ret. Time	Area	Height	Area %	Height %
1	8.889	11589154	832316	49.553	48.201
2	9.461	11798194	894428	50.447	51.799
Total		23387348	1726744	100.000	100.000

^1H NMR (400 MHz, $CDCl_3$)

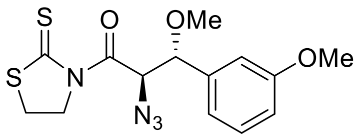

5f

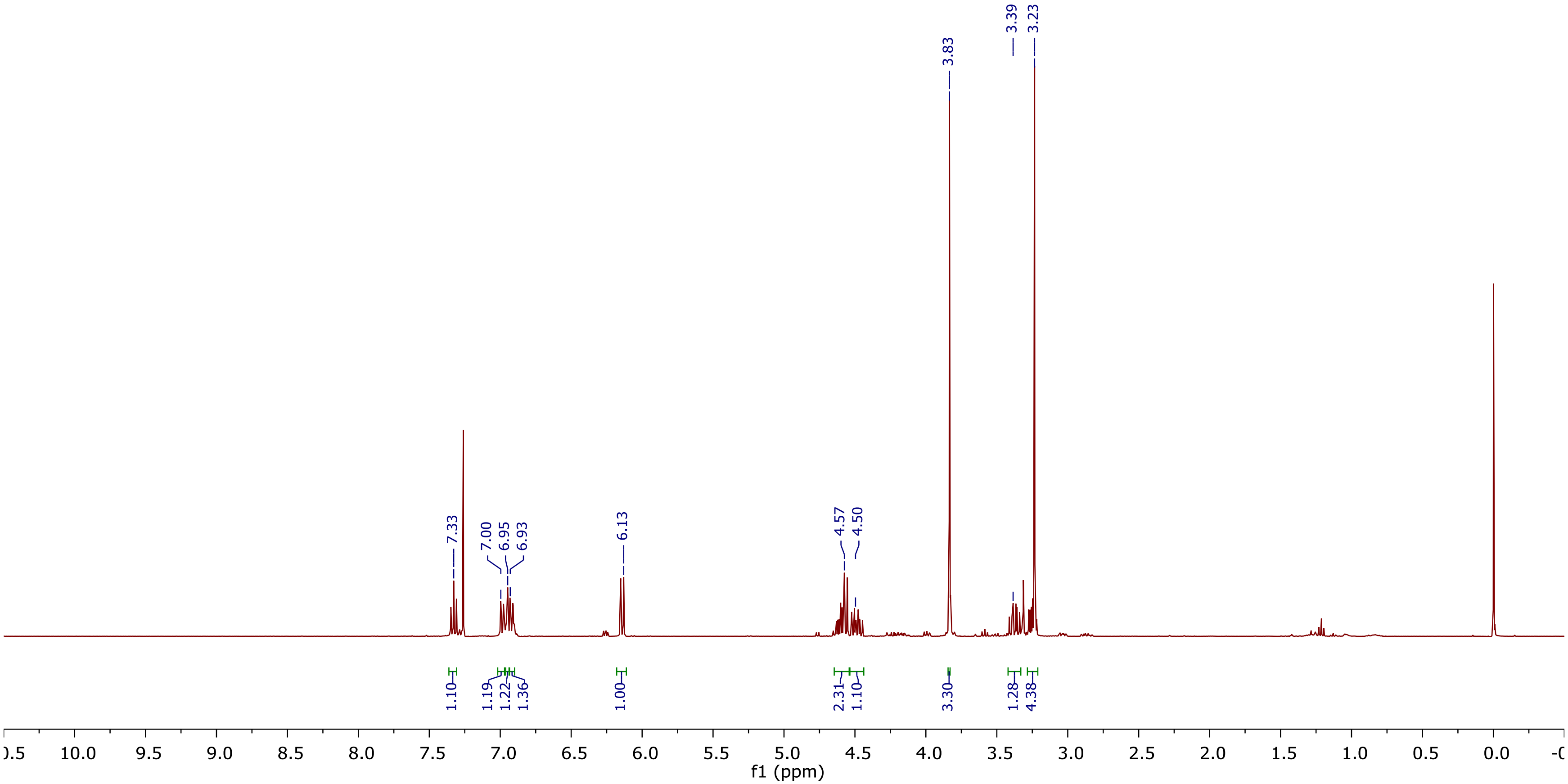

$^{13}C\{^1H\}$ NMR (100.6 MHz, $CDCl_3$)

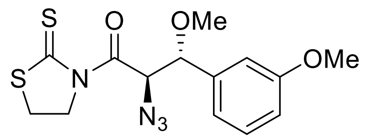

5f

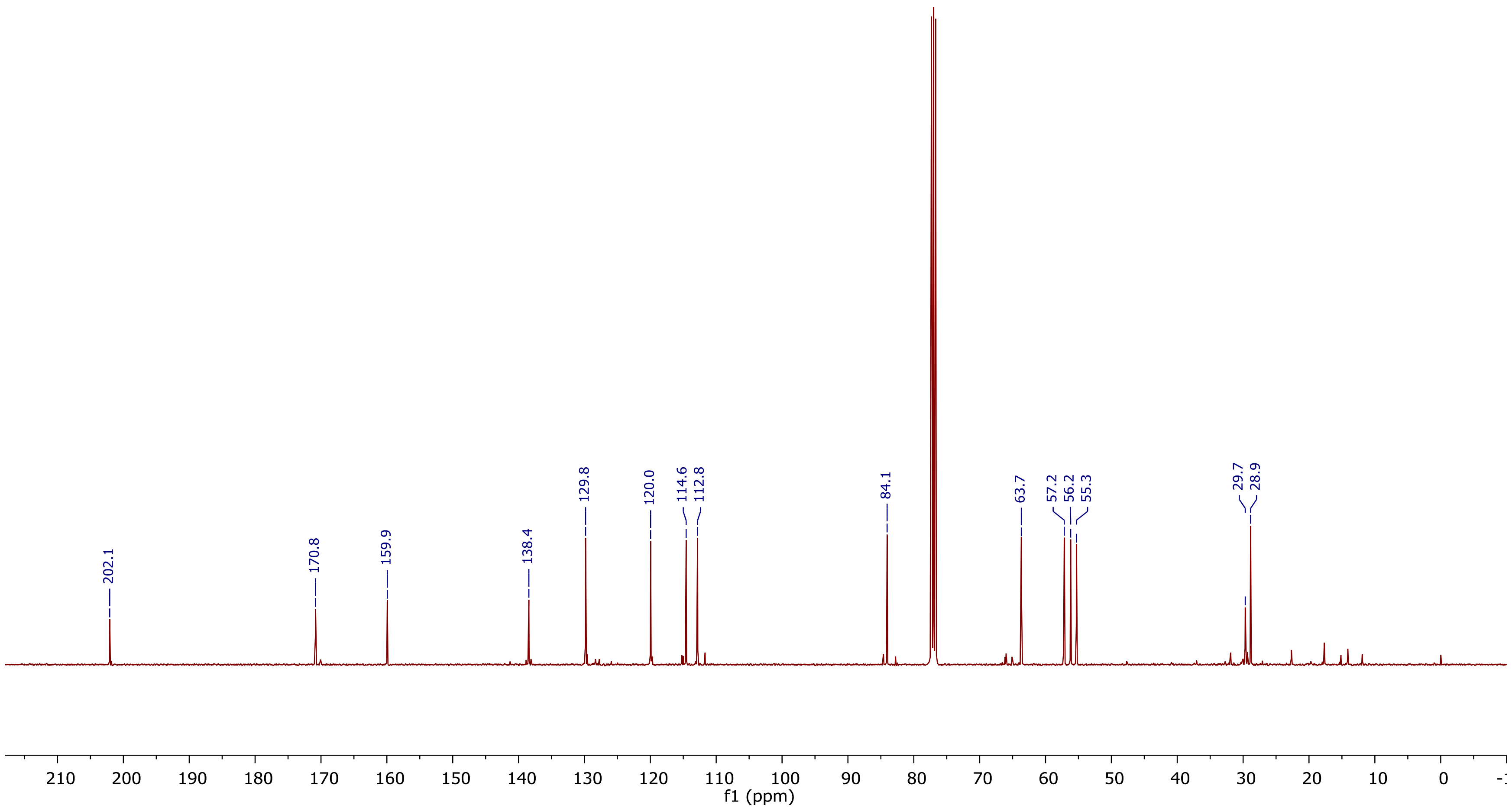

¹H - ¹H COSY NMR (400 MHz, $CDCl_3$)

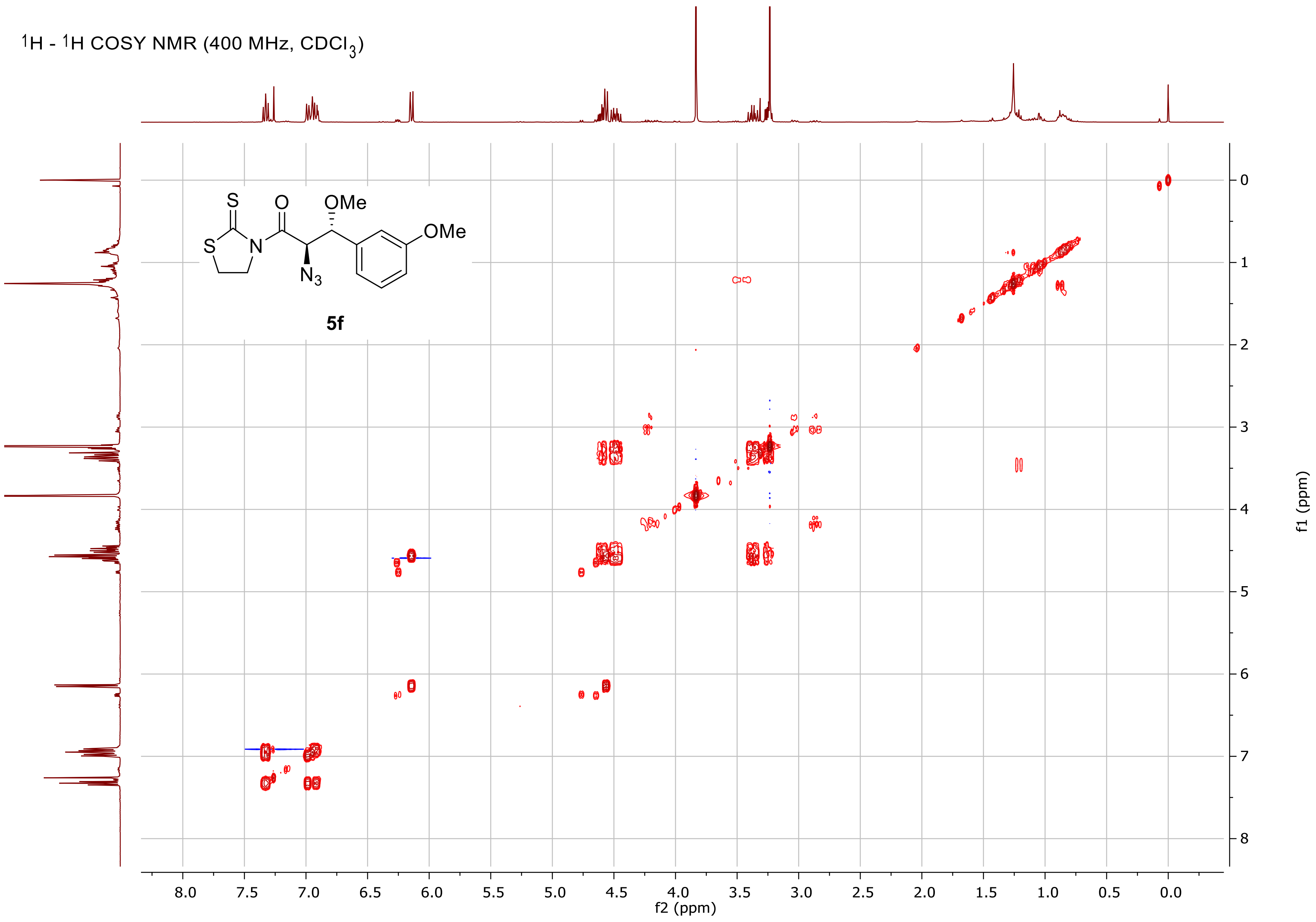

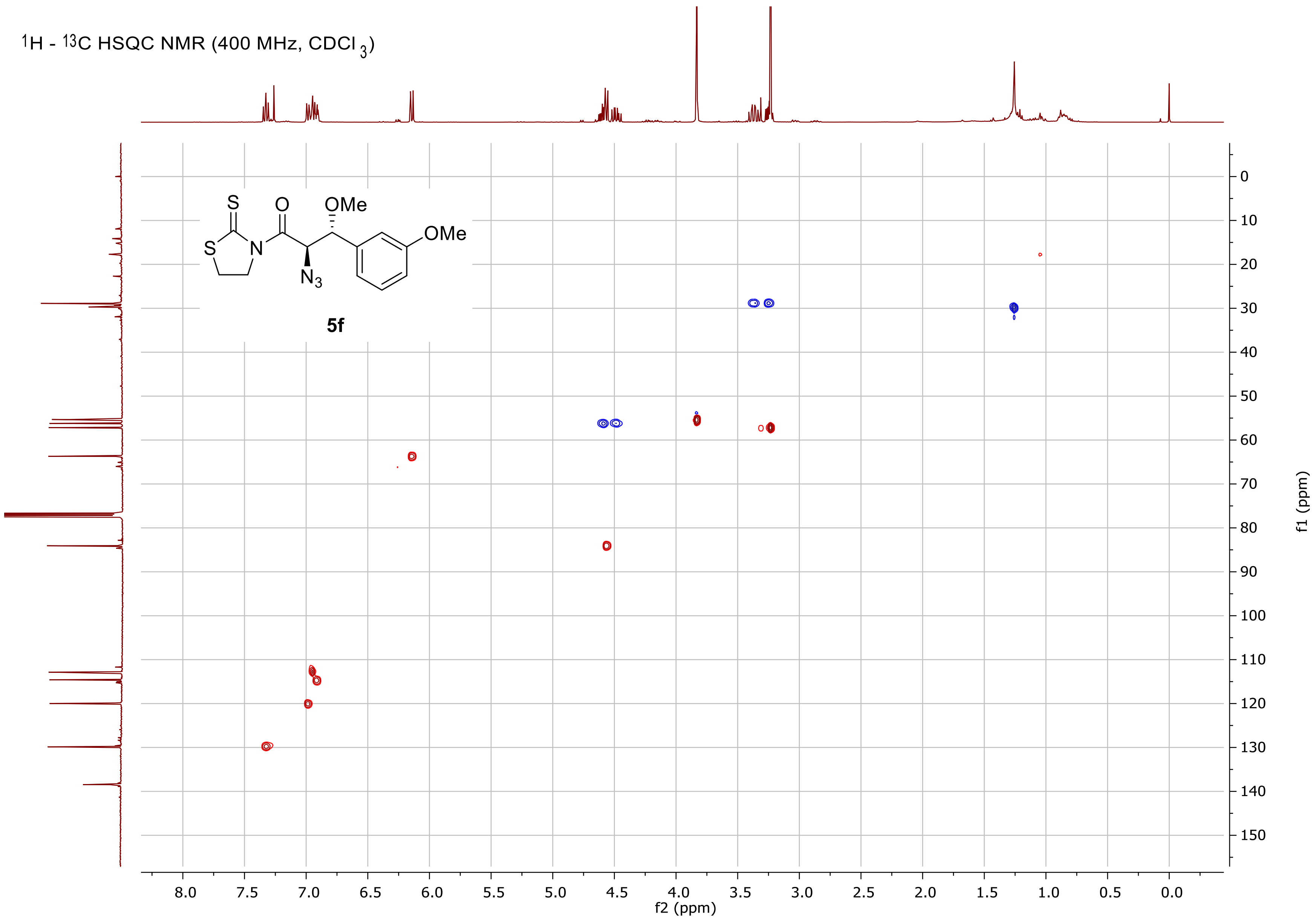

^{1}H - ^{13}C HSQC NMR (400 MHz, $CDCl_3$)
OMe
OMe
O
S
S
N
N_3
5f
f2 (ppm)
f1 (ppm)

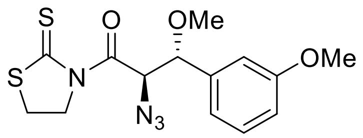

5f

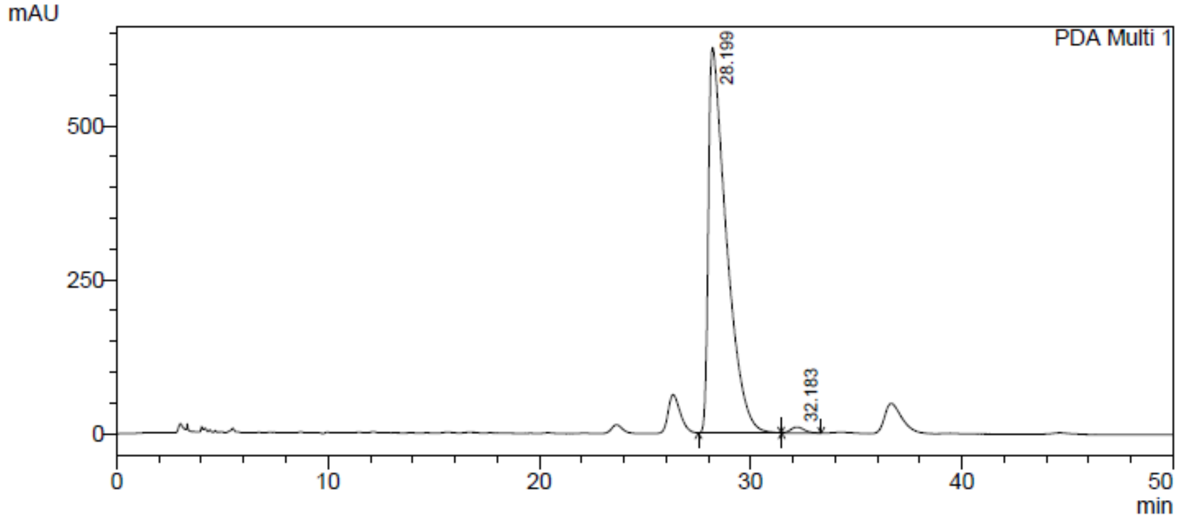

1 PDA Multi 1/254nm 4nm

PeakTable

PDA Ch1 254nm 4nm

Peak#	Ret. Time	Area	Height	Area %	Height %
1	28.199	37208323	626069	98.897	98.486
2	32.183	415015	9625	1.103	1.514
Total		37623338	635694	100.000	100.000

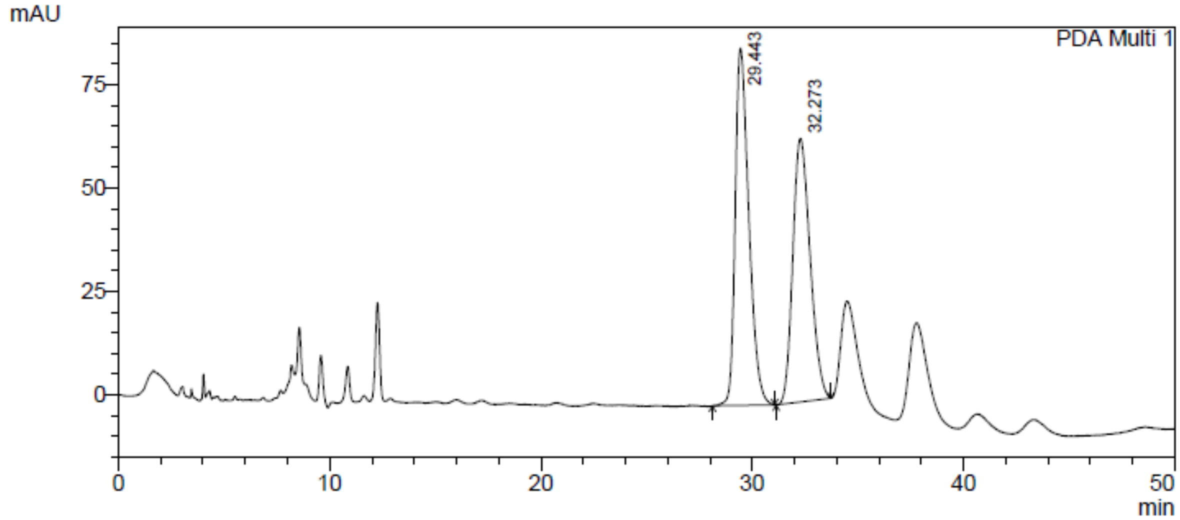

1 PDA Multi 1/254nm 4nm

PeakTable

PDA Ch1 254nm 4nm

Peak#	Ret. Time	Area	Height	Area %	Height %
1	29.443	3869884	86387	51.635	57.528
2	32.273	3624832	63778	48.365	42.472
Total		7494717	150164	100.000	100.000

^{1}H NMR (400 MHz, $CDCl_3$)

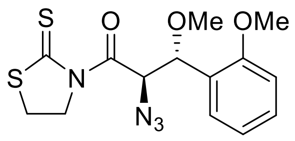

5g

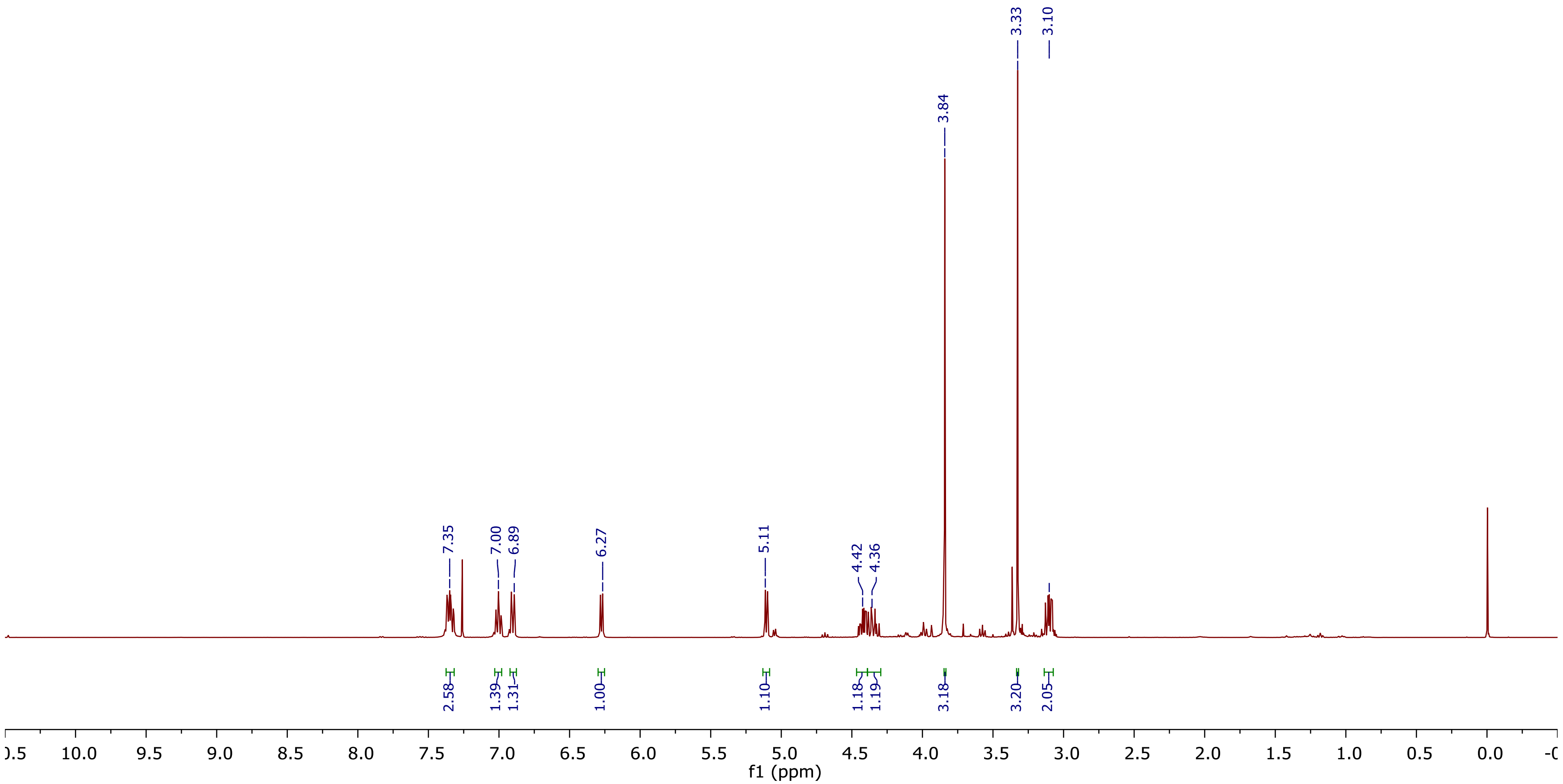

$^{13}C\{^{1}H\}$ NMR (100.6 MHz, $CDCl_3$)

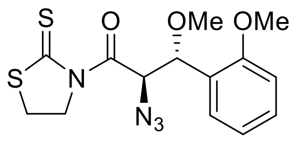

5g

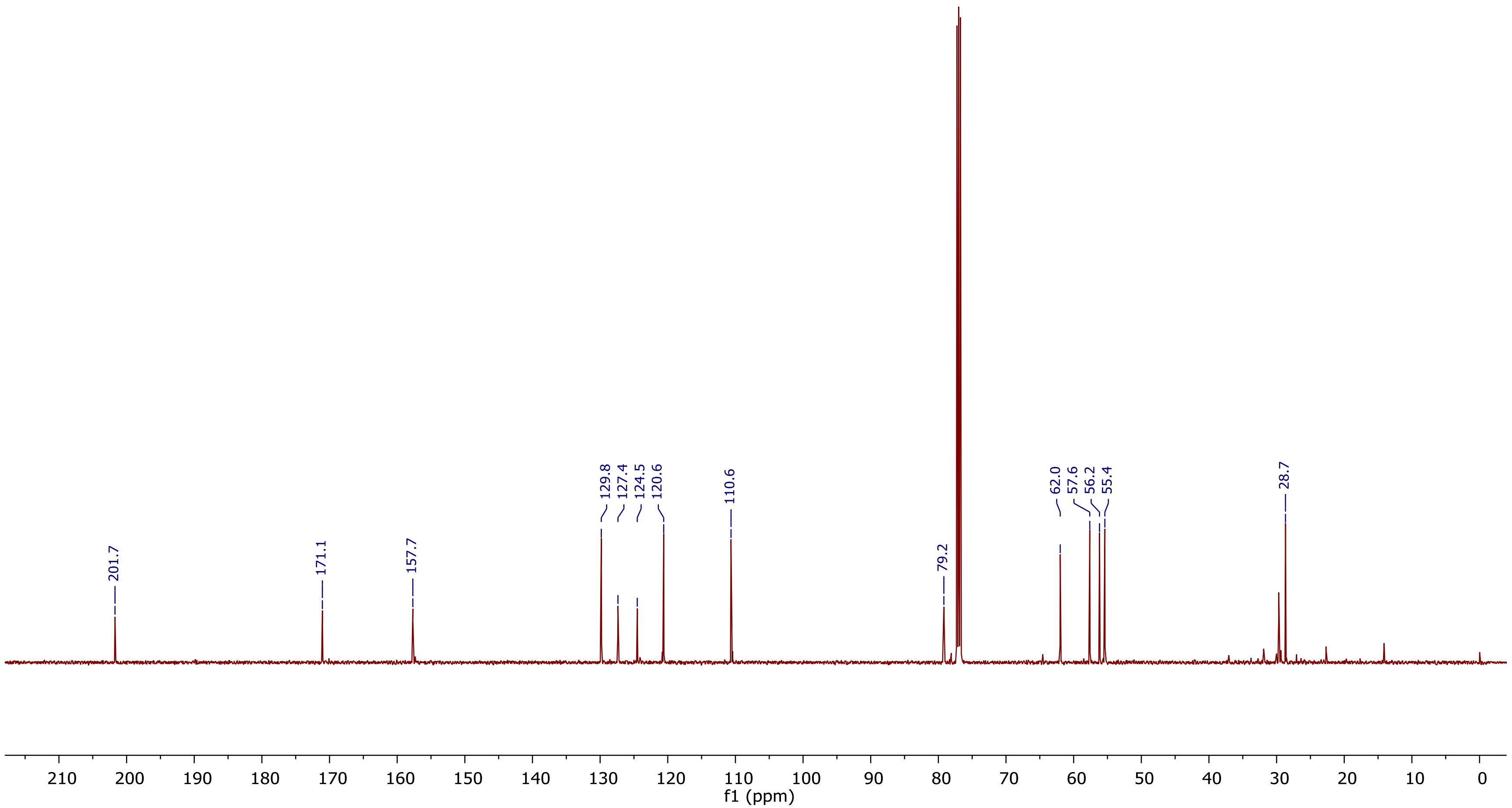

^1H - ^1H COSY NMR (400 MHz, $CDCl_3$)

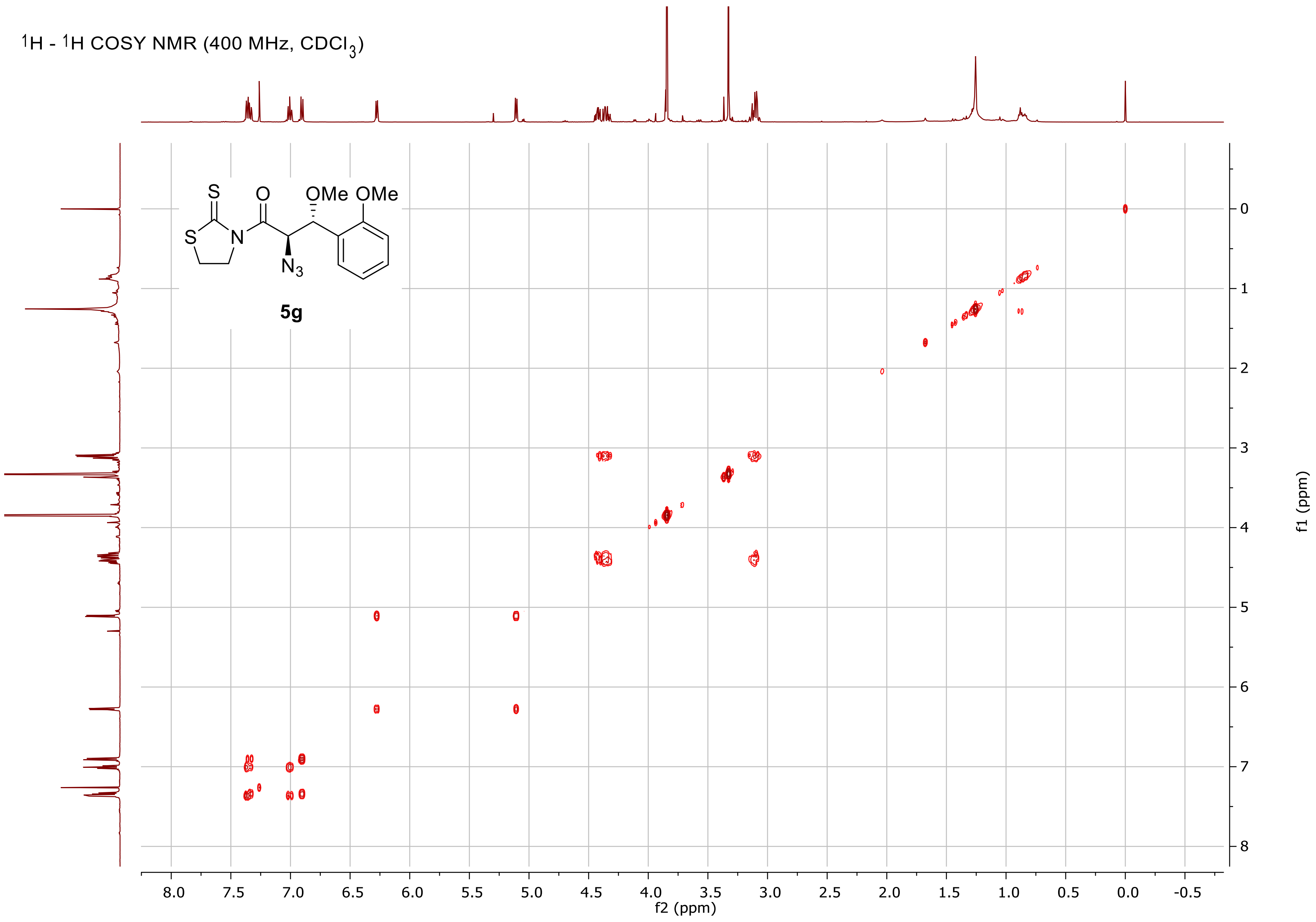

5g

^{1}H - ^{13}C HSQC NMR (400 MHz, $CDCl_3$)

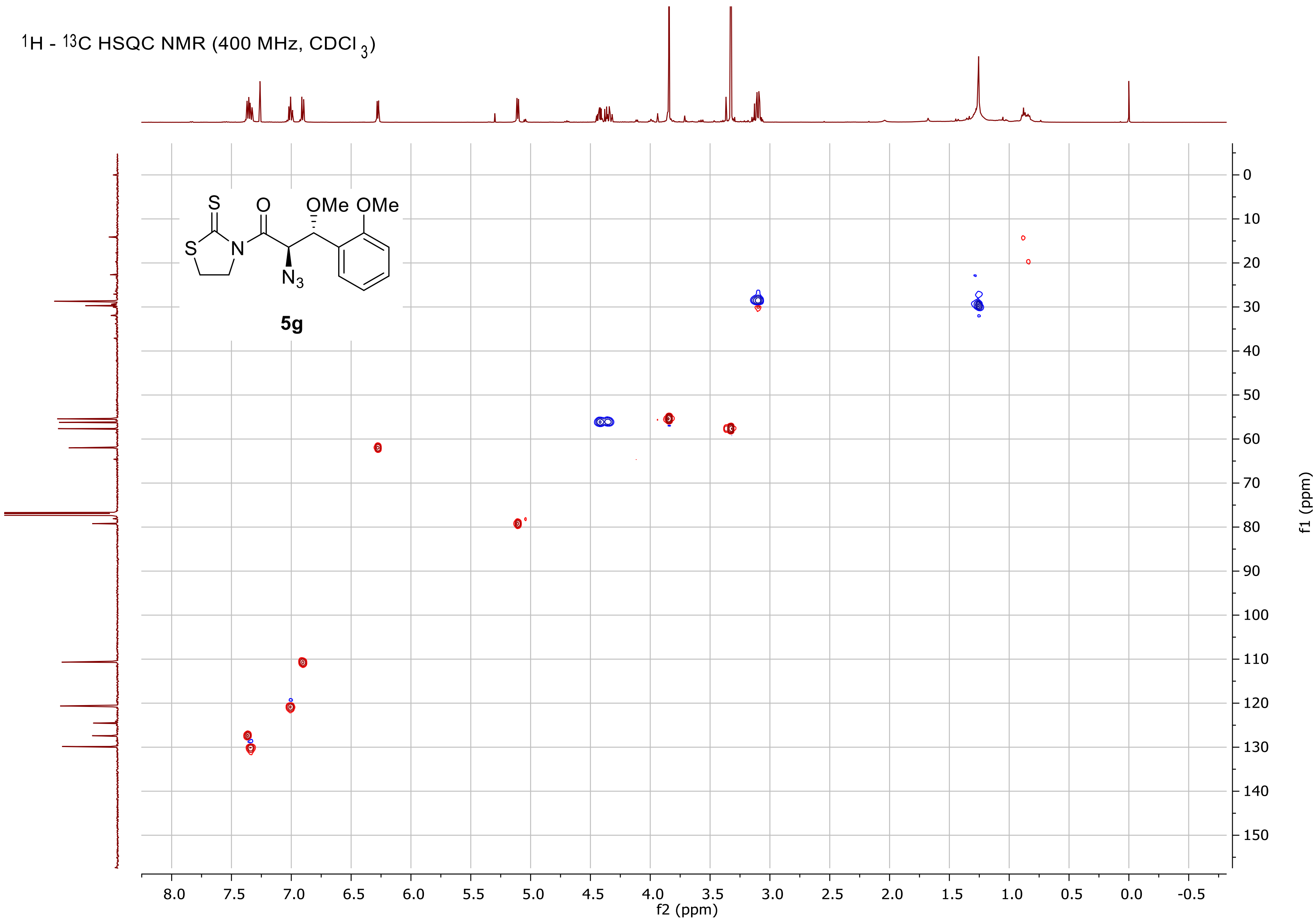

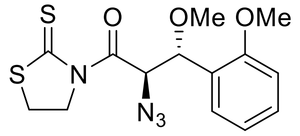

5g

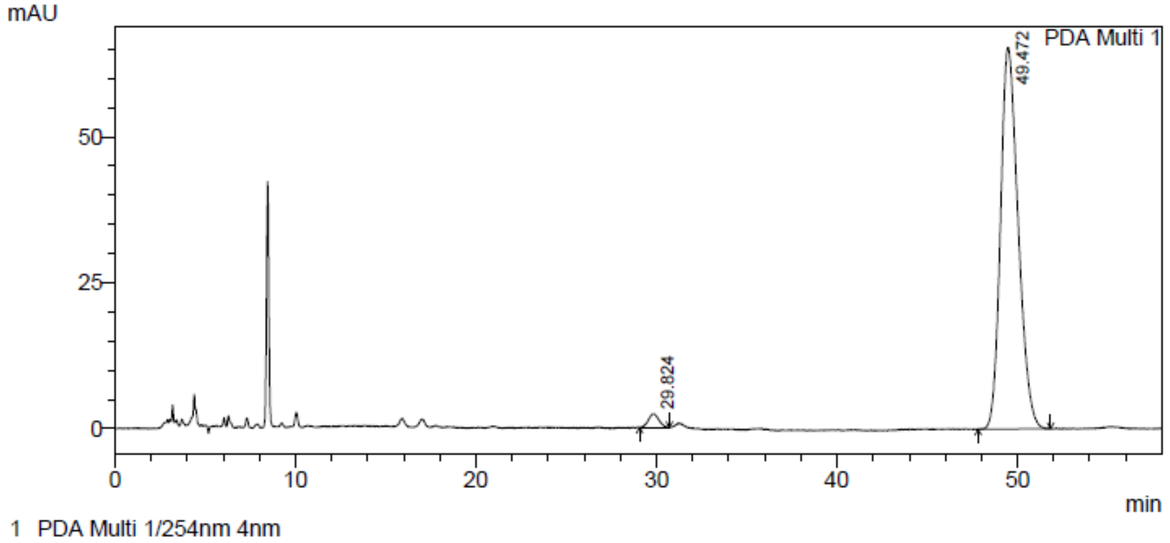

PeakTable

PDA Ch1 254nm 4nm

Peak#	Ret. Time	Area	Height	Area %	Height %
1	29.824	92581	2315	2.039	3.418
2	49.472	4447641	65405	97.961	96.582
Total		4540222	67720	100.000	100.000

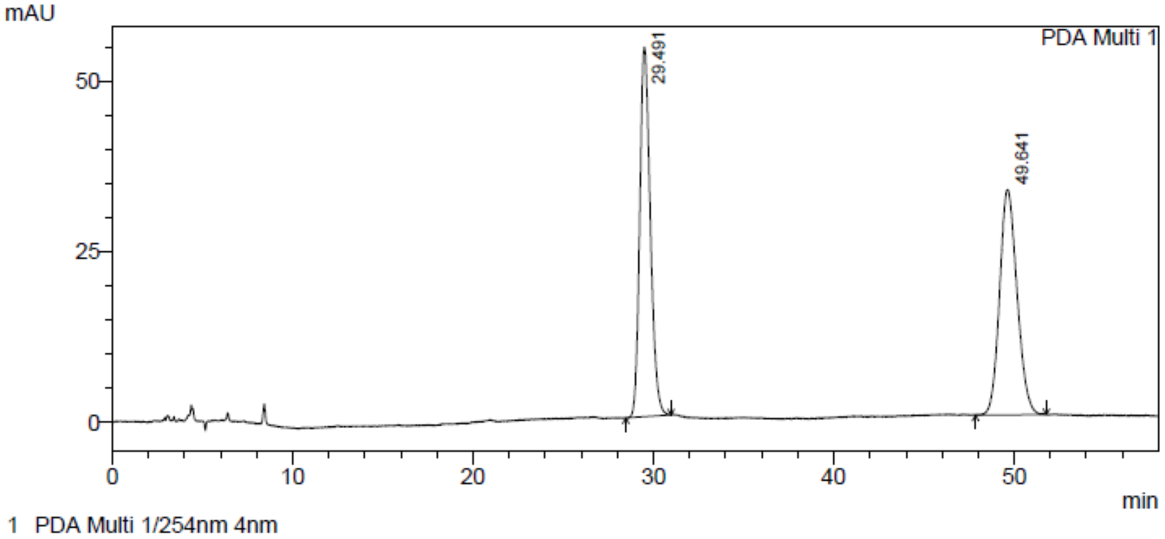

PeakTable

PDA Ch1 254nm 4nm

Peak#	Ret. Time	Area	Height	Area %	Height %
1	29.491	2204131	54292	49.693	62.119
2	49.641	2231326	33108	50.307	37.881
Total		4435457	87400	100.000	100.000

^1H NMR (400 MHz, $CDCl_3$)

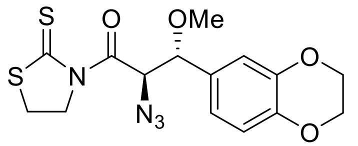

5h

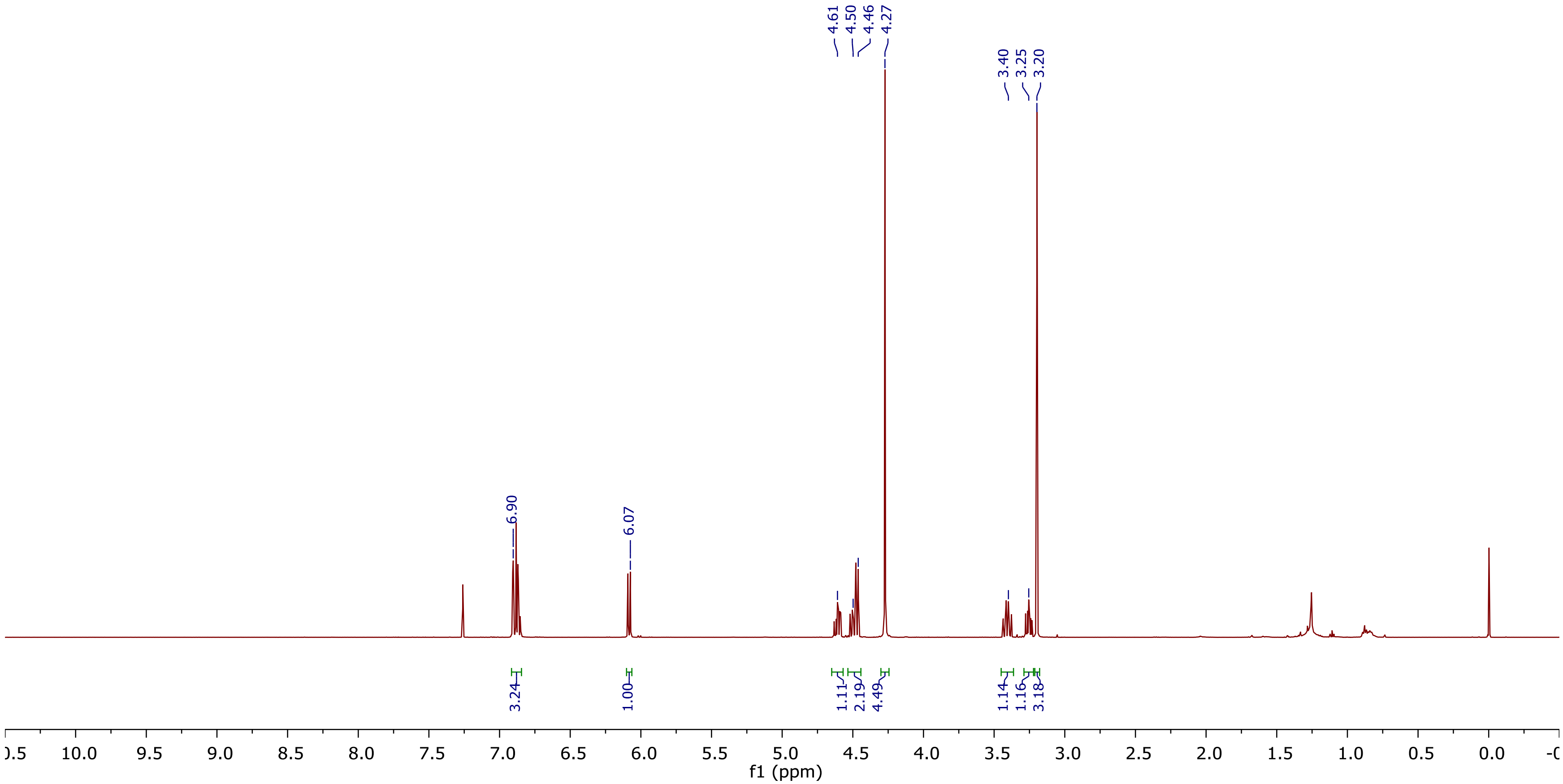

$^{13}C\{^1H\}$ NMR (100.6 MHz, $CDCl_3$)

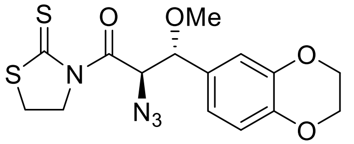

5h

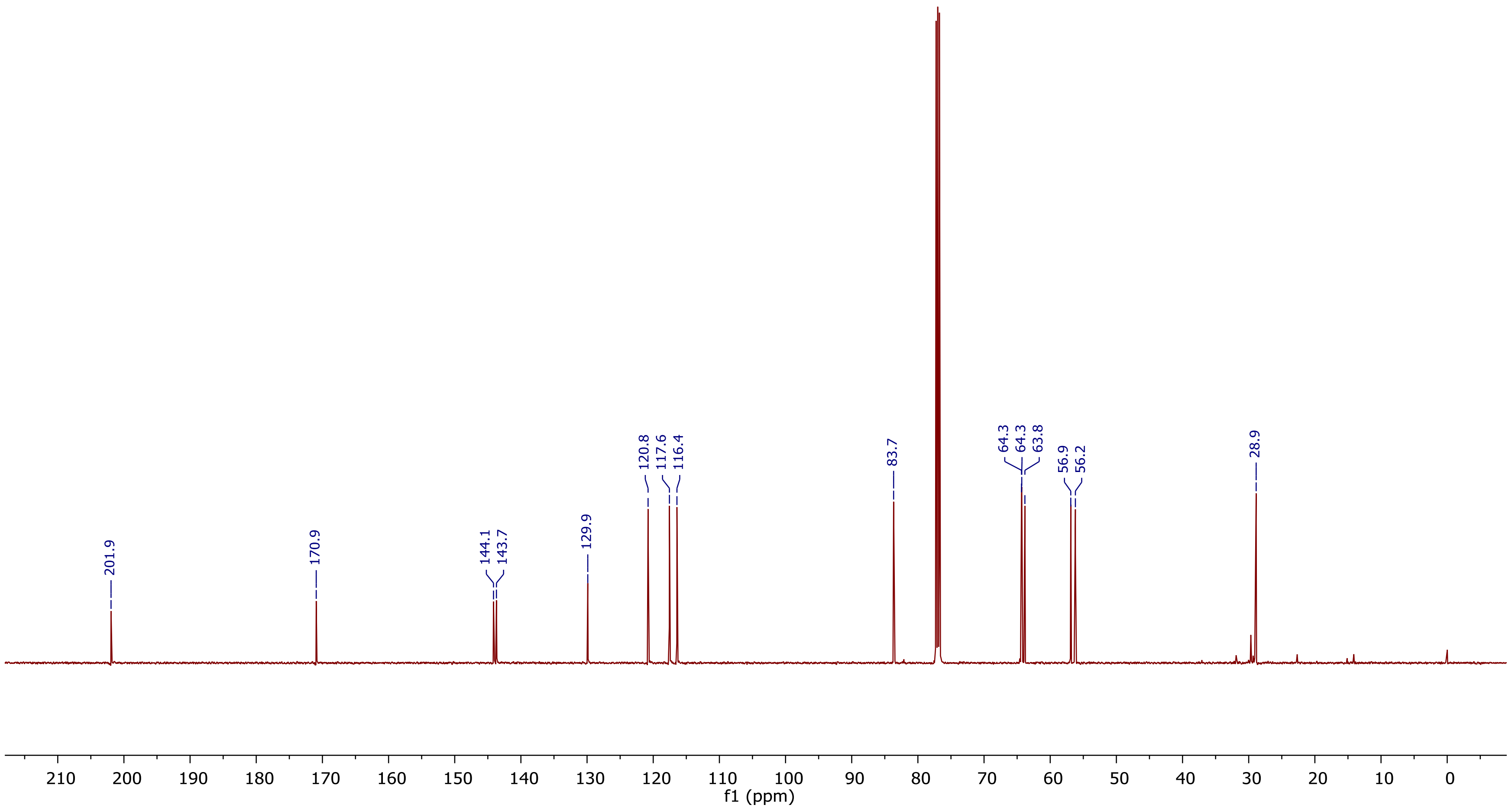

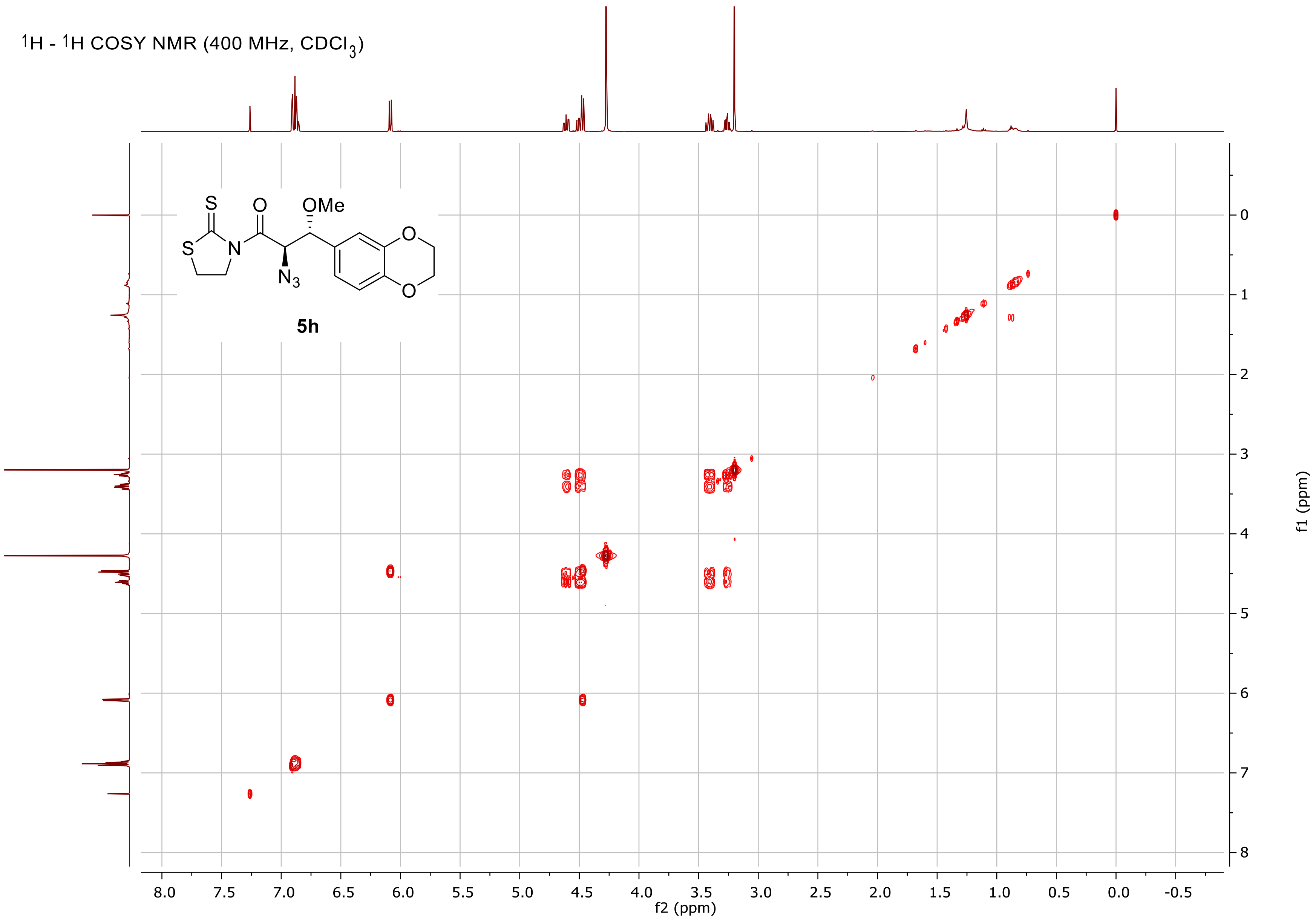

1H - 1H COSY NMR (400 MHz, CDCl3)
S
O
OMe
S
N
N3
O
O
5h
f2 (ppm)
f1 (ppm)

^{1}H - ^{13}C HSQC NMR (400 MHz, $CDCl_3$)

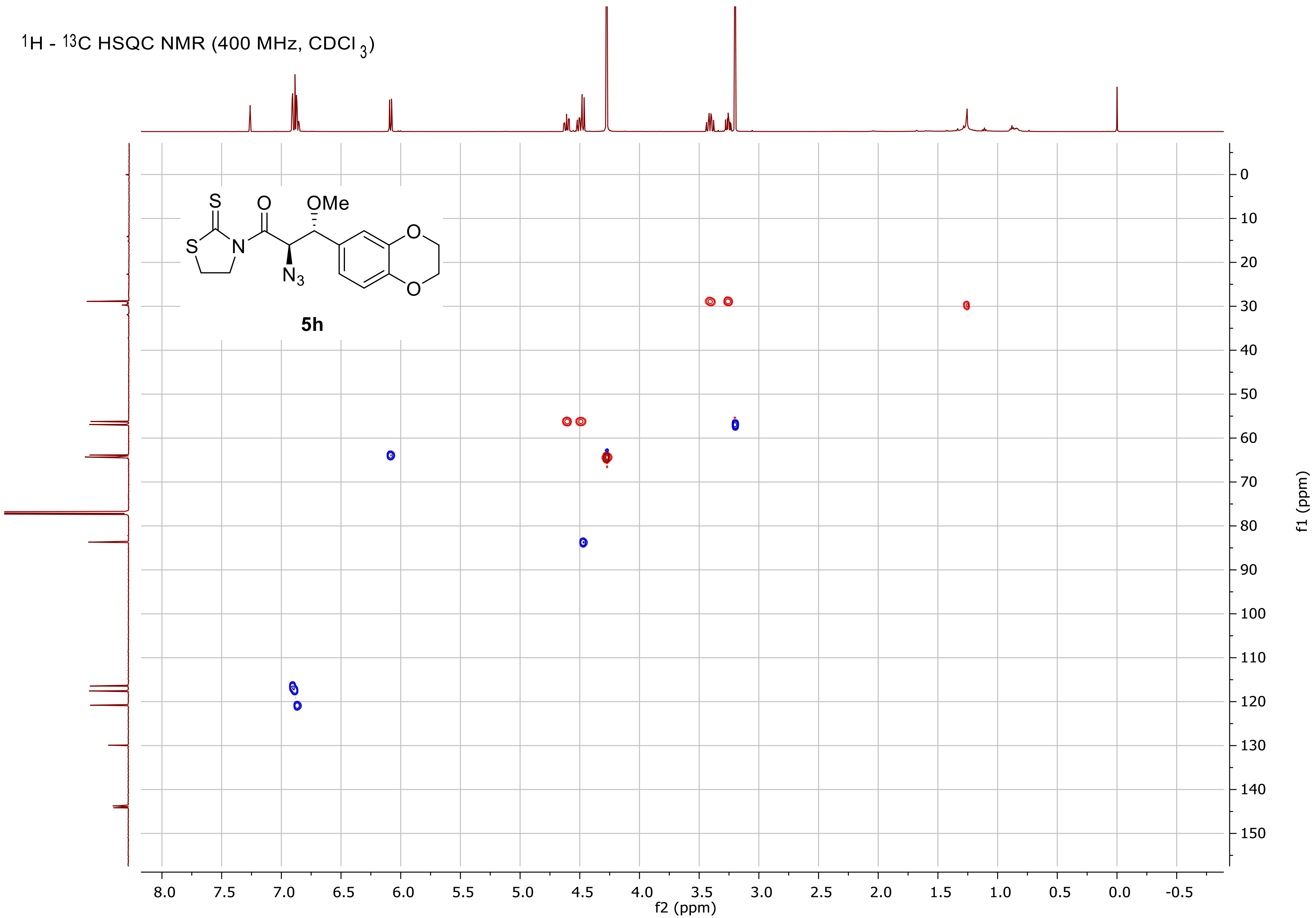

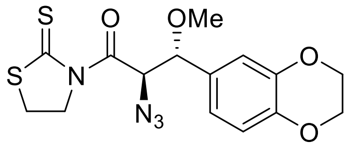

5h

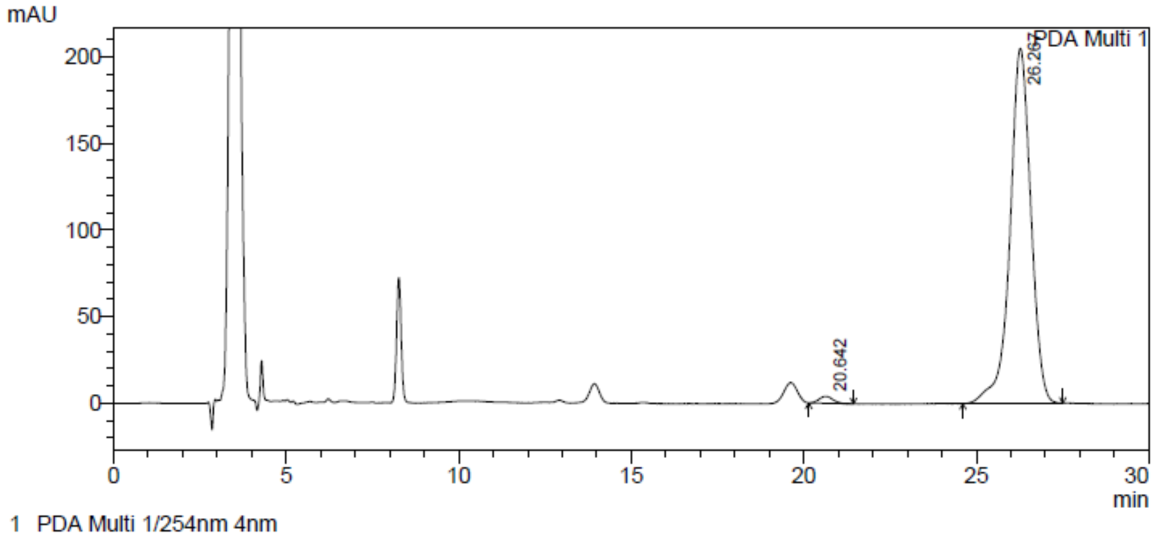

PeakTable

PDA Ch1 254nm 4nm

Peak#	Ret. Time	Area	Height	Area %	Height %
1	20.642	108056	3933	1.236	1.883
2	26.267	8636421	204900	98.764	98.117
Total		8744477	208833	100.000	100.000

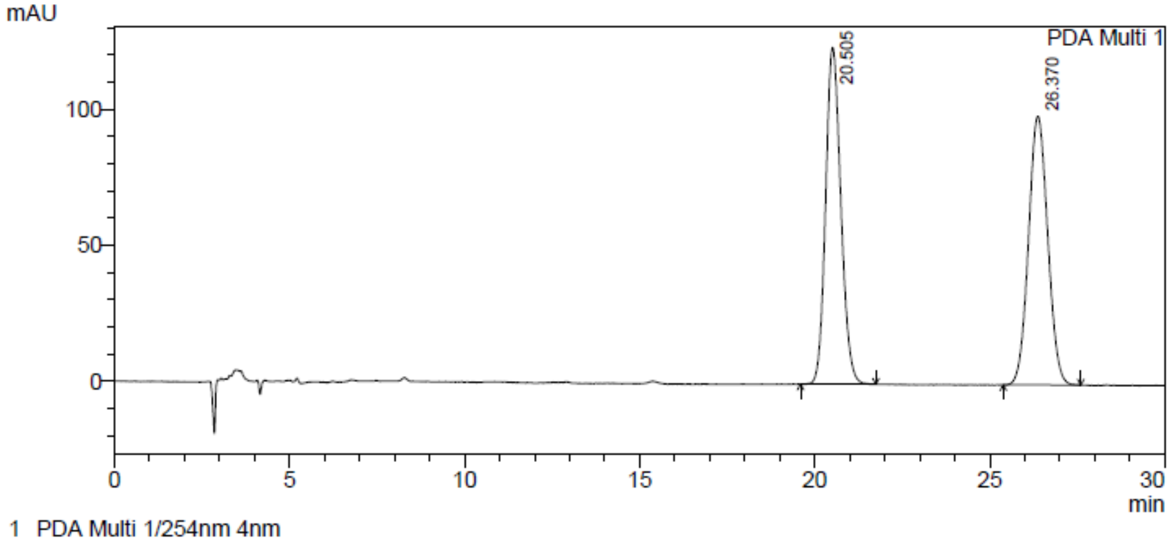

PeakTable

PDA Ch1 254nm 4nm

Peak#	Ret. Time	Area	Height	Area %	Height %
1	20.505	3753370	123905	49.936	55.618
2	26.370	3763055	98874	50.064	44.382
Total		7516425	222779	100.000	100.000

^1H NMR (400 MHz, $CDCl_3$)

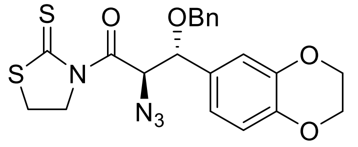

5i

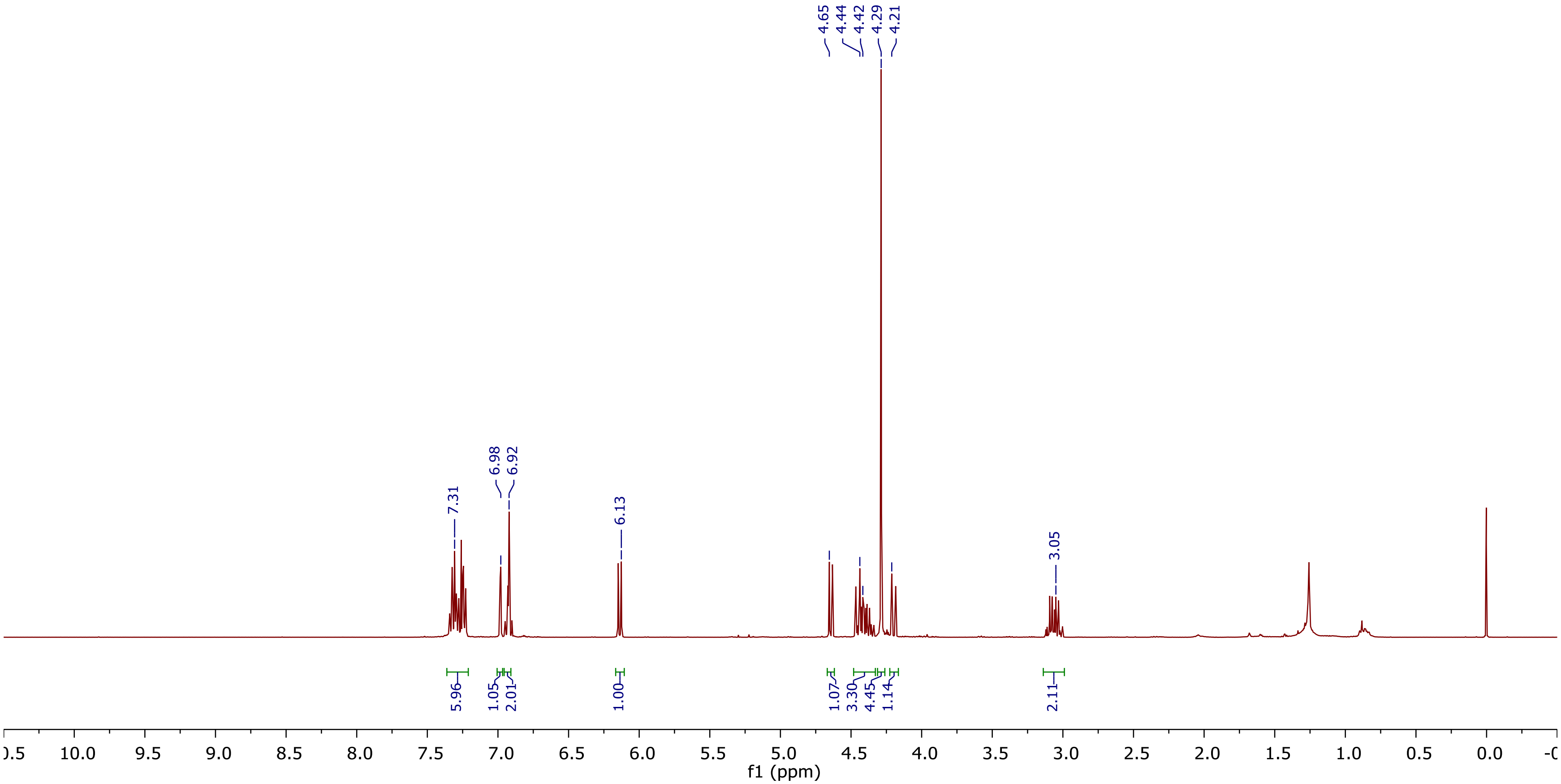

$^{13}C\{^{1}H\}$ NMR (100.6 MHz, $CDCl_3$)

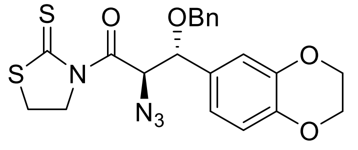

5i

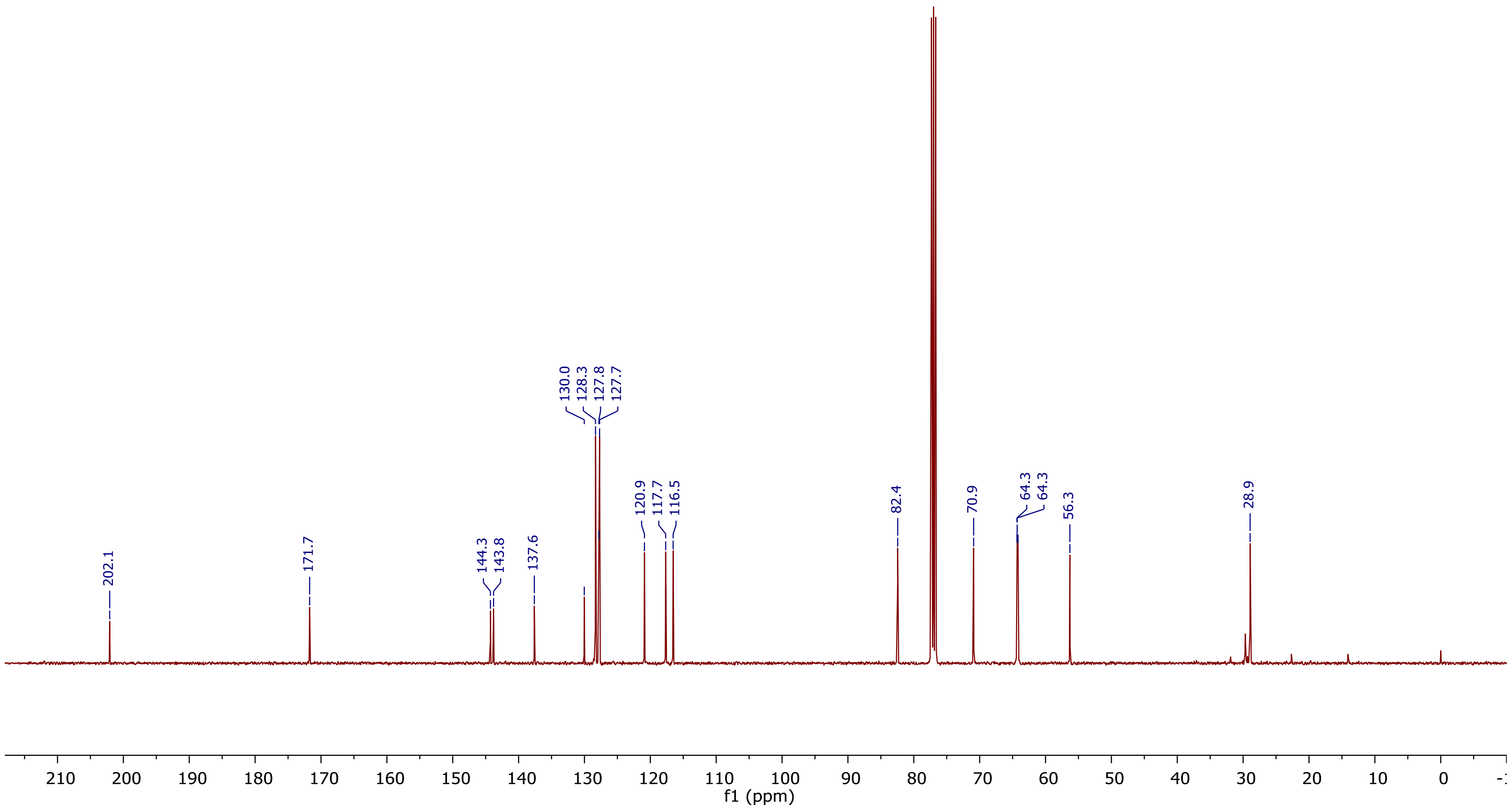

^1H - ^1H COSY NMR (400 MHz, $CDCl_3$)

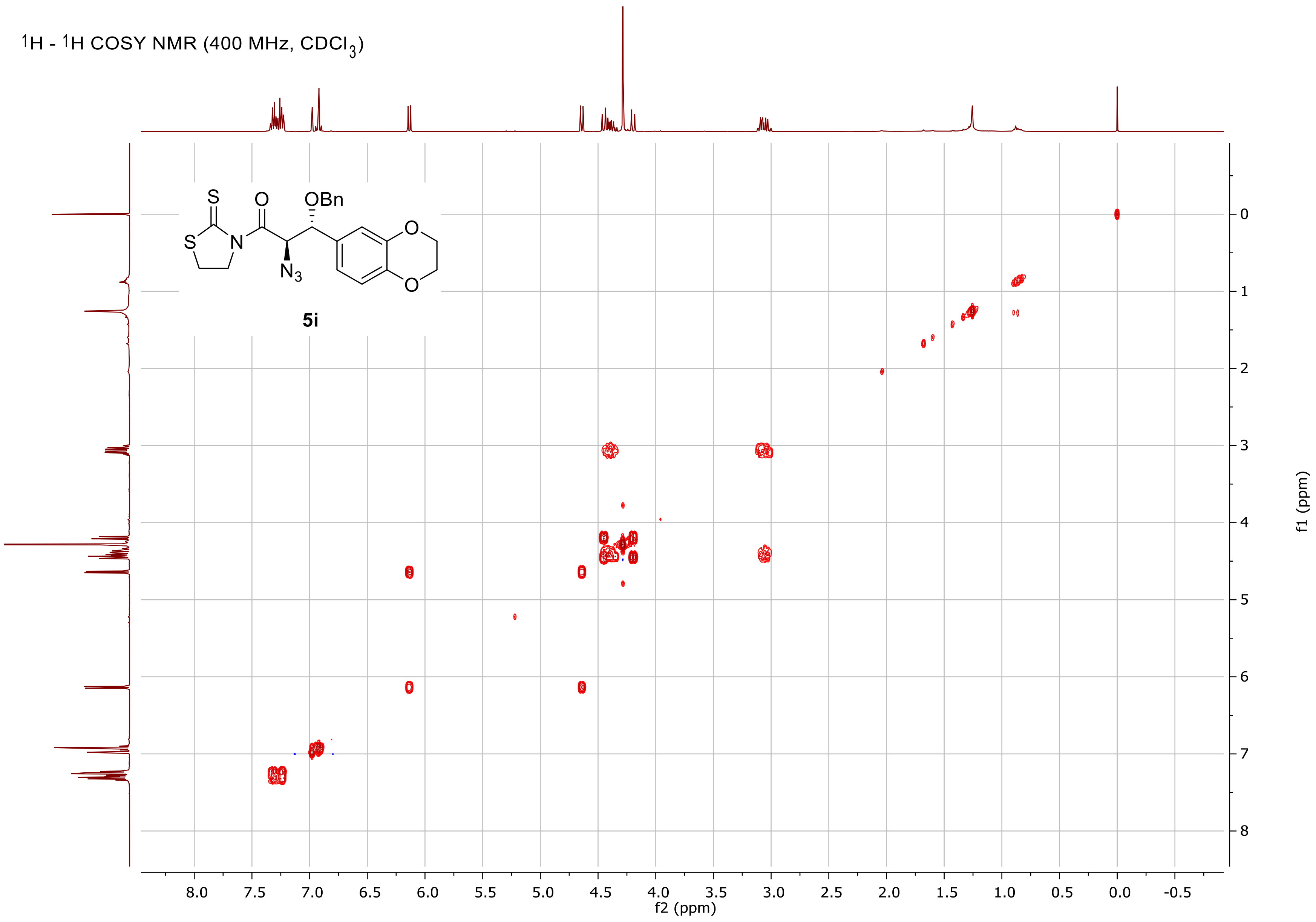

^{1}H - ^{13}C HSQC NMR (400 MHz, $CDCl_3$)

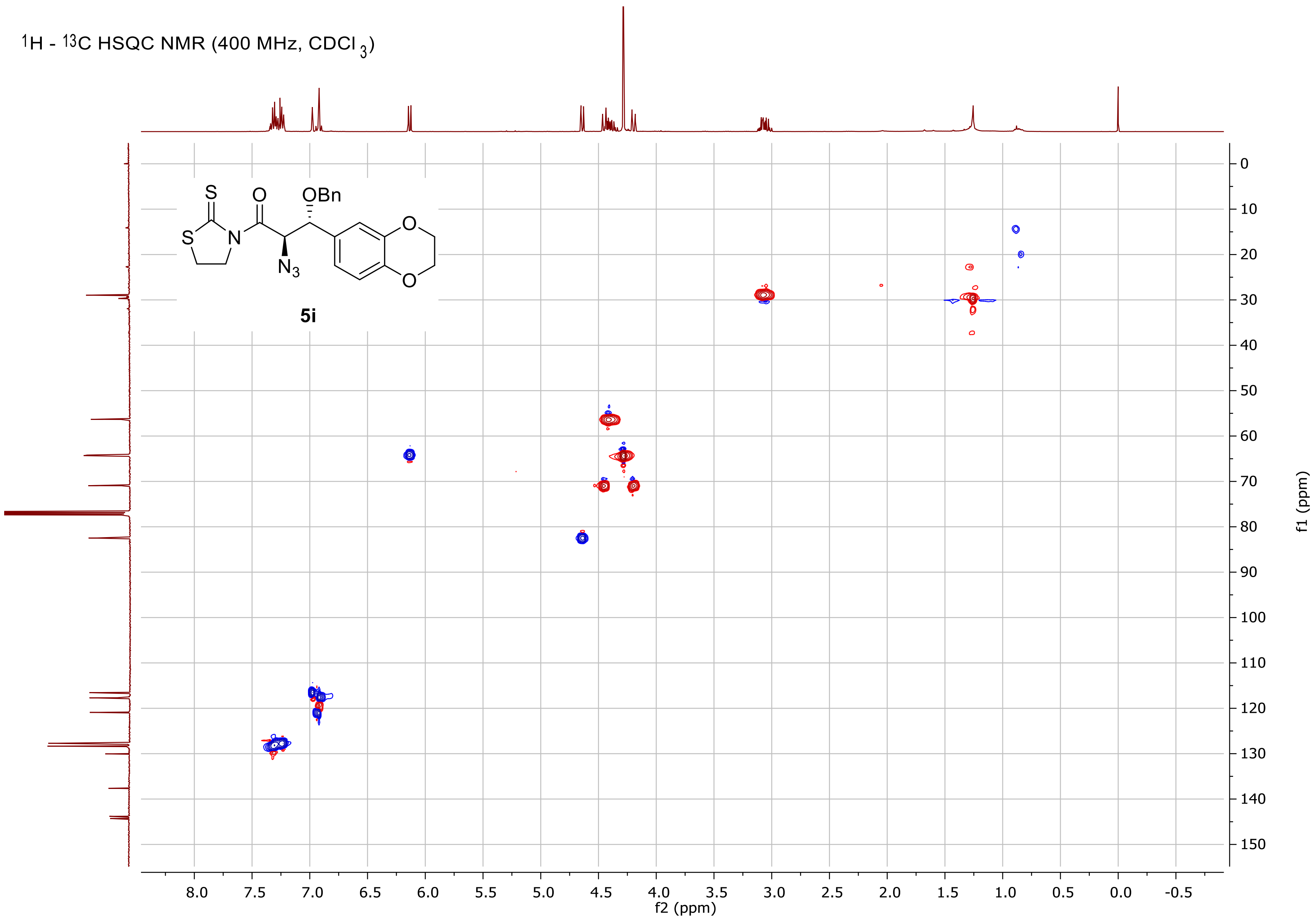

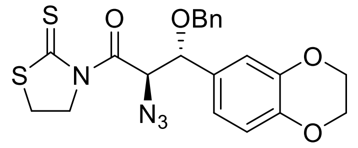

5i

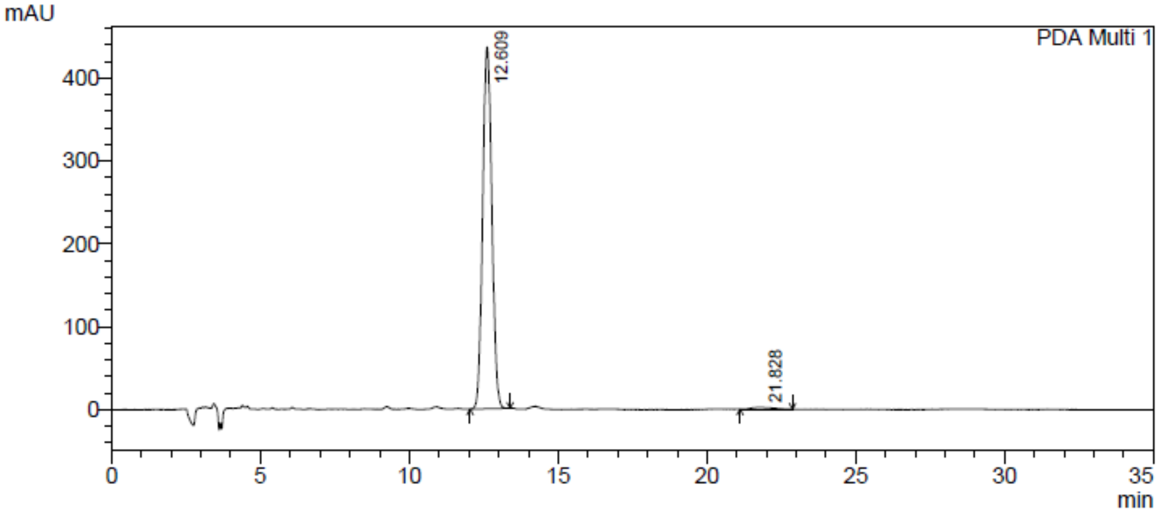

1 PDA Multi 1/254nm 4nm

PeakTable

PDA Ch1 254nm 4nm

Peak#	Ret. Time	Area	Height	Area %	Height %
1	12.609	9366188	436224	98.896	99.474
2	21.828	104528	2305	1.104	0.526
Total		9470716	438530	100.000	100.000

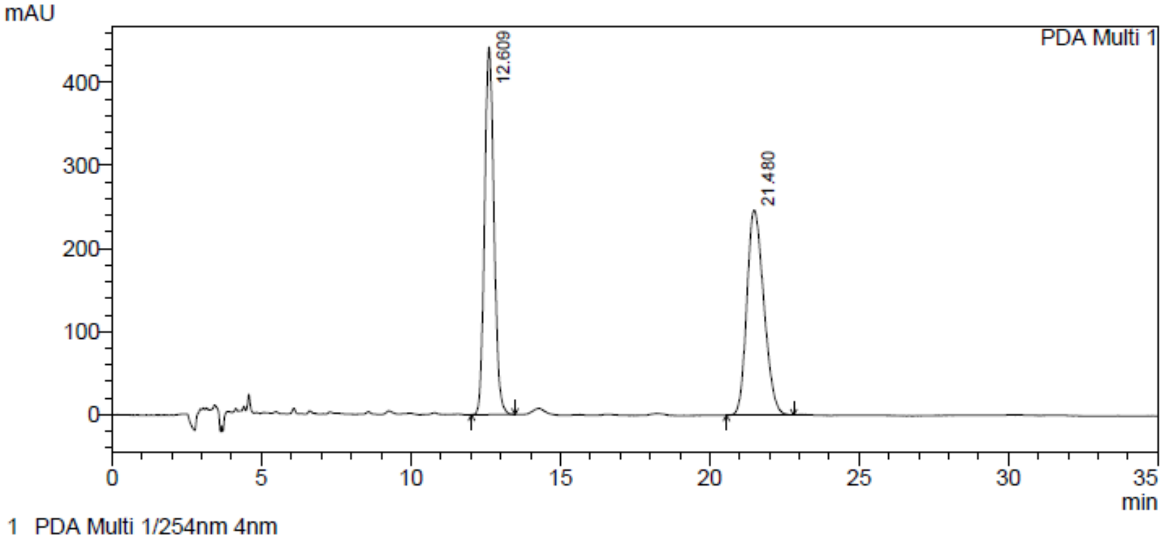

1 PDA Multi 1/254nm 4nm

PeakTable

PDA Ch1 254nm 4nm

Peak#	Ret. Time	Area	Height	Area %	Height %
1	12.609	9670046	442031	49.766	64.172
2	21.480	9760870	246792	50.234	35.828
Total		19430916	688823	100.000	100.000

^{1}H NMR (400 MHz, $CDCl_3$)

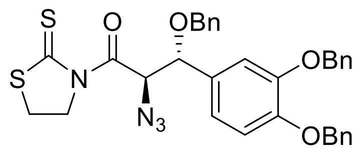

5j

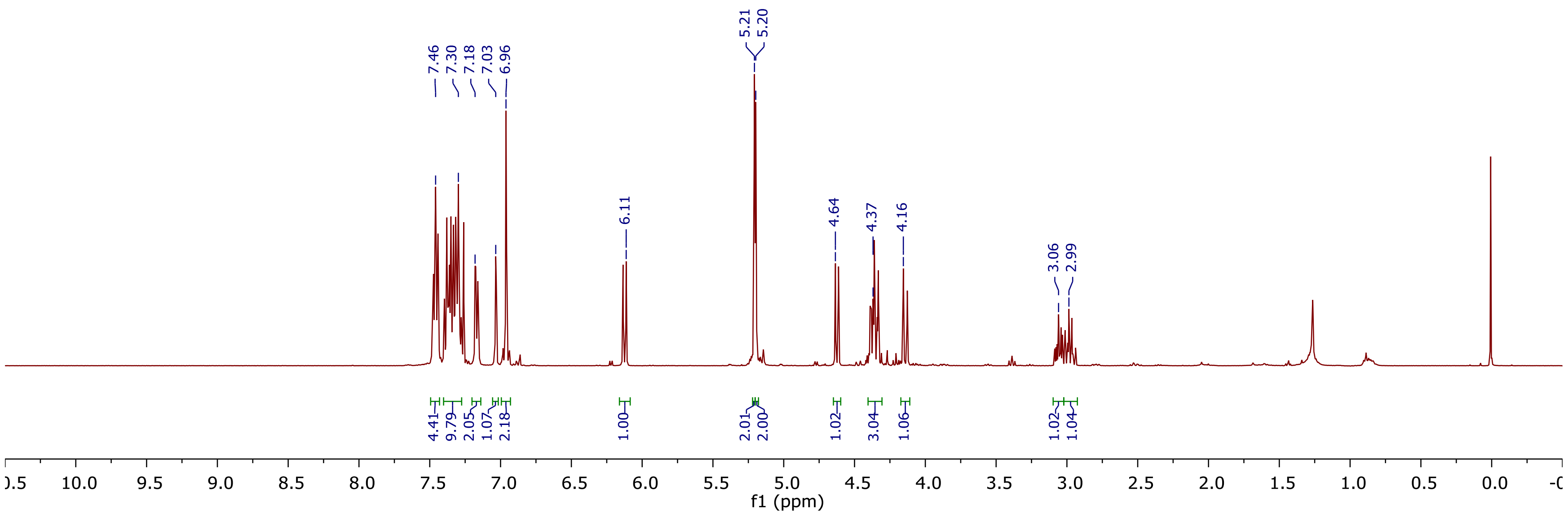

$^{13}C\{^1H\}$ NMR (100.6 MHz, $CDCl_3$)

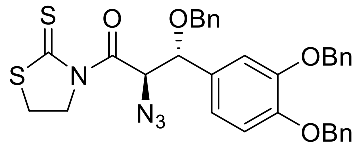

5j

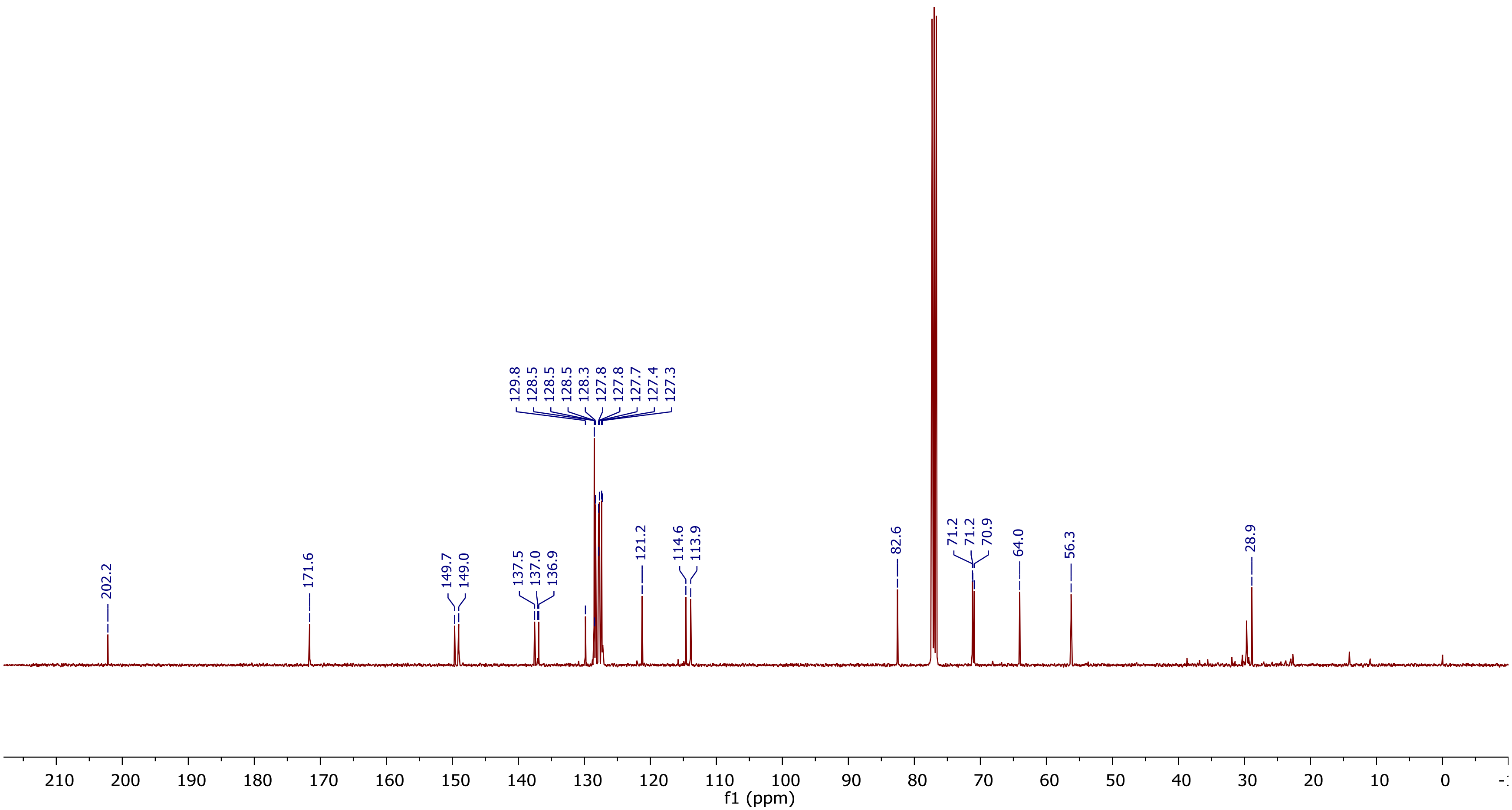

^1H - ^1H COSY NMR (400 MHz, $CDCl_3$)

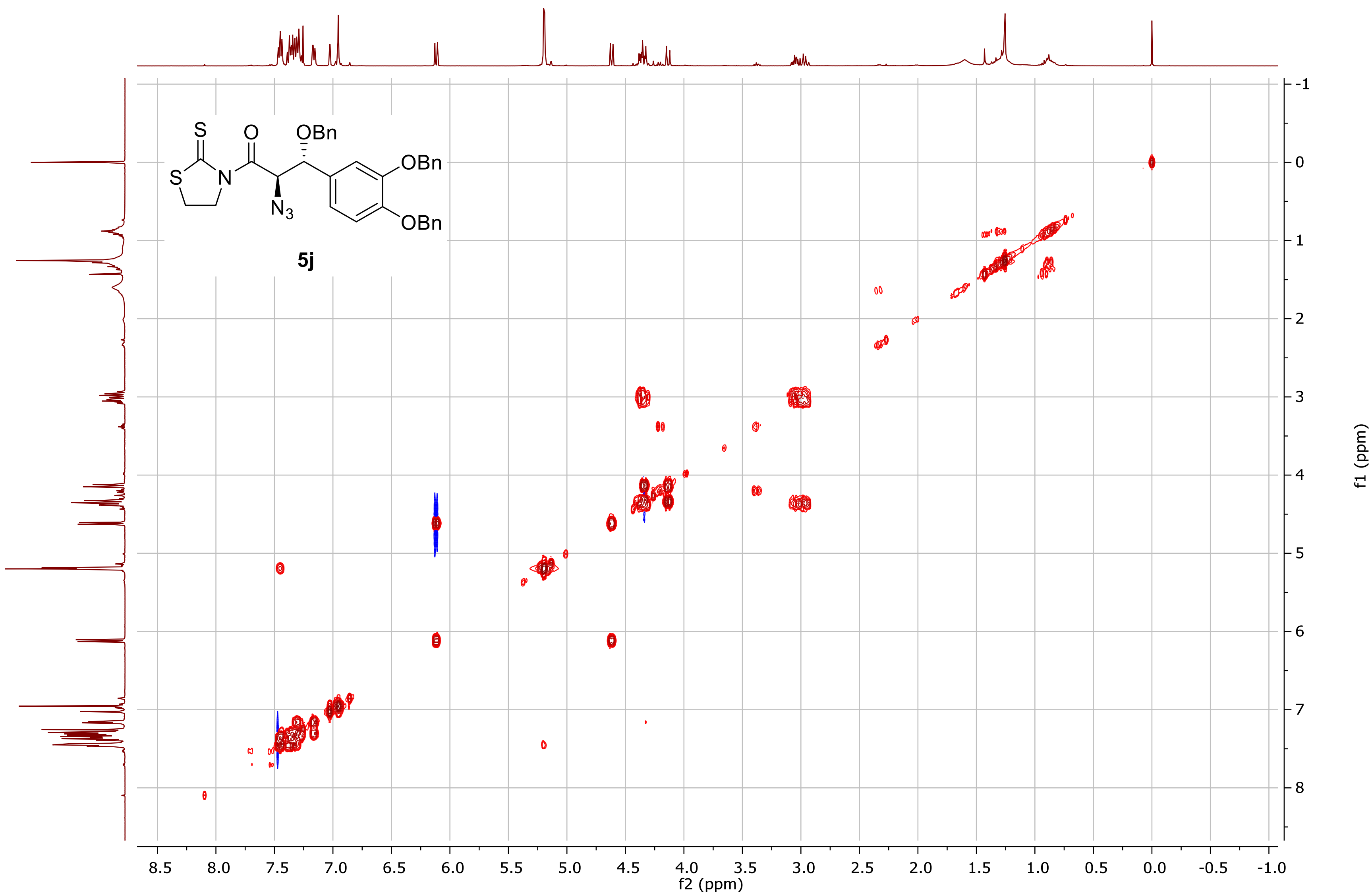

^{1}H - ^{13}C HSQC NMR (400 MHz, $CDCl_3$)

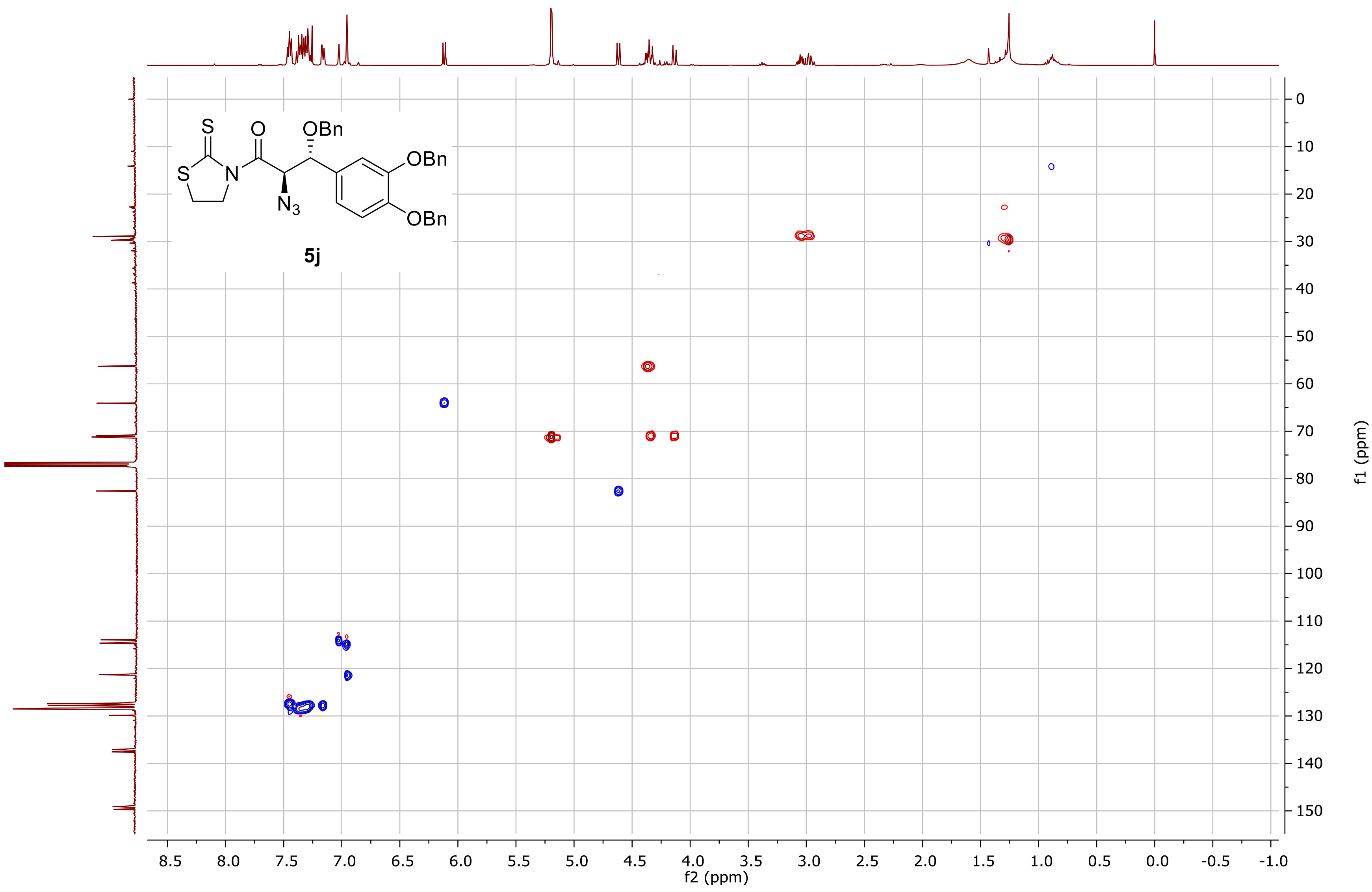

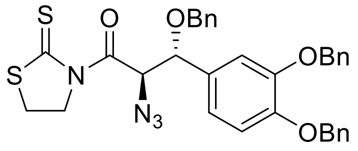

5j

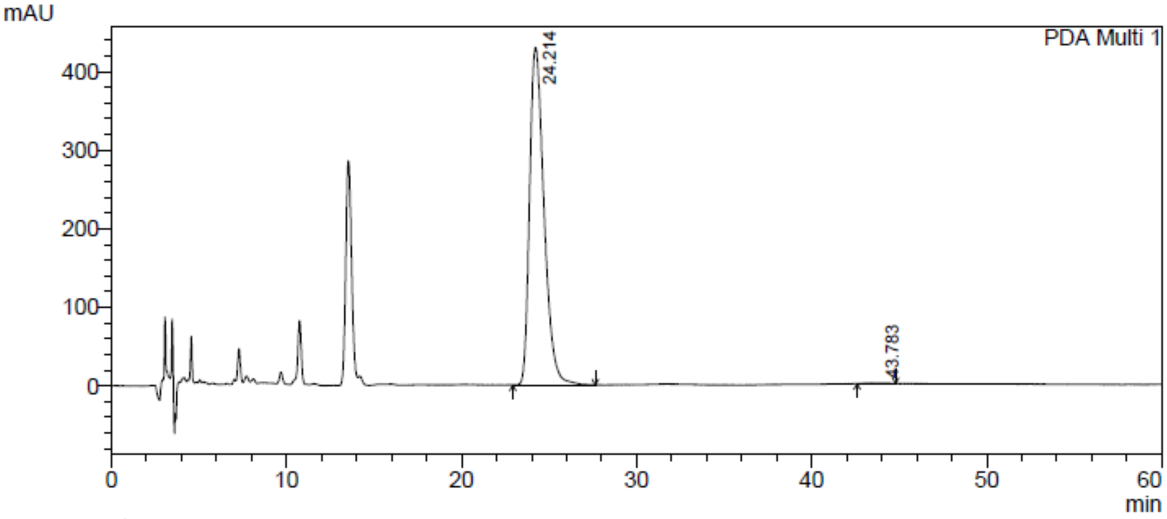

1 PDA Multi 1/254nm 4nm

PeakTable

PDA Ch1 254nm 4nm

Peak#	Ret. Time	Area	Height	Area %	Height %
1	24.214	24573664	429438	99.757	99.800
2	43.783	59757	860	0.243	0.200
Total		24633421	430298	100.000	100.000

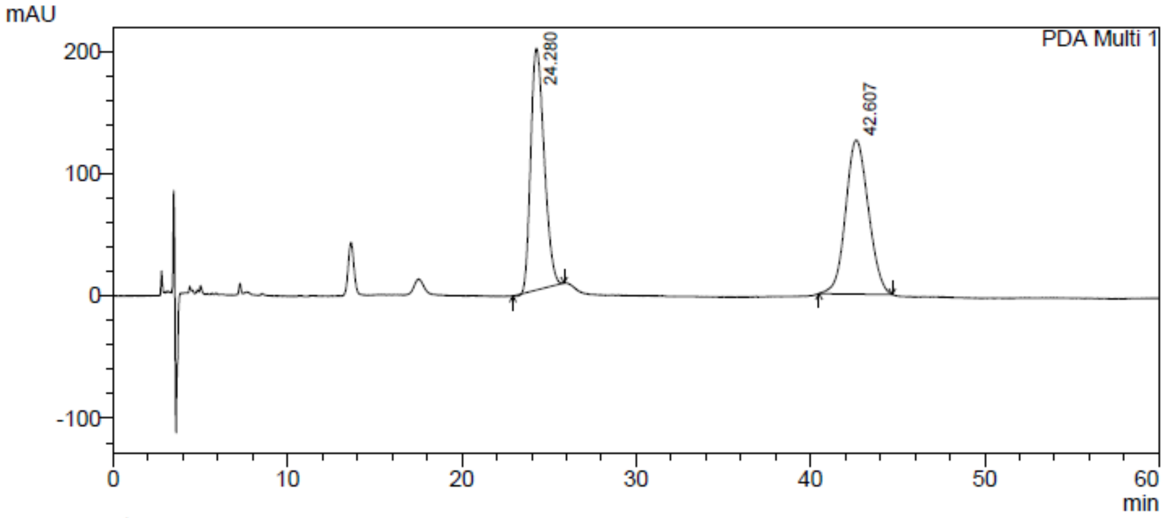

1 PDA Multi 1/254nm 4nm

PeakTable

PDA Ch1 254nm 4nm

Peak#	Ret. Time	Area	Height	Area %	Height %
1	24.280	10602211	197545	48.078	61.092
2	42.607	11449788	125812	51.922	38.908
Total		22052000	323358	100.000	100.000

^1H NMR (400 MHz, $CDCl_3$)

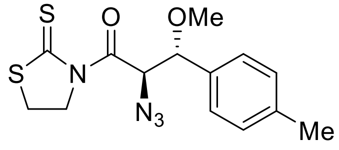

5k

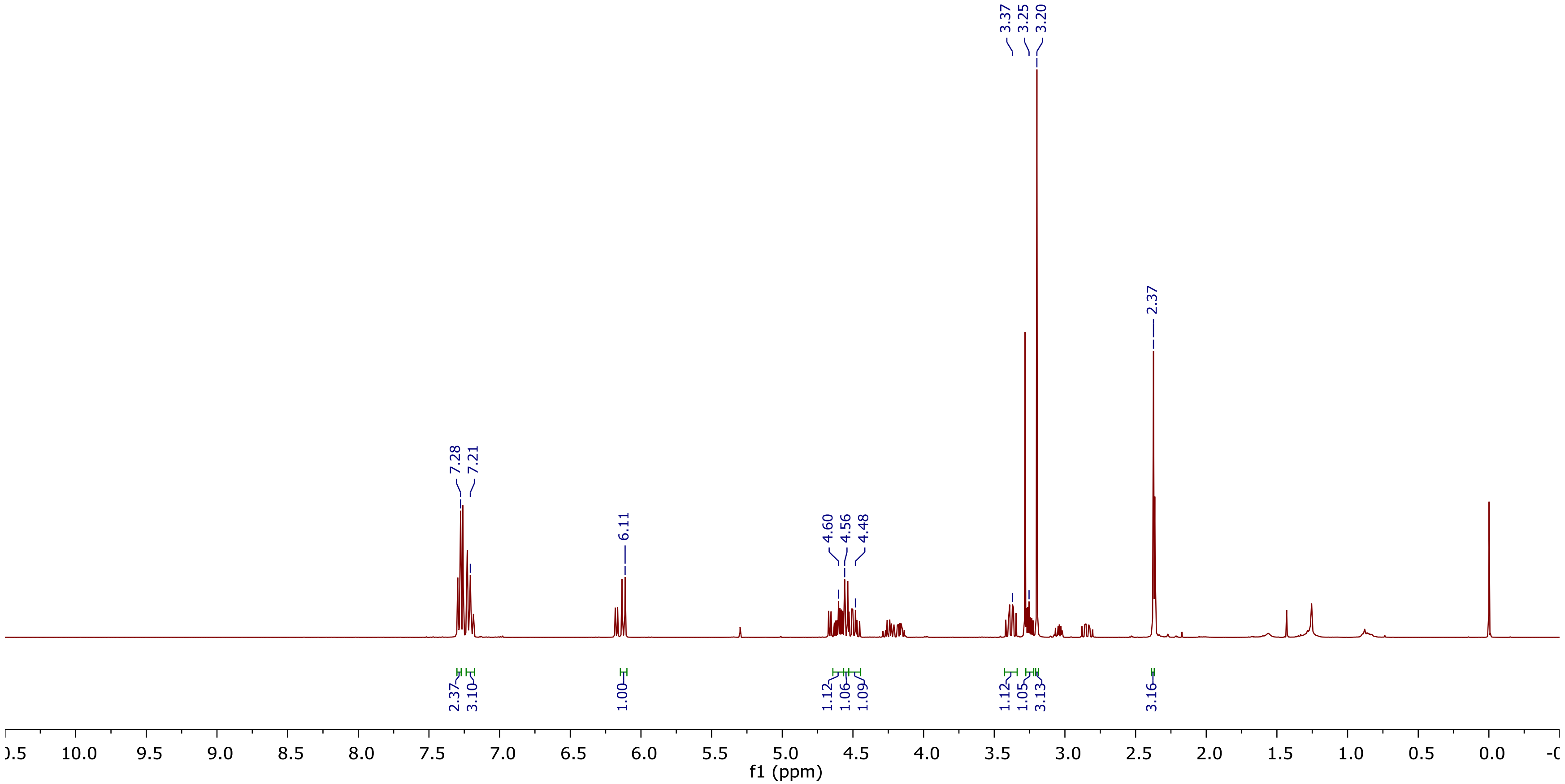

$^{13}C\{^1H\}$ NMR (100.6 MHz, $CDCl_3$)

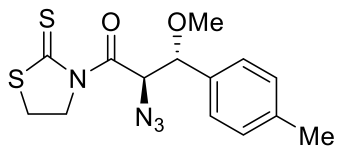

5k

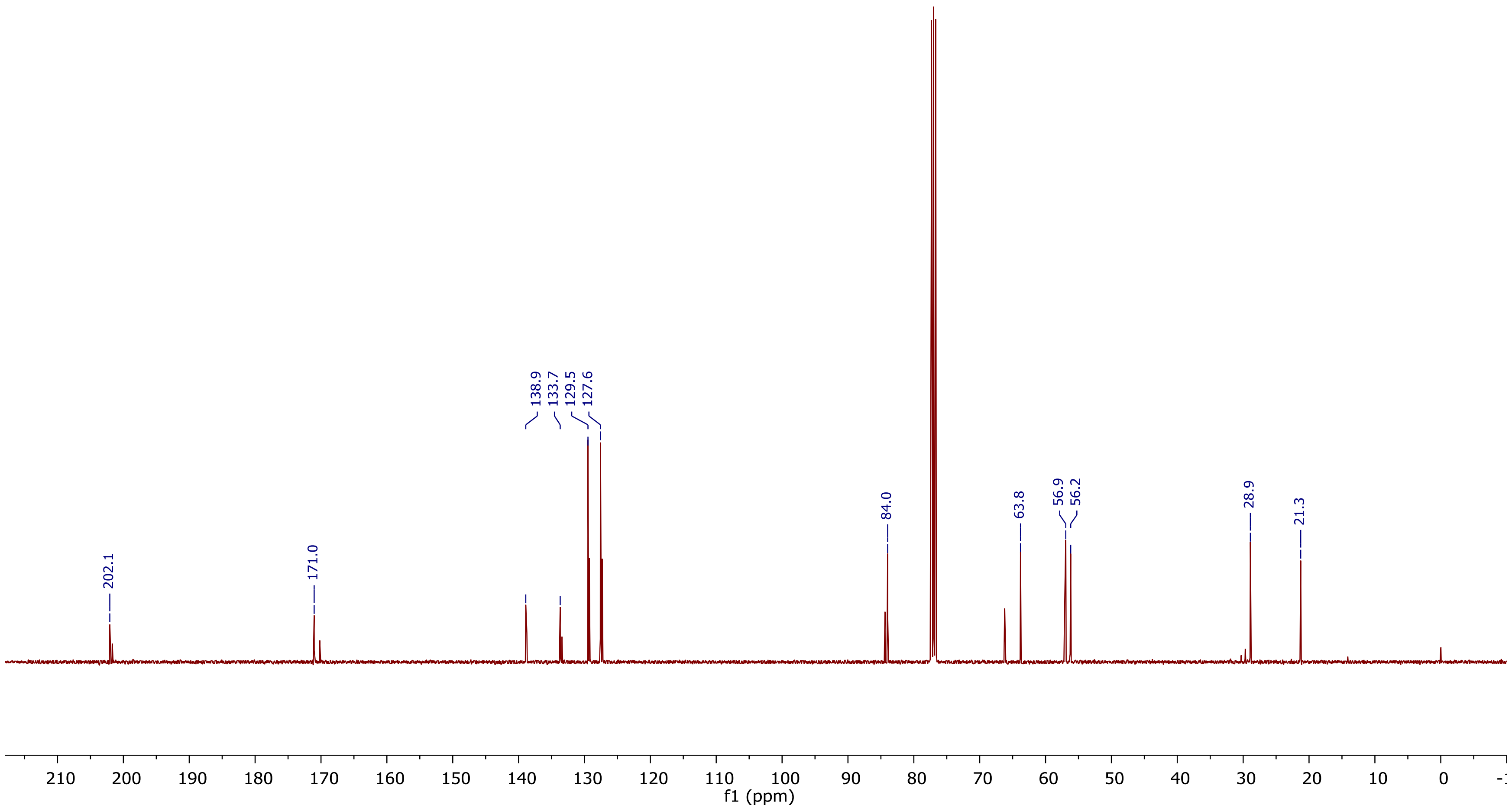

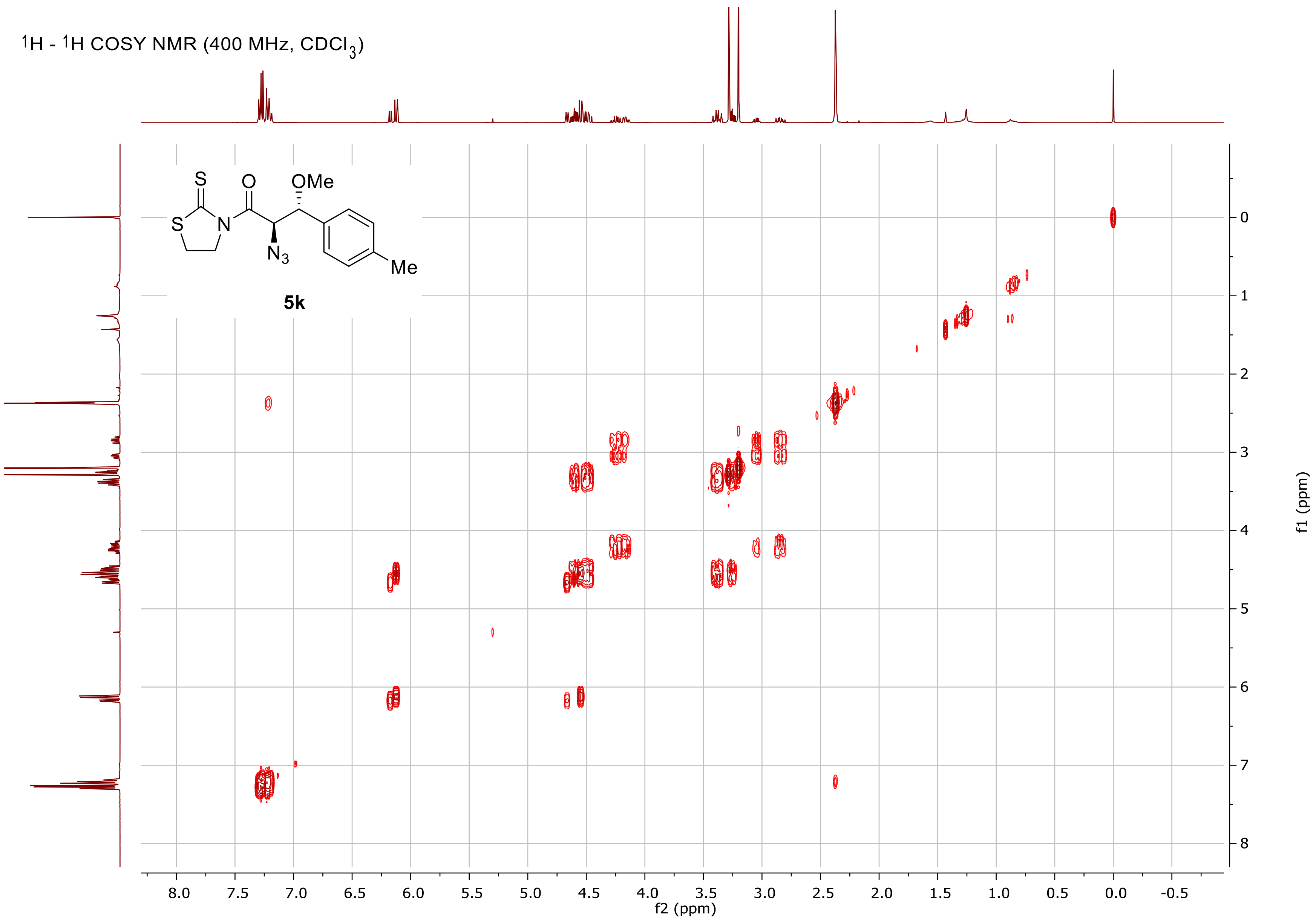

^{1}H - ^{1}H COSY NMR (400 MHz, $CDCl_3$)
S
O
OMe
S
N
N3
Me
5k
f2 (ppm)
f1 (ppm)

^1H - ^{13}C HSQC NMR (400 MHz, $CDCl_3$)

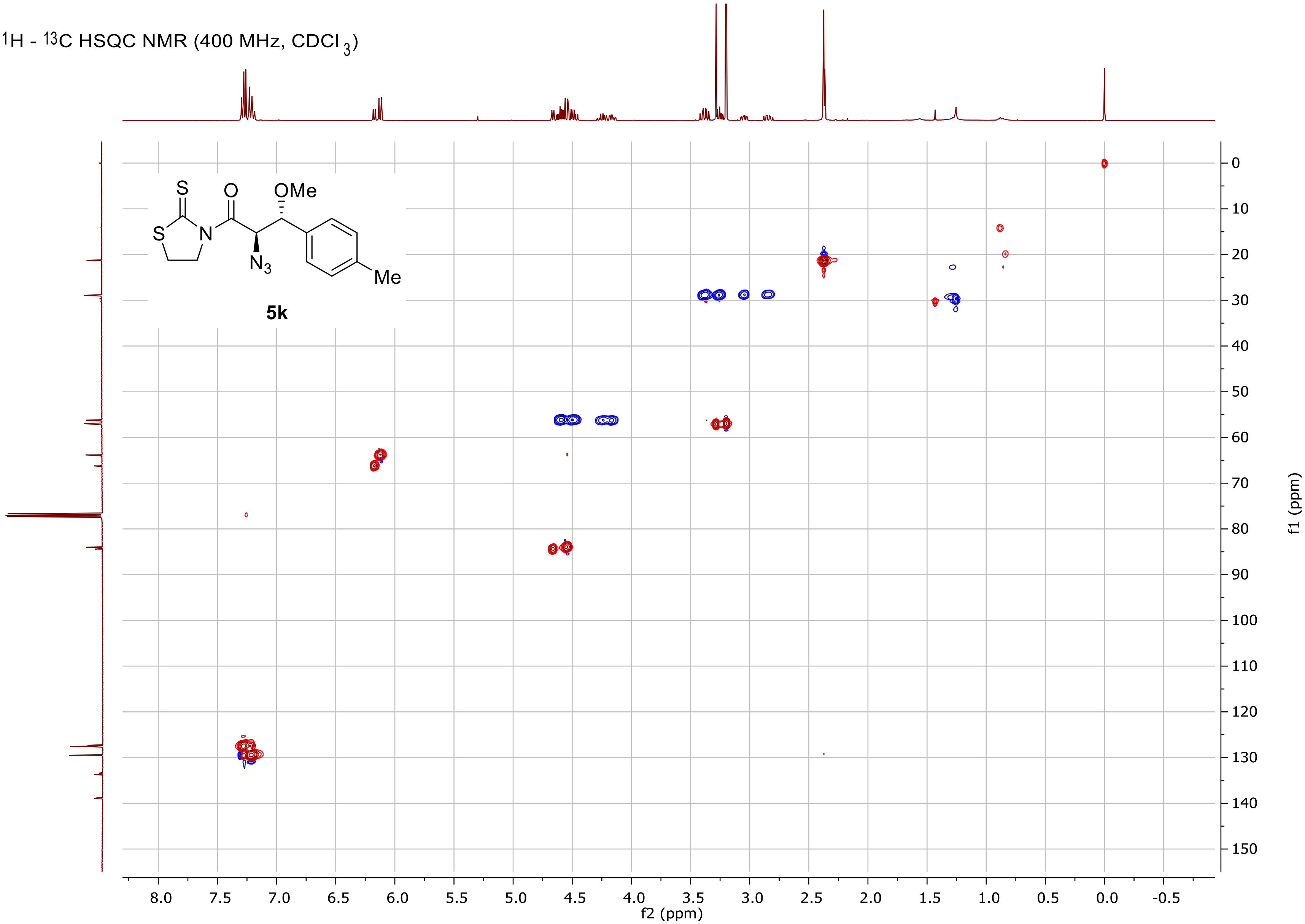

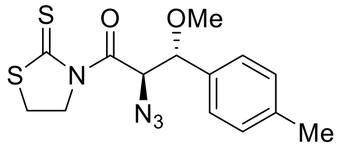

5k

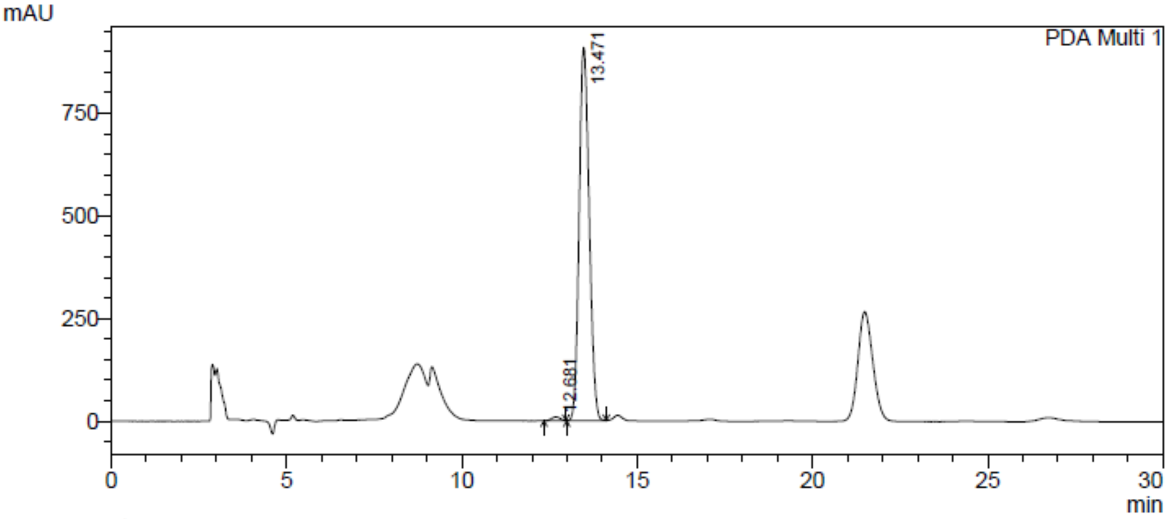

1 PDA Multi 1/254nm 4nm

PeakTable

PDA Ch1 254nm 4nm

Peak#	Ret. Time	Area	Height	Area %	Height %
1	12.681	161380	9566	0.885	1.042
2	13.471	18082372	908390	99.115	98.958
Total		18243752	917956	100.000	100.000

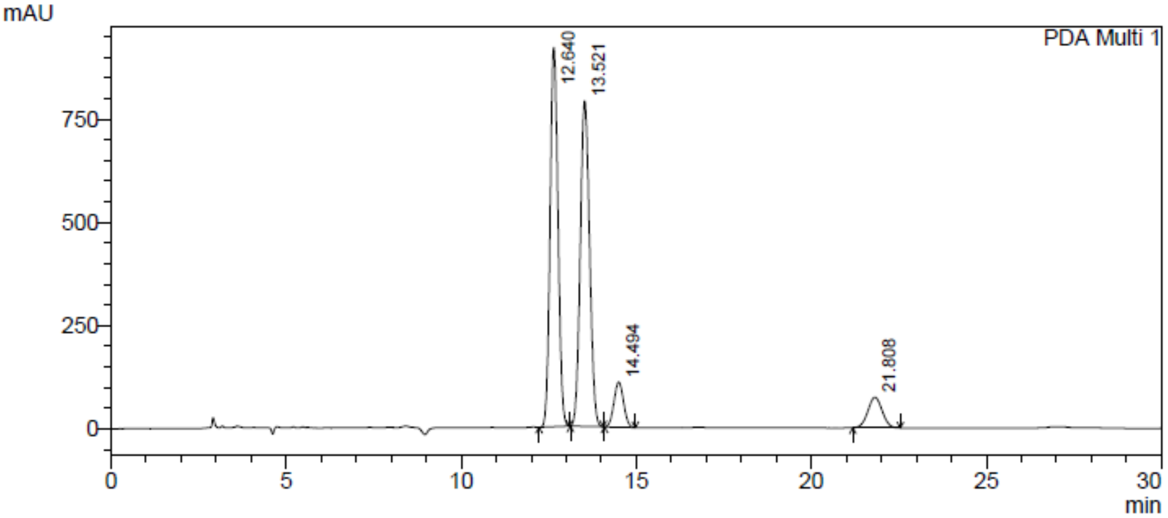

1 PDA Multi 1/254nm 4nm

PeakTable

PDA Ch1 254nm 4nm

Peak#	Ret. Time	Area	Height	Area %	Height %
1	12.640	14400160	918583	43.453	48.592
2	13.521	14540649	788304	43.877	41.701
3	14.494	2086360	109354	6.296	5.785
4	21.808	2112766	74150	6.375	3.922
Total		33139935	1890393	100.000	100.000

^1H NMR (400 MHz, $CDCl_3$)

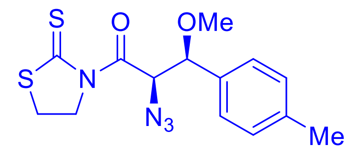

7k

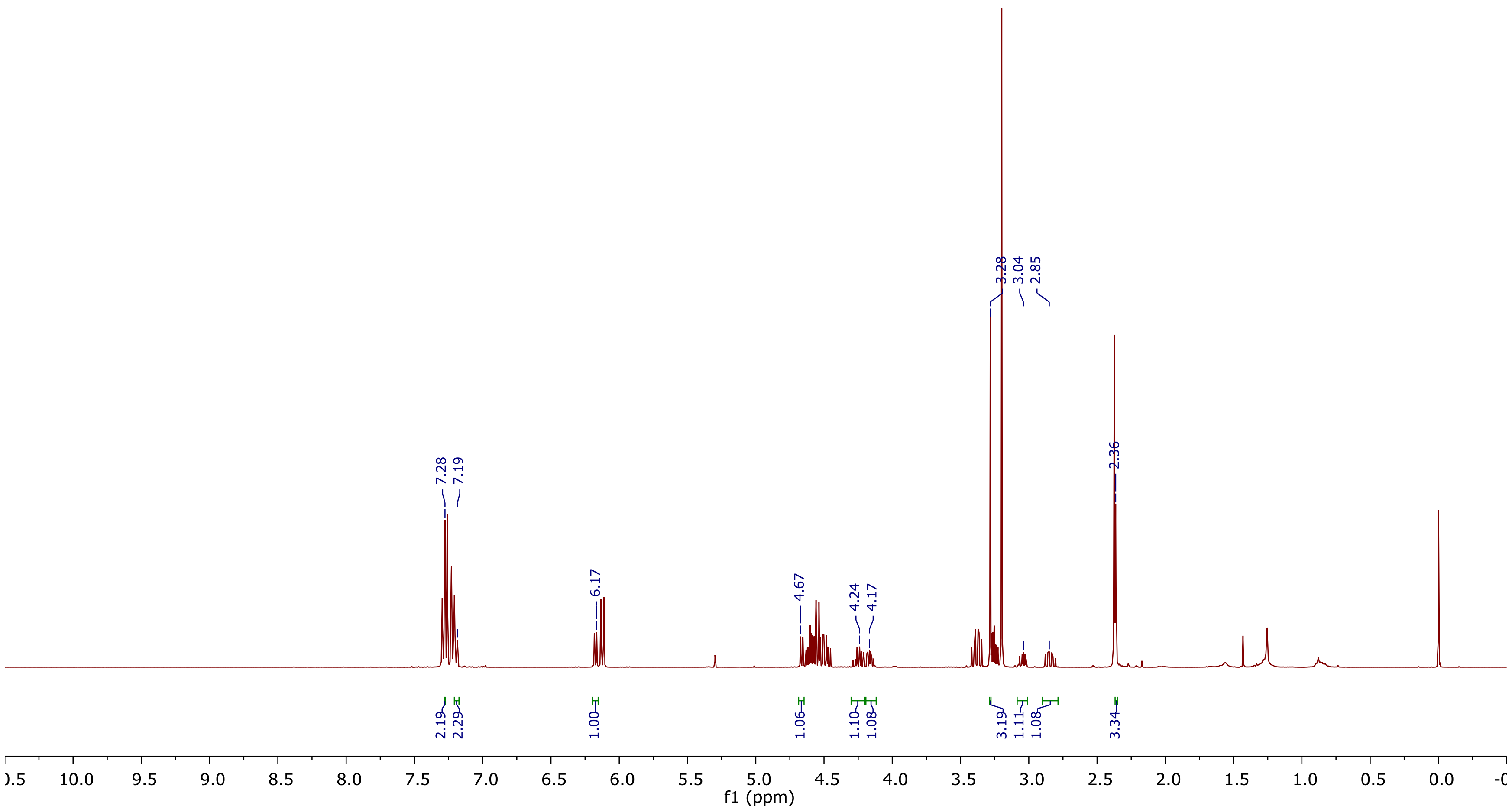

$^{13}C\{^1H\}$ NMR (100.6 MHz, $CDCl_3$)

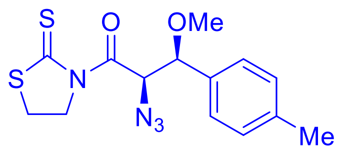

7k

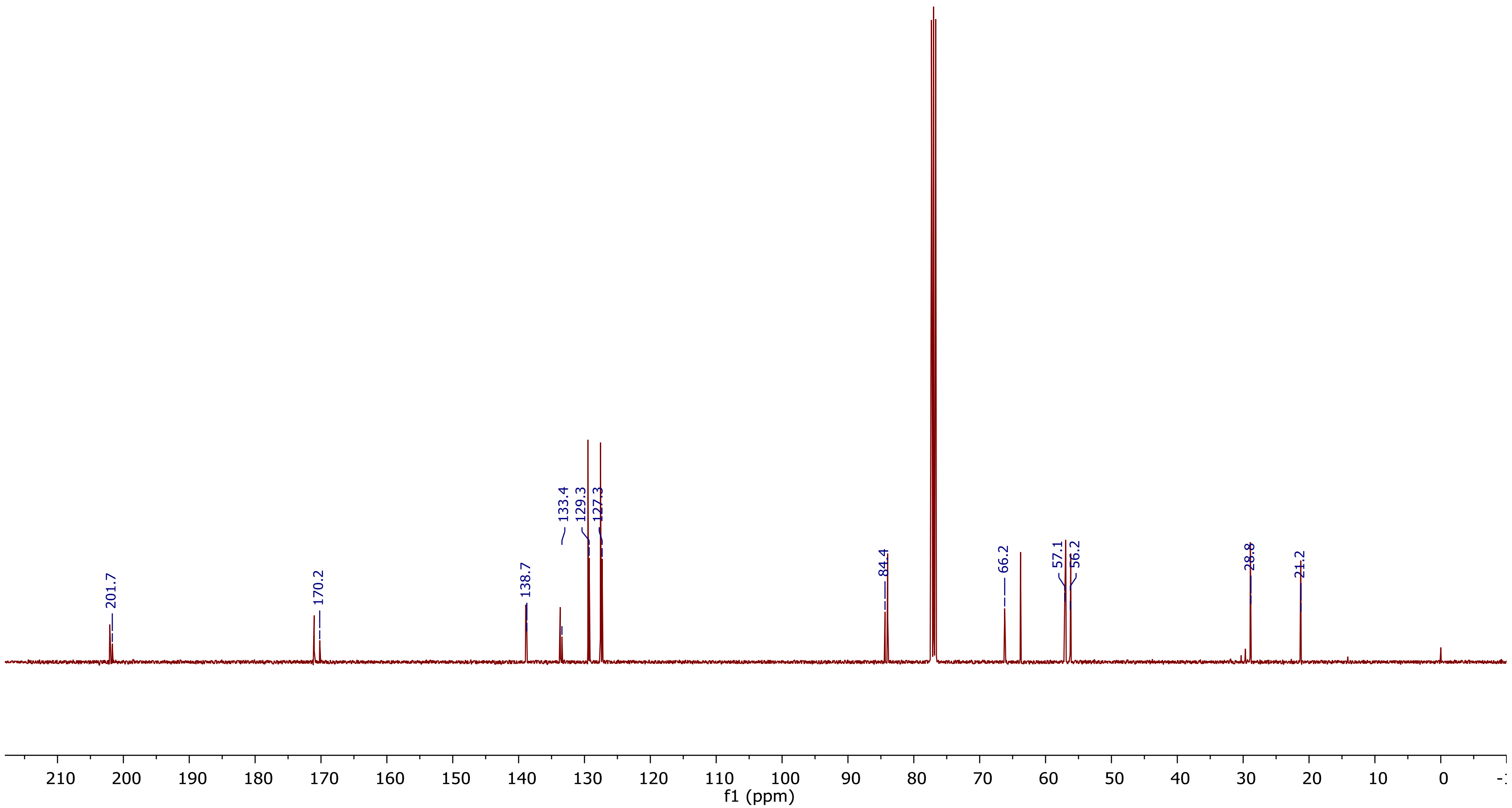

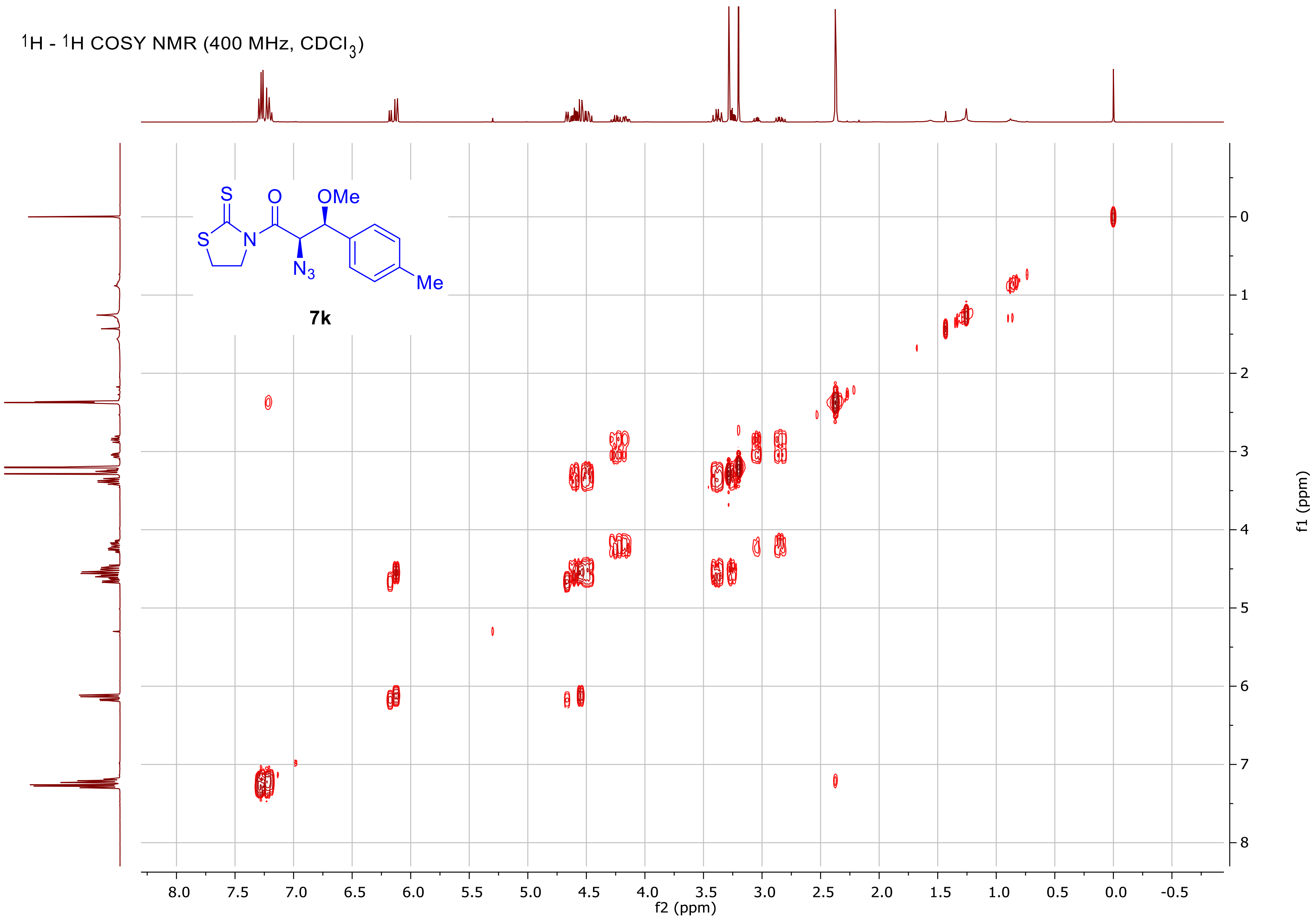
1H - 1H COSY NMR (400 MHz, CDCl3)
S
O
OMe
S
N
N3
Me
7k
8.0
7.5
7.0
6.5
6.0
5.5
5.0
4.5
4.0
3.5
3.0
2.5
2.0
1.5
1.0
0.5
0.0
-0.5
f2 (ppm)
0
1
2
3
4
5
6
7
8
f1 (ppm)

^{1}H - ^{13}C HSQC NMR (400 MHz, $CDCl_3$)

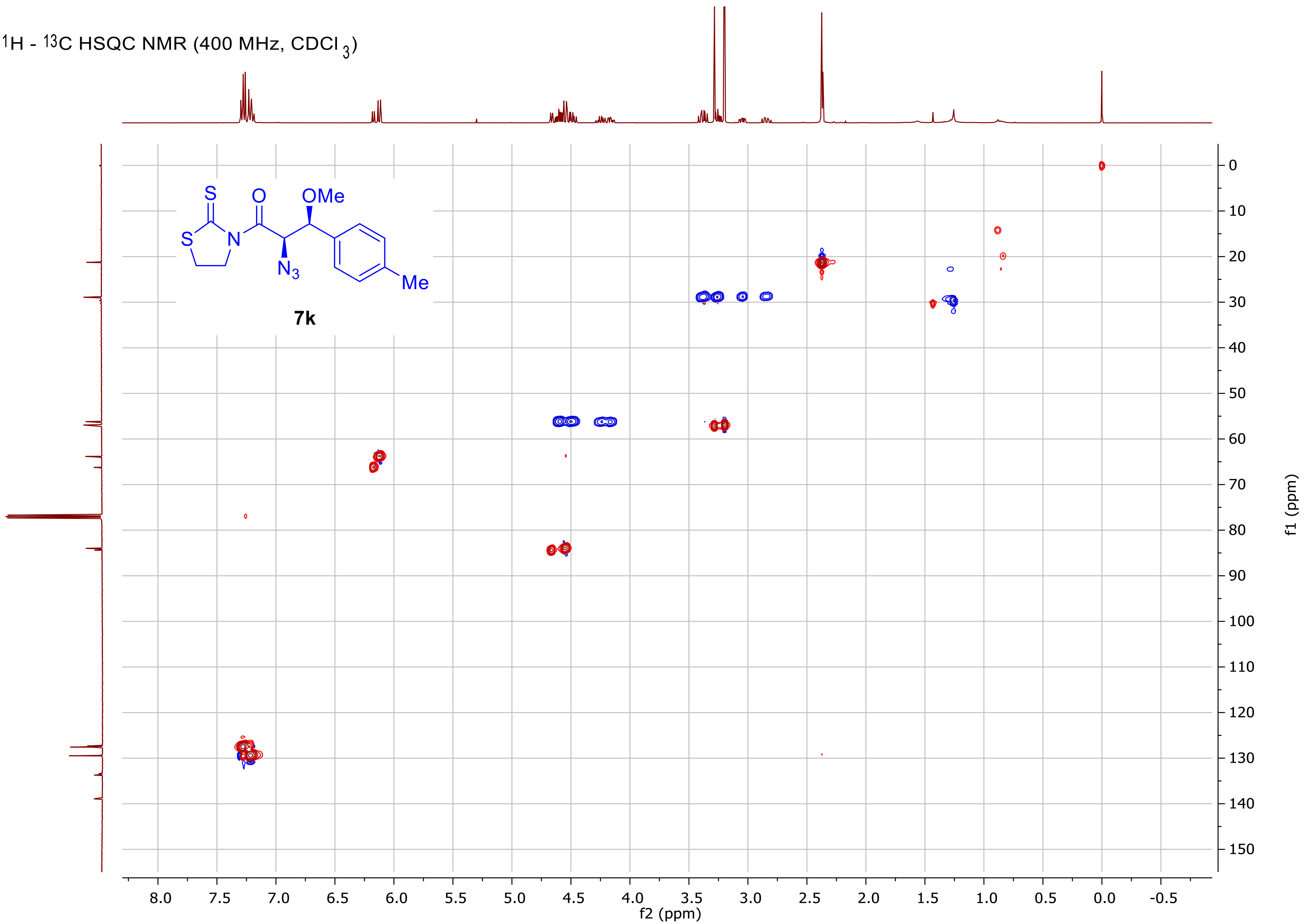

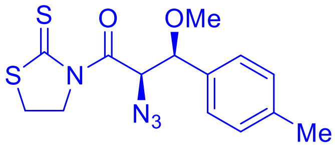

7k

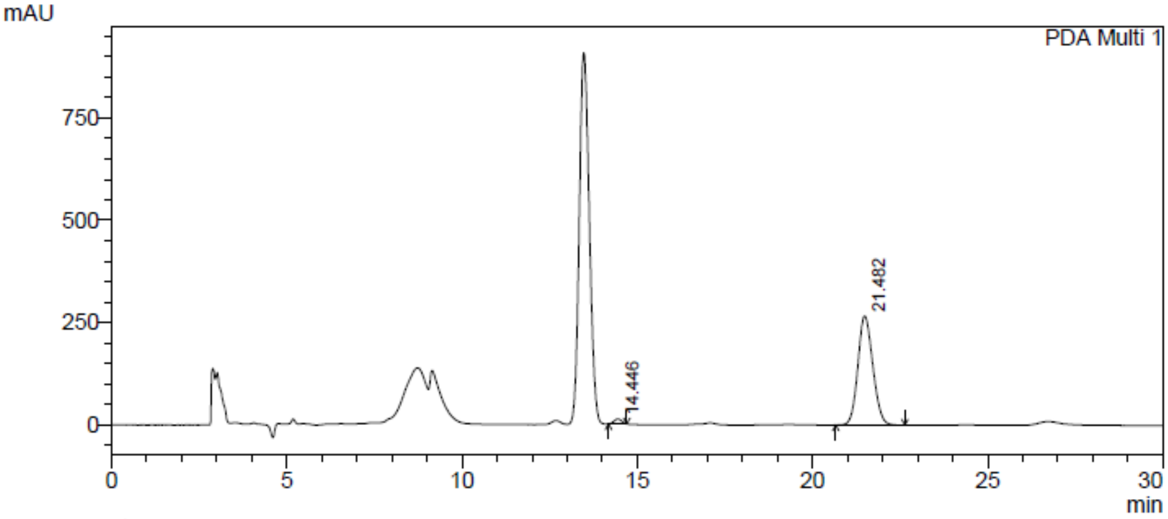

1 PDA Multi 1/254nm 4nm

PeakTable

PDA Ch1 254nm 4nm

Peak#	Ret. Time	Area	Height	Area %	Height %
1	14.446	184874	12015	2.288	4.311
2	21.482	7894608	266677	97.712	95.689
Total		8079482	278692	100.000	100.000

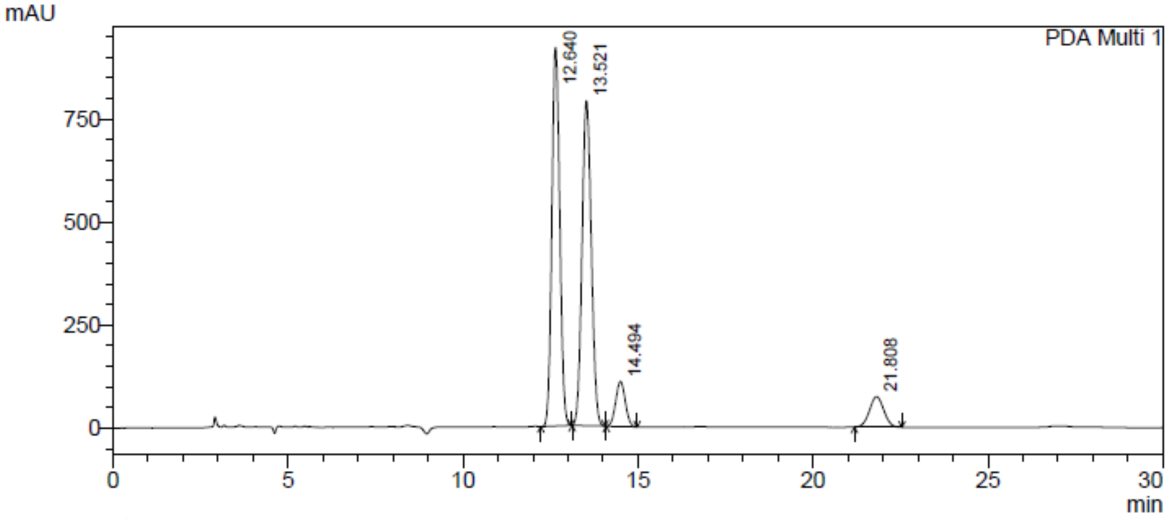

1 PDA Multi 1/254nm 4nm

PeakTable

PDA Ch1 254nm 4nm

Peak#	Ret. Time	Area	Height	Area %	Height %
1	12.640	14400160	918583	43.453	48.592
2	13.521	14540649	788304	43.877	41.701
3	14.494	2086360	109354	6.296	5.785
4	21.808	2112766	74150	6.375	3.922
Total		33139935	1890393	100.000	100.000

^{1}H NMR (400 MHz, $CDCl_3$)

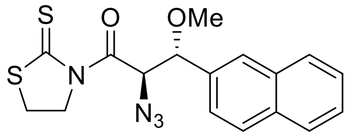

5l

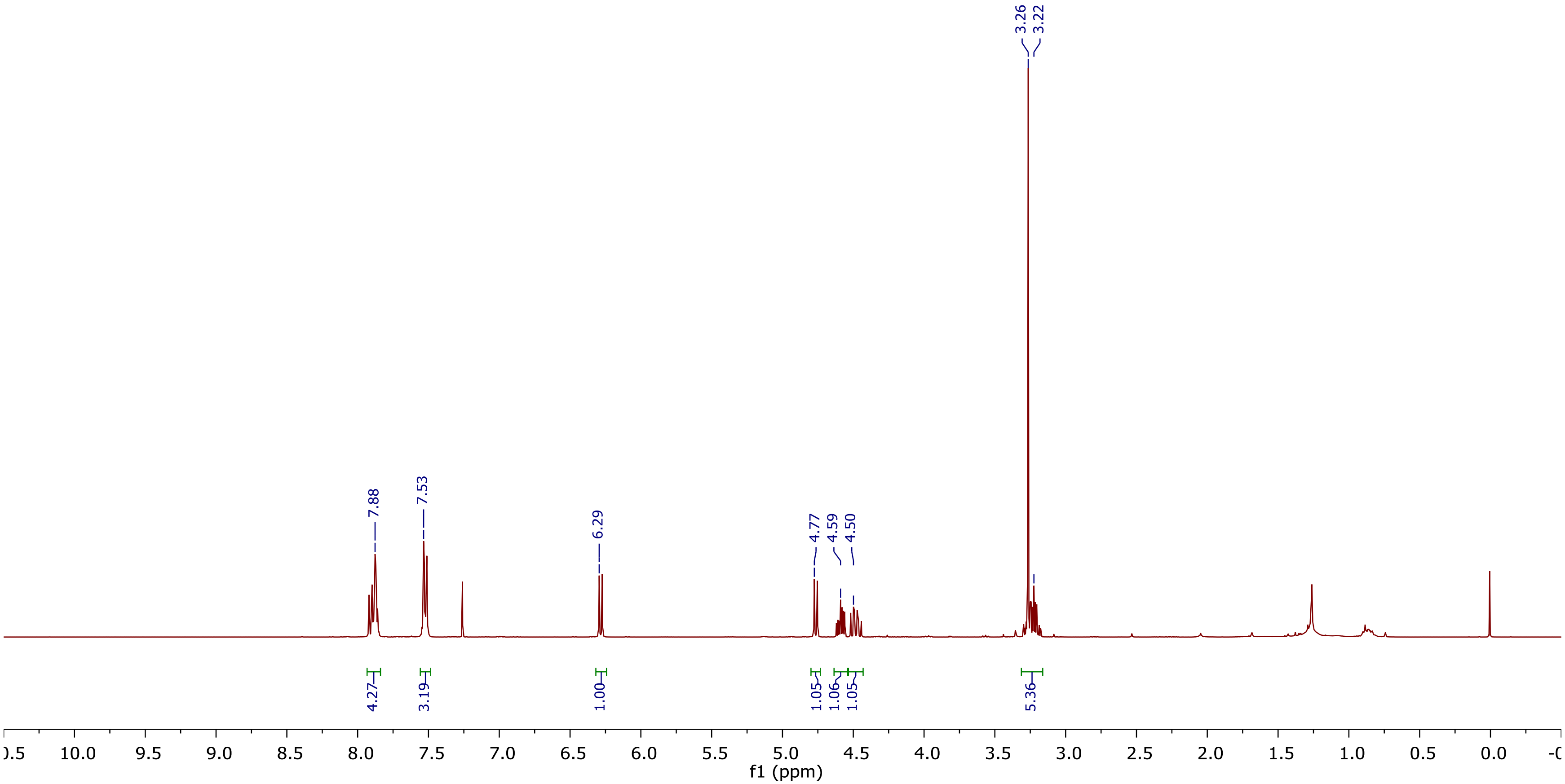

$^{13}C\{^1H\}$ NMR (100.6 MHz, $CDCl_3$)

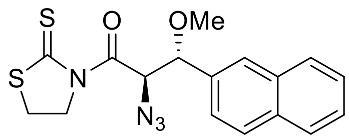

5l

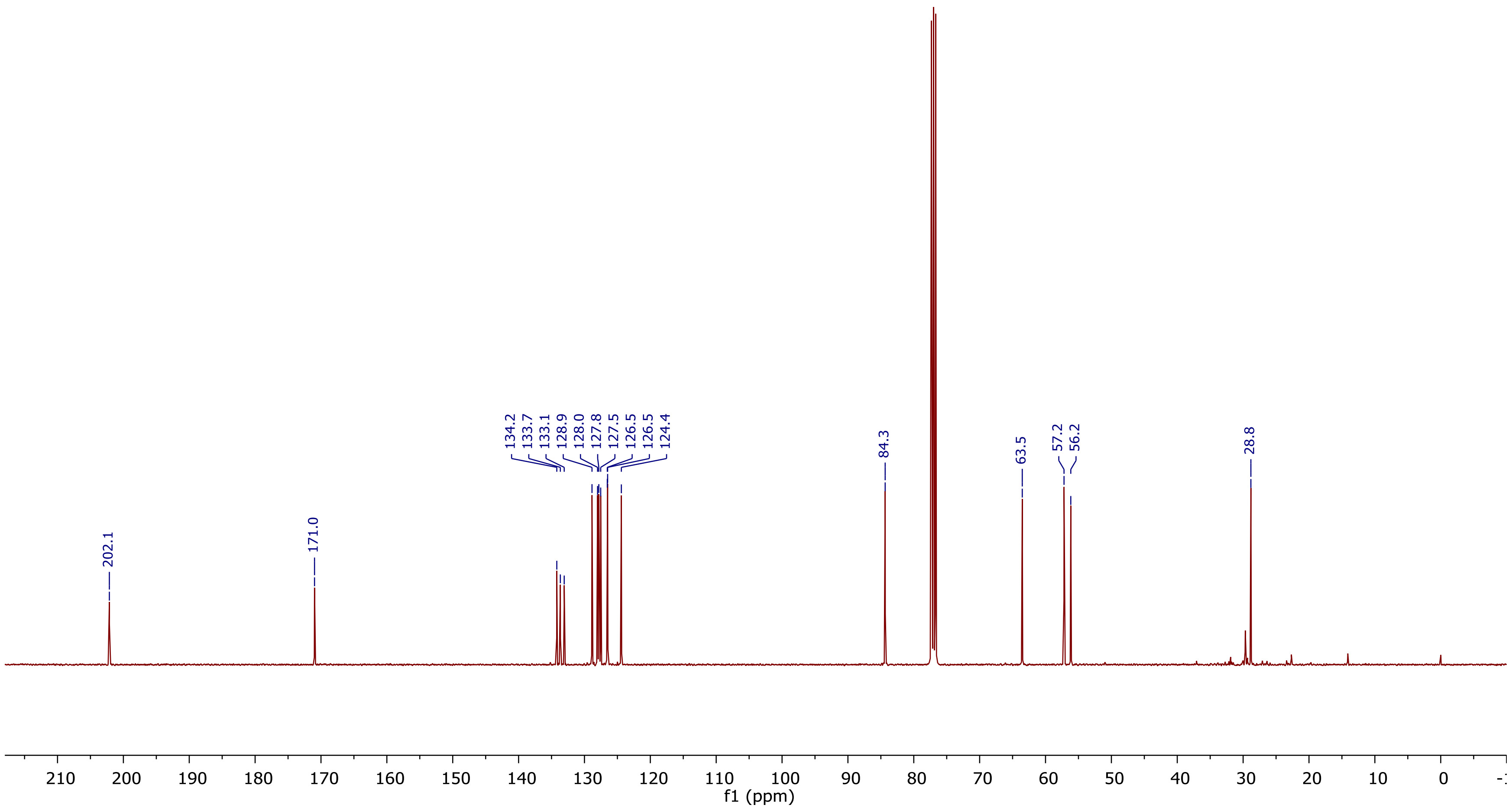

^1H - ^1H COSY NMR (400 MHz, $CDCl_3$)

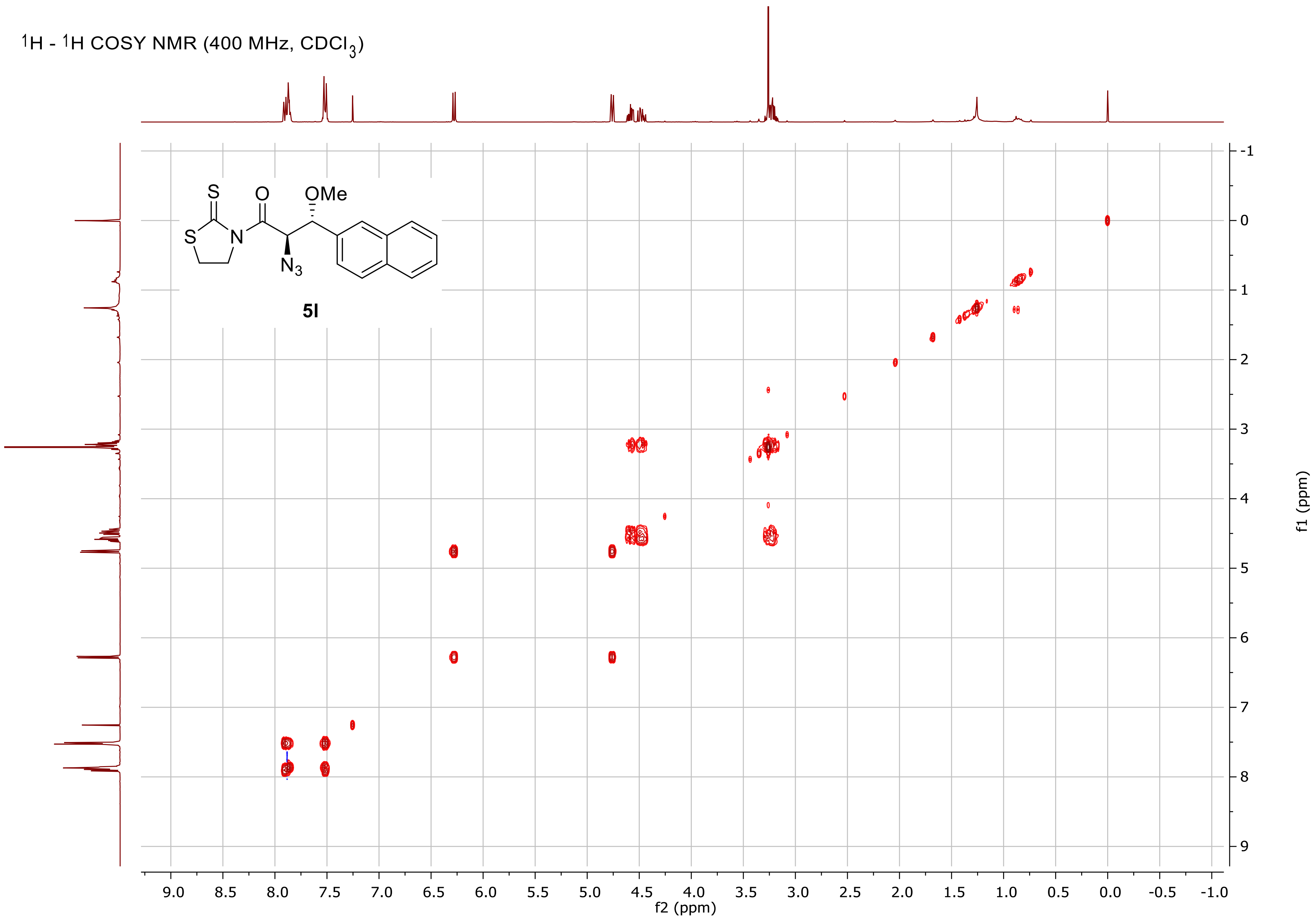

^1H - ^{13}C HSQC NMR (400 MHz, $CDCl_3$)

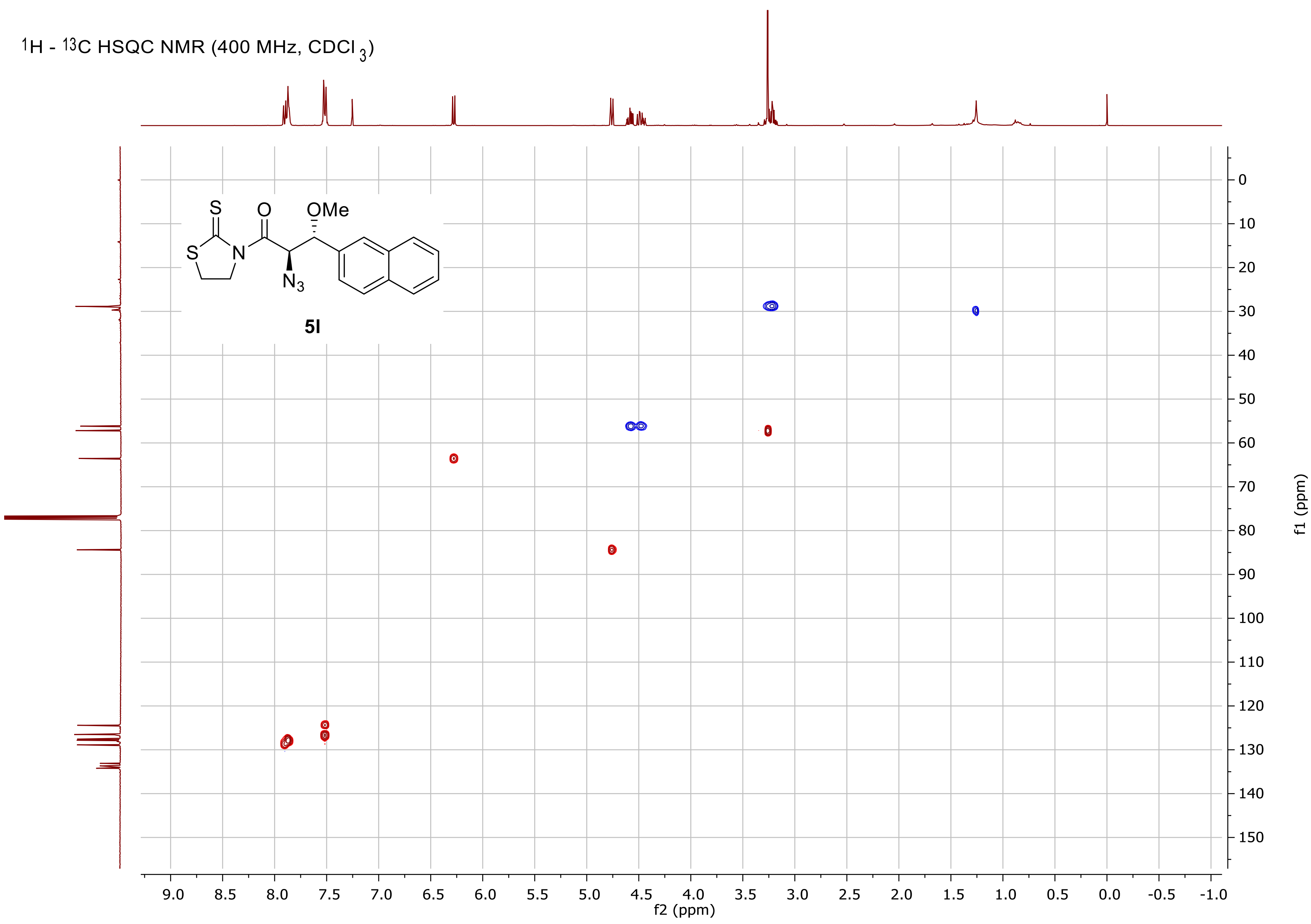

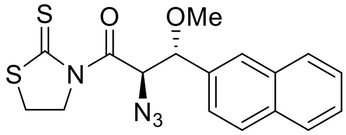

5l

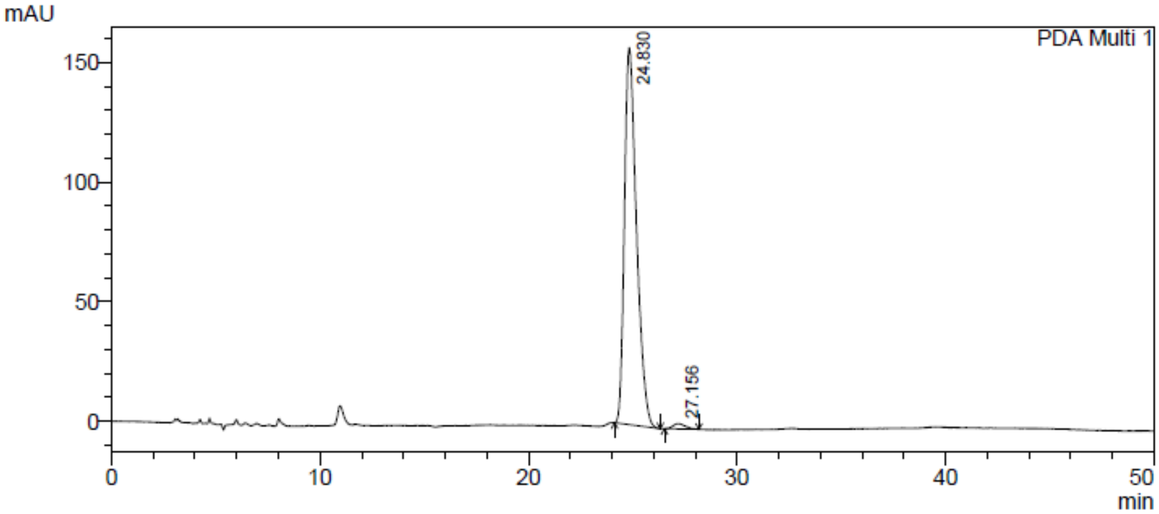

1 PDA Multi 1/254nm 4nm

PeakTable

PDA Ch1 254nm 4nm

Peak#	Ret. Time	Area	Height	Area %	Height %
1	24.830	6327216	157467	98.541	98.563
2	27.156	93709	2296	1.459	1.437
Total		6420925	159764	100.000	100.000

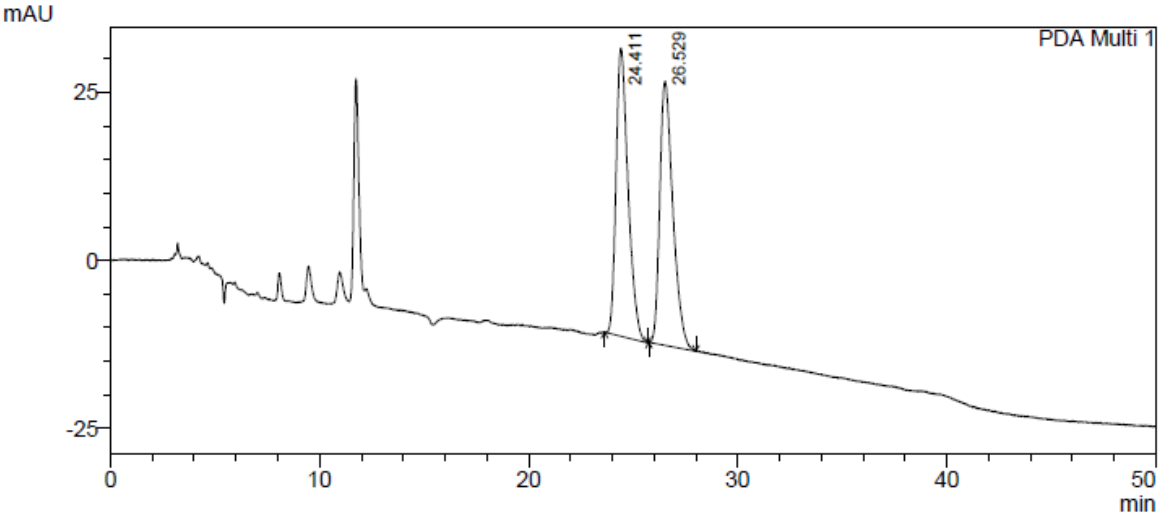

1 PDA Multi 1/254nm 4nm

PeakTable

PDA Ch1 254nm 4nm

Peak#	Ret. Time	Area	Height	Area %	Height %
1	24.411	1707698	42850	49.946	52.150
2	26.529	1711392	39317	50.054	47.850
Total		3419089	82168	100.000	100.000

^1H NMR (400 MHz, $CDCl_3$)

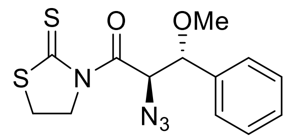

5m

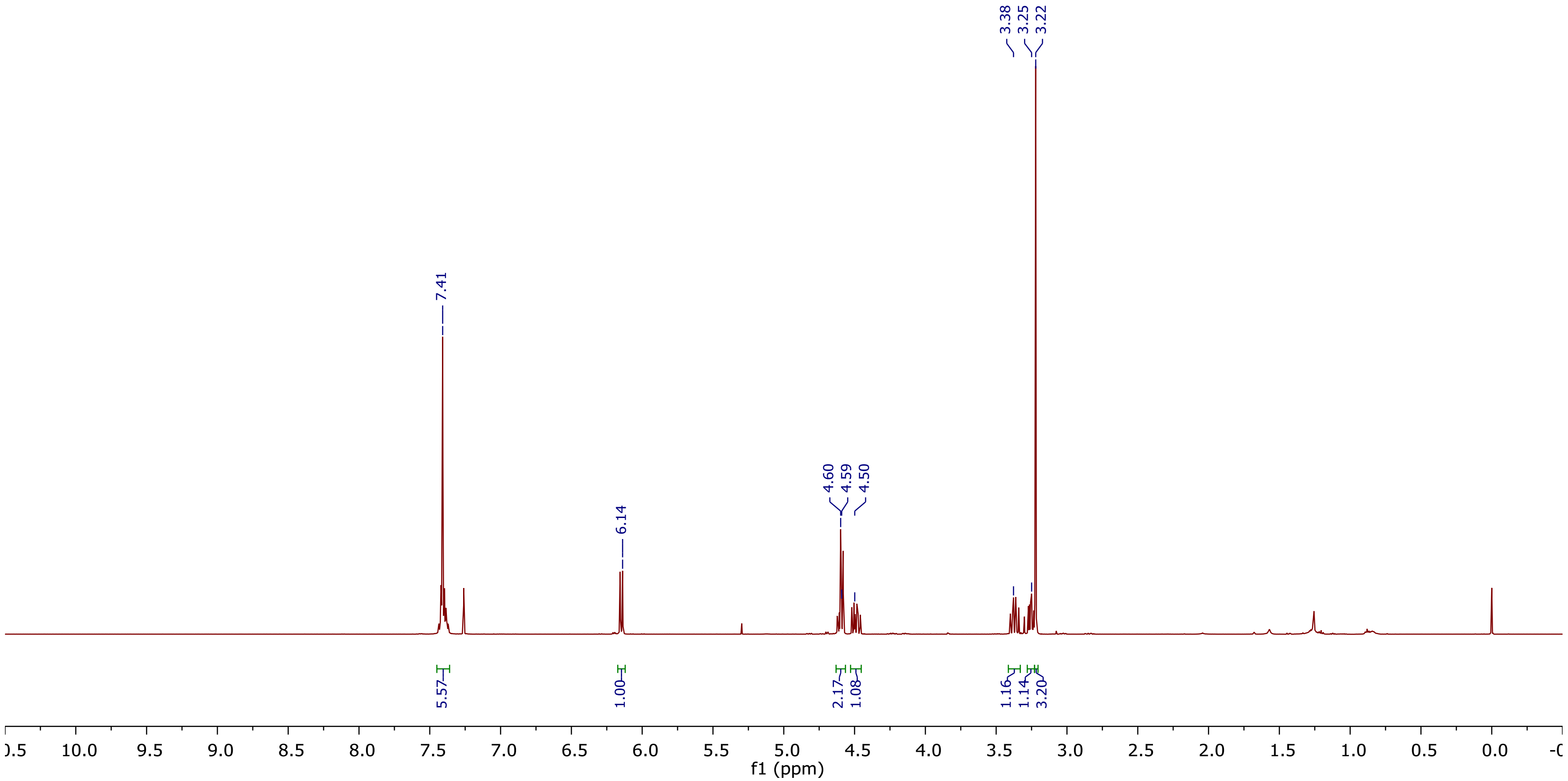

$^{13}C\{^{1}H\}$ NMR (100.6 MHz, $CDCl_3$)

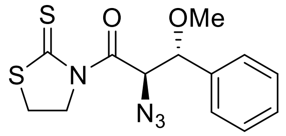

5m

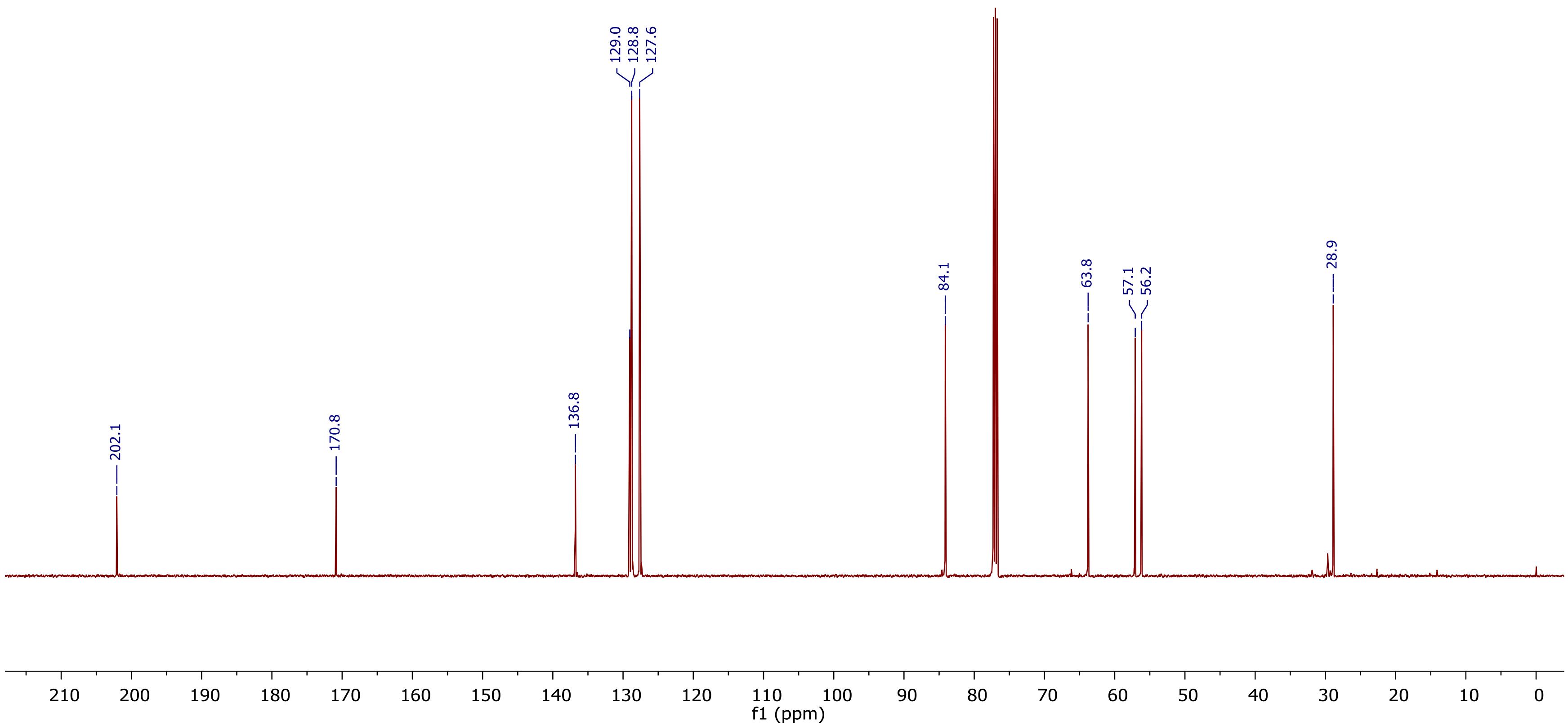

^1H - ^1H COSY NMR (400 MHz, $CDCl_3$)

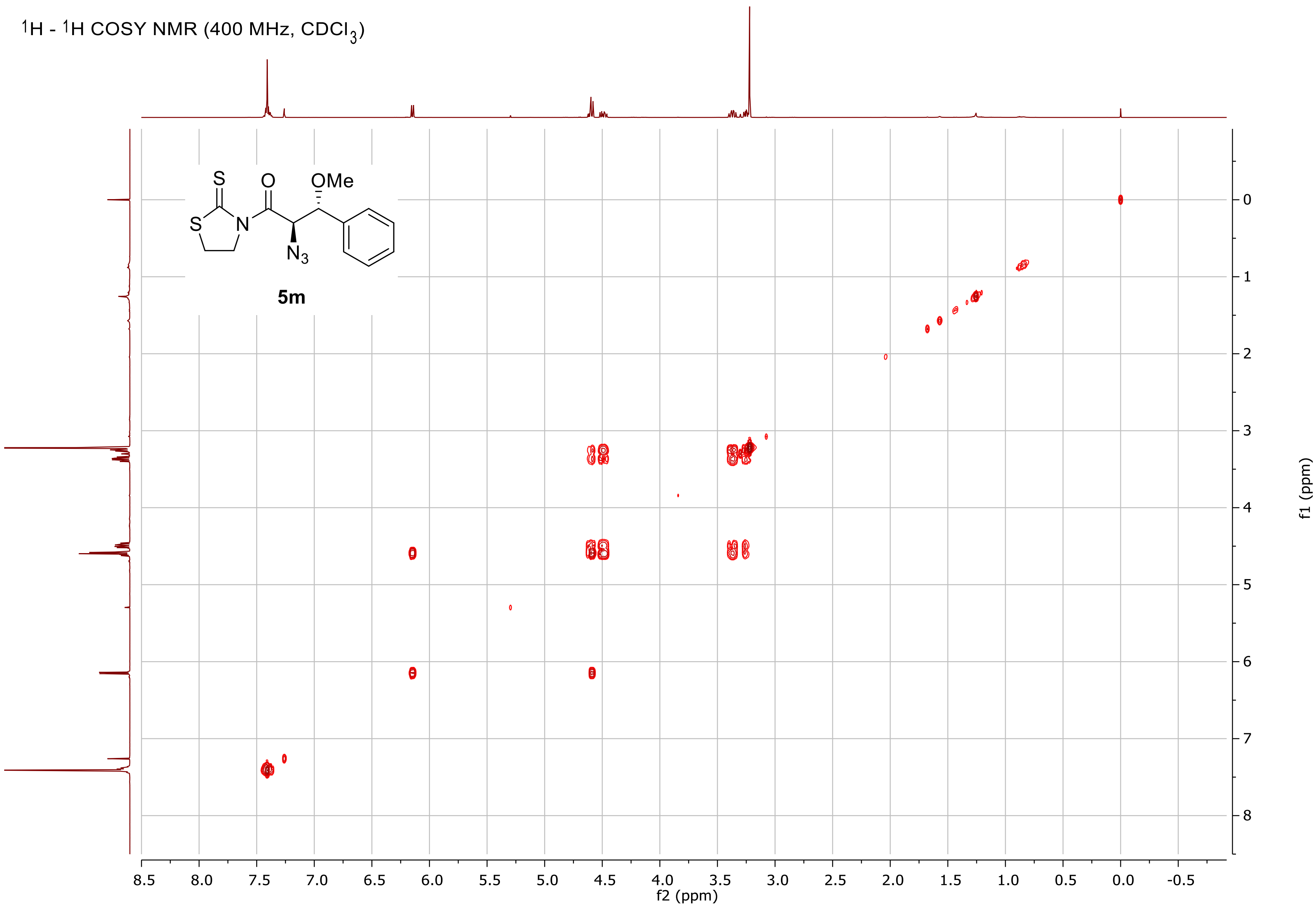

^{1}H - ^{13}C HSQC NMR (400 MHz, $CDCl_3$)

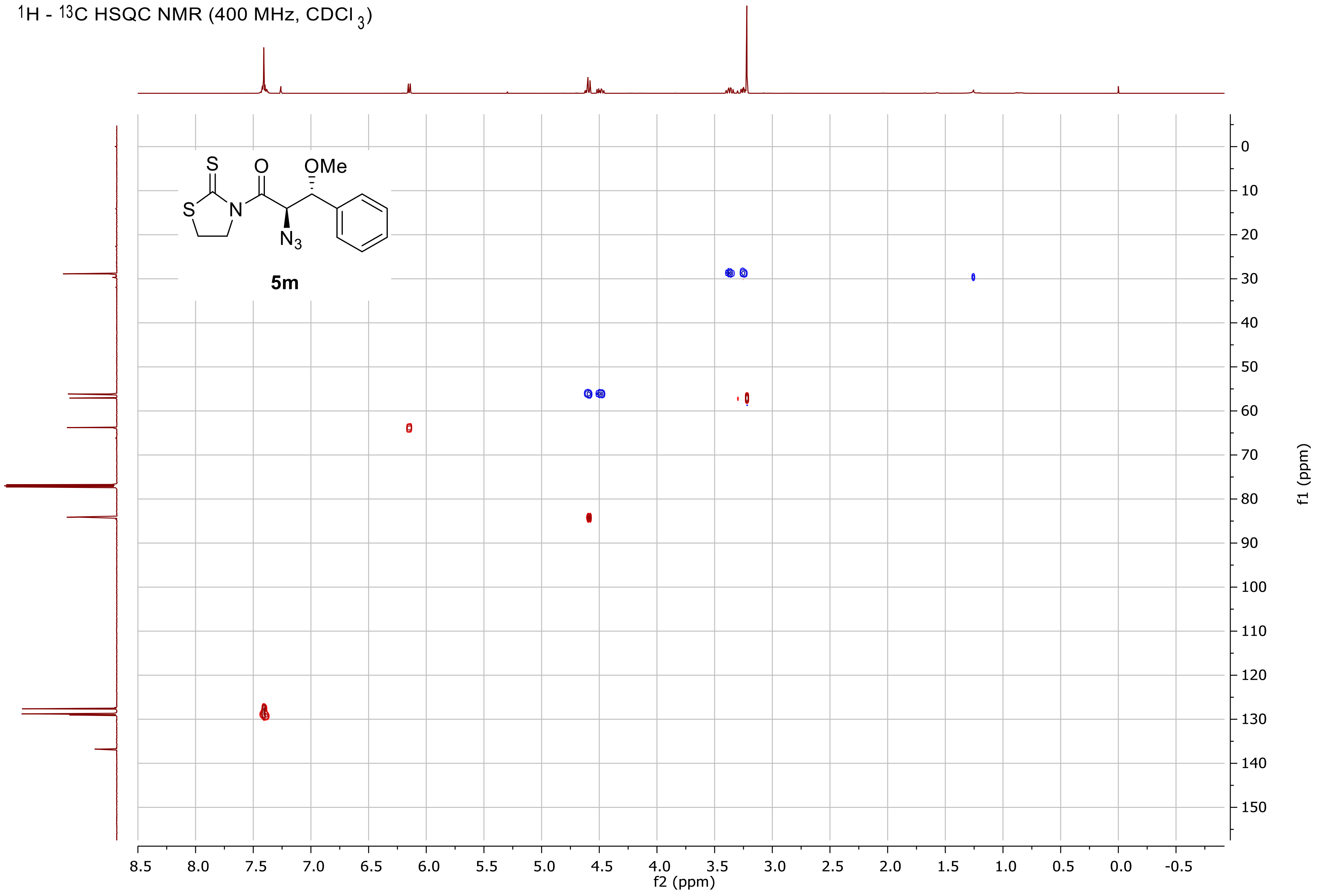

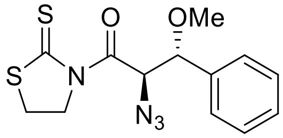

5m

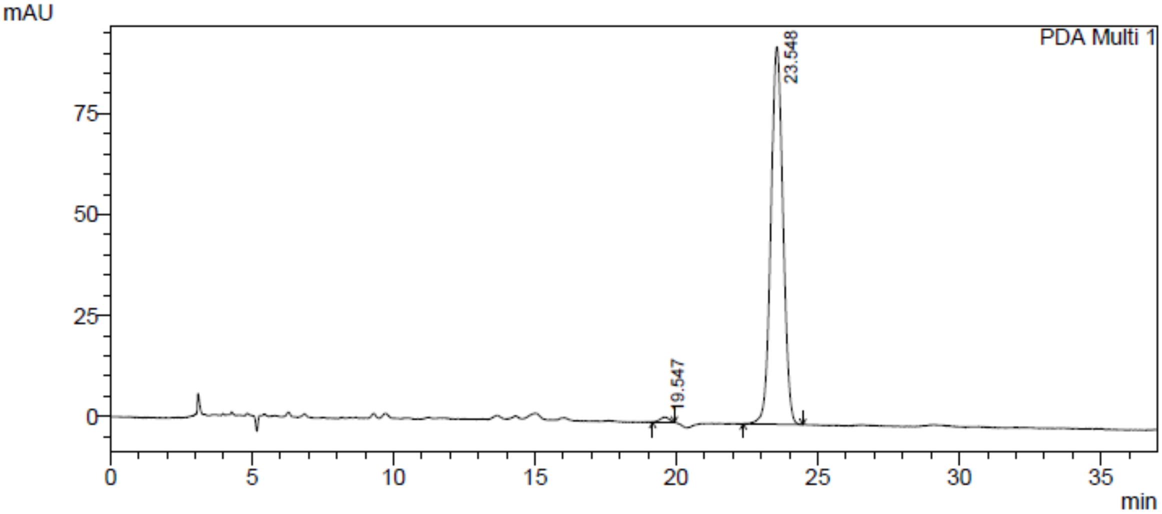

1 PDA Multi 1/254nm 4nm

PeakTable

PDA Ch1 254nm 4nm

Peak#	Ret. Time	Area	Height	Area %	Height %
1	19.547	29730	1278	1.059	1.350
2	23.548	2776836	93438	98.941	98.650
Total		2806567	94716	100.000	100.000

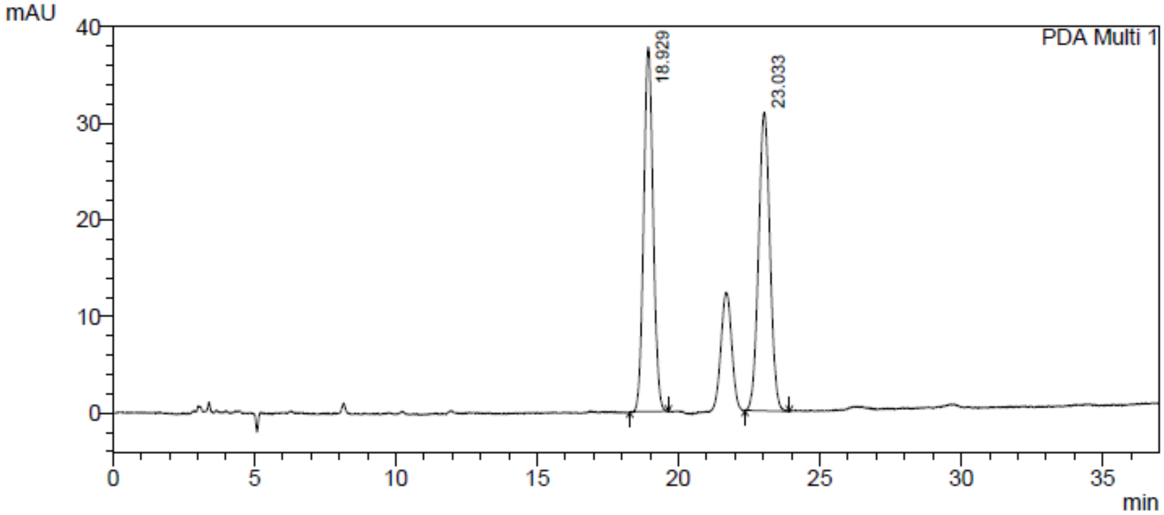

1 PDA Multi 1/254nm 4nm

PeakTable

PDA Ch1 254nm 4nm

Peak#	Ret. Time	Area	Height	Area %	Height %
1	18.929	881053	37771	50.105	54.990
2	23.033	877350	30916	49.895	45.010
Total		1758403	68687	100.000	100.000

^1H NMR (400 MHz, $CDCl_3$)

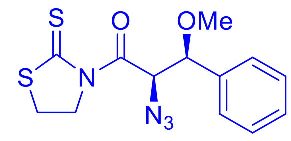

7m

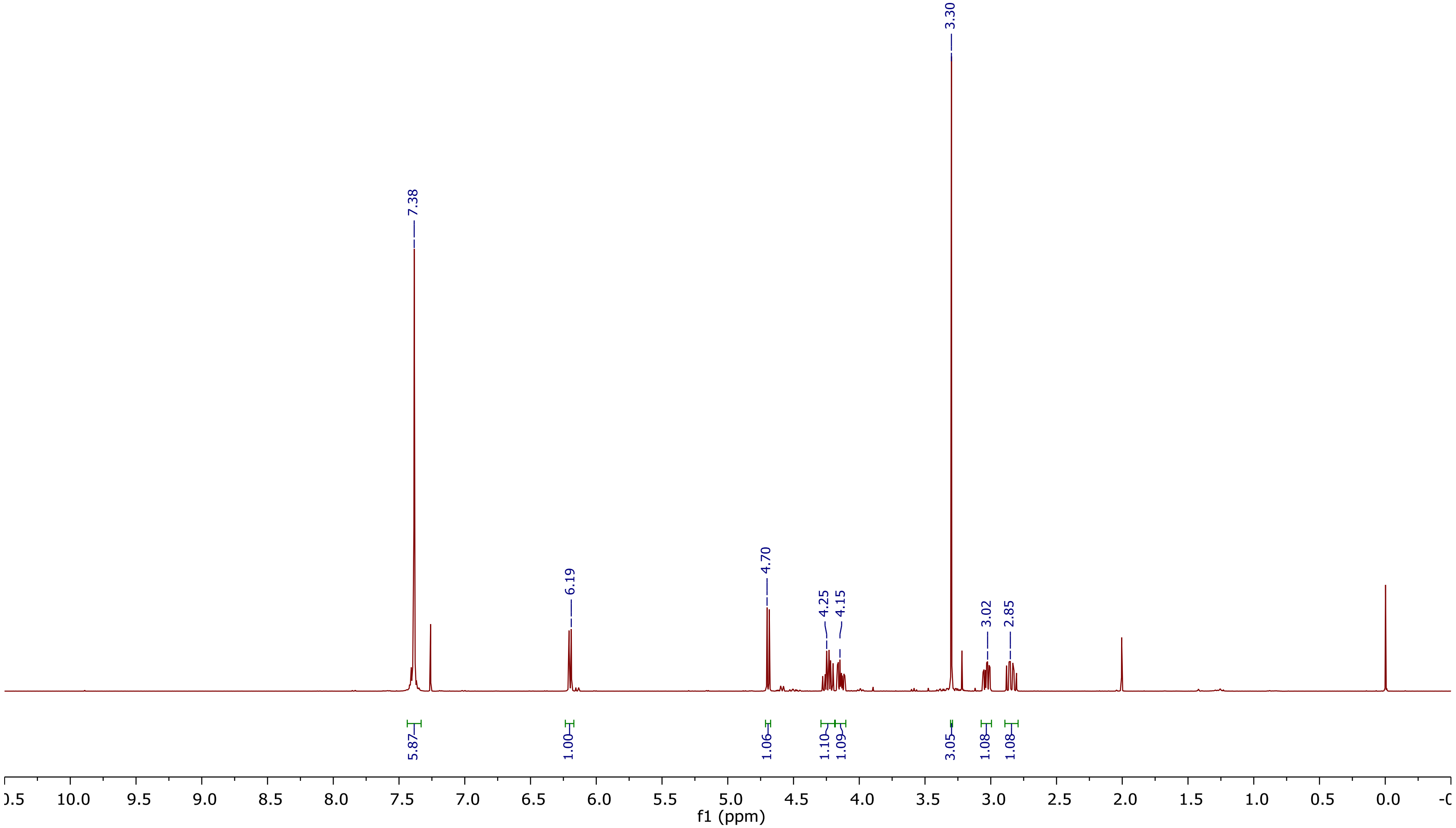

$^{13}C\{^1H\}$ NMR (100.6 MHz, $CDCl_3$)

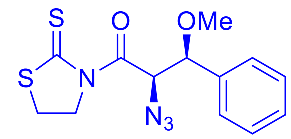

7m

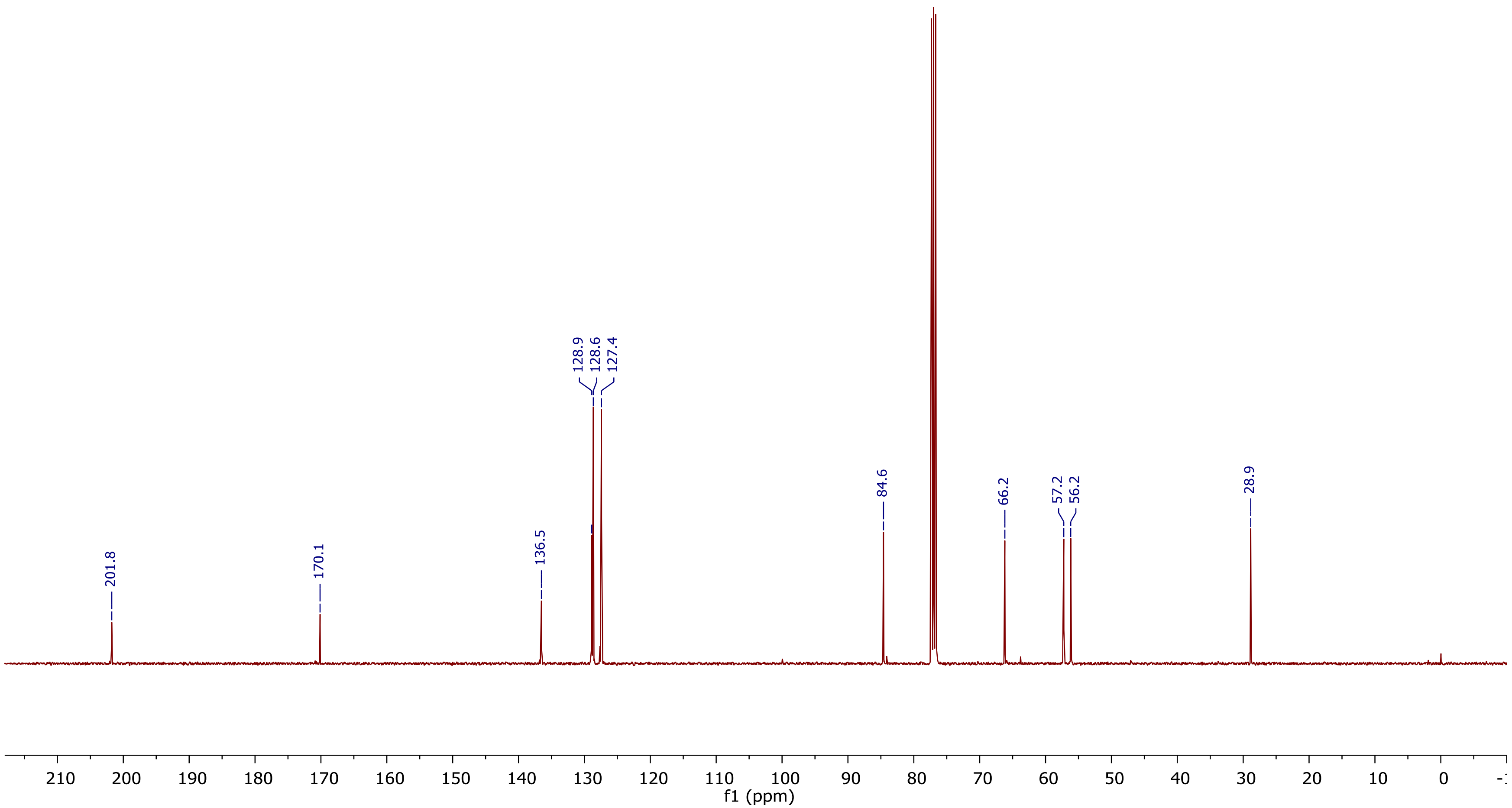

^1H - ^1H COSY NMR (400 MHz, $CDCl_3$)

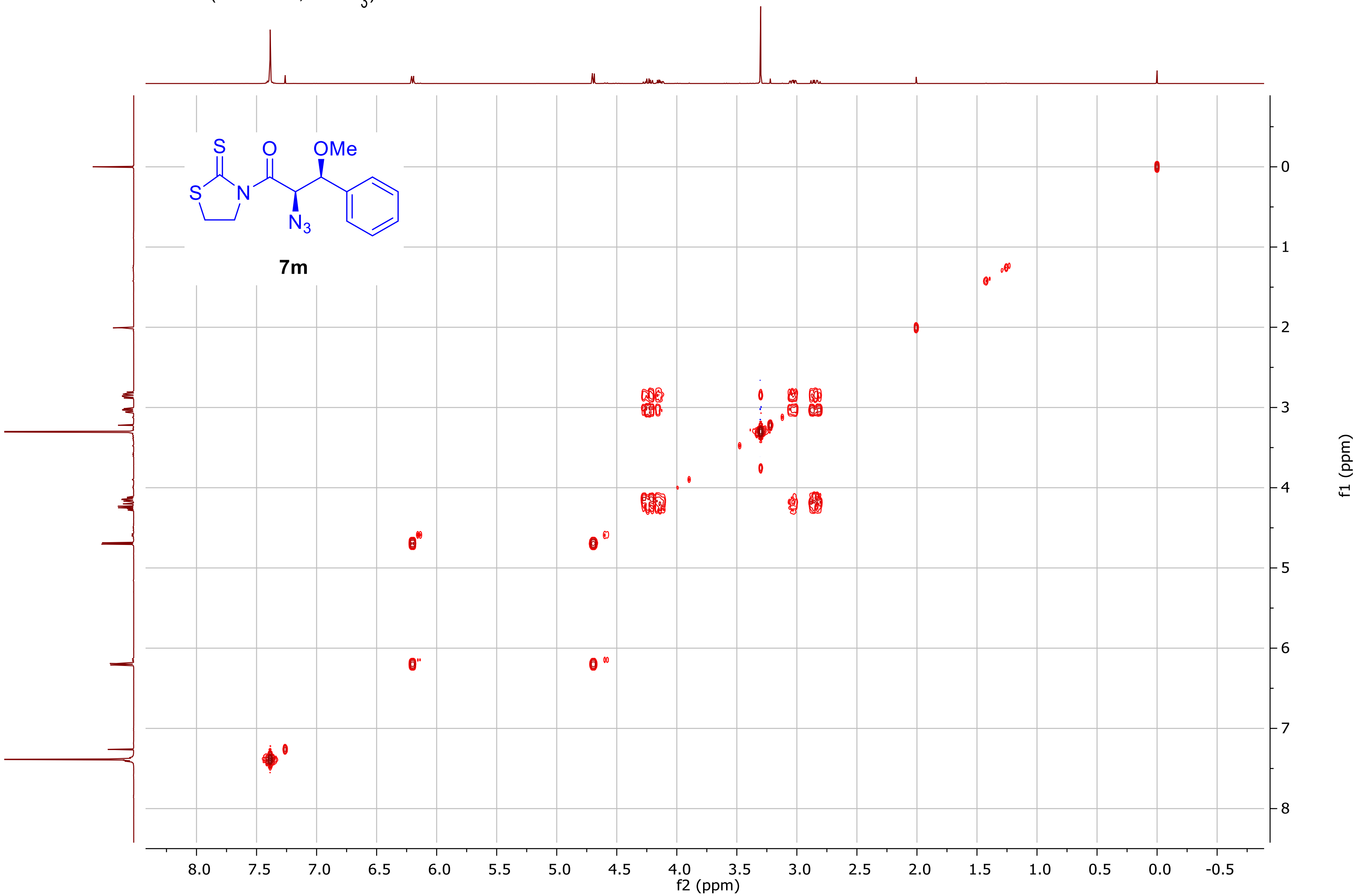

^{1}H - ^{13}C HSQC NMR (400 MHz, $CDCl_3$)

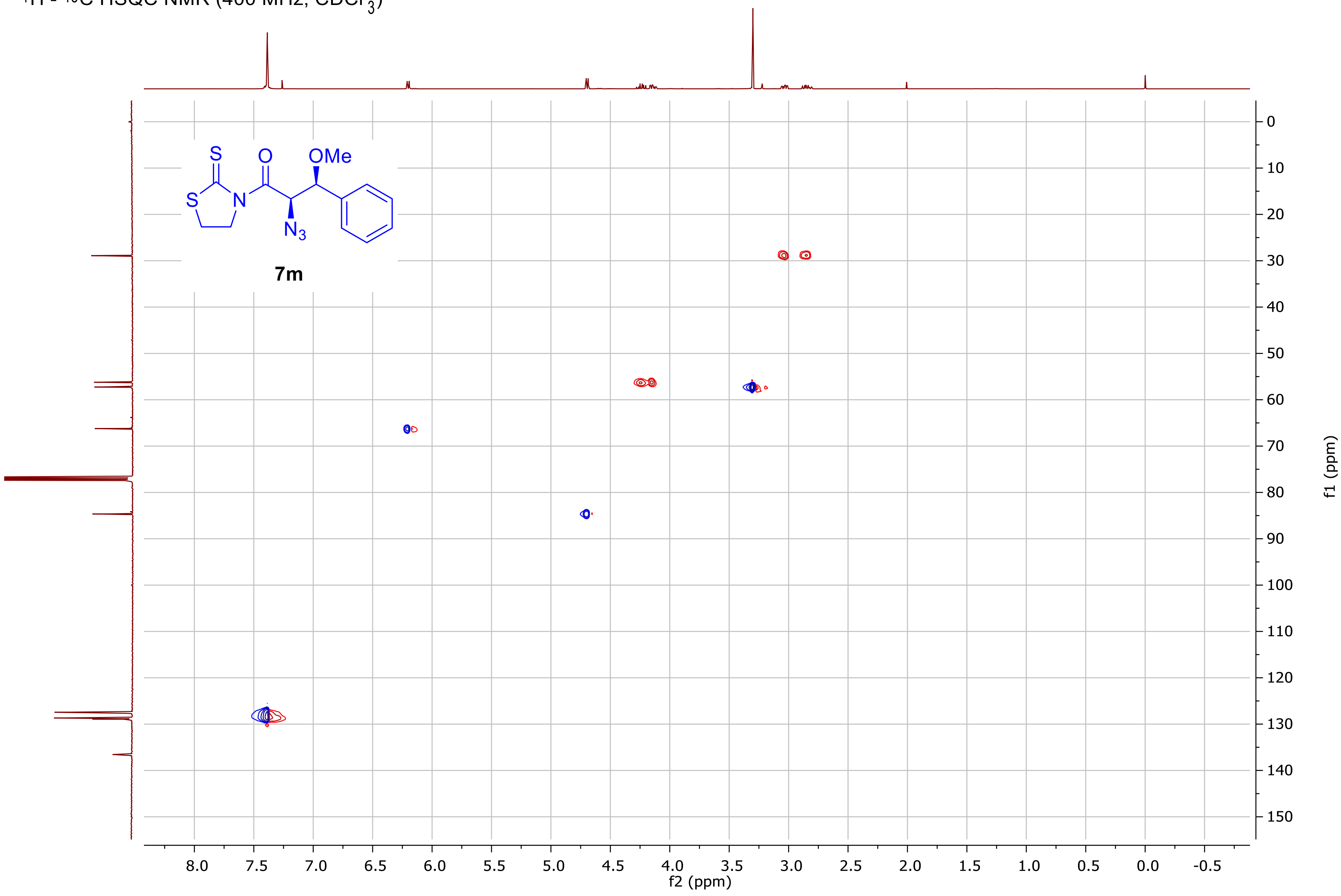

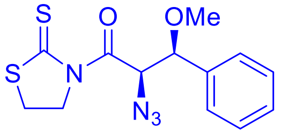

7m

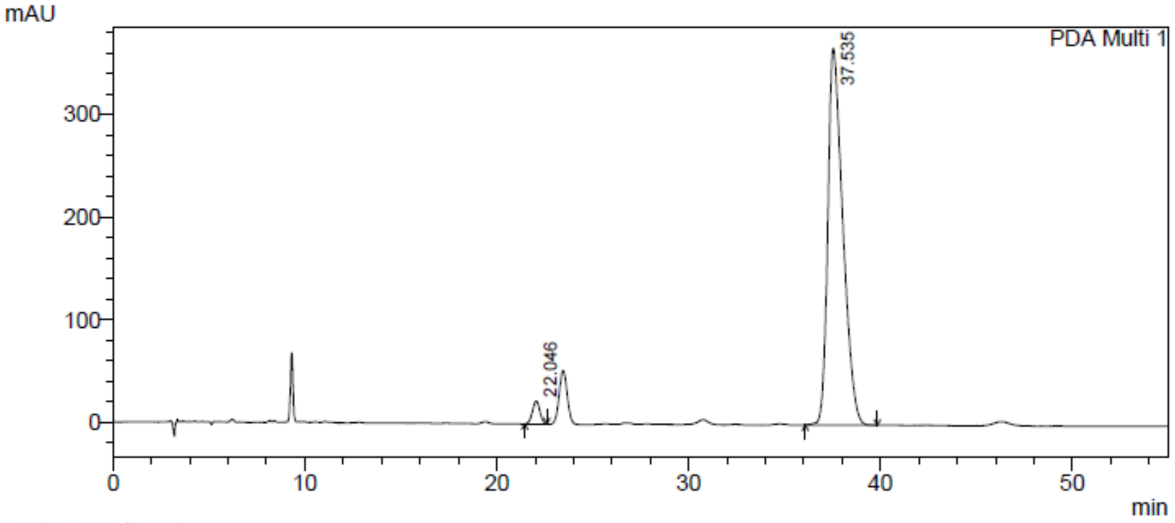

1 PDA Multi 1/254nm 4nm

PeakTable

PDA Ch1 254nm 4nm

Peak#	Ret. Time	Area	Height	Area %	Height %
1	22.046	597570	22260	2.898	5.707
2	37.535	20020134	367752	97.102	94.293
Total		20617704	390012	100.000	100.000

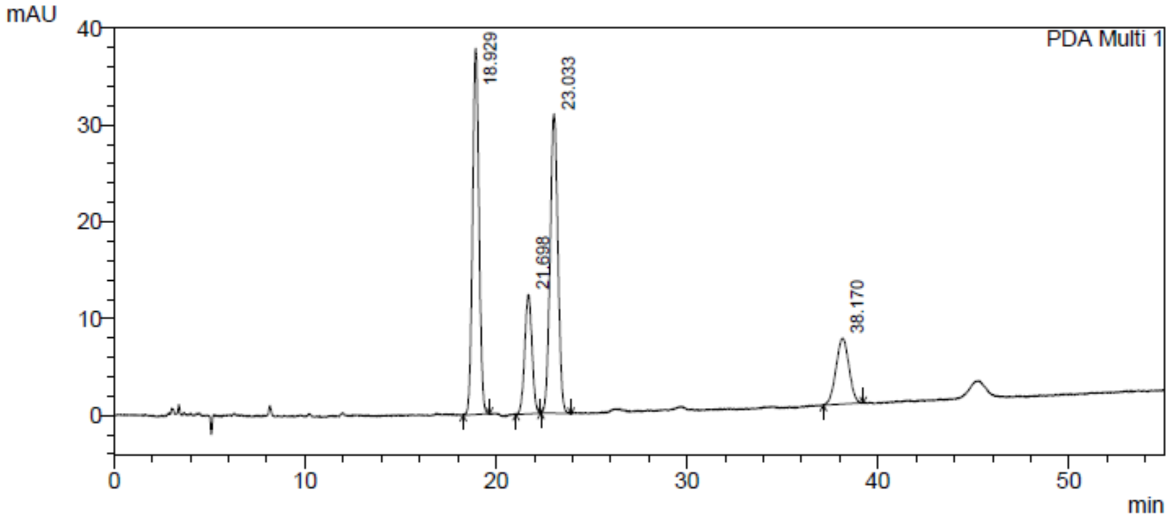

1 PDA Multi 1/254nm 4nm

PeakTable

PDA Ch1 254nm 4nm

Peak#	Ret. Time	Area	Height	Area %	Height %
1	18.929	886661	37841	36.757	43.058
2	21.698	327961	12342	13.596	14.044
3	23.033	878988	30937	36.439	35.202
4	38.170	318597	6764	13.208	7.697
Total		2412207	87885	100.000	100.000

^{1}H NMR (400 MHz, $CDCl_3$)

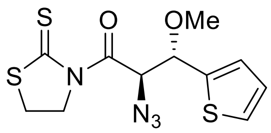

5n

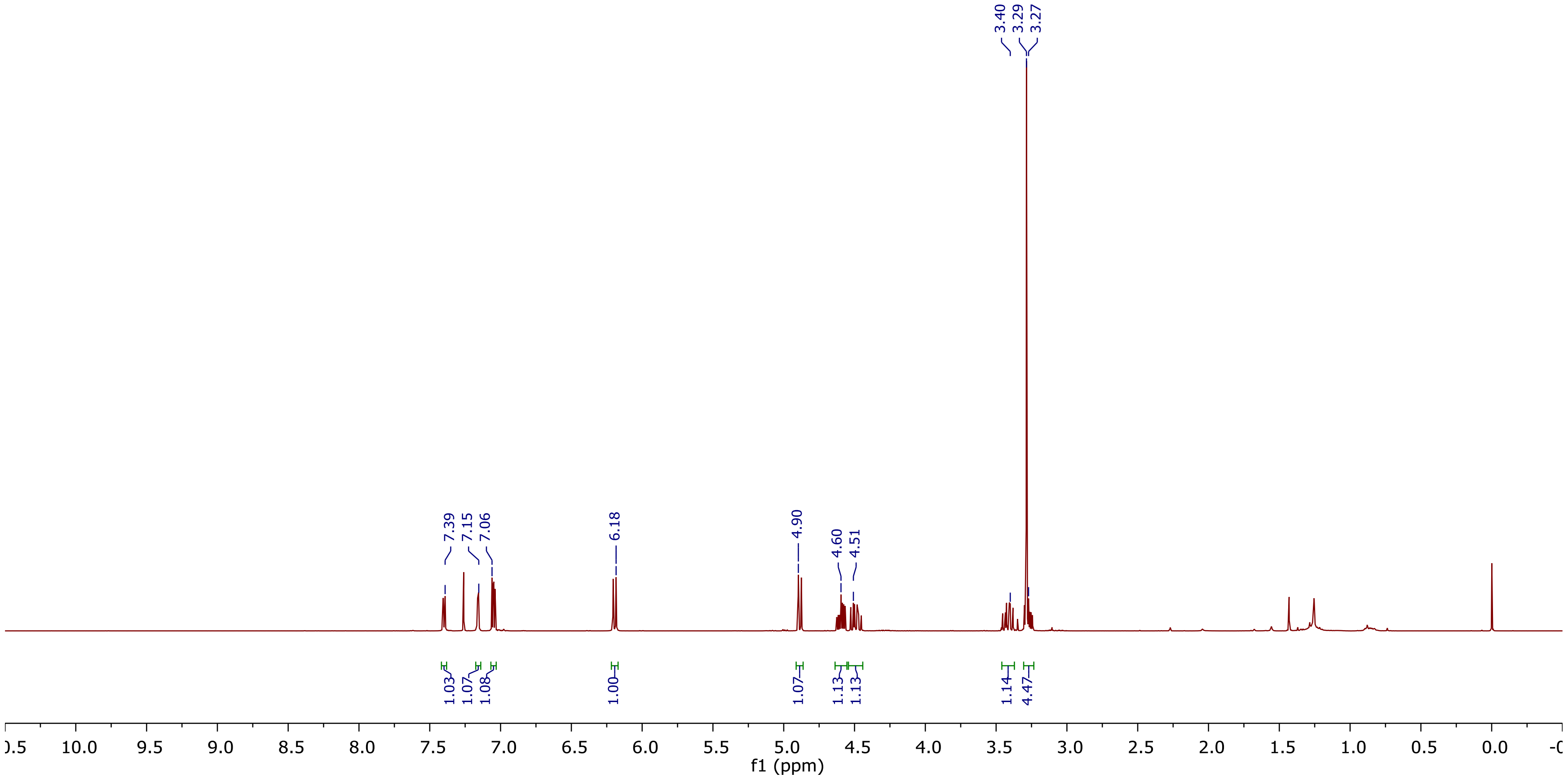

$^{13}C\{^{1}H\}$ NMR (100.6 MHz, $CDCl_3$)

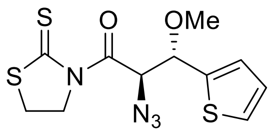

5n

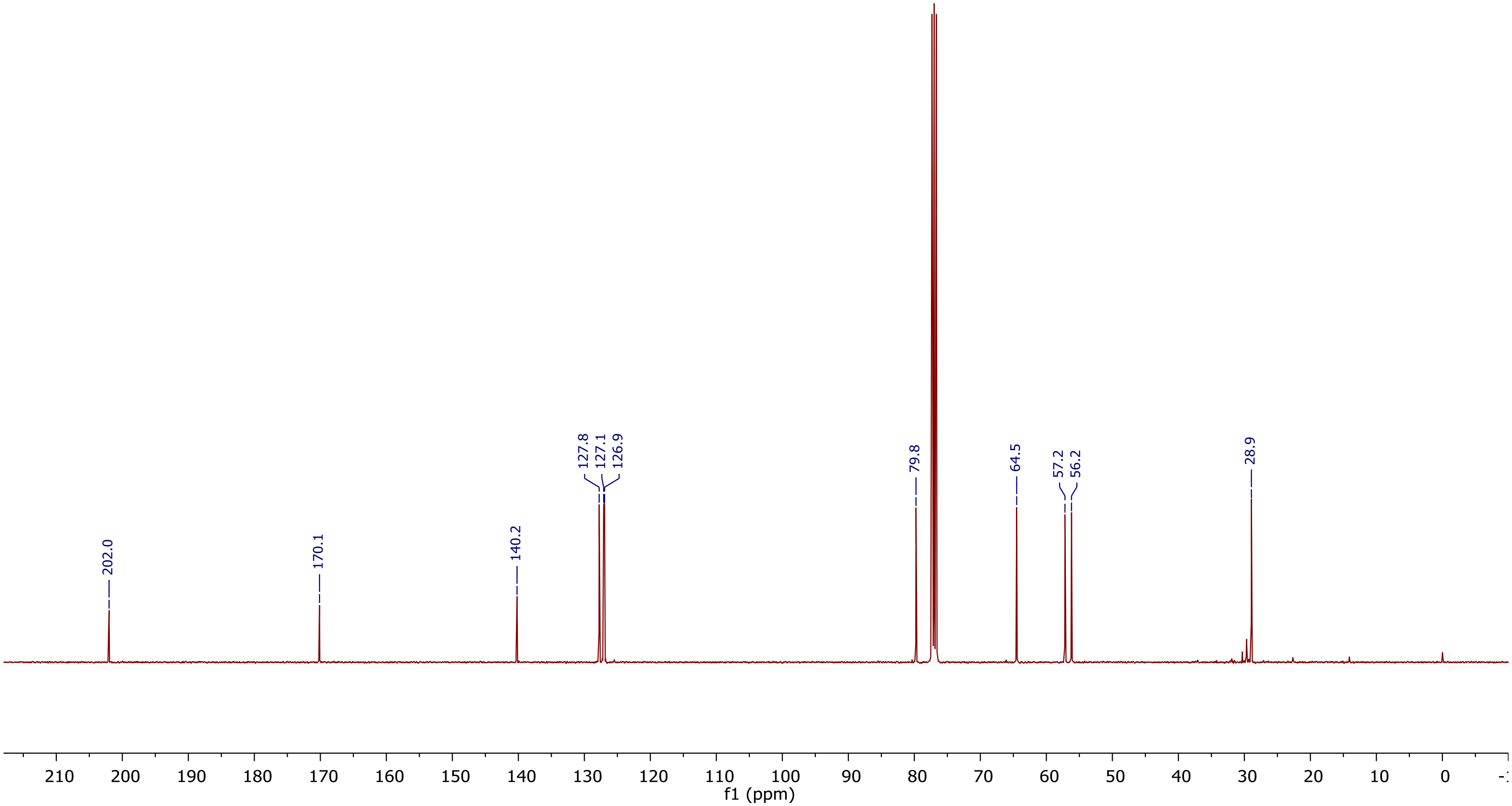

^{1}H - ^{1}H COSY NMR (400 MHz, $CDCl_3$)

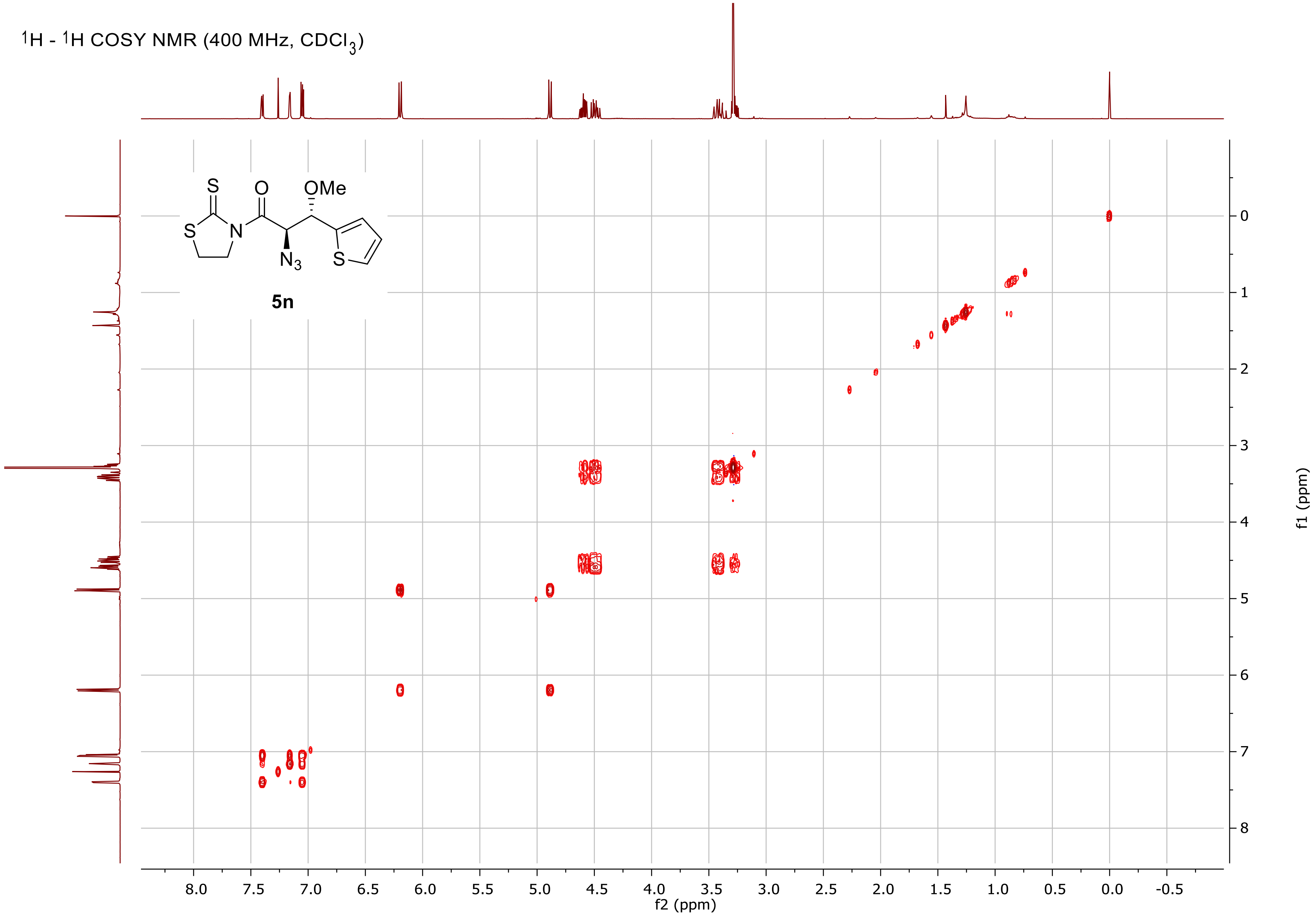

^{1}H - ^{13}C HSQC NMR (400 MHz, $CDCl_3$)

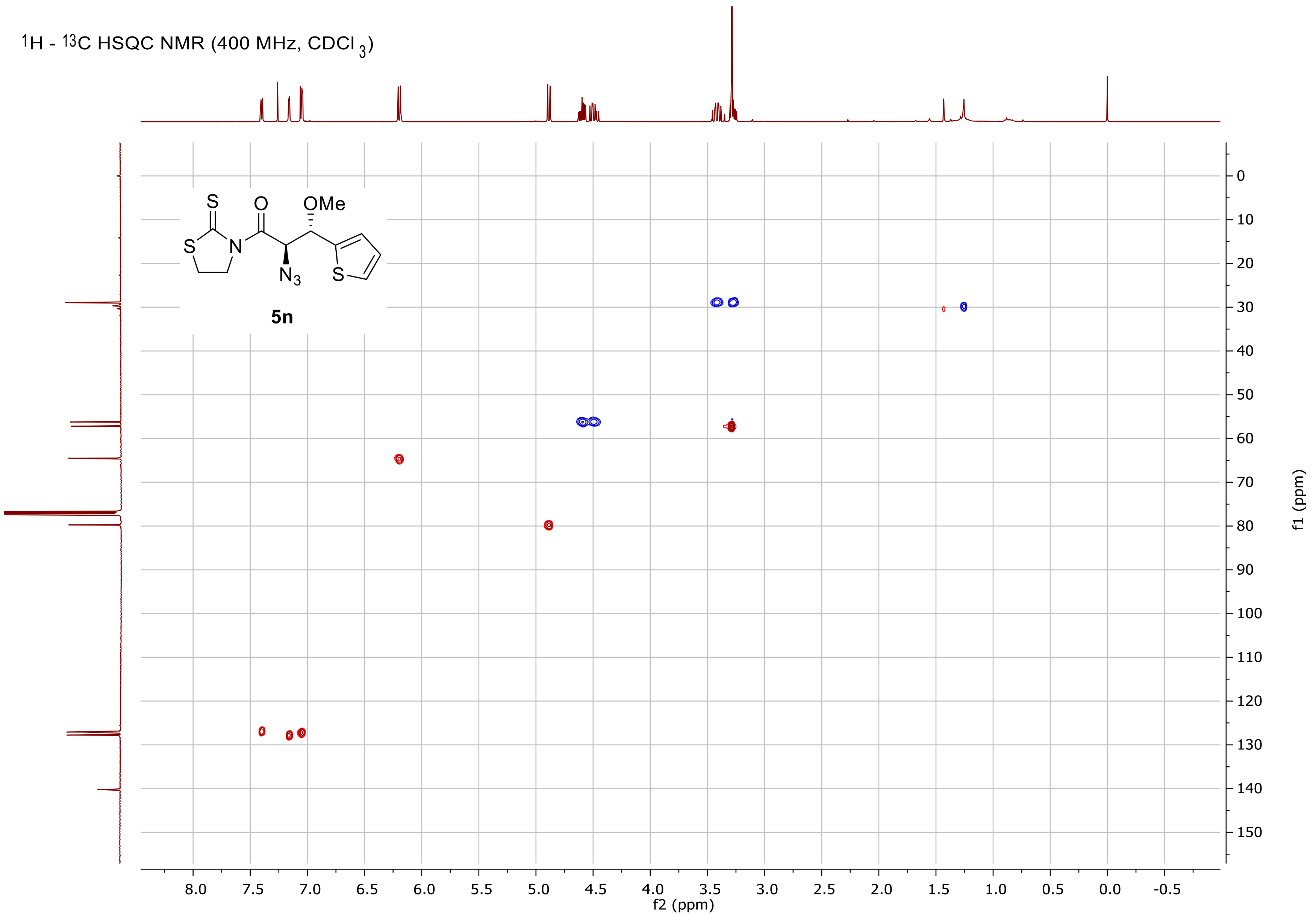

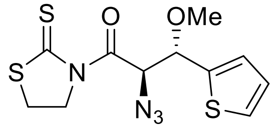

5n

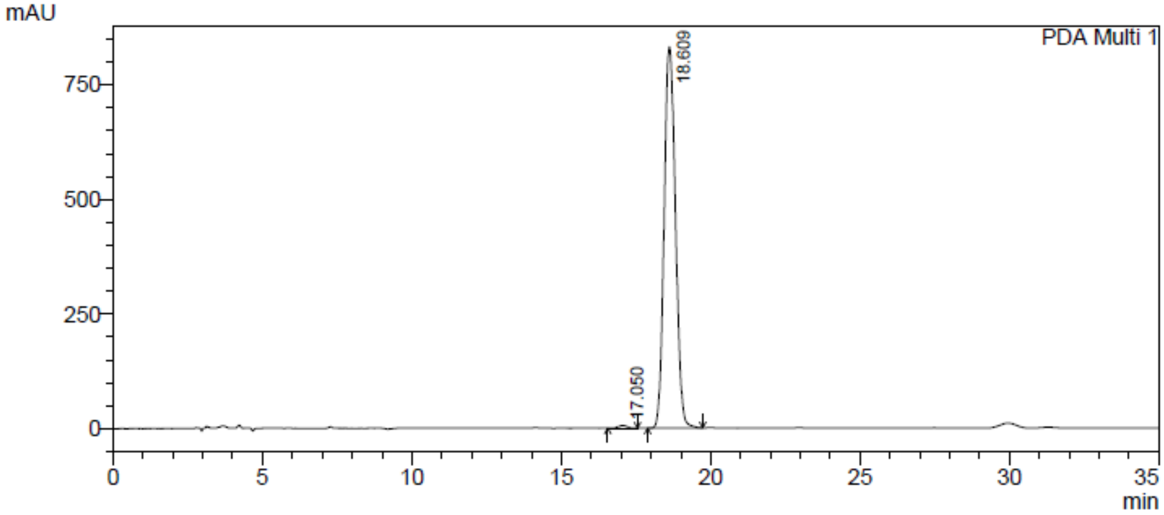

1 PDA Multi 1/254nm 4nm

PeakTable

PDA Ch1 254nm 4nm

Peak#	Ret. Time	Area	Height	Area %	Height %
1	17.050	122878	5810	0.558	0.694
2	18.609	21878728	831850	99.442	99.306
Total		22001605	837660	100.000	100.000

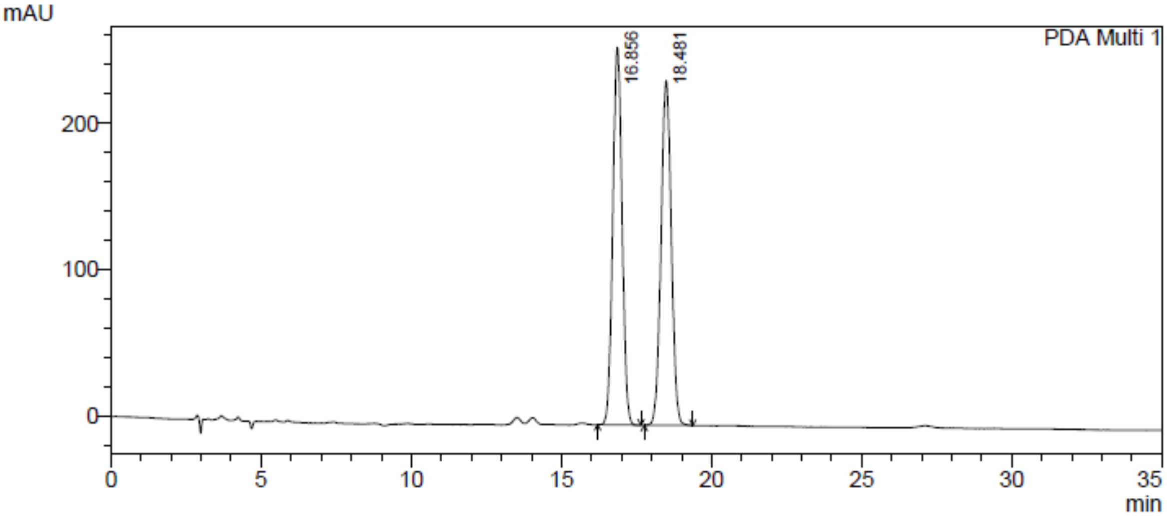

1 PDA Multi 1/254nm 4nm

PeakTable

PDA Ch1 254nm 4nm

Peak#	Ret. Time	Area	Height	Area %	Height %
1	16.856	5548273	257632	49.797	52.274
2	18.481	5593528	235217	50.203	47.726
Total		11141801	492849	100.000	100.000

^1H NMR (400 MHz, $CDCl_3$)

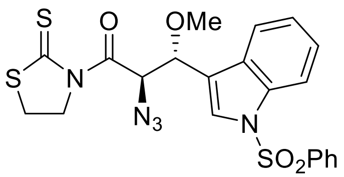

5o

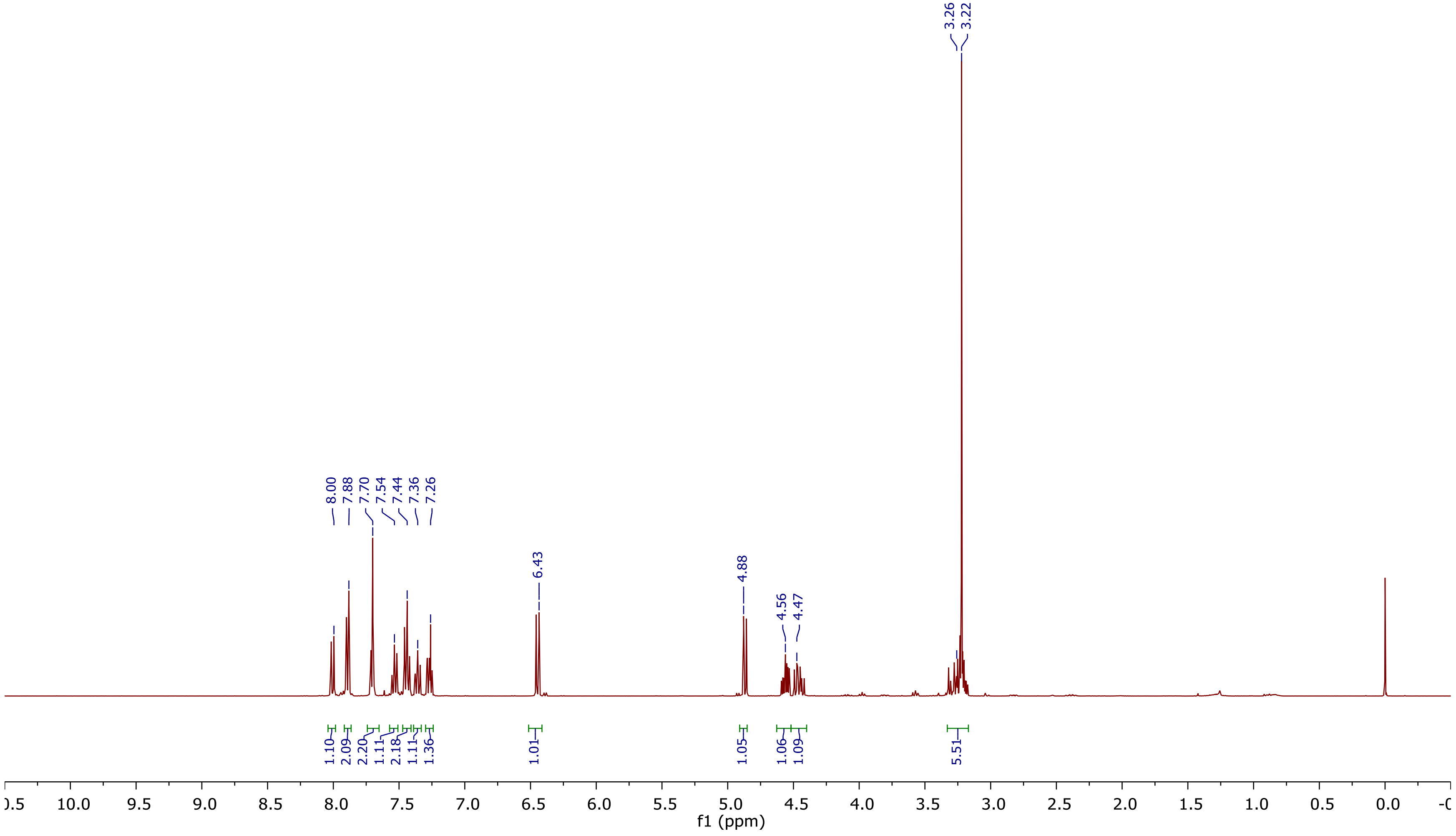

$^{13}C\{^1H\}$ NMR (100.6 MHz, $CDCl_3$)

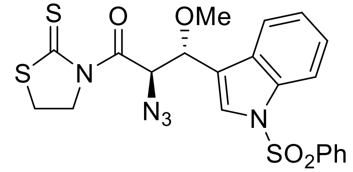

5o

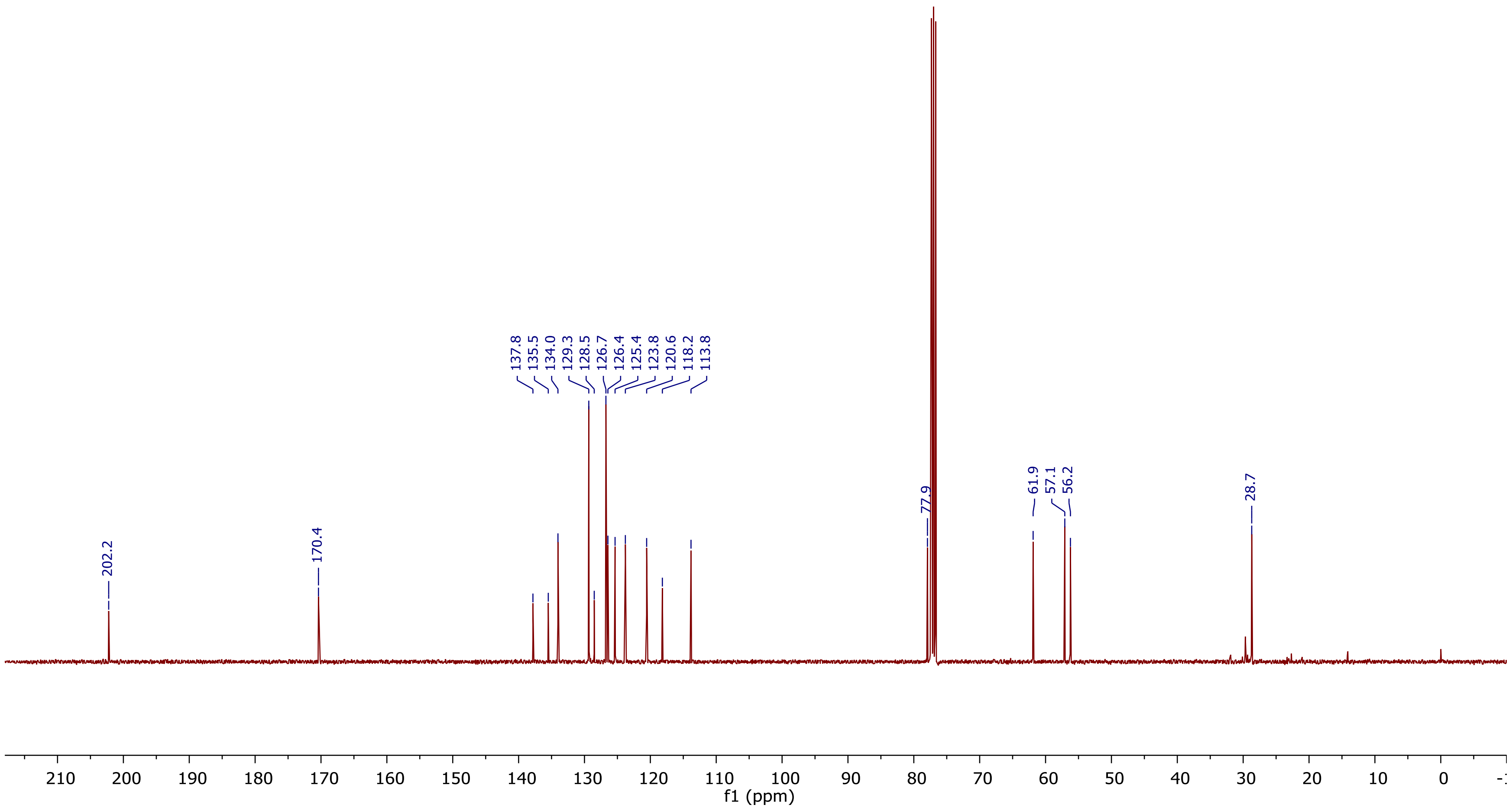

^{1}H - ^{1}H COSY NMR (400 MHz, $CDCl_3$)

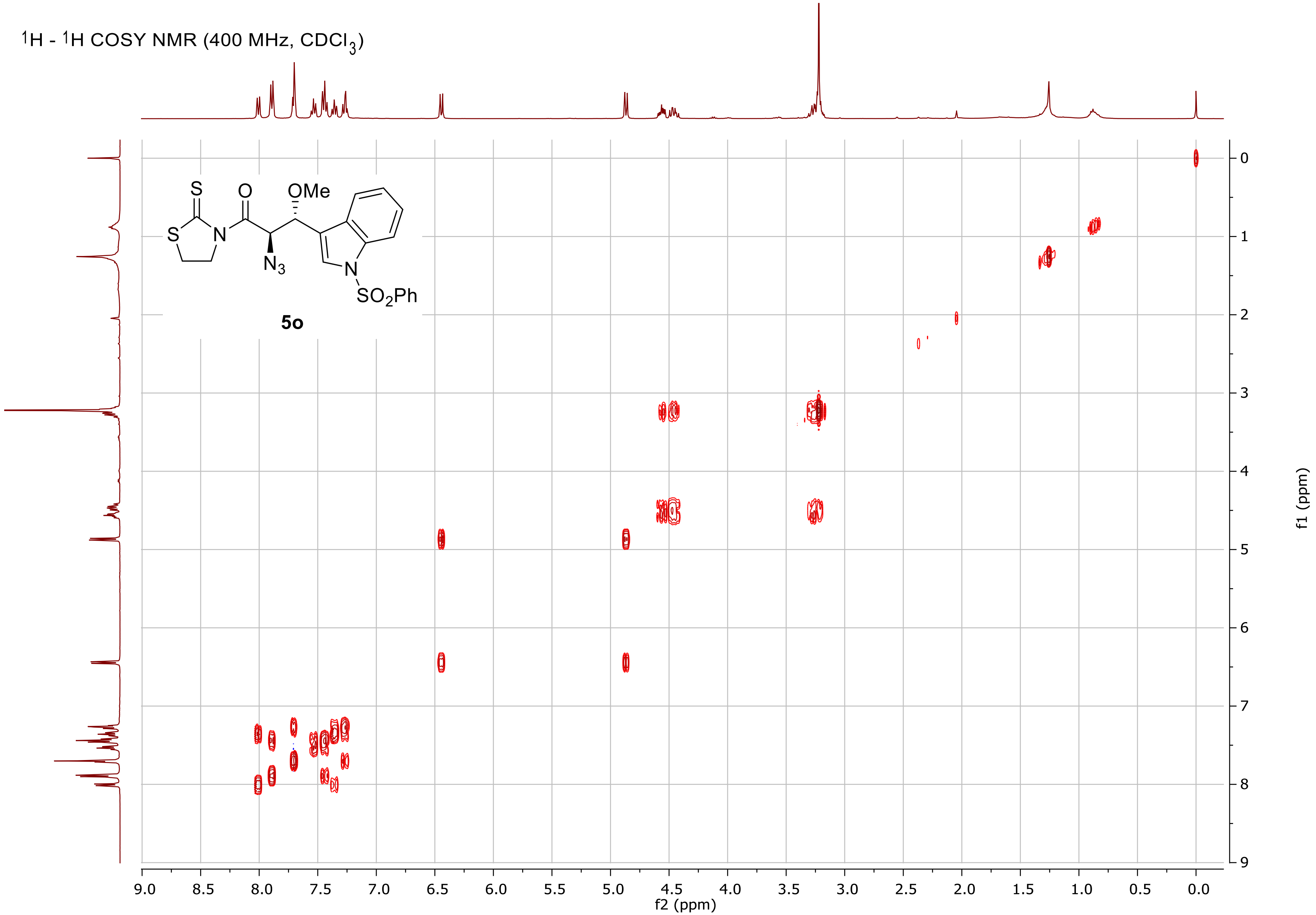

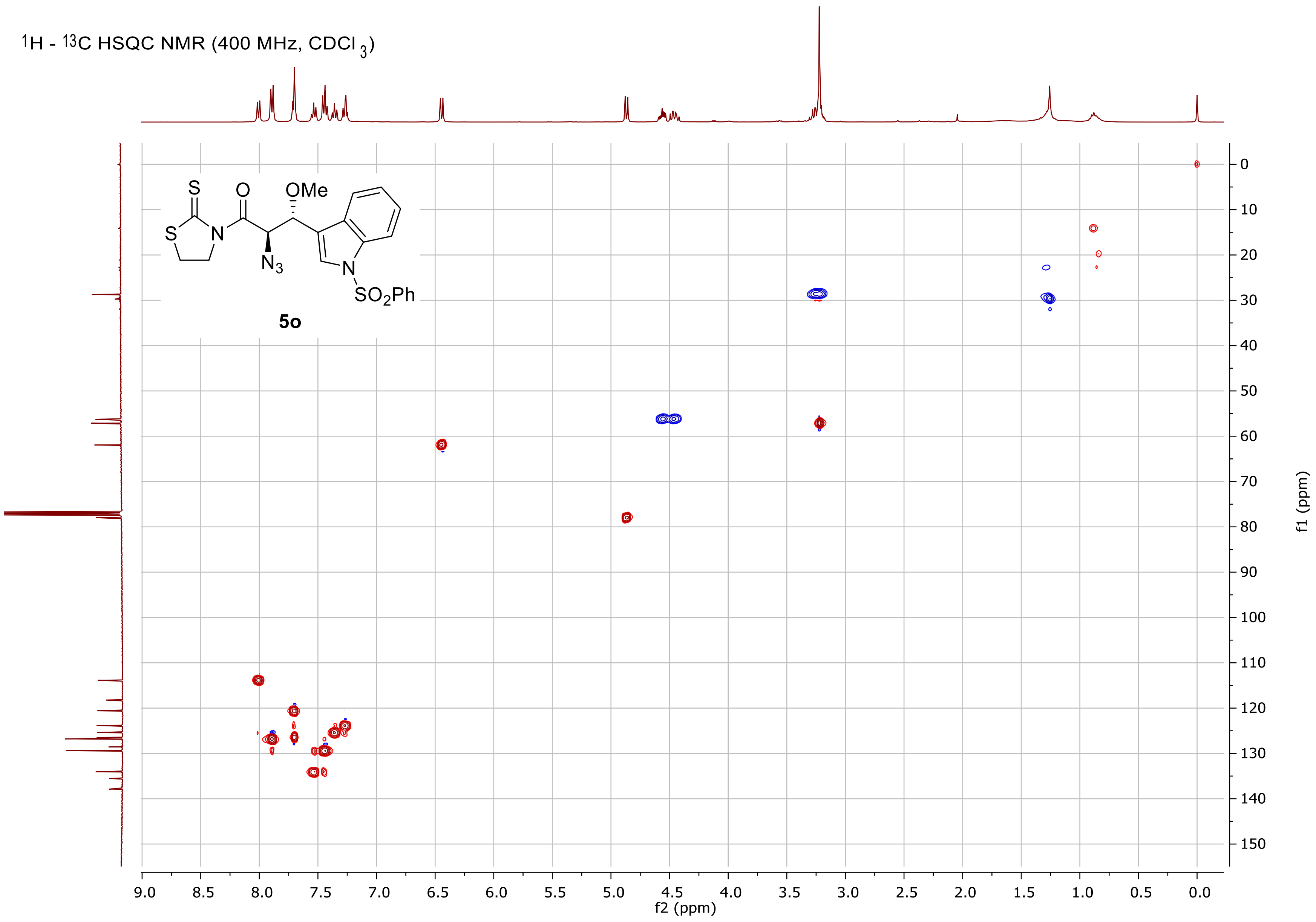

1H - 13C HSQC NMR (400 MHz, CDCl 3)
S
O
OMe
S
N
N3
N
SO2Ph
5o
f2 (ppm)
f1 (ppm)
9.0 8.5 8.0 7.5 7.0 6.5 6.0 5.5 5.0 4.5 4.0 3.5 3.0 2.5 2.0 1.5 1.0 0.5 0.0
0 10 20 30 40 50 60 70 80 90 100 110 120 130 140 150

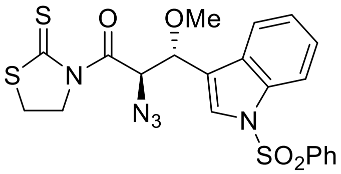

5o

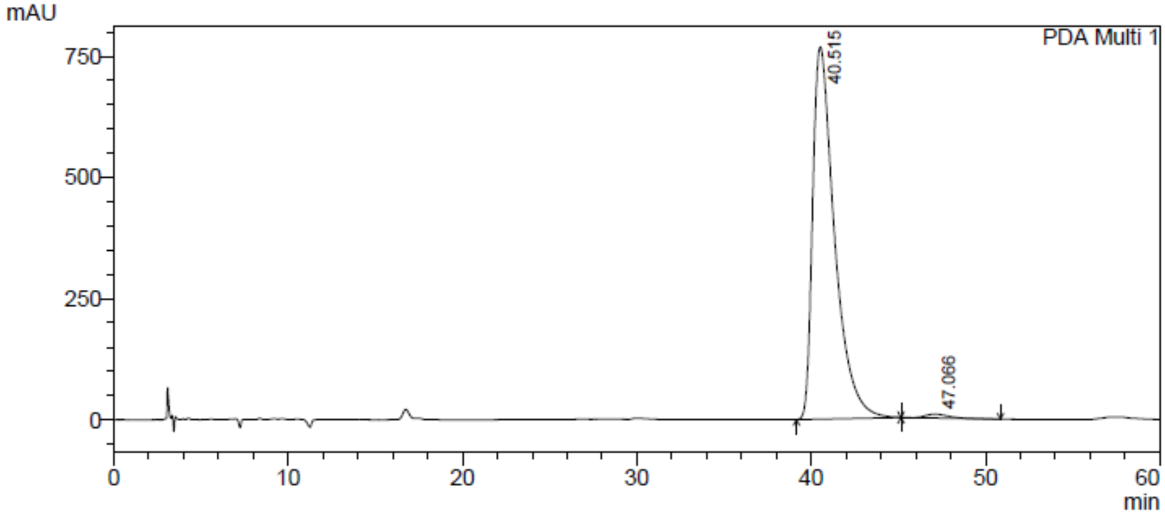

1 PDA Multi 1/254nm 4nm

PeakTable

PDA Ch1 254nm 4nm

Peak#	Ret. Time	Area	Height	Area %	Height %
1	40.515	67081857	767897	98.905	98.932
2	47.066	742801	8288	1.095	1.068
Total		67824658	776185	100.000	100.000

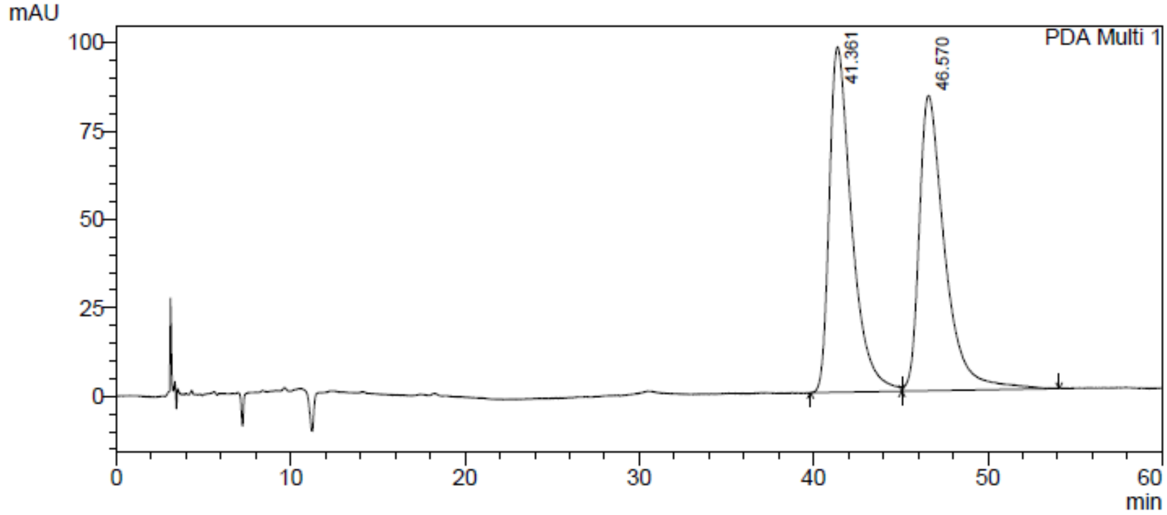

1 PDA Multi 1/254nm 4nm

PeakTable

PDA Ch1 254nm 4nm

Peak#	Ret. Time	Area	Height	Area %	Height %
1	41.361	8732145	97740	50.540	53.940
2	46.570	8545580	83463	49.460	46.060
Total		17277725	181202	100.000	100.000

^{1}H NMR (400 MHz, $CDCl_3$)

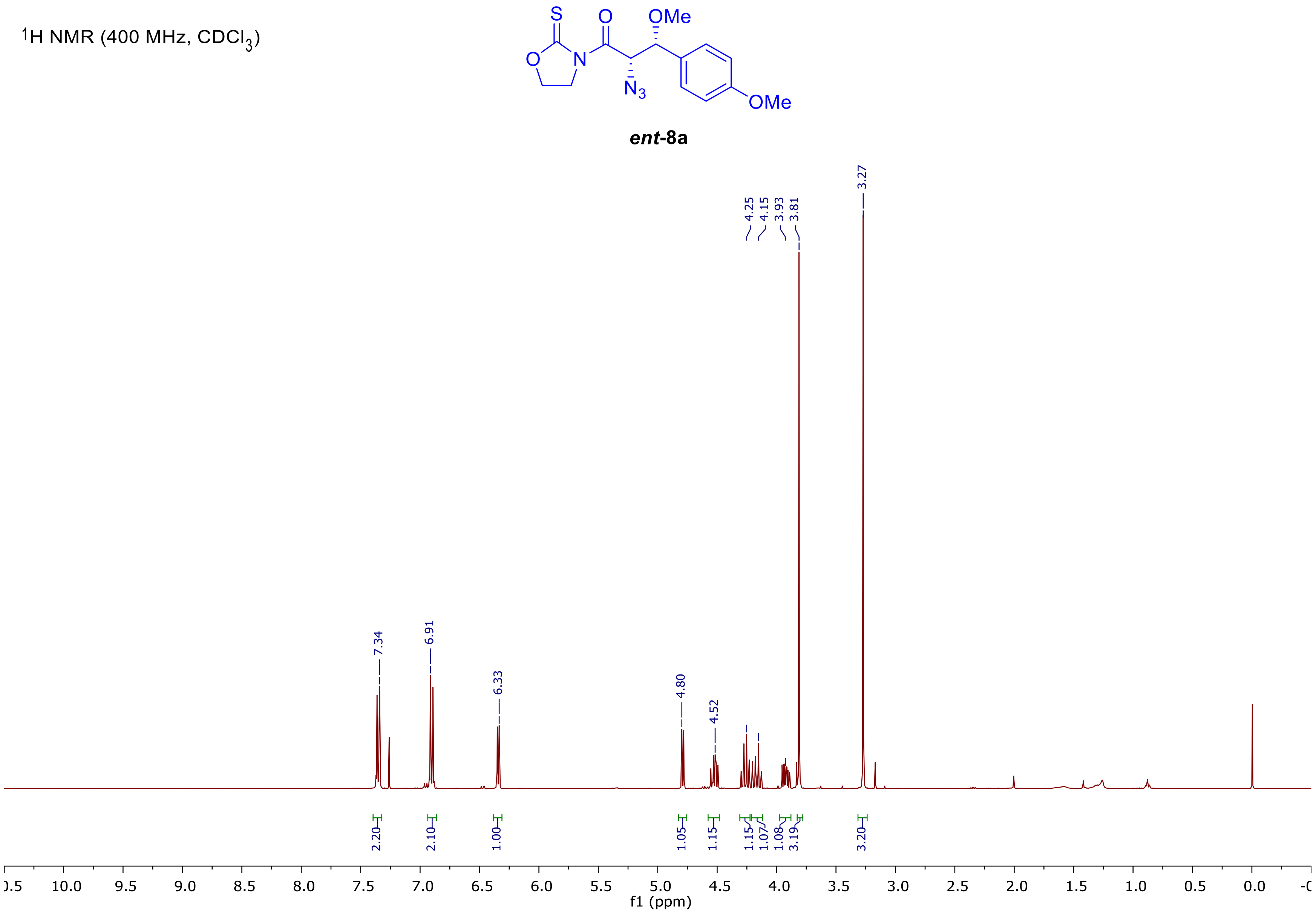

$^{13}C\{^{1}H\}$ NMR (100.6 MHz, $CDCl_3$)

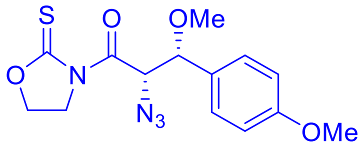

***ent*-8a**

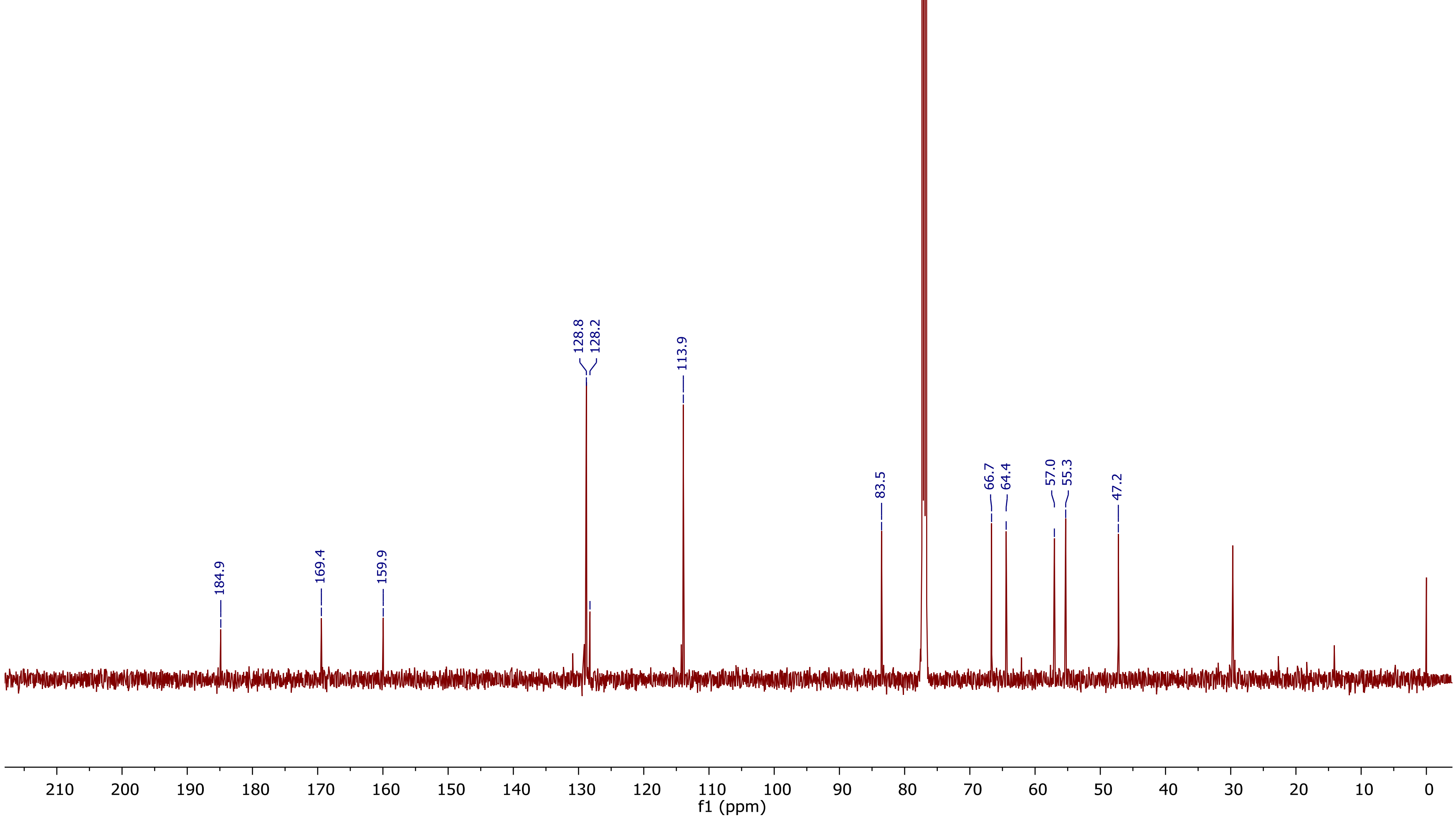

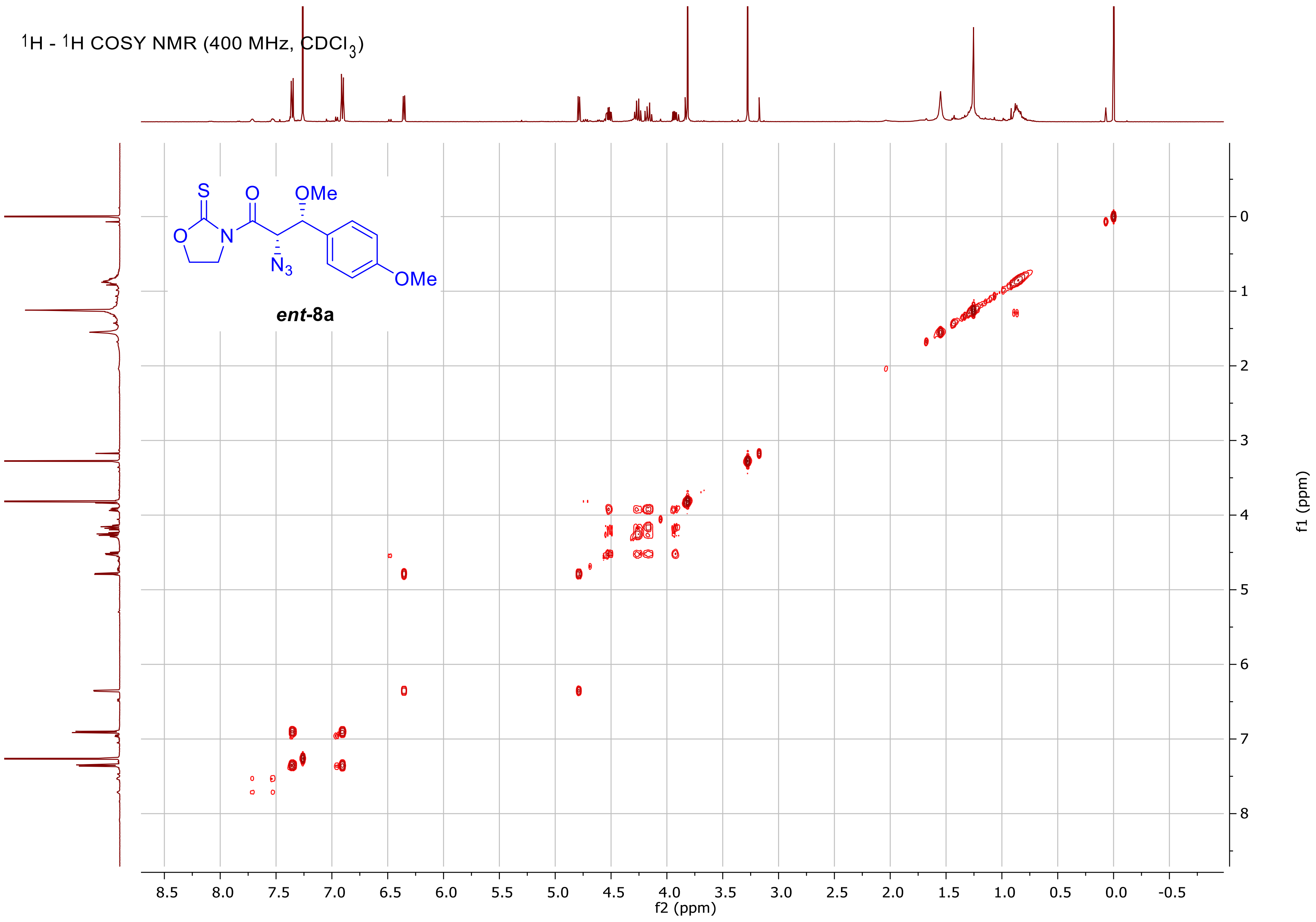

1H - 1H COSY NMR (400 MHz, CDCl3)
S
O
OMe
O
N
N3
OMe
ent-8a
f2 (ppm)
f1 (ppm)

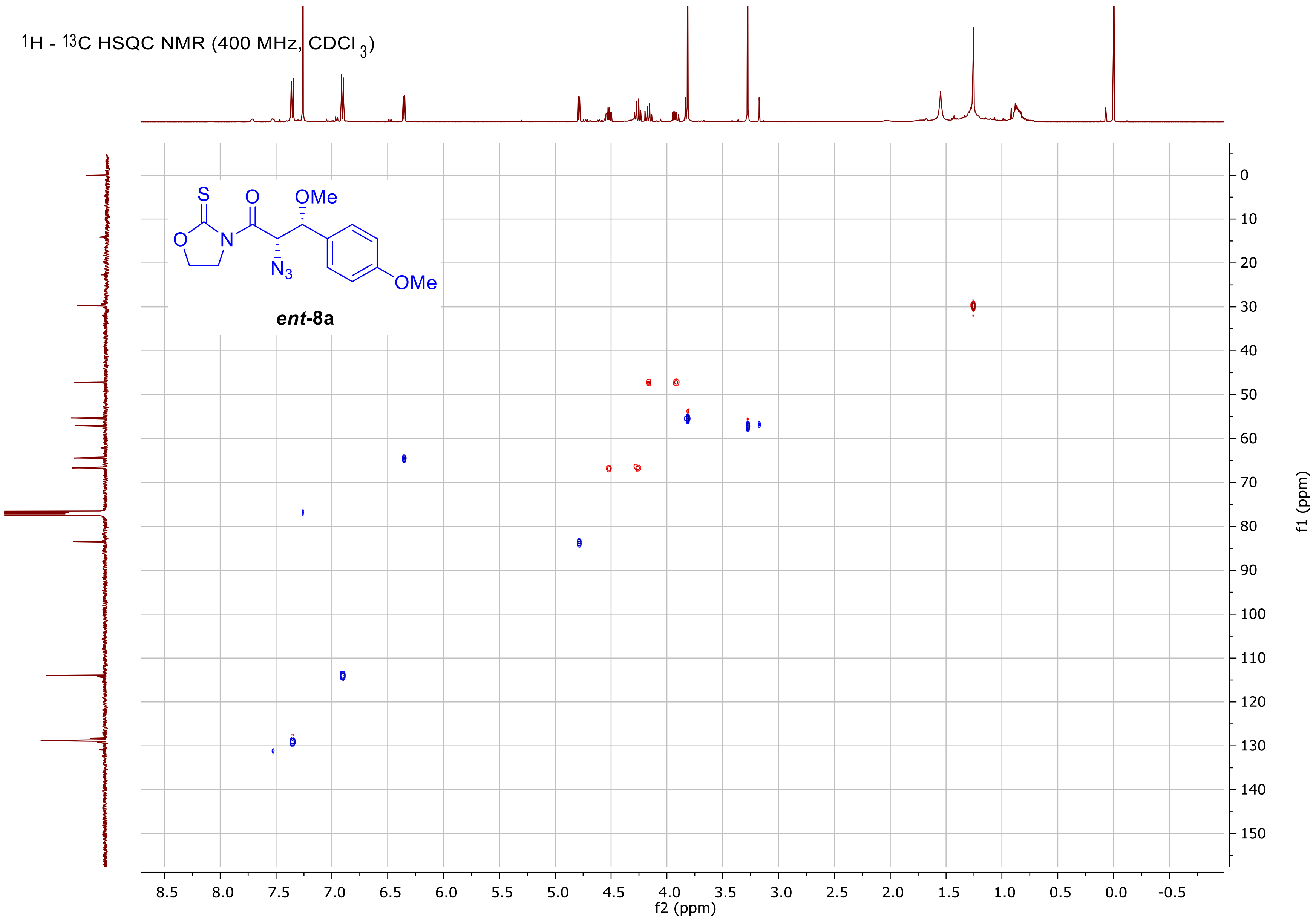
^{1}H - ^{13}C HSQC NMR (400 MHz, CDCl$_3$)
S
O
OMe
O
N
N3
OMe
ent-8a
f2 (ppm)
f1 (ppm)

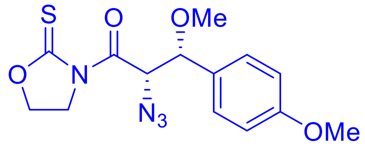

***ent*-8a**

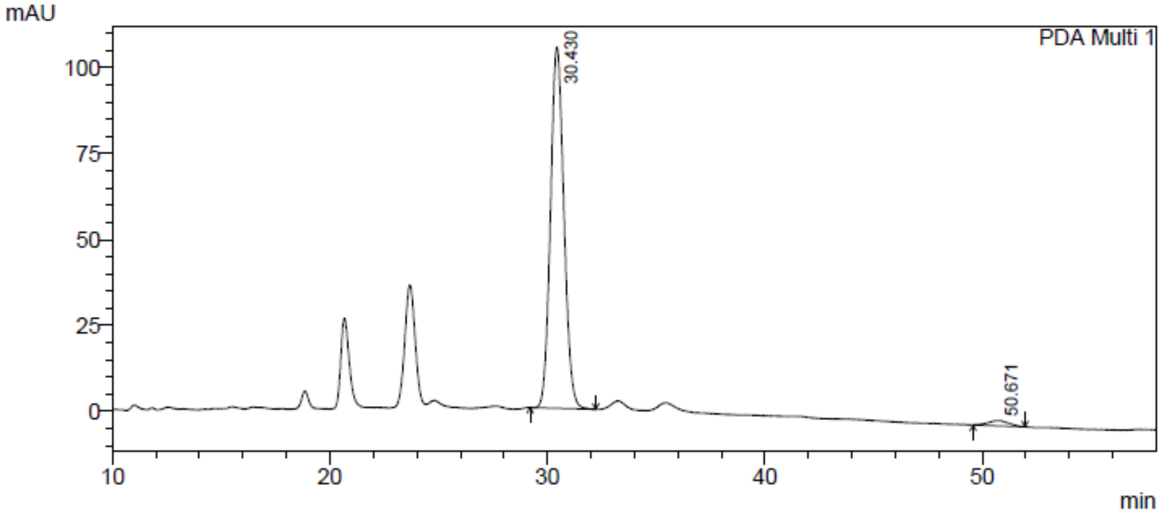

1 PDA Multi 1/254nm 4nm

PeakTable

PDA Ch1 254nm 4nm

Peak#	Ret. Time	Area	Height	Area %	Height %
1	30.430	4507870	104990	97.879	98.588
2	50.671	97704	1503	2.121	1.412
Total		4605574	106494	100.000	100.000

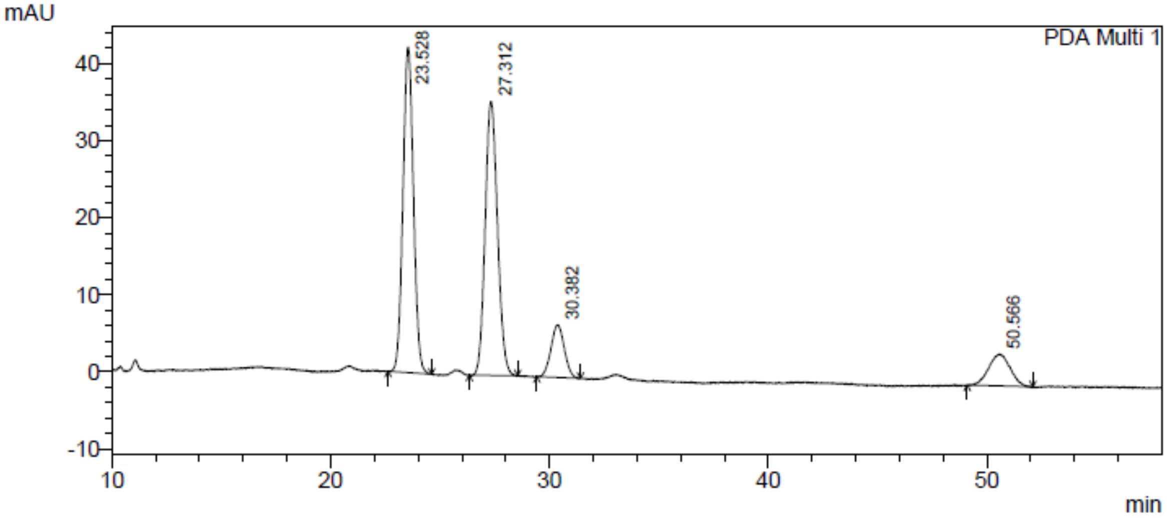

1 PDA Multi 1/254nm 4nm

PeakTable

PDA Ch1 254nm 4nm

Peak#	Ret. Time	Area	Height	Area %	Height %
1	23.528	1403712	42249	41.786	47.576
2	27.312	1392512	35578	41.452	40.063
3	30.382	289083	6841	8.605	7.703
4	50.566	274016	4136	8.157	4.658
Total		3359323	88804	100.000	100.000

^{1}H NMR (400 MHz, $CDCl_3$)

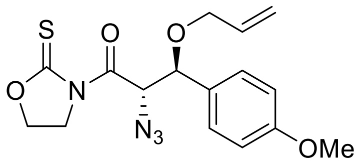

***ent*-6b**

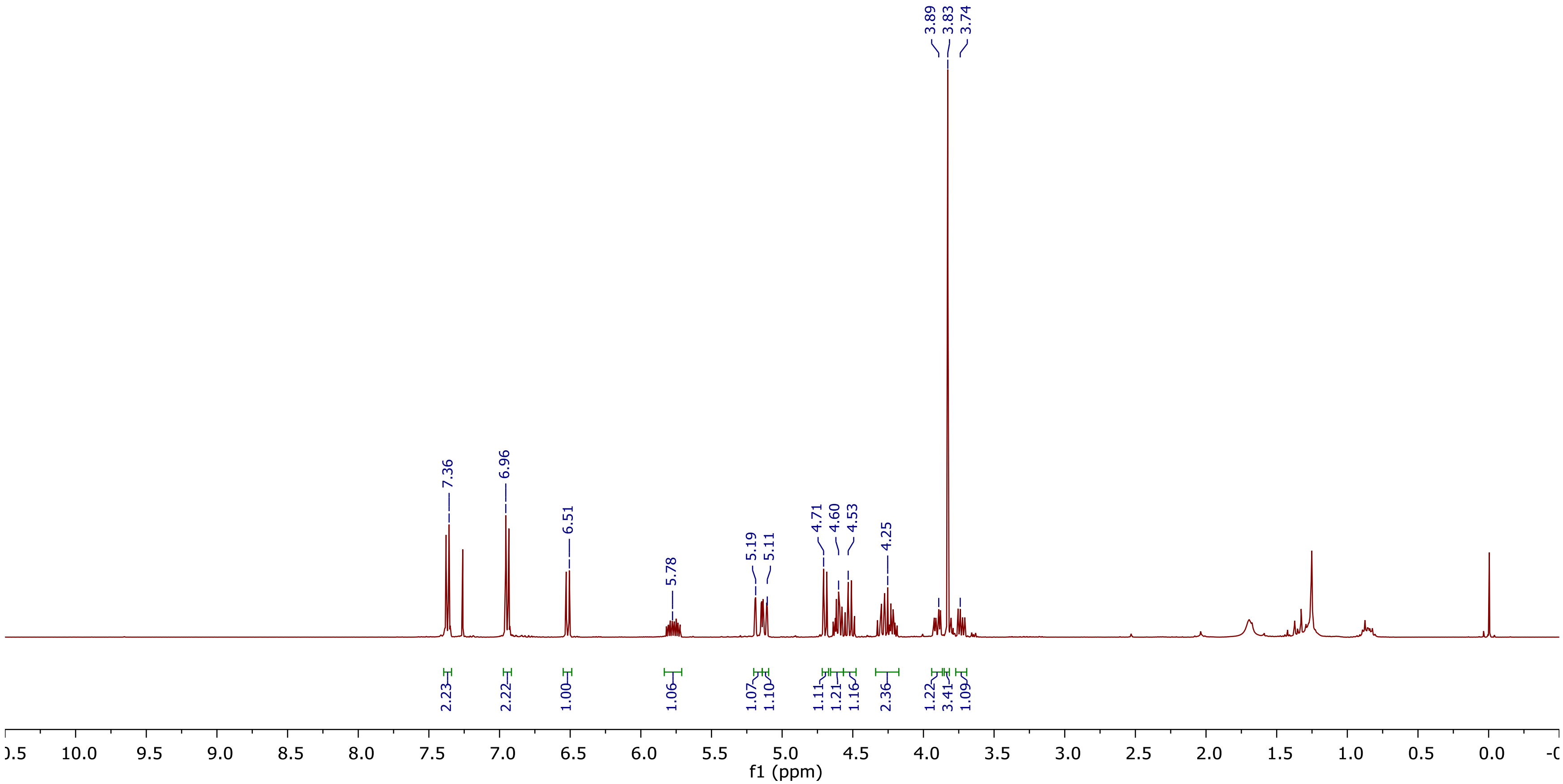

$^{13}C\{^{1}H\}$ NMR (100.6 MHz, $CDCl_3$)

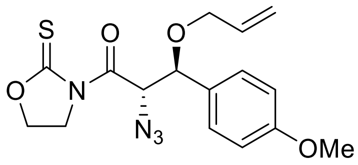

***ent*-6b**

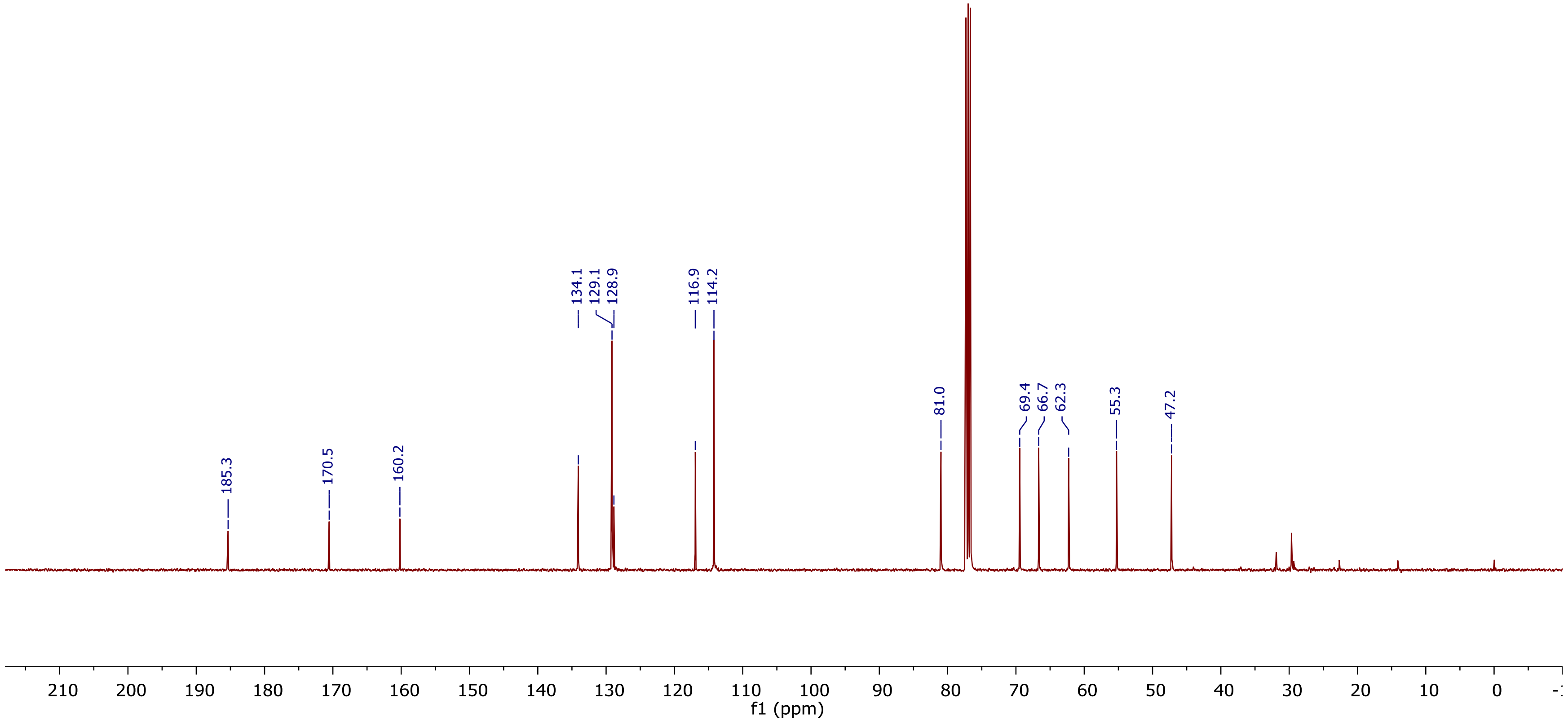

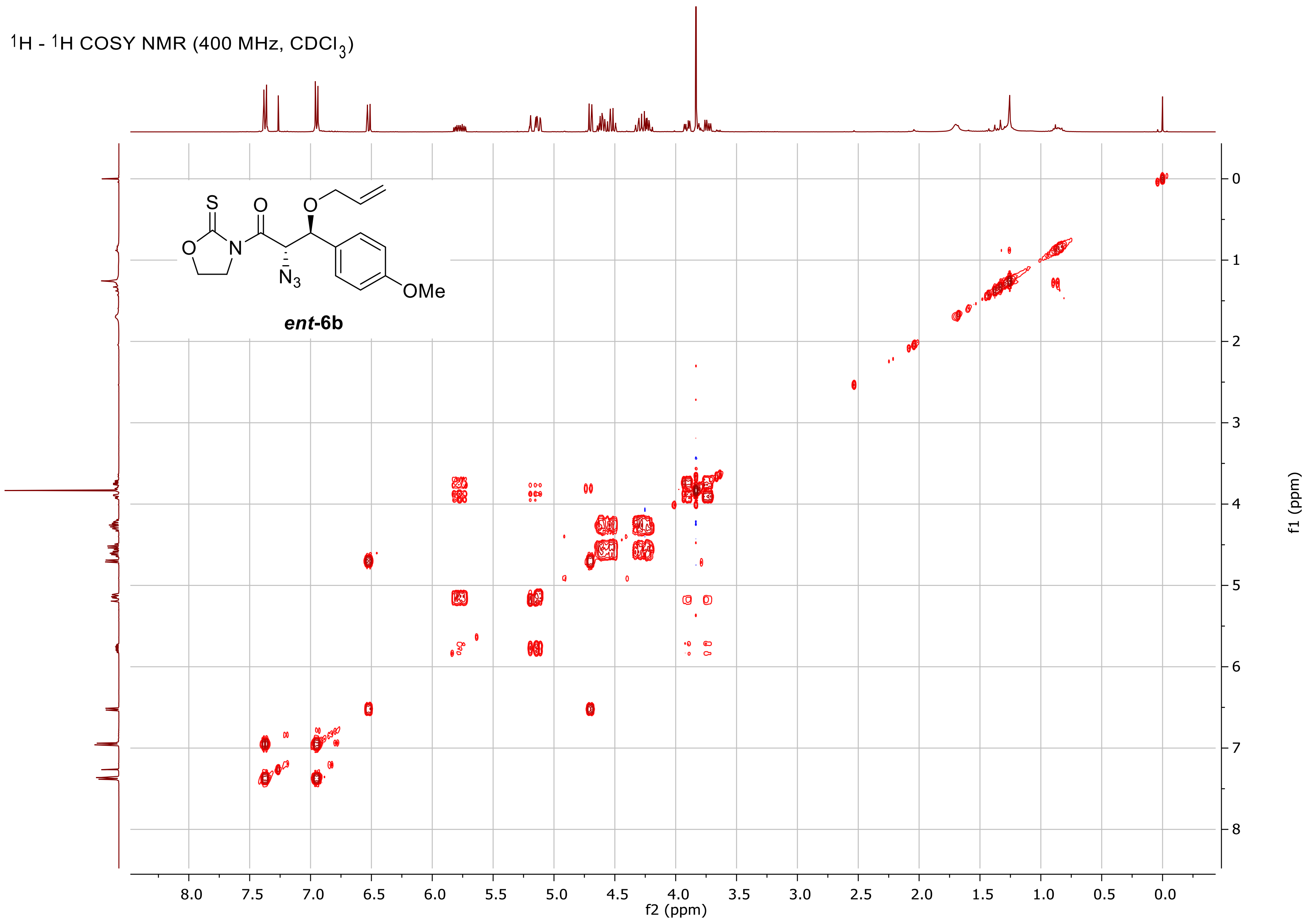
1H - 1H COSY NMR (400 MHz, CDCl3)
S
O
O
N
O
N3
OMe
ent-6b
f2 (ppm)
f1 (ppm)

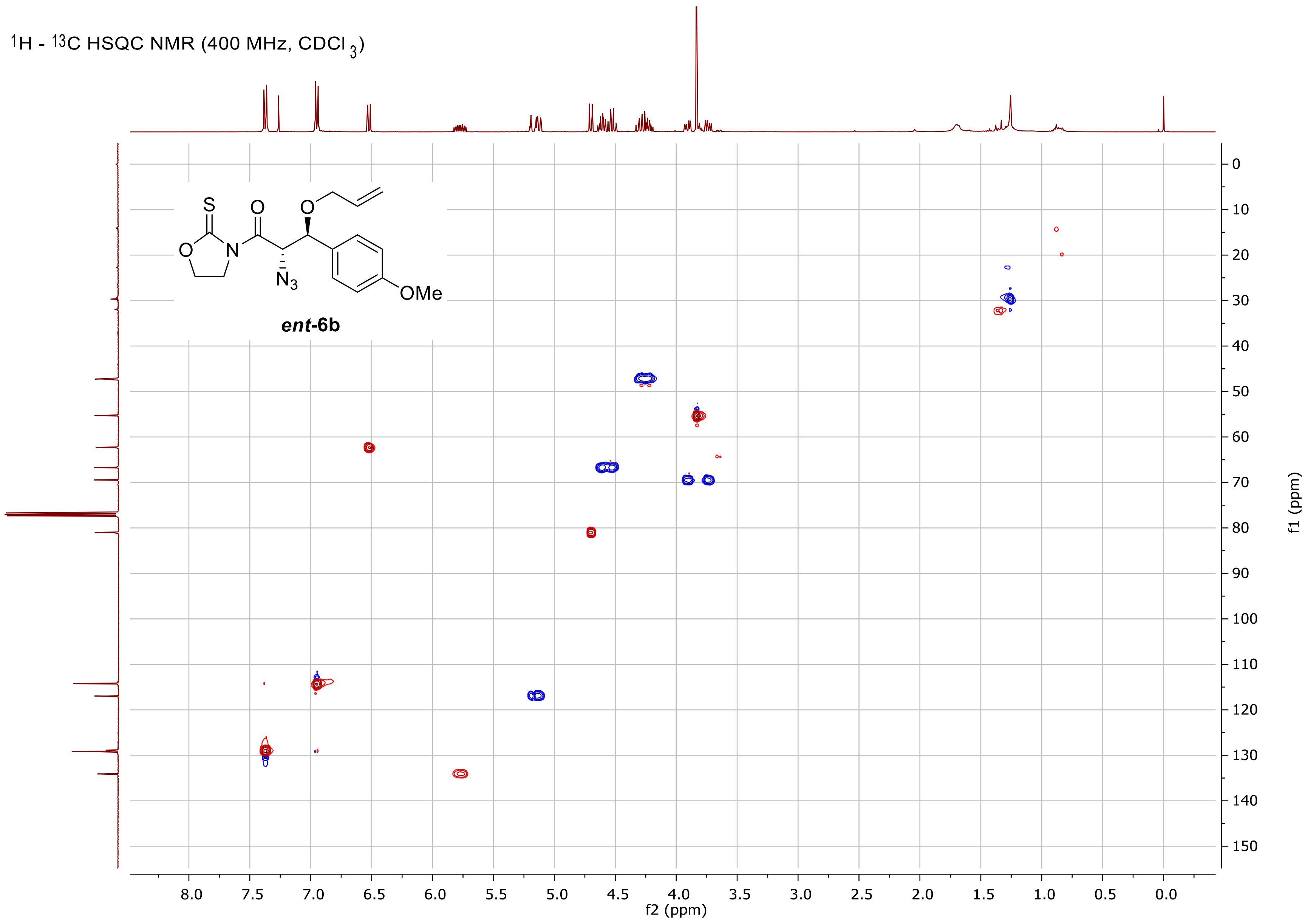
^{1}H - ^{13}C HSQC NMR (400 MHz, CDCl$_3$)
S
O
O
N
O
N3
OMe
ent-6b
f2 (ppm)
f1 (ppm)

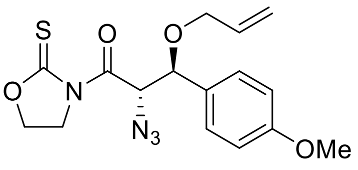

***ent*-6b**

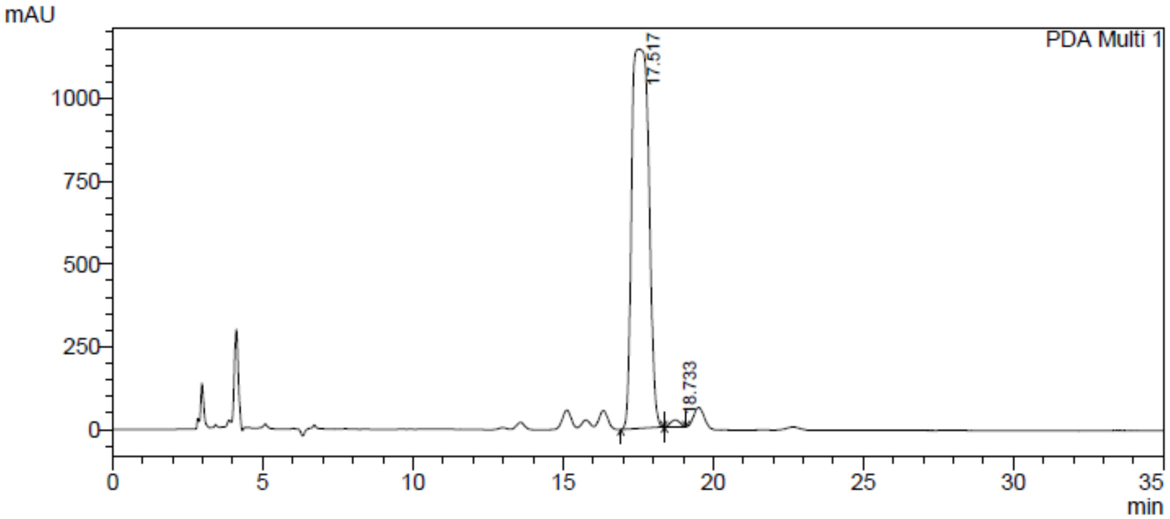

1 PDA Multi 1/254nm 4nm

PeakTable

PDA Ch1 254nm 4nm

Peak#	Ret. Time	Area	Height	Area %	Height %
1	17.517	45309324	1146062	99.025	98.263
2	18.733	445895	20260	0.975	1.737
Total		45755219	1166323	100.000	100.000

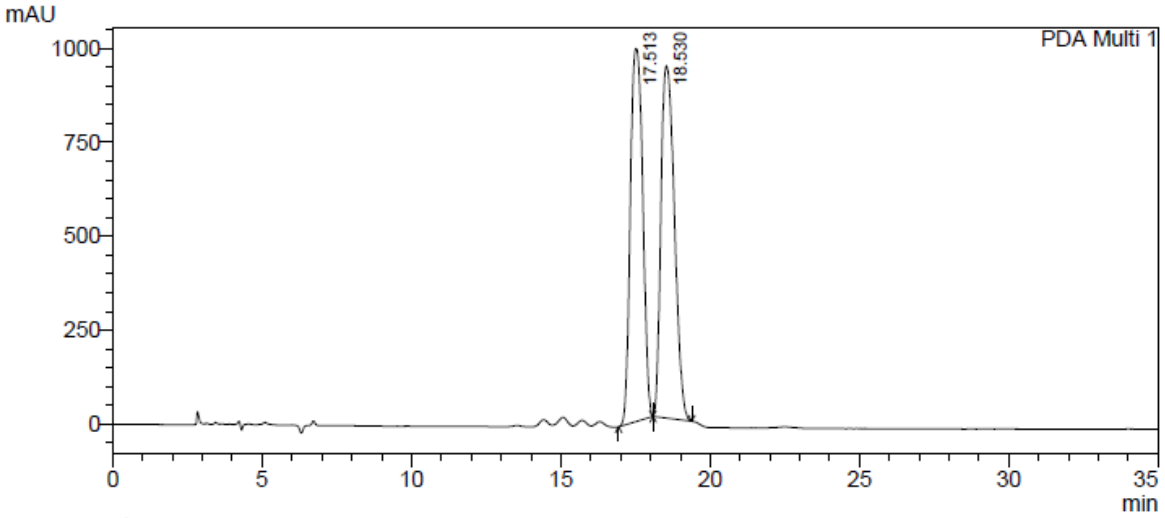

1 PDA Multi 1/254nm 4nm

PeakTable

PDA Ch1 254nm 4nm

Peak#	Ret. Time	Area	Height	Area %	Height %
1	17.513	28602638	993109	49.314	51.445
2	18.530	29397847	937302	50.686	48.555
Total		58000485	1930411	100.000	100.000

^{1}H NMR (400 MHz, $CDCl_3$)

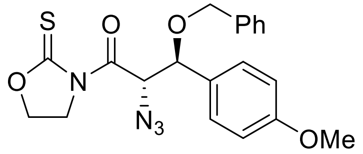

***ent*-6c**

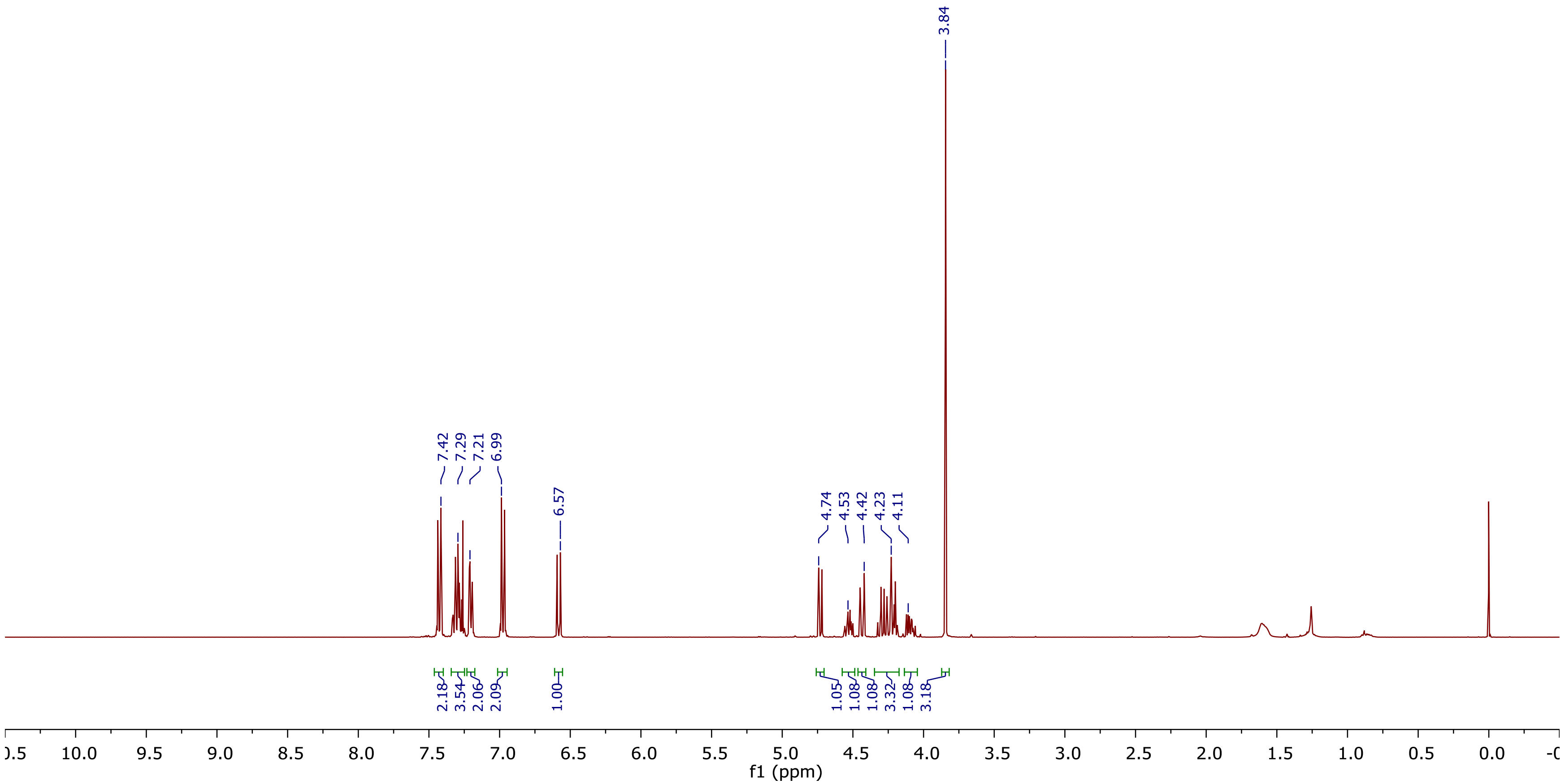

$^{13}C\{^1H\}$ NMR (100.6 MHz, $CDCl_3$)

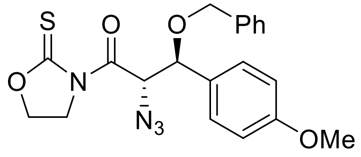

***ent*-6c**

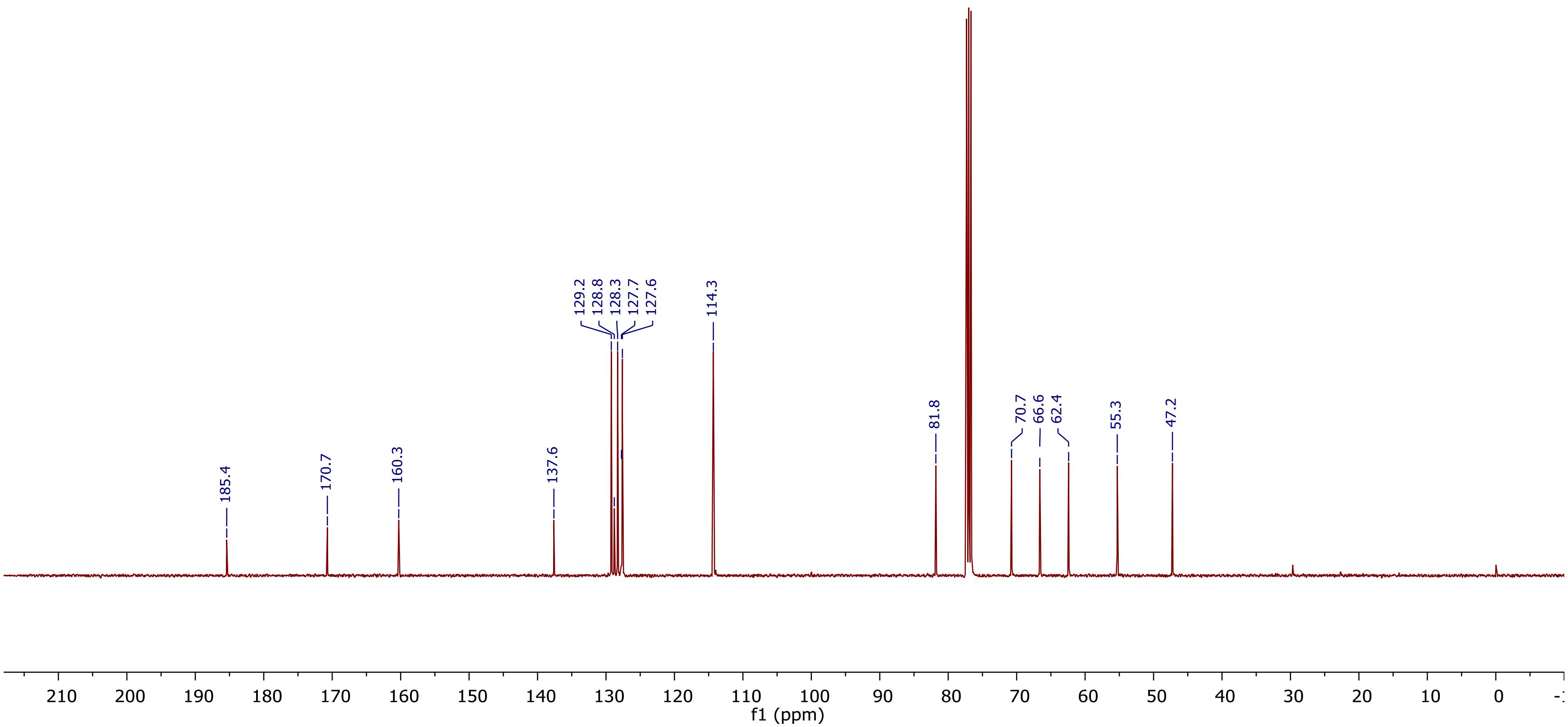

^1H - ^1H COSY NMR (400 MHz, $CDCl_3$)

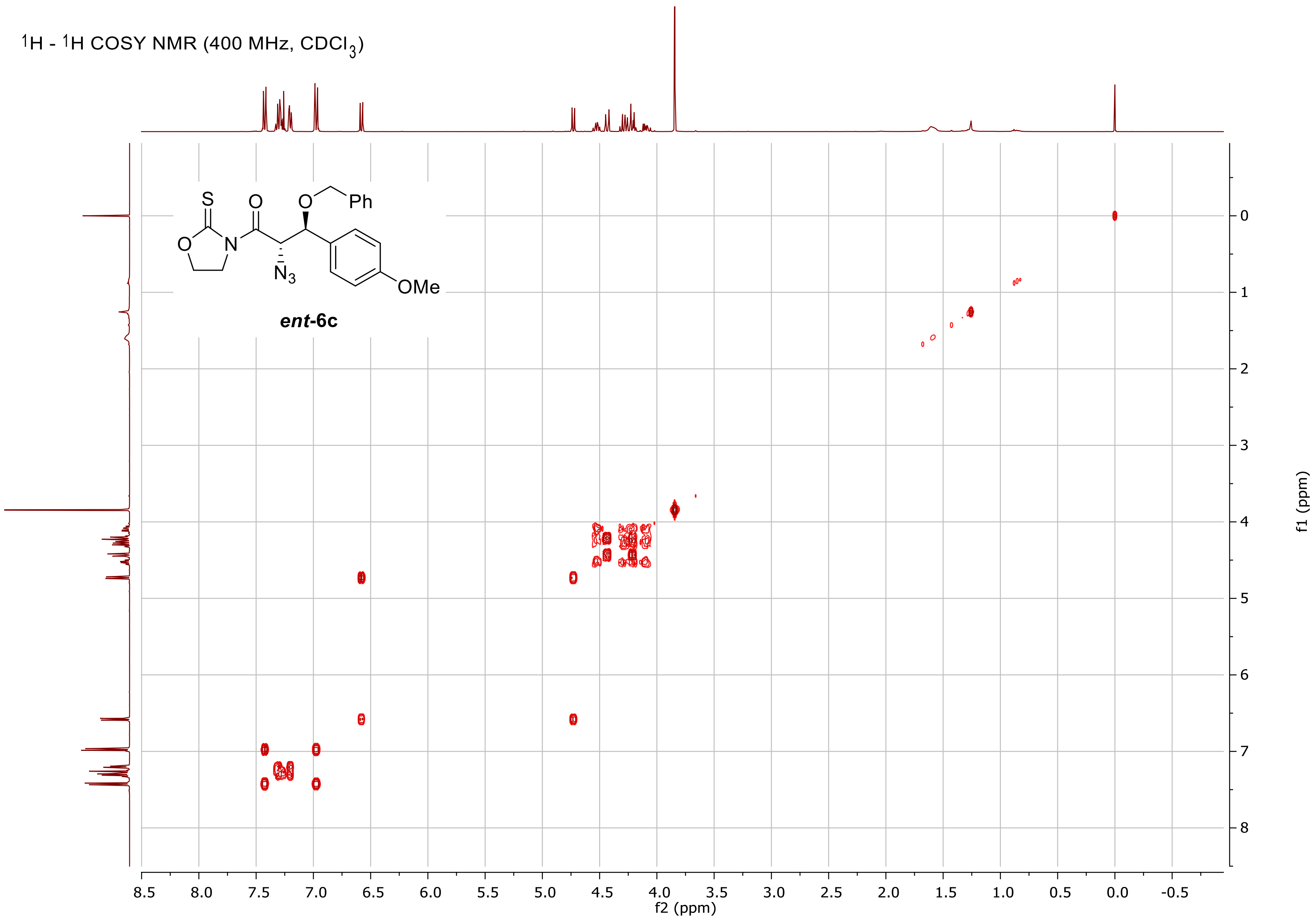

^1H - ^{13}C HSQC NMR (400 MHz, $CDCl_3$)

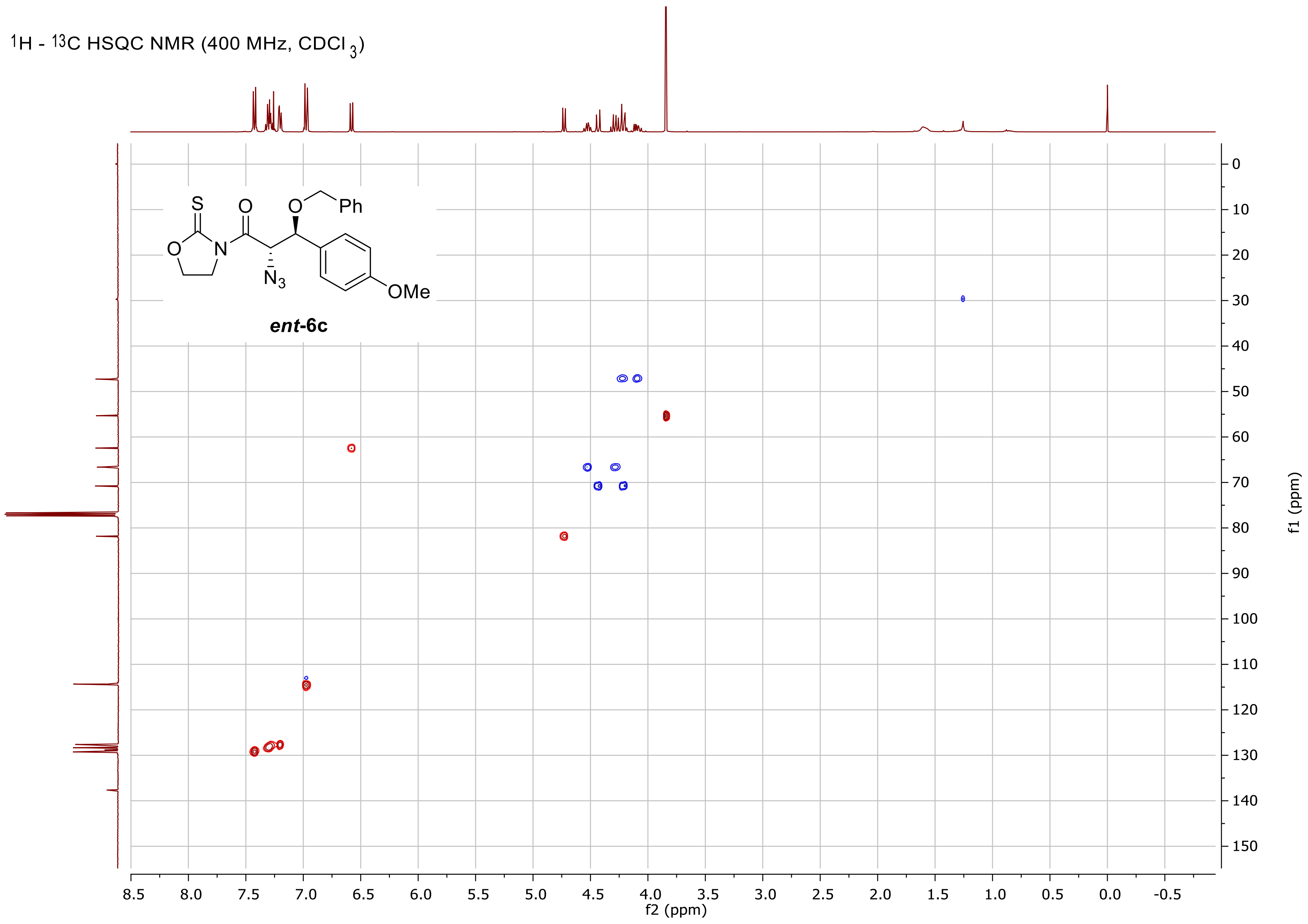

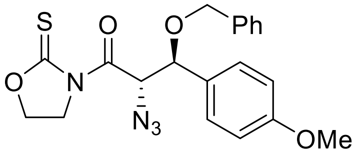

***ent*-6c**

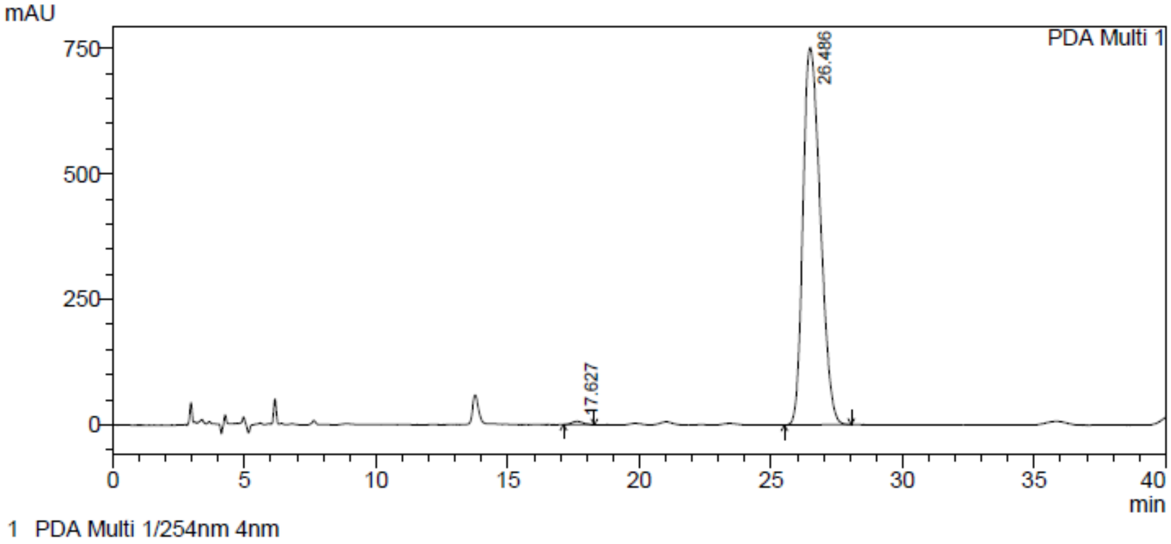

PeakTable

PDA Ch1 254nm 4nm

Peak#	Ret. Time	Area	Height	Area %	Height %
1	17.627	190337	6735	0.550	0.889
2	26.486	34386047	751259	99.450	99.111
Total		34576383	757995	100.000	100.000

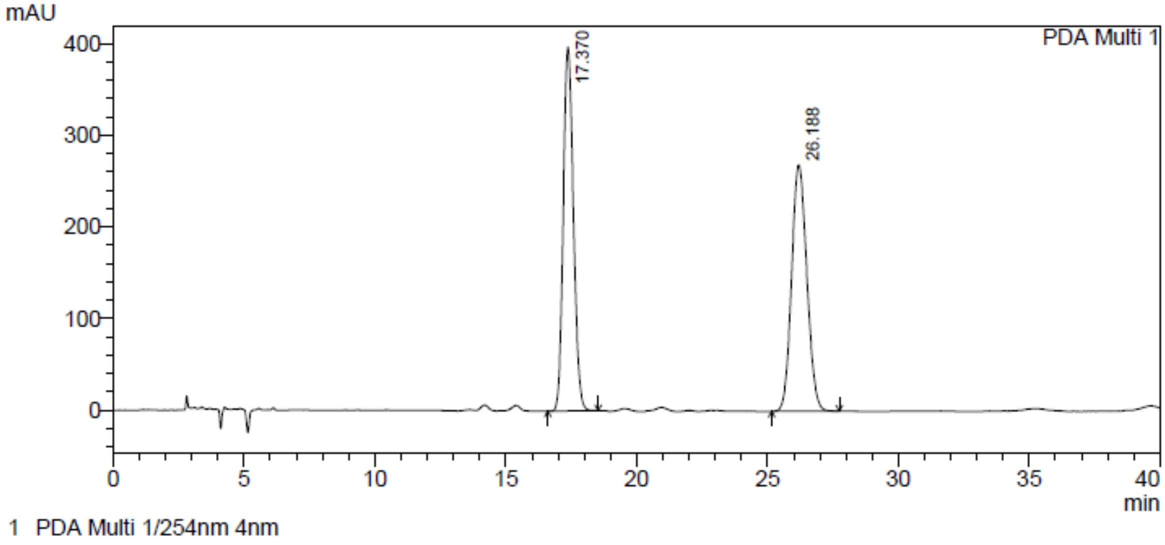

PeakTable

PDA Ch1 254nm 4nm

Peak#	Ret. Time	Area	Height	Area %	Height %
1	17.370	10710174	397403	49.568	59.631
2	26.188	10896690	269029	50.432	40.369
Total		21606864	666432	100.000	100.000

^1H NMR (400 MHz, $CDCl_3$)

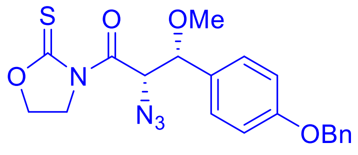

***ent*-8d**

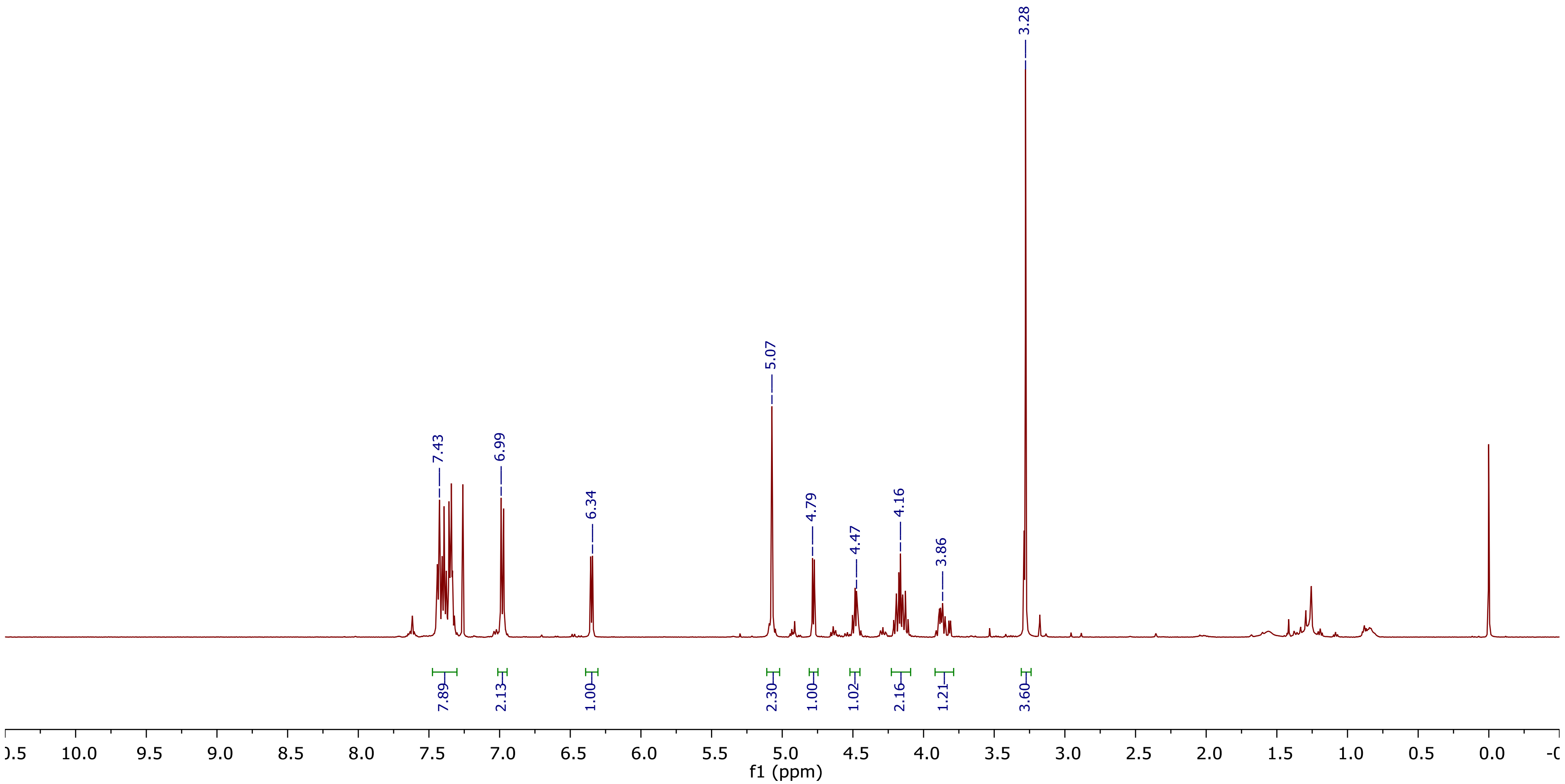

$^{13}C\{^1H\}$ NMR (100.6 MHz, $CDCl_3$)

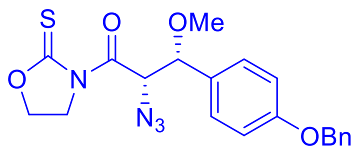

***ent*-8d**

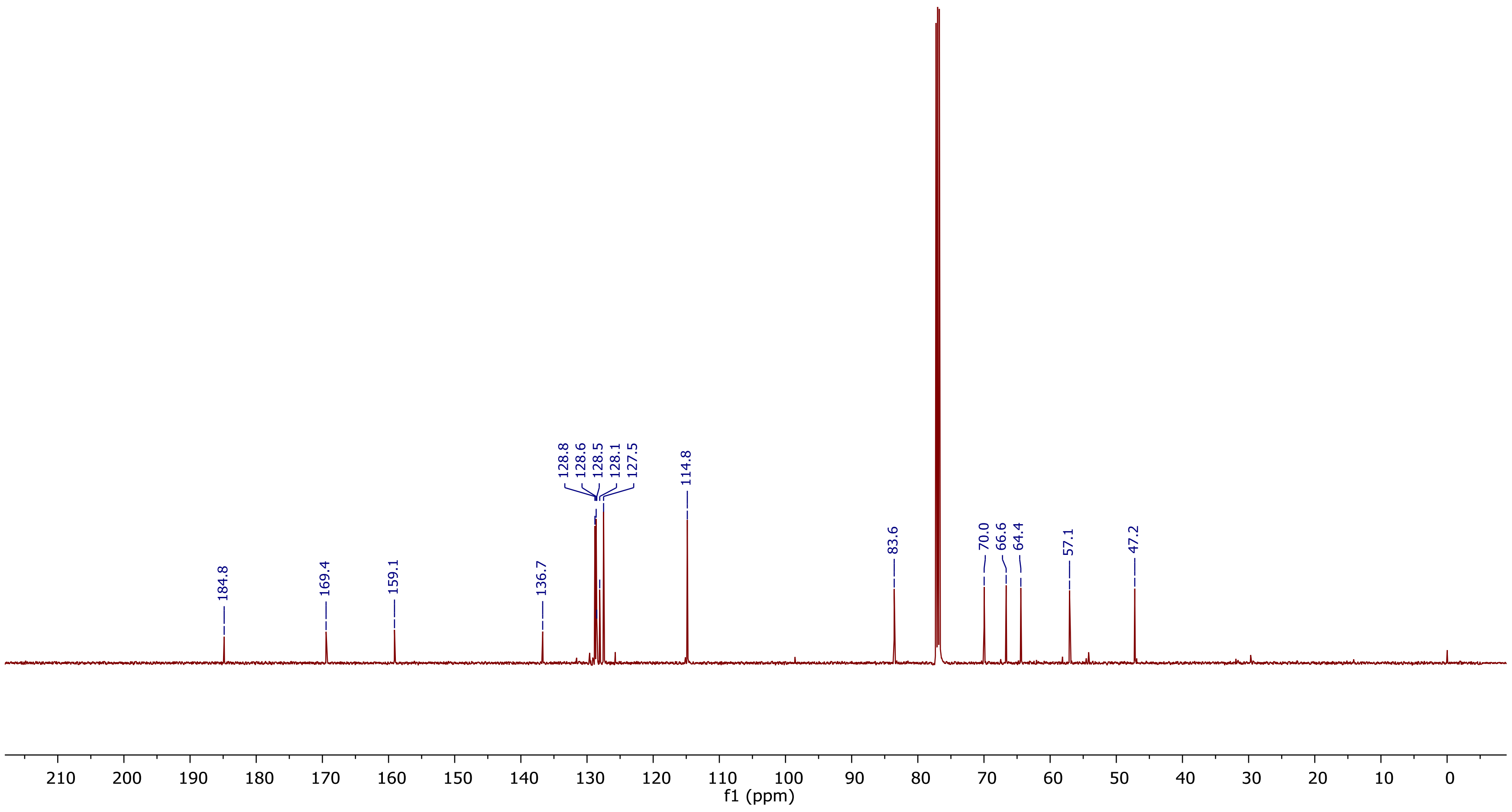

^{1}H - ^{1}H COSY NMR (400 MHz, $CDCl_3$)

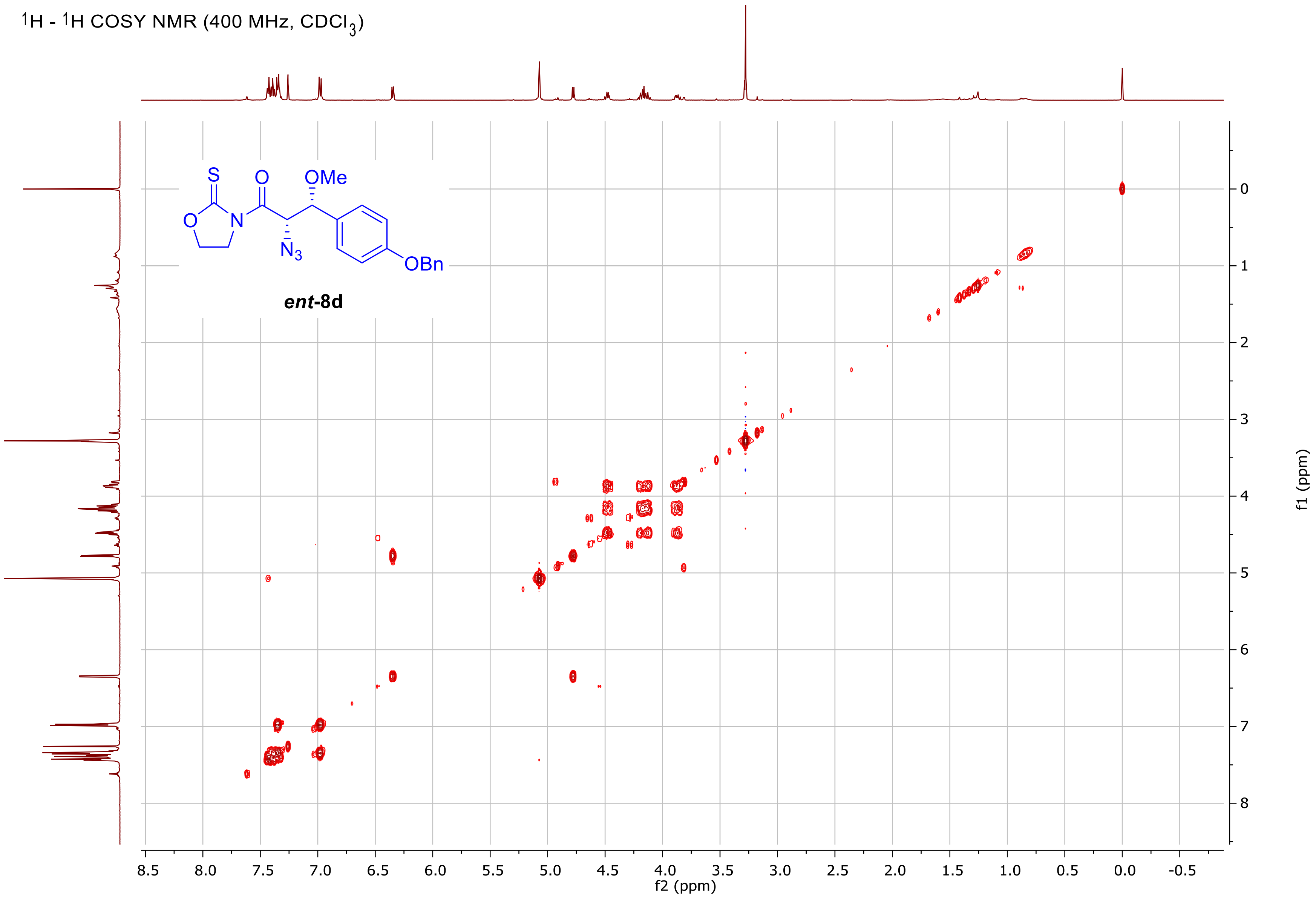

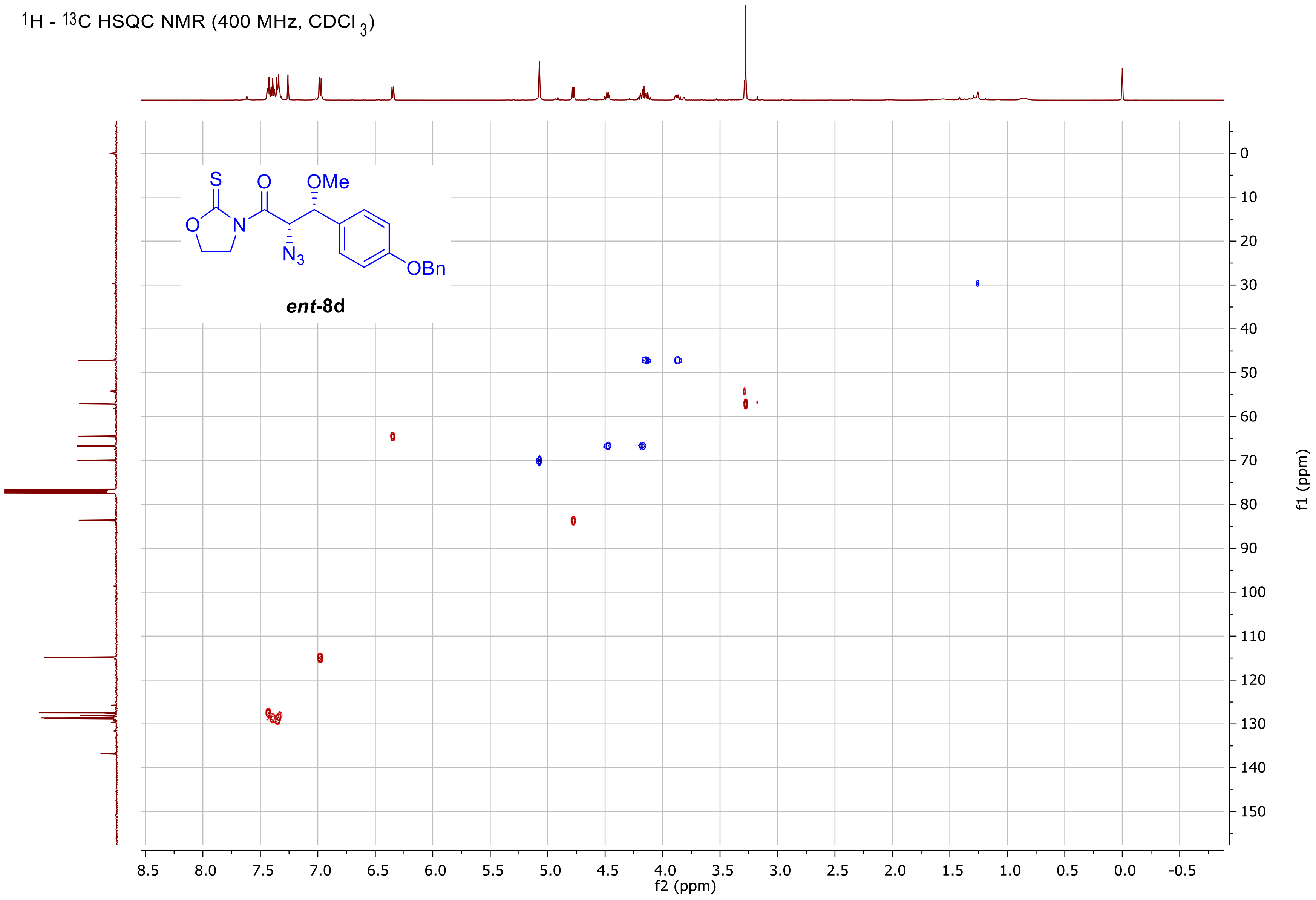

^{1}H - ^{13}C HSQC NMR (400 MHz, $CDCl_3$)
S
O
OMe
O
N
N_3
OBn
ent-**8d**
f2 (ppm)
f1 (ppm)

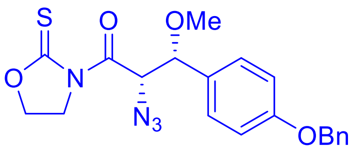

***ent*-8d**

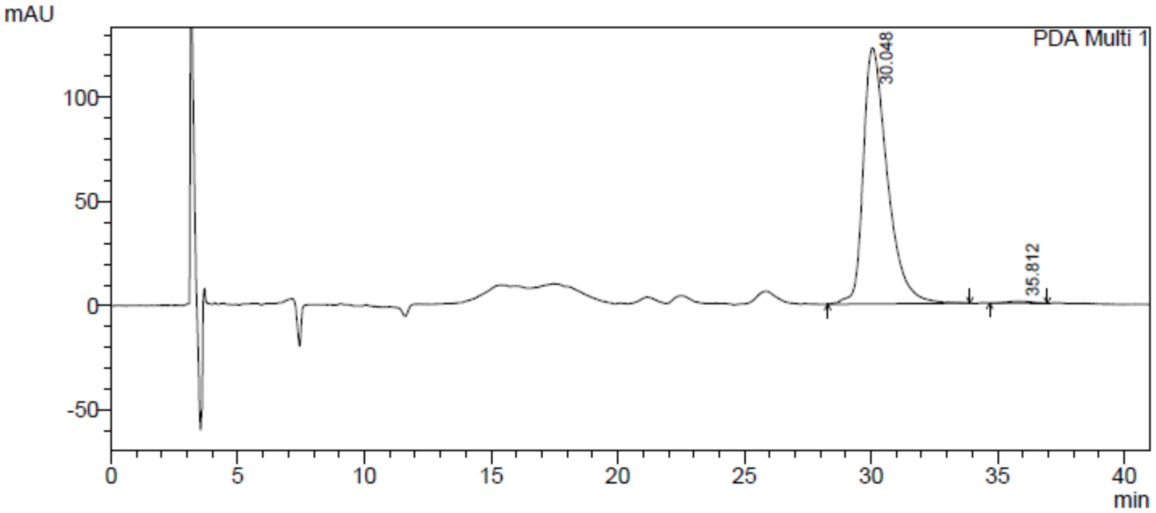

1 PDA Multi 1/254nm 4nm

PeakTable

PDA Ch1 254nm 4nm

Peak#	Ret. Time	Area	Height	Area %	Height %
1	30.048	8122797	122741	99.237	99.195
2	35.812	62487	996	0.763	0.805
Total		8185285	123736	100.000	100.000

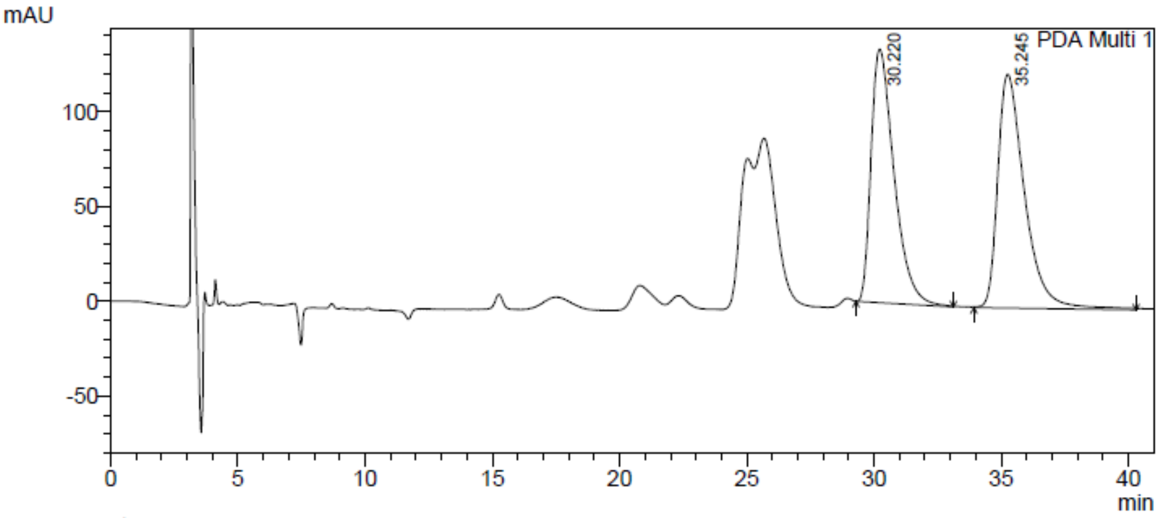

1 PDA Multi 1/254nm 4nm

PeakTable

PDA Ch1 254nm 4nm

Peak#	Ret. Time	Area	Height	Area %	Height %
1	30.220	8417539	133705	48.167	52.036
2	35.245	9058315	123244	51.833	47.964
Total		17475853	256949	100.000	100.000

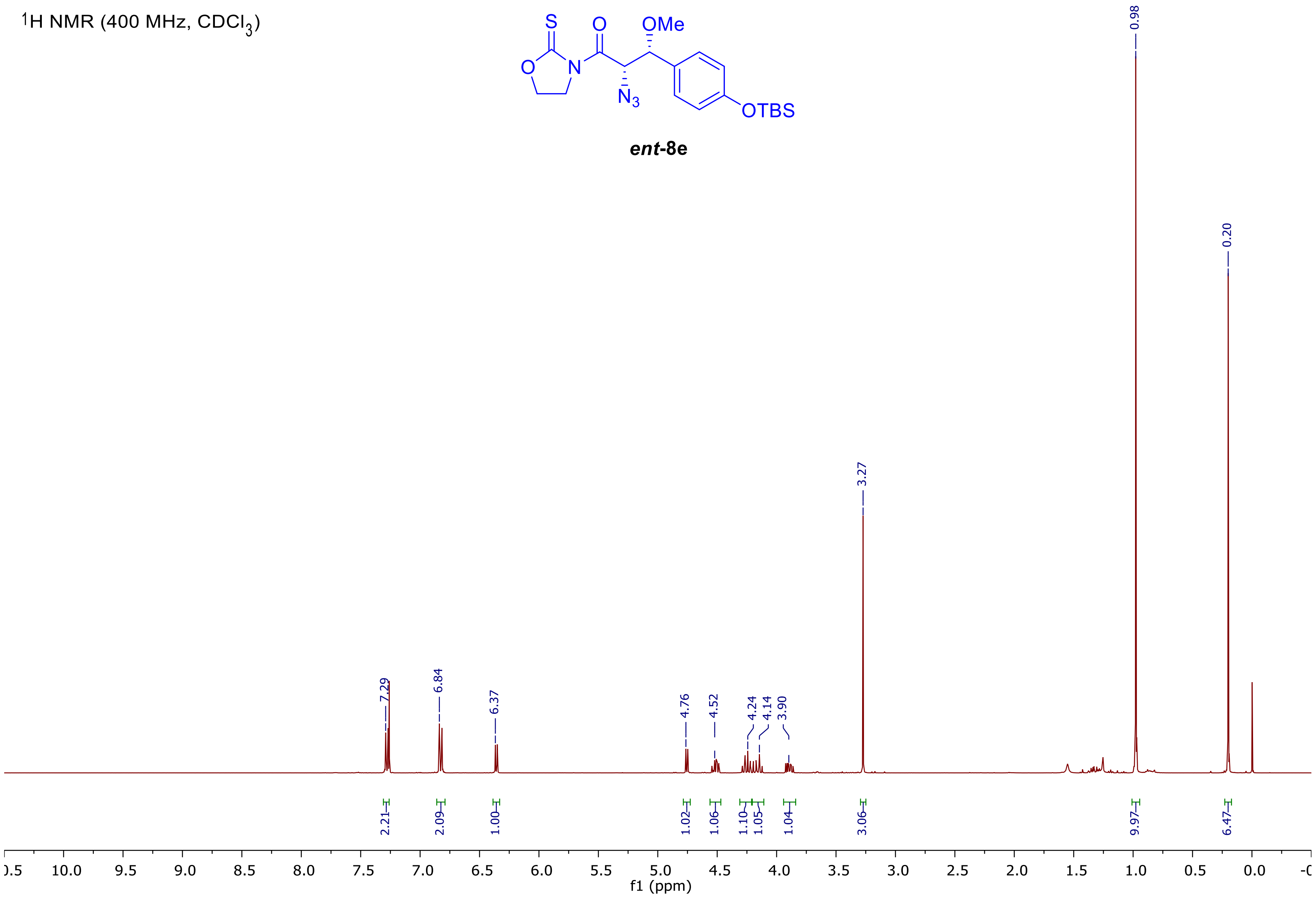
1H NMR (400 MHz, CDCl3)
OMe
N3
OTBS
ent-8e
0.98
0.20
3.27
7.29
6.84
6.37
4.76
4.52
4.24
4.14
3.90
2.21
2.09
1.00
1.02
1.06
1.10
1.05
1.04
3.06
9.97
6.47
f1 (ppm)

$^{13}C\{^1H\}$ NMR (100.6 MHz, $CDCl_3$)

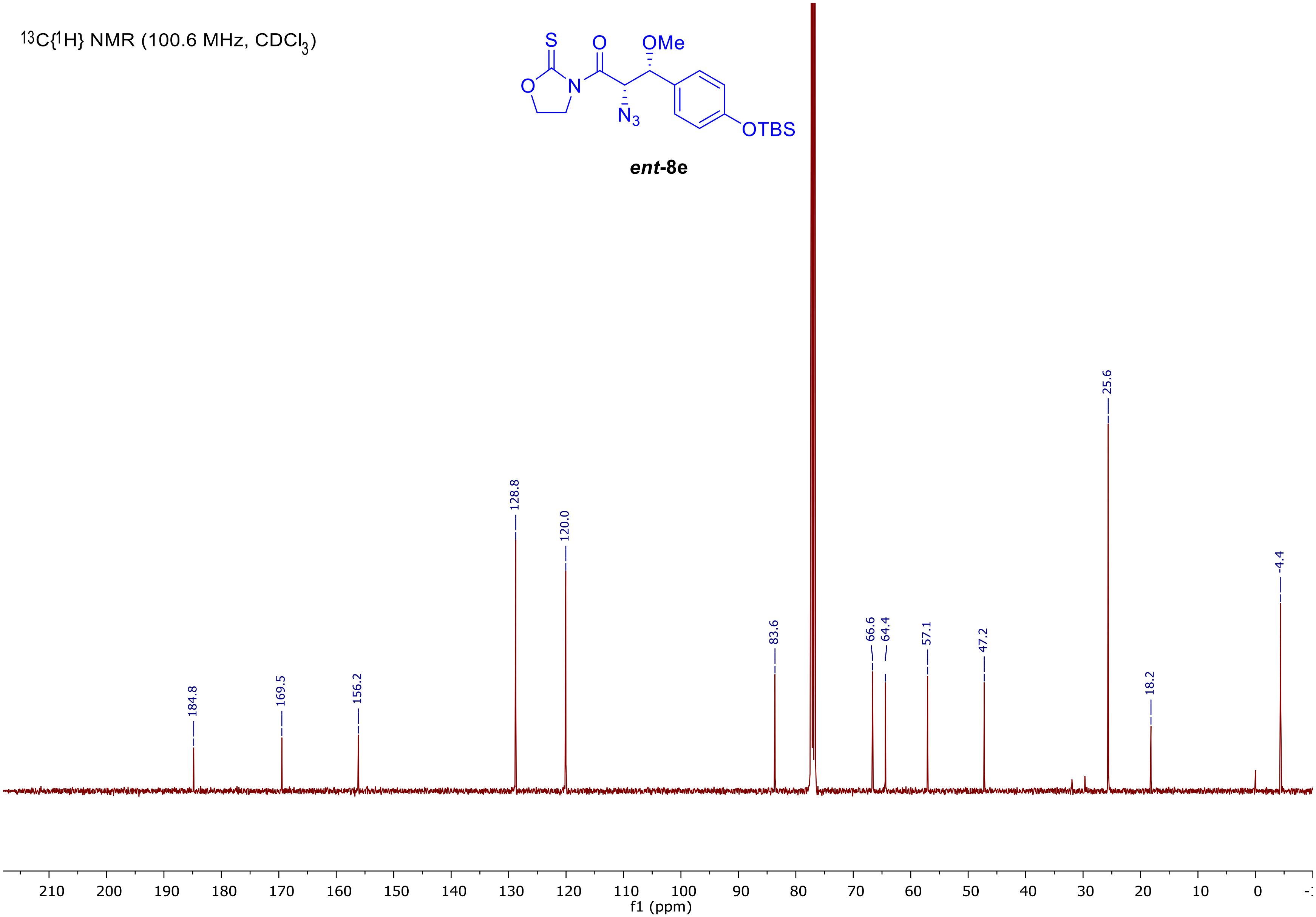

ent-**8e**

^1H - ^1H COSY NMR (400 MHz, $CDCl_3$)

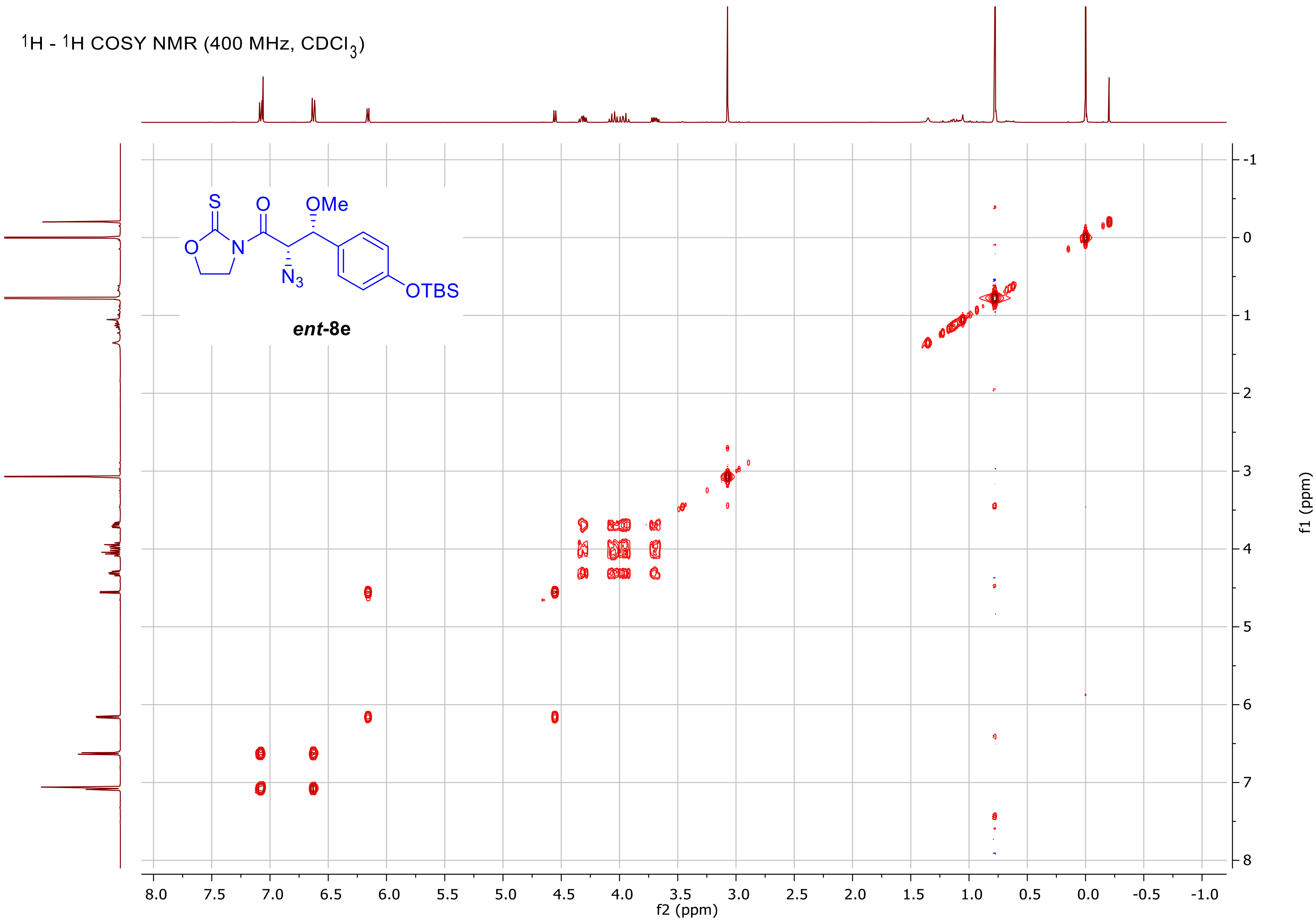

***ent*-8e**

^1H - ^{13}C HSQC NMR (400 MHz, $CDCl_3$)

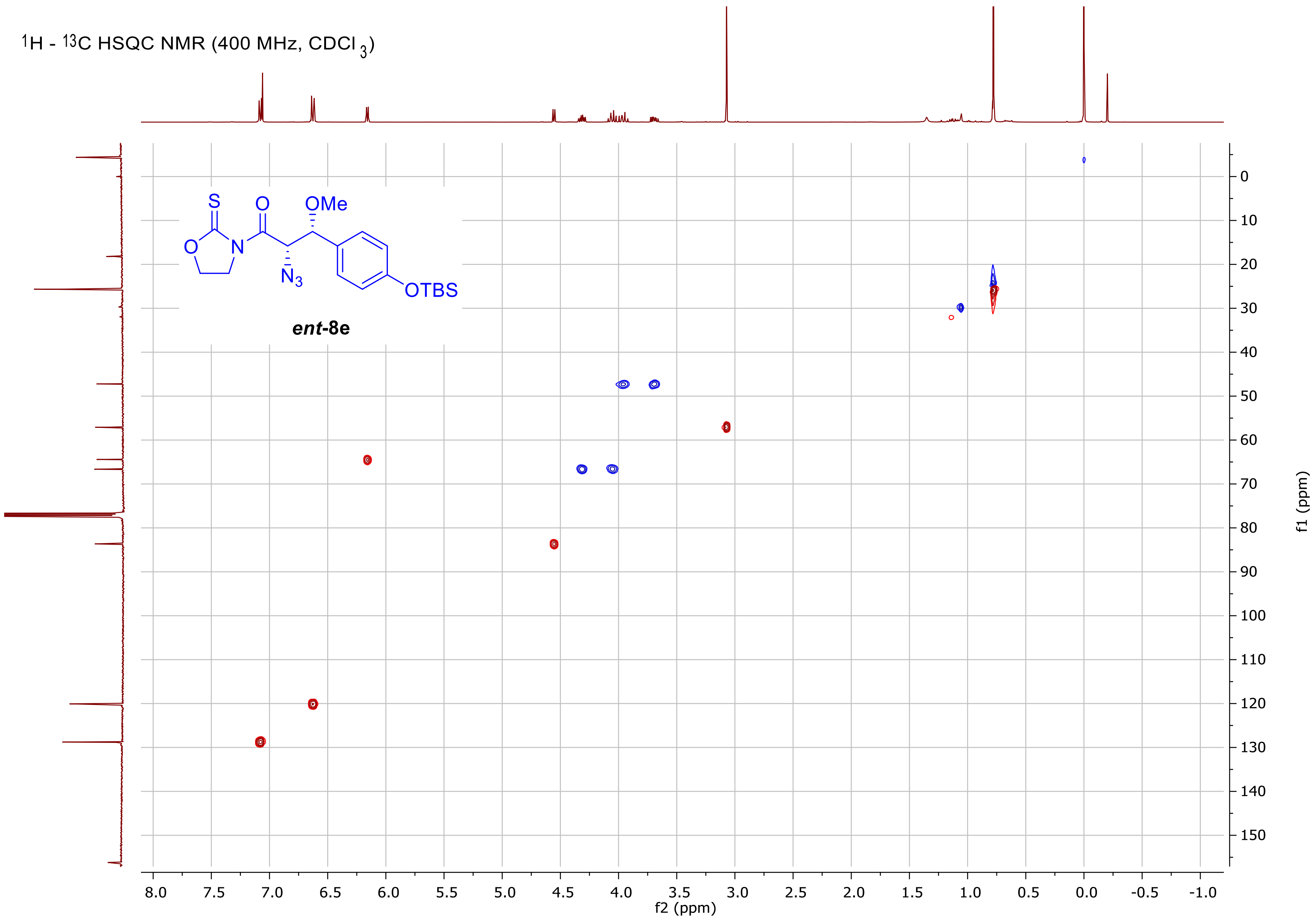

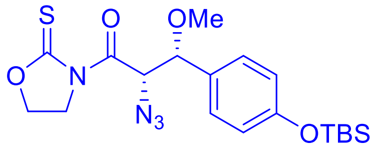

***ent*-8e**

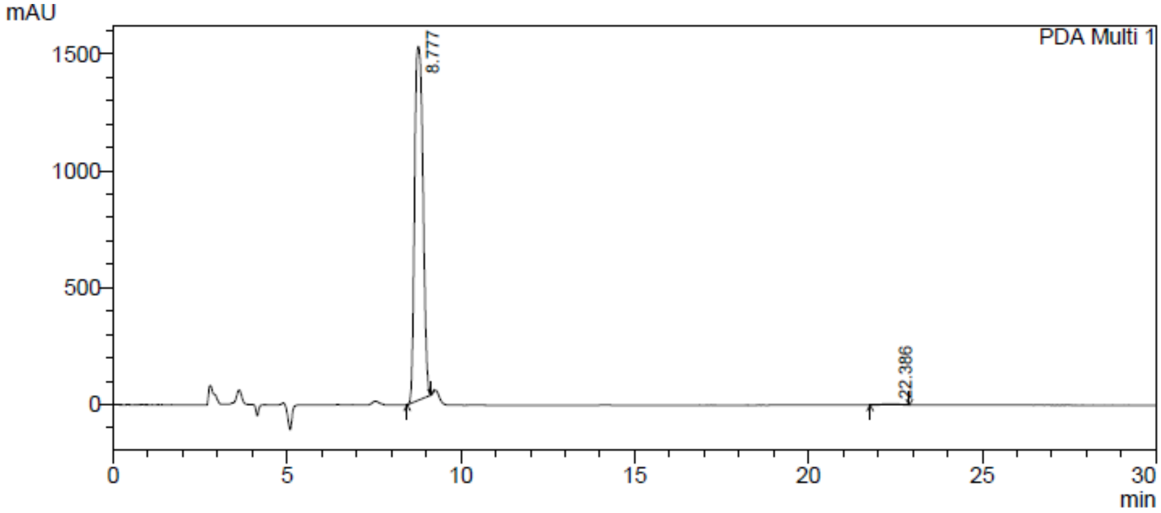

1 PDA Multi 1/254nm 4nm

PeakTable

PDA Ch1 254nm 4nm

Peak#	Ret. Time	Area	Height	Area %	Height %
1	8.777	25327400	1512830	99.255	99.645
2	22.386	190038	5391	0.745	0.355
Total		25517437	1518221	100.000	100.000

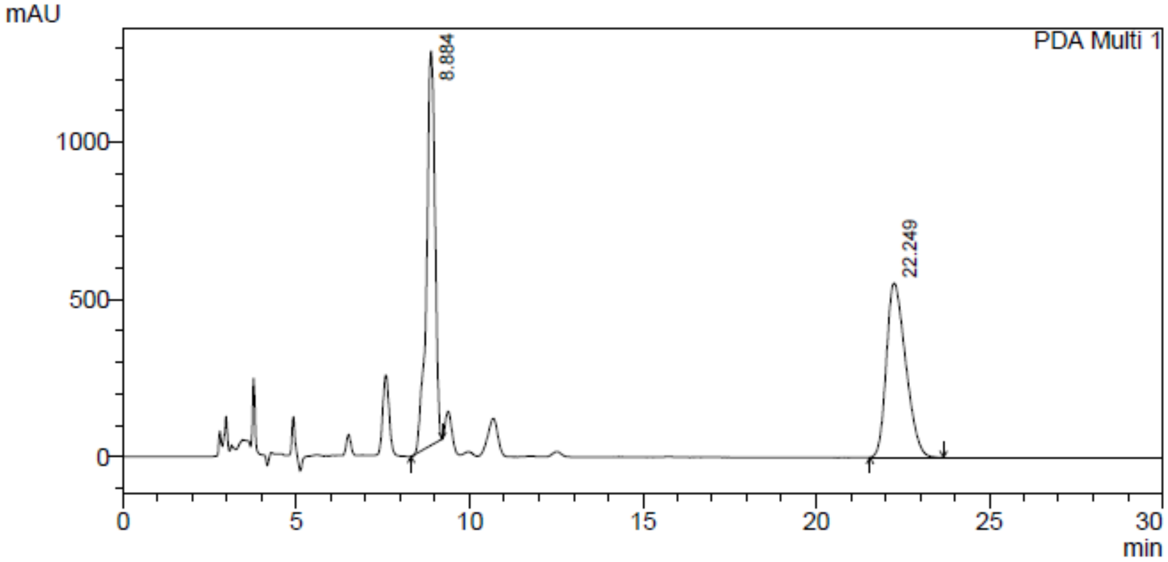

1 PDA Multi 1/254nm 4nm

PeakTable

PDA Ch1 254nm 4nm

Peak#	Ret. Time	Area	Height	Area %	Height %
1	8.884	21280860	1253853	49.734	69.316
2	22.249	21508903	555030	50.266	30.684
Total		42789763	1808883	100.000	100.000

^{1}H NMR (400 MHz, $CDCl_3$)

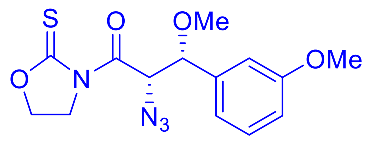

***ent*-8f**

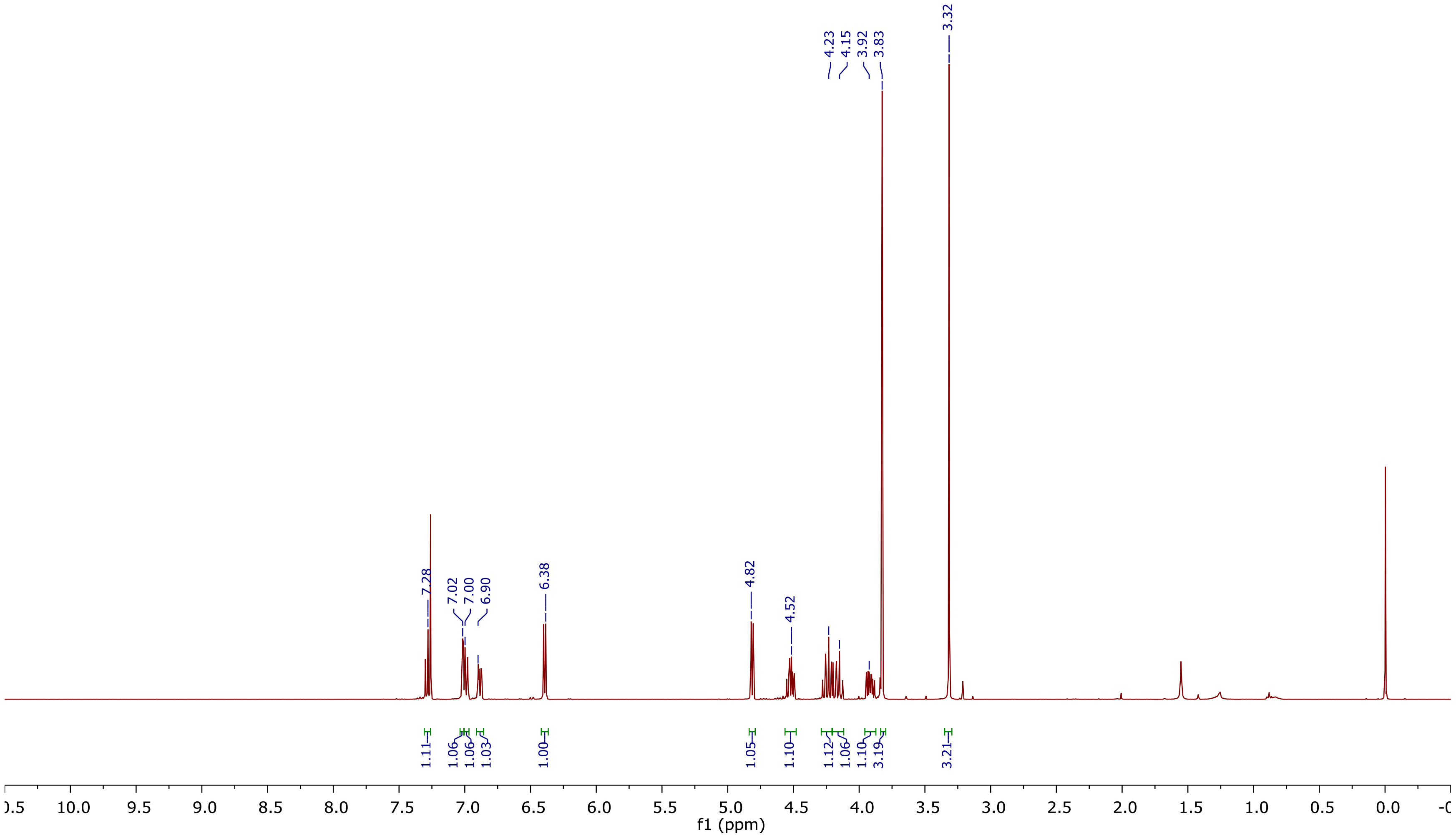

$^{13}C\{^{1}H\}$ NMR (100.6 MHz, $CDCl_3$)

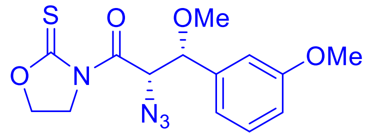

***ent*-8f**

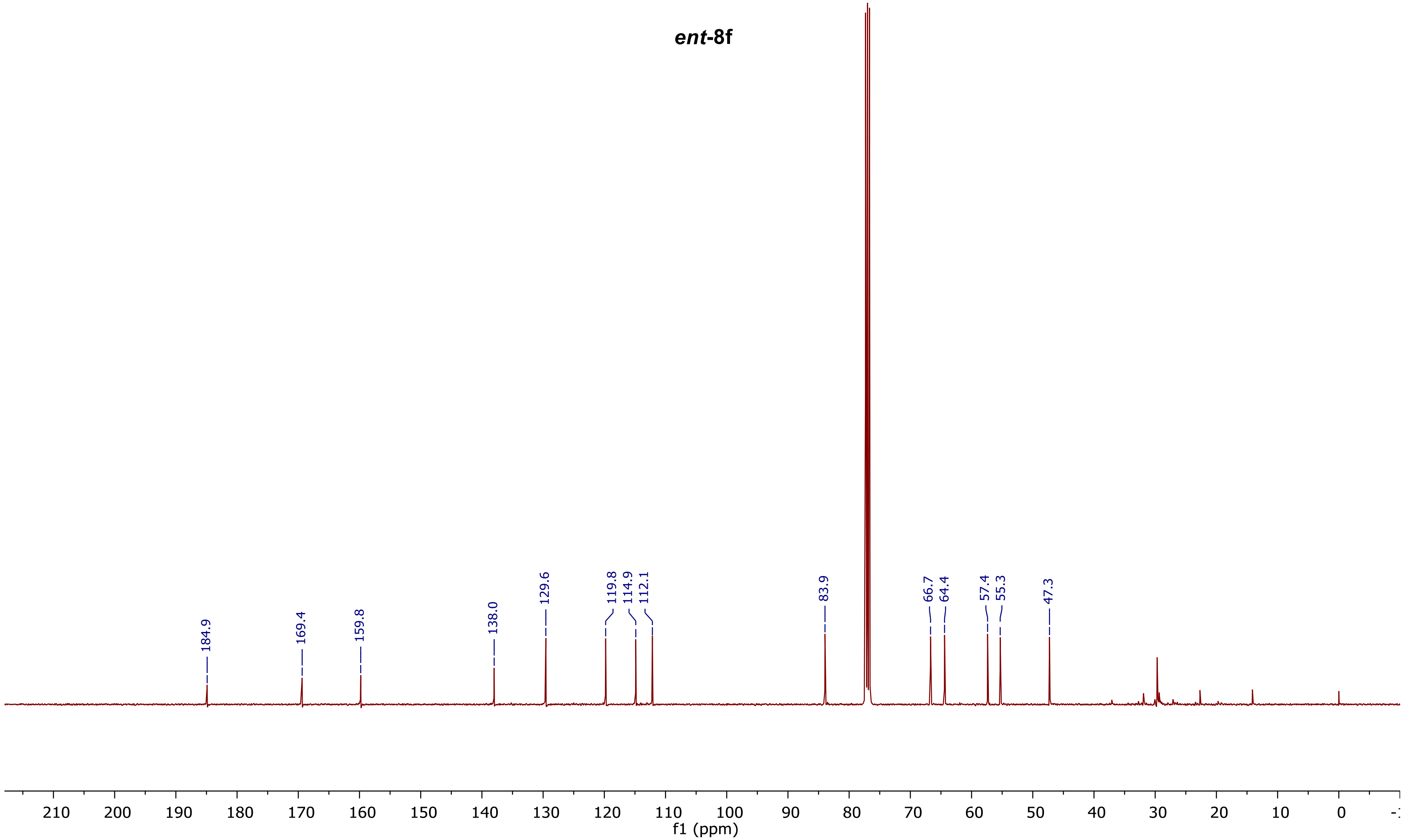

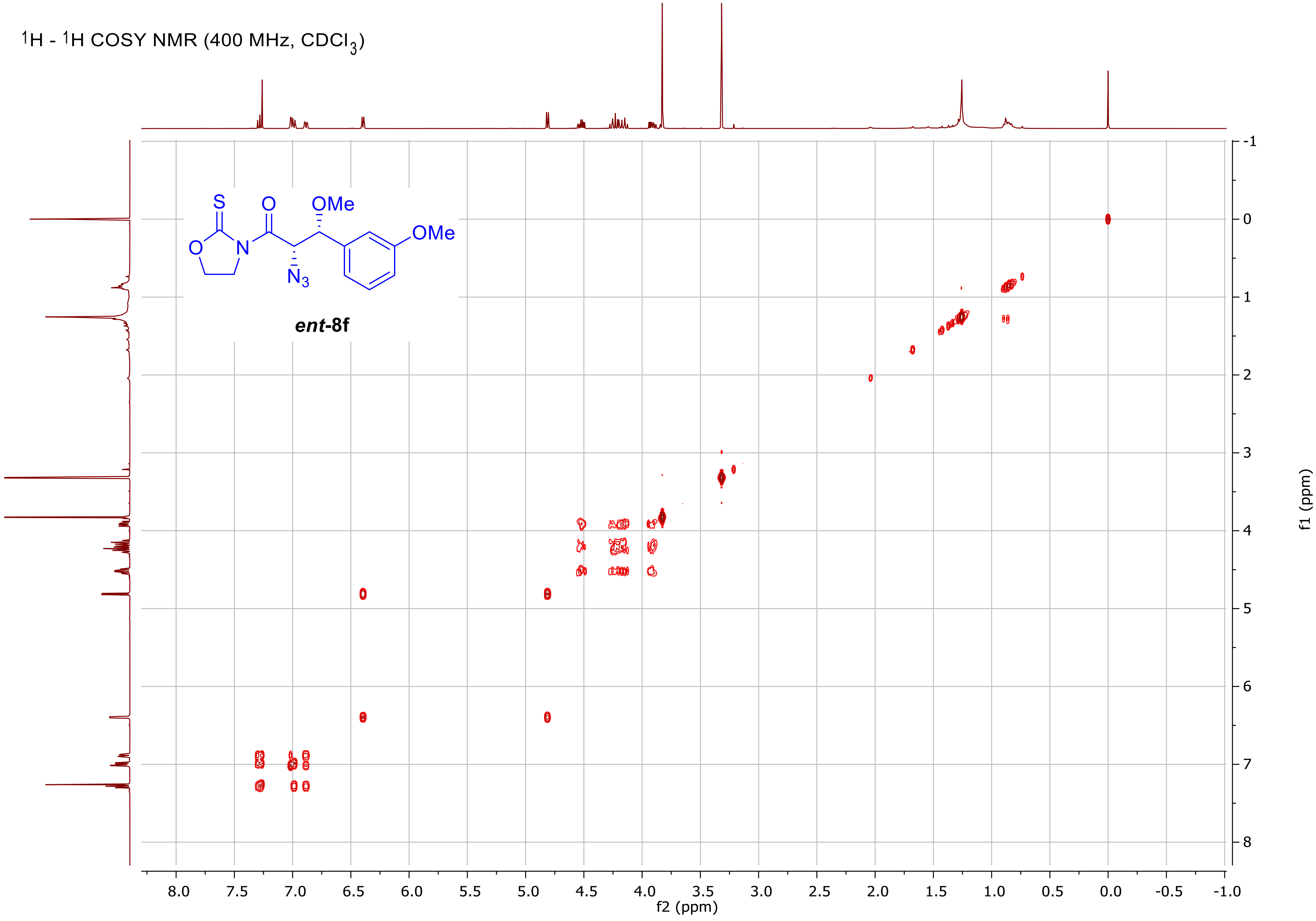

1H - 1H COSY NMR (400 MHz, CDCl3)
S
O
OMe
OMe
O
N
N3
ent-8f
f2 (ppm)
f1 (ppm)

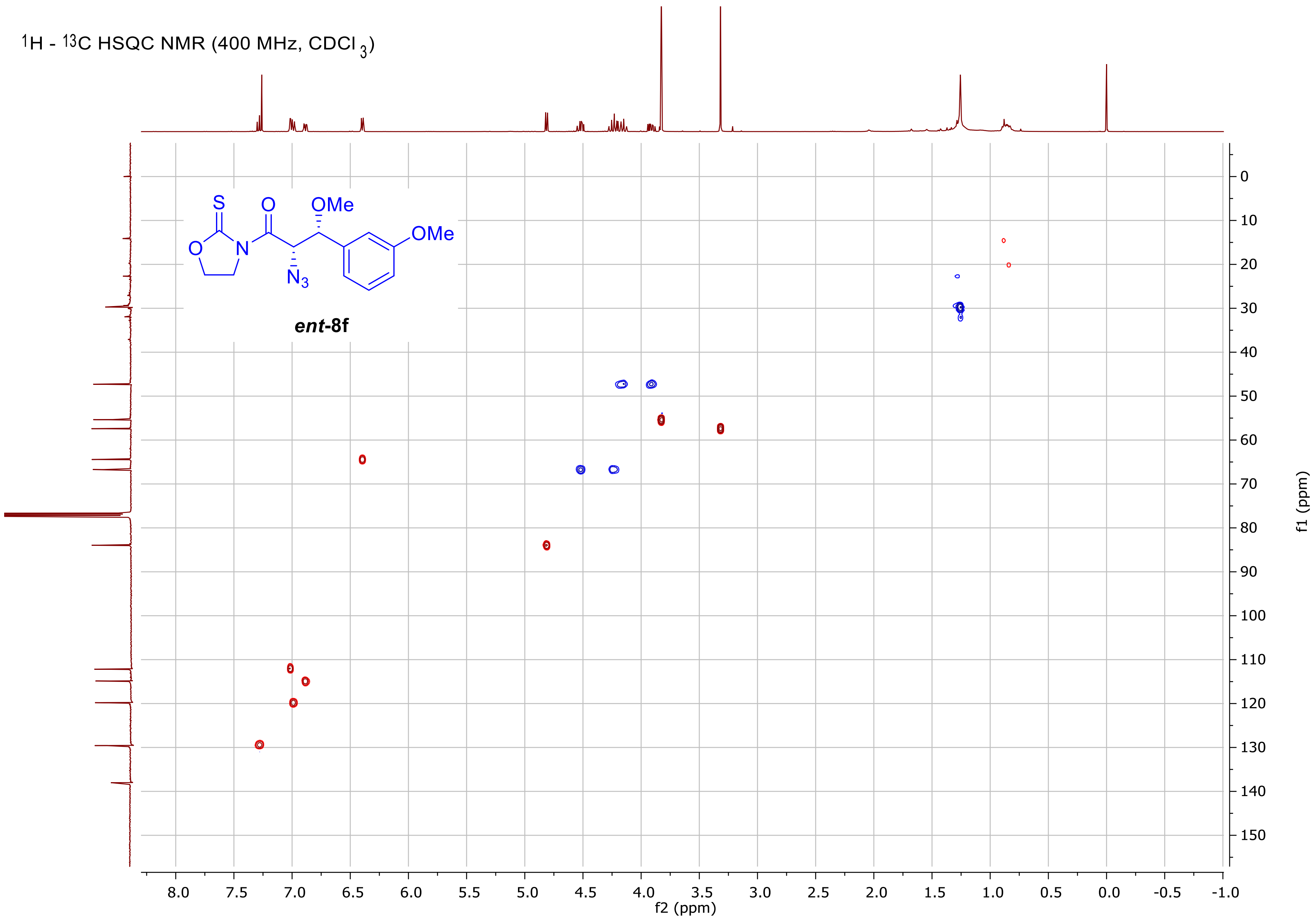
1H - 13C HSQC NMR (400 MHz, CDCl 3)
S
O
OMe
O
N
N3
OMe
ent-8f
f2 (ppm)
f1 (ppm)

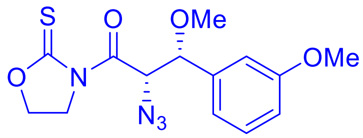

***ent*-8f**

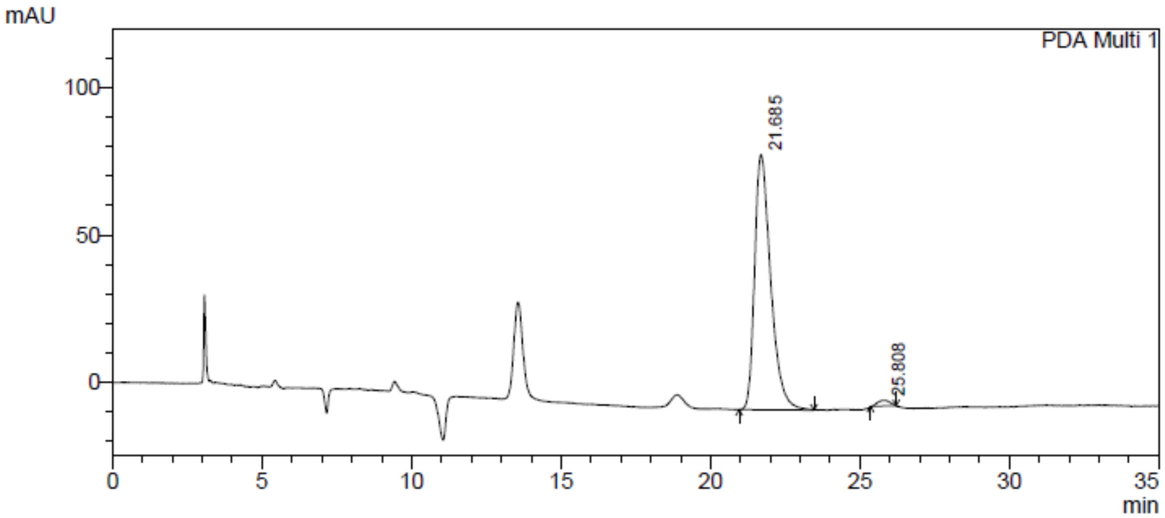

1 PDA Multi 1/254nm 4nm

PeakTable

PDA Ch1 254nm 4nm

Peak#	Ret. Time	Area	Height	Area %	Height %
1	21.685	3231019	86459	98.331	97.786
2	25.808	54855	1958	1.669	2.214
Total		3285874	88417	100.000	100.000

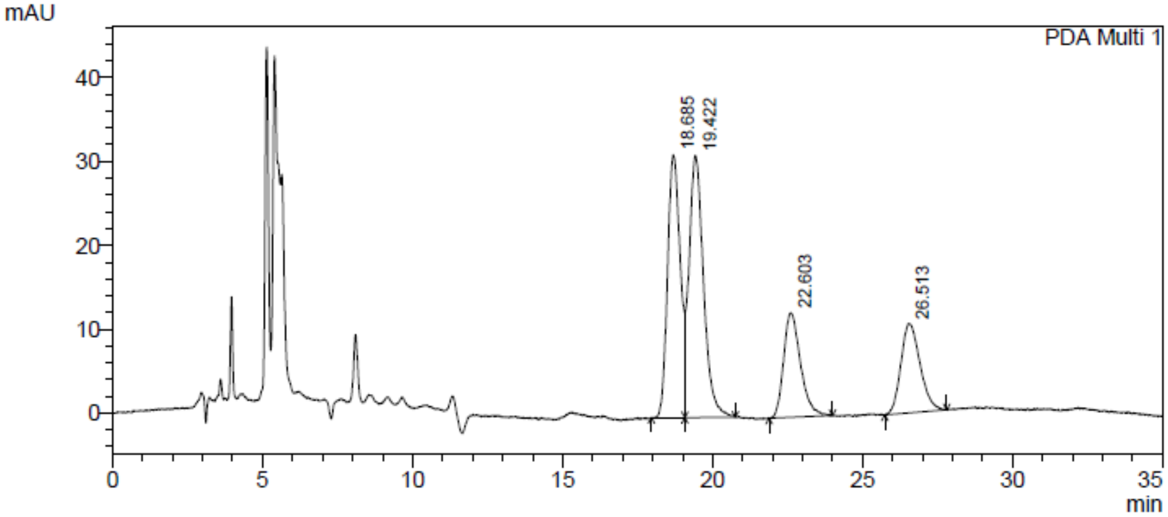

1 PDA Multi 1/254nm 4nm

PeakTable

PDA Ch1 254nm 4nm

Peak#	Ret. Time	Area	Height	Area %	Height %
1	18.685	916048	31342	31.208	36.570
2	19.422	1055197	31245	35.949	36.456
3	22.603	489227	12461	16.667	14.539
4	26.513	474781	10657	16.175	12.435
Total		2935254	85705	100.000	100.000

^{1}H NMR (400 MHz, $CDCl_3$)

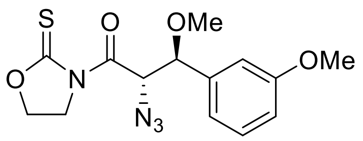

***ent*-6f**

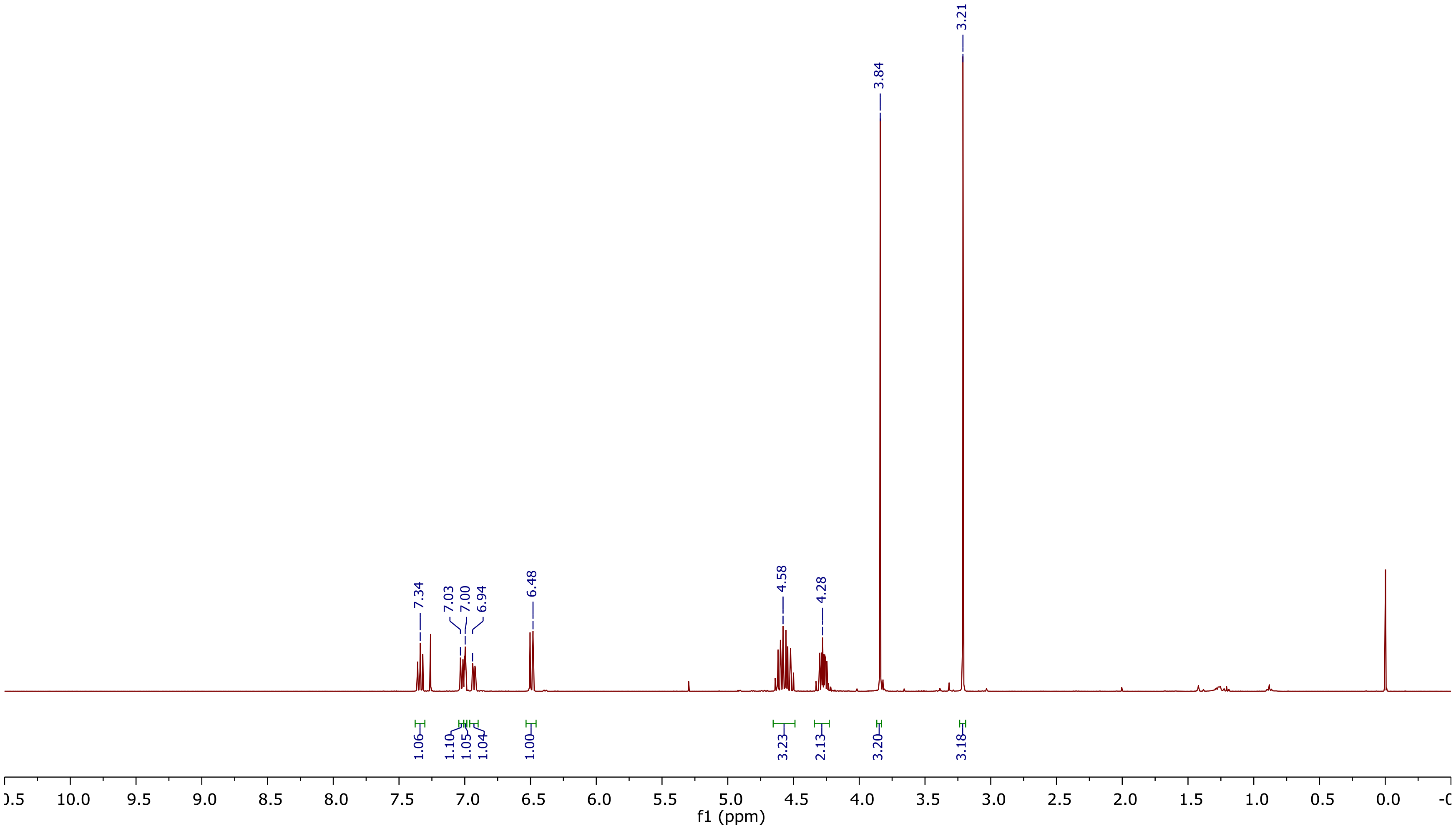

$^{13}C\{^1H\}$ NMR (100.6 MHz, $CDCl_3$)

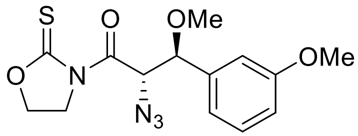

***ent*-6f**

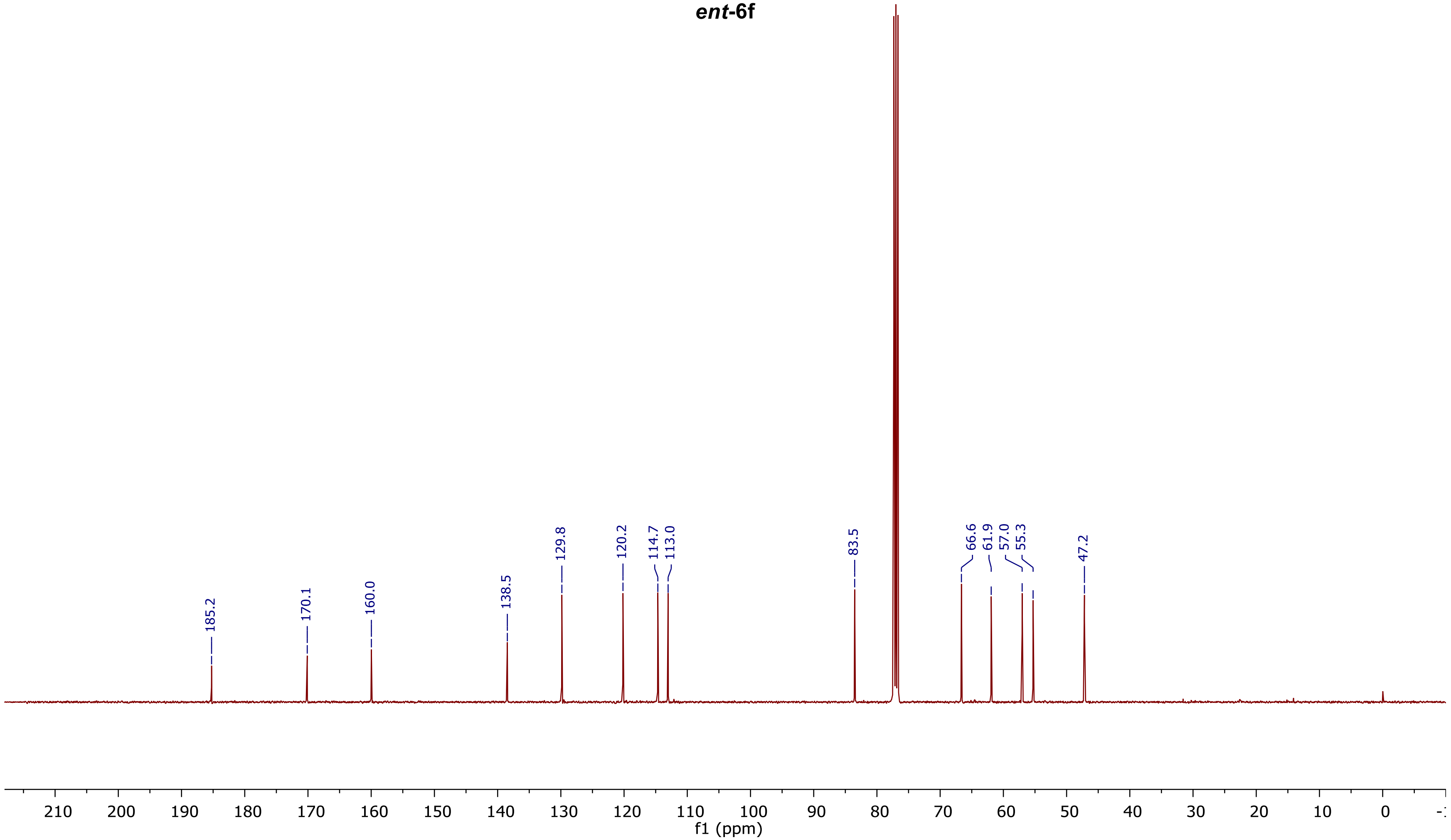

^{1}H - ^{1}H COSY NMR (400 MHz, $CDCl_3$)

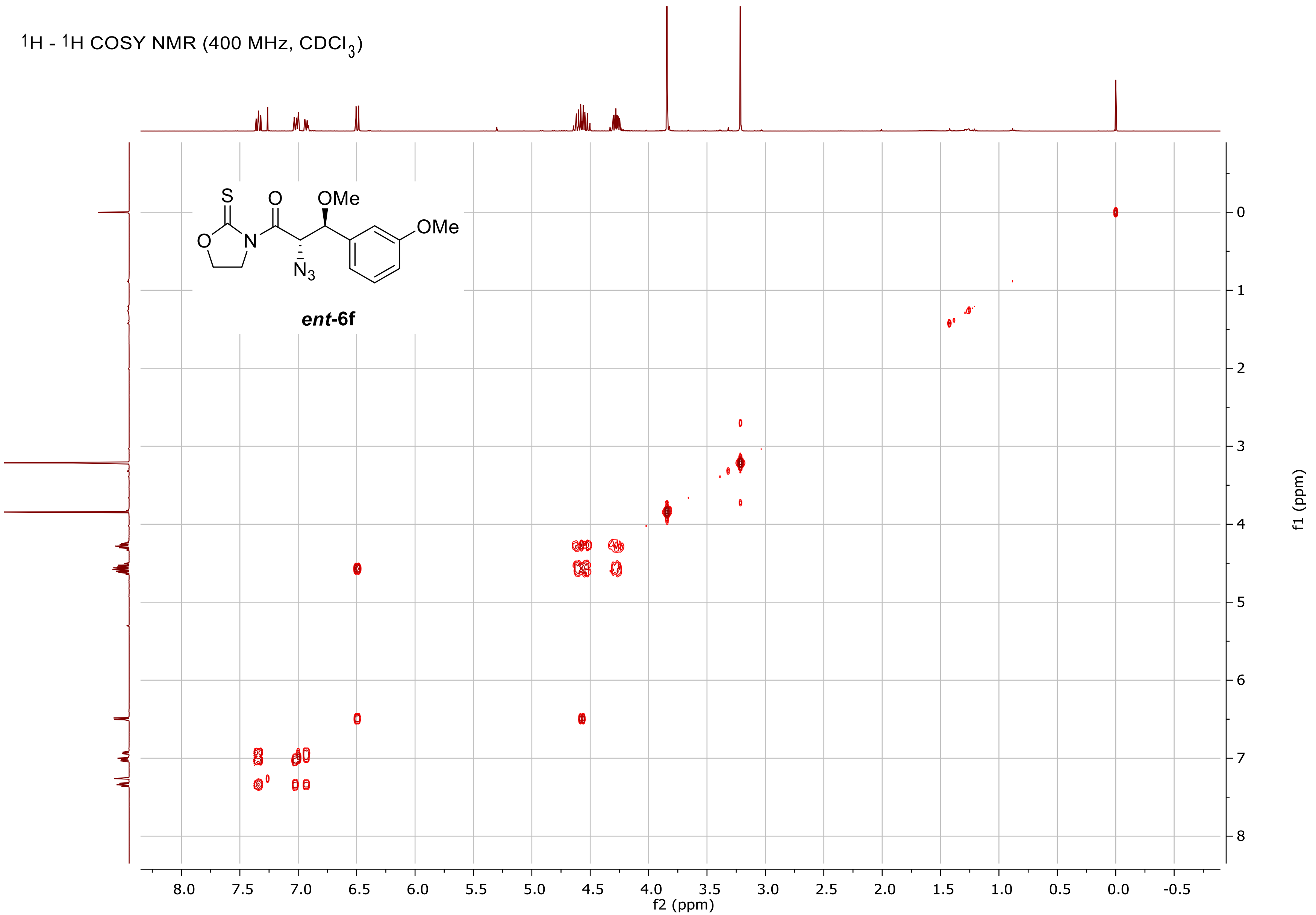

***ent*-6f**

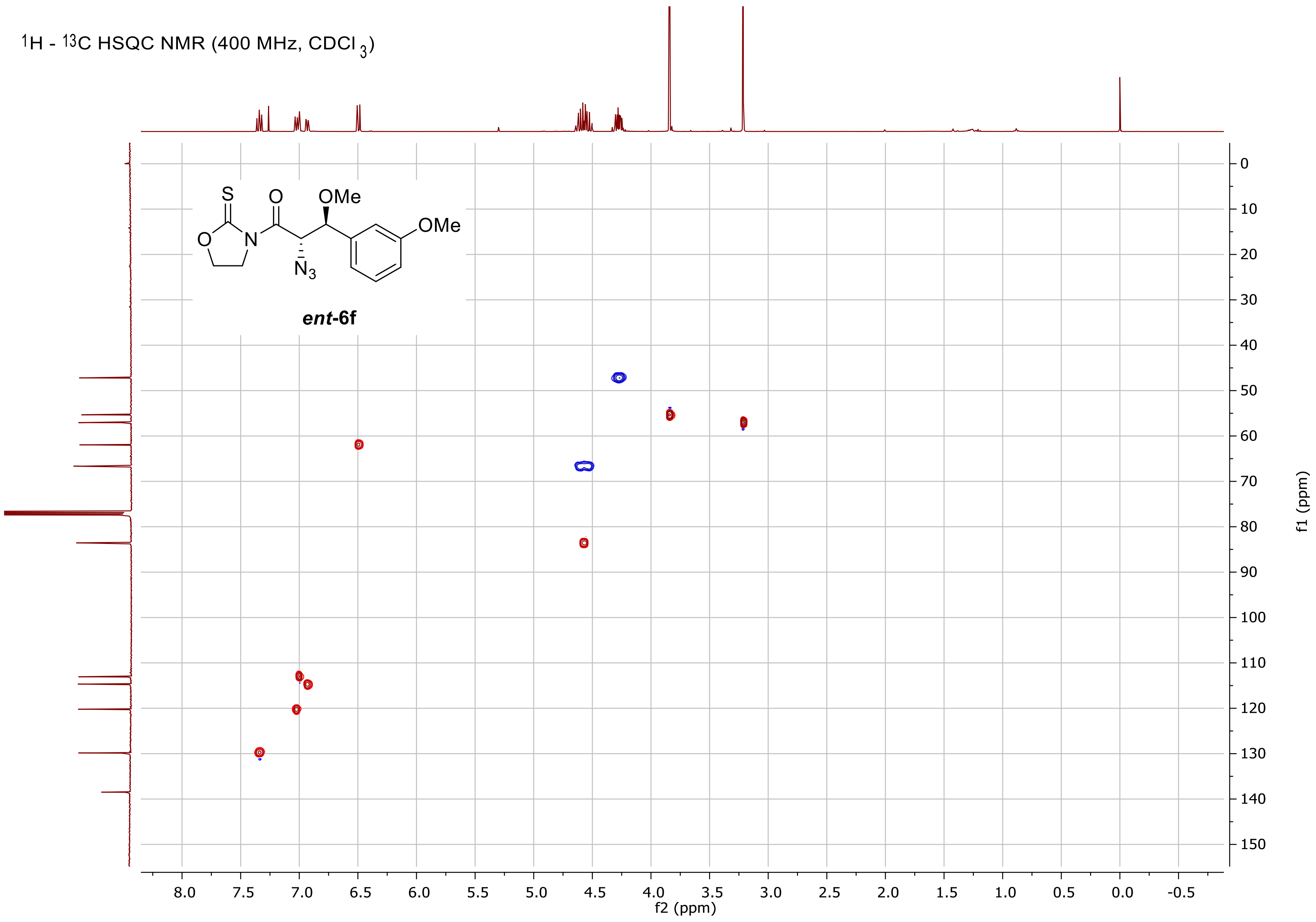

^1H - ^{13}C HSQC NMR (400 MHz, $CDCl_3$)
S
O
OMe
OMe
O
N
N_3
ent-6f
f2 (ppm)
f1 (ppm)

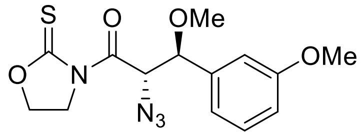

***ent*-6f**

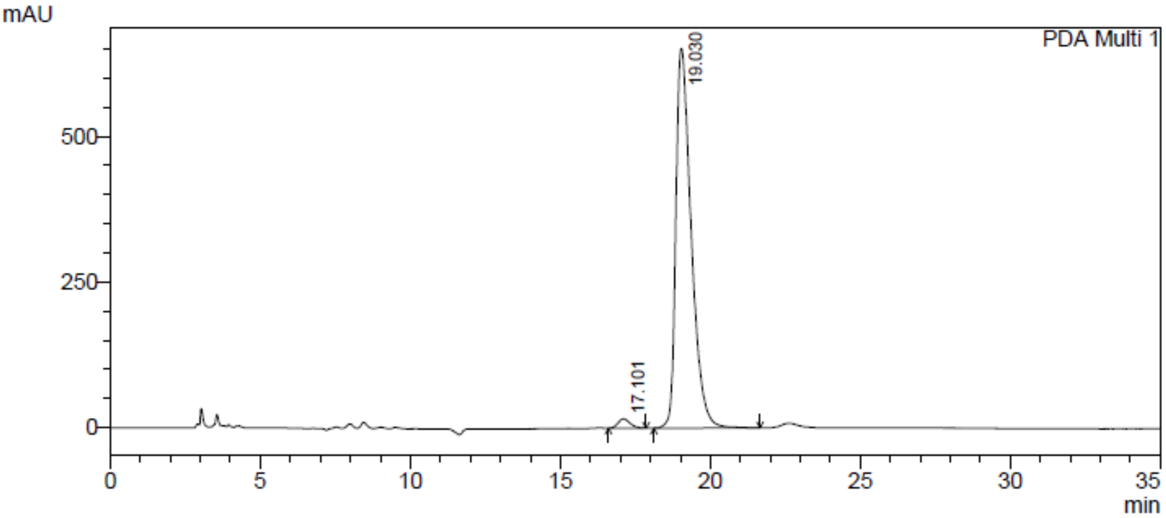

1 PDA Multi 1/254nm 4nm

PeakTable

PDA Ch1 254nm 4nm

Peak#	Ret. Time	Area	Height	Area %	Height %
1	17.101	444822	15964	1.880	2.392
2	19.030	23209753	651324	98.120	97.608
Total		23654575	667288	100.000	100.000

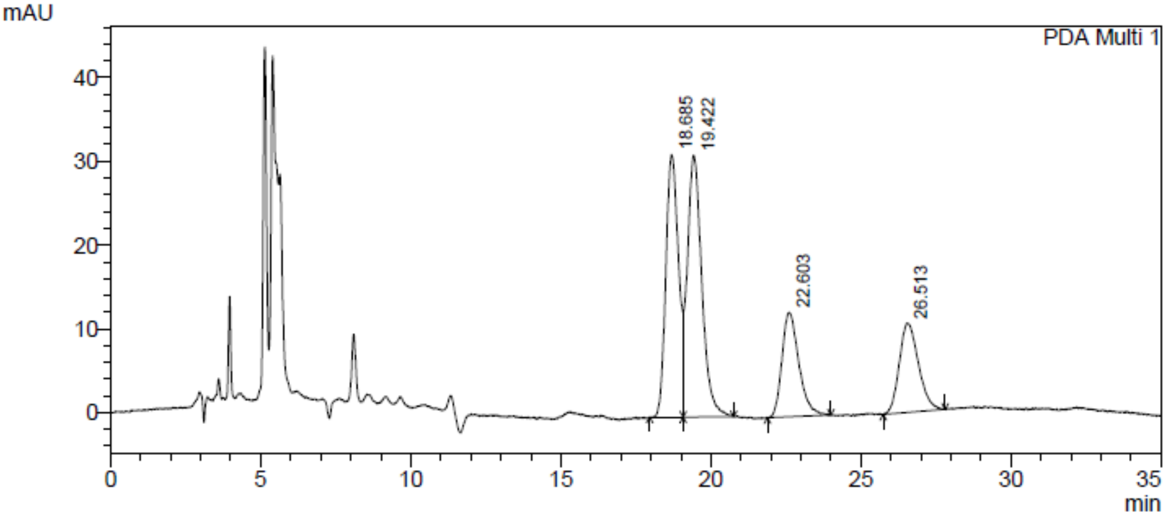

1 PDA Multi 1/254nm 4nm

PeakTable

PDA Ch1 254nm 4nm

Peak#	Ret. Time	Area	Height	Area %	Height %
1	18.685	916048	31342	31.208	36.570
2	19.422	1055197	31245	35.949	36.456
3	22.603	489227	12461	16.667	14.539
4	26.513	474781	10657	16.175	12.435
Total		2935254	85705	100.000	100.000

^1H NMR (400 MHz, $CDCl_3$)

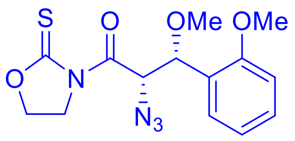

***ent*-8g**

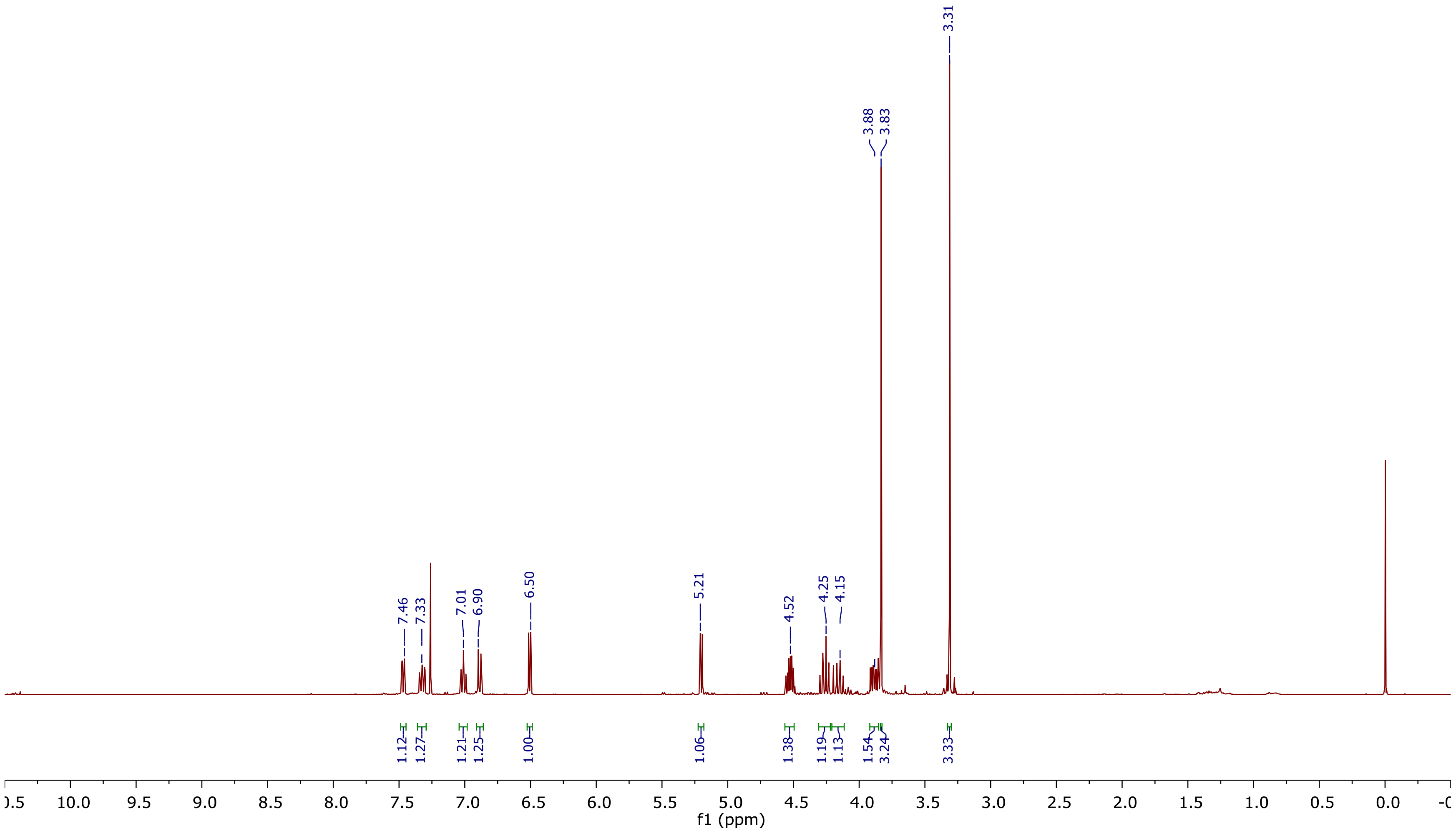

$^{13}C\{^1H\}$ NMR (100.6 MHz, $CDCl_3$)

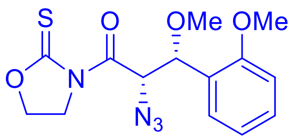

***ent*-8g**

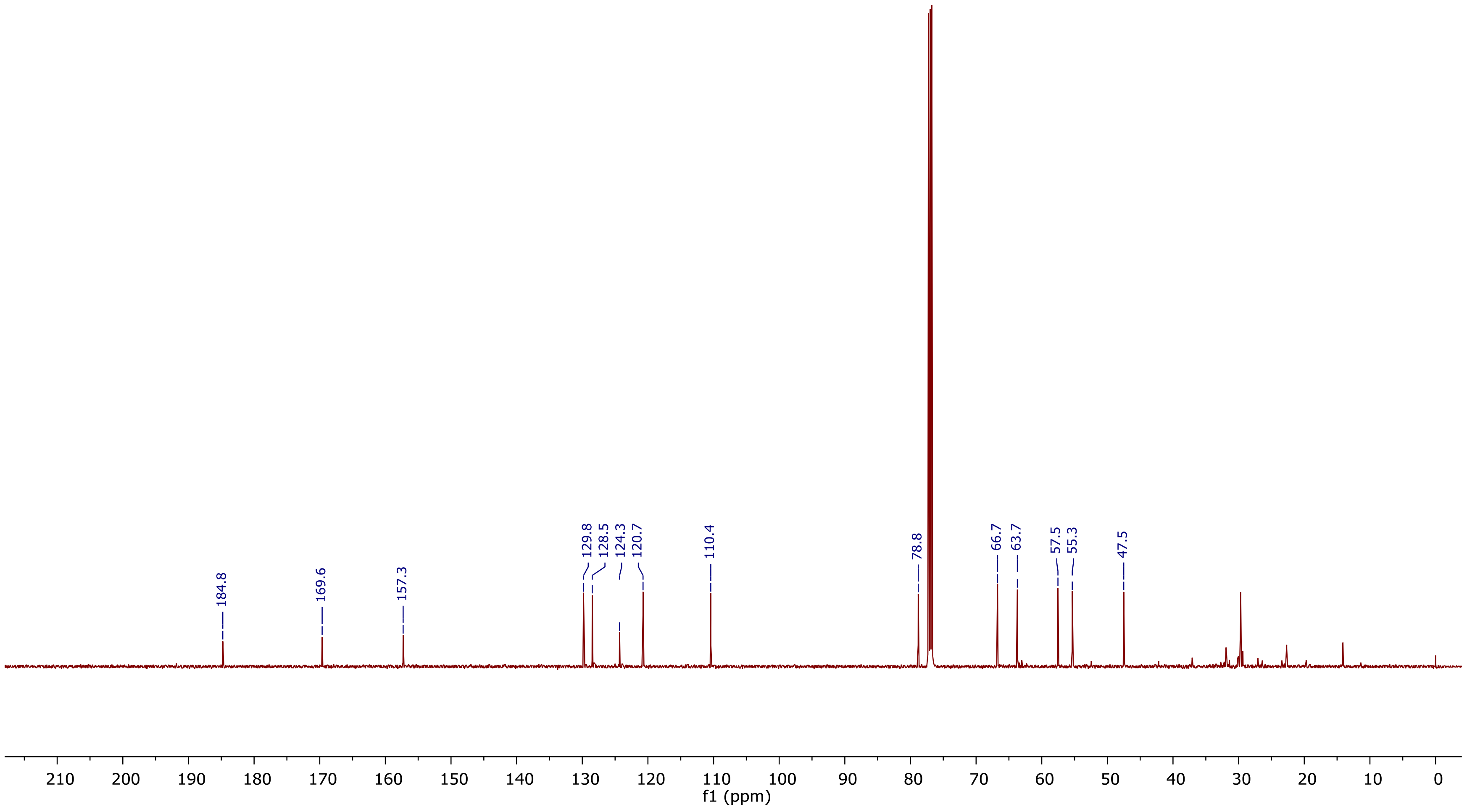

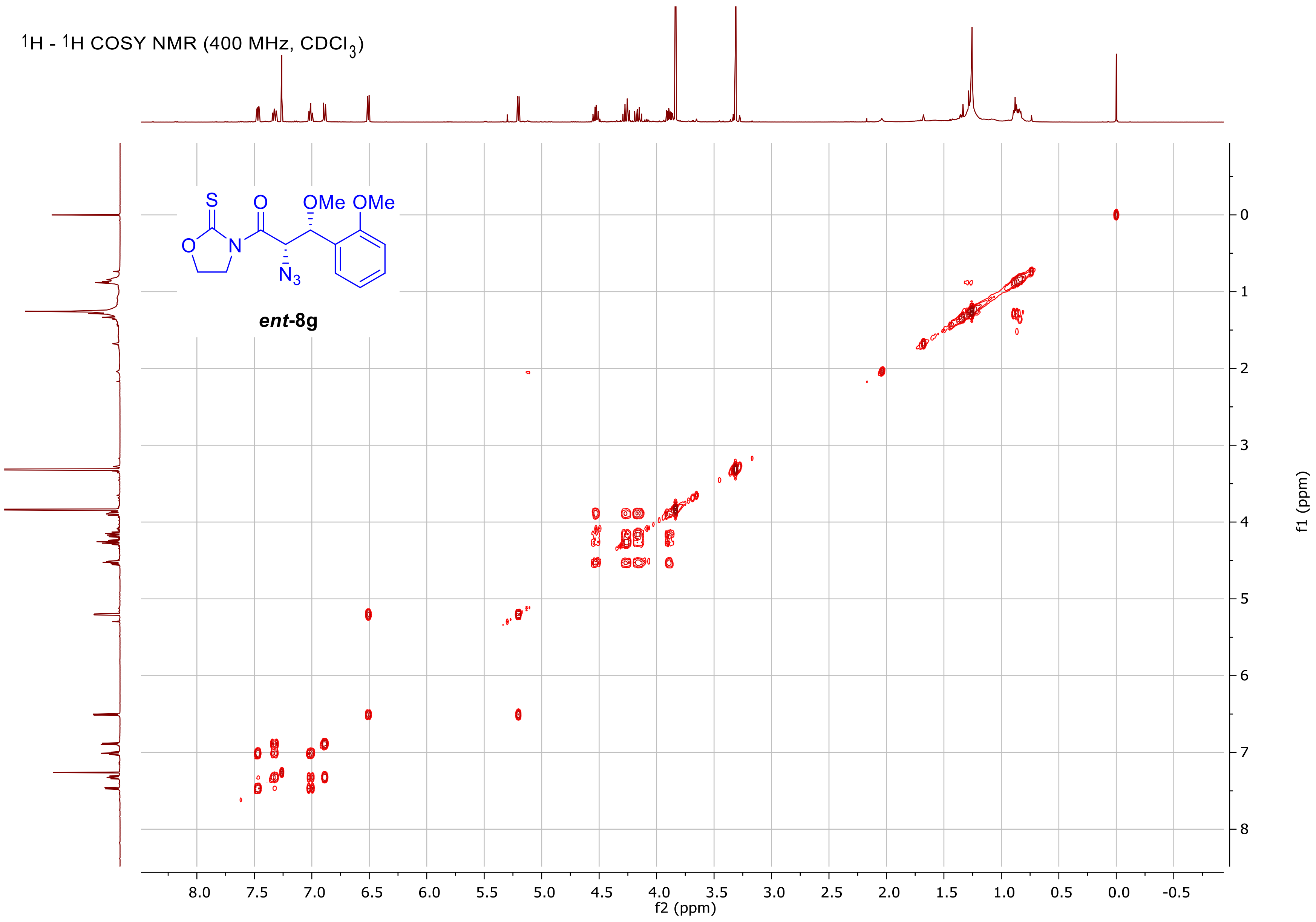

1H - 1H COSY NMR (400 MHz, CDCl3)
S
O
O
N
OMe
OMe
N3
ent-8g
8.0
7.5
7.0
6.5
6.0
5.5
5.0
4.5
4.0
3.5
3.0
2.5
2.0
1.5
1.0
0.5
0.0
-0.5
f2 (ppm)
0
1
2
3
4
5
6
7
8
f1 (ppm)

^{1}H - ^{13}C HSQC NMR (400 MHz, $CDCl_3$)

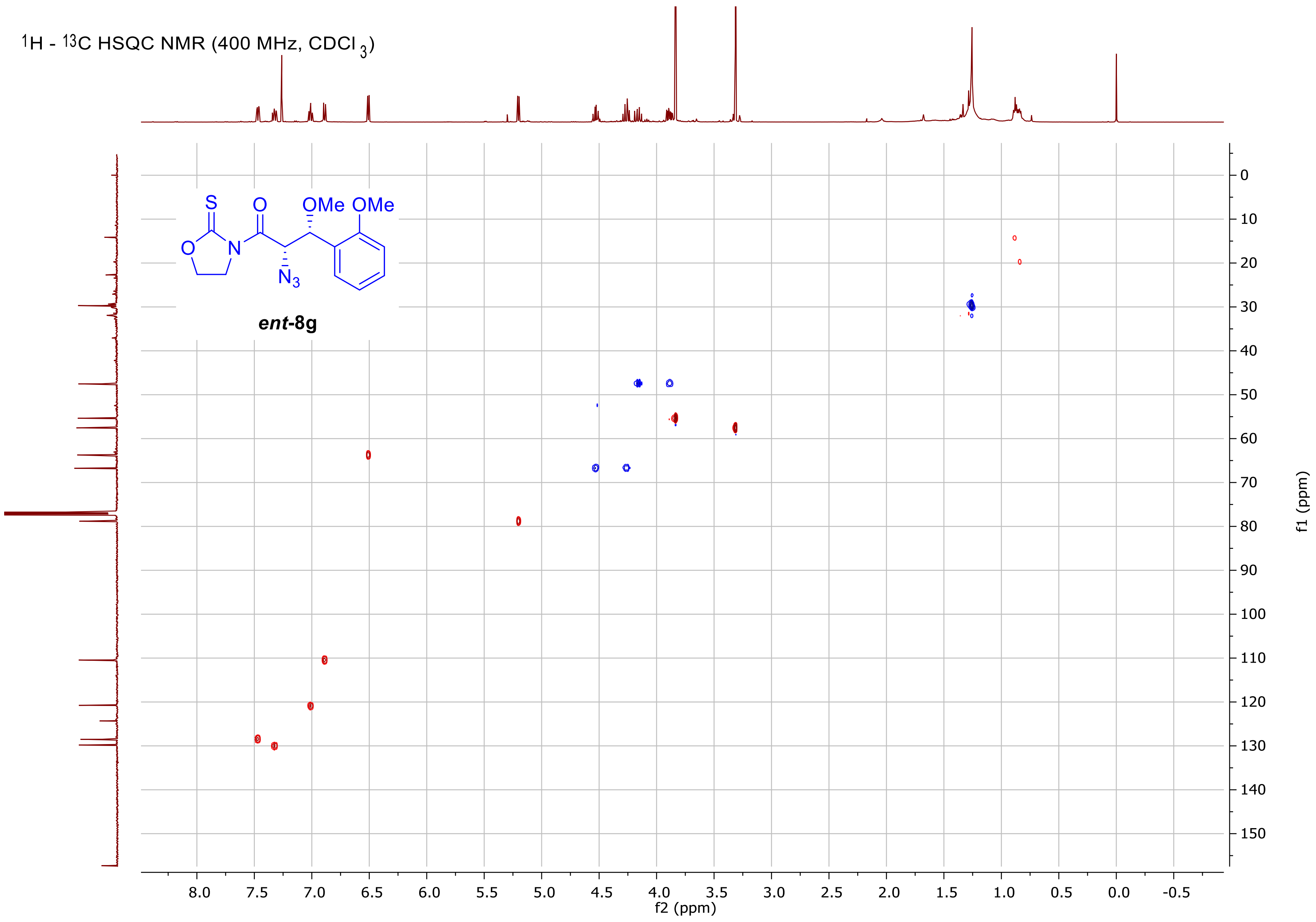

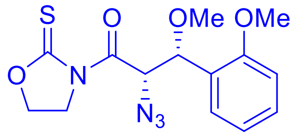

***ent*-8g**

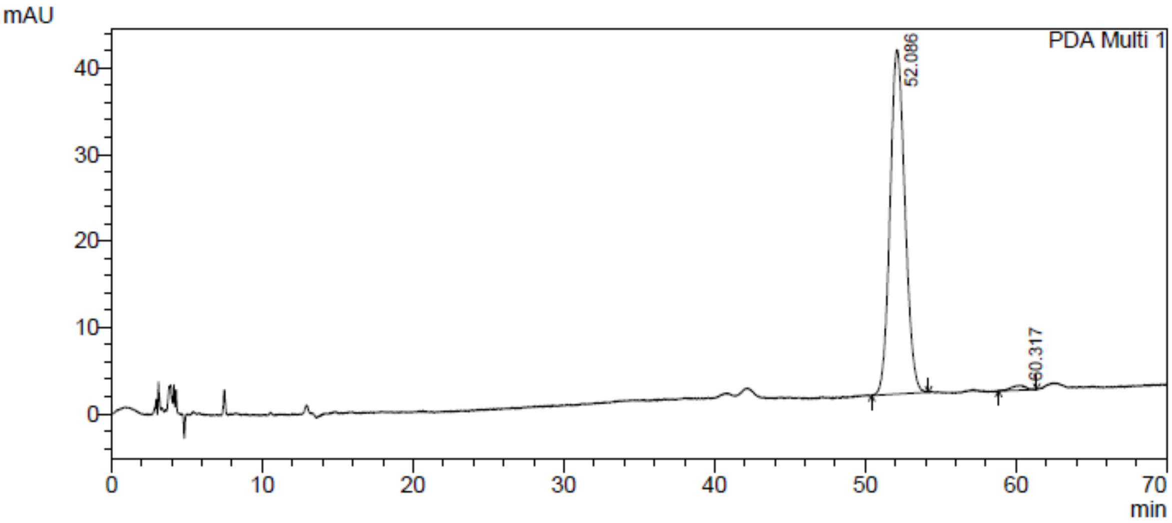

1 PDA Multi 1/254nm 4nm

PeakTable

PDA Ch1 254nm 4nm

Peak#	Ret. Time	Area	Height	Area %	Height %
1	52.086	2744384	39813	98.769	98.600
2	60.317	34194	565	1.231	1.400
Total		2778578	40378	100.000	100.000

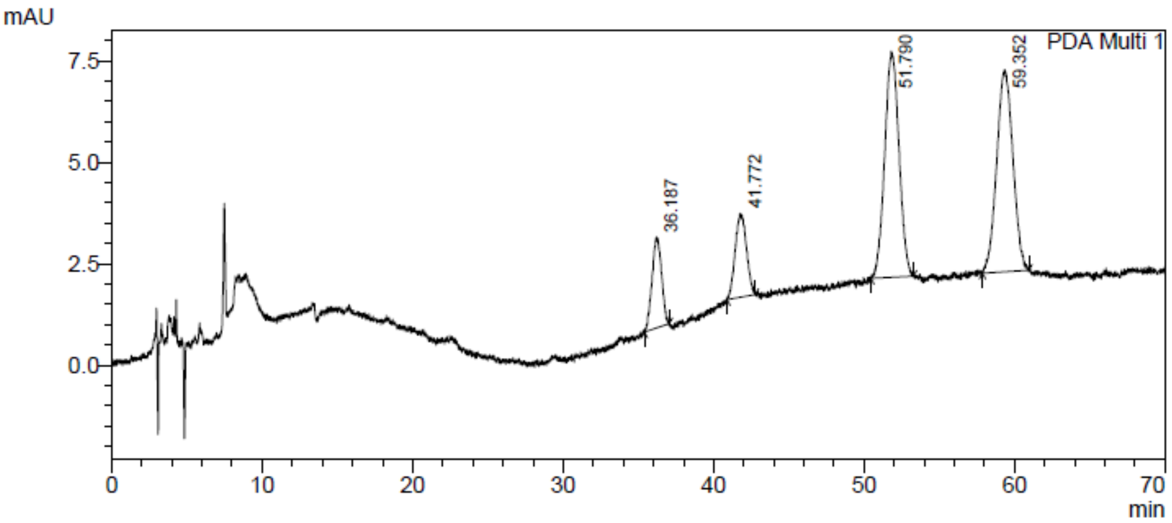

1 PDA Multi 1/254nm 4nm

PeakTable

PDA Ch1 254nm 4nm

Peak#	Ret. Time	Area	Height	Area %	Height %
1	36.187	98041	2241	10.370	15.086
2	41.772	106958	2066	11.313	13.910
3	51.790	368162	5570	38.942	37.504
4	59.352	372244	4975	39.374	33.500
Total		945405	14852	100.000	100.000

^{1}H NMR (400 MHz, $CDCl_3$)

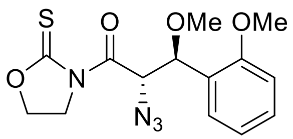

***ent*-6g**

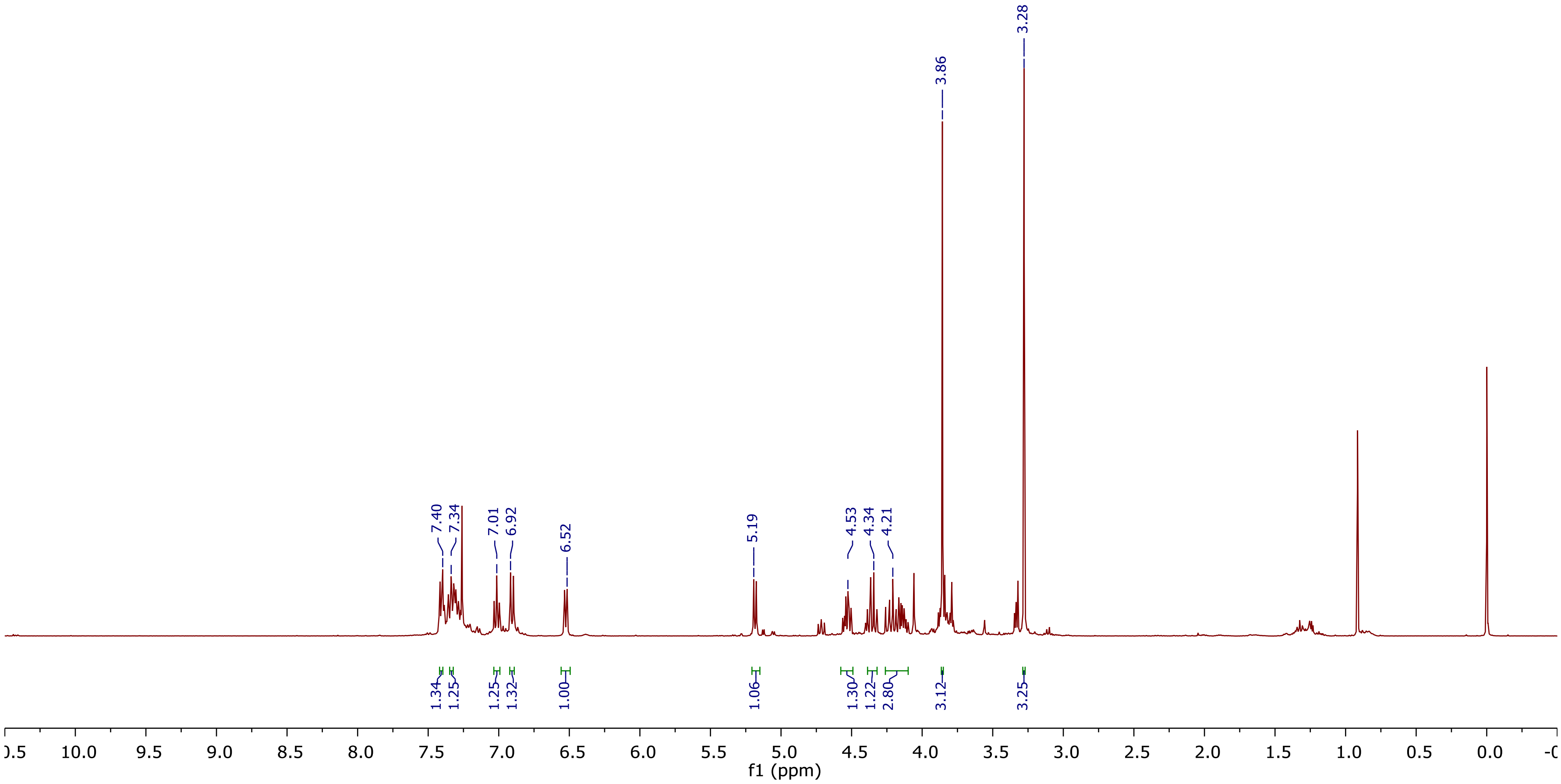

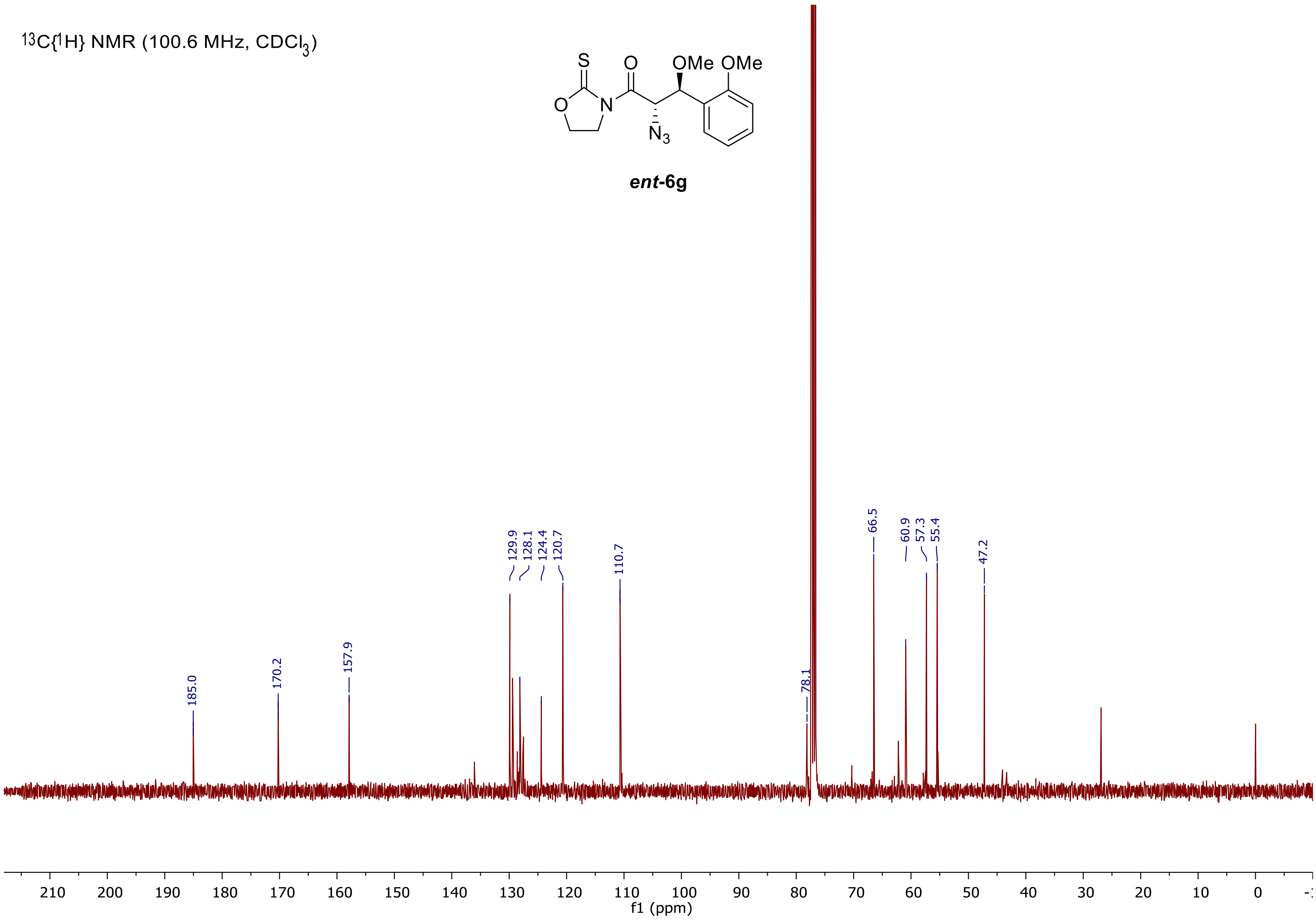

13C{1H} NMR (100.6 MHz, CDCl3)
S
O
OMe
OMe
O
N
N3
ent-6g
185.0
170.2
157.9
129.9
128.1
124.4
120.7
110.7
78.1
66.5
60.9
57.3
55.4
47.2
210
200
190
180
170
160
150
140
130
120
110
100
90
80
70
60
50
40
30
20
10
0
f1 (ppm)

^{1}H - ^{1}H COSY NMR (400 MHz, $CDCl_3$)

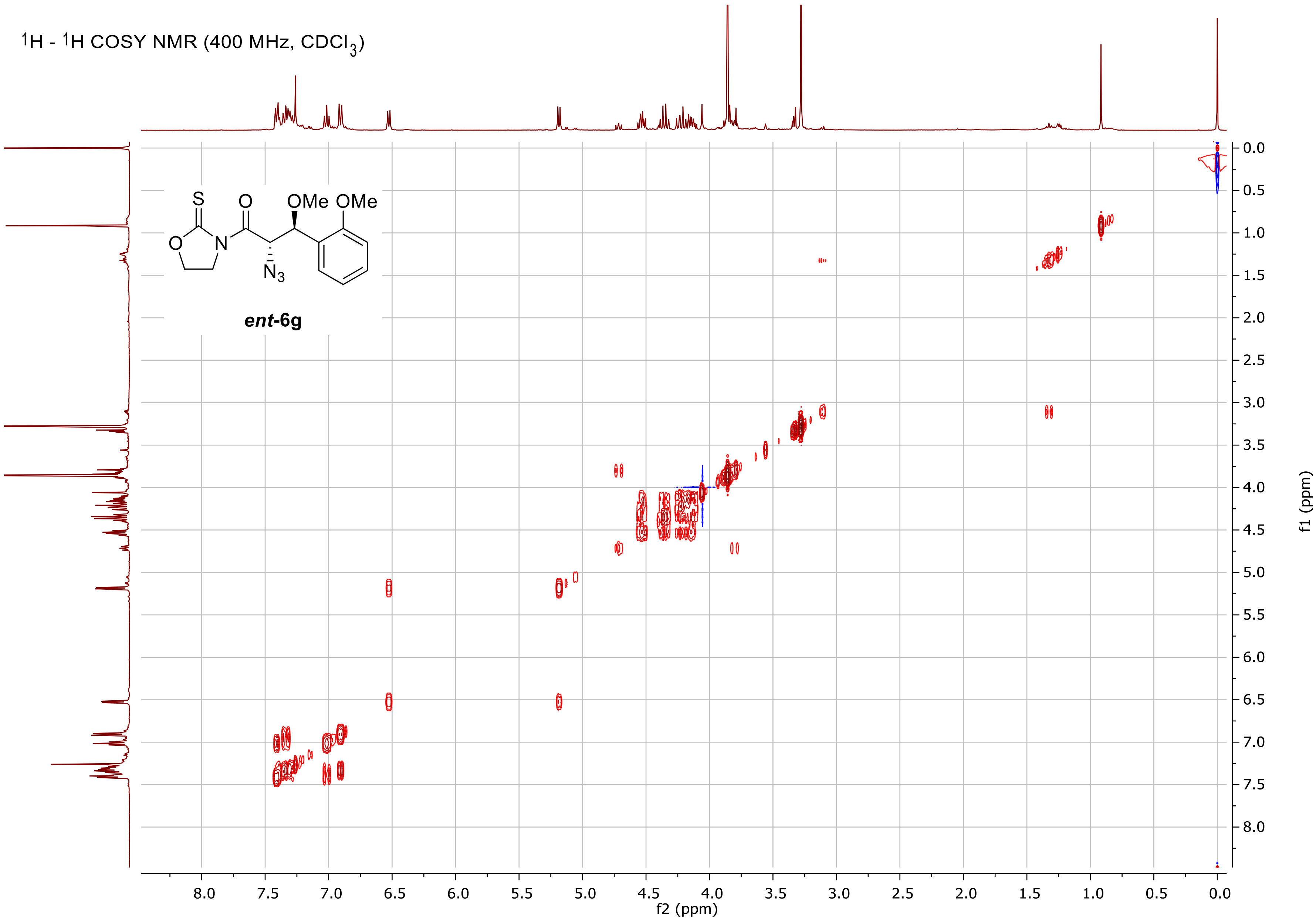

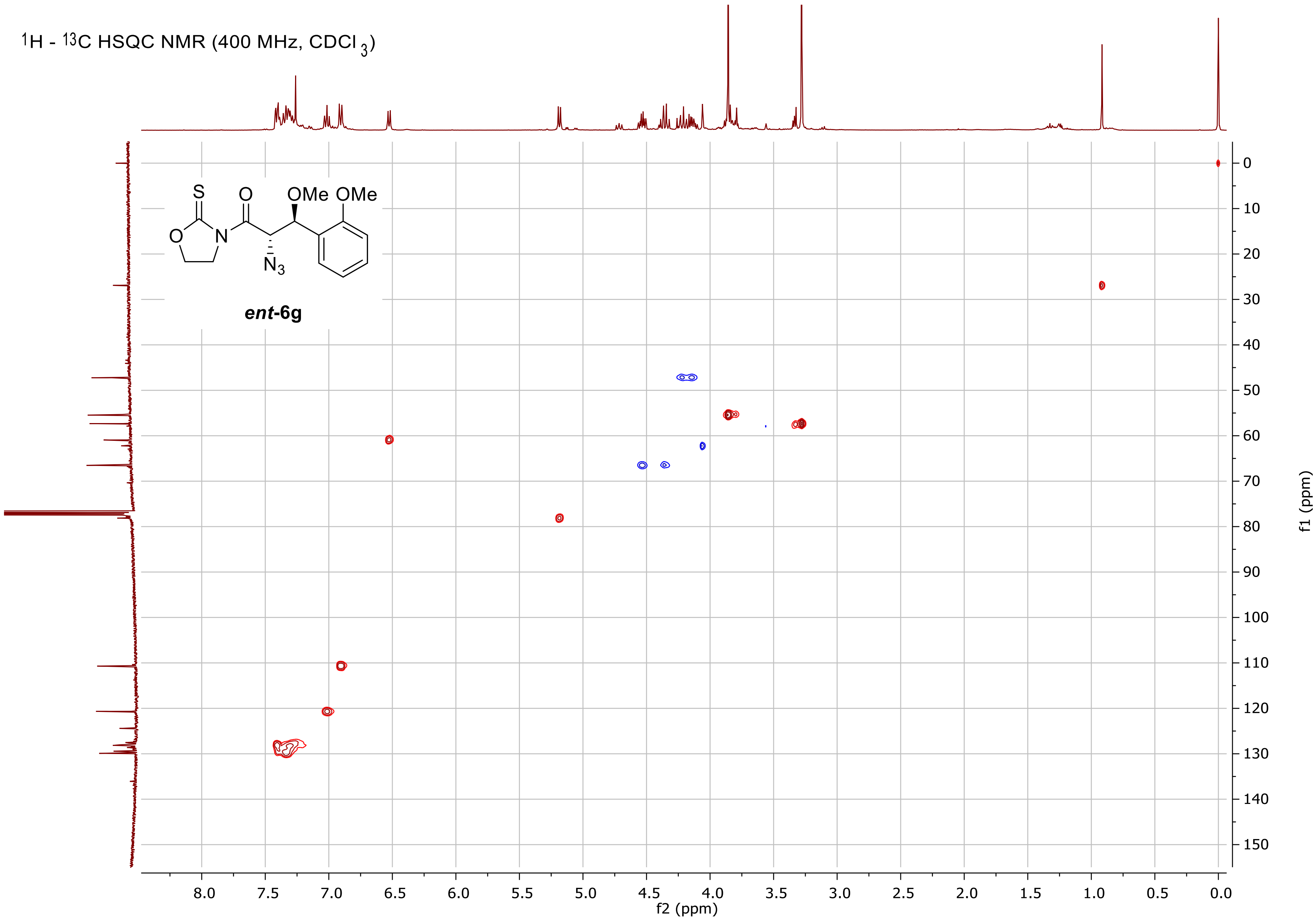

1H - 13C HSQC NMR (400 MHz, CDCl 3)
S
O
OMe OMe
O
N
N3
ent-6g
f2 (ppm)
f1 (ppm)

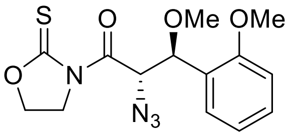

***ent*-6g**

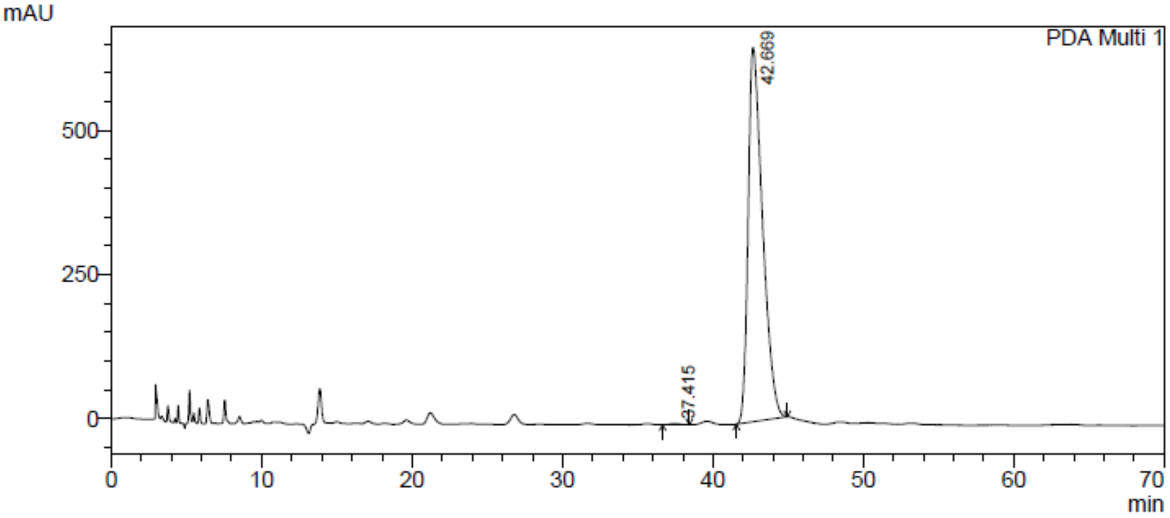

1 PDA Multi 1/254nm 4nm

PeakTable

PDA Ch1 254nm 4nm

Peak#	Ret. Time	Area	Height	Area %	Height %
1	37.415	89908	1846	0.208	0.283
2	42.669	43236726	649417	99.792	99.717
Total		43326634	651263	100.000	100.000

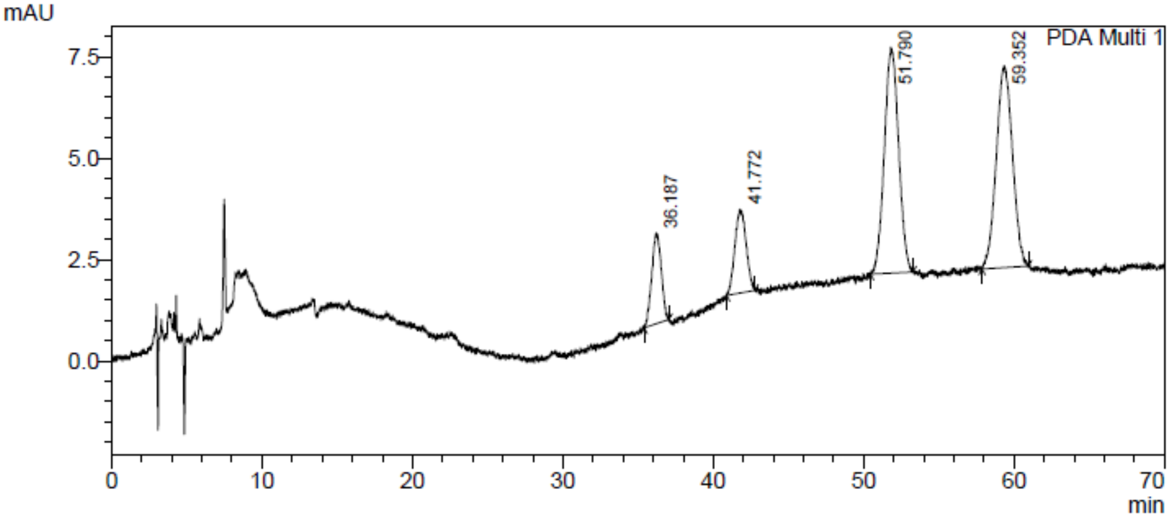

1 PDA Multi 1/254nm 4nm

PeakTable

PDA Ch1 254nm 4nm

Peak#	Ret. Time	Area	Height	Area %	Height %
1	36.187	98041	2241	10.370	15.086
2	41.772	106958	2066	11.313	13.910
3	51.790	368162	5570	38.942	37.504
4	59.352	372244	4975	39.374	33.500
Total		945405	14852	100.000	100.000

^1H NMR (400 MHz, $CDCl_3$)

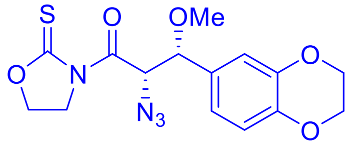

***ent*-8h**

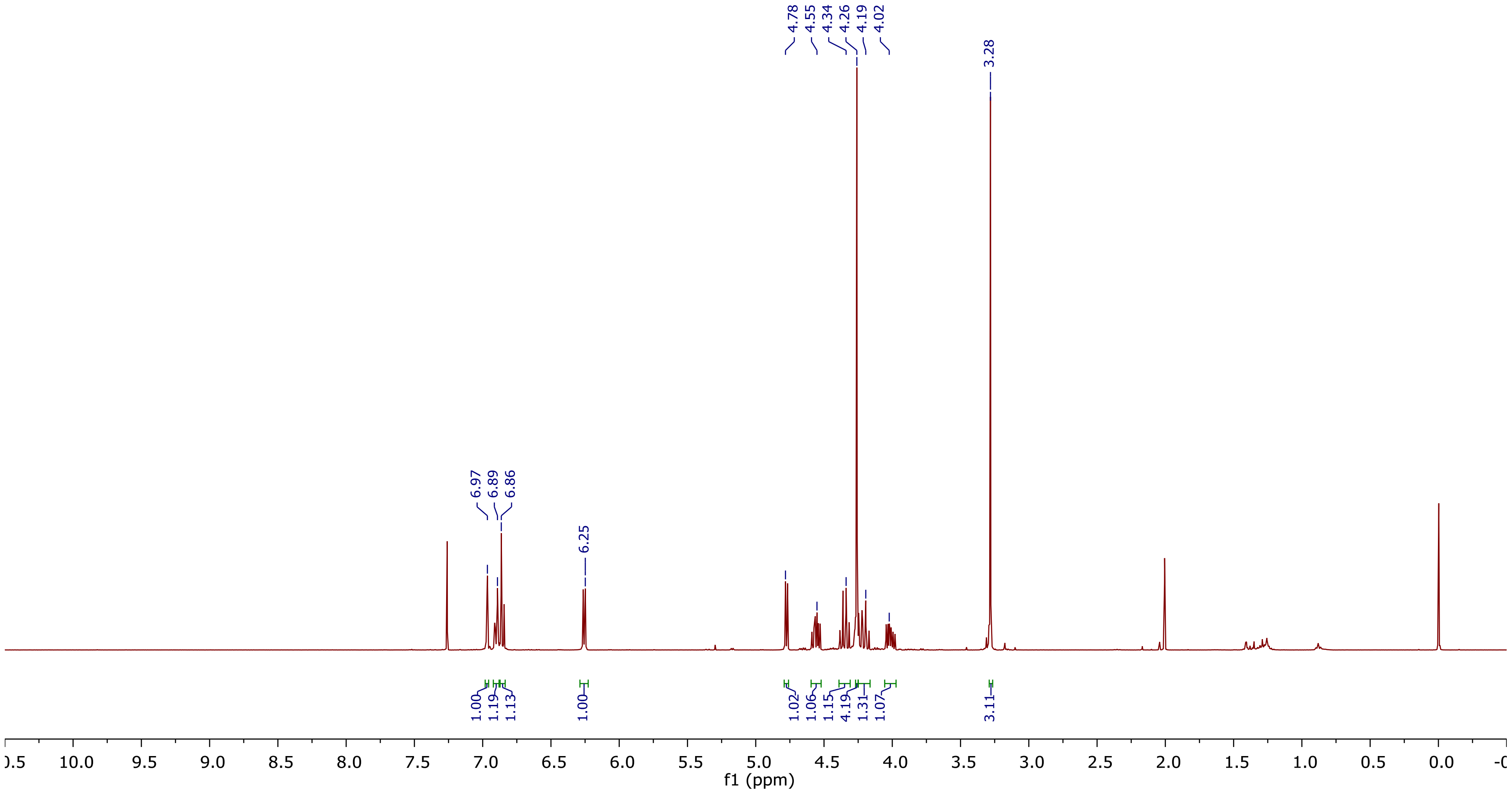

$^{13}C\{^{1}H\}$ NMR (100.6 MHz, $CDCl_3$)

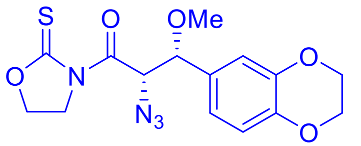

***ent*-8h**

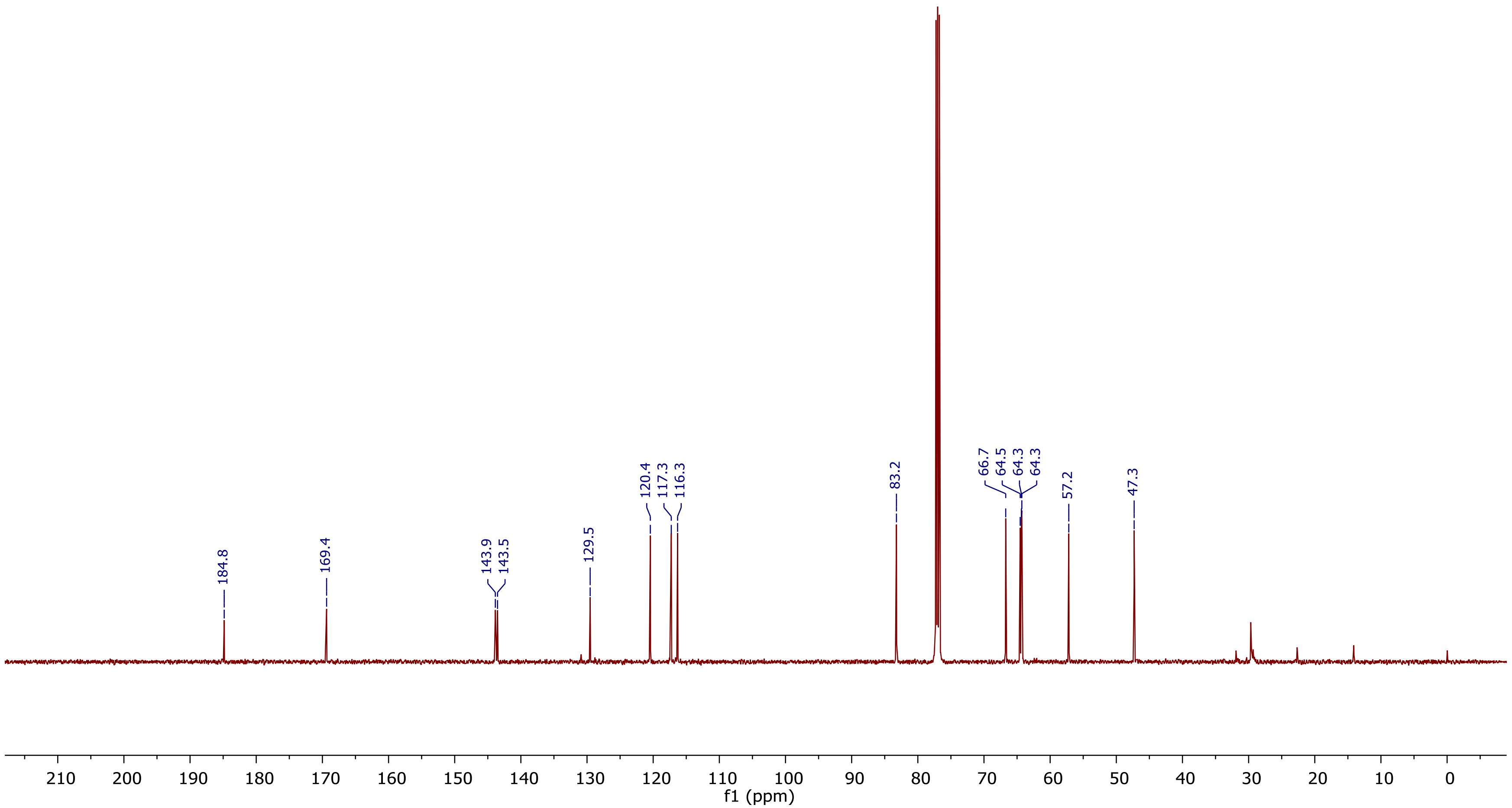

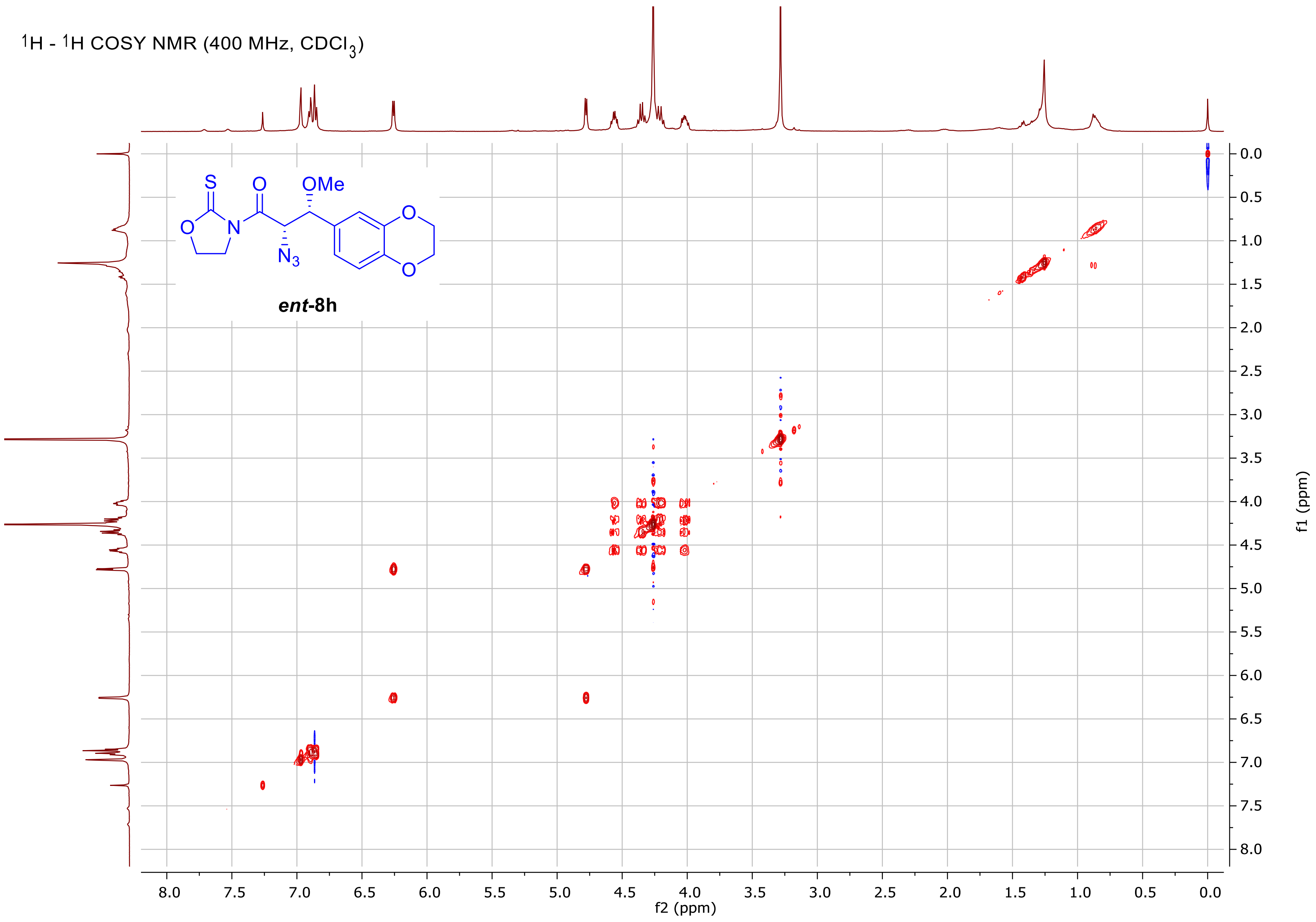

^{1}H - ^{1}H COSY NMR (400 MHz, $CDCl_3$)
S
O
OMe
O
N
N_3
O
O
ent-8h
f2 (ppm)
f1 (ppm)

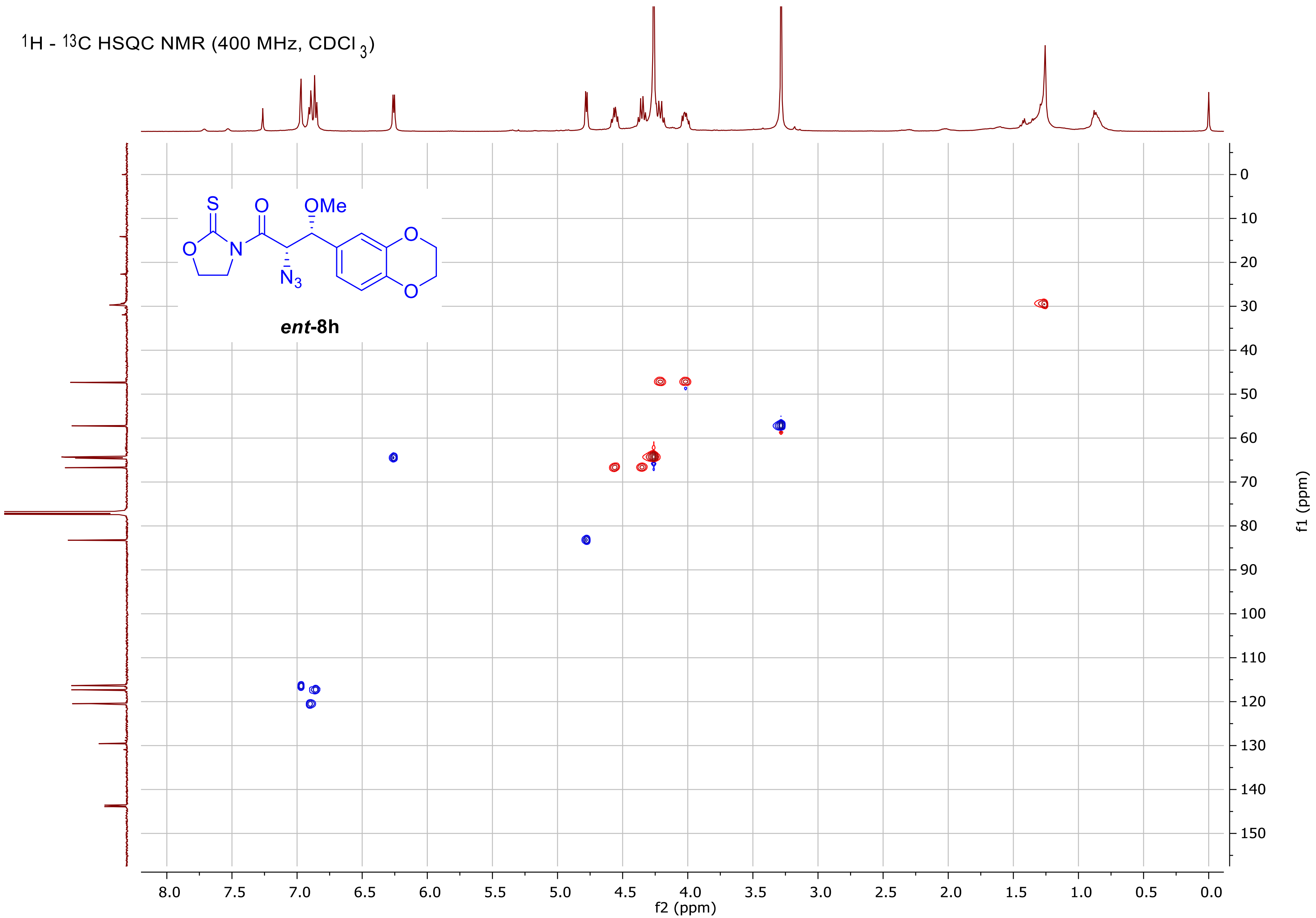

^{1}H - ^{13}C HSQC NMR (400 MHz, CDCl$_3$)
***ent*-8h**
f2 (ppm)
f1 (ppm)

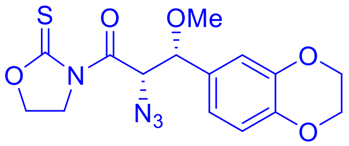

***ent*-8h**

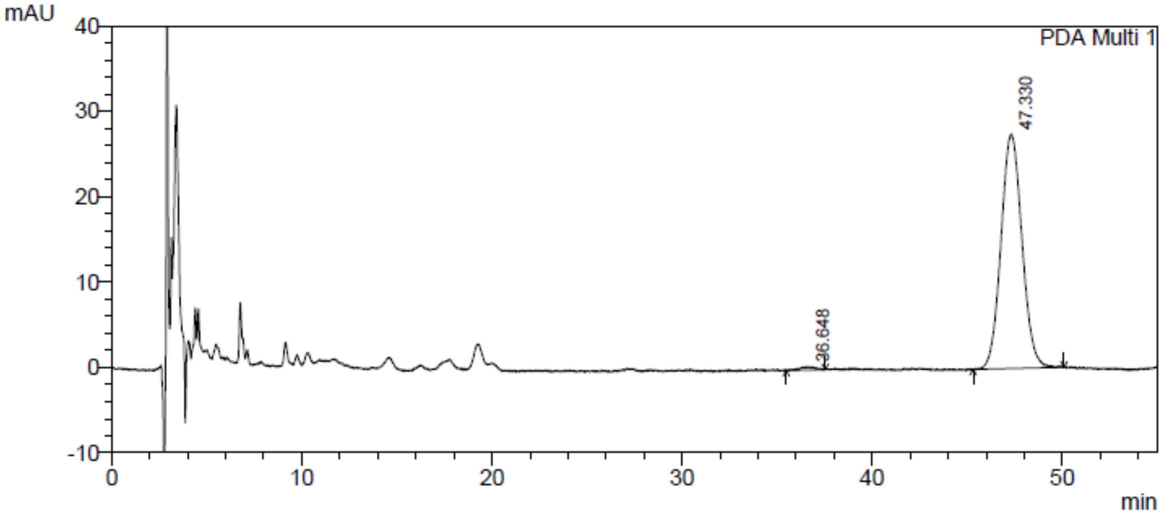

1 PDA Multi 1/254nm 4nm

PeakTable

PDA Ch1 254nm 4nm

Peak#	Ret. Time	Area	Height	Area %	Height %
1	36.648	20556	393	0.967	1.411
2	47.330	2104293	27436	99.033	98.589
Total		2124849	27828	100.000	100.000

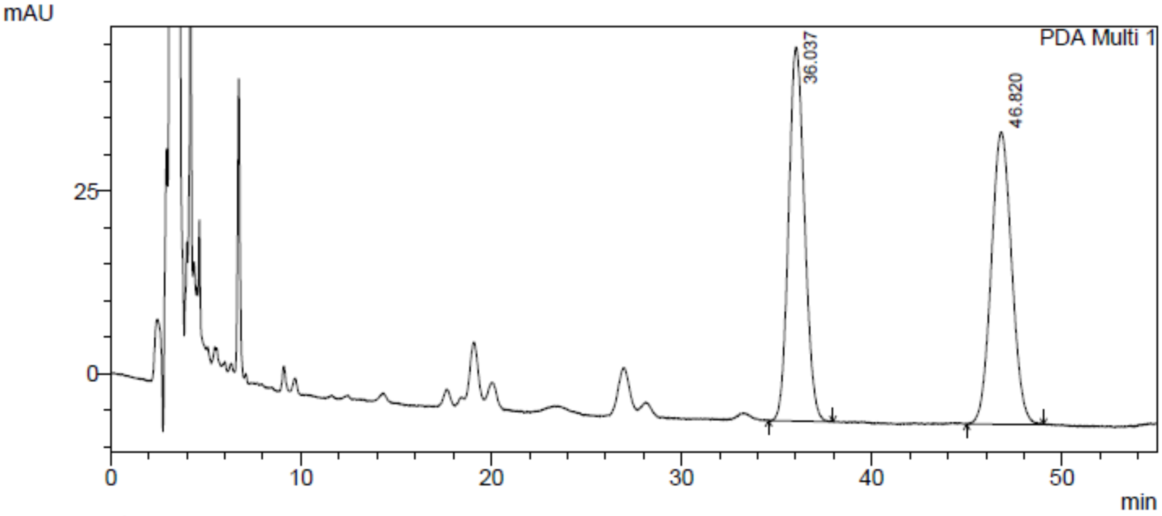

1 PDA Multi 1/254nm 4nm

PeakTable

PDA Ch1 254nm 4nm

Peak#	Ret. Time	Area	Height	Area %	Height %
1	36.037	2987716	51233	49.989	56.113
2	46.820	2989008	40071	50.011	43.887
Total		5976724	91304	100.000	100.000

^1H NMR (400 MHz, $CDCl_3$)

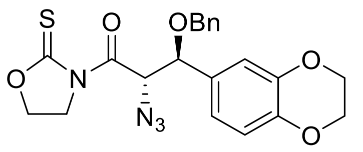

***ent*-6i**

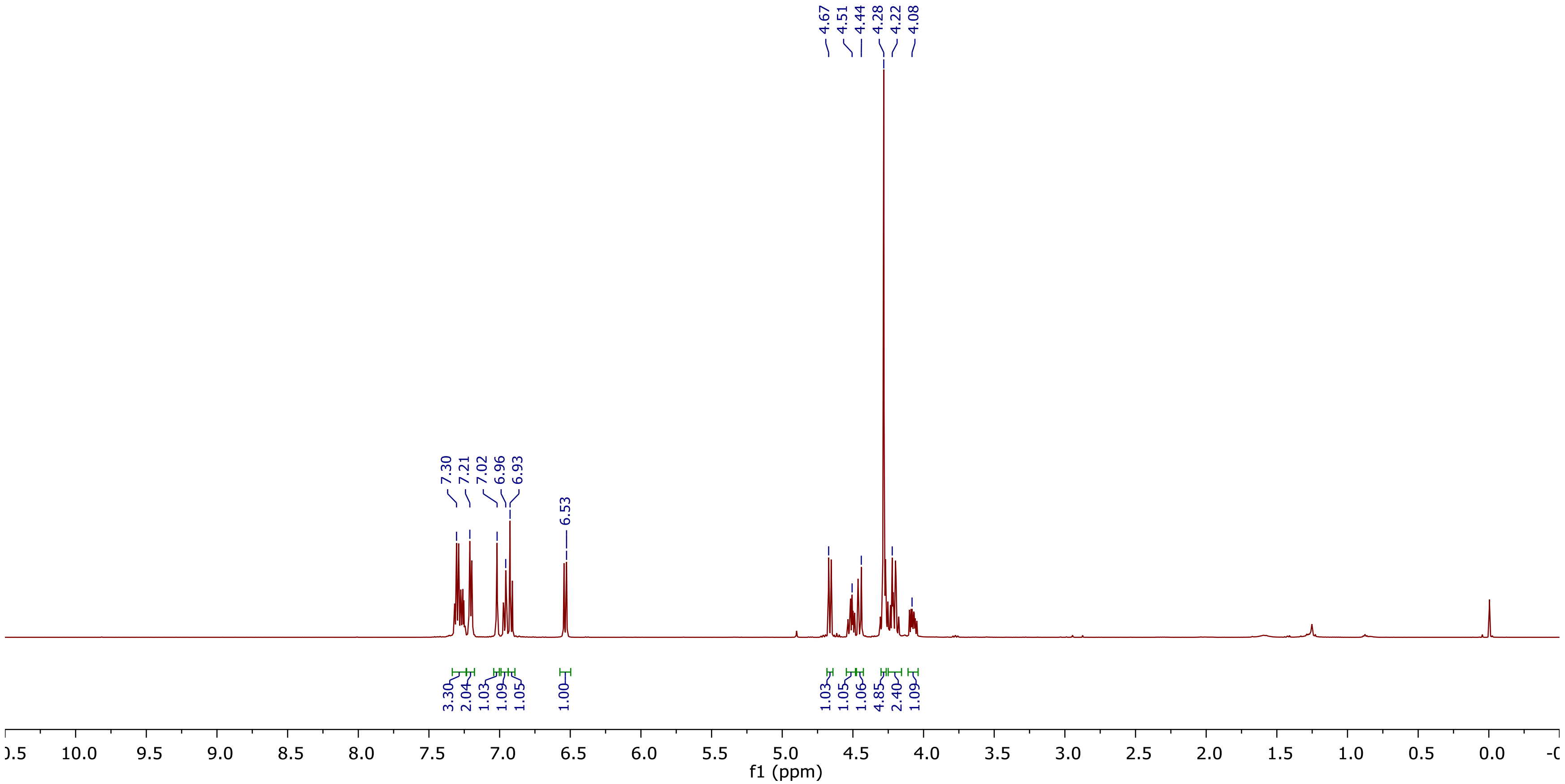

$^{13}C\{^1H\}$ NMR (100.6 MHz, $CDCl_3$)

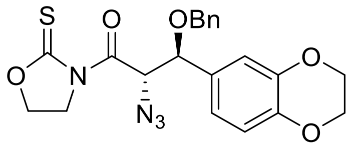

***ent*-6i**

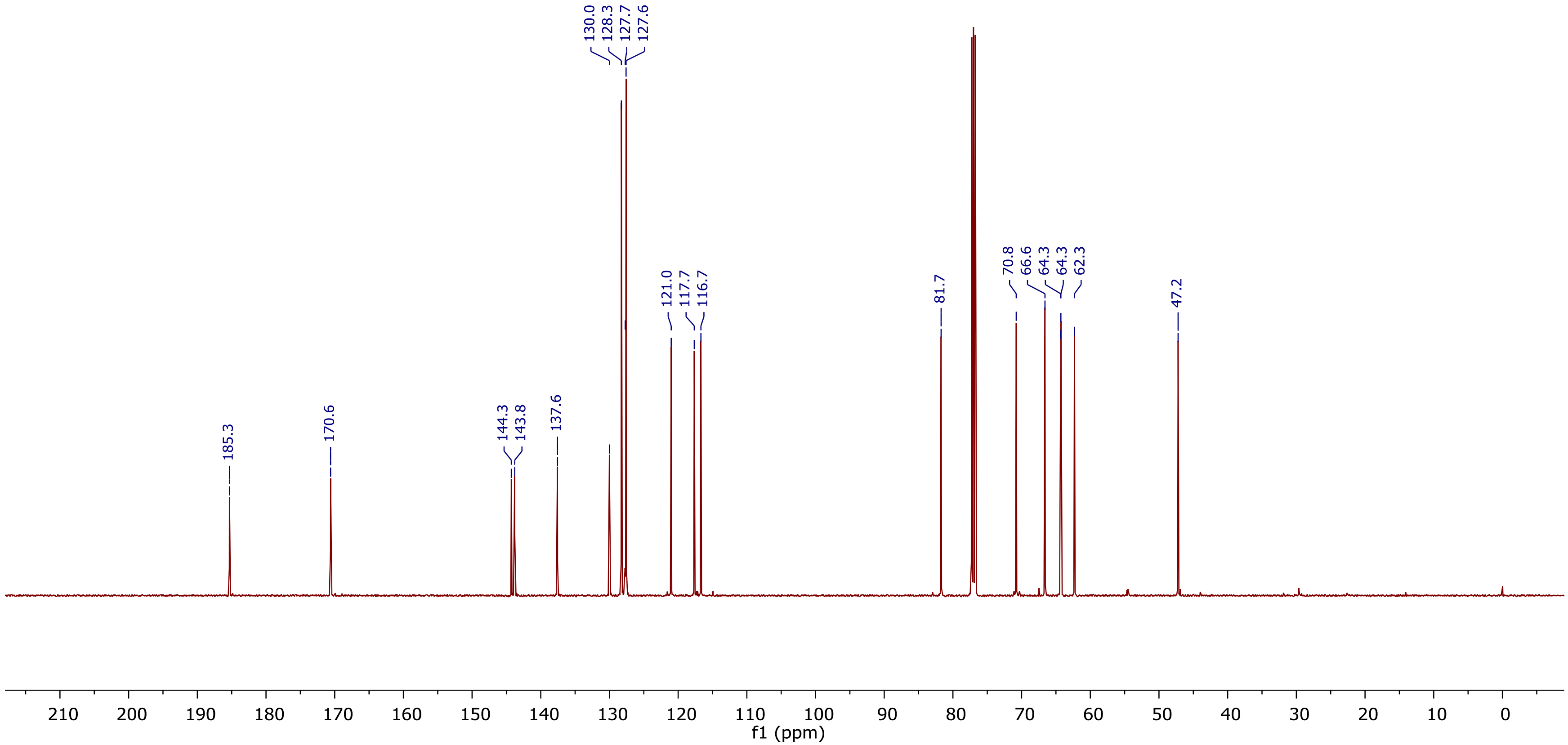

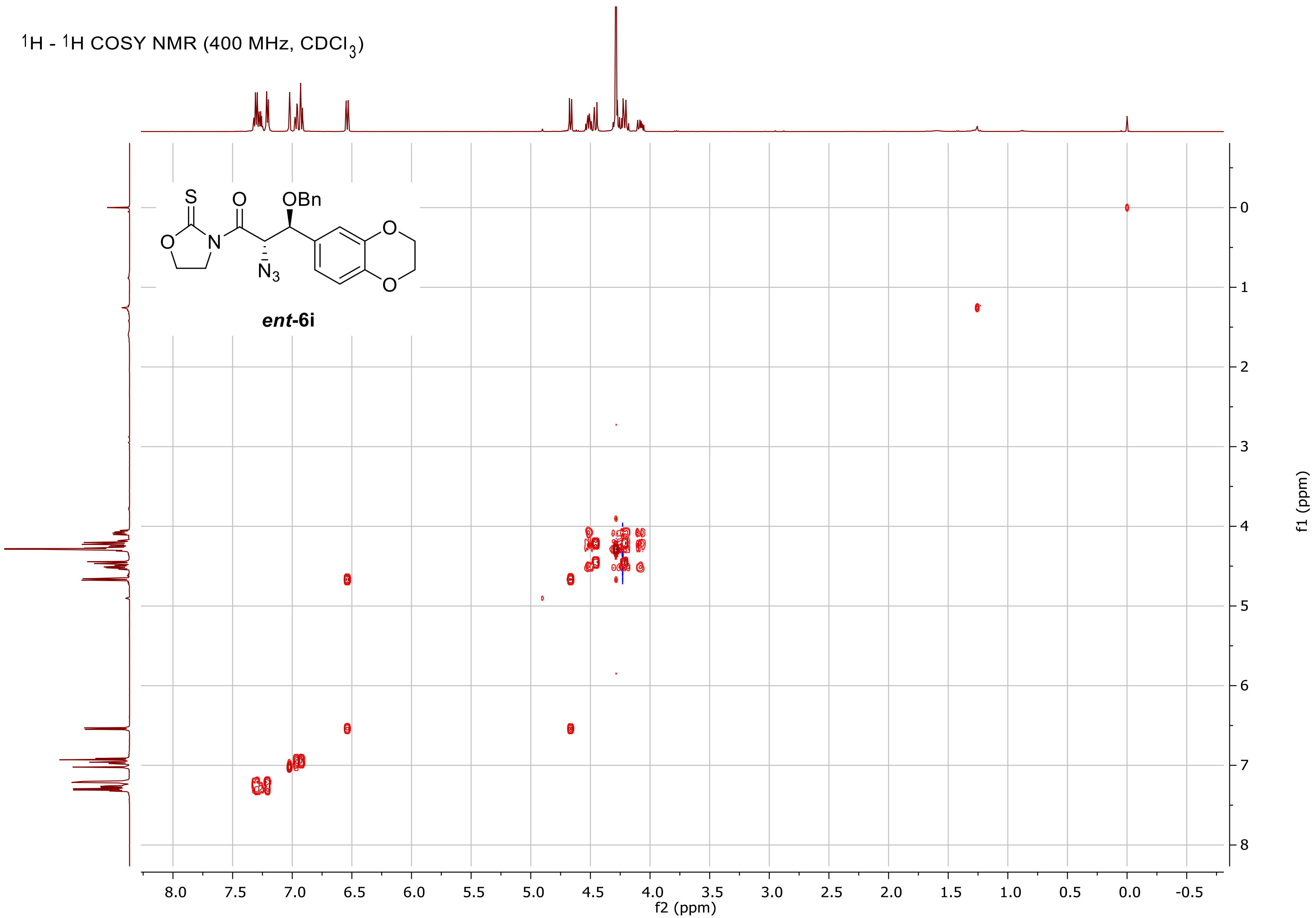

^{1}H - ^{1}H COSY NMR (400 MHz, $CDCl_3$)
S
O
OBn
O
N
O
N_3
O
ent-6i
f2 (ppm)
f1 (ppm)

^{1}H - ^{13}C HSQC NMR (400 MHz, $CDCl_3$)

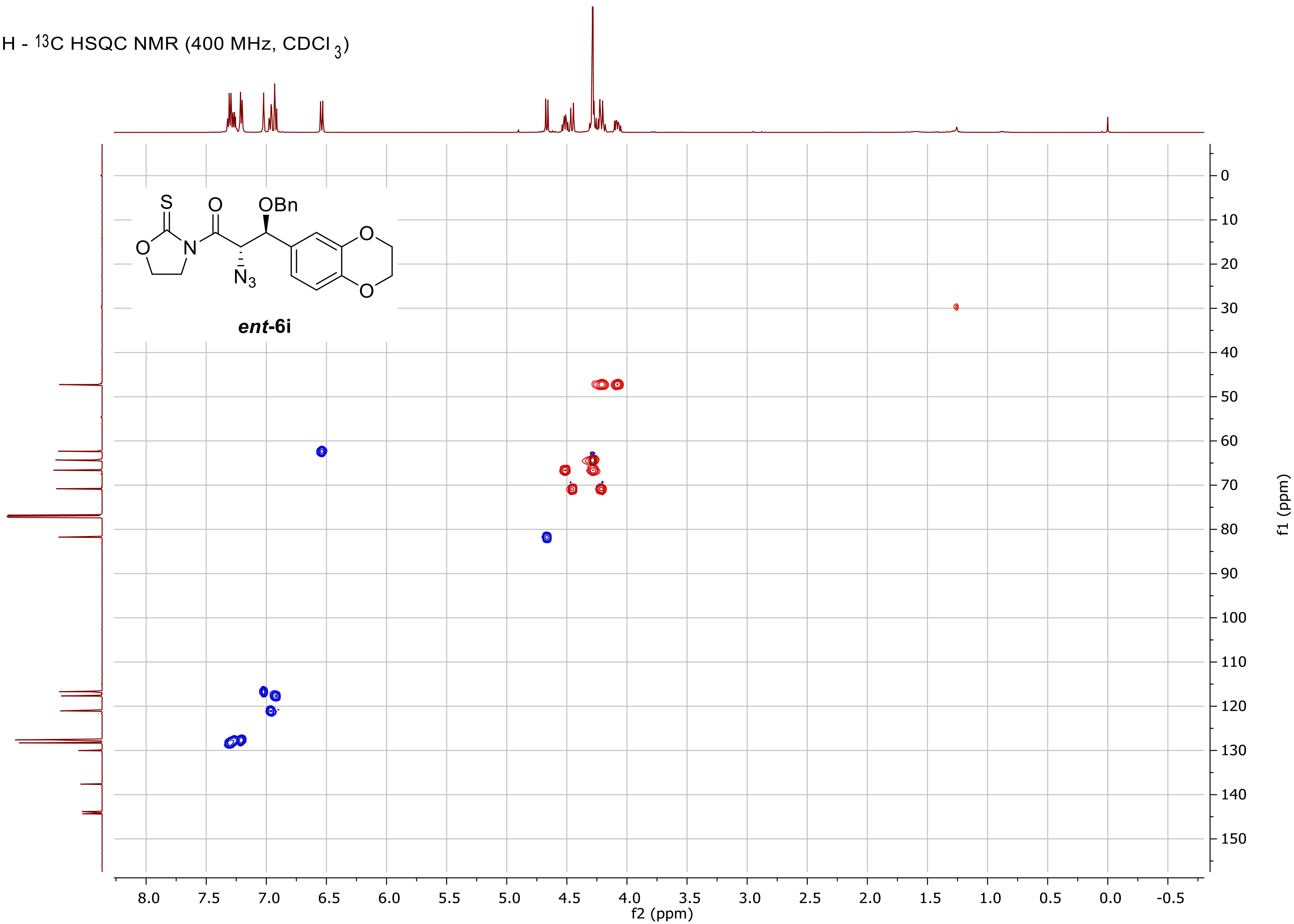

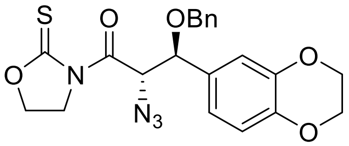

***ent*-6i**

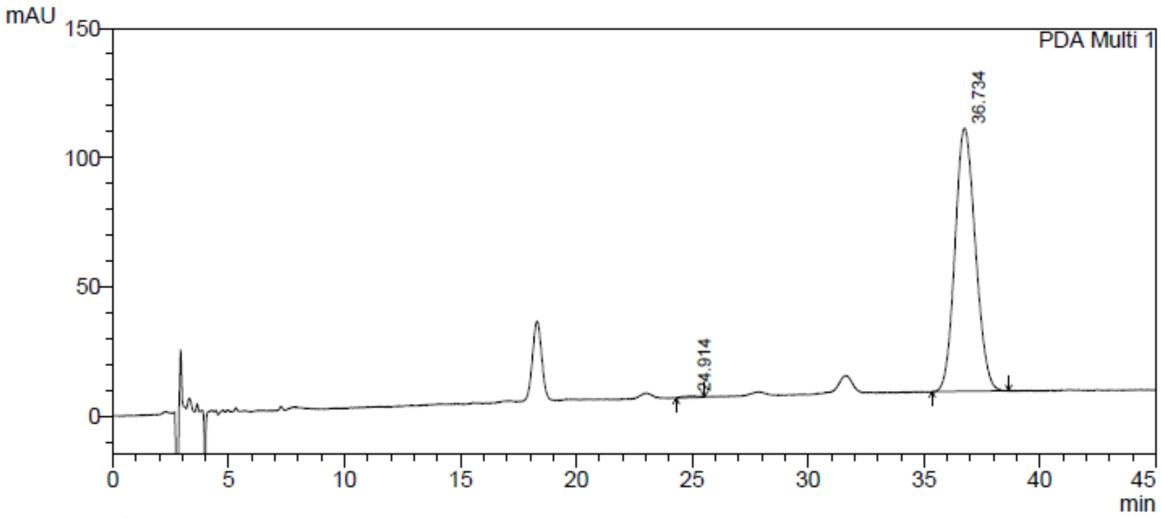

1 PDA Multi 1/254nm 4nm

PeakTable

PDA Ch1 254nm 4nm

Peak#	Ret. Time	Area	Height	Area %	Height %
1	24.914	26821	719	0.428	0.702
2	36.734	6233969	101772	99.572	99.298
Total		6260790	102491	100.000	100.000

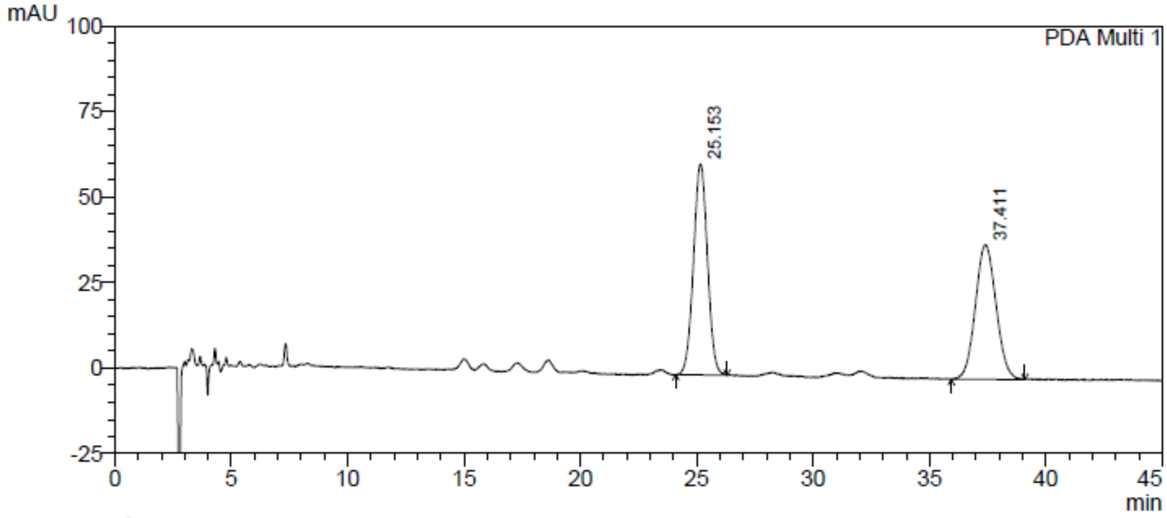

1 PDA Multi 1/254nm 4nm

PeakTable

PDA Ch1 254nm 4nm

Peak#	Ret. Time	Area	Height	Area %	Height %
1	25.153	2574417	61699	51.389	61.060
2	37.411	2435273	39347	48.611	38.940
Total		5009690	101046	100.000	100.000

^{1}H NMR (400 MHz, $CDCl_3$)

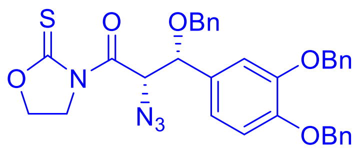

***ent*-8j**

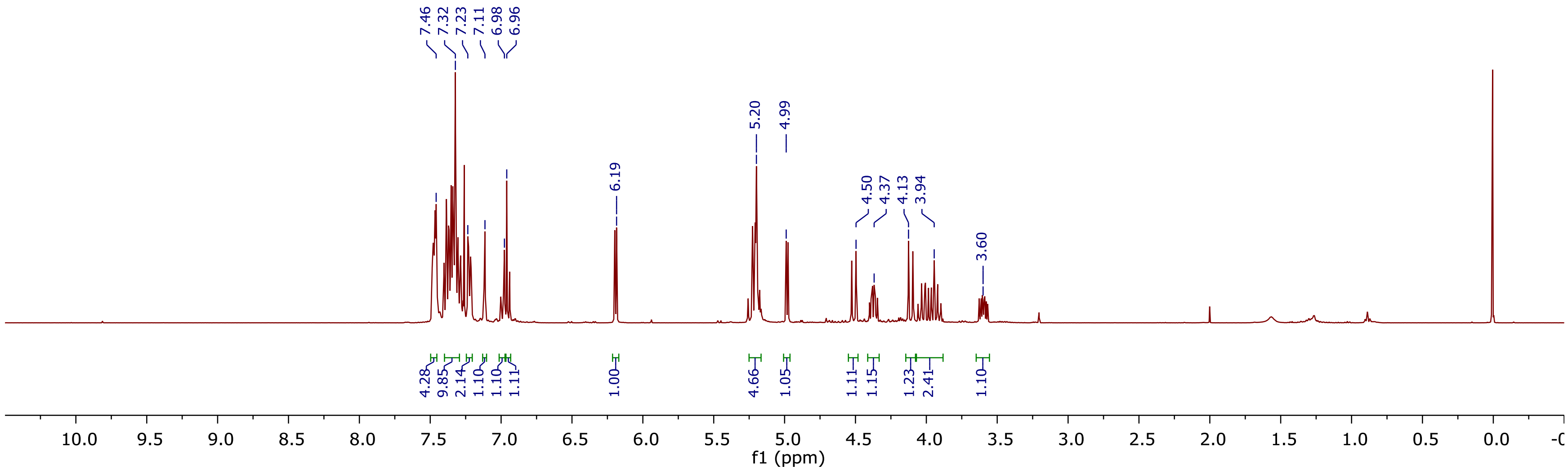

$^{13}C\{^{1}H\}$ NMR (100.6 MHz, $CDCl_3$)

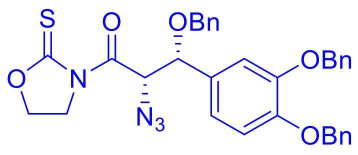

***ent*-8j**

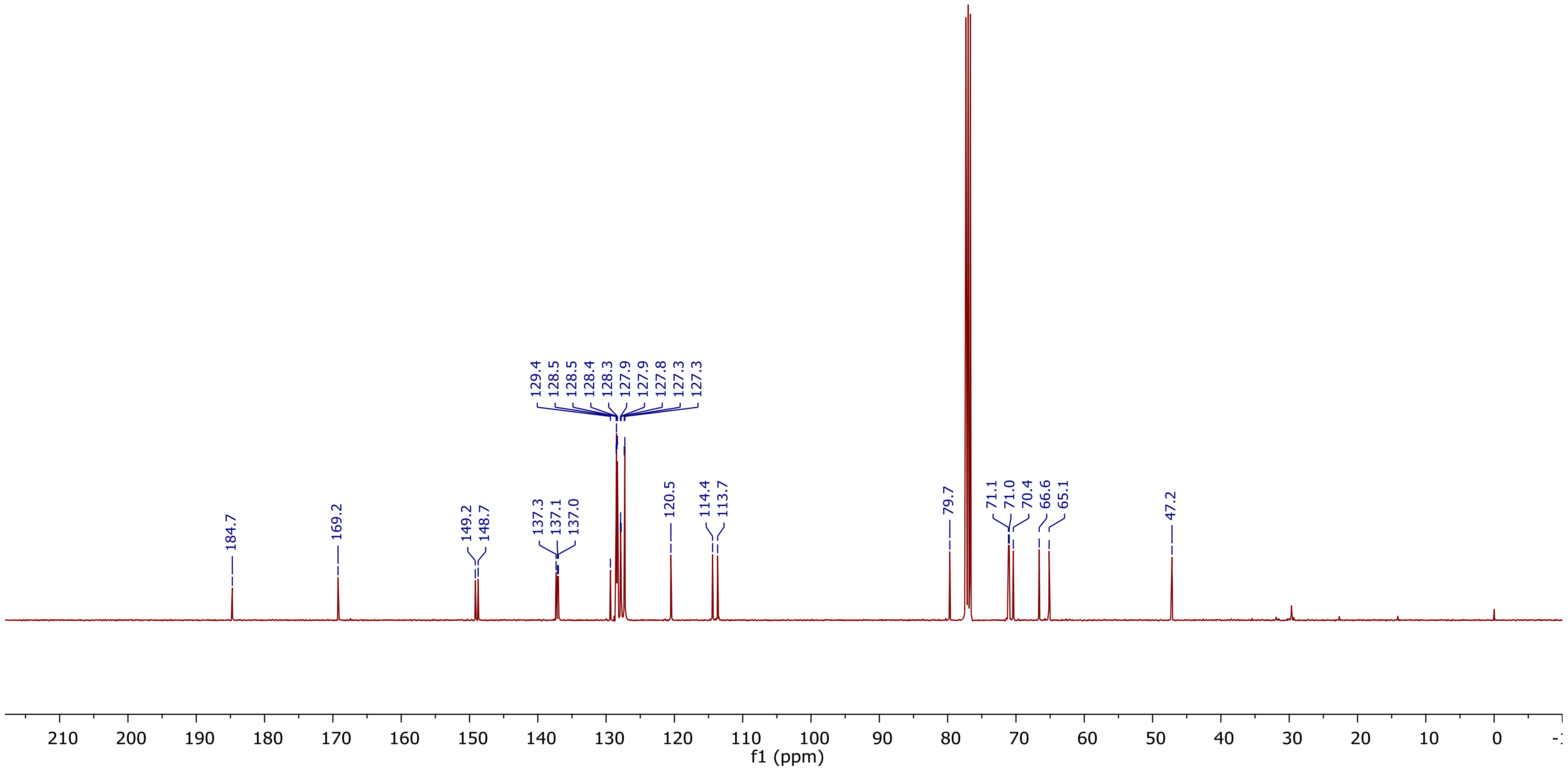

^1H - ^1H COSY NMR (400 MHz, $CDCl_3$)

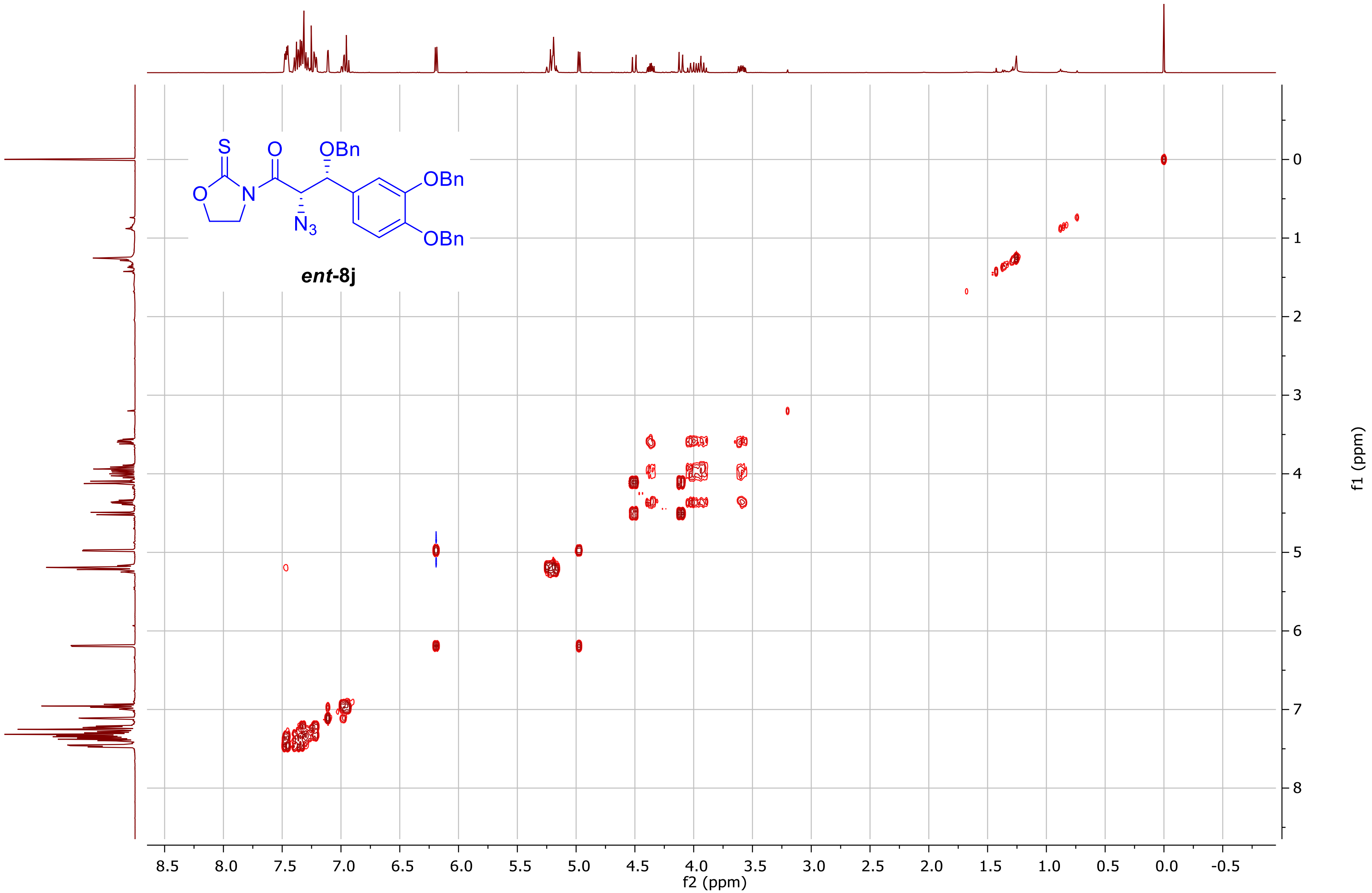

^1H - ^{13}C HSQC NMR (400 MHz, $CDCl_3$)

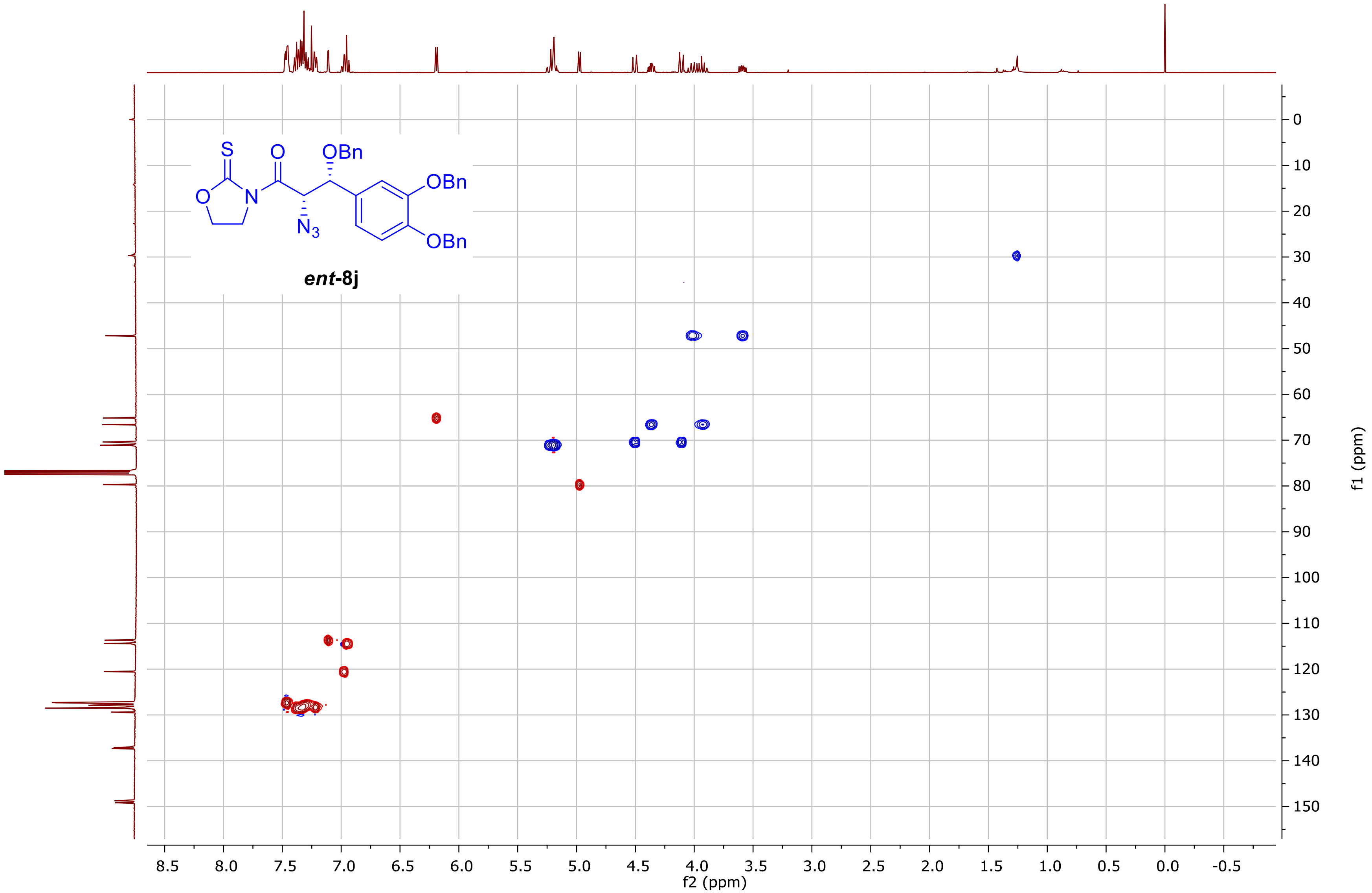

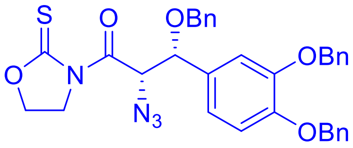

***ent*-8j**

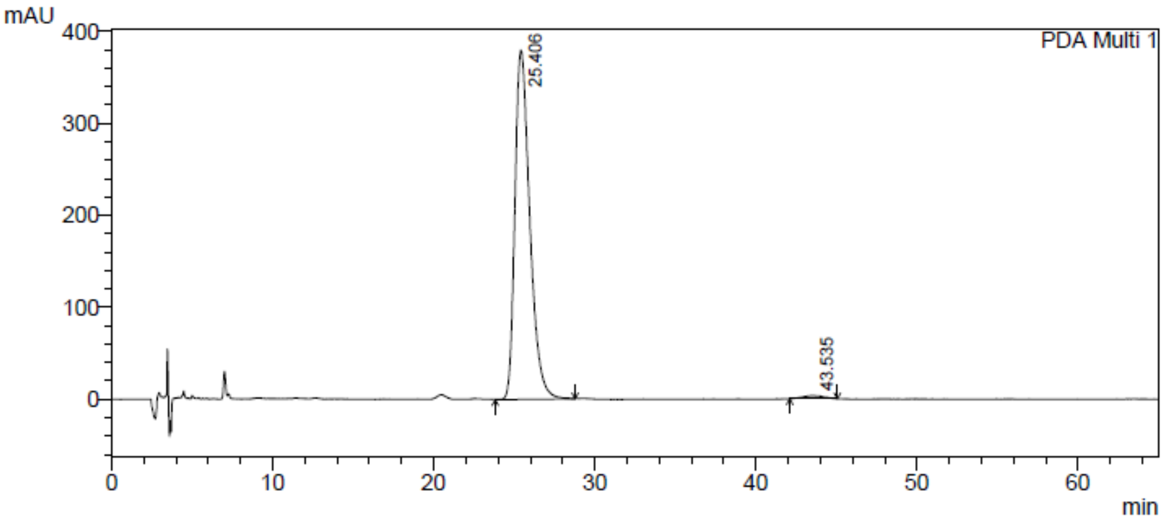

1 PDA Multi 1/254nm 4nm

PeakTable

PDA Ch1 254nm 4nm

Peak#	Ret. Time	Area	Height	Area %	Height %
1	25.406	24380729	379883	98.751	99.062
2	43.535	308400	3596	1.249	0.938
Total		24689129	383479	100.000	100.000

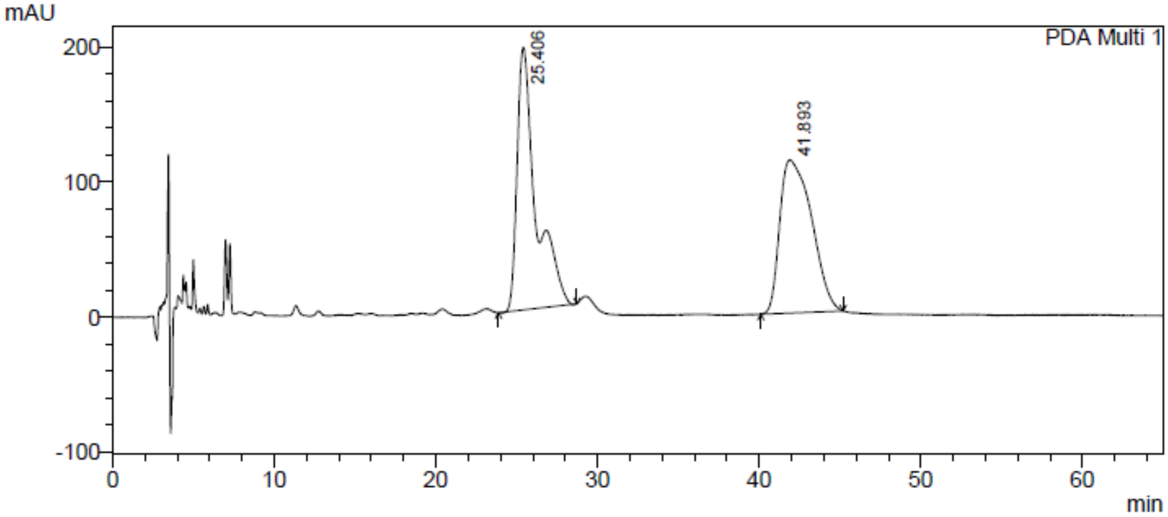

1 PDA Multi 1/254nm 4nm

PeakTable

PDA Ch1 254nm 4nm

Peak#	Ret. Time	Area	Height	Area %	Height %
1	25.406	15606634	194682	50.104	63.178
2	41.893	15541682	113468	49.896	36.822
Total		31148316	308150	100.000	100.000

^1H NMR (400 MHz, $CDCl_3$)

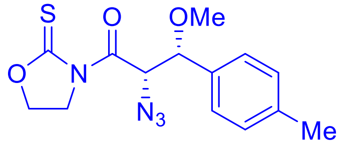

***ent*-8k**

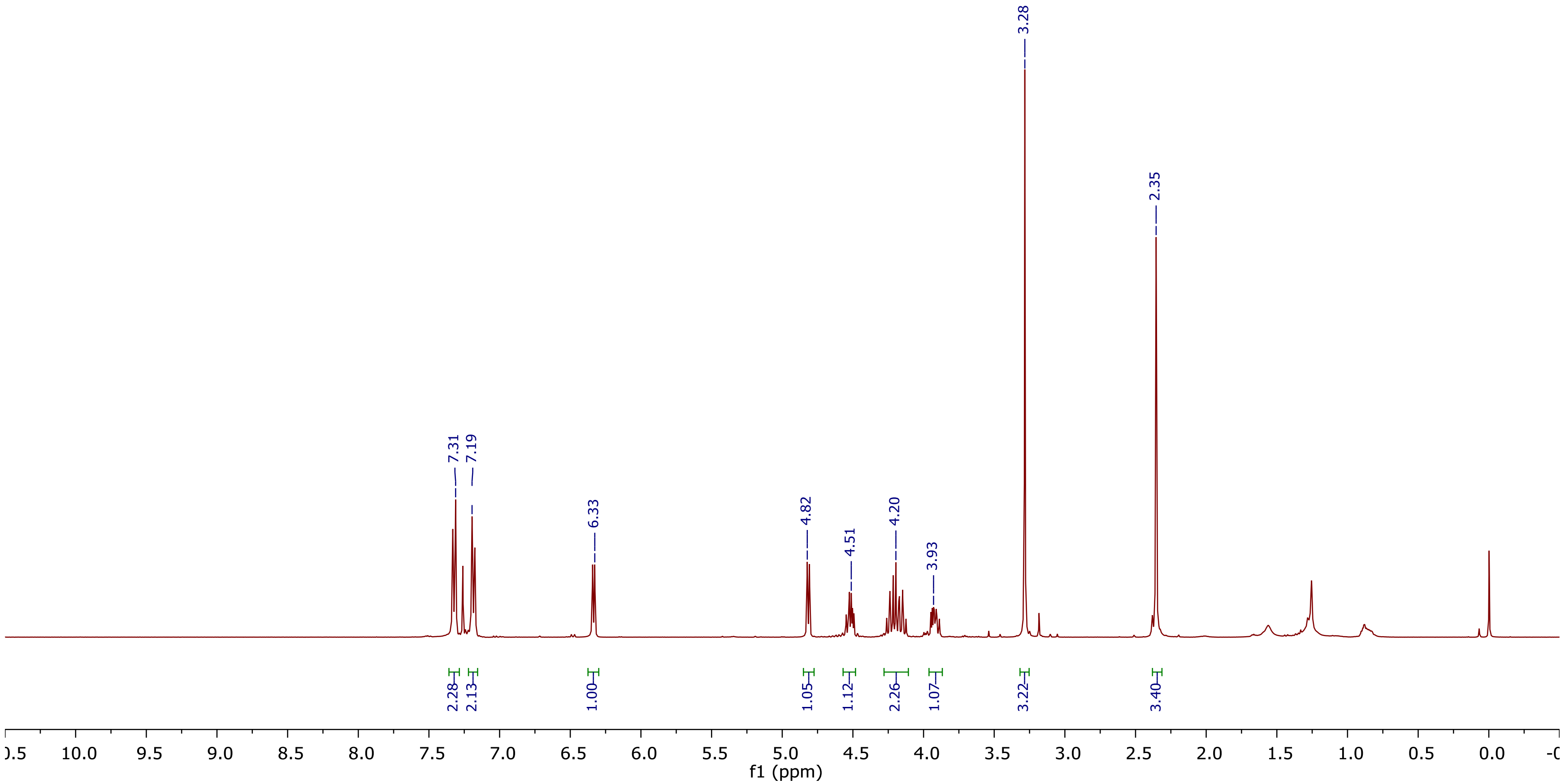

$^{13}C\{^1H\}$ NMR (100.6 MHz, $CDCl_3$)

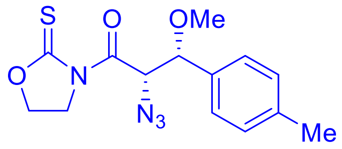

***ent*-8k**

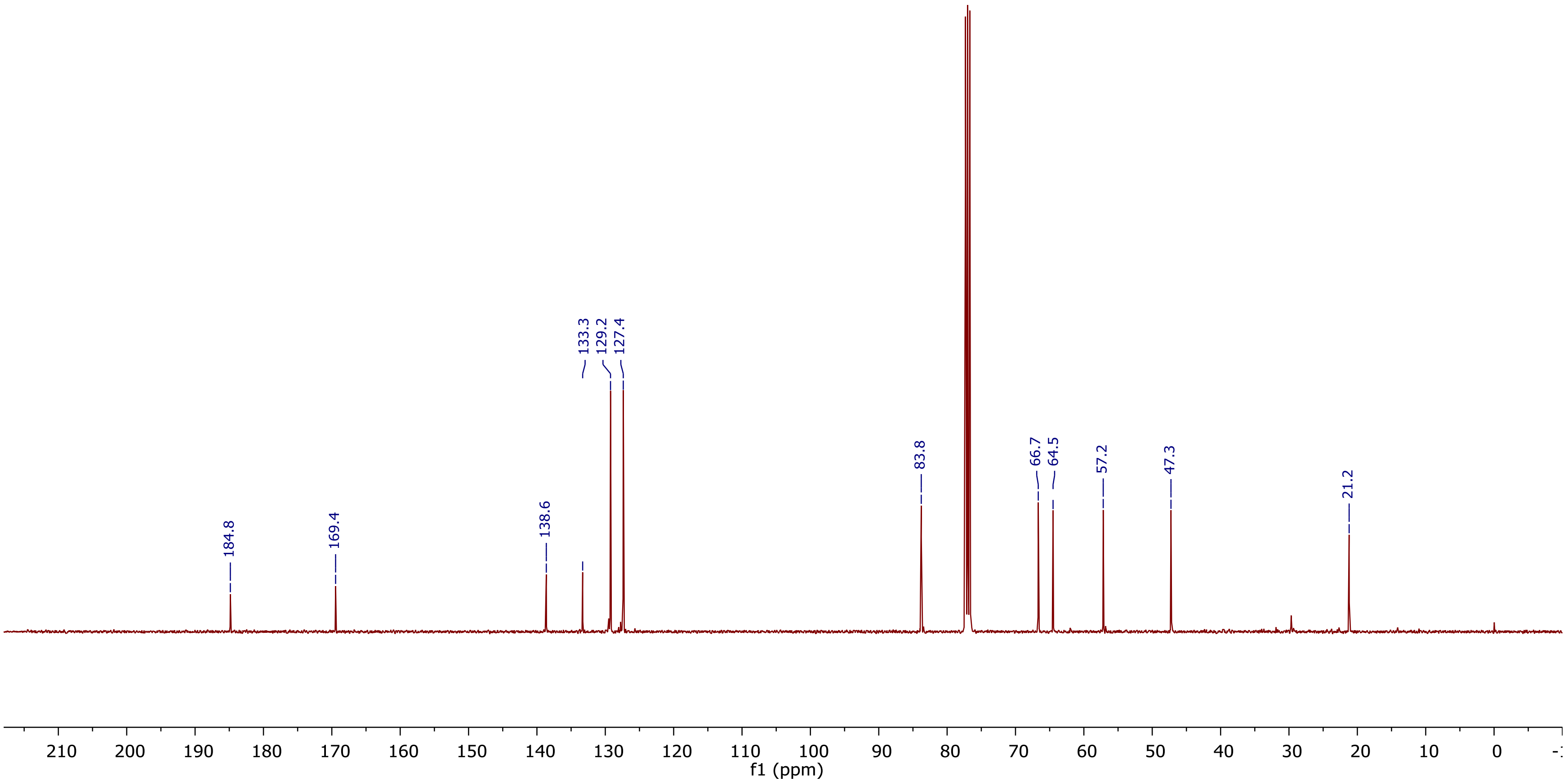

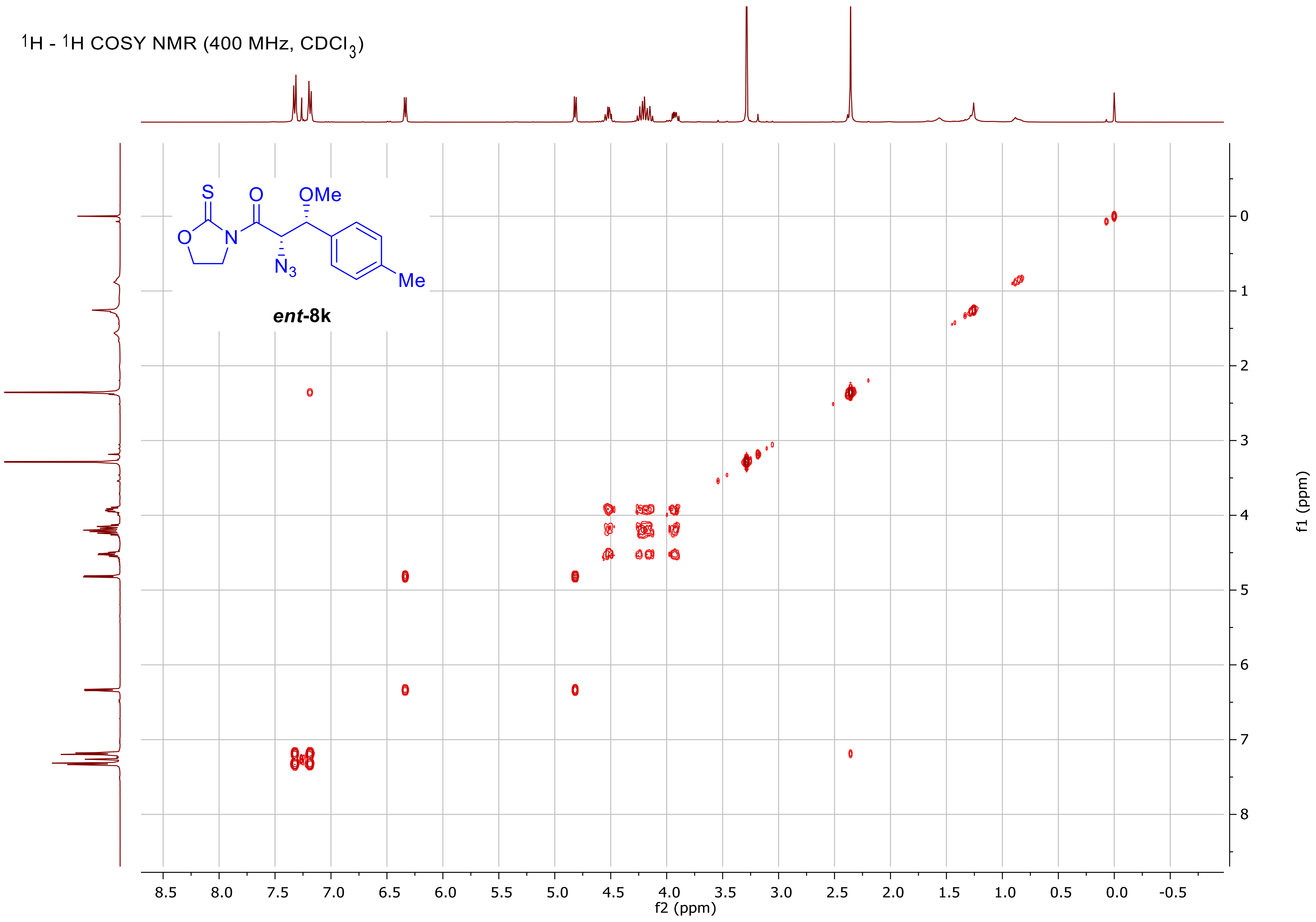
1H - 1H COSY NMR (400 MHz, CDCl3)
S
O
OMe
O
N
N3
Me
ent-8k
f2 (ppm)
f1 (ppm)

^1H - ^{13}C HSQC NMR (400 MHz, $CDCl_3$)

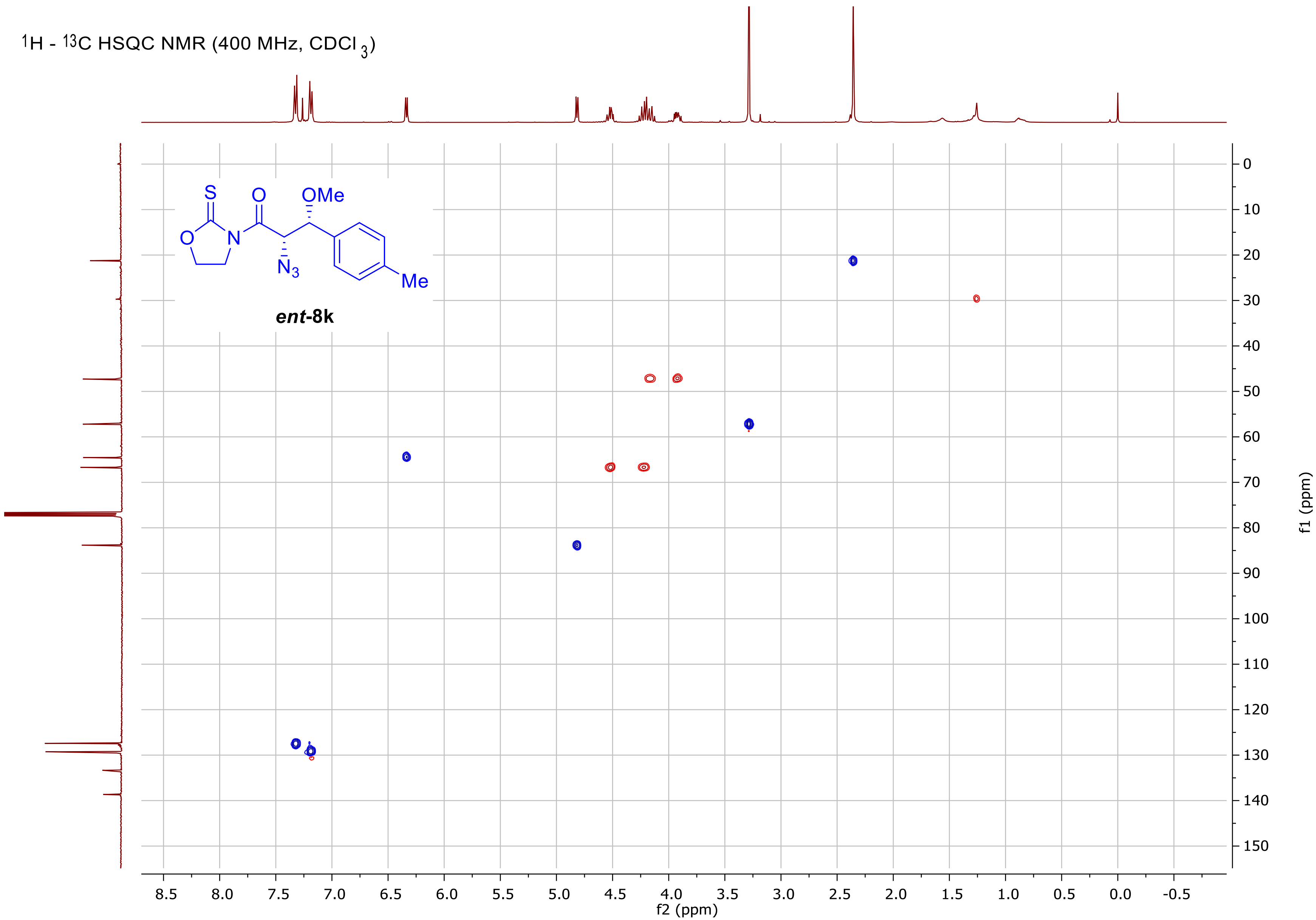

***ent*-8k**

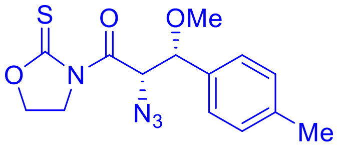

***ent*-8k**

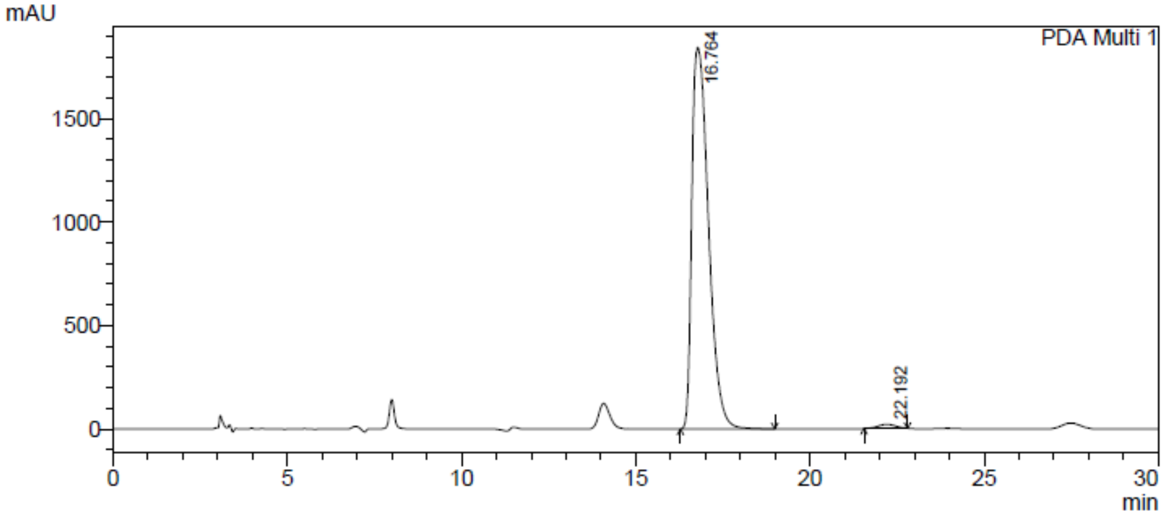

1 PDA Multi 1/254nm 4nm

PeakTable

PDA Ch1 254nm 4nm

Peak#	Ret. Time	Area	Height	Area %	Height %
1	16.764	61342600	1845251	98.909	98.939
2	22.192	676558	19788	1.091	1.061
Total		62019158	1865039	100.000	100.000

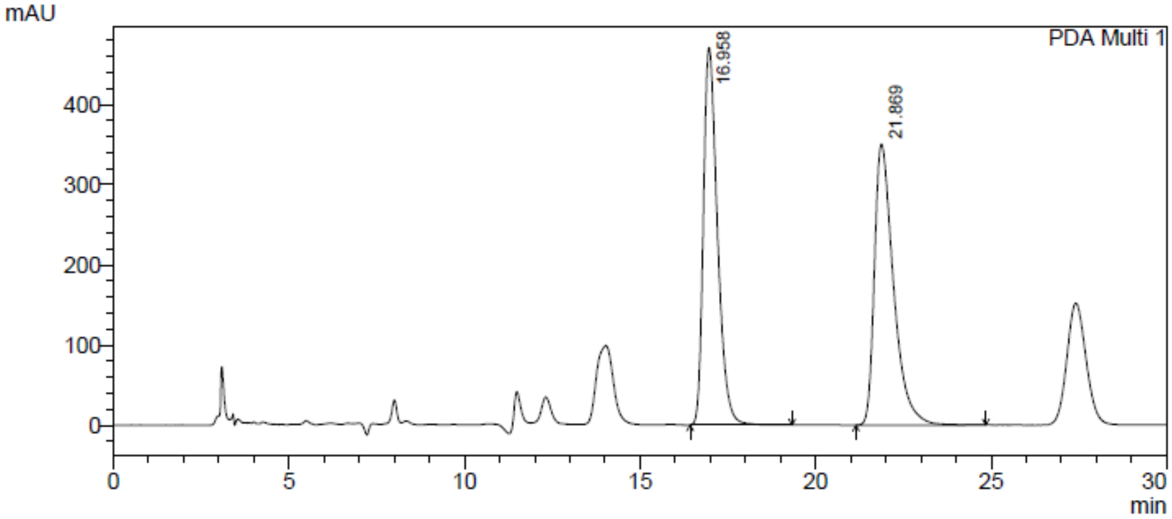

1 PDA Multi 1/254nm 4nm

PeakTable

PDA Ch1 254nm 4nm

Peak#	Ret. Time	Area	Height	Area %	Height %
1	16.958	13120080	470571	49.731	57.322
2	21.869	13261913	350357	50.269	42.678
Total		26381993	820928	100.000	100.000

^{1}H NMR (400 MHz, $CDCl_3$)

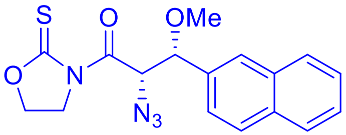

***ent*-8l**

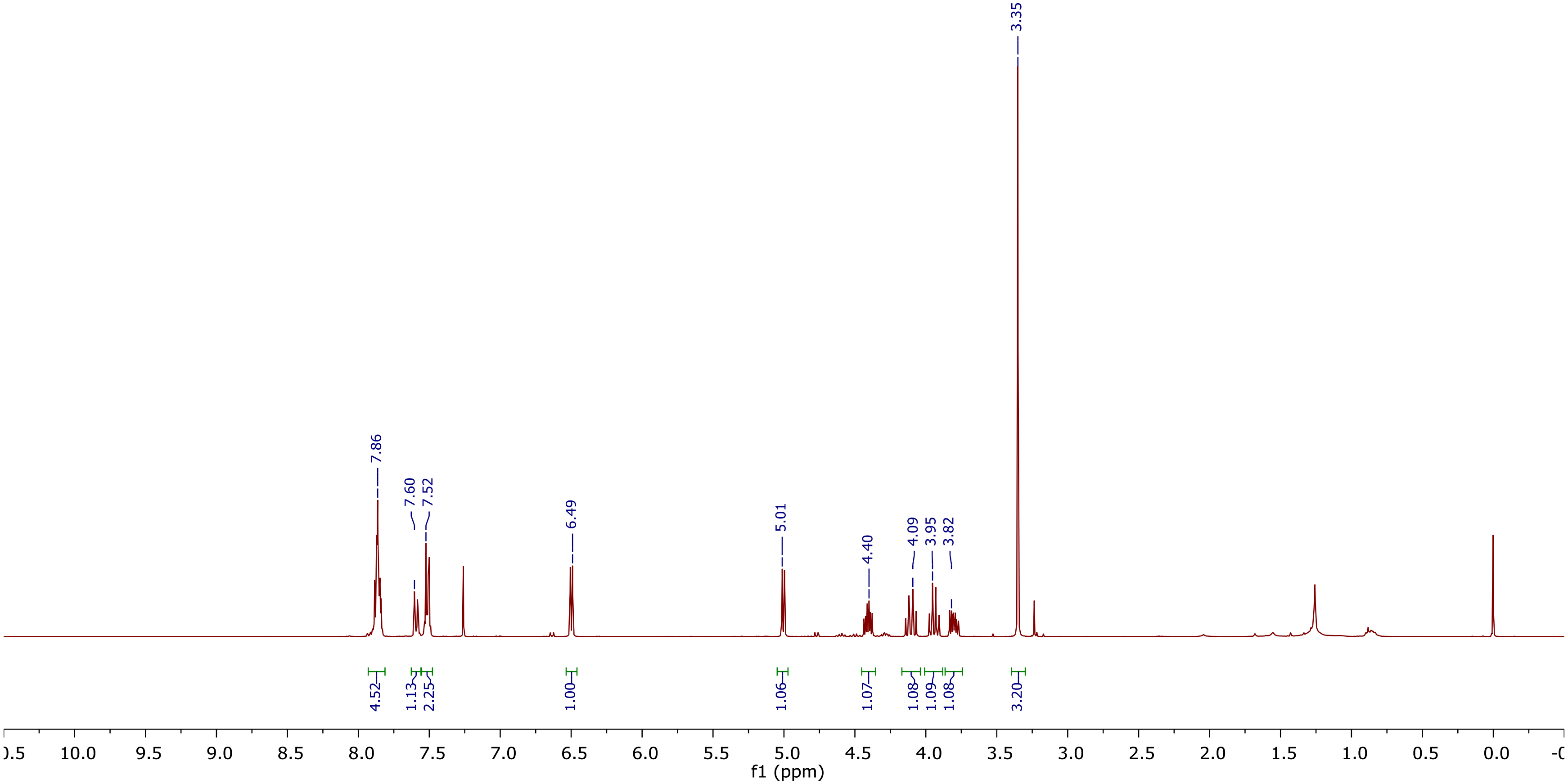

$^{13}C\{^1H\}$ NMR (100.6 MHz, $CDCl_3$)

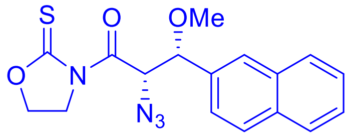

***ent*-8l**

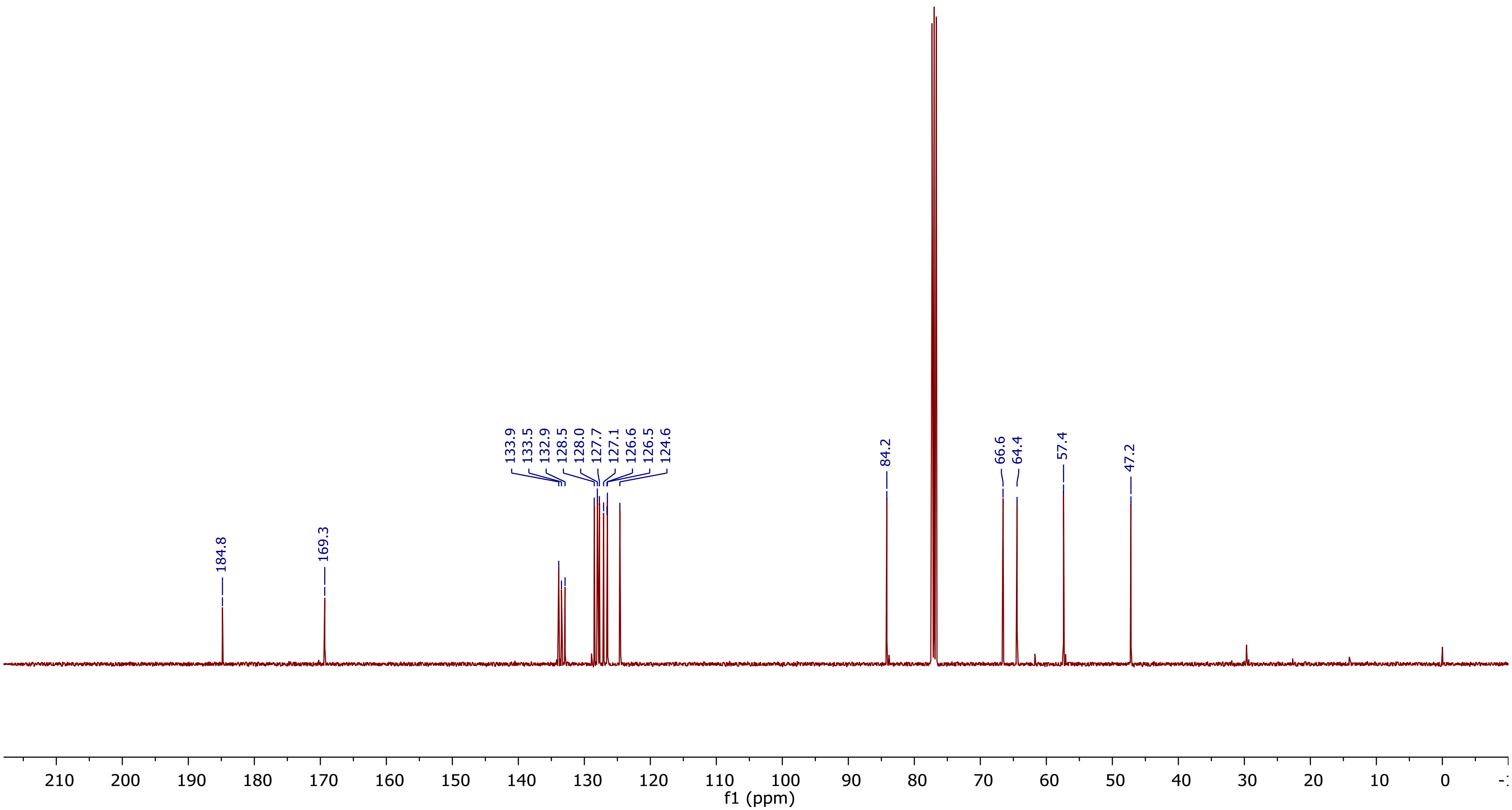

^{1}H - ^{1}H COSY NMR (400 MHz, $CDCl_3$)

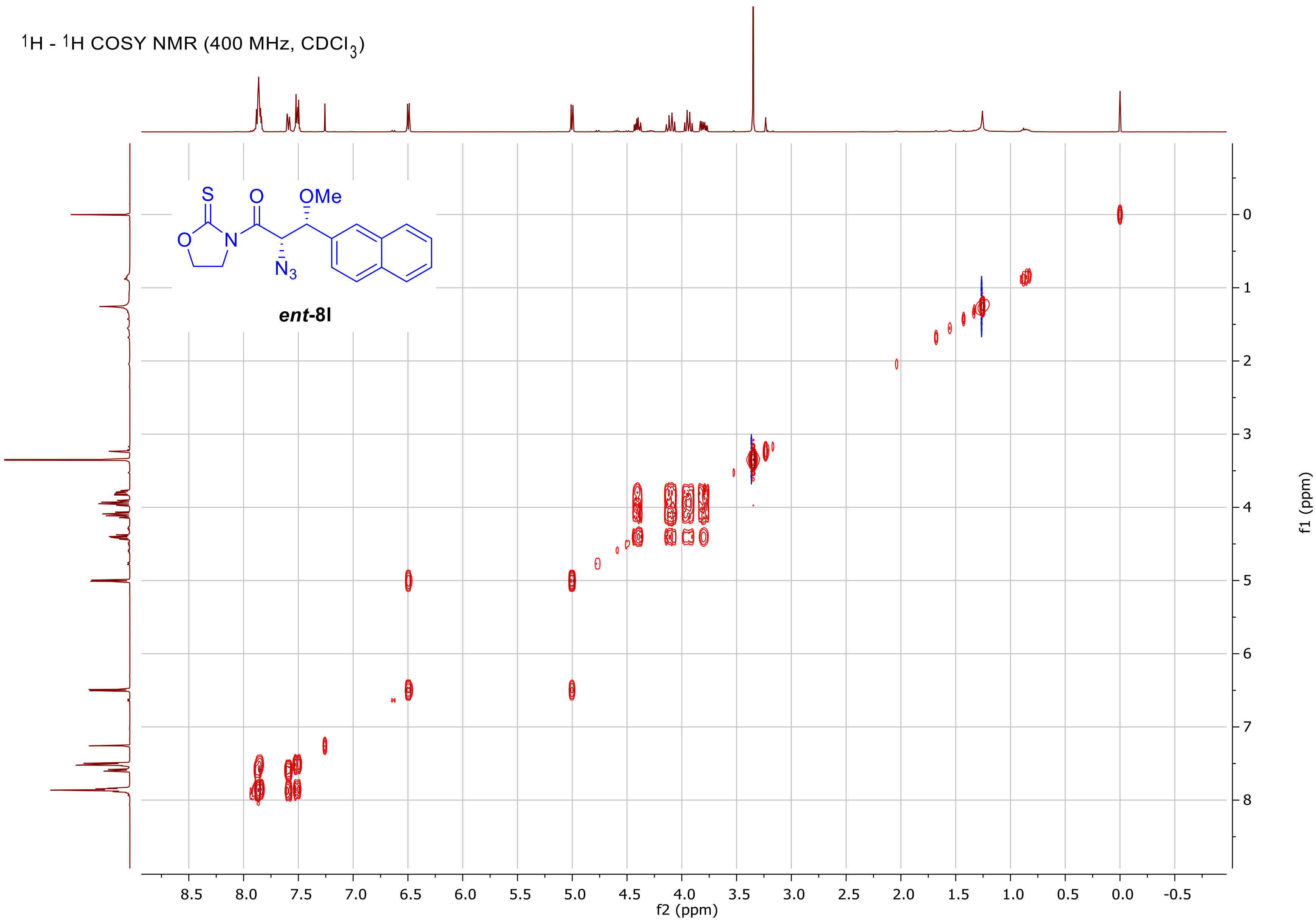

^{1}H - ^{13}C HSQC NMR (400 MHz, $CDCl_3$)

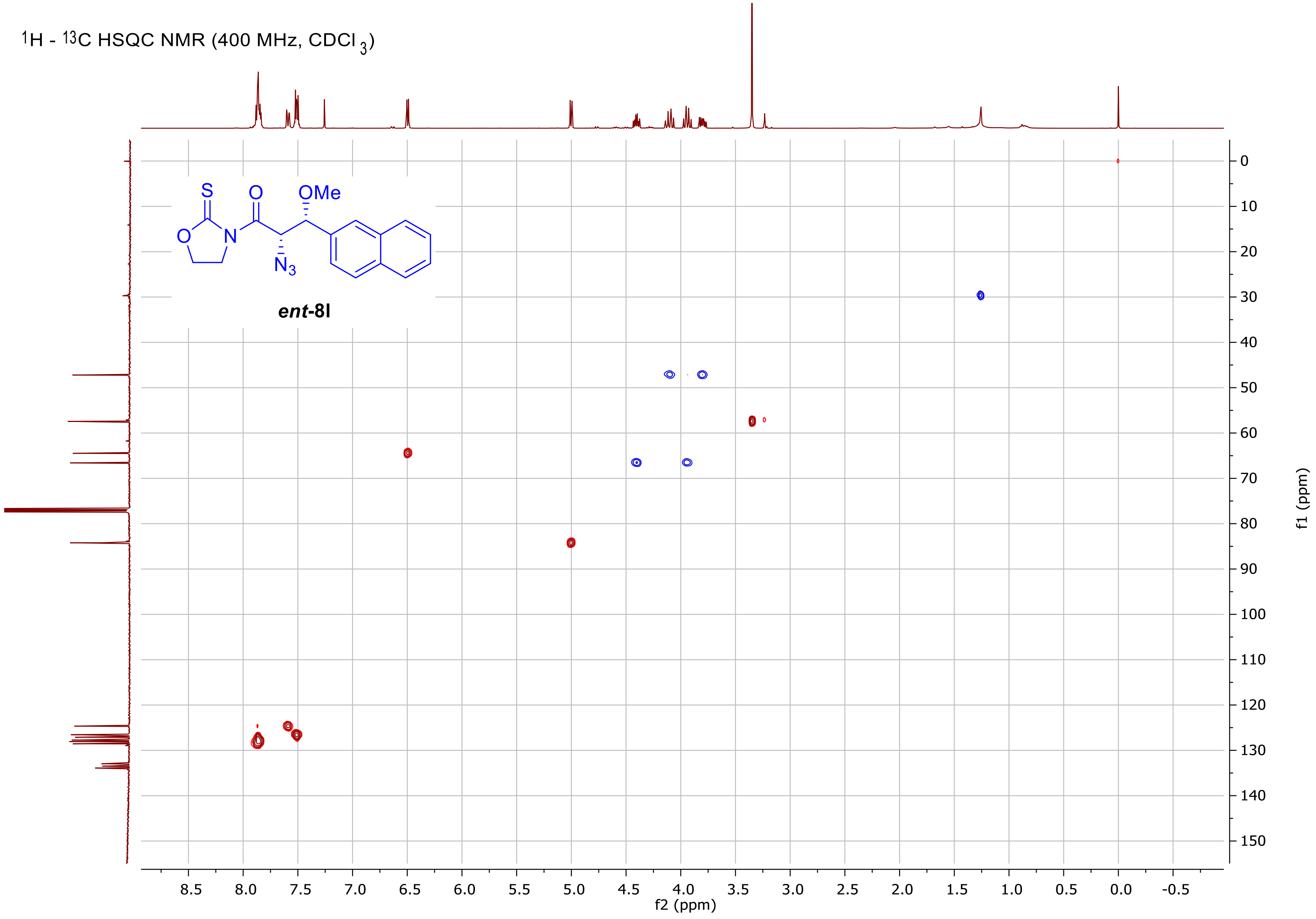

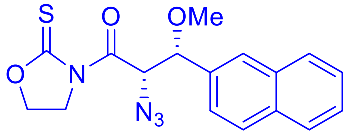

***ent*-8l**

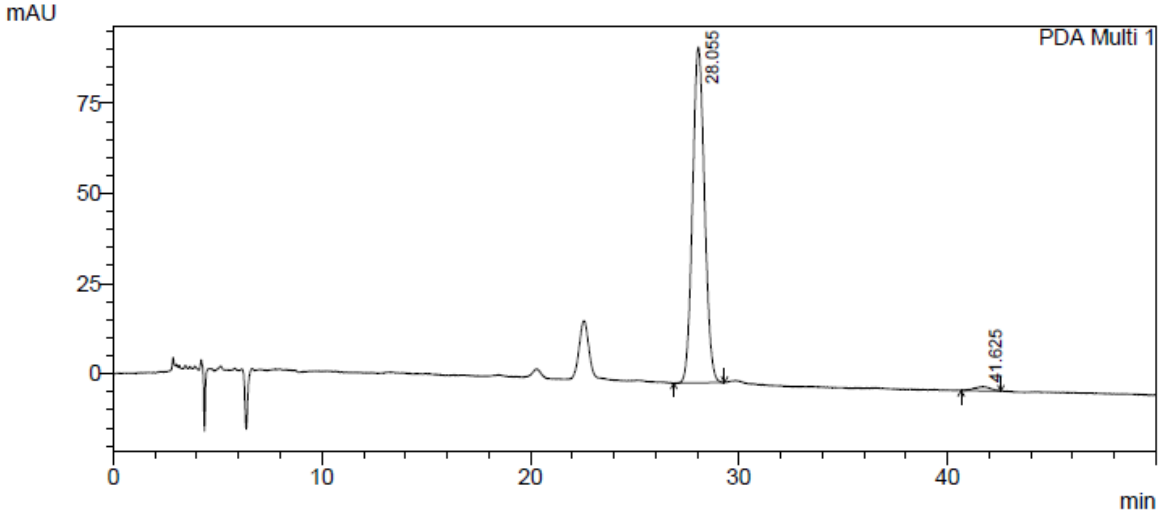

1 PDA Multi 1/254nm 4nm

PeakTable

PDA Ch1 254nm 4nm

Peak#	Ret. Time	Area	Height	Area %	Height %
1	28.055	3721598	93071	98.384	98.775
2	41.625	61122	1155	1.616	1.225
Total		3782721	94226	100.000	100.000

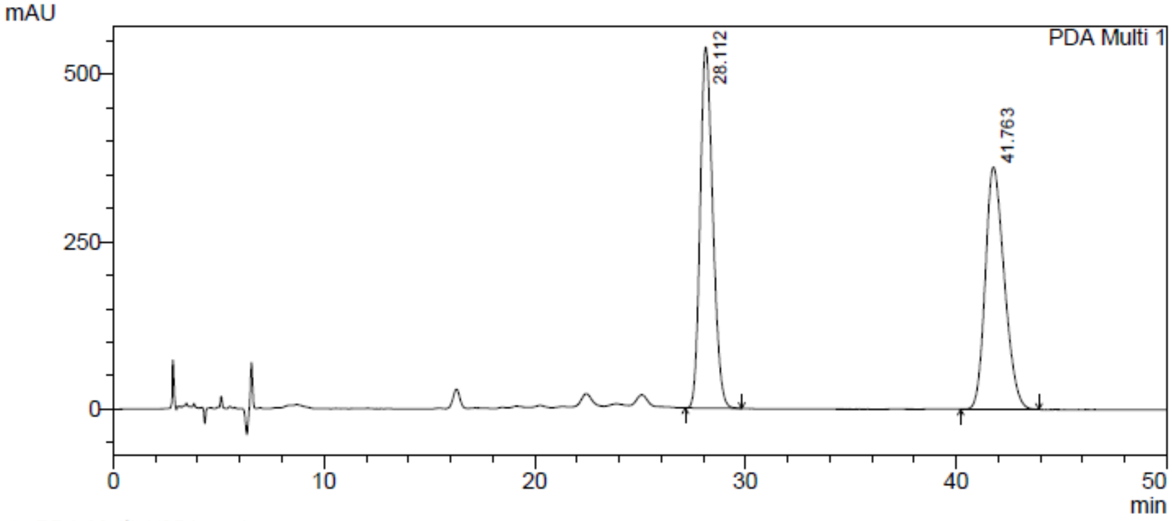

1 PDA Multi 1/254nm 4nm

PeakTable

PDA Ch1 254nm 4nm

Peak#	Ret. Time	Area	Height	Area %	Height %
1	28.112	22549534	538340	49.797	59.837
2	41.763	22733566	361337	50.203	40.163
Total		45283100	899677	100.000	100.000

^{1}H NMR (400 MHz, $CDCl_3$)

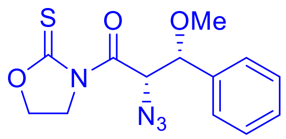

***ent*-8m**

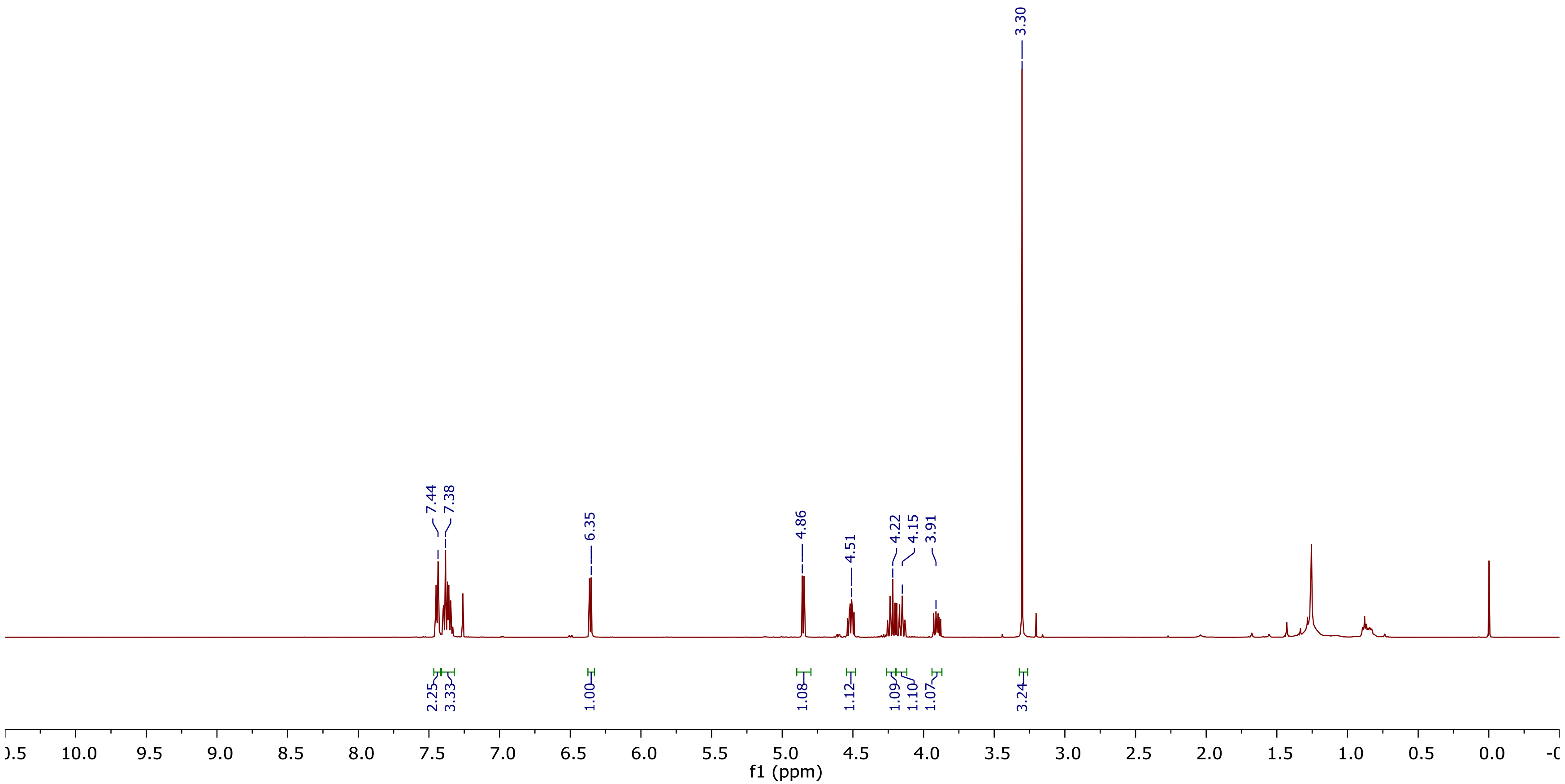

$^{13}C\{^{1}H\}$ NMR (100.6 MHz, $CDCl_3$)

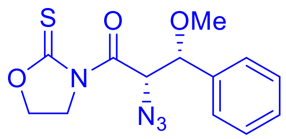

***ent*-8m**

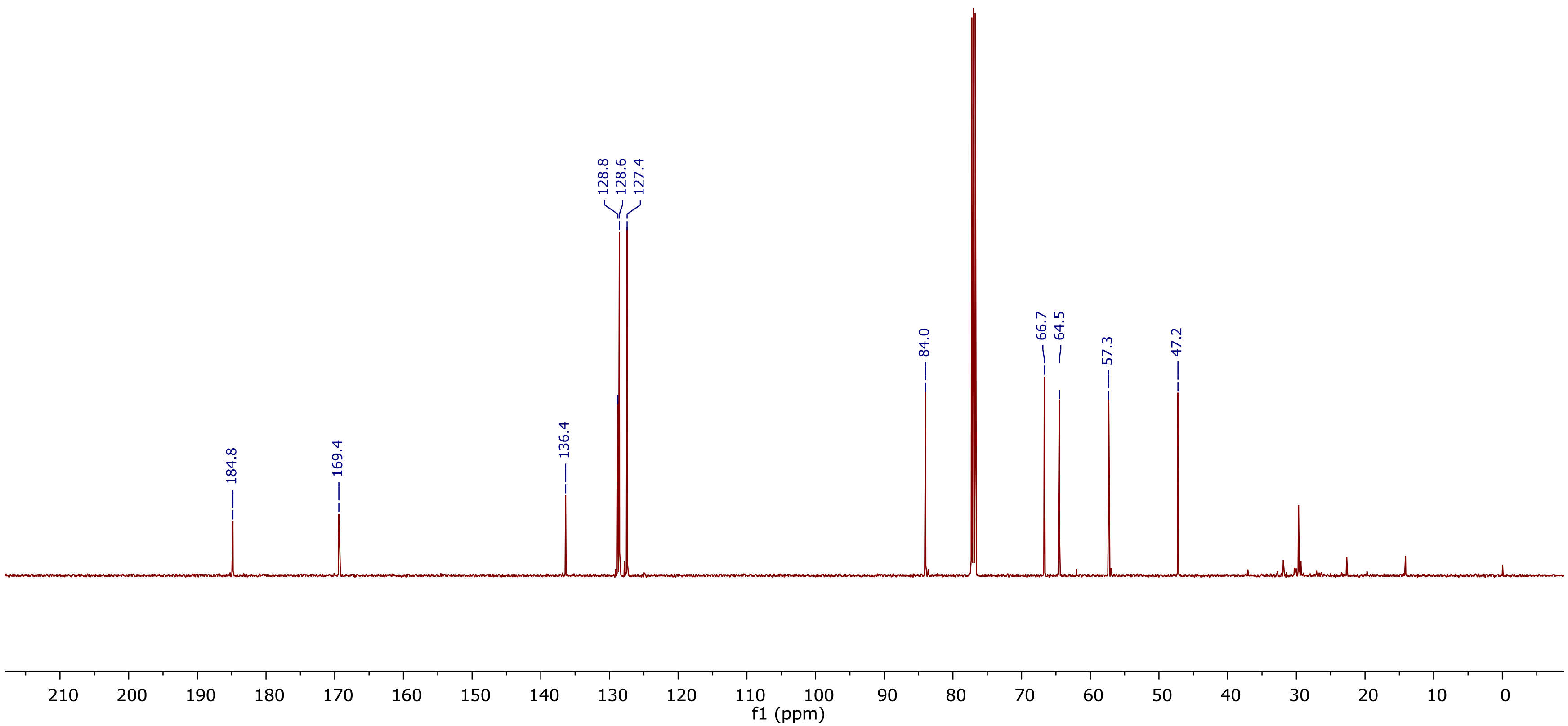

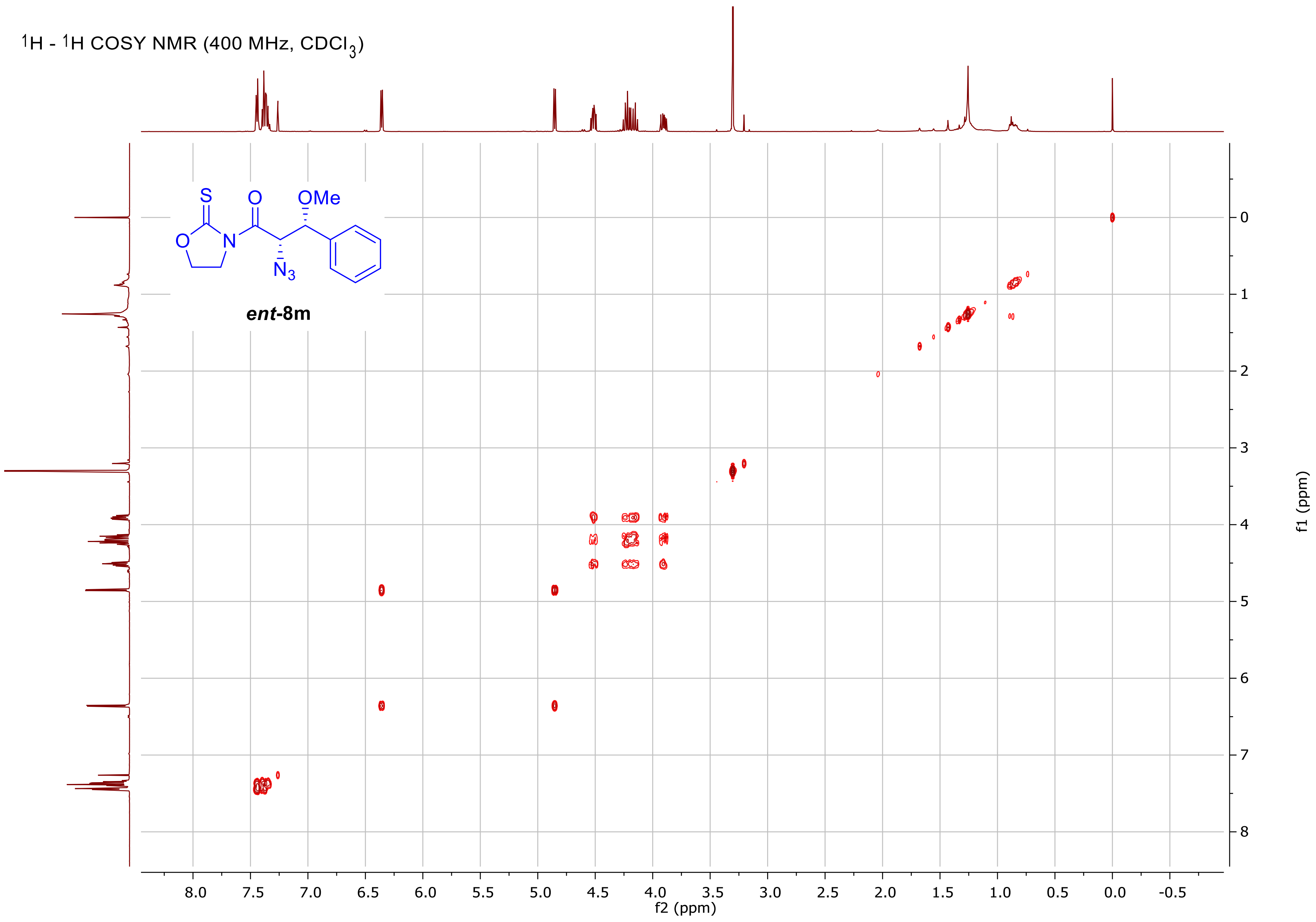

^1H - ^1H COSY NMR (400 MHz, $CDCl_3$)
S
O
OMe
O
N
N_3
ent-8m
f2 (ppm)
f1 (ppm)

^{1}H - ^{13}C HSQC NMR (400 MHz, $CDCl_3$)

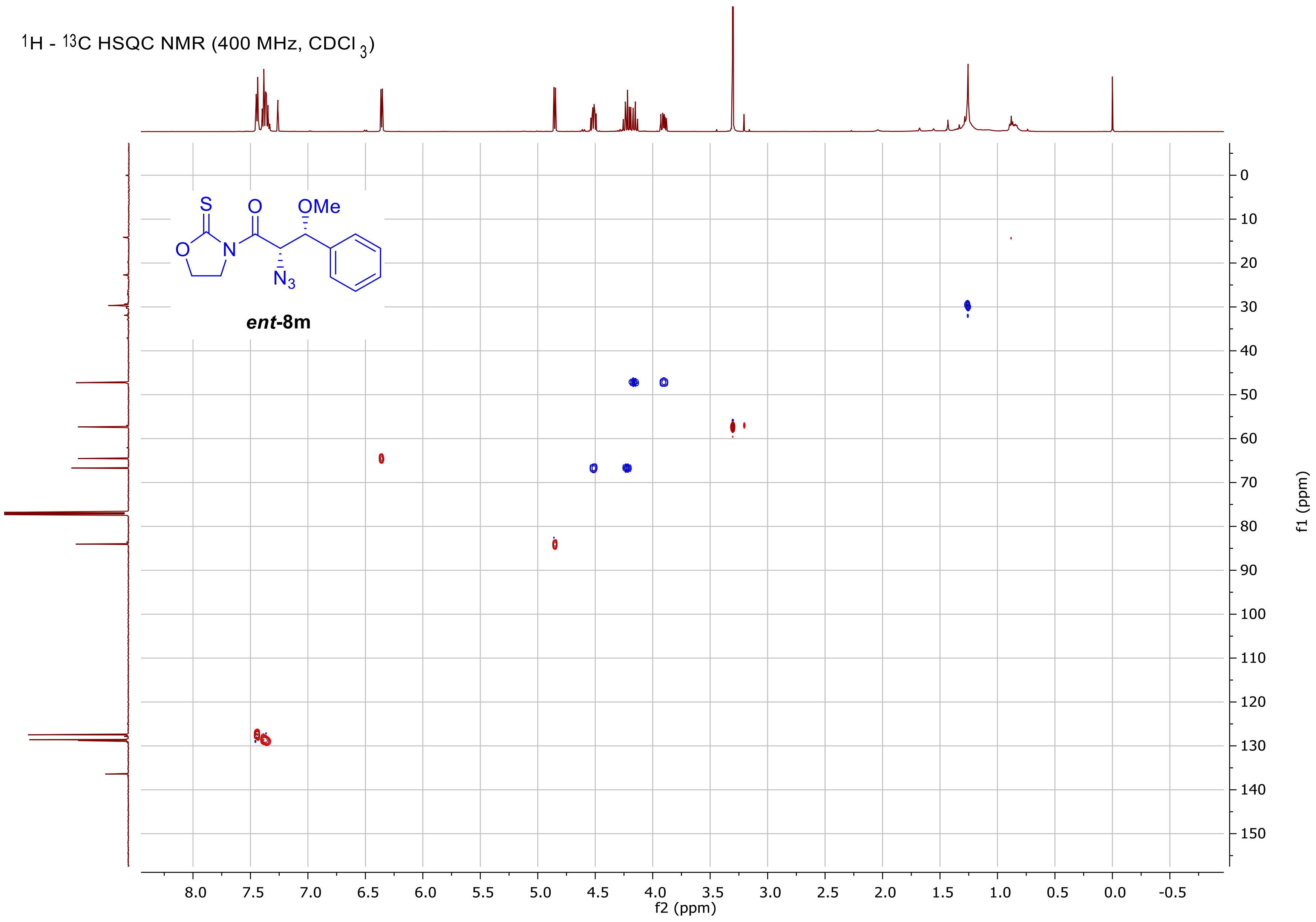

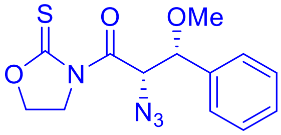

***ent*-8m**

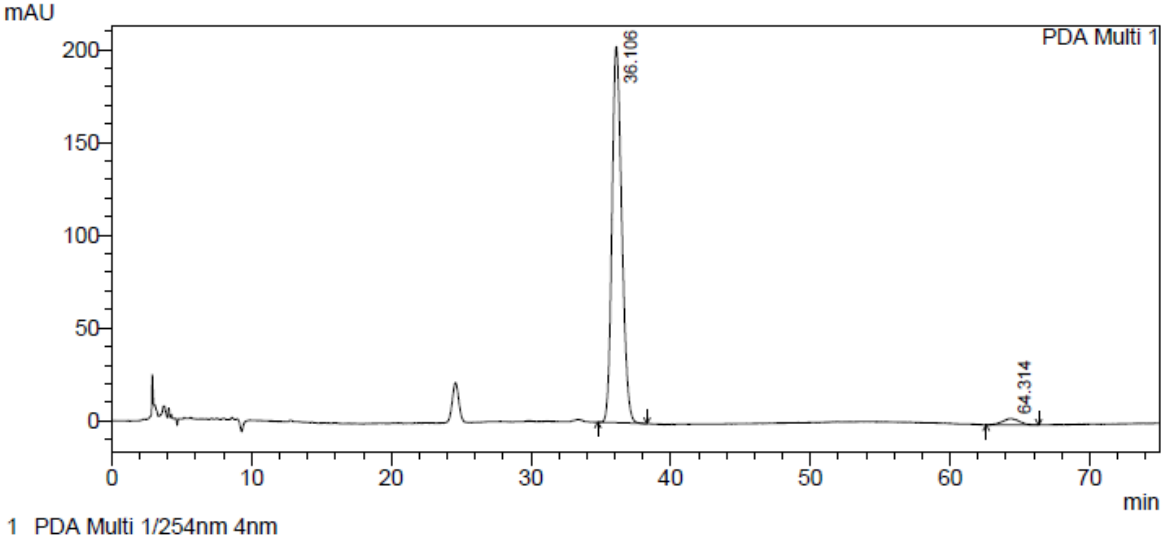

PeakTable

PDA Ch1 254nm 4nm

Peak#	Ret. Time	Area	Height	Area %	Height %
1	36.106	9850684	202449	97.333	98.361
2	64.314	269947	3373	2.667	1.639
Total		10120631	205822	100.000	100.000

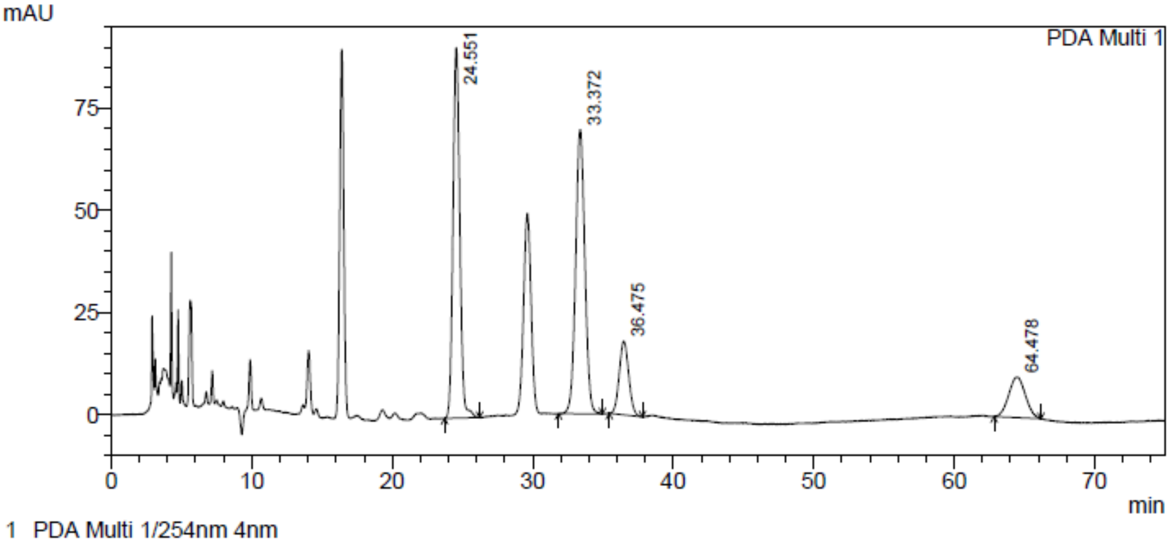

PeakTable

PDA Ch1 254nm 4nm

Peak#	Ret. Time	Area	Height	Area %	Height %
1	24.551	3045261	90719	38.684	48.191
2	33.372	3162891	69552	40.178	36.947
3	36.475	844909	18043	10.733	9.585
4	64.478	819038	9934	10.404	5.277
Total		7872098	188248	100.000	100.000

^{1}H NMR (400 MHz, CDCl$_3$)

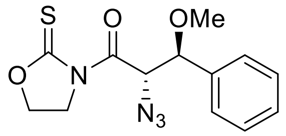

***ent*-6m**

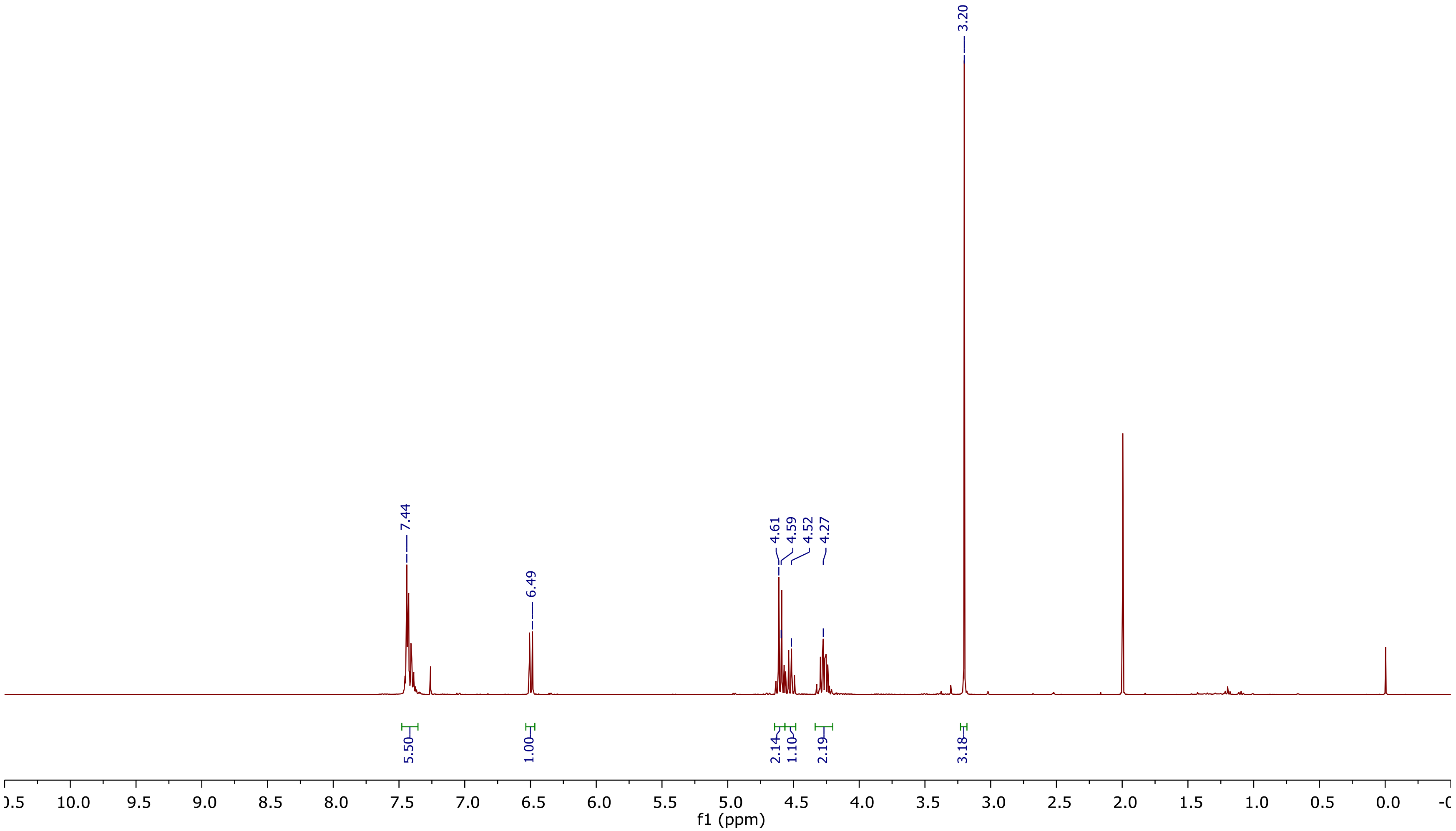

$^{13}C\{^1H\}$ NMR (100.6 MHz, $CDCl_3$)

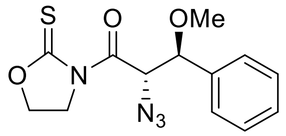

***ent*-6m**

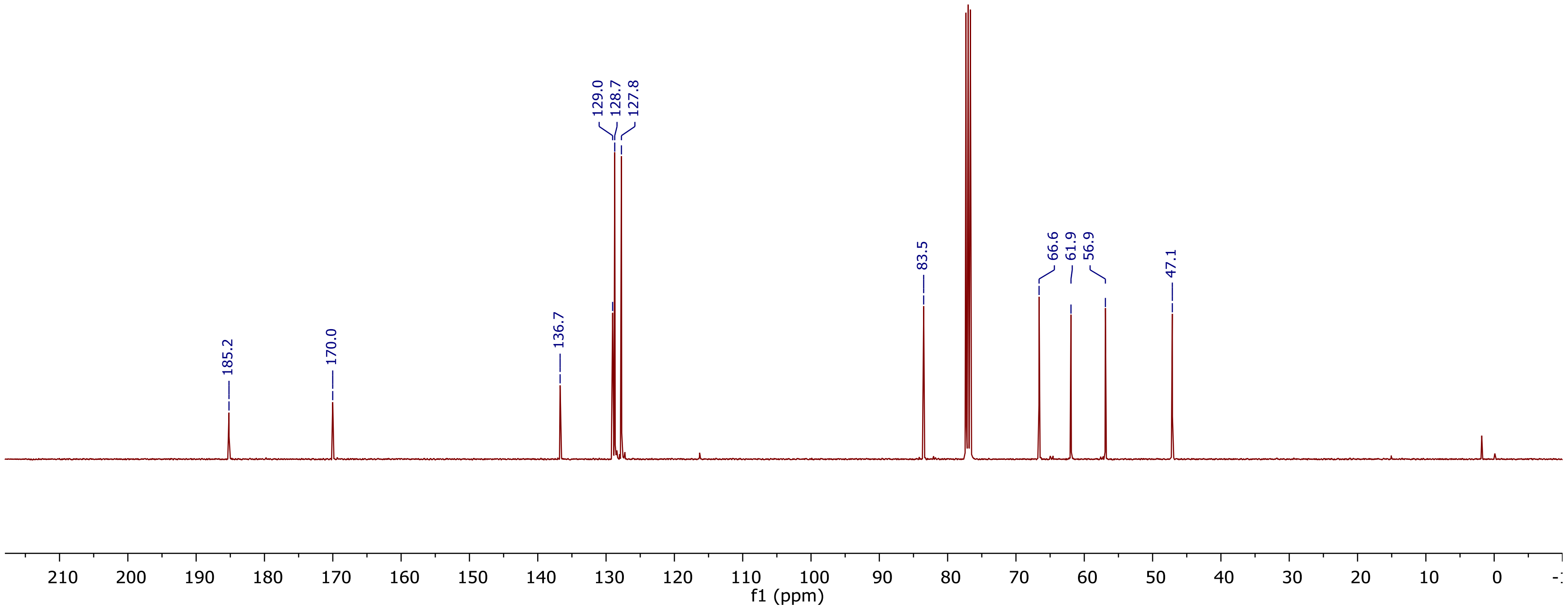

^1H - ^1H COSY NMR (400 MHz, $CDCl_3$)

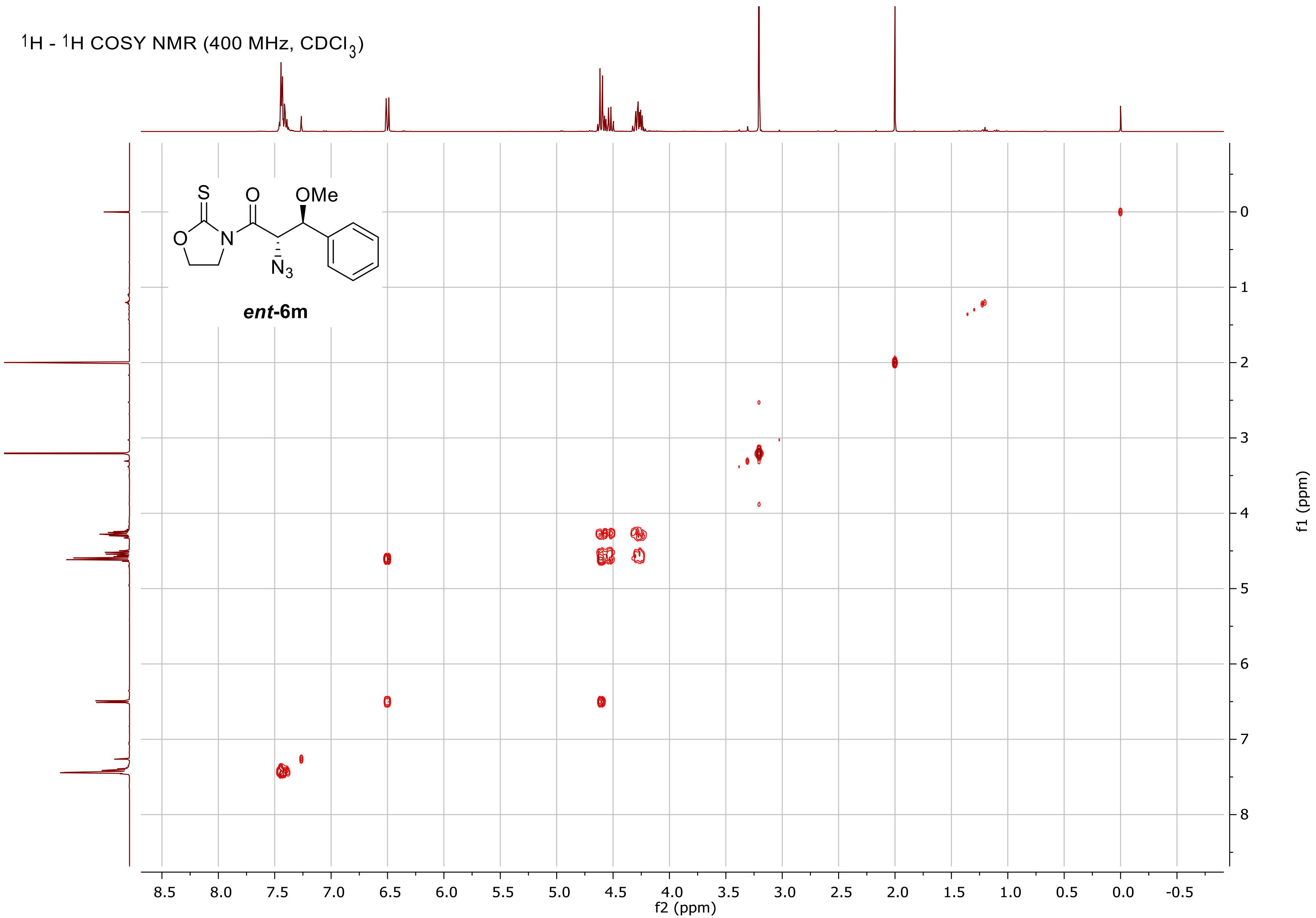

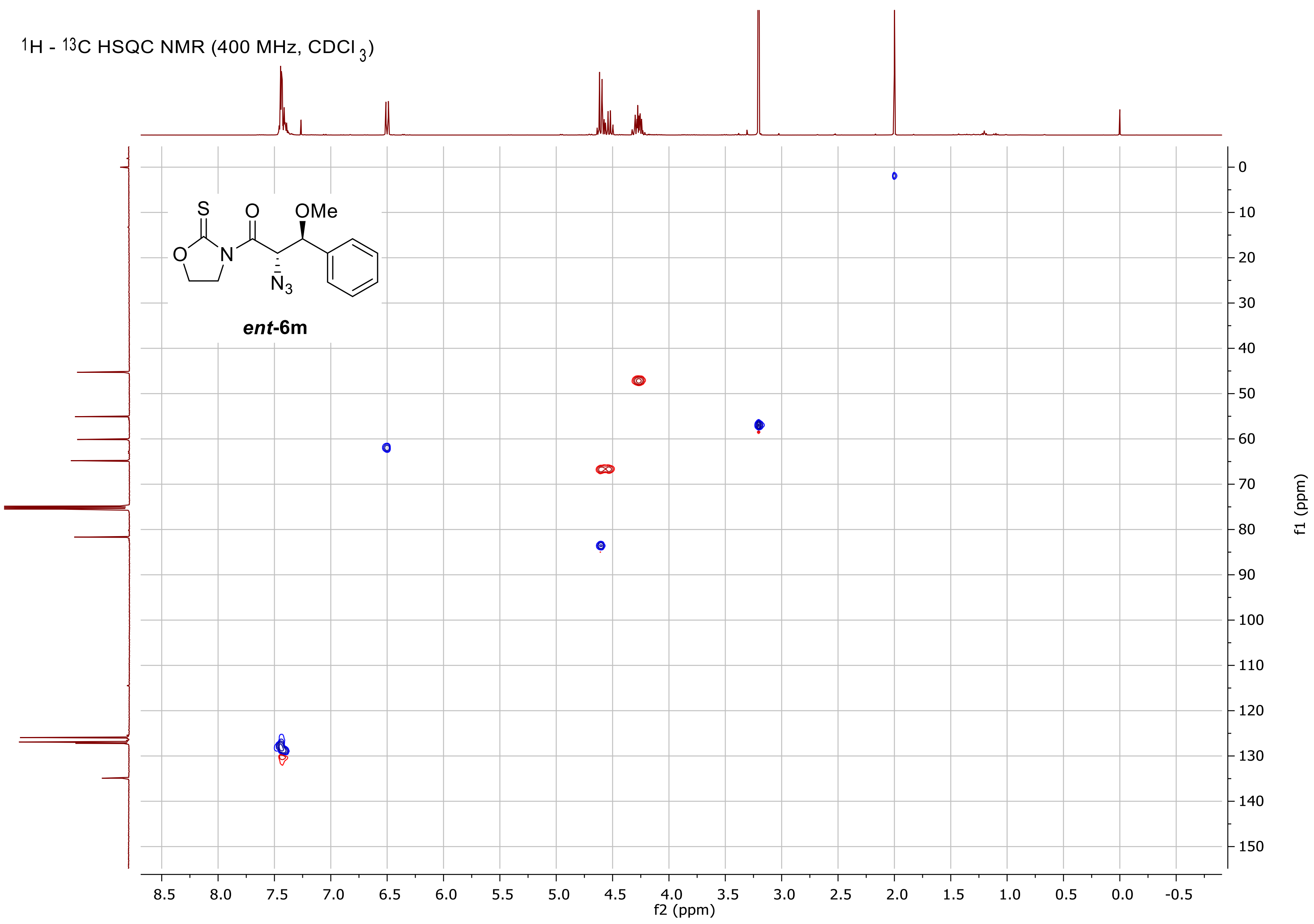

1H - 13C HSQC NMR (400 MHz, CDCl 3)
S
O
OMe
O
N
N3
ent-6m
f2 (ppm)
f1 (ppm)

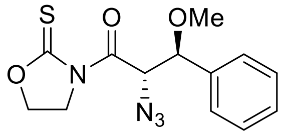

***ent*-6m**

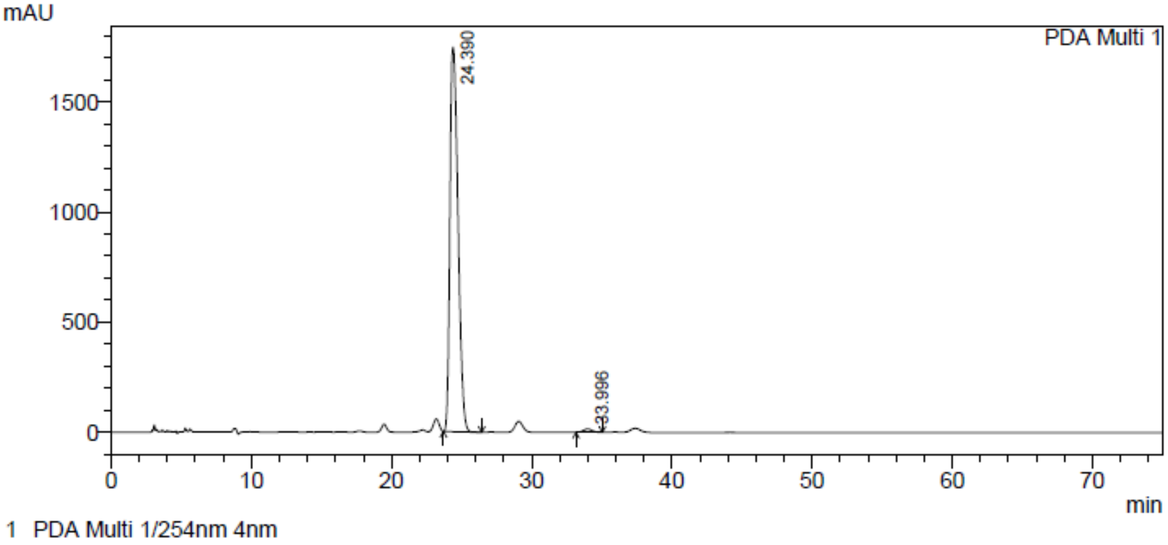

PeakTable

PDA Ch1 254nm 4nm

Peak#	Ret. Time	Area	Height	Area %	Height %
1	24.390	70016702	1746503	99.080	99.141
2	33.996	649962	15139	0.920	0.859
Total		70666664	1761642	100.000	100.000

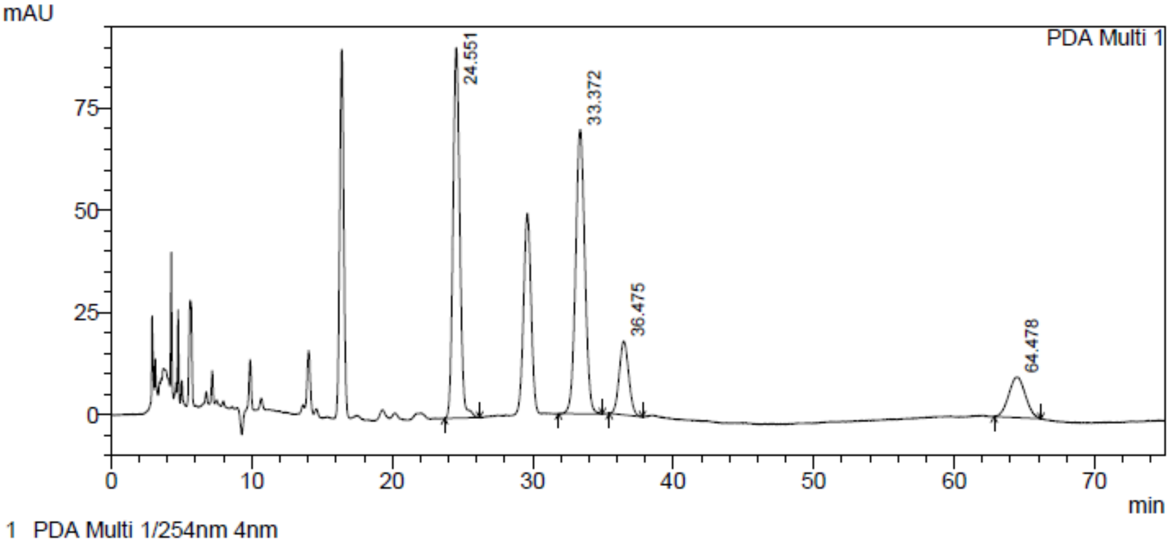

PeakTable

PDA Ch1 254nm 4nm

Peak#	Ret. Time	Area	Height	Area %	Height %
1	24.551	3045261	90719	38.684	48.191
2	33.372	3162891	69552	40.178	36.947
3	36.475	844909	18043	10.733	9.585
4	64.478	819038	9934	10.404	5.277
Total		7872098	188248	100.000	100.000

^{1}H NMR (400 MHz, $CDCl_3$)

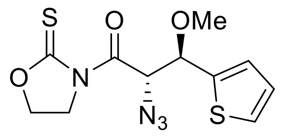

***ent*-6n**

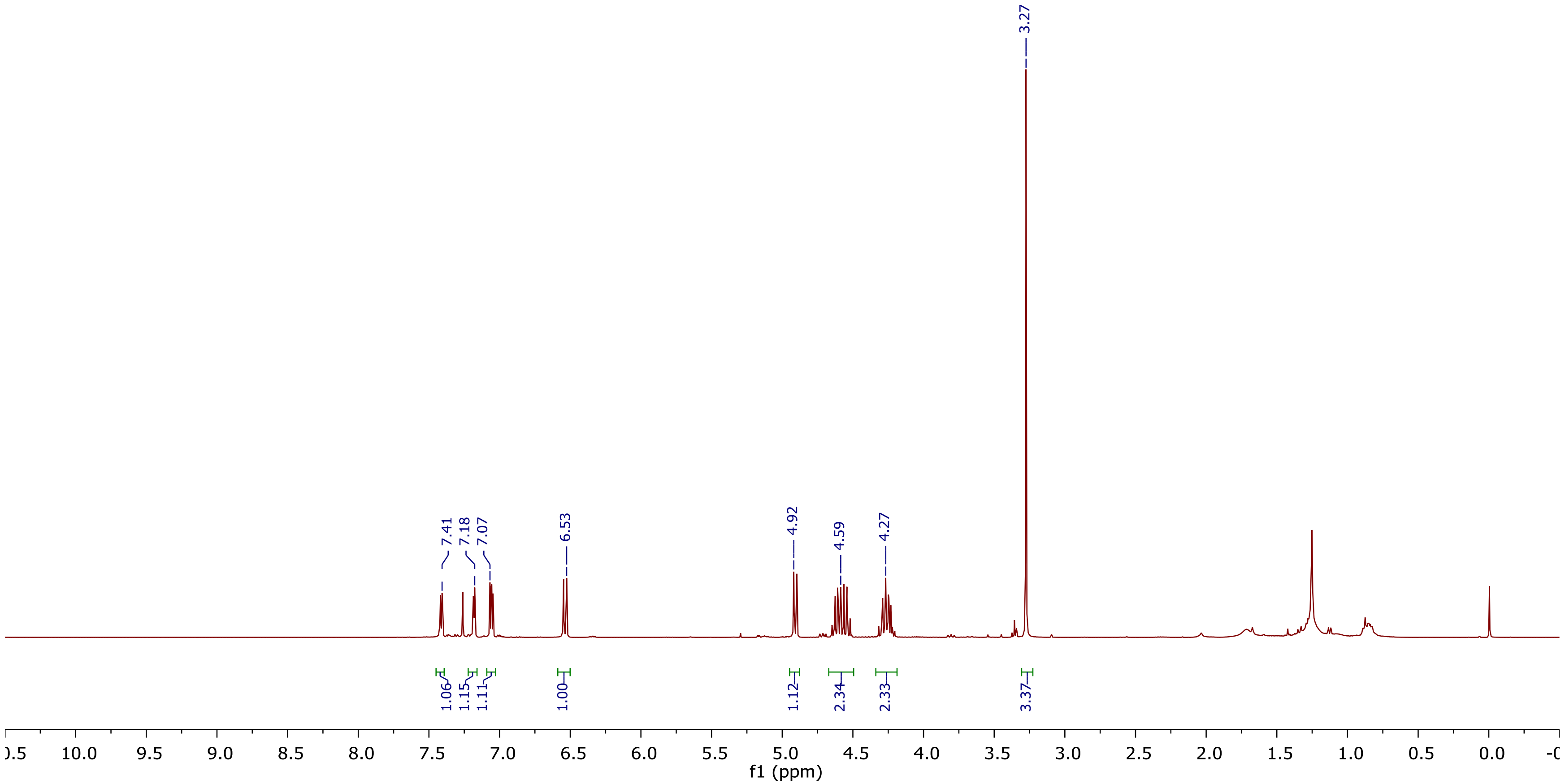

$^{13}C\{^1H\}$ NMR (100.6 MHz, $CDCl_3$)

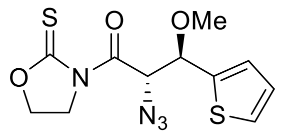

***ent*-6n**

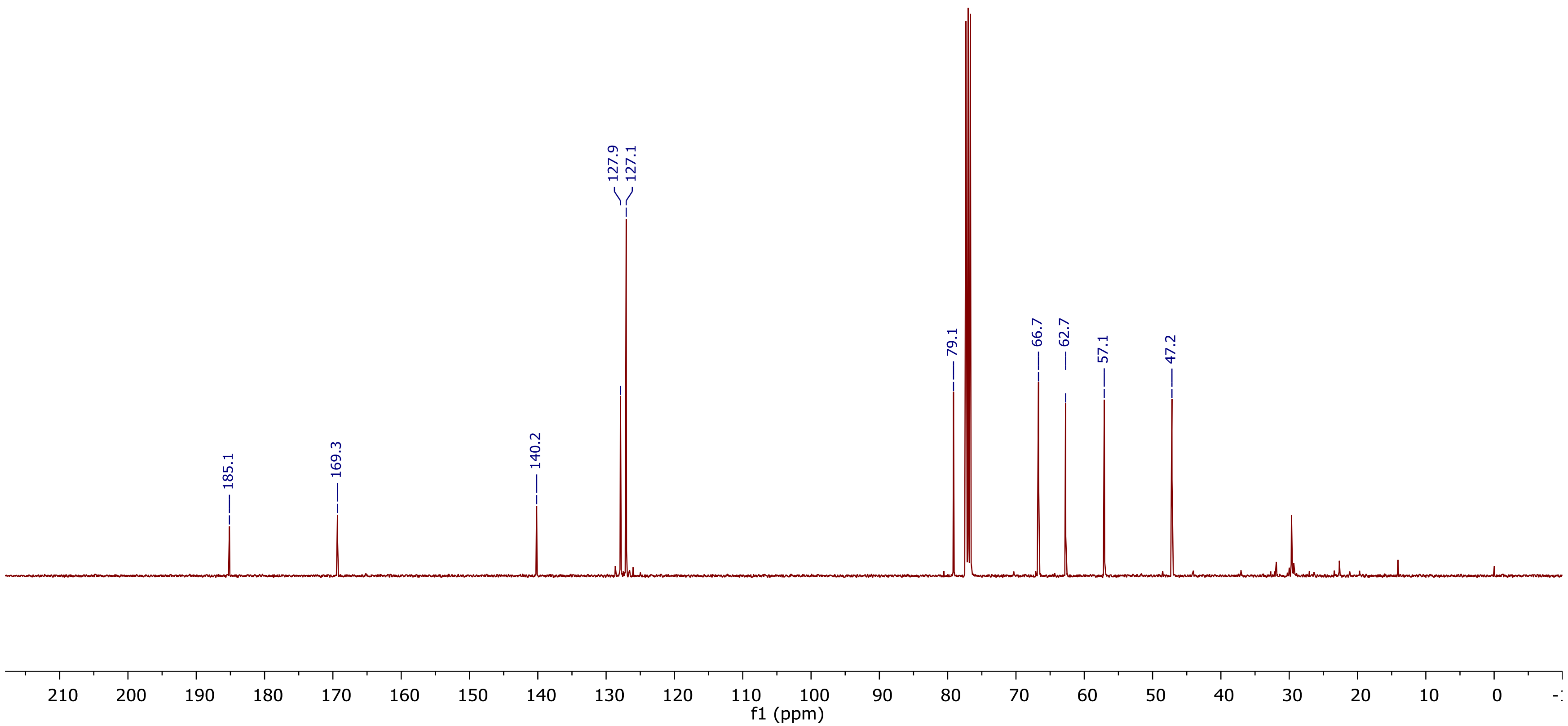

^{1}H - ^{1}H COSY NMR (400 MHz, $CDCl_3$)

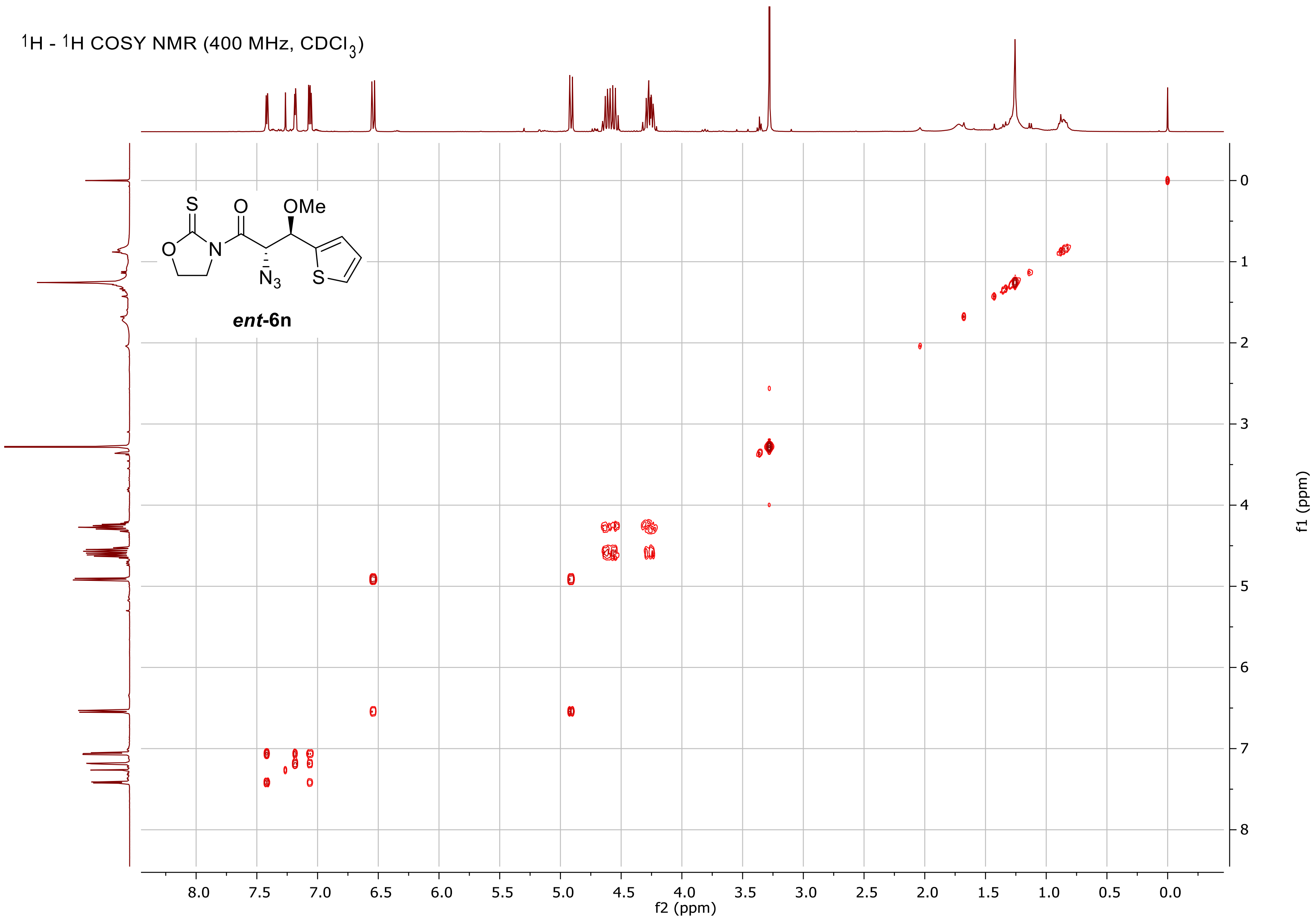

***ent*-6n**

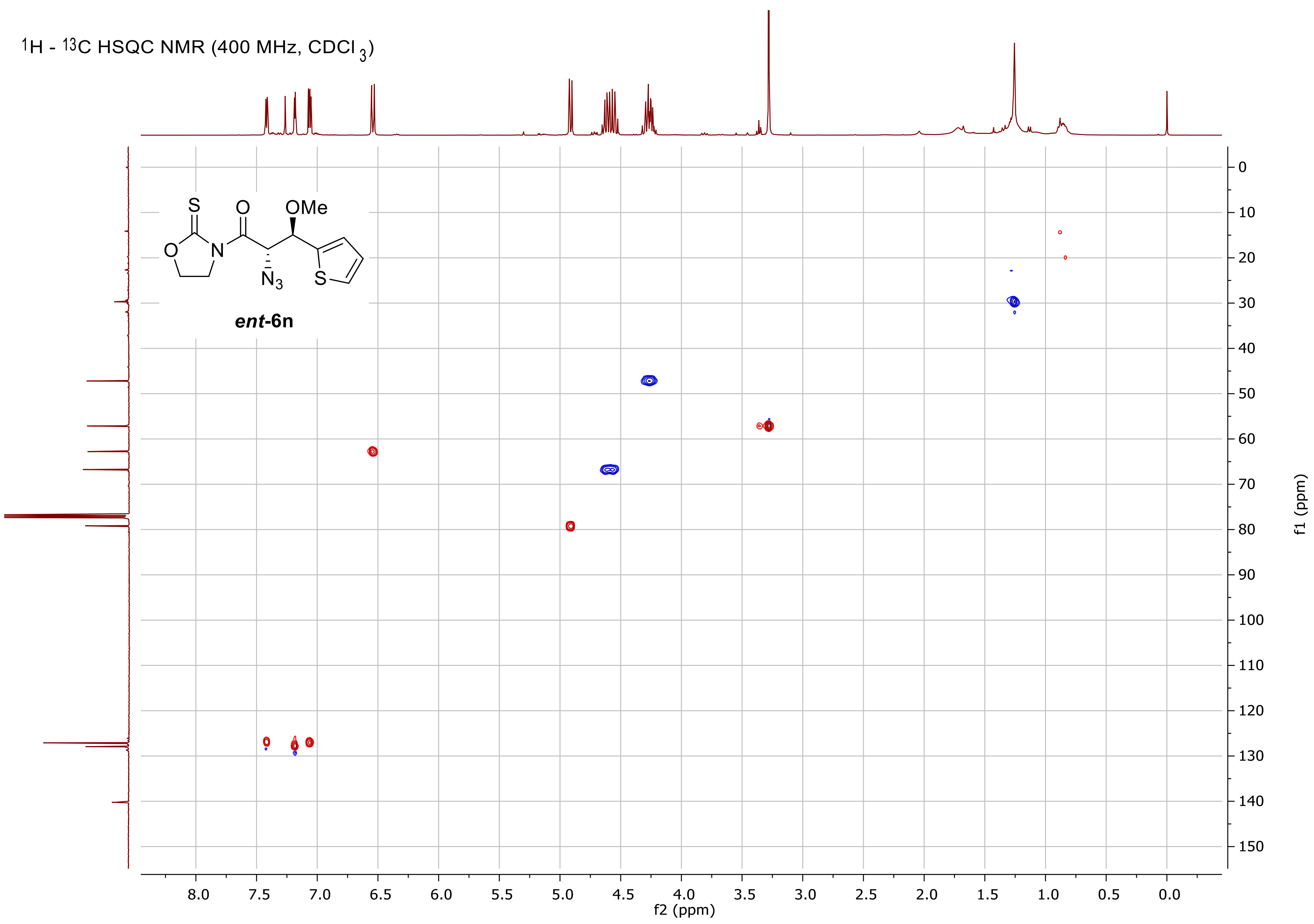

^1H - ^{13}C HSQC NMR (400 MHz, $CDCl_3$)
S
O
OMe
O
N
N_3
S
ent-**6n**
f2 (ppm)
f1 (ppm)

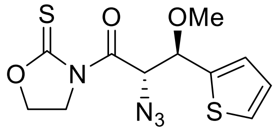

***ent*-6n**

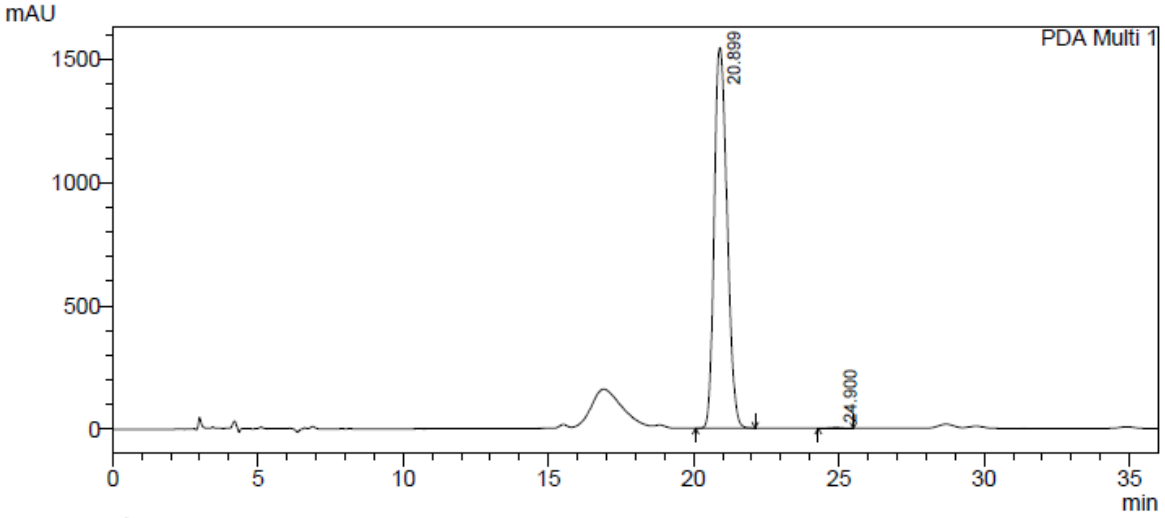

1 PDA Multi 1/254nm 4nm

PeakTable

PDA Ch1 254nm 4nm

Peak#	Ret. Time	Area	Height	Area %	Height %
1	20.899	47480388	1543338	99.813	99.818
2	24.900	88840	2820	0.187	0.182
Total		47569227	1546158	100.000	100.000

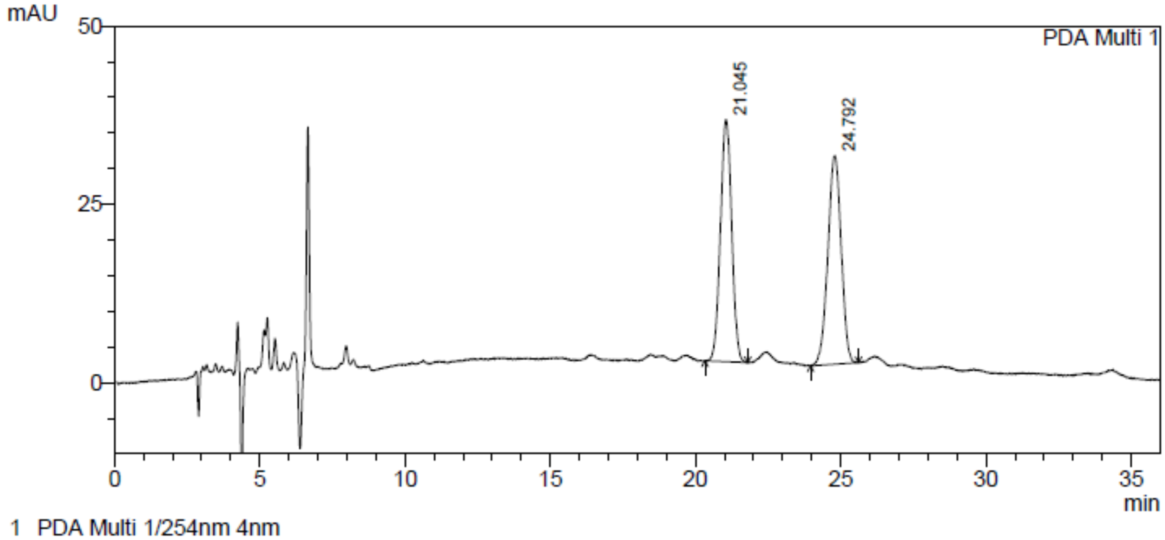

1 PDA Multi 1/254nm 4nm

PeakTable

PDA Ch1 254nm 4nm

Peak#	Ret. Time	Area	Height	Area %	Height %
1	21.045	916608	33929	49.520	53.732
2	24.792	934395	29217	50.480	46.268
Total		1851003	63146	100.000	100.000

^{1}H NMR (400 MHz, $CDCl_3$)

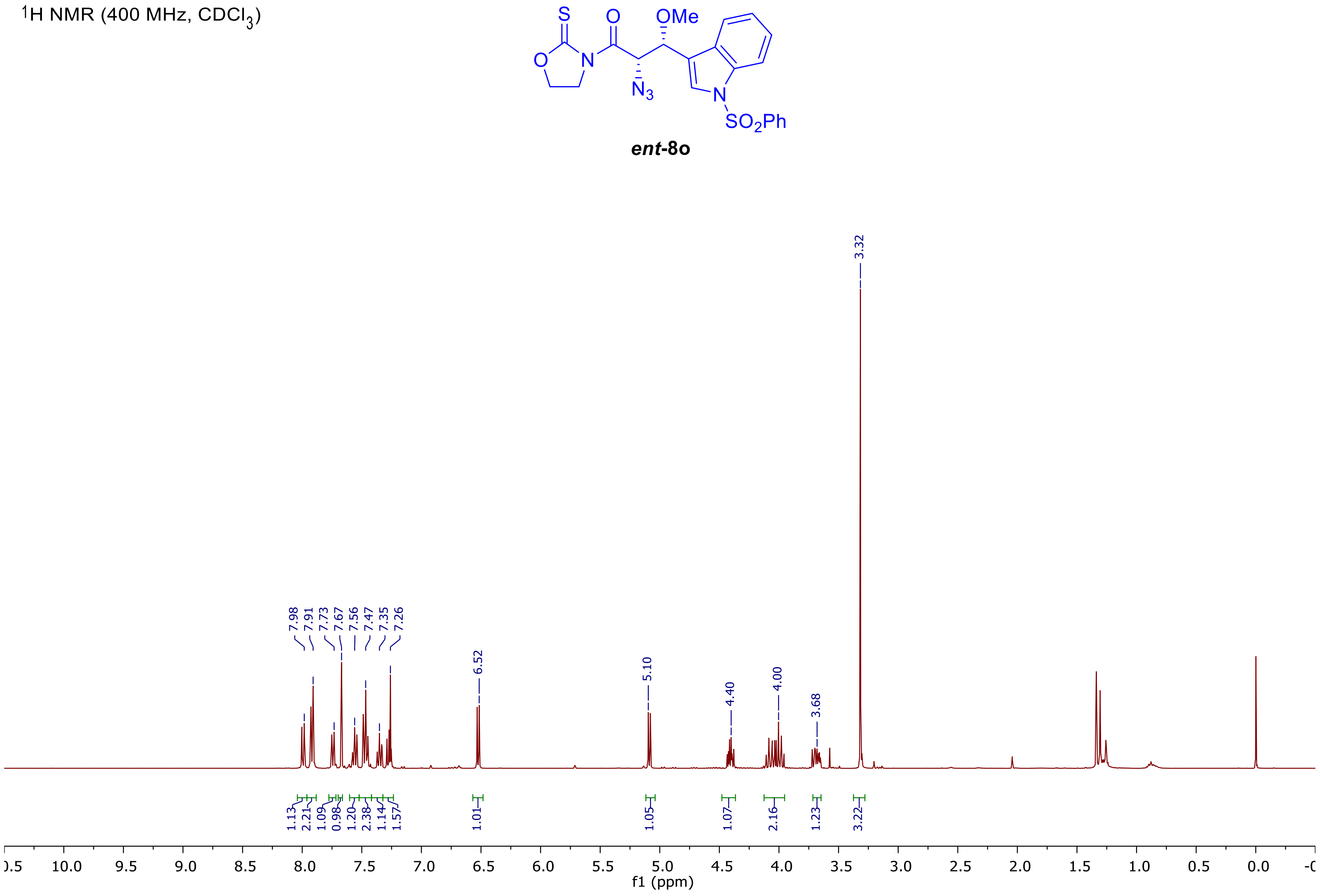

$^{13}C\{^{1}H\}$ NMR (100.6 MHz, $CDCl_3$)

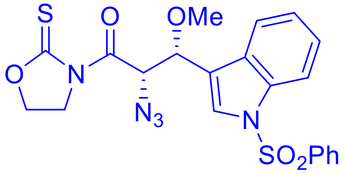

***ent*-8o**

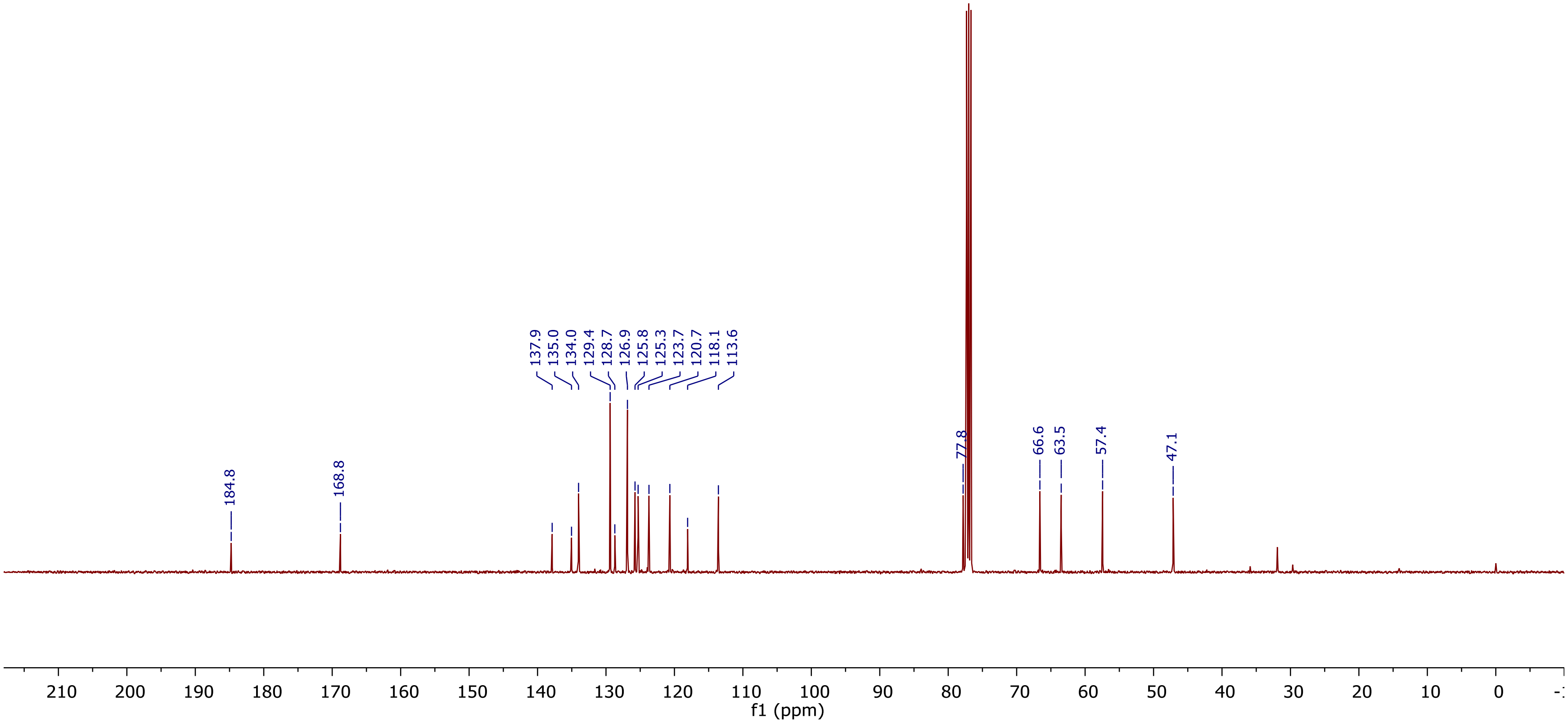

¹H - ¹H COSY NMR (400 MHz, $CDCl_3$)

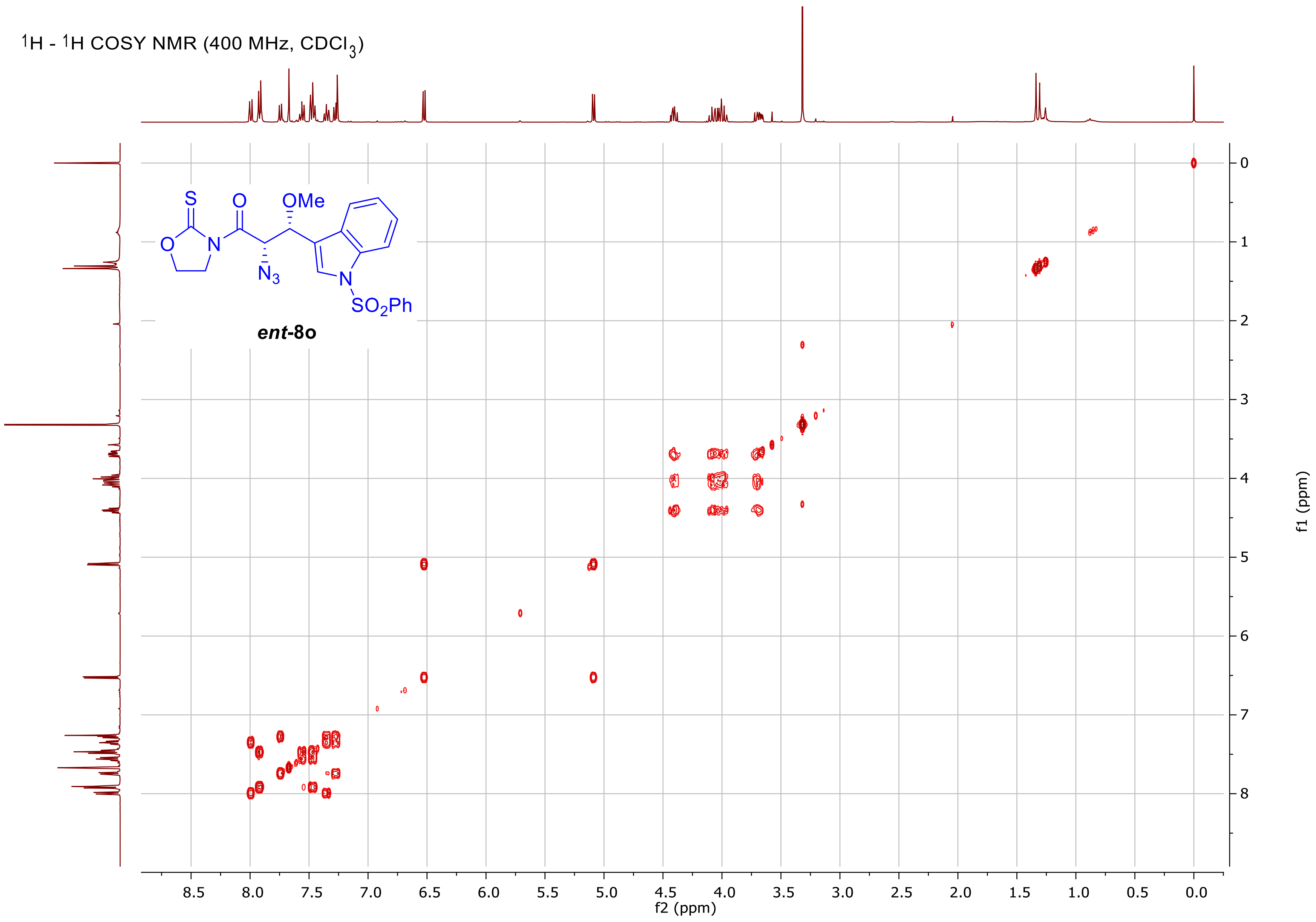

^{1}H - ^{13}C HSQC NMR (400 MHz, $CDCl_3$)

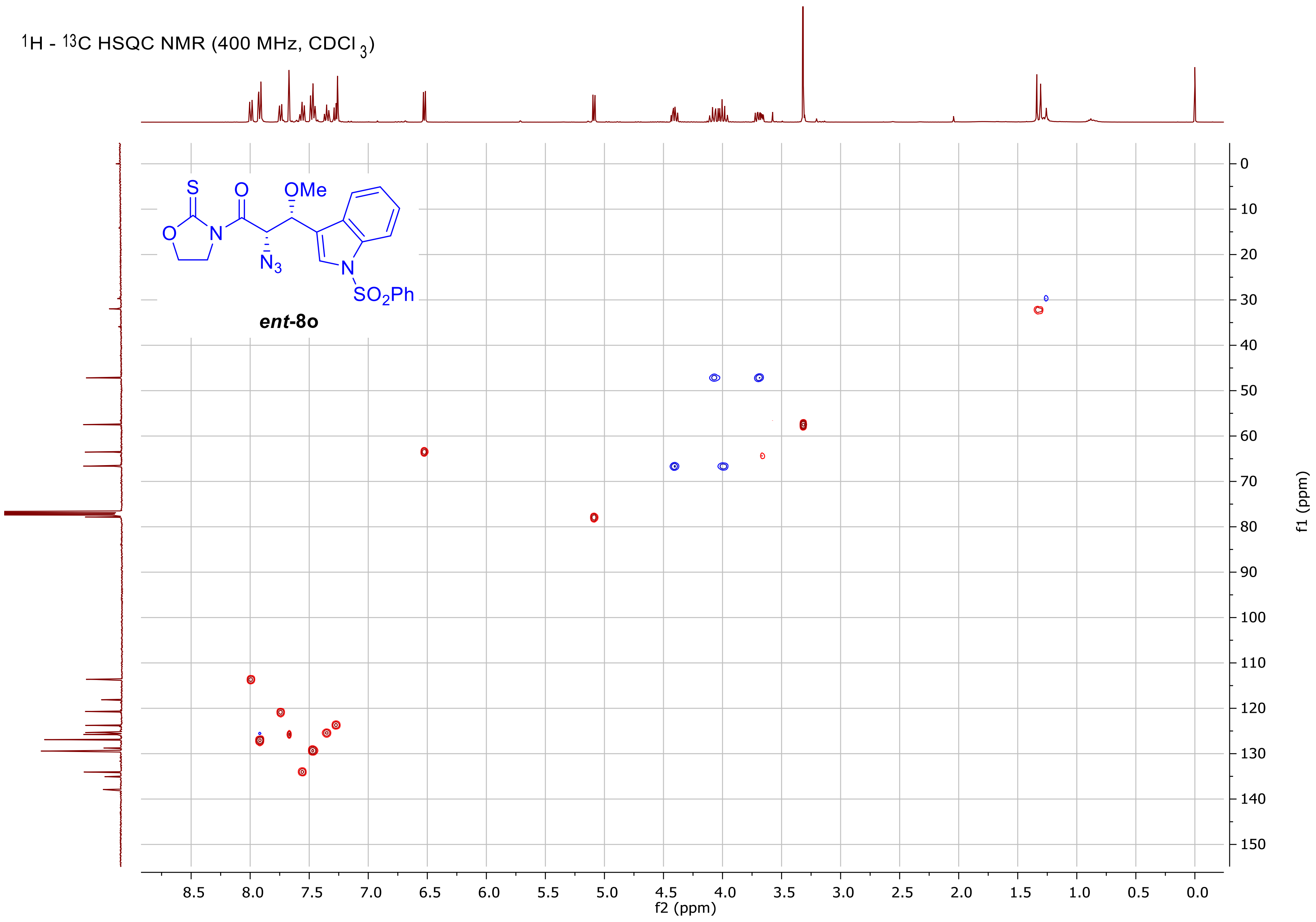

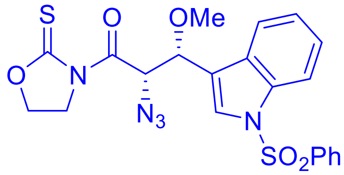

***ent*-8o**

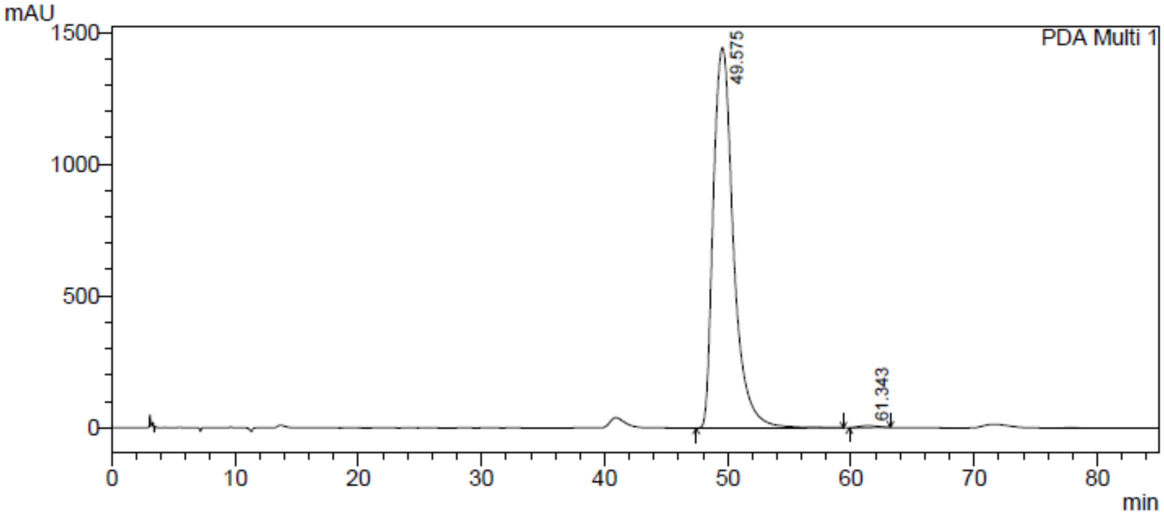

1 PDA Multi 1/254nm 4nm

PeakTable

PDA Ch1 254nm 4nm

Peak#	Ret. Time	Area	Height	Area %	Height %
1	49.575	163844936	1444729	99.558	99.508
2	61.343	727943	7147	0.442	0.492
Total		164572879	1451875	100.000	100.000

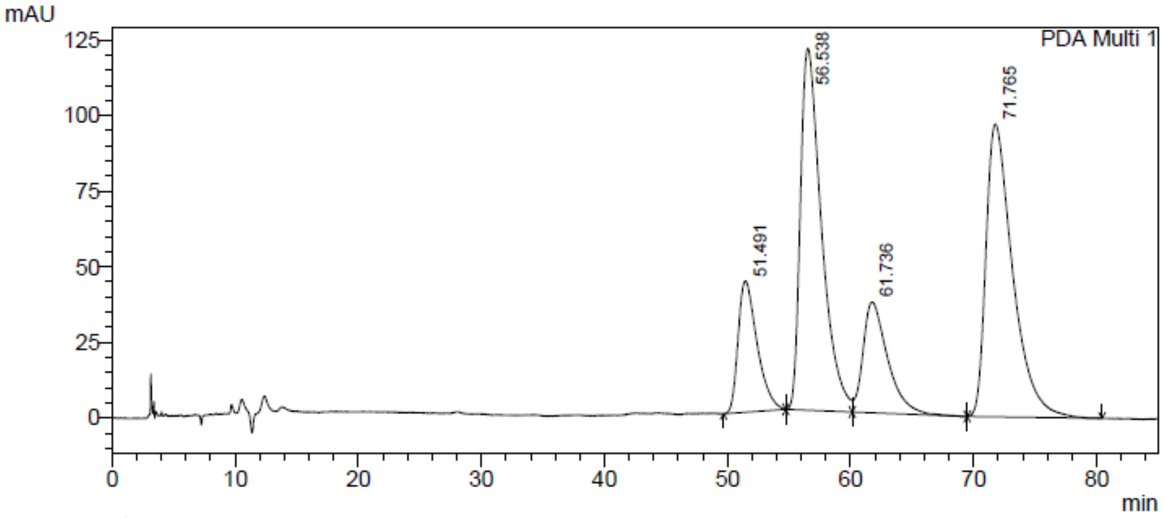

1 PDA Multi 1/254nm 4nm

PeakTable

PDA Ch1 254nm 4nm

Peak#	Ret. Time	Area	Height	Area %	Height %
1	51.491	4655842	43427	12.041	14.639
2	56.538	14271938	119703	36.910	40.351
3	61.736	5099313	36639	13.188	12.351
4	71.765	14640093	96889	37.862	32.660
Total		38667185	296658	100.000	100.000

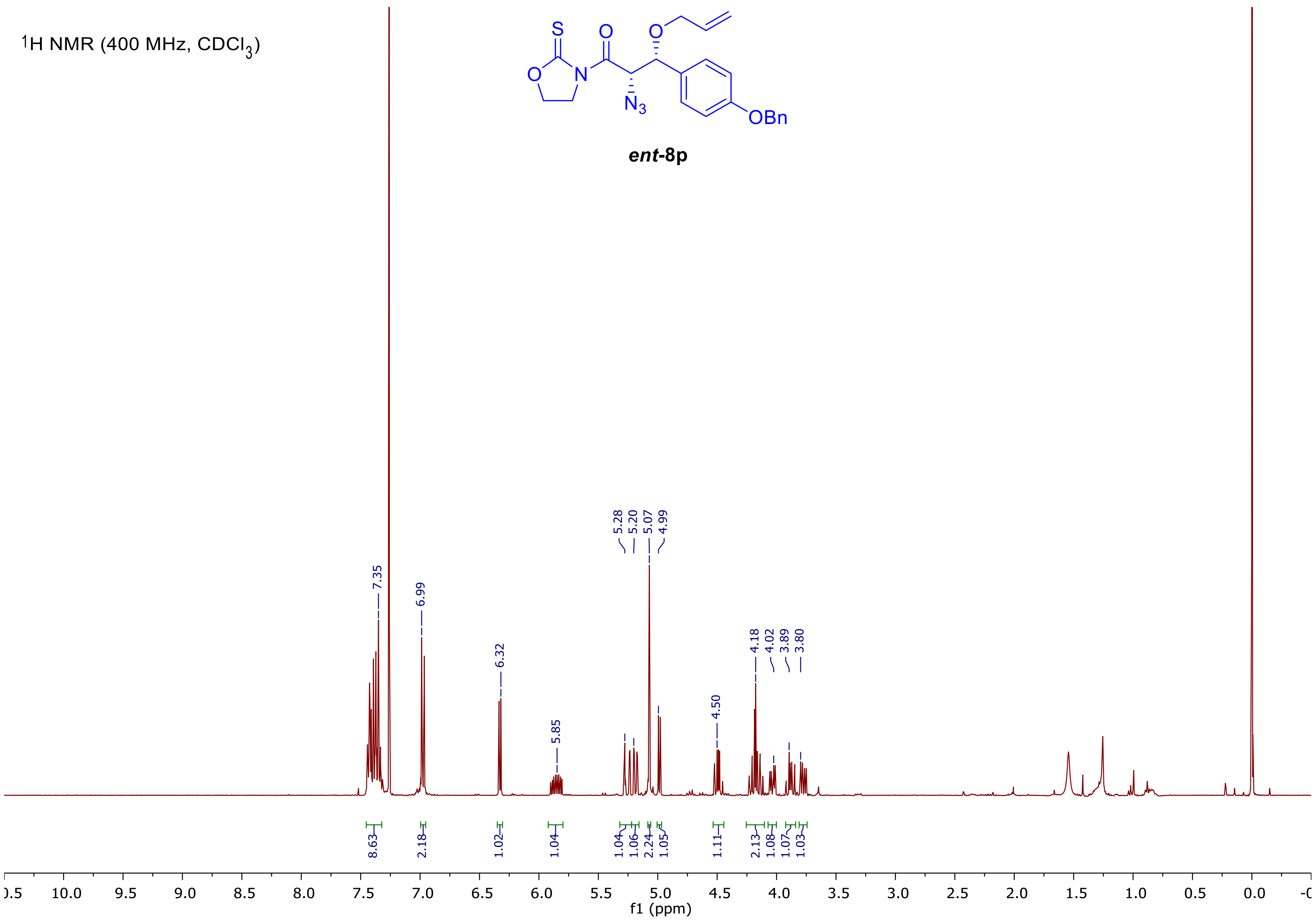

¹H NMR (400 MHz, CDCl3)
S
O
O
N
O
N3
OBn
ent-8p
7.35
6.99
6.32
5.85
5.28
5.20
5.07
4.99
4.50
4.18
4.02
3.89
3.80
8.63
2.18
1.02
1.04
1.04
1.06
2.24
1.05
1.11
2.13
1.08
1.07
1.03
f1 (ppm)

$^{13}C\{^1H\}$ NMR (100.6 MHz, $CDCl_3$)

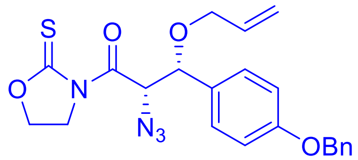

***ent*-8p**

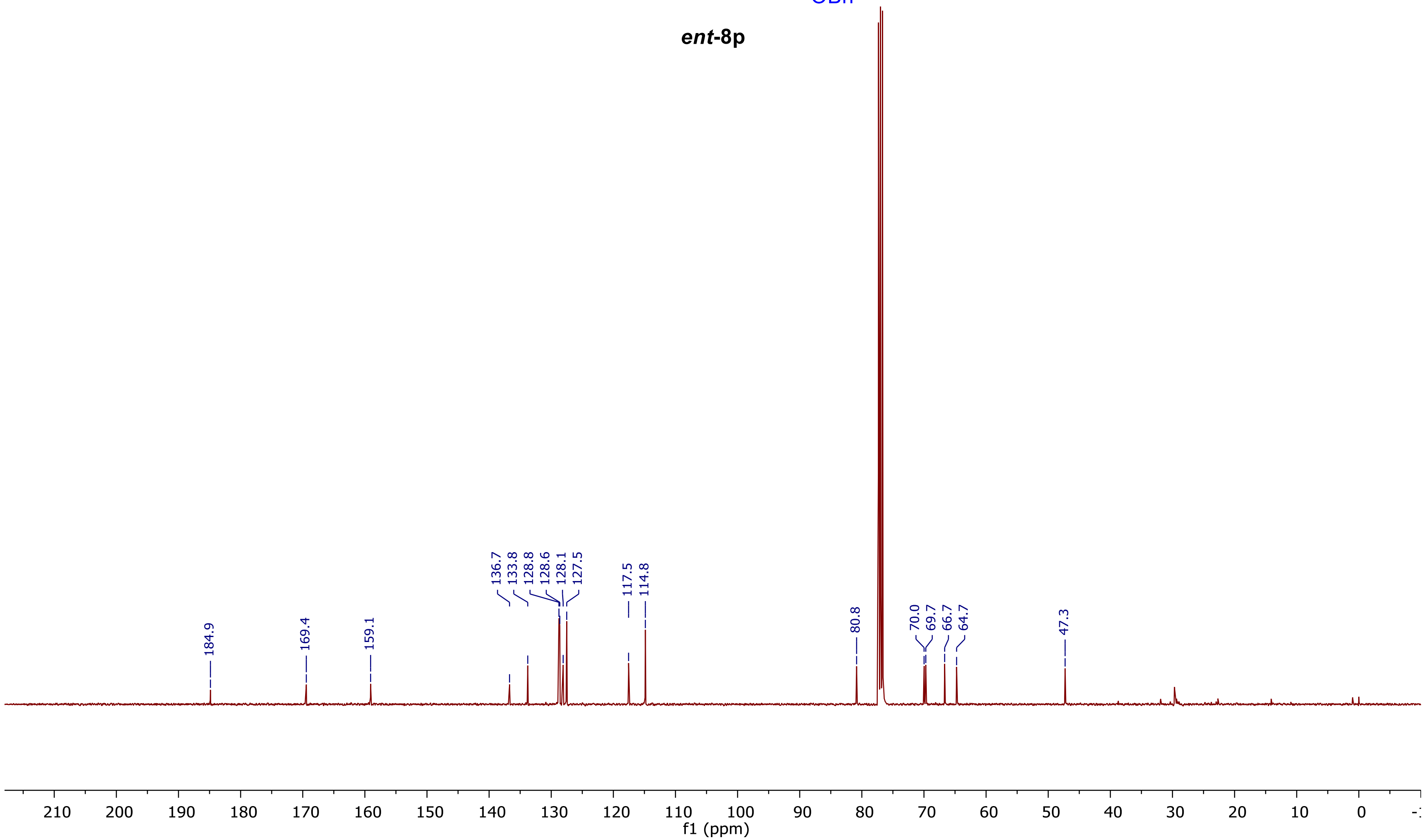

^1H - ^1H COSY NMR (400 MHz, $CDCl_3$)

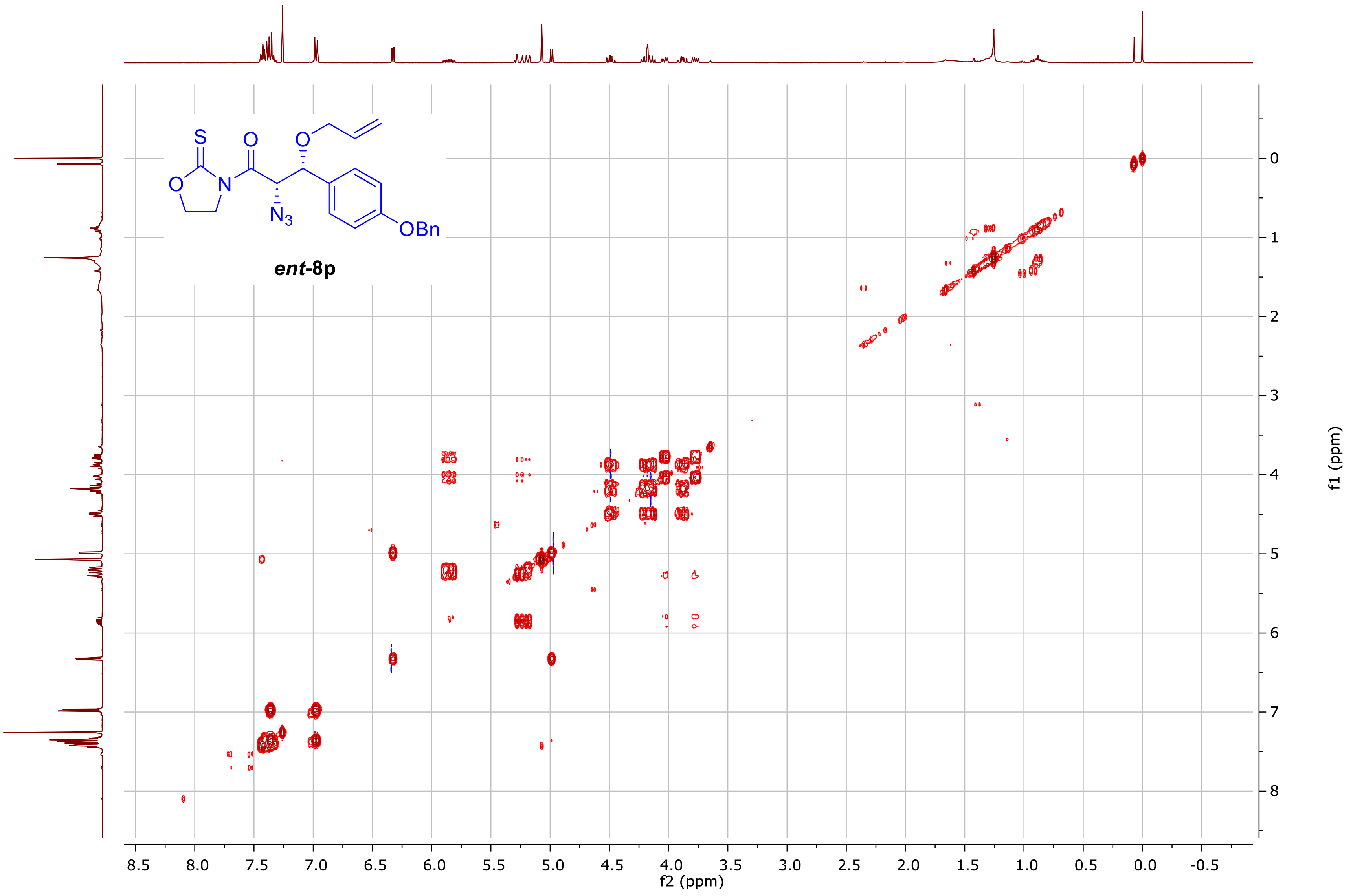

^{1}H - ^{13}C HSQC NMR (400 MHz, $CDCl_3$)

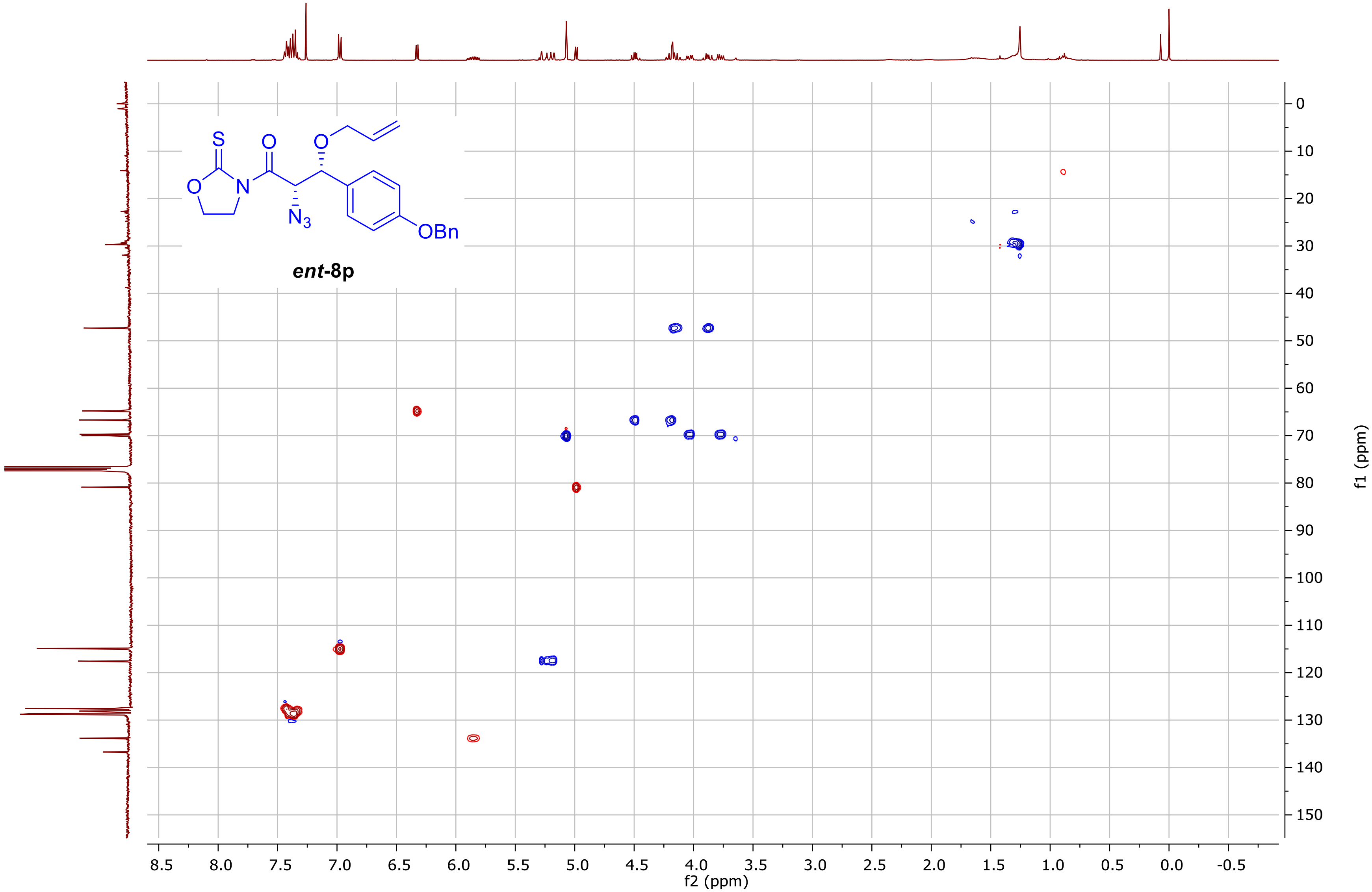

^1H NMR (400 MHz, $CDCl_3$)

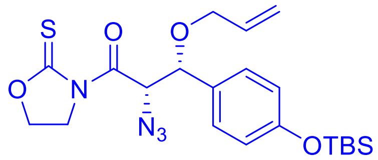

ent-**8q**

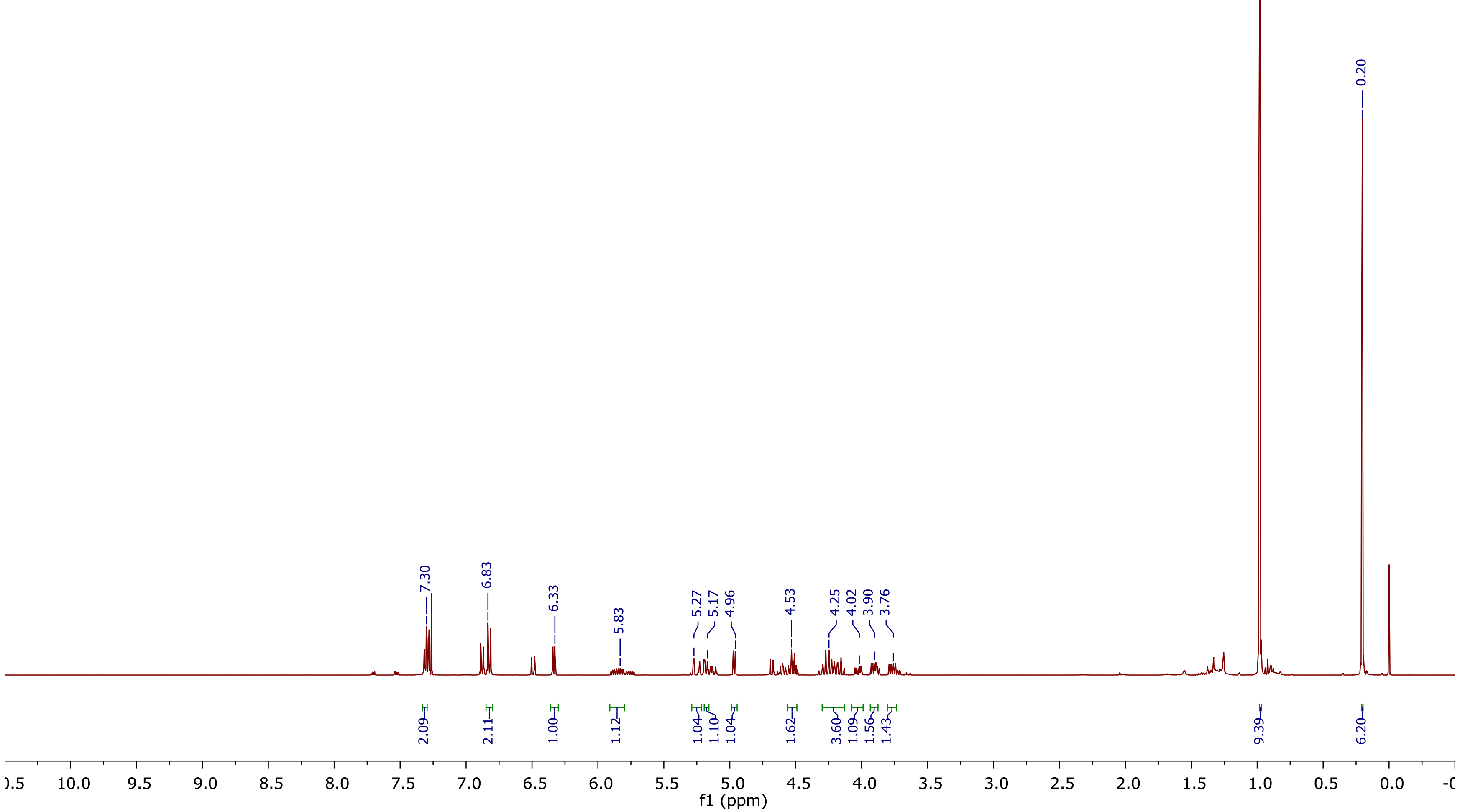

13C{1H} NMR (100.6 MHz, CDCl$_3$)

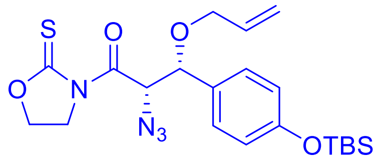

ent-**8q**

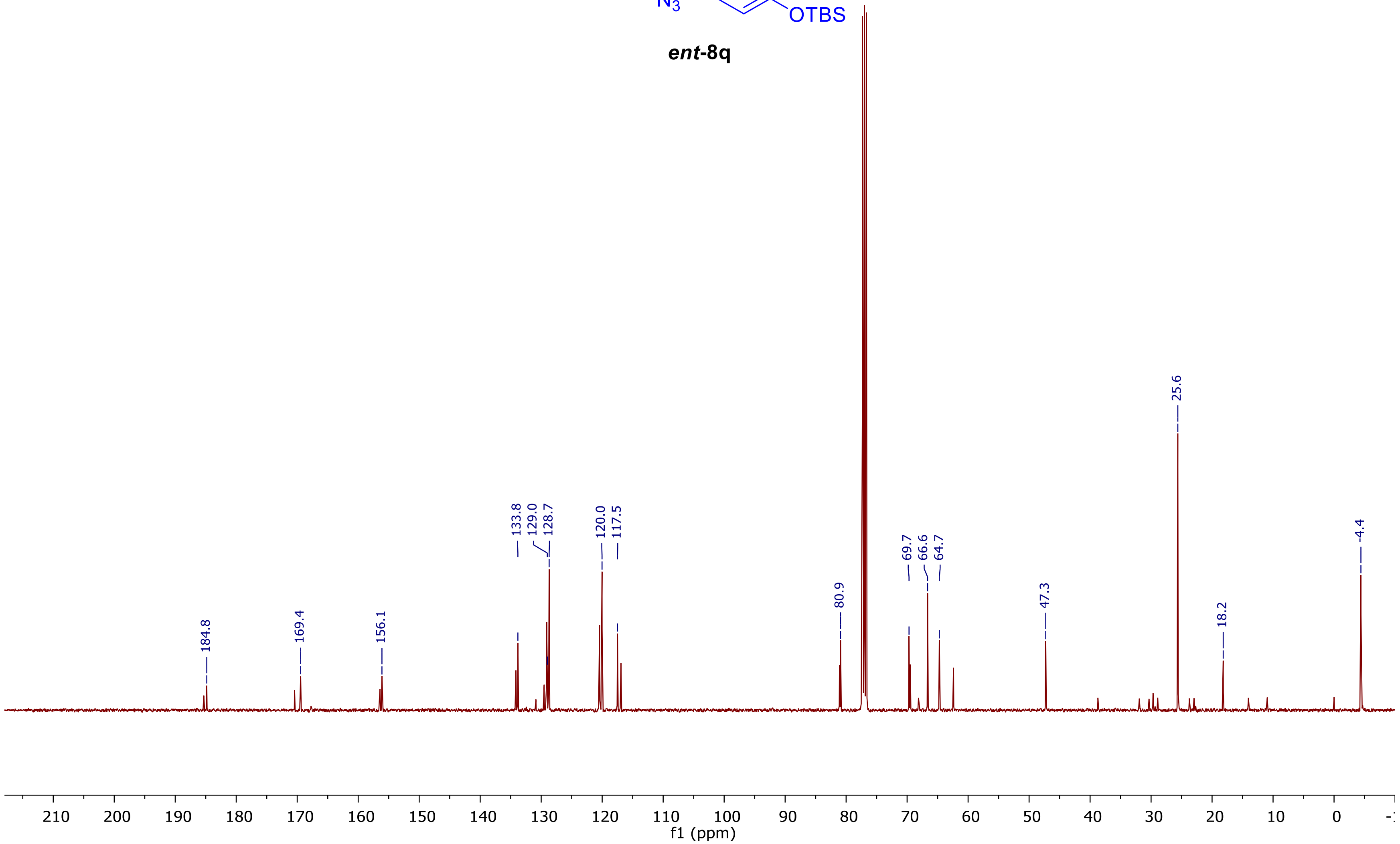

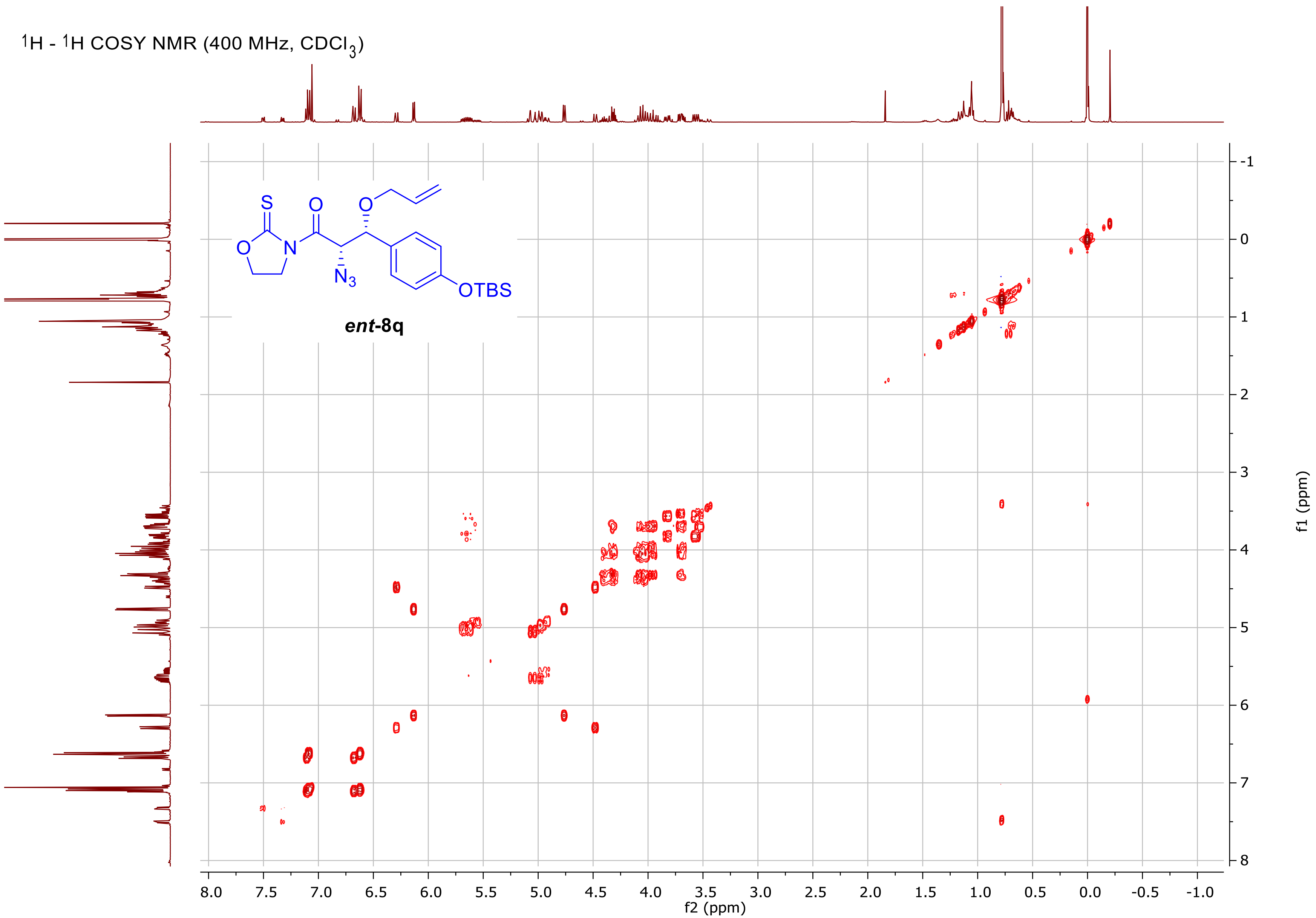

1H - 1H COSY NMR (400 MHz, CDCl3)
S
O
O
N
O
N3
OTBS
ent-8q
f1 (ppm)
f2 (ppm)

^1H - ^{13}C HSQC NMR (400 MHz, $CDCl_3$)

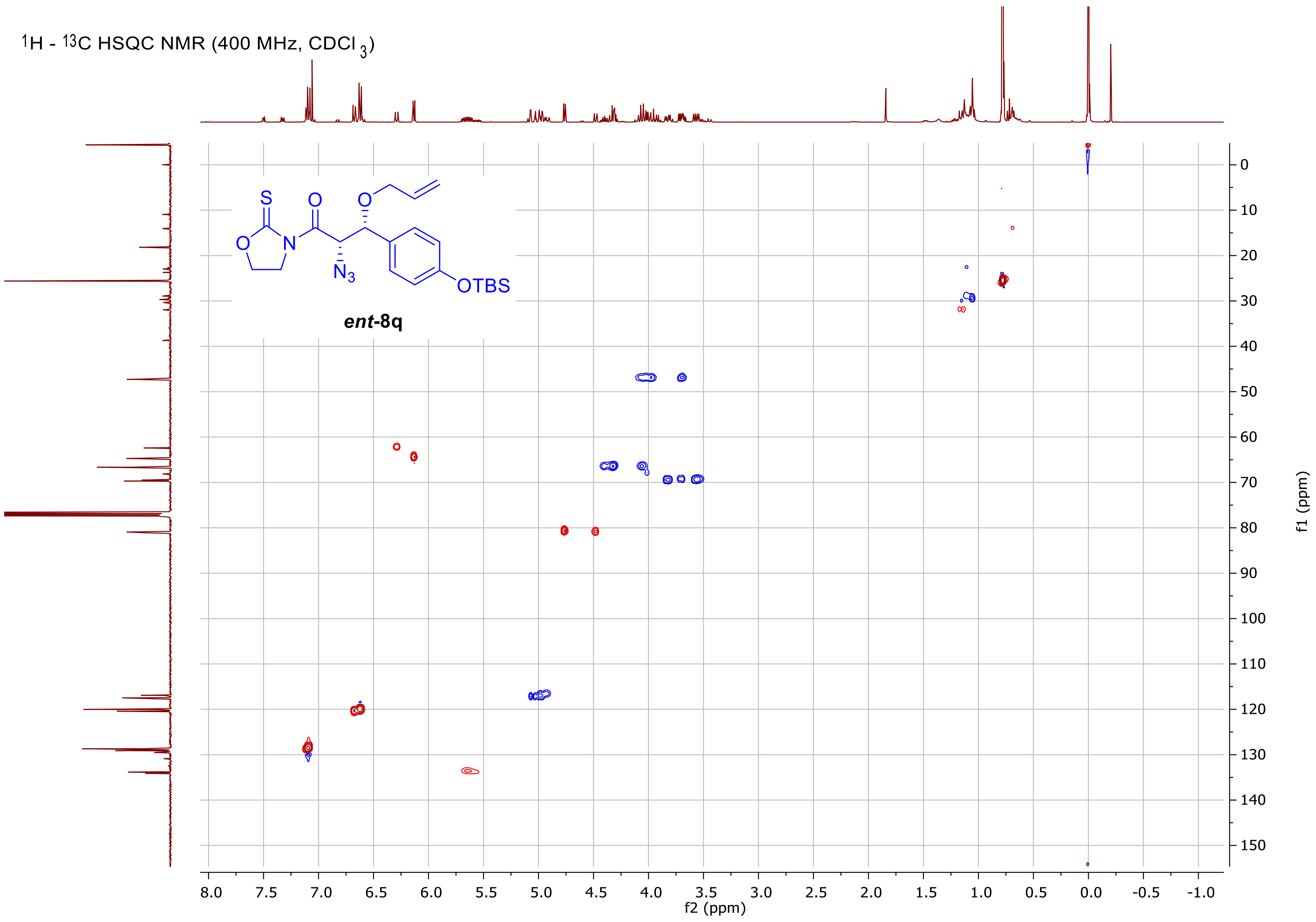

^1H NMR (400 MHz, $CDCl_3$)

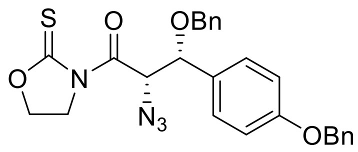

***ent*-6r**

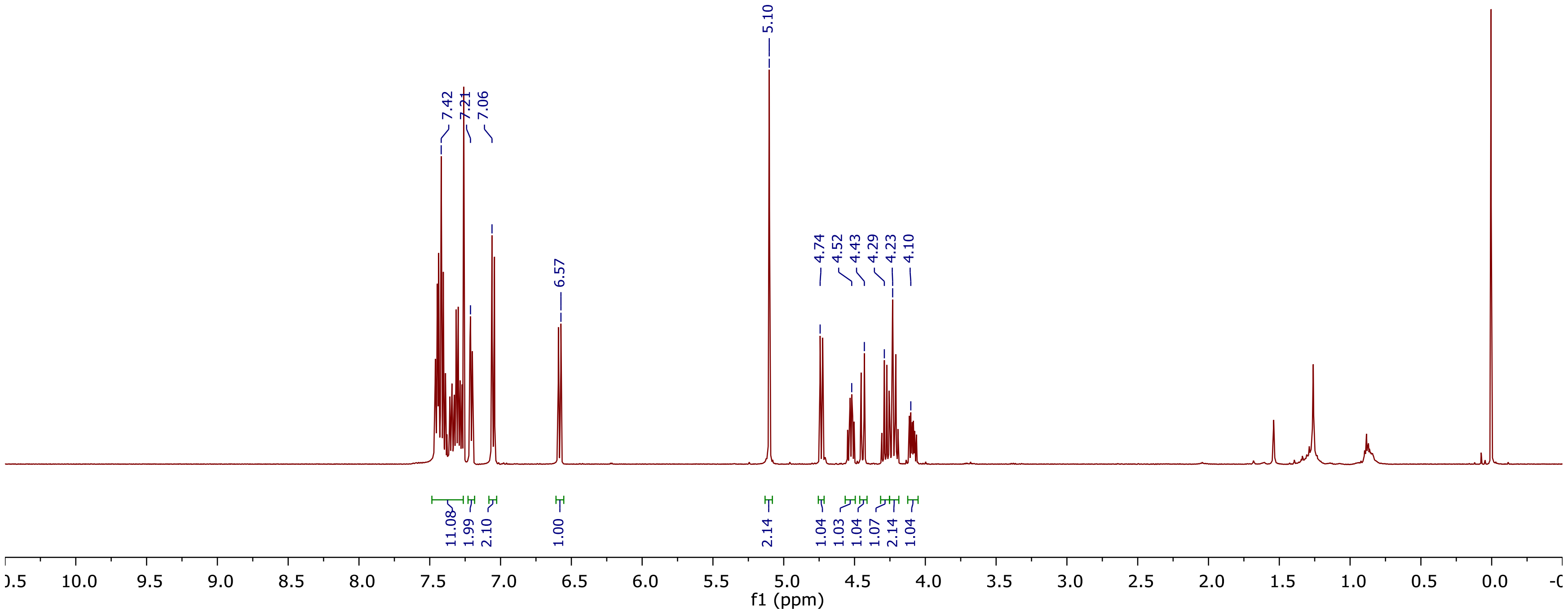

$^{13}C\{^{1}H\}$ NMR (100.6 MHz, $CDCl_3$)

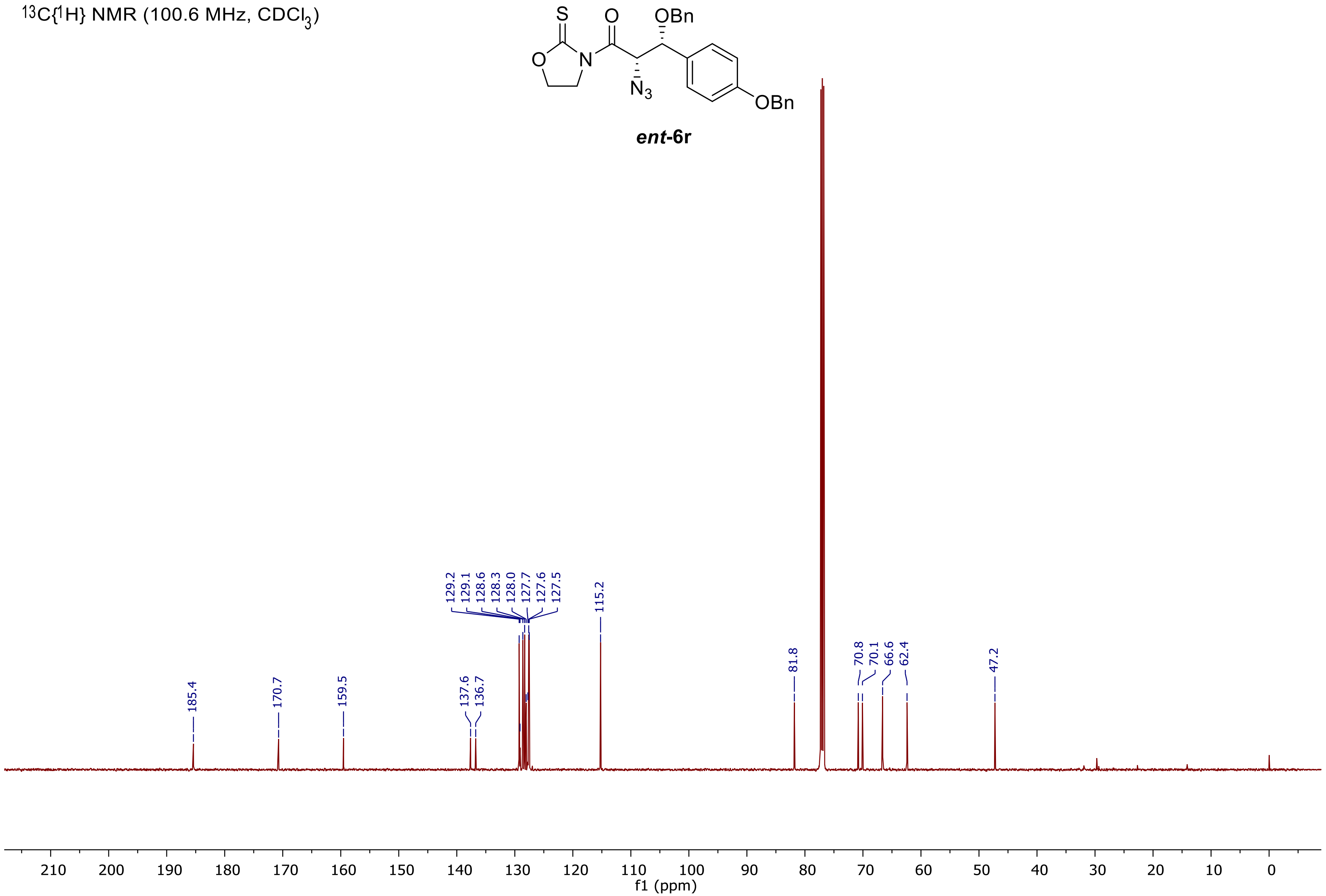

***ent*-6r**

^1H - ^1H COSY NMR (400 MHz, $CDCl_3$)

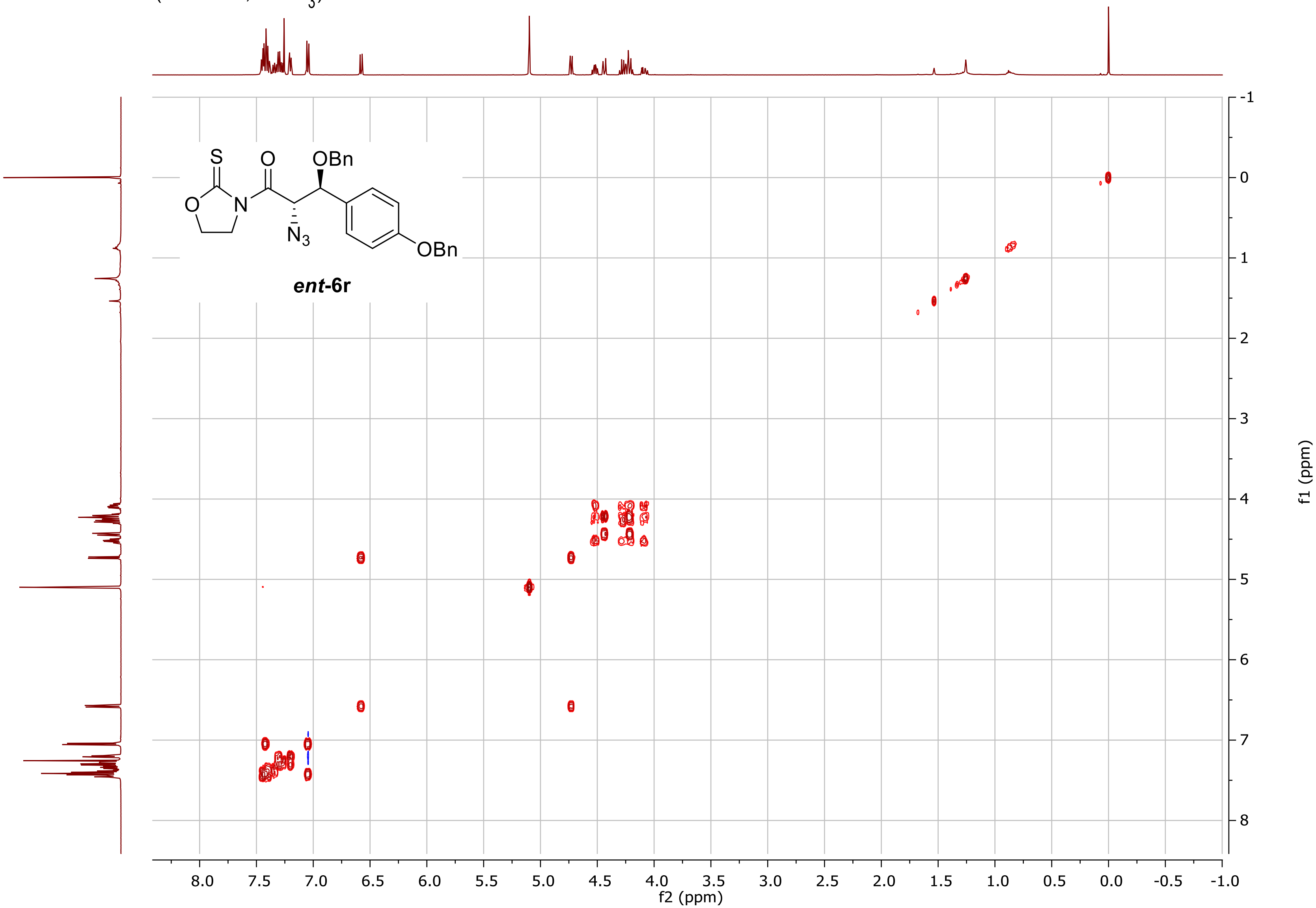

^{1}H - ^{13}C HSQC NMR (400 MHz, $CDCl_3$)

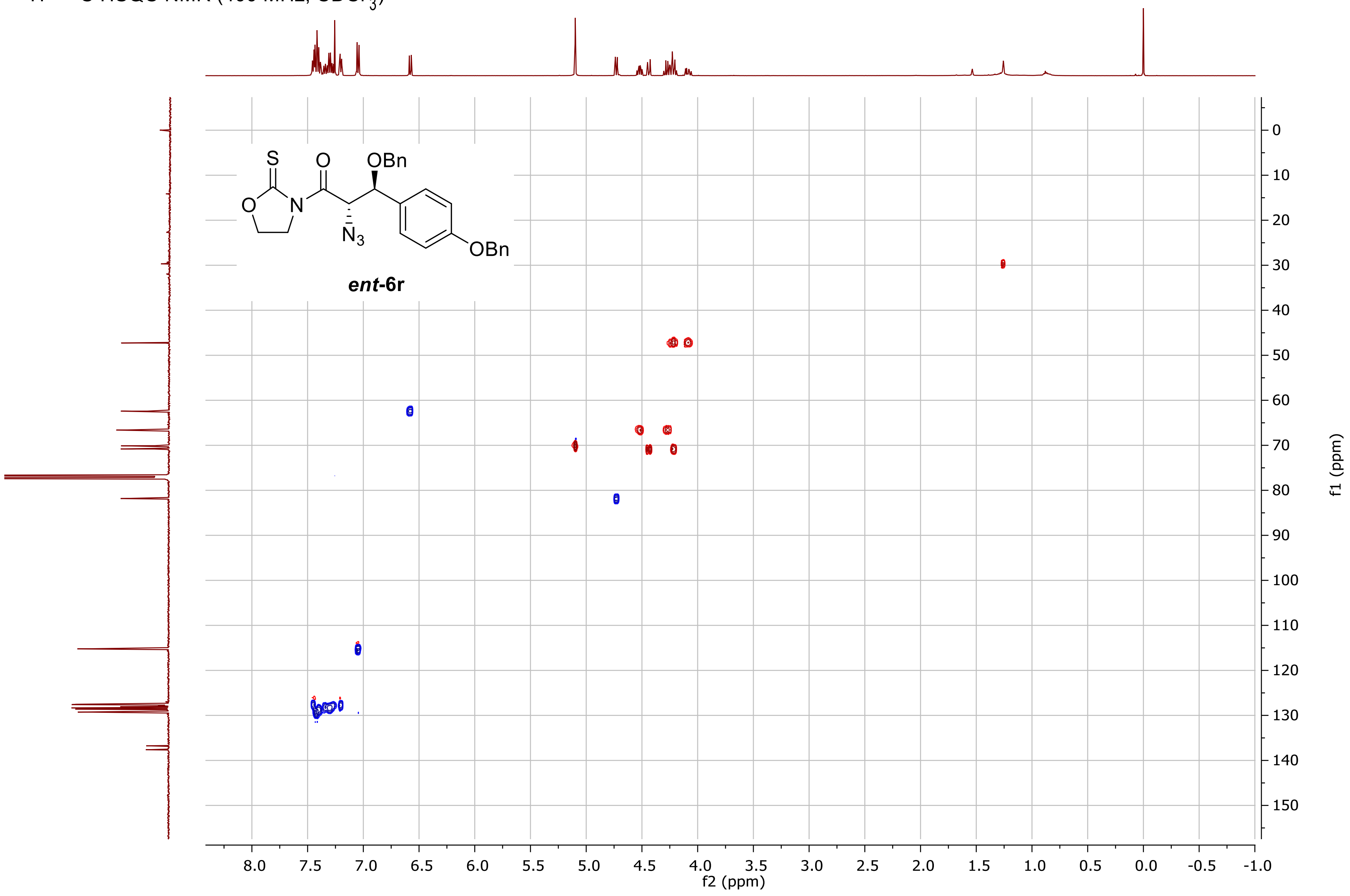

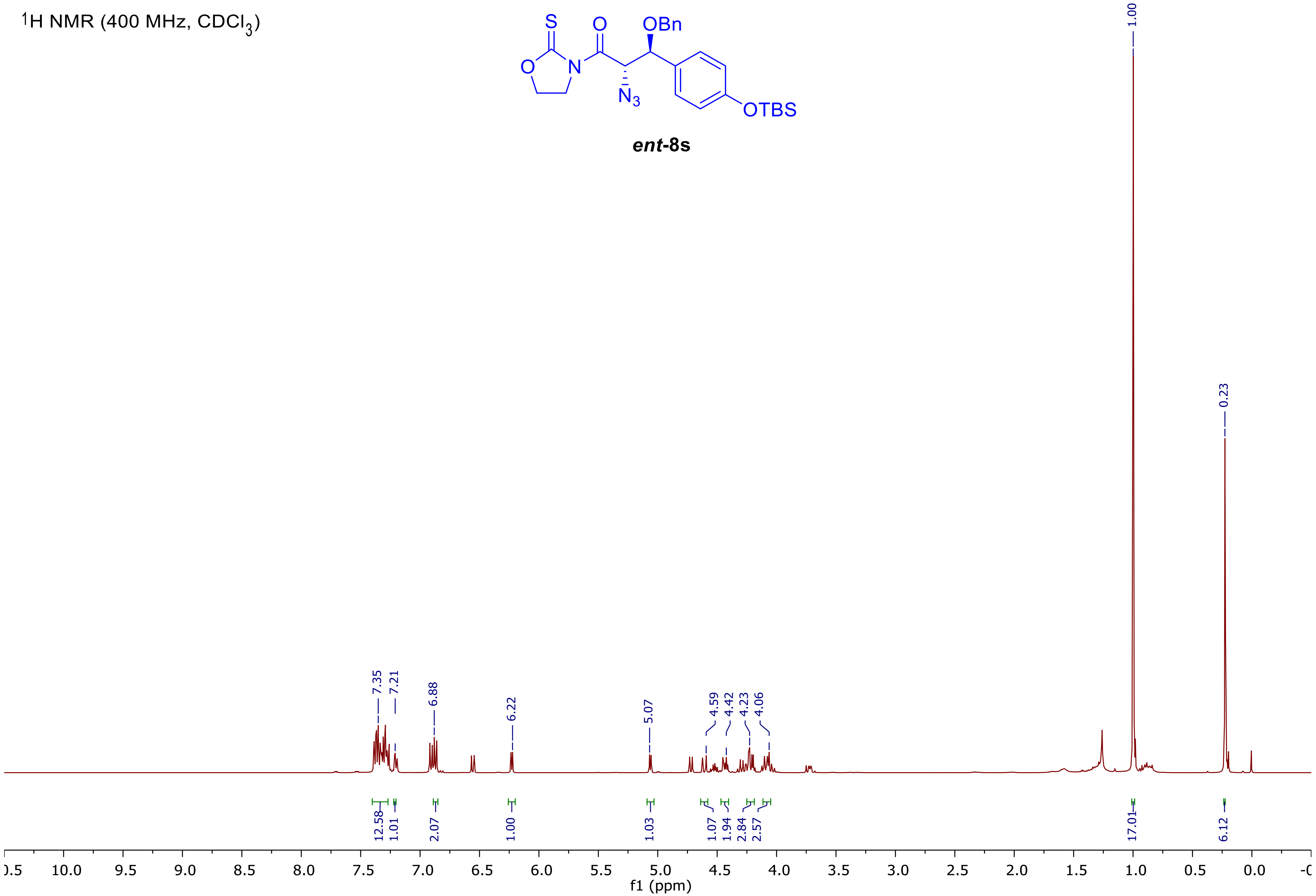

1H NMR (400 MHz, CDCl3)
S
O
OBn
O
N
N3
OTBS
ent-8s
7.35
7.21
6.88
6.22
5.07
4.59
4.42
4.23
4.06
1.00
0.23
12.58
1.01
2.07
1.00
1.03
1.07
1.94
2.84
2.57
17.01
6.12
10.0
9.5
9.0
8.5
8.0
7.5
7.0
6.5
6.0
5.5
5.0
4.5
4.0
3.5
3.0
2.5
2.0
1.5
1.0
0.5
0.0
f1 (ppm)

$^{13}C\{^1H\}$ NMR (100.6 MHz, $CDCl_3$)

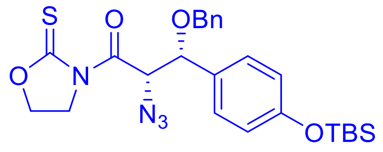

***ent*-8s**

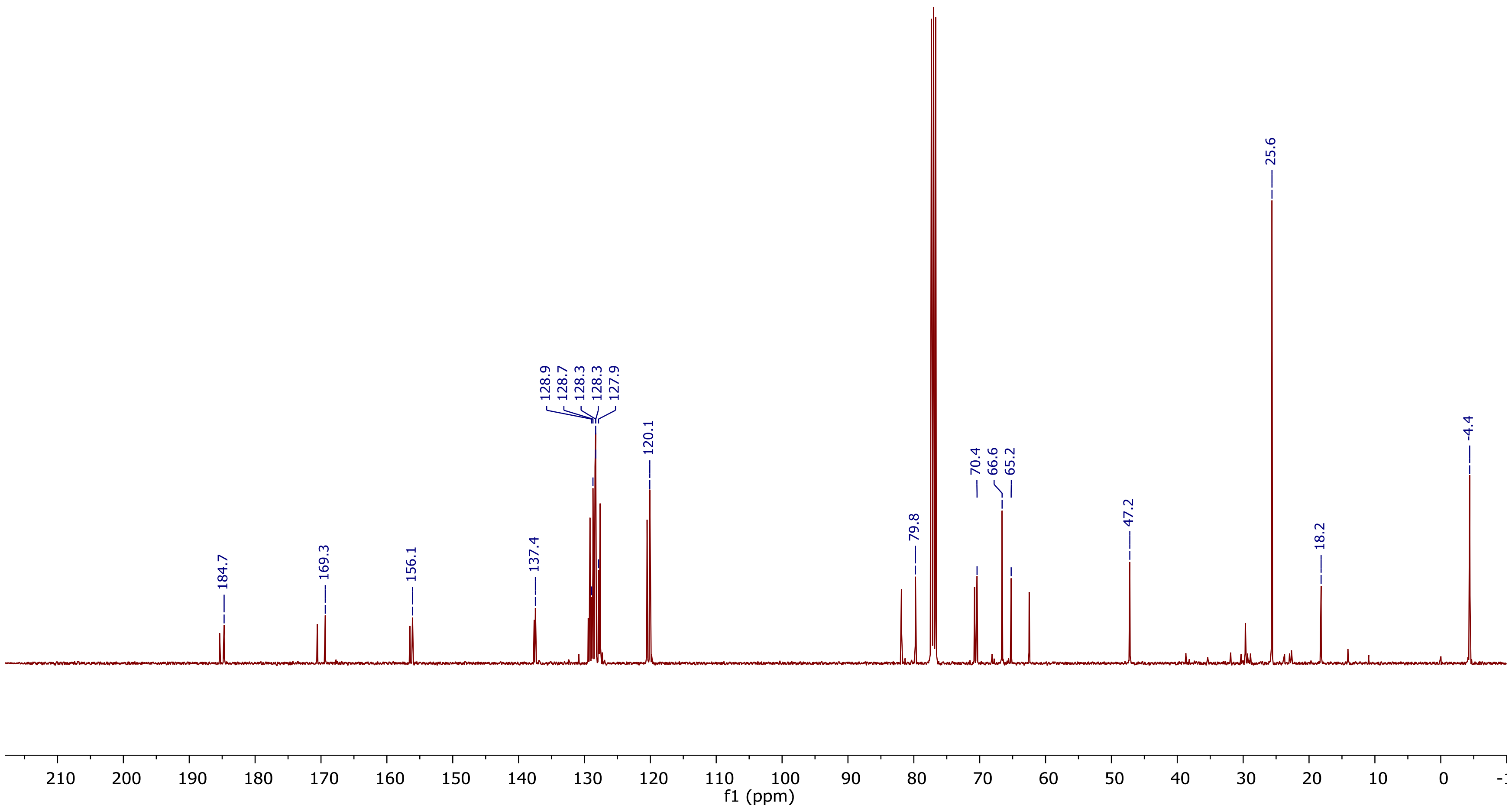

^{1}H - ^{1}H COSY NMR (400 MHz, $CDCl_3$)

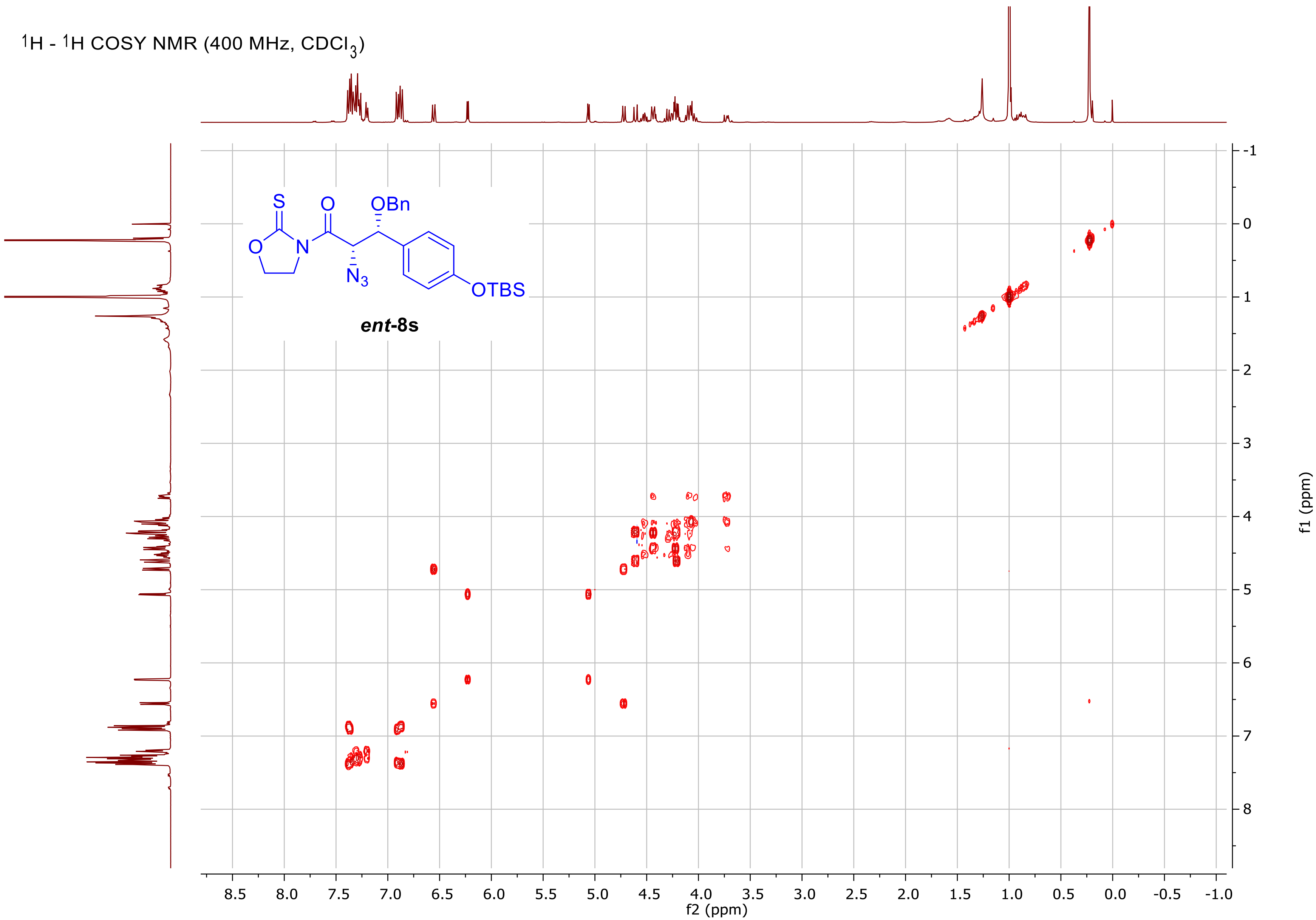

^{1}H - ^{13}C HSQC NMR (400 MHz, $CDCl_3$)

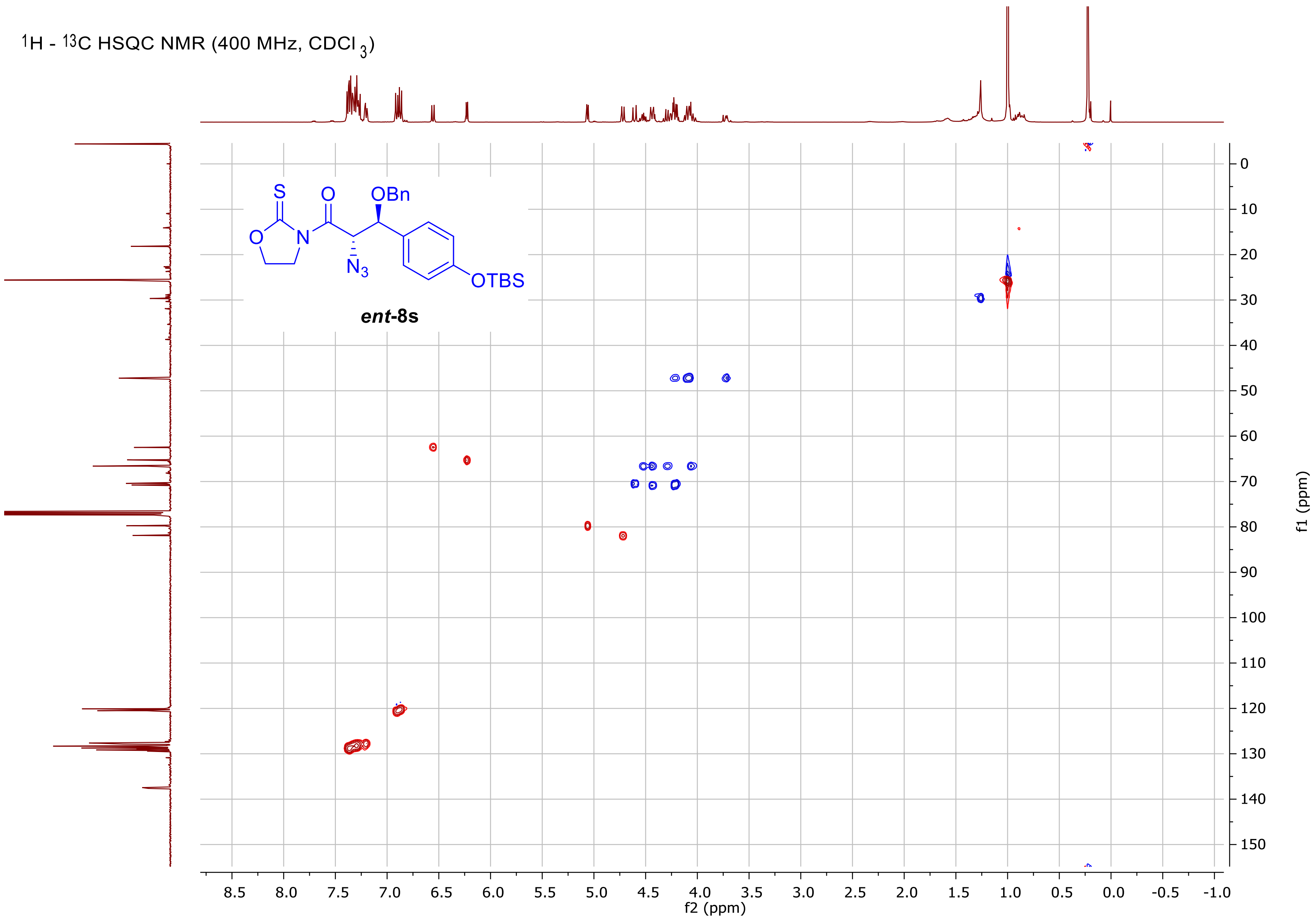

^1H NMR (400 MHz, $CDCl_3$)

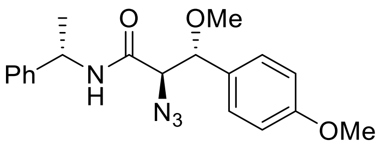

9

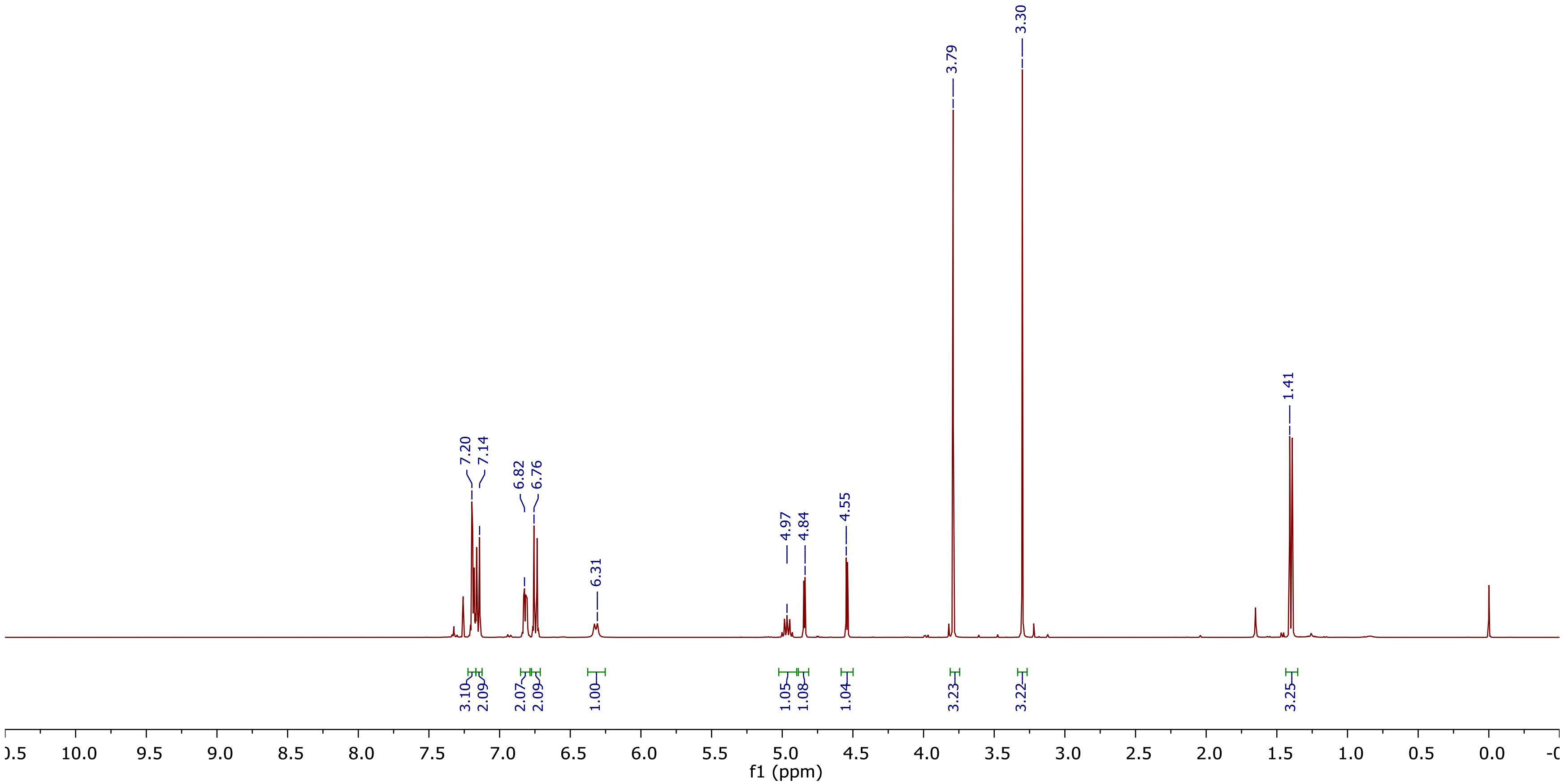

$^{13}C\{^{1}H\}$ NMR (100.6 MHz, $CDCl_3$)

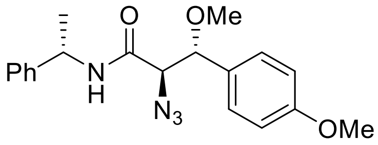

9

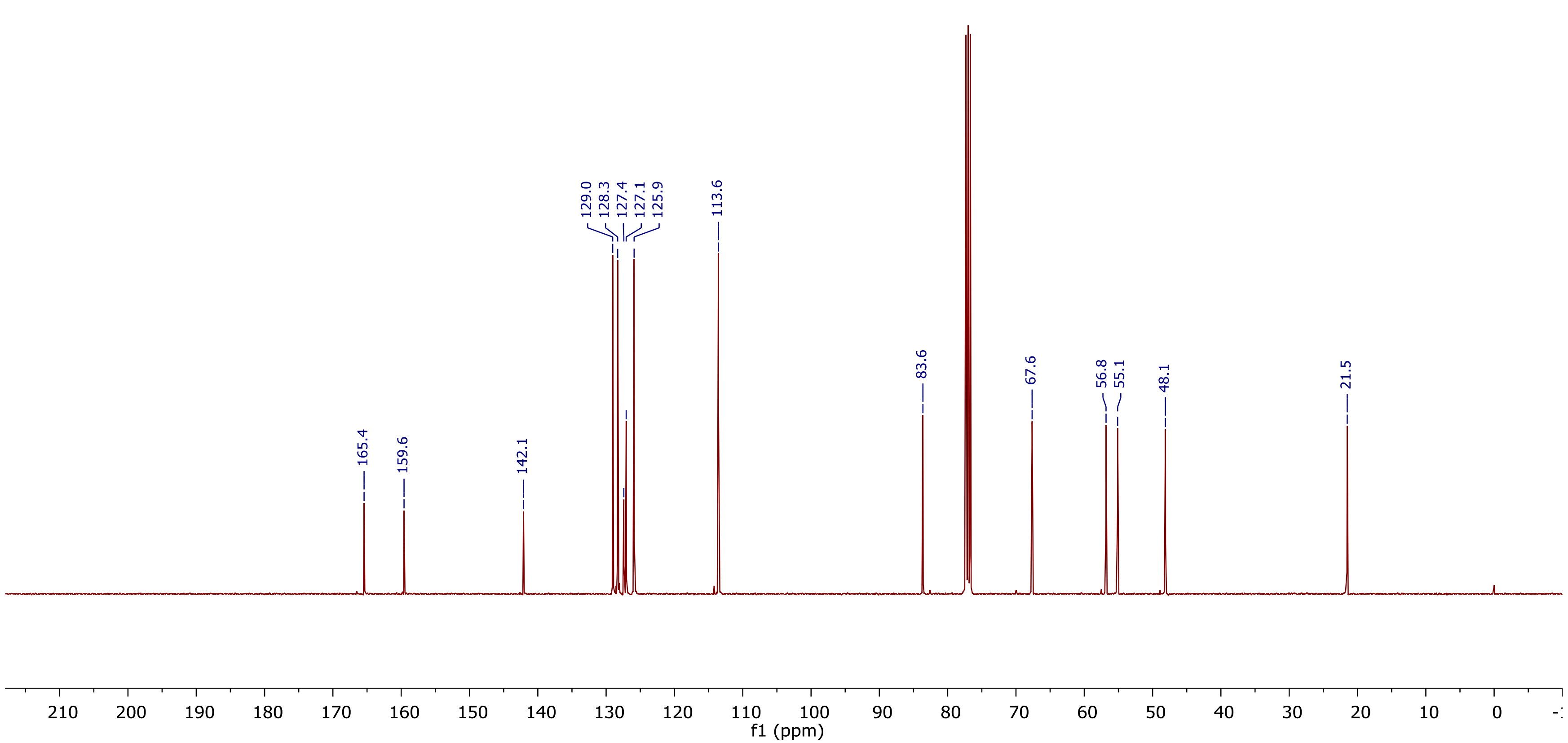

^1H - ^{13}C HSQC NMR (400 MHz, $CDCl_3$)

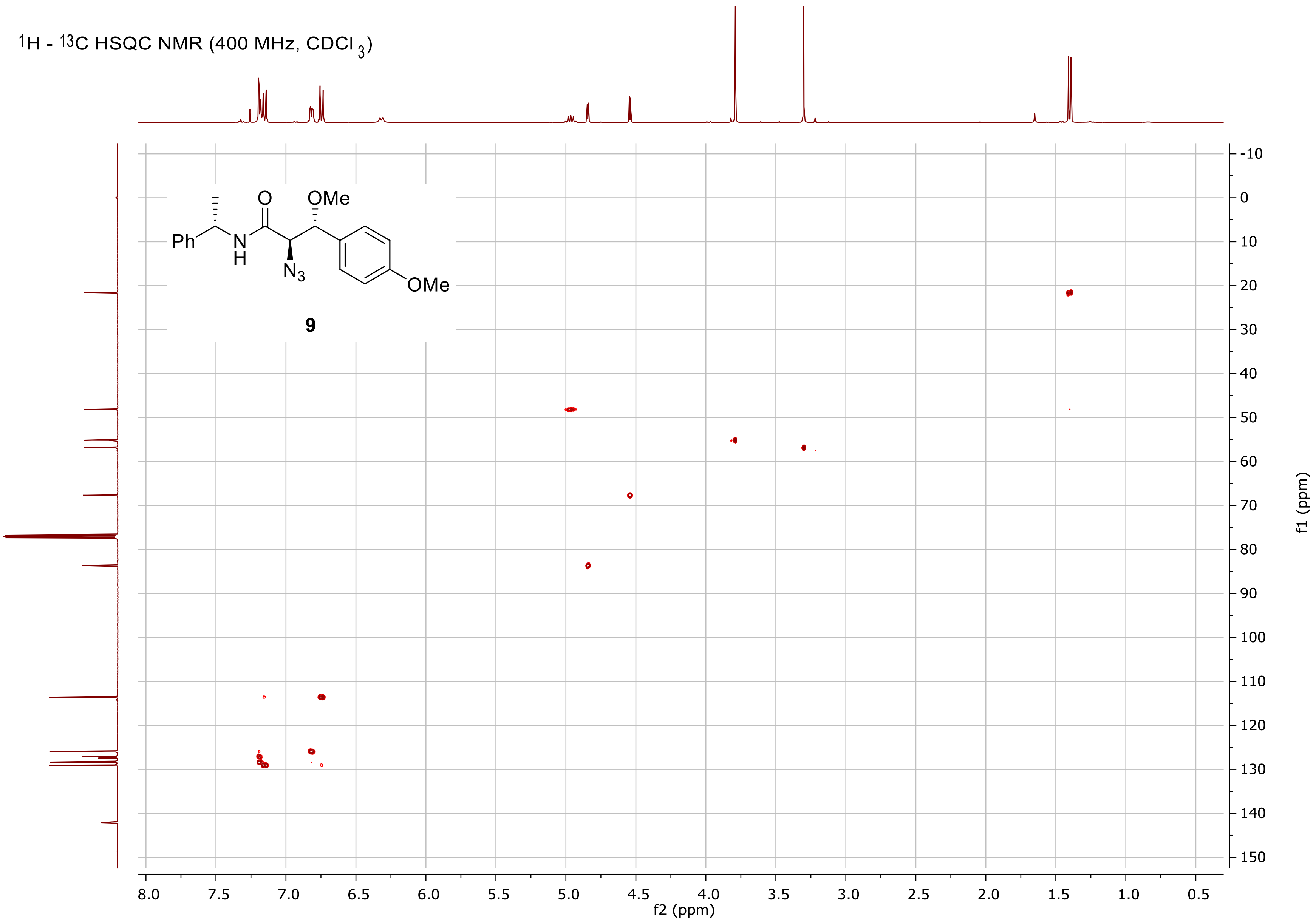

^{1}H NMR (400 MHz, $CDCl_3$)

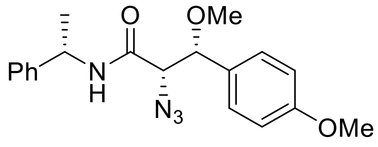

10

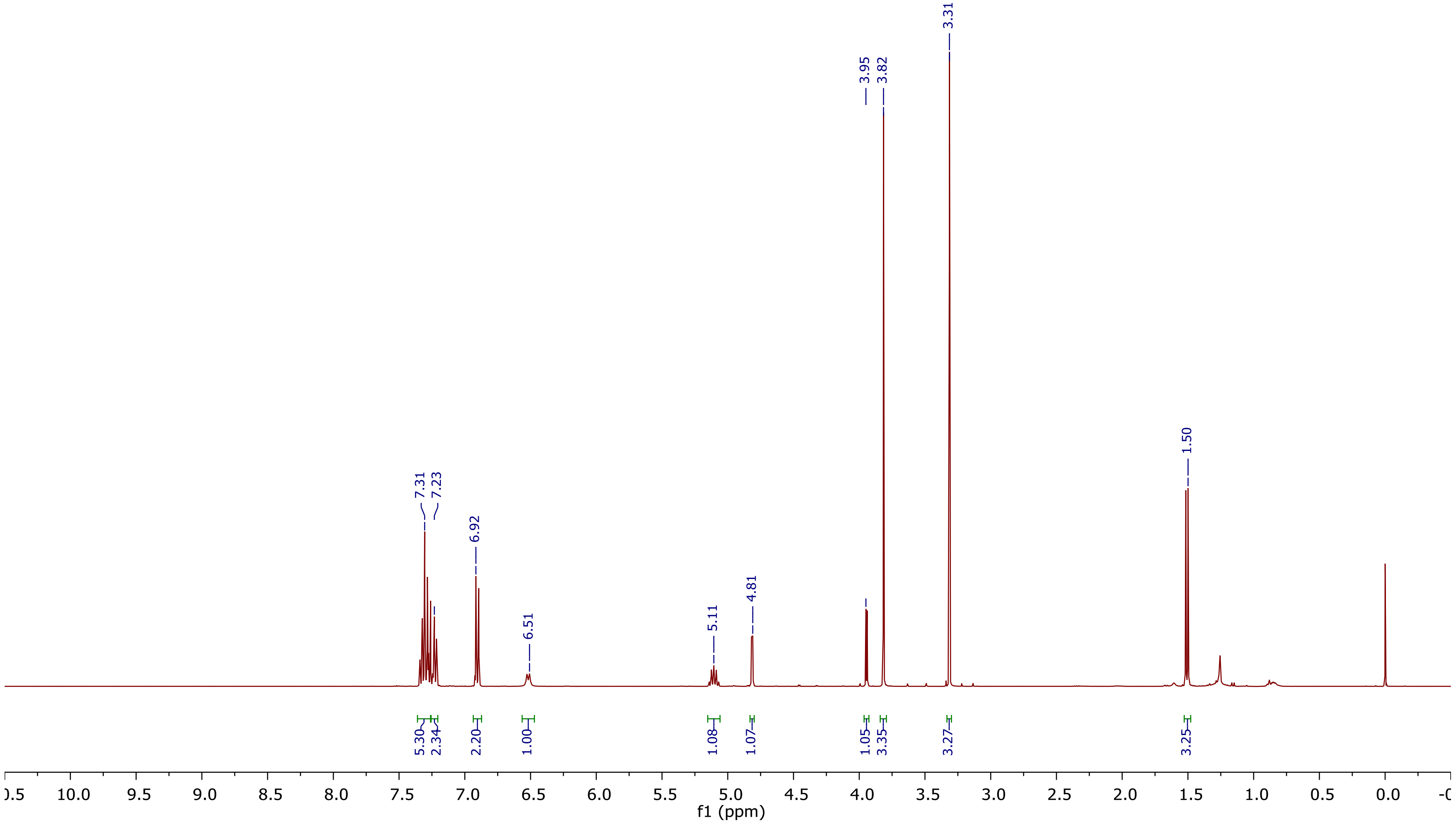

$^{13}C\{^{1}H\}$ NMR (100.6 MHz, $CDCl_3$)

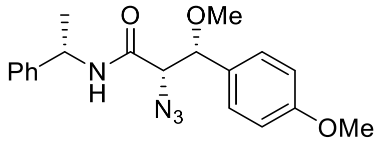

10

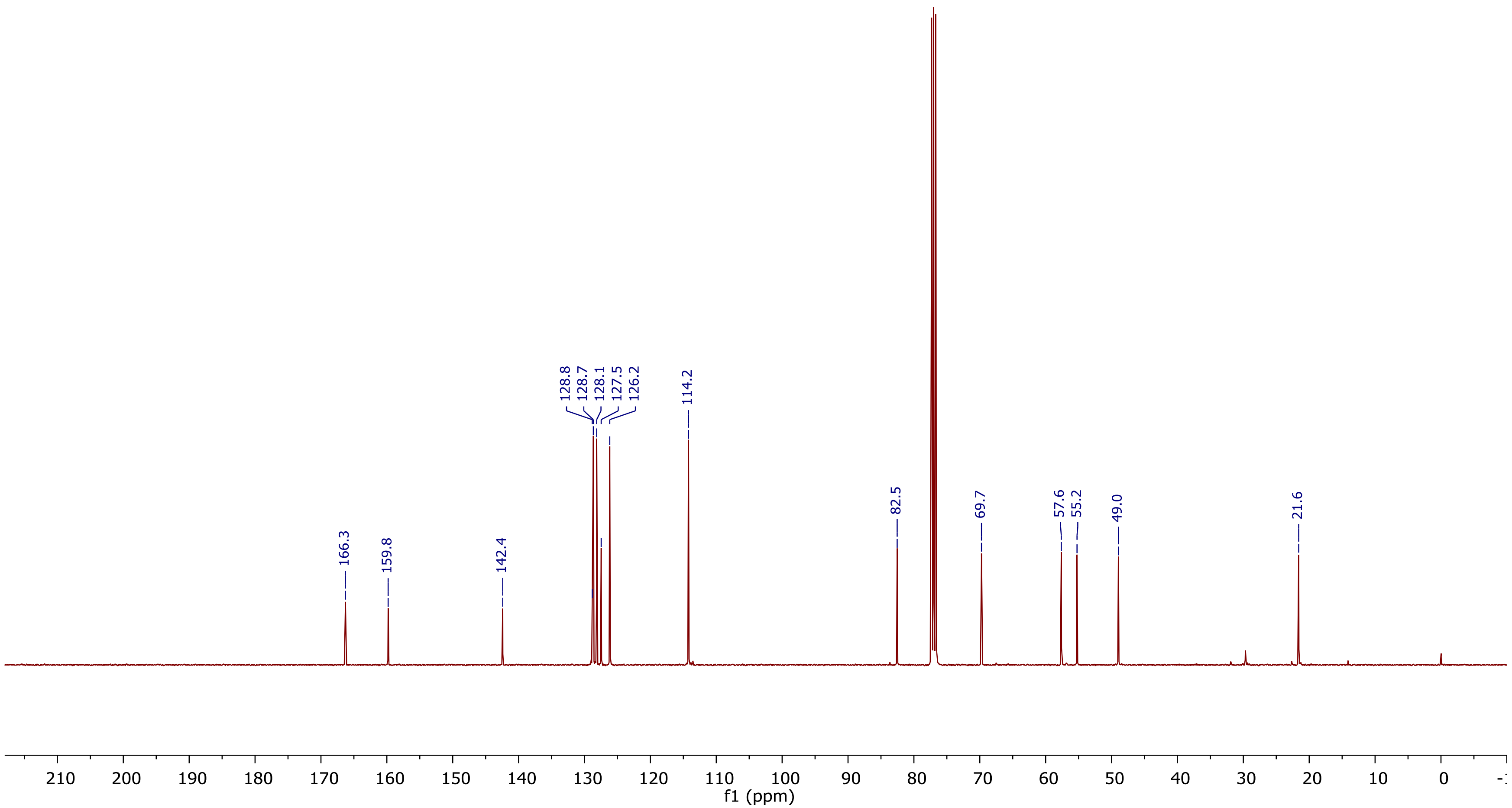

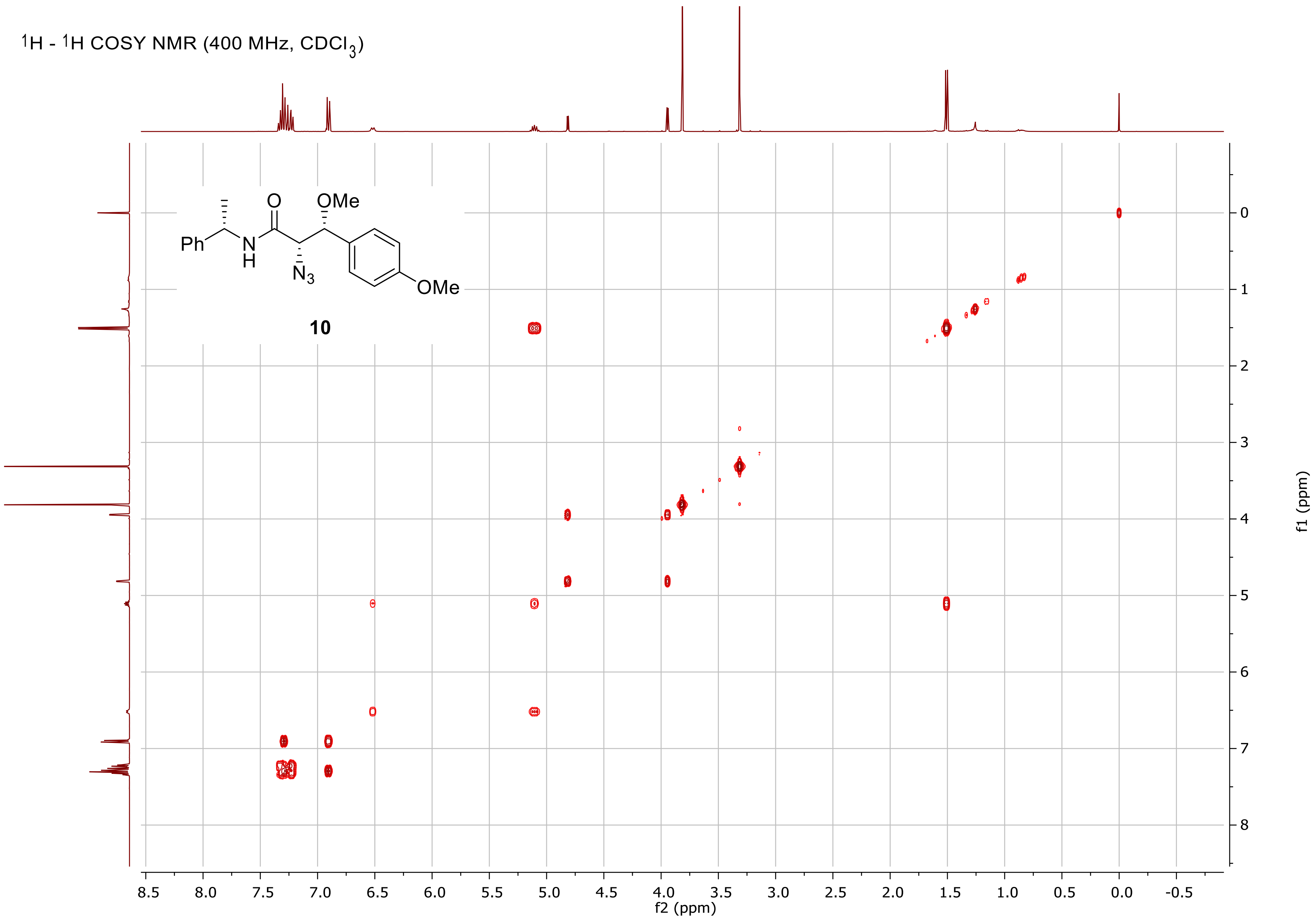

^1H - ^1H COSY NMR (400 MHz, $CDCl_3$)
OMe
O
Ph
N
H
N_3
OMe
10
f2 (ppm)
f1 (ppm)

^{1}H - ^{13}C HSQC NMR (400 MHz, $CDCl_3$)

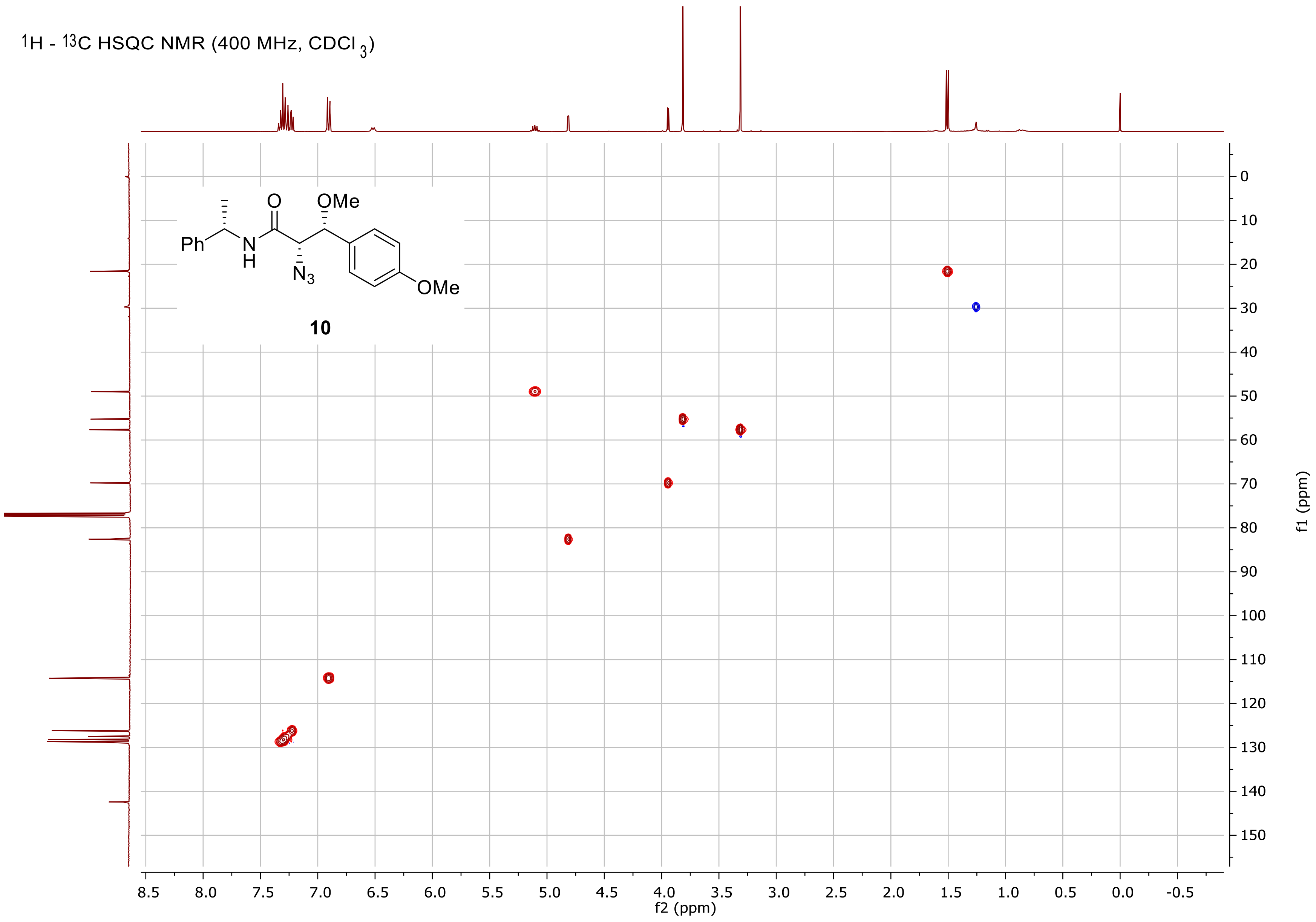

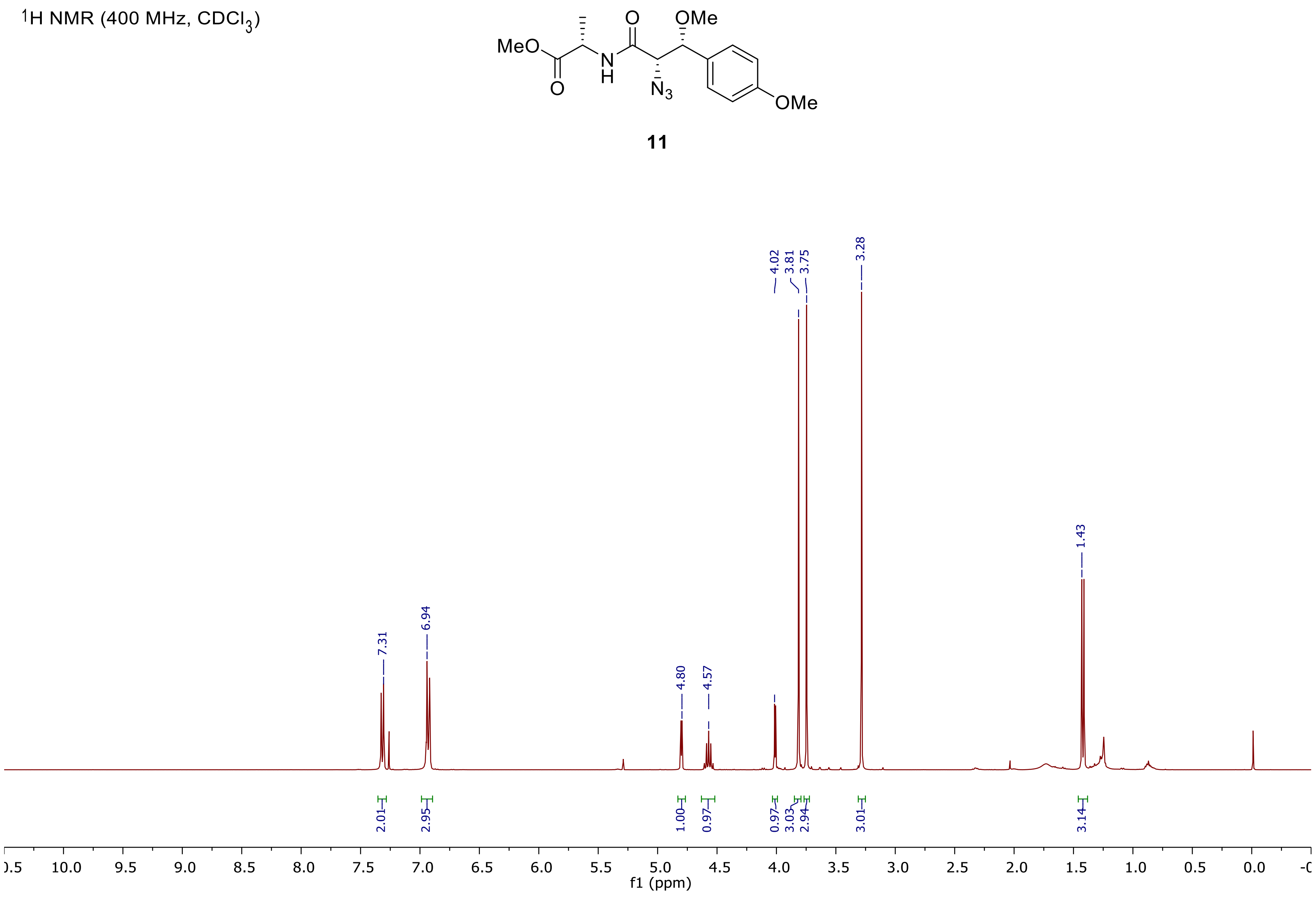

¹H NMR (400 MHz, CDCl3)
MeO
O
N
H
O
OMe
N3
OMe
11
7.31
6.94
4.80
4.57
4.02
3.81
3.75
3.28
1.43
2.01
2.95
1.00
0.97
0.97
3.03
2.94
3.01
3.14
f1 (ppm)

$^{13}C\{^{1}H\}$ NMR (100.6 MHz, $CDCl_3$)

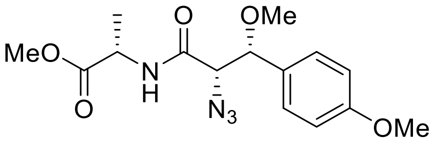

11

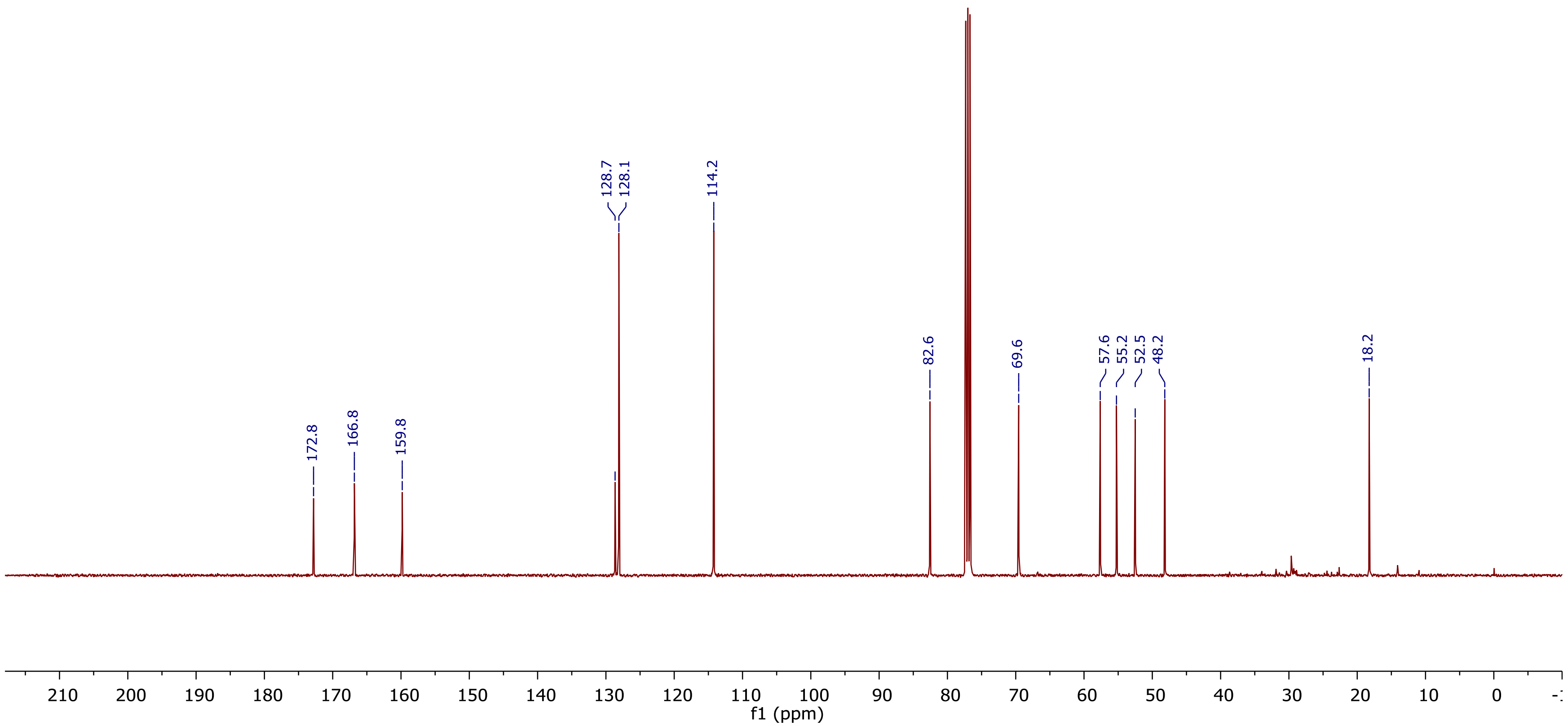

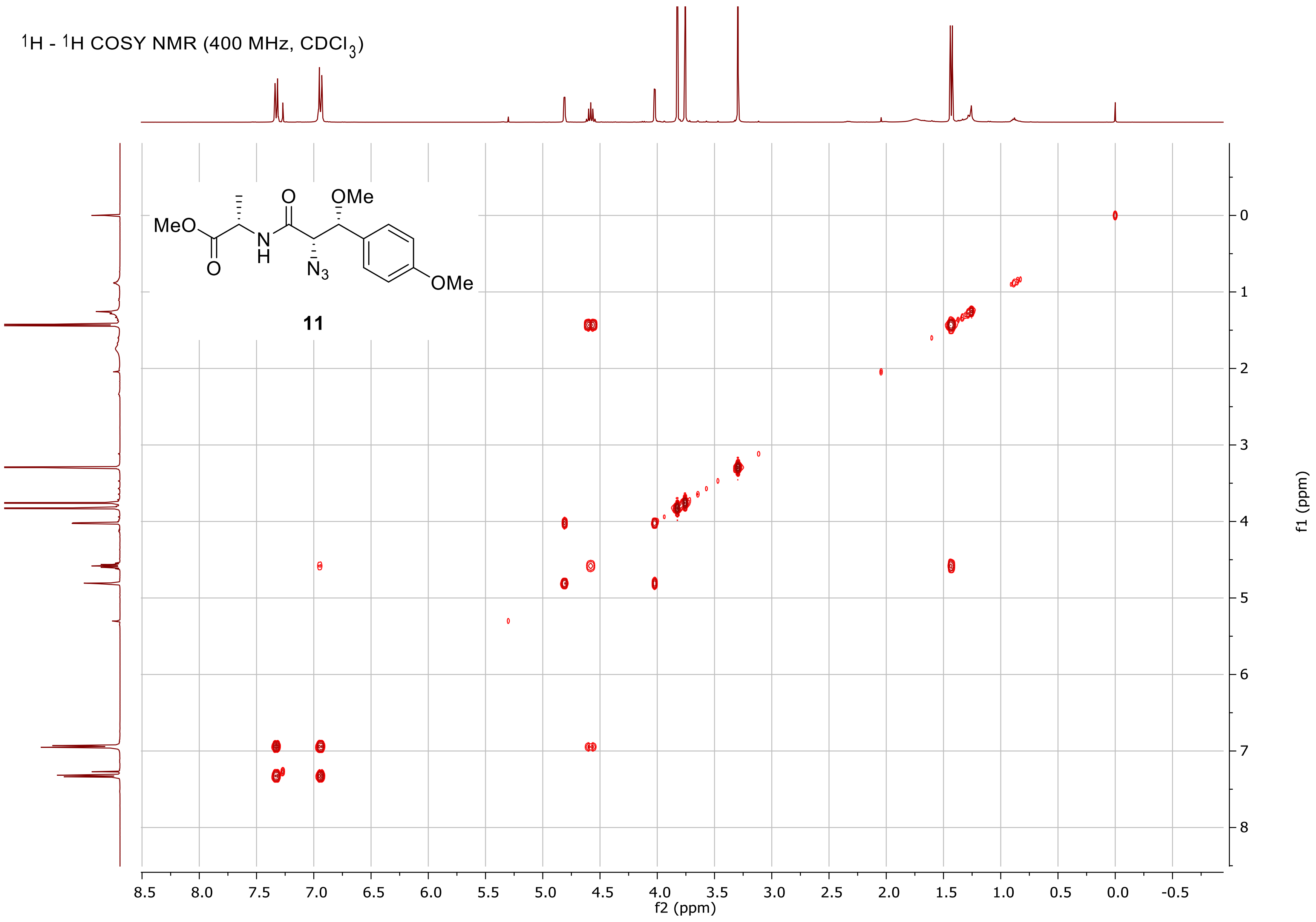

1H - 1H COSY NMR (400 MHz, CDCl3)
MeO
O
N
H
O
N3
OMe
OMe
11
f2 (ppm)
f1 (ppm)

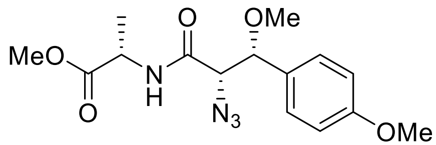

11

^{1}H - ^{13}C HSQC NMR (400 MHz, $CDCl_3$)

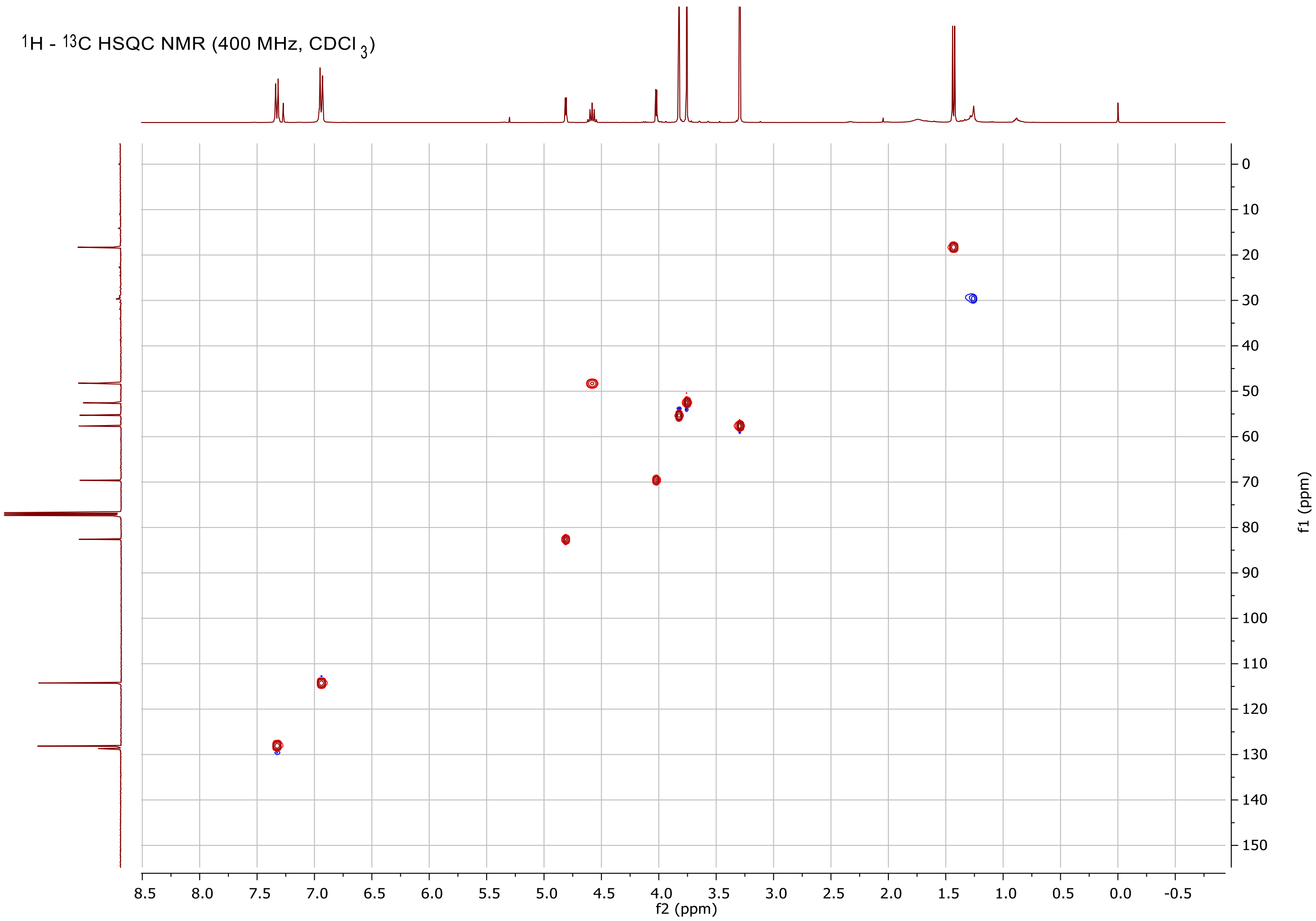

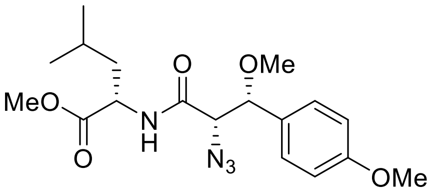

12

^{1}H NMR (400 MHz, $CDCl_3$)

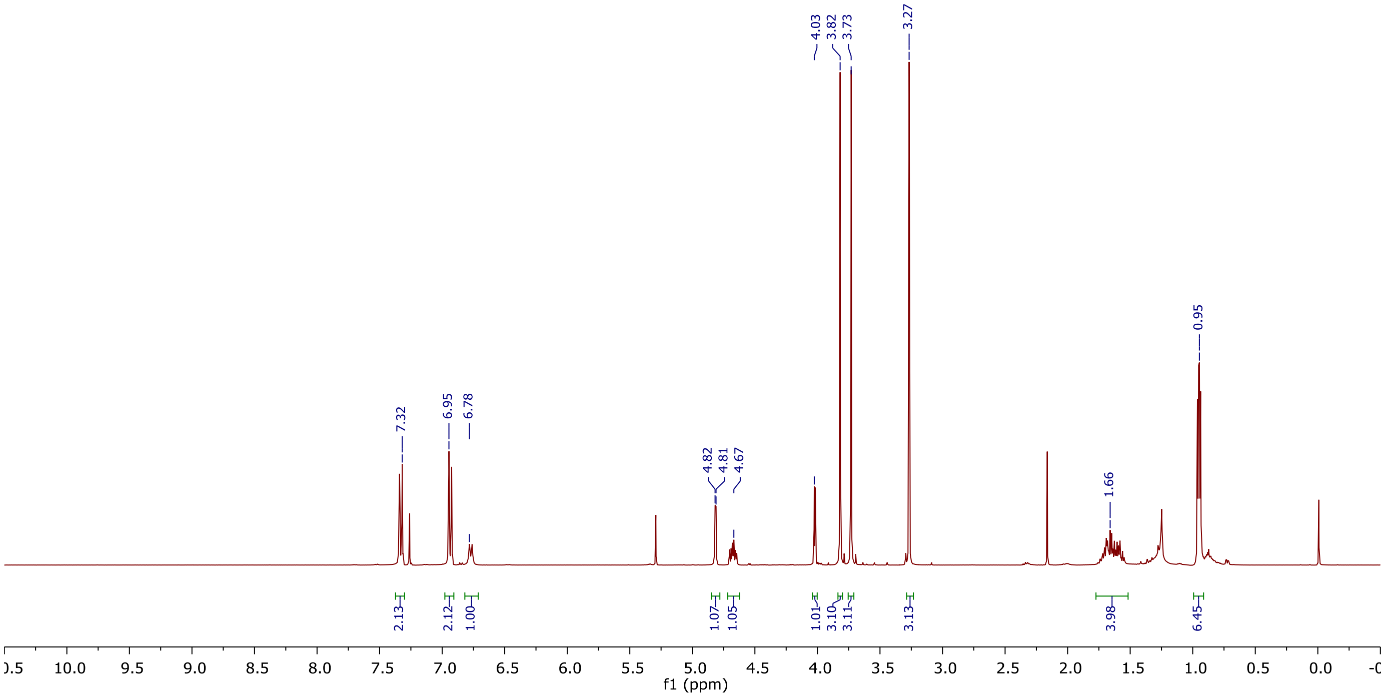

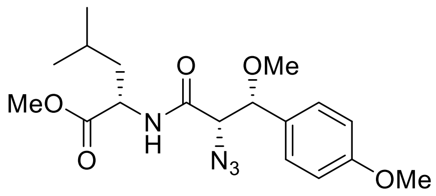

12

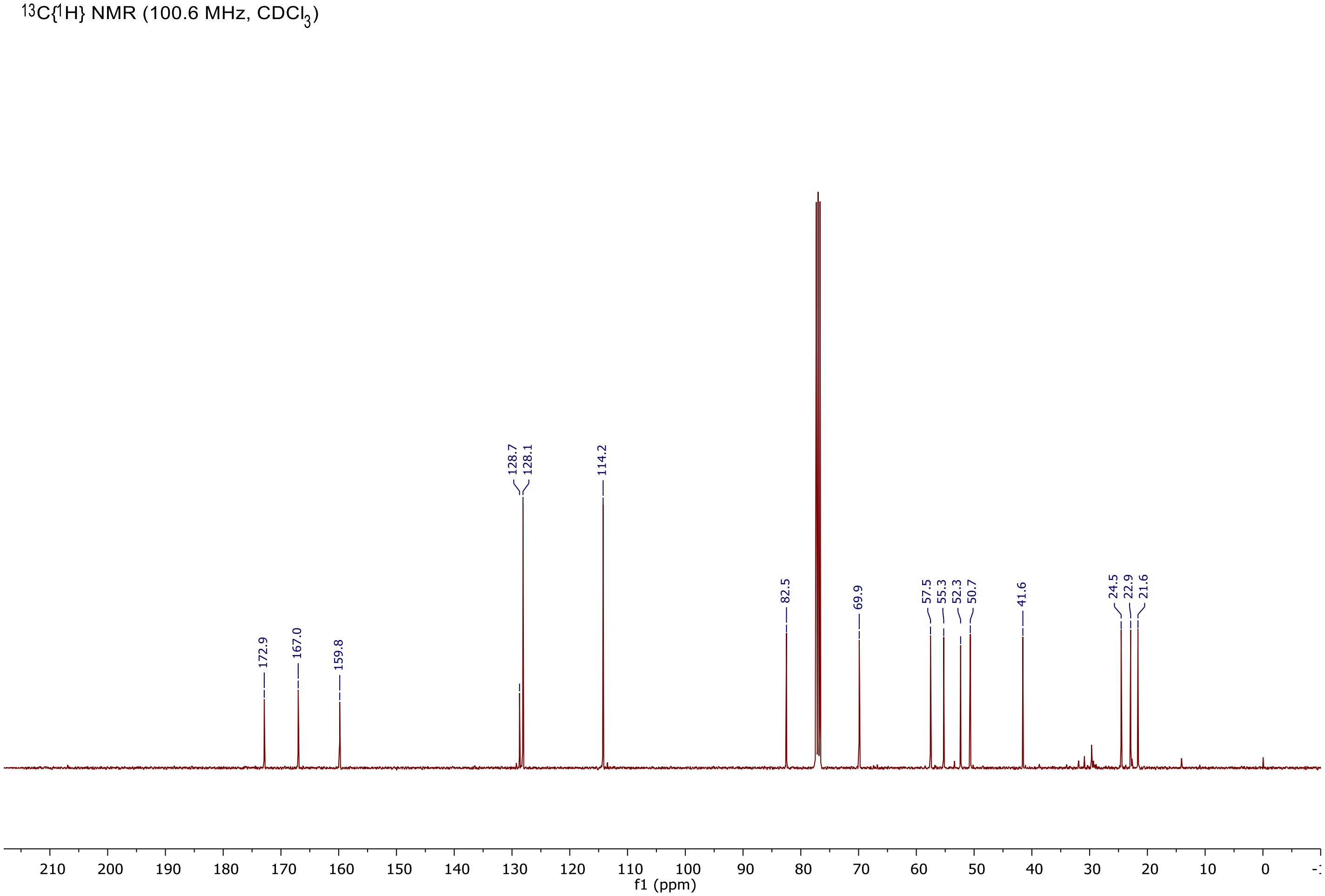

$^{13}C\{^1H\}$ NMR (100.6 MHz, $CDCl_3$)
172.9
167.0
159.8
128.7
128.1
114.2
82.5
69.9
57.5
55.3
52.3
50.7
41.6
24.5
22.9
21.6
f1 (ppm)

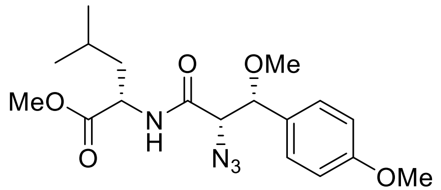

12

^1H - ^1H COSY NMR (400 MHz, $CDCl_3$)

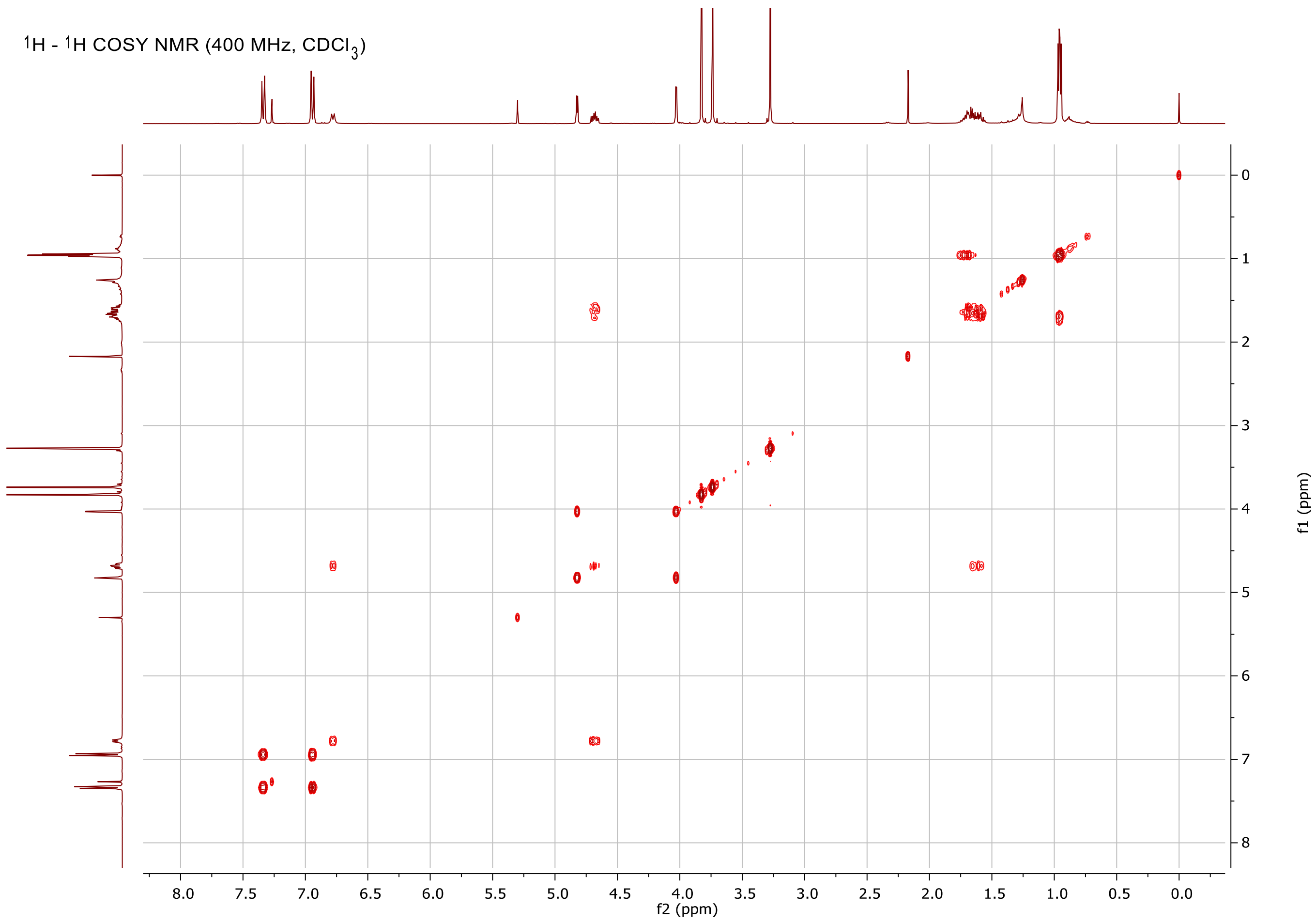

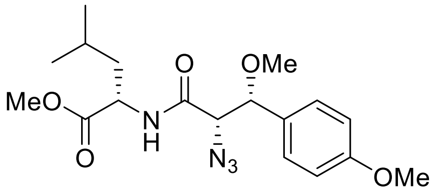

12

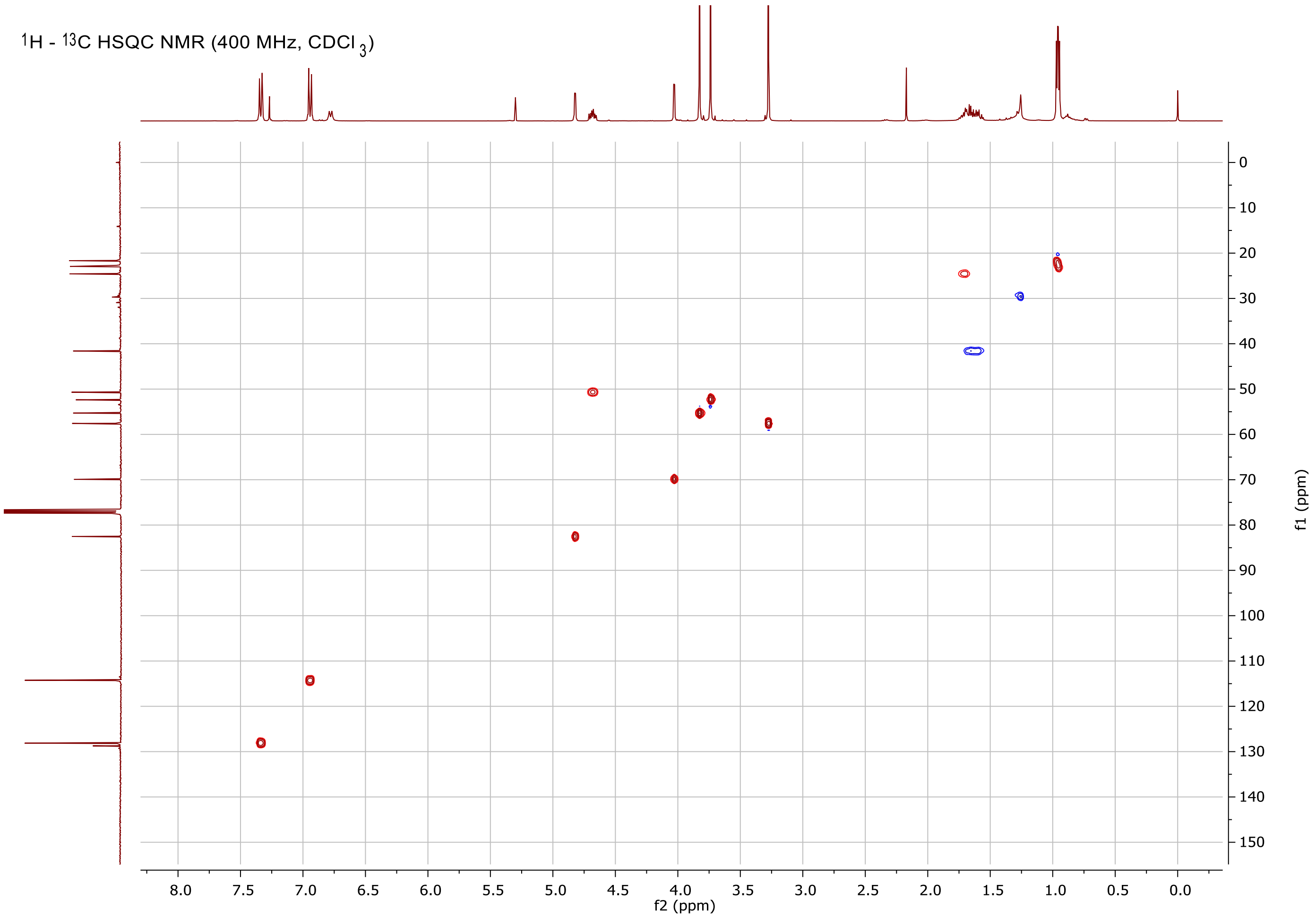

Supplement: Supplementary file 1 [file jo5c00390_si_001.pdf]
